# Author Correction: The rise of predation in Jurassic lampreys

**DOI:** 10.1038/s41467-023-44296-7

**Published:** 2023-12-14

**Authors:** Feixiang Wu, Philippe Janvier, Chi Zhang

**Affiliations:** 1 Key Laboratory of Vertebrate Evolution and Human Origins of Chinese Academy of Sciences, Institute of VertebratePaleontology and Paleoanthropology, Chinese Academy of Sciences, 100044 Beijing, China; 2 Muséum national d’Histoire naturelle, UMR 7207, CP38, 8, rue Buffon 75231, Paris, Cedex 05 France

**Keywords:** Palaeontology, Ichthyology, Biogeography, Phylogenetics

Correction to: *Nature Communications* 10.1038/s41467-023-42251-0, published online 31 October 2023

The original version of this Article contained errors in the name of the locality of the holotype of *Yanliaomyzon occicor*. In the Results, the **Horizon and locality** for *Yanliaomyzon occisor* incorrectly read “Tiaojishan Formation, Oxfordian, earliest Late Jurassic, ca. 158.58–160 million years ago (Ma)^19^; Daxishan, Linglongta Town, Jianchang County, Liaoning Province (Holotype), and Nanshimen Village, Gangou Town, Qinglong County, Hebei Province (Paratype), China.” The correct version states ‘Toudaoyingzi Town’ in place of ‘Linglongta Town’. The Methods subsection **Fossil Specimens** incorrectly read “The specimens of *Yanliaomyzon occisor* gen. et sp. nov., IVPP V 15830 comes from the Tiaojishan Formation, earliest Late Jurassic, ca. 158.58–160 Ma^19^ of Daxishan, Linglongta Town, Jianchang County, Liaoning Province, and IVPP V 18956A, B from the corresponding layers of Nanshimen Village, Gangou Town, Qinglong County, Hebei Province, China.” The correct version states ‘Toudaoyingzi Town’ in place of ‘Linglongta Town’. This has been corrected in both the PDF and HTML versions of the Article.

The original version of the Reporting Summary associated with this Article also incorrectly read ‘Linglongta Town’ for the locality of the holotype of *Yanliaomyzon occicor*. The correct version states ‘Toudaoyingzi Town’ in place of ‘Linglongta Town’. The HTML has been updated to include a corrected version of the Reporting Summary.

## Reporting summary

Further information on research design is available in the Nature Portfolio Reporting Summary linked to this article.

### Supplementary information

Updated Reporting Summary

