# Supplementary material for: The rise of predation in Jurassic lampreys
Source: Nat Commun. 2023 Oct 31;14:6652. doi: 10.1038/s41467-023-42251-0 (PMC10618186; doi:10.1038/s41467-023-42251-0)
Supplement: Supplementary file 1 — Supplementary Information [file 41467_2023_42251_MOESM1_ESM.pdf]

The Supplementary Information for:

The rise of predation in Jurassic lampreys

Feixiang Wu*[1], Philippe Janvier[2], Chi Zhang*[1]

1 Key Laboratory of Vertebrate Evolution and Human Origins of Chinese Academy of Sciences, Institute of Vertebrate Paleontology and Paleoanthropology, Chinese Academy of Sciences, Beijing 100044, China

2 Museum National d'Histoire Naturelle, UMR 7207, CP38, 8, rue Buffon 75231, Paris Cedex 05, France

* Corresponding authors

The supplementary information include:

1. Supplementary Notes 1-2
2. Supplementary Discussion
3. Supplementary References

Other supplementary materials include:

4. Supplementary Figures 1–3 and Legends
5. Supplementary Table 1

1. Supplementary Notes

1-1 Supplementary Note 1

Description and comparison

The cartilages of the skull can be roughly distinguished according to their imprints on the matrix, which can be divided into the anterior dorsal cartilage (adc) and posterior dorsal cartilage (pdc) (Supplementary Fig. 1a) just as in living lampreys[1]. In the paratype of *Yanliaomyzon occsior* there is a length of a black line preserved in front of the eyes which is possibly the trace of the ophthalmic ramus of trigeminal nerve (V1?) (Fig. 1c, d) according to the anatomy of living and other fossil lampreys[2]. The eyes are oval in shape with the longer longitudinal axis slightly inclined. The olfactory organ (ns) left a clear round imprint in the holotype of *Yanliaomyzon ingensdentes* in the position slightly in front of the level of the eyes (Fig. 1f, Supplementary Fig. 1a); however, more details of the nasal sac cannot be distinguished.

The feeding structures, including the oral disc, keratinous teeth, laminae surrounding the oral aperture and on the gouging tongue are superbly preserved current materials (Figs. 1, 2a–f, Supplementary Figs. 1b–d, g–i, 2h–l). The closely arranged disc teeth are much smaller than the circumoral teeth and decrease in size outwards. The morphology and arrangement of the disc teeth are very similar to the situation in the feeding adult *Geotria australis* (figs. 2–4, and 8 in ref.[3]) but different from the counterparts in the Patagonian lamprey, *Geotria macrostoma*, whose disc teeth are much more numerous and pointed in shape[4]. Fifteen and thirteen circumoral teeth are preserved in *Y. occisor* and *Y. ingensdentes,* respectively, which also lacks the posterior series. Considering the morphology of the teeth and the arrangement pattern of this teeth series, totally 16 and 23 circumoral teeth can be estimated for *Y. occisor* and *Y. ingensdentes,* respectively. They are generally trihedral in shape and each bear three edges of different lengths that forma tip pointing slightly backward and inward. These anterior and lateral circumoral teeth are therefore stronger and more complicated in structure than their counterparts in *Geotria*[3–5]. In the circumoral series, teeth show some morphological variations. The supraoral lamina is very large and roughly corresponds to 11 circumoral teeth in *Y. ingensdentes* and even encircles most of the circumference of the oral aperture in *Y. occisor*. Again, it closely resembles that of *Geotria*[3–5] in having a pair of central inner cusps flanked by a pair of large and curved wings. The inner bicuspid cusps both bear a posterior blade. Apart from the relative size, the supraoral lamina of *Y. occisor* is different from that of *Y. ingensdentes* in having an accessorial projection located immediately laterally to the central cuspid (Figs. 1b, 2a–c, f, g). This lamina in both fossil species bears an anterior recessed part, analogous to that in *Geotria* just behind the thick annular muscle (fig. 4e in ref.[5]). The infraoral lamina is wide, convexly curved and bears a straight row of about ten stout cusps, of which the central ones are slightly larger than the lateral ones. In proportion, the infraoral lamina in *Y. occisor* is larger than that in *Y. ingensdentes.* The transverse lingual lamina is extraordinarily large. In *Y. ingensdentes* it almost fills up the whole oral aperture. On

the anterior semicircle of the periphery of the oral disc, there are some triangular imprints of the oral papillae (op) and ca. 30 comb-like imprints of oral fimbriae (of) and numerous irregular oral papillae preserved in IVPP V16715 (*Y. ingensdentes*) (Figs. 1g, 2e, Supplementary Fig. 1b, c). In *Y. occisor*, two anterolateral oral papillae appear to be larger than the others (Figs. 1b, 2f, Supplementary Fig. 2b, k).

The prebranchial region and branchial apparatus left clear imprint on the slabs, with the former is longer than the latter (Fig. 1c–e, f, h). In the presumed position of the junction of the esophagus and respiratory tube, there is an irregularly shaped imprint which might be associated with the velum (vl). In the presumed position of the notochord, there is always a dark band suggesting the pathway of the dorsal aorta (da) and its accompanying vessels. Some faint imprints of the piston can be spotted. The anteriormost part of the gill apparatus is located behind the otic capsule (Fig. 1c, d, f). Immediately behind the branchial apparatus, the pericardial cartilage (pc) left a semicircular depression. Behind the position of this cartilage is an orange area that was likely caused by the liver (lv) as in some specimens of the Cretaceous lamprey *Mesomyzon*[2].

As for the fins of lampreys, here we, as proposed somewhere else[2,6], reinterpret the terminology of the dorsal fin (adf) and caudal fin (cf) according to possible homology and their topological relation to the cloaca (ca), which usually marks the boundary of the abdominal and caudal regions in lower vertebrates[7]. In *Yanliaomyzon ingensdentes*, the dorsal fin is contiguous with the anterior lobe of the caudal fin (pdf) (this is probably also the case in *Y. occisor*), which is triangular and higher than the dorsal fin and continuous with the smaller, posterior part of the caudal fin. In proportion, the triangular anterior caudal lobe is larger in *Y. ingesdentes* than in *Y. occisor*. The dorsal (dcf) and ventral (vcf) lobes of the posterior caudal fin are almost equally developed. The fin radials in dorsal and caudal fins are clearly visible (Fig. 1c–f, h, Supplementary Fig. 2a, g). The anal fin fold (af) is present in *Y. ingensdentes* (Fig. 1f, h, Supplementary Fig.

1a). The dorsal fin of *Y. ingesdentes* is proportionally slightly higher than that of *Y. occisor*.

1-2 Supplementary Note 2

Character list and coding for phylogenetic analyses

Characters 1-166 adopted from ref.[8] and references there, characters 167-196 adopted from ref.[9] and characters 197-208 are newly defined characters. These also were included in the Supplementary Codes (export from software as nexus format and put in Supplementary Code 1).

1. Skeletal derivatives of neural crest: (0) absent; (1) present.

2. Ectodermal placodes: (0) absent; (1) present.

3. Distinct prechordal head: (0) absent or weakly developed; (1) prominent.

4. Tripartite vesicles at anterior end of neural tube (prosencephalon, mesencephalon, rhombencephalon): (0) absent; (1) present.

5. Morphologically distinct cerebellum with corpus cerebelli: (0) absent; (1) present.

6. Profundal nerve ganglion: (0) separate from trigeminal ganglion; (1) fused with trigeminal ganglion.

7. Tripartite division of facial nerve into pharyngeal, pretrematic, and postrematic branches: (0) absent; (1) present.

8. Spinal cord in cross section: (0) round; (1) flattened.

9. Ventral and dorsal roots of spinal nerve: (0) united; (1) separate.

10. Ventral and dorsal roots of spinal nerve originates: (0) intersegmentally; (1) intrasegmentally.

11. Mauthner fibers at rhombomere 4: (0) absent; (1) present.

12. Pineal organ (extra-ocular photoreceptor region expressing pineal opsins): (0) absent; (1) present.

13. Pineal opening: (0) covered; (1) uncovered.

14. Adenohypophysis: (0) absent; (1) present.

15. Olfactory peduncles: (0) absent; (1) present.

16. Encapsulated olfactory epithelium with external opening: (0) absent; (1) present.

17. Position of nasohypophyseal opening: (0) terminal; (1) dorsal.

Revision: Fossil lampreys have dorsal positioned nasohypophyseal opening, *Mesomyzon* was coded as (1) as in *Mayomyzon* and *Priscomyzon* according to ref.[2];

18. Nasohypophyseal canal: (0) nasohypophyseal canal blind; (1) opening into pharynx.

Revision: *Mesomyzon* was coded as (0) instead of "?" according to ref.[2];

19. Nasohypophyseal opening: (0) single; (1) paired.

20. Olfactory organ: (0) unpaired; (1) paired.

21. Nasohypophyseal canal: (0) maintains width and height anteriorly; (1) tapers anteriorly.

22. External opening of nasohypophyseal canal: (0) terminal aperture; (1) tubular extension.

23. Nasohypophyseal barbels extend from: (0) rim of nasohypophyseal aperture; (1) posteriorly to nasohypophyseal aperture.

24. Nasohypophyseal papillae, ventral element: (0) absent; (1) present.

25. Nasohypophyseal papillae, dorsal element(s): (0) midline; (1) paired.

26. Eye with retinal pigmented epithelium: (0) absent; (1) present.

27. Eyes: (0) exposed; (1) covered by dermis; (2) covered by trunk muscles.

28. Extrinsic eye musculature: (0) absent; (1) present.

29. Muscles innervated by oculomotor nerve: (0) three; (1) four.

30. Oblique muscle innervated by trochlear nerve: (0) posterior oblique; (1) superior oblique.

31. Rectus muscles innervated by abducens nerve: (0) two; (1) one.

32. Eyes: (0) laterally placed (interorbital distance equal to width of head at that; (1) close together near midline (interorbital distance substantially less than width of head at that position; (2) on prominent eyestalk.

33. Cartilaginous otic capsules: (0) absent; (1) present.

34. Vertical semicircular canals forming loops: (0) absent; (1) present.

35. Anterior and posterior semicircular canals: (0) share a canal toward utriculus after meeting in a confluence; (1) meet each other at half a loop.

36. Horizontal semicircular canal: (0) absent; (1) present.

37. Statoliths composed of calcium phosphate: (0) absent; (1) present.

38. Endolymphatic duct: (0) is blind; (1) opens externally.

39. Electroreceptive cells: (0) absent; (1) present.

40. Sensory lines: (0) absent; (1) present.

41. Sensory-lines: (0) on head only; (1) on head plus body.

42. Sensory-line: (0) enclosed in grooves; (1) enclosed in canals.

43. Internal taste buds, or functionally equivalent end chemosensory organs innervated by cranial nerves in head: (0) absent; (1) present; (2) lacking internal taste buds but function replaced by Schreiner organs.

44. Preoptic head length: (0) shorter than branchial length; (1) approximately equal to branchial length; (2) longer than branchial length.

Revision: *Mesomyzon* was coded as equally in length according to ref.[2];

45. Branchial apparatus: (0) retains arrangement of pharyngula such that first branchial opening assumes infra- or postotic position; (1) displaced anteriorly; (2) displaced posteriorly.

46. Branchial apparatus, displaced anteriorly such that: (0) first branchial opening assumes preotic position; (1) multiple branchial arches occupy preotic position.

47. Branchial apparatus, displaced posteriorly such that prebranchial length is: (0) less than a quarter; (1) approximately a quarter; (2) greater than a third of body length.

48. Pharyngeal skeleton: (0) delineates pharyngeal slits with ciliary band; (1) supports well-developed branchial lamellae.

49. Main skeletal support for branchial apparatus with respect to lamellae: (0) lateral; (1) medial.

50. Pharyngeal skeleton: (0) skeletal arches fused with each other; (1) arches isolated.

51.Hyomandibular pouch: (0) blind; (1) externally open (spiracle).

52. Respiratory current exits through: (0) atrial space; (1) through excurrent duct (=branchial pouch); (2) through parabranchial cavity.

53. Single confluent branchial opening: (0) absent; (1) present.

54. Branchial excurrent duct: (0) opens roughly at position of branchial pouch; (1) extends posteriorly.

55. Branchial openings: (0) spaced accordingly with dimensions of branchial cavities; (1) packed closely together; (2) organized into multiple parallel rows.

56. Number of arches (or pouches) in branchial apparatus: (0) unconstrained to five; (1) held constant at five.

57. Number of arches (or pouches) in branchial apparatus, maximum number: (0) greater than five and fewer than 20; (1) greater than 20.

58. Number of arches (pouches) in branchial apparatus: (0) four or five; (1) six or seven; (2) eight to ten; (3) greater than ten.

59. Branchial series extends: (0) substantially less than half the body length; (1) semiequal to or greater than half the body length.

60. Lateral branchial openings: (0) at similar horizontal level; (1) in a posteroventrally inclined row.

61. Opercular flaps associated with gill openings: (0) absent; (1) present.

62. Branchial epithelium in relative to the body: (0) internal; (1) external.

63. External branchial openings, demarcated by: (0) single element entirely; (1) single element dorsally; (2) multiple plates; (3) a framework of multiple spines; (4) micromeres; (5) naked (mineralized exoskeleton locally absent around the opening.

64. Position of mouth: (0) terminal; (1) subterminal.

Revision: *Myxineidus* was coded as subterminal (1) according to refs.[6,10], similar to the hagfishes (e.g., *Tethymyxine*).

65. Epidermal oral cirri: (0) absent; (1) present.

66. Postoptically derived ectomesenchyme anterior to mandibular arch gives rise to palatal structures that: (0) meet at midline under nasal/nasohypophyseal organs; (1)

meet at dorsal midline anterior to nasohypophyseal organs and form a prominent oral roof.

67. Velum: (0) absent; (1) present.

Revision: *Mesomyzon* was coded as present (1) according to ref.[2];

68. Velar cartilages: (0) in hyomandibular position; (1) extend posteriorly.

69. Velar cartilages, functions at terminal ontogenetic stages: (0) pump and valve; (1) valve.

70. Velar wings: (0) absent; (1) present.

71. Velar tentacles, papillae or tubercles: (0) absent; (1) present.

72. Multi-chamber heart: 0, absent; 1, present.

73. Closed pericardium: (0) absent; (1) present.

74. Circulatory system: (0) open; (1) closed.

75. Massive subcutaneous sinus: (0) absent; (1) present.

76. Paired dorsal aortae: (0) absent; (1) present.

Revision: *Mesomyzon* was coded as absent according to ref.[2]; hagfishes all have paired dorsal aorta, whereas lampreys not.

77. Lateral head vein: (0) drains into anterior cardinal vein or its derivative; (1) continues into (or functions as anterior extension of) anterior cardinal vein or its derivative.

78. Lymphocytes: (0) absent; (1) present.

79. Lymphocytes antigen receptors: (0) VLR; (1) T and B.

80. Subaponeurotic vascular plexus: (0) absent; (1) present.

81. Body forms, relative length: (0) less than five times the next largest dimension (height or width); (1) greater than five but less than ten times; (2) greater than ten times.

82. Body forms, width against height: (0) subcircular or compressed so that branchial openings are lateral; (1) depressed so that branchial openings are ventral.

83. Endoskeletal fin supports: (0) absent; (1) present.

84. Distinct dorsal fin: (0) absent; (1) present.

(Note: Character 85 in ref.[8] was replaced with character 186 below)

85. Fin(s) along dorsal midline originates: (0) above branchial series or anterior to mid-trunk; (1) above anus/anal fin or anterior; (2) posteriorly to anus/anal fin.

Revision: *Mesomyzon* coded as (0) according to ref.[2]; *Priscomyzon* recoded as uncertain according to ref.[11] and personal communications to Dr. Miyashita.

86. Distinct anal fin: (0) absent; (1) present.

Notes: *Mesomyzon* probably has an anal fin fold according to ref.[2]; however, it is not determined whether it has radials support or not.

87. Paired skin folds (epidermal ridges) in suprapharyngeal position: (0) absent; (1) present.

88. Constricted pectoral fins with endoskeletal elements: (0) absent; (1) present.

89. Long continuous preanal skin fold (epidermal ridge): (0) absent; (1) present.

90. Preanal skin fold (epidermal ridge): (0) midline; (1) paired.

Miyashista et al.[8] coded cephalochordata, *Pikaia*, *Haikouella*, *Myllokunmingia*, *Haikouichthys*, all hagfish species, *Jamoytius*, *Euphanerops*, *Cornovichthys*, *Jamoytius*, *Euphanerops*, *Lasanius*, *Birkenia*, *Rhyncholepis* as having paired preanal skin fold (1); whereas we coded *Jamoytius*, *Euphanerops*, *Lasanius*, *Birkenia*, *Rhyncholepis* as having paired preanal skin fold)

Revision: *Mesomyzon* has a preanal skin fold according to ref.[2], which is continuous and running along the midline.

91. Preanal skin fold (epidermal ridge): (0) longitudinal; (1) discrete pelvic fins.

Revision: *Mesomyzon* was coded as (0) according to ref.[2].

92. Tail shape: (0) no distinct lobes developed; (1) dorsal lobe much larger than ventral; (2) ventral lobe much larger than dorsal; (3) dorsal and ventral lobes almost equally developed.

Revisions: Miyashita et al.[8] coded character 93 for *Euophanerops*, *Achanarella*, *Cornovichthys*, *Lasanius*, *Birkenia*, *Rhyncholepis*, *Turnia*, *Loganellia* as “ventral lobe much larger than dorsal” (state 1 in their character descriptions), which might be a

typing error. Here we revised state 1 as “dorsal lobe much larger than ventral”, and 2 as “ventral lobe much larger than dorsal”. *Myxineidus, Gilpichthys, Tethymyxine* and extant hagfishes coded as (0); *Myxinikela* code as (1); fossil (including *Pipiscius*) and extant lampreys all coded as (1); Euconodonta coded as (1). As noted by Janvier[6], we redefined the caudal fin in lampreys and the traditionally called ‘posterior dorsal fin’ should be the anterior part of the caudal fin, which is usually located behind the level of the opening of the cloaca[2].

93. Chordal disposition relative to tail development: (0) isochordal; (1) hypochordal; (2) hyperchordal.

94. Skeletal elements consisting of calcium phosphate: (0) absent; (1) present.

95. Bone: (0) absent; (1) present.

96. Cellular bone: (0) absent; (1) present.

97. Lamellar aspidin: (0) absent; (1) present.

98. Perichondral bone: (0) absent; (1) present.

99. Calcified cartilage: (0) absent; (1) present.

100. Cellular cartilages with hypertrophied chondrocytes (30-50 μm in diameter): (0) absent; (1) present.

101. Mature chondrocytes: (0) become separated and generally even spaced by extracellular mat; (1) remain nested in a pair.

102. Dentine: (0) absent; (1) present.

103. Spherical/globular dentine: (0) absent; (1) present.

104. Tubular dentine: (0) absent; (1) present.

105. Tubular dentine, odontobalsts tend to: (0) retreat into pulp cavity; (1) remain in dentinous matrix.

106. Tubular dentine, interconnections of tubules/canaliculi for odontoblasts tend to be: (0) polarized; (1) non-polarized.

107. Tubular dentine, interconnections of canaliculi and spacing between odontoblasts tend to be: (0) regular; (1) irregular.

108. Enamel/oid: (0) absent; (1) present.

109. Enamel: (0) monotypic; (1) bitypic.

110. Calcification/ossification occurs in endoskeleton: (0) absent; (1) present.

111. Calcification/ossification occurs in exoskeleton: (0) absent; (1) present.

112. Mineralized integumentary skeleton, surface coverage: (0) extensive; (1) limited (with evidence for variation and potentials for reduction).

113. Odontodes: (0) monodontodes; (1) polyodontodes.

114. Exoskeleton, organization of superficial layer: (0) spherical; (1) tubular; (2) lamellar.

115. Exoskeleton, vascular/cancellar layer of osteons: (0) absent; (1) present. [an important feature is the three-layered exoskeleton]

116. Exoskeleton, basal tissue: (0) basal lamella; (1) basal attachment.

117. Cancellar layer in exoskeleton, with honeycomb-shaped cavities: (0) absent; (1) present.

118. Scale shape: (0) diamond-shaped; (1) rod-shaped.

119. Oak-leaf-shaped tubercles: (0) absent; (1) present.

120. Triradiate postbranchial spines: (0) absent; (1) present.

121. Median dorsal ridge scales: (0) absent; (1) present.

122. Median dorsal ridge scales: (0) simple; (1) hooked.

123. Vascular canal systems in integumentary skeleton: (0) absent; (1) present.

124. Scales: (0) without visceral ribs; (1) with visceral ribs.

125. Oral plates: (0) absent; (1) present.

126. Odontodes: (0) restricted to exoskeleton; (1) in oral cavity; (2) in pharynx.

127. Dermal head covering in adult state: (0) absent; (1) present.

128. Dermal head covering in adult state: (0) micromeric; (1) large (macromeric) dermal plates or shield.

129. Dermal head covering, macromeric: (0) large unpaired plates covering dorsal and ventral sides; (1) covered by tesserae; (2) multiple plates.

130. Dermal head covering, macromeric/shield: (0) head and anterior trunk continuous; (1) head and anterior trunk decoupled.

131. Endoskeletal contribution to dermal head covering: (0) absent; (1) present.

132. Mineralized exoskeletal circumocular elements: (0) absent; (1) present.

133. Mineralized endoskeletal circumocular elements (sclerotic elements): (0) absent; (1) present.

134. Sclerotic endoskeleton: (0) isolated circumocular elements; (1) eye capsule or stalk.

135. Fusion of visceral arches to the neurocranium: (0) absent; (1) present.

136. Denticulate/cuspidate elements of feeding apparatus, perioral: (0) absent; (1) present.

Revision: *Mesomyzon* was code present according to ref.[2];

137. Perioral feeding elements: (0) continuous epidermal covering; (1) discontinuous arrangement.

Revision: *Mesomyzon* was coded as having discontinuous arrangement according to ref.[2].

138. Denticulate/cuspidate elements of feeding apparatus, housed within or around buccal cavity: (0) absent; (1) present.

139. Longitudinally aligned tooth rows providing transverse bite: (0) absent; (1) present.

Revision: We herein coded lampreys, both fossil and living, as absent (0).

140. Perioral/buccal feeding structure consisting of keratin: (0) absent; (1) present

141. Feeding apparatus forming a pulley-like system of cartilages and protractor-retractor complex in mandibular arch: (0) absent; (1) present.

Revision: *Mesomyzon* was coded present as *Priscomyzon* and *Mayomyzon*;

142. Jaws (dorsoventral bite): (0) absent; (1) present.

143. Keratinous tooth plate, anterior element, number of fused cusps: (0) two; (1) three.

144. Radial organization of circumoral structures: (0) absent; (1) present.

Revision: *Mesomyzon*, *Mayomyzon* were coded as present as in *Priscomyzon*; *Myxineidus* was coded as absent according to refs.[6,10].

145. Circumoral keratinous teeth, number of tooth rows in lateral field: (0) two to four; (1) five or greater; (2) one row lateral row; (3) plate linking lateral teeth.

146. Cartilaginous trematic rings: (0) absent; (1) present.

Revision: *Priscomyzon* and *Mesomyzon* were coded present.

147. Axial skeletal condensations derived from sclerotomes: (0) absent; (1) present.

148. Axial skeletons around dorsal nerve cord (=neural arches): (0) absent; (1) present.

149. Axial skeletons around notochord (=centra): (0) absent; (1) present.

150. Axial skeletons around dorsal aorta (=haemal arches): (0) absent; (1) present.

151. Parachordal cartilages: (0) absent; (1) present.

Revision: *Mesomyzon* herein was coded as present[2], sharing the state of *Priscomyzon*, *Mayomyzon*[6,12].

152. Braincase with lateral walls: (0) absent; (1) present.

153. Occiput enclosing vagus and glossopharyngeal nerves: (0) absent; (1) present.

154. Annular cartilage: (0) absent; (1) present.

Revision: *Mesomyzon* was coded present according to ref.[2]; *Myxineidus* was coded absent according to the teeth arrangement referred in refs.[6,10].

155. Large oral disc: (0) absent; (1) present.

Revision: *Myxineidus* was coded as absent according to ref.[10].

156. Barbels supported by cartilages: (0) absent; (1) present.

Revision: *Myxineidus* was coded as absent.

157. Forked subnasal cartilage: (0) absent; (1) present.

158. Tectal cartilages: (0) absent; (1) present.

159. Male gametes shed directly through the coelom: (0) absent; (1) present.

160. Postotic myomeres migrate anteriorly to the position of eye: (0) absent; (1) present absent.

161. Inflected myomeres: (0) Z-shaped; (1) W-shaped.

Revision: *Mesomyzon* was coded as Z-shaped rather than W-shaped according to ref.[2]; the myomere condition in *Mayomyzon* is uncertain.

162. Digestive tract in relation to branchial region: (0) follows pharynx; (1) passes (or loops) over branchial apparatus.

Revisions: *Mesomyzon* was coded passing over the branchial region according to ref.[2]; *Mayomyzon* was coded passing over branchial region according to refs.[11]; *Hardistiella* was coded so according to ref.[13]; *Priscomyzon* was coded so according to the degree of the development of its branchial apparatus, suggesting the possible divergence of branchial tube and the esophagus as in living lampreys[1,11]; *Pipiscius* coded as in *Mayomyzon* according to refs.[11,14]. The digestive tube is arranged in a different pattern in relation to the branchial apparatus and follows the pharynx in hagfishes.

163. Anus, with respect to distribution of mesoderm: (0) terminal or subterminal; (1) non-terminal.

164. Globular slime glands: (0) absent; (1) present.

165. Number of slime glands: (0) approximately 100 or fewer; (1) substantially greater than 100.

166. Slime pores: (0) overlap region of external branchial openings; (1) do not overlap region of external branchial openings.

Character 168 in ref.[8] (gular pouch in adult male) was represented by character 189 below.

167. Shape of labial teeth: (0) pointed; (1) rounded; (2) spatulate.

168. Alate tooth rows: (0) absent; (1) present.

169. Number of lateral circumoral teeth per side: (0) 3; (1) 4; (2) $\geqslant 5$.

Notes: *Mesomyzon* coded as larger than 5 according to ref.[2]; *Priscomyzon* coded as 4 (state 1) according to ref.[11] and comparison to living lampreys with this number of lateral circumoral teeth.

170. Structure of second lateral circumoral tooth: (0) unicuspid; (1) bicuspid; (2) tricuspid; (3) expanded plates bearing 3-5 cusps; (4) absent.

171. Structure of supraoral laminale: (0) single bridgeless lamina bearing one or more teeth; (1) Two teeth linked by a bridge; (2) two triangular laminae bearing a tooth at

each apex; (3) two central teeth flanked by lateral flanges; (4) supraoral lamina not developed.

172. Structure of infraoral lamina: (0) absent; (1) present and bearing 5 teeth; (2) present and bearing > 5 teeth.

173. Shape of transverse lingual lamina: (0) W-shaped; (1) Straight/almost straight; (2) U-shaped.

174. Arrangement of cusps on transverse lingual lamina: (0) uniform in size; (1) median cusp slightly enlarged; (2) median cusp greatly enlarged; (3) three large cusps only, one median and two lateral ones; (4) median and subterminal cusps slightly enlarge.

175. Shape of longitudinal lingual lamina: (0) parenthesis-shaped; (1) hook-shaped; (2) straight.

176. Length of velar tentacles: (0) short; (1) long.

177. Lateralmost velar tentacles folded over onto the dorsal surface of the velar apparatus to form "wings": (0) absent; (1) present.

178. Surface of velar tentacles: (0) smooth;(1) tuberculated or papillose.

179. Number of velar tentacles: (0) < 5; (1)5-9; (2)10-21; (3)23-32.

180. Median velar tentacle(s): (0) absent; (1) one or two free tentacle(s); (2) two tentacles fused at base along part of their lengths.

181. Development of oral fimbriae: (0) present and distributed over the full perimeter of the oral disc; (1) absent.

182. Structure of oral papillae (anterior and lateral section): (0) uniform size; (1) two of the lateral papillae enlarged.

183. Arrangement of oral papillae: (0) absent posteriorly; (1) absent both anteriorly and posteriorly; (2) absent anteriorly.

184. Position of eyes in mature adults: (0) dorsolateral; (1) dorsal.

185. Types of retinal photoreceptor cell: (0) one cone and one rod; (1) two cones and one rod; (2) one rod.

186. Position of dorsal fins (conventional terminology) relative to each other in mature adults: (0) continuous; (1) contiguous; (2) separate.
187. Connection of 'second dorsal fin'/posterior lobe and caudal fin in immature adults: (0) connected; (1) separate.
188. Number of trunk myomeres in adults: (0) <60; (1) ≥60≤70; (2) >70.
189. Gular pouch in adult males: (0) absent; (1) small; (2) large.
190. Position of cloaca in adults: (0) anterior to or under the origin of the second dorsal fin; (1) under the anterior half of the second dorsal fin or anterior half of the posterior lobe of the single dorsal fin; (2) under the middle or posterior half of the second dorsal fin.
191. Pigmentation of dorsal and upper lateral surfaces of body in adults: (0) uniform; (1) mottled (*Petromyzon*); (2) pair of blue-green dorsolateral stripes.
192. Pigmentation of neuromasts (lateral line organs) in adults: (0) unpigmented; (1) darkly pigmented.
193. Position of otic capsule relative to first branchial opening in adults: (0) posterior to first branchial opening; (1) anterior to or directly over first branchial opening.
194. Predominant type and diploid number of chromosomes: (0) acrocentric and 164-168; (1) metacentric and submetacentric 76; (2) acrocentric and 180.
195. Number of intestinal diverticula in ammocoetes: (0) no intestinal diverticula; (1) 2; (2) 1.
196. Piston cartilage: (0) absent: (1) present.
197. Posterior circumoral teeth: (0) present; (1) absent.
198. Posterior blade on circumorals and disc teeth: (0) absent; (1) present.
199. Posterior exolateral teeth: (0) present and complete; (1) posterior intermediate absent, marginal present; (2) posterior intermediate and marginal absent.
200. Arrangement of circumoral teeth: (0) sparsely arranged; (1) closely apposed
201. Anterior circumoral size relative to lateral circumorals: (0) nearly equally sized; (1) anterior smaller than lateral.

202. Anterior circumoral size in relative to inner most anterior sic teeth: (0) nearly equally in size or even circumoral smaller; (1) anterior circumorals larger than first anterior row teeth; (2) anterior circumoral lacking or far away from oral aperture; (3) anterior disc teeth lacking.

203. Supraoral in relative to lateral circumorals: (0) nearly equally in size; (1) supraoral much larger.

204. Grooves in rear side of supraoral lamina: (0) absent; (1) present.

205. Cusps on infraoral lamina: (0) uniform; (1) differentiated in size.

206. Cusps on lingual longitudinal lamina: (0) small and straight linearly aligned; (1) large and straight linearly aligned; (2) large and gently curving, comb-like; (3) large and V-shaped; (4) two lines of cusps.

207. Circumoral teeth and oral disc teeth or anterior field teeth including anterior circumorals: (0) of similar shape; (1) distinct in shape.

208. Circumoral teeth and oral disc teeth or anterior field teeth including anterior circumoral: (0) of similar size; (1) circumorals larger than anterior disc teeth.

2. Supplementary Discussion

The palaeotemperature curve was projected to our dated petromyzontiform phylogeny (Fig. 4) to reveal the pattern of the tempo-spatial distribution of the recorded fossil lampreys. The graph shows that the emergence of the fossil lampreys appears to match the cool or cold geological intervals or derive from productive areas associated with continental ice sheets, cooling events or the formation of highland topography. *Priscomyzon* occurred in the high-latitude South Africa of the Famennian-Tournaisian Ice Age, *Hardistiella*, *Pipiscius* and *Mayomyzon* in the coldest phase of the Permo-Carboniferous Ice Age[15,16] with an estimated mean surface temperature of the tropical seas of ca. 23℃[15,17], and *Yanliaomyzon* and *Mesomyzon* from Yanliao and Jehol basins that were linked to the Yanshanian mountain-building activities in the Mid-Late Jurassic and Early Cretaceous cool intervals[15], with the fossil localities of

Yanliaomyzon possibly situated on a highland[18,19], which is reminiscent of the distribution of Mexican lampreys (*Tetrapleurodon*) in the tropical zone at 20º N but restricted in high altitude cool watersheds[20].

The morphological data were divided into three partitions with independent relaxed clock models (Supplementary Code 2), making it available to investigate the evolutionary rate heterogeneity among them.

The characters of feeding apparatus (mechanism) evolved extremely rapidly with relative rate 10.7 (0.02, 45.1) (median and 95% HPD, same for below) in the transition from the common ancestor shared by petromyzontiform (lampreys) and myxinoidea (hagfishes) (Fig. 3 and Supplementary Fig. 3) and then dramatically slowed down to 1.5 (0.01, 7.6) leading to *Pipiscius* and 0.4 (0.0, 2.9) leading to the stem and crown petromyzontiforms. This suggests that morphological changes in the feeding mechanism were rapid at the dawn of the history of the group and this functional unit might have played a vital role in this stage just as previously supposed[1,6]. The rates continued to slow down substantially and differentiated radically during their later history (Fig. 3, Supplementary Fig. 3). Most of the rates are well below 0.5, however, it is worth noting that the rate at the branch from the Palaeozoic to Mesozoic lampreys is roughly six times that along the branch of crown-group lampreys, and that a high rate of 5.4 (0.01, 23.6) is observed along the branch toward Mordaciidae + Petromyzontidae, which is ~77 times of that along the branch toward *Geotria* (Supplementary Fig. 3). Additionally, within the Mesozoic forms, a modest evolutionary acceleration is also detected at the branch towards *Yanliaomyzon* species when lampreys diverge from *Mesomyzon* lineage in the Early Jurassic (Supplementary Fig. 3). These observations concur with the significant increase of the adult body size of the lampreys since the Palaeozoic era in tandem with the establishment of the ‘modern’ three-phased life history.

The timetree of all taxa estimated from total-evidence dating combining both morphological and molecular data is shown in Fig. 3. Making use of all available data

including the fossil ages refines the phylogeny comparing to previous analyses[8]. We highlight several taxa positions in the timetree which appear more reasonable. Using the morphological characters alone, fossil *Tethymyxine* was placed in the crown group of Myxinoidea (sister to *Rubicundus*, ref.[8]), now it is at the stem when taking its age into account in the total-evidence analysis (Fig. 3), because the age of *Tethymyxine* is older than the estimated age of crown Myxinoidea. Southern Hemisphere lampreys were paraphyletic when using both morphological and molecular data, while *Geotria australis* was placed as a sister group of *Mordacia* species when only morphological characters were used. This difference is mostly due to incorporating both times and rates in the dating analyses, indicated by long time span (~78 Myr) but slow evolutionary rates leading to *Geotria australis*.

The divergence times of the major clades are generally younger than those estimated in the recent study[8] but more compatible with the estimates of the former molecular study for the myxinoid crown[21]. In comparison, a few credibility intervals appeared either too wide or too narrow in the previous study (figure S9 in ref.[8]). For example, the interval only spanned 7 million years (543.3–536.0 Ma) for Cyclostomi (total group), but spanned 185 million years (350.5–165.0 Ma) for the node of *Mesomyzon* + crown-group petromyzontiform. This is largely due to the two-step approach applied in their study and each step only incorporated part of the data. They first inferred the phylogeny using purely morphological characters. Then the tree topology was fixed while the fossil ages and molecular data were used to date the tree (discarding the morphological characters). Thus, the divergence time estimates were mainly informed by the fossilized birth-death prior without the morphological characters. Our approach takes full use of the data, including fossil ages, morphological characters and molecular sequences in a coherent analysis. Besides, we also included more fossils in the stem and revised the morphological data (see above).

3. Supplementary References

1. Hardisty, M. W. *Biology of Cyclostomes* (Chapman and Hall, London, 1979).

2.Wu, F. X., Chang, M.-M. & Janvier, P. A new look at the Cretaceous lamprey *Mesomyzon mengae* Chang, Miao & Zhang, 2006 from the Jehol Biota. *Geodiversitas* **3(23)**, 1293–1307 (2021).

3.Hilliard, R. W., Potter, I. C. & Macey, D. J. The dentition and feeding mechanism in adults of the Southern Hemisphere lamprey *Geotria australis* Gray. *Acta Zool.* (Stockh.) **66**, 159–170 (1985).

4.Baker, C. F. *et al.* Morphometric and physical characteristics distinguishing adult Patagonian lamprey, *Geotria macrostoma* from the pouched lamprey, *Geotria australis*. *PLoS ONE* **16(5)**, e0250601 (2021).

5.Potter, I. C. & Hilliard, R. W. A proposal for the functional and phylogenetic significance of differences in the dentition of lampreys (Agnatha: Petromyzontiformes). *J. Zool. Lond.* **212**, 713–737 (1987).

6.Janvier, P. Early jawless vertebrates and cyclostome origins. *Zool. Sci.* **25**, 1045–1056 (2008).

7.Goodrich, E. S. *Studies on the Structure and Development of Vertebrates* (Dover, 1930).

8. Miyashita, T. *et al.* Hagfish from the Cretaceous Tethys Sea and a reconciliation of the morphological-molecular conflict in early vertebrate phylogeny. *Proc. Natl. Acad. Sci. USA* **116**, 2146–2151 (2019).

9. Gill, H. S. *et al.* Phylogeny of living parasitic lampreys (Petromyzontiformes) based on morphological data. *Copeia* **2003**, 687–703 (2003).

10. Poplin, C., Sotty, D. & Janvier, P. Un Myxinoïde (Craniata, Hyperotreti) dans le Konservat-Lagerstätte Carbonifère supérieur de Montceau-les-Mines (Allier, France). *CR Acad. Sci. Paris* **332**, 345–350 (2001).

11. Miyashita, T., Gess, R. W., Tietjen, K., Coates, M. I. Non-ammocoete larvae of Palaeozoic stem lampreys. *Nature* **591**, 408–412 (2021).

12. Bardack, D. & Zangerl, R. First fossil lamprey: A record from the Pennsylvanian of Illinois. *Science* **162**, 1265–1267 (1968).

13. Janvier, P. & Lund, R. *Hardistiella montanensis* n. gen. et sp. (Petromyzontida) from the Lower Carboniferous of Montana, with remarks on the affinities of the lampreys. *J. Vert. Paleontol.* **2**, 407–413 (1983).

14. Bardack, D. & Richardson, E. S. New agnathous fishes from the Pennsylvanian of Illinois. *Fieldiana Geol.* **33**, 489–510 (1977).

15. Scotese, C. R., Song, H. J., Mills, B. J. M. & van der Meer, D. G. Phanerozoic paleotemperatures: The earth's changing climate during the last 540 million years. *Earth-Sci. Rev.* **215**, 103503 (2021).

16. Peyser, C. E. & Poulsen, C. J. Controls on Permo-Carboniferous precipitation over tropical Pangaea: A GCM sensitivity study. *Palaeogeogr. Palaeoclimatol. Palaeoecol.* **268**, 181–192 (2008).

17. Pardo, J. D., Small, B. J., Milner, A. R. & Huttenlocker, A. K. Carboniferous-Permian climate change constrained early land vertebrate radiations. *Nat. Ecol. Evol.* **3**, 200–206 (2019).

18. Zhou, Z. H. & Wang, Y. Vertebrate assemblages of the Jurassic Yanliao Biota and the Early Cretaceous Jehol Biota: Comparisons and implications. *Paleoworld* **26**, 241–252 (2017).

19. Li X. B, Zhang Y. & Tong Y. B. Preliminary analysis on the paleogeography and paleoenvironment in the eastern Yanliao area during the Jurassic-Cretaceous tectonic transition. *Earth Sci. Front.* **28**, 391–411 (2021). (In Chinese with English abstract)

20. Renaud, C. B. *Lampreys of the World. An annotated and illustrated catalogue of lamprey species known to date* (Food and Agriculture Organization of the United Nations, 2011).

21. Kuraku, S. & Kuratani, S. Time scale for cyclostome evolution inferred with a phylogenetic diagnosis of hagfish and lamprey cDNA sequences. *Zool. Sci.* **23**, 1053–1064 (2006).

4. Supplementary Figures and Legends

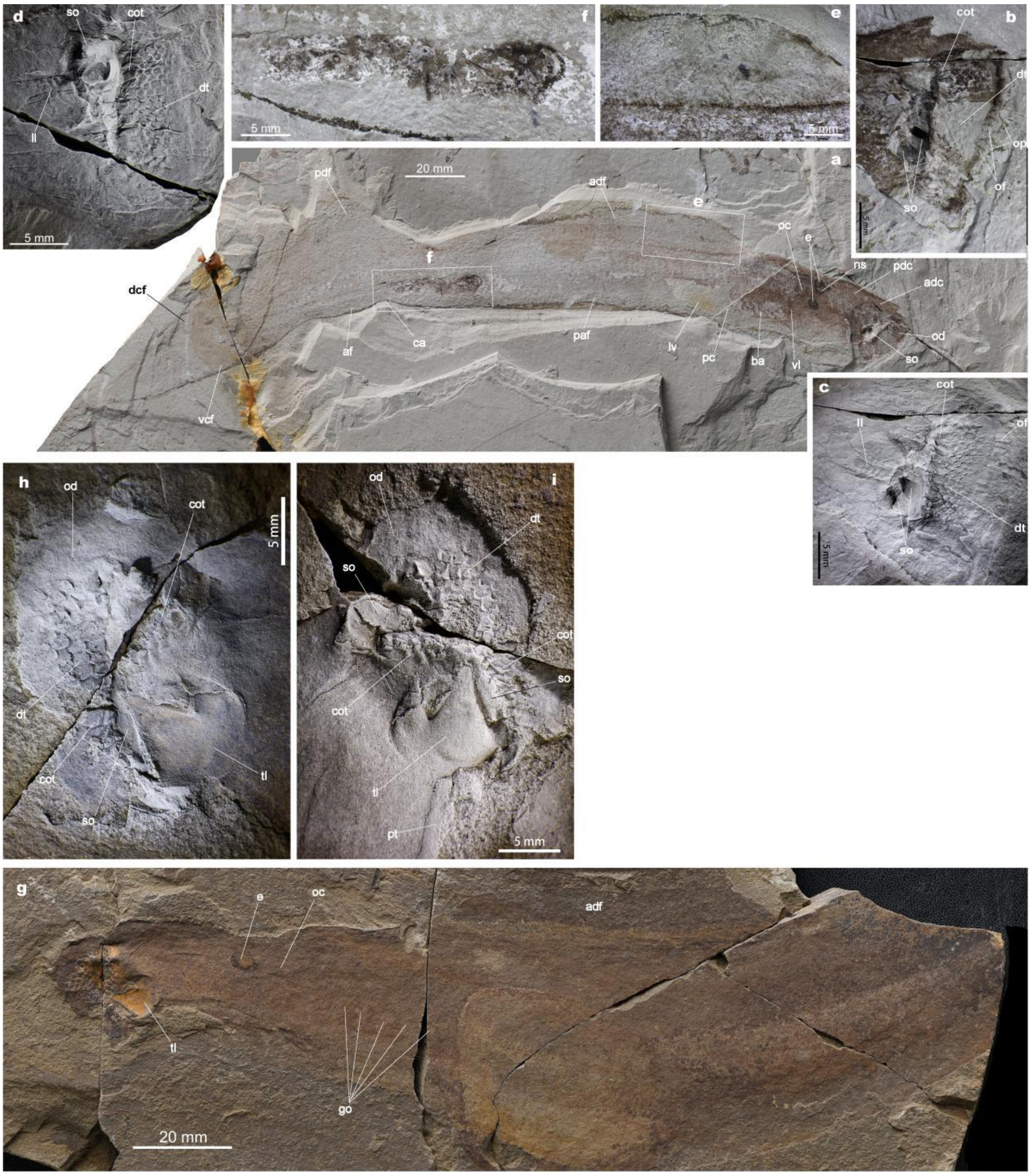

Supplementary Figure 1. Jurassic lampreys, *Yanliaomyzon occisor* gen. et sp. nov. and *Yanliaomyzon ingensdentes* gen. et sp. nov. a to **f**, *Yanliaomyzon ingensdentes* gen. et sp. nov. (**a**) Counterpart of holotype (IVPP V 16715A); (**b**) Photograph and (**c**) that whitened by ammonia chloride of oral disc and dentition of (**a**); (**d**) oral disc and dentition of part of holotype (IVPP V16715B), photograph covered with ammonia chloride and horizontally flipped for comparison; (**e**) Close-up of front section of anterior dorsal fin; (**f**) Close-up of intestine contents, showing the skeletal relics of the

prey; **g–i**, *Yanliaomyzon occisor* gen. et sp. nov. (**g**) Counterpart of paratype (IVPP V 18956A); (**h**) oral disc and dentition of (**g**), whitened by ammonia chloride; (**i**) oral disc and dentition of part specimen of paratype (IVPP V 18956B), whitened by ammonia chloride. Abbreviations: **adc**, anterior dorsal cartilage; **adf**, ‘anterior dorsal fin’ (dorsal fin); **af**, anal fin fold; **ba**, branchial apparatus; **ca**, cloaca (anus); **cot**, circumoral teeth; **dt**, oral disc teeth; **e**, eyes; **go**, external gill openings; **ll**, longitudinal lingual lamina; **lv**, liver; **ns**, olfactory organ ; **oc**, otic capsule; **od**, oral disc; **of**, oral fimbriae; **op**, oral papilla(e); **paf**, precloacal skin fold; **pc**, pericardial cartilage; **pdc**, posterior dorsal cartilage; **pt**, piston cartilage; **so**, supraoral lamina; **tl**, transverse lingual lamina; **vl**, velum.

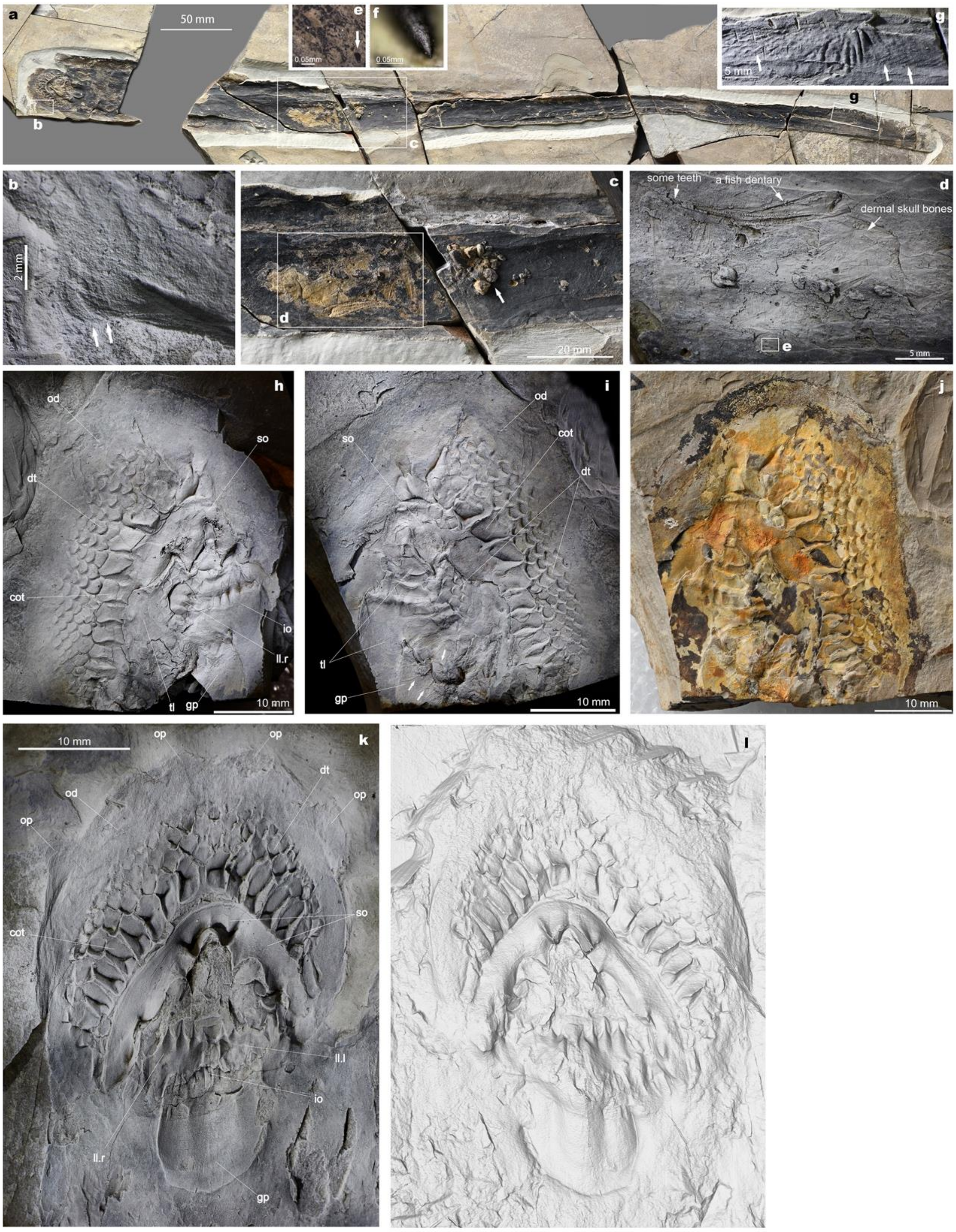

Supplementary Figure 2. Feeding apparatus of *Yanliaomyzon* and preys of *Yanliaomyzon occisor* gen. et sp. nov. a–g, *Yanliaomyzon occisor* gen. et sp. nov. (**a)** Holotype (IVPP V 15830); (**b**) Close-up of an oral papilla, whitened by ammonia chloride; (**c**) Close-up some intestine contents; (**d**) Some disarticulated bones of an unknown ray-finned fish, image upside-down to facilitate observation; (**e**) an isolated phosphatic tooth within the intestine contents (arrowed); (**f**) Close-up of (**e**); (**g**) Close-

up of posterior dorsal fin, arrows pointing to the fin radials; **h**–**j**, Oral disc and dentition of *Yanliaomyzon ingensdentes* gen. et sp. nov. (**h**) Part (IVPP V16716B) and (**i**) counterpart (IVPP V16716B) of the paratype, images whitened by ammonia chloride; (**j**) Counterpart (IVPP V16716A) of paratype; **k** and **l**, Oral disc and dentition of holotype of *Yanliaomyzon occisor* gen. et sp. nov. (**k**) Photograph, (**l**) Computed tomography (produced using VGstudio 2.2), showing the morphology of the dentition which was actually preserved as a depressed mold. Abbreviations: **cot**, circumoral teeth; **dt**, oral disc teeth; **gp**, gular pouch; **io**, infraoral lamina; **ll.l**, left longitudinal lingual lamina; **ll.r**, right longitudinal lingual lamina; **od**, oral disc; **of**, oral fimbriae; **op**, oral papilla(e); **so**, supraoral lamina; **tl**, transverse lingual lamina.

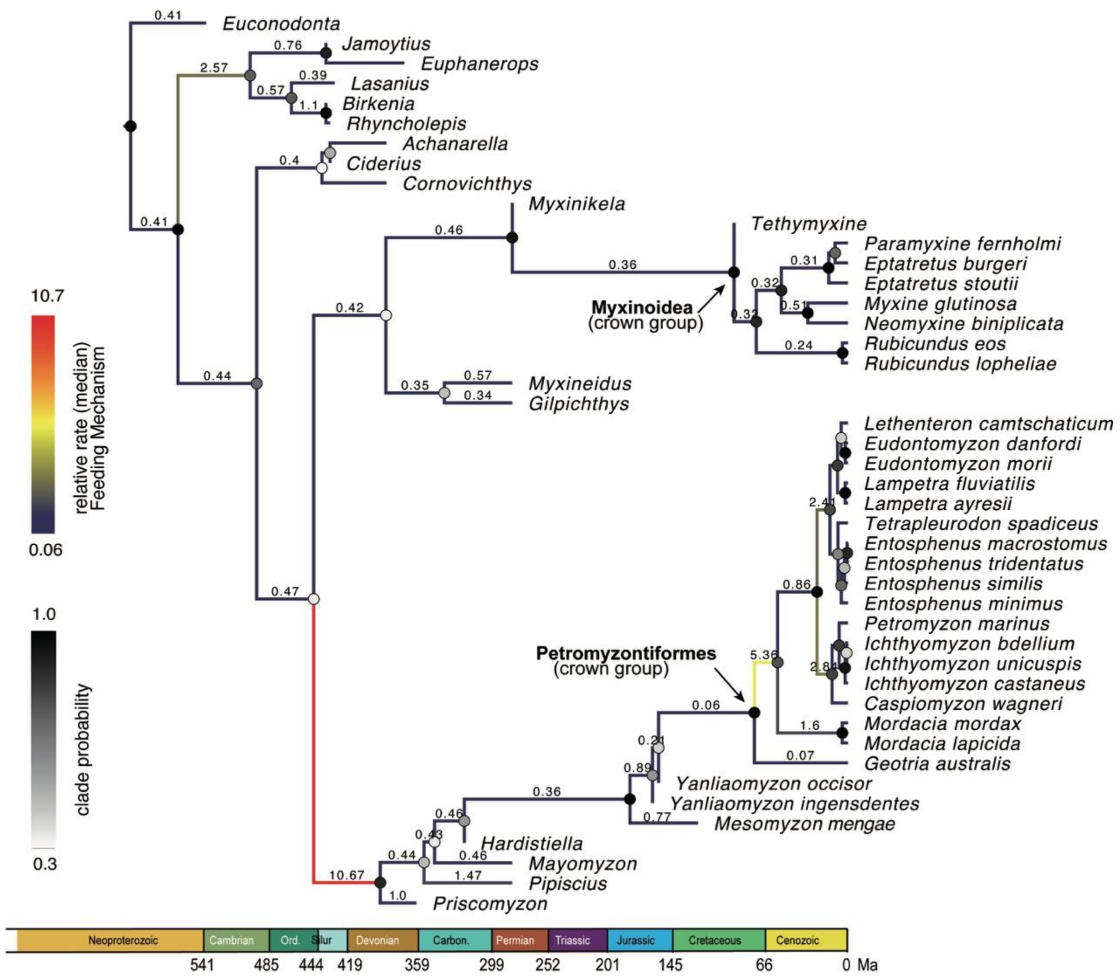

Supplementary Figure 3. Dated phylogeny (timetree) of cyclostomes and the evolutionary rates of their feeding mechanism. The colour of the branch represents the

median relative rate of the feeding mechanism characters at that branch. The median values of the relative rates in some focal branches are also shown. For abbreviations see the legend of Fig. 3. For more parameter estimates, such as the branch rates of different partitions, see the log file and tree file (Supplementary Codes 2, 3).

6. Supplementary Table 1. Measurements of *Yanliaomyzon occisor* gen. et sp. nov and *Yanliaomyzon ingensdentes* gen. et sp. nov, and comparison with other fossil lampreys

Abbreviations and interpretations: **a-C**, tail length; **d-O**, preorbital length (from the anteriormost edge of the oral disc to anterior edge of the eye); **d-B1**, prebranchial length or head length; **d-n**, snout length (where narial opening not well-preserved, measured between the anterior edge of the naso-hypophysial organ to the anteriormost edge of oral disc); **B_1-B_2**, interbranchial opening length; **B_1-B_7**, branchial length; **B_7-a**, trunk length; **O**, eye length; **oa**, length between the anteriormost edge of the oral disc tip to the cloaca; **O-B_1**, postocular length; **od**, length of maximal axis of the oral disc; **TL**, total length. All measurements are in mm. For *Yanliaomyzon occisor* some values were taken from the paratype (IVPP V18956), otherwise were from the holotype (IVPP V 15830).

Taxon	TL	oa	B7-a	a-C	d-n	O	O-B_1	B1-B2	od	d-O	d-B1	B1-B7	od/TL（%）
Yanliaomyzon occisor	642	463	?	179	?	4.3 (V 18956)	?	?	33.5	38.3 (V 18956)	51.7 (V 18956)	30.9 (V 18956)	5.2
Yanliaomyzon ingensdentes	296	177	116	119	29	4	6.5	?	?	33	42	12	?
Mesomyzon mengae	150-217	113-166	74-107	37-51	13-20	2-2.5	6.5-8	2-3	7.5-13	15.5-17.5	20-32	13-21	4.4
Mayomyzon	33-61	?	?	?	?	?	?	?	?	?	7.8	?	3.5
Hardistiella	<117	ca. 76	?	36.9	?	?	?	?	5	?	13.5	?	6.1
Pipiscius	40-65	?	?	?	?	?	?	?	?	?	?	?	?
Priscomyzon	4.2	?	26.5	?	?	?	?	?	8.3	?	9.1	?	21.7
