# Supplementary material for: The rise of predation in Jurassic lampreys
Source: Nat Commun. 2023 Oct 31;14:6652. doi: 10.1038/s41467-023-42251-0 (PMC10618186; doi:10.1038/s41467-023-42251-0)
Supplement: Supplementary file 3 — Description of Additional Supplementary Files [file 41467_2023_42251_MOESM3_ESM.pdf]

Description of Additional Supplementary Files

File Name: Supplementary Code 1
Description: Total evidence data matrix

File Name: Supplementary Code 2
Description: Total-evidence analyses command and log file

File Name: Supplementary Code 3
Description: All-compatible tree file

File Name: Supplementary Code 4
Description: Ancestral state reconstruction

File Name: Supplementary Code 5
Description: Reconstruction of ancestral states of feeding mechanism (1)

File Name: Supplementary Code 6
Description: Reconstruction of ancestral states of feeding mechanism-2

File Name: Supplementary Code 7
Description: Reconstruction of ancestral areas of some key nodes (distribution partitions)

File Name: Supplementary Code 8
Description: Reconstruction of ancestral areas of some key nodes (run)
