# Supplementary material for: The rise of predation in Jurassic lampreys
Source: Nat Commun. 2023 Oct 31;14:6652. doi: 10.1038/s41467-023-42251-0 (PMC10618186; doi:10.1038/s41467-023-42251-0)
Supplement: Supplementary file 4 — Supplementary Code 1-8 [file 41467_2023_42251_MOESM4_ESM.zip › Supplementary Codes 1-8/Supplementary Code 1.rtf]

Supplementary Code 1: Total evidence data matrix#NEXUSBegin data;       Dimensions ntax = 45 nchar = 1701;       Format datatype = mixed (standard:1-208, DNA:209-1701) interleave = yes gap = - missing = ?;       Matrix       Euconodonta                ?10?????????????????-----10????0???????????0---???????1??00???-??0???--?????????201010?00--1110---0??1111111110-1------------2------0-?0-110?--0-????????00????01?1???------------------------------------------     Jamoytius                  111????????????11?0?-----10????0???????????010-?00?10000030?0051?????--?????????10?0??00110??1??00?????????0-011??0?--000-?-0-0----0--?0-0-0?0-0-????????100????1?????------------------------------------------       Euphanerops                111????????????110??-----10????????????????010-100?100001-1100-101???--?????????101021001101110--01110-------10-------------0-0-----0-?0-0-0?0-0-?111111?10001??1110--------------------------------------------       Achanarella                111????????????11?00-----10????0???????????00--?00??0??0131?00-??????--?????????10112100?--110-----??---------------------------------?0-?????-?-?????????00??????1???------------------------------------------       Ciderius                   111????????????11?00-----10????0???????????00--?00?10??0130?00-??????--?????????101???00?--??0-----??---------------------------------?0-????0-?-?????????00????1?1???------------------------------------------       Cornovichthys              ?11?????????????????-----10?????1??????????00--?00?10000031100-?0????--?????????10102000101110-----??---------------------------------?0-????0-?-?????????00??????1???------------------------------------------     Lasanius                   ?11????????????11?00-----10????01??????????02-0????101100101005001???--?????????1010210011011110100??????--0-011????-10111?-000----0--?0-0-??0-0-????????100????1?1???------------------------------------------       Birkenia                   111?????????1??11?0?-----10????0???????110?02-0????101100201003001???--?????????0010210011011110100??110---1-0101000-101110110102-010-?100-??0-0-?????????00??????1???------------------------------------------       Rhyncholepis               111?????????1??11?0?-----10????0???????110?02-0????101100201000001???--?????????0010210011011110100??110---1-0101000-101111110102-010-?100-??0-0-?????????00??????1???------------------------------------------       Myxinikela                 111????????????10?00000??10????11??????????12-0?00?10??0000??0-??0???--?????????10?010?00--1?0-----??---------------------------------10-????0?0-?????100001????1??0--------------------------------------------       Tethymyxine                1?1????????????1010?101????????????????????02-1????10??0020??0-1?0110--?????????20?010001000?0-----??---------------------------------10-11110?0-?????1??00110???01110------------------------------------------       Paramyxine_fernholmi       111101011000-101010000001110---11010100100202-2100010120010000-100110--10111010020101000100010-----??---------------------------------10-1111010-110011000011011101101------------------------------------------       Eptatretus_burgeri         111101011000-10101000000?110---11010100100202-1100010110010000-100110--10111010020101000100010-----1?---------------------------------10-1111010-110011000011011101100------------------------------------------       Eptatretus_stoutii         111101011000-101010000011110---11010100100202-2100010110030000-100110--10111010020101000100010-----11---------------------------------10-1111010-110011000011011101100------------------------------------------       Myxine_glutinosa           111101011000-101010000000120---11010100100202-2100011--0010-00-100110--10111010020101000100010-----11---------------------------------10-1111000-110011000011011101101------------------------------------------       Rubicundus_eos             11110?011000-1010100111??110---11010?00100?02-1100010000000000-100110--101110?0020101000100010-----??---------------------------------10-1111010-110011000011011101110------------------------------------------       Rubicundus_lopheliae       11110?011000-101010011111110---11010?00100?02-1100010000000000-100110--101110?0020101000100010-----??---------------------------------10-1111010-110011000011011101100------------------------------------------       Neomyxine_biniplicata      11110?011000-1010100000?0120---11010?00100?02-1100011--0010000-100110--101110?0020101000100010-----??---------------------------------10-1111000-110011000011011101110------------------------------------------       Myxineidus                 ??1?????????????????-----??????????????????10--????1????0?0??0-??110????????????20?????0?????0-----??---------------------------------?0-11??0-0?0???????000??????????------------------------------------------       Gilpichthys                ?11?????????????????-----10????01??????????20--????10?1001000?-0?0???--?????????10?0?0?00--010-----??---------------------------------?0-11????0-????????000????1?10--------------------------------------------       Lethenteron_camtschaticum  11110101011111011000-----10100001000101110100--100010000010100-00110111111000100201110000--110-----11---------------------------------11110110-101110011011001111110--020112220111110000010201001??1001012100000       Petromyzon_marinus         11110101011111011000-----10100001000101110100--100010000010100-00110100111000100201110000--110-----11---------------------------------11110110-111110011011001111110--011102001000000000010211111001000100000300       Lampetra_fluviatilis       11110101011111011000-----10100001000101110100--100010000010100-00110101111000100201110000--110-----11---------------------------------11110110-101110011011001111110--020212222101110000010101011001101012100000       Geotria_australis          11110?01011111011000-----1010000100010?110100--100010000010100-00110110111000100201110000--110-----??---------------------------------11110110-111110011011001111110--212032230110320100121220211211012101110111       Ichthyomyzon_bdellium      11110101011111011000-----10100001000101110100--100010000010100-00110100111000100201110000--110-----11---------------------------------11110110-111110011011001111110--011102000000000000?0001101???1000110000200       Ichthyomyzon_castaneus     11110101011111011000-----10100001000101110100--100010000010100-00110100111000100201110000--110-----11---------------------------------11110110-111110011011001111110--011102100000000000?00011011??1000110000200       Ichthyomyzon_unicuspis     11110101011111011000-----10100001000101110100--100010000010100-00110100111000100201110000--110-----11---------------------------------11110110-111110011011001111110--011002000000000000000011011??1000110000200       Mordacia_mordax            11110?01011111011000-----10100001000?0?110100--100010000010100-00110100111000?0?201110000--110-----??---------------------------------11110110-131110011011001111110--012322041000011011221202011121000011101400       Mordacia_lapicida          11110?01011111011000-----10100001000?0?110100--100010000010100-00110100111000?0?201110000--110-----??---------------------------------11110110-131110011011001111110--012322041000011021?20222?????1000011101400       Caspiomyzon_wagneri        11110101011111011000-----10100001000101110100--100010000010100-00110101111000100201110000--110-----11---------------------------------11110110-111110011011001111110--111001100101010000?10111001??1000101000?00       Tetrapleurodon_spadiceus   11110101011111011000-----10100001000101110100--100010000010100-00110111111000100201110000--110-----11---------------------------------11110110-101110011011001111110--01111211011121000??101?100???1001012100?00       Entosphenus_macrostomus    11110101011111011000-----10100001000101110100--100010000010100-00110111111000100201110000--110-----11---------------------------------11110110-101110011011001111110--02121111011121000???01?101???1001012100200       Entosphenus_minimus        11110101011111011000-----10100001000101110100--100010000010100-00110110111000100201110000--110-----11---------------------------------11110110-101110011011001111110--021211110010110000?1011100???1001012100200       Entosphenus_similis        11110101011111011000-----10100001000101110100--100010000010100-00110111111000100201110000--110-----11---------------------------------11110110-101110011011001111110--02121111011111000???010101???1001012100200       Entosphenus_tridentatus    11110101011111011000-----10100001000101110100--100010000010100-00110111111000100201110000--110-----11---------------------------------11110110-101110011011001111110--021211110111210000010111011?01001012100200       Eudontomyzon_danfordi      11110101011111011000-----10100001000101110100--100010000010100-00110111111000100201110000--110-----11---------------------------------11110110-111110011011001111110--010112222111210000?1010100???1000010100?00       Eudontomyzon_morii         11110101011111011000-----10100001000101110100--100010000010100-00110111111000100201110000--110-----11---------------------------------11110110-111110011011001111110--01011222211111000???02?100???1000010100?00       Lampetra_ayresii           11110101011111011000-----10100001000101110100--100010000010100-00110101111000100201110000--110-----11---------------------------------11110110-101110011011001111110--020212222101010000?1010100???1101012100000       Mesomyzon              111????????????11000-----10????01??????????10--100?10000010100-00111????1??0????20110100100110-----??---------------------------------11110110?121????11?11001??0110--00200?????????01?0?20112??1??11?21031???--       Yanliaomyzon_ ingensdentes  111?????????????????-----10????01??????????20--100?10000010000-0011?????1??0????20110?000--110-----??---------------------------------11110110-111????1??11001??0110--212032230????? 00?0?10?11 ??1??11121 01110111       Yanliaomyzon_ occisor   111?????????????????-----10????01??????????20--100?10000010000-0011?????1??0????20110?00???110-----??---------------------------------11110110-111????1??11001??0110--212032230??? ?? 00?0?10?1???1??1112101110111       Priscomyzon                111?????????????1???-----10????01??????????20--100?10000010??0-001??????1???????01?0???00--??0-----??---------------------------------11110110-?21????1??110?1???110-?-01?40?????????????00?????0??10??003????--       Mayomyzon                  111????????????110??-----10????01??????????20--100?10?10010100-001??????????????100010?00--110-----??---------------------------------11111?10-1?1????1??11001???110-???????????????????????????0??10???????????       Hardistiella               ?11?????????????1???-----10????0????1??????20--1???10?10010??0-001??????????????101111?00--110-----??---------------------------------???????0-???????????11?1???11??????????????????????1???0??????????????????       Pipiscius                  11??????????????????-----10????01??????????21--????10?1??0010?-0?????--?????????101020?00--1?0-----??---------------------------------?110-?00-?-?????????10????1110--0021?0?????????????????0??0??10??10?--??--              [16S, 772 sites]     Euconodonta                ????????????????????????????????????????????????????????????????????????????????????????????????????????????????????????????????????????????????????????????????????????????????????????????????????????????????????????????????????????????????????????????????????????????????????????????????????????????????????????????????????????????????????????????????????????????????????????????????????????????????????????????????????????????????????????????????????????????????????????????????????????????????????????????????????????????????????????????????????????????????????????????????????????????????????????????????????????????????????????????????????????????????????????????????????????????????????????????????????????????????????????????????????????????????????????????????????     Jamoytius                  ????????????????????????????????????????????????????????????????????????????????????????????????????????????????????????????????????????????????????????????????????????????????????????????????????????????????????????????????????????????????????????????????????????????????????????????????????????????????????????????????????????????????????????????????????????????????????????????????????????????????????????????????????????????????????????????????????????????????????????????????????????????????????????????????????????????????????????????????????????????????????????????????????????????????????????????????????????????????????????????????????????????????????????????????????????????????????????????????????????????????????????????????????????????????????????????????????     Euphanerops                ????????????????????????????????????????????????????????????????????????????????????????????????????????????????????????????????????????????????????????????????????????????????????????????????????????????????????????????????????????????????????????????????????????????????????????????????????????????????????????????????????????????????????????????????????????????????????????????????????????????????????????????????????????????????????????????????????????????????????????????????????????????????????????????????????????????????????????????????????????????????????????????????????????????????????????????????????????????????????????????????????????????????????????????????????????????????????????????????????????????????????????????????????????????????????????????????????     Achanarella                ????????????????????????????????????????????????????????????????????????????????????????????????????????????????????????????????????????????????????????????????????????????????????????????????????????????????????????????????????????????????????????????????????????????????????????????????????????????????????????????????????????????????????????????????????????????????????????????????????????????????????????????????????????????????????????????????????????????????????????????????????????????????????????????????????????????????????????????????????????????????????????????????????????????????????????????????????????????????????????????????????????????????????????????????????????????????????????????????????????????????????????????????????????????????????????????????????     Ciderius                   ????????????????????????????????????????????????????????????????????????????????????????????????????????????????????????????????????????????????????????????????????????????????????????????????????????????????????????????????????????????????????????????????????????????????????????????????????????????????????????????????????????????????????????????????????????????????????????????????????????????????????????????????????????????????????????????????????????????????????????????????????????????????????????????????????????????????????????????????????????????????????????????????????????????????????????????????????????????????????????????????????????????????????????????????????????????????????????????????????????????????????????????????????????????????????????????????????     Cornovichthys              ????????????????????????????????????????????????????????????????????????????????????????????????????????????????????????????????????????????????????????????????????????????????????????????????????????????????????????????????????????????????????????????????????????????????????????????????????????????????????????????????????????????????????????????????????????????????????????????????????????????????????????????????????????????????????????????????????????????????????????????????????????????????????????????????????????????????????????????????????????????????????????????????????????????????????????????????????????????????????????????????????????????????????????????????????????????????????????????????????????????????????????????????????????????????????????????????????     Lasanius                   ????????????????????????????????????????????????????????????????????????????????????????????????????????????????????????????????????????????????????????????????????????????????????????????????????????????????????????????????????????????????????????????????????????????????????????????????????????????????????????????????????????????????????????????????????????????????????????????????????????????????????????????????????????????????????????????????????????????????????????????????????????????????????????????????????????????????????????????????????????????????????????????????????????????????????????????????????????????????????????????????????????????????????????????????????????????????????????????????????????????????????????????????????????????????????????????????????     Birkenia                   ????????????????????????????????????????????????????????????????????????????????????????????????????????????????????????????????????????????????????????????????????????????????????????????????????????????????????????????????????????????????????????????????????????????????????????????????????????????????????????????????????????????????????????????????????????????????????????????????????????????????????????????????????????????????????????????????????????????????????????????????????????????????????????????????????????????????????????????????????????????????????????????????????????????????????????????????????????????????????????????????????????????????????????????????????????????????????????????????????????????????????????????????????????????????????????????????????     Rhyncholepis               ????????????????????????????????????????????????????????????????????????????????????????????????????????????????????????????????????????????????????????????????????????????????????????????????????????????????????????????????????????????????????????????????????????????????????????????????????????????????????????????????????????????????????????????????????????????????????????????????????????????????????????????????????????????????????????????????????????????????????????????????????????????????????????????????????????????????????????????????????????????????????????????????????????????????????????????????????????????????????????????????????????????????????????????????????????????????????????????????????????????????????????????????????????????????????????????????????     Myxinikela                 ????????????????????????????????????????????????????????????????????????????????????????????????????????????????????????????????????????????????????????????????????????????????????????????????????????????????????????????????????????????????????????????????????????????????????????????????????????????????????????????????????????????????????????????????????????????????????????????????????????????????????????????????????????????????????????????????????????????????????????????????????????????????????????????????????????????????????????????????????????????????????????????????????????????????????????????????????????????????????????????????????????????????????????????????????????????????????????????????????????????????????????????????????????????????????????????????????     Tethymyxine                ????????????????????????????????????????????????????????????????????????????????????????????????????????????????????????????????????????????????????????????????????????????????????????????????????????????????????????????????????????????????????????????????????????????????????????????????????????????????????????????????????????????????????????????????????????????????????????????????????????????????????????????????????????????????????????????????????????????????????????????????????????????????????????????????????????????????????????????????????????????????????????????????????????????????????????????????????????????????????????????????????????????????????????????????????????????????????????????????????????????????????????????????????????????????????????????????????       Paramyxine_fernholmi       -----------------------------ATATAATAAGTCCTACCTGCCCAG--TGAT-TT--TTTCAACGGCCAT-------CTCTGATGATAACGTAGCATAATCAATTGTCTATTAATTATAGACTAGAATGAATGGATTAACGAAAGCCAGA-----------------------CTGTCTCCTAATTTTAATCAATGAAATTATCCTTTCTGTCCAAAGACAGAAATTCTTTTATAAGACGAAAAGACCCTA-AAAGCTTCAAACATTAA----A-TTAACCTATCTAT----------------------AA--AAACAA-TAAAGAAAA--ATACTAATTT-TAAA---AA--------------------------------------------TTTGGTTGGGGCAACCAC-------------------TGAATAAAACTATCATCAATAATTAAACGA--AA--A-----ATCT-TCTTA------------AAACAAAGTAACAACTTAGTTAAA-----------TAAGAAACTTATTAA--AATGACCCA-GA------ACCTTCTG-----A--TCAATGAA-CAAGTTACTTTAGGGATAACAGC-GCTATTTCTTTTTAAAGTTCACATCGACAAAGAAGCTTACGACCTCGATGTTGGATTAAGATACCCAAGTGGTGCAAAAGCTACTAATGGTTCGTTTGTTCAACGAATAATA-TCTTACGTGATCTGA---------------------       Eptatretus_burgeri         TTATTGTTTAATTA---------------ACATAATAAGTCCTACCTGCCCAG--TGAT-TTT--TTCAACGGCCAT-------CTCTGATGATAACGTAGCATAATCAATTGTCTATTAATTATAGACTAGAATGAATGGATTAACGAAAGCCAAA-----------------------CTGTCTCCTAATTTTAATCAATGAAATTATCCTTTCTGTTCAAAGACAGAAATTTTCTTATAAGACGAAAAGACCCTA-AAAGCTTCAAACATTAA----A-TTAACCTATCTAT-------------------AA-----AAACAAATAAAGAAAA--ATACTAATTT-TAAA---------------------------------------------A--ATTTGGTTGGGGCAACCAT-------------------TGAATAAAACTATCATCAATAATTAAACGA--AA--A-----ATTT-TCTTA------------AAACAAAGTAACAACTTAATTAAA-----------TAAGAAACTTATTAA--AATGACCCA-GA------ATCTTCTG-----A--TCAATGAA-CAAGTTACTTTAGGGATAACAGC-GCTATTTCTTTTTAAAGTTCACATCGACAAAGAAGCTTACGACCTCGATGTTGGATTAAGATACCCAAGTGGTGCAAAAGCTACTAATGGTTCGTTTGTTCAACGAATAATA-TCTTACATGATCTGAGTTCAGACCGGAGTAATCC--       Eptatretus_stoutii         CGCTTATTGTTAAA------------TTAATATAATAAGTCCTACCTGCCCAG--TGAT-TT--T-TCAACGGCCAT-------CTCTGATGATAACGTAGCATAATCAATTGTCTATTAATTATAGACTAGAATGAATGGATTAACGAAAGCCAAA-----------------------CTGTCTCCTAATTTTTATCAATGAAATTATCCTTTCTGTTCAAAGACAGAAATCCTCTTATAAGACGAAAAGACCCTA-AAAGCTTCAAACATTAA----A-TTAACCTATCTGT----------------------AA--AAACAAATAAAGAAAA--ATACTAATTT-TAAA---AA--------------------------------------------TTTGGTTGGGGCAACCAT-------------------TGAATAAAACTATCATCAACAATTAAACGA--AA--A-----ATTT-TCTTA------------AAACAGAATTACAACTCAGTTAAA-----------TAAGAAACTTATTAA--AATGACCCA-GA------ATCCTCTG-----A--TCAATGAA-CAAGTTACTTTAGGGATAACAGC-GCTATTTCTTTTTAAAGTTCACATCGACAAAGAAGCTTACGACCTCGATGTTGGATTAAGATACCCAAGTGGTGCAAAAGCTACTAA--------------------------------------------------------------       Myxine_glutinosa           TTATTGTTATAAAA---------------ATATAATAAGTCCTACCTGCCCAG--TGAT-TTT--TTCAACGGCCAT-------CTTTGATGATAACGTAGCATAATCAATTGTCTATTAATTTTAGACTAGAATGAATGGATTAACGAAAGCCGAA-----------------------CTGTCTCCTATTTCCTATCAATGAAATTAACCTCTCTGTCCAAAGGCAGAGATAAGCCTATAAGACGAAAAGACCCTA-AAAGCTTCAAAT---------A-TTAGATTACCTAG---------------CTTAAA-----CAACAACTAAAGAAAA--GTTTTAATTCCTAAA---------------------------------------------A---TTTGGTTGGGGCAACCATTGATATTAAAGAATCATCAATAATTTAACTAATAACTTGCATAATATCA---------------------------------------GAGTTACATCTCAATTAAGTAAGAAACTTATAAAAATGACCCAGAAAACTGATCAA-TG------A---------------A--------CAAGTTACTTTAGGGATAACAGC-GCTATTTCTTTTTAAAGTCCCCATCGACAAAGAAGCTTACGACCTCGATGTTGGATTAAGATATCCAAGTGGTGCAAAAGCTACTAACGGTTCGTTTGTTCAACGAGTAATA-TCTTACATGATCTGAGTTCAGACCGGAGTAATCC--     Rubicundus_eos             ????????????????????????????????????????????????????????????????????????????????????????????????????????????????????????????????????????????????????????????????????????????????????????????????????????????????????????????????????????????????????????????????????????????????????????????????????????????????????????????????????????????????????????????????????????????????????????????????????????????????????????????????????????????????????????????????????????????????????????????????????????????????????????????????????????????????????????????????????????????????????????????????????????????????????????????????????????????????????????????????????????????????????????????????????????????????????????????????????????????????????????????????????????????????????????????????????       Rubicundus_lopheliae       -----------------------------ATATAATAAGTCATTCCTGCCCAG--TGAT-TT--TTTTAACGGCCAT-------CTTTGATGATAACGTAGCATAATCAATTGTCTATTAATTATAGACTAGAATGAACGGATTAACGAAAGCCGAA-----------------------CTGTCTCCTAAATTCCATCAATGAAATTAACCTCTCTGTTCAAAGGCAGAGATACTTTTATAAGACGAAAAGACCCTA-AAAGCTTCAAATACTAA----AATTAACTTATCTAT----------------------TA--TATCAAATAAAGAAAA--ATACTAATTT-TTAA---AA--------------------------------------------TTTGGTTGGGGCAACCAT-------------------AGATTAAAACTATCATCTATAATTAAATTA--GTCTA-----CCAC-TATAA------------TATTAGGGAAACACCTCTATTAAA-----------TAAGACACTTAATAT--AATGATCCA-GA------AATAACTG-----A--TCAATGAA-CAAGTTACTTTAGGGATAACAGC-GCTATTTCTTTCAAAAGTTCCTATCGACAAAGAAGCTTACGACCTCGATGTTGGATTAAGATACCCAAGTGGTGCAAAAGCTACTAAAGGTTCGTTTGTTCAACGAATAATA-TCTTACGTGATCTGAG--------------------       Neomyxine_biniplicata      -----------------------------TAATAATAAGTCCTACCTGCCCAG--TGATTTT--TTTCAACGGCCAT-------CTTTGATGATAACGTAGCATAATCAATTGTCTATTAACTGTAGACTTGAATGAATGGATTAACGAGAGCCAAA-----------------------CTGTCTCCTAATTTTAATCAATGAAACTAACCTTTCTGTCCAAAGGCAGAAATAAATATATAAGACGAAAAGACCCTA-AAAGCTTCAAATACTAA----A-TTAACCTTCTTGC----------------------GA--ATAACAACTTAAAGAA--CCACTAATTT-CTAA---AA--------------------------------------------TTTGGTTGGGGCAACCAT-------------------TTAAGCAAACCATCATCAATAATTAAACTT--A---A-----CCTT-AATGA------------TTCAAGAGTGACTACTCAATAAAC-----------TAAGAAACTTATTAA--TATGACCCAGGA------ATAATCTG-----A--TCAATGAA-CAAGTTACTTTAGGGATAACAGC-GCTATTTCTTTTCAAAGTTCCCATCGACAAAGAAGCTTACGACCTCGATGTTGGATTAAGATACCCTAGTGGTGCAAAAGCTACTAACGGTTCGTTTGTTCAACGATTAATA-TCTTACGTGATCTGAGTTCAGA------------CC     Myxineidus                 ????????????????????????????????????????????????????????????????????????????????????????????????????????????????????????????????????????????????????????????????????????????????????????????????????????????????????????????????????????????????????????????????????????????????????????????????????????????????????????????????????????????????????????????????????????????????????????????????????????????????????????????????????????????????????????????????????????????????????????????????????????????????????????????????????????????????????????????????????????????????????????????????????????????????????????????????????????????????????????????????????????????????????????????????????????????????????????????????????????????????????????????????????????????????????????????????????     Gilpichthys                ????????????????????????????????????????????????????????????????????????????????????????????????????????????????????????????????????????????????????????????????????????????????????????????????????????????????????????????????????????????????????????????????????????????????????????????????????????????????????????????????????????????????????????????????????????????????????????????????????????????????????????????????????????????????????????????????????????????????????????????????????????????????????????????????????????????????????????????????????????????????????????????????????????????????????????????????????????????????????????????????????????????????????????????????????????????????????????????????????????????????????????????????????????????????????????????????????       Lethenteron_camtschaticum  CTCCAGATAAGAAT------------CACCTATTGGAGGCAAGACCTGCCCAA--TGAT-TAATATTGAATGGCCGCGGTACTTTGACCGTGTAAAAGTAGCGTAATCACTTGTCTTGTAAATTAAGACTGGAATGAAAGGTTACACGAGGGCATAA-----------------------CTGTCTCCTTATCCCTATCAATGAAATTGACCTACCCGTGCAAAGGCGGGTATAAACCCATAAGACGAGAAGACCCTGTGGAGCTTCCAAACATTTAC--A-TCGCATAATCATT-----------------------------------ATTCACG--ATGCACAGTT------------------------------------------------------TTAGGTTGGGGCAACCAC-------------------GGAACAAAAGTAATATCCACGACGACGAAA--AT--A--CA-ATTT-TCTTA------------ACCTAGAGTTACAACTCTAAGCAC-----------TAGTAAAACTAACGTTAATAGACCCA-GC------ATCACTTG-CTG-C--CTAACGAAACAAGTTACCCCAGGGATAACAGC-GCAATCCTTTCCACGAGCCCGAATCAACGAAAGGGTTTACGACCTCGATGTTGGATCGGGGCACCCCAATGGCGCAAAAGCTATTAAAGGTTCGTTTGTTCAACGATTAAAG-CCCCACGTGATCTGAGTTCAGACCGGAGTAATCC--       Petromyzon_marinus         CTCCAGATAAAAAT------------CAAGTATTGGAGGCAAGACCTGCCCAA--TGAT-TAATATTGAATGGCCGCGGTACTTTGACCGTGTAAAAGTAGCGTAATCACTTGTCTTGTAAATTAAGACTGGAATGAAAGGTTACACGAGGGCATAA-----------------------CTGTCTCCTTATCCCTATCAATGAAATTGACCTACCCGTGCAAAGGCGGGTATAAACCCATAAGACGAGAAGACCCTGTGGAGCTTCCAAACATTTAC--A-TCGAATAATAATT-----------------------------------ATTTACG--ATGTACAGTT------------------------------------------------------TTAGGTTGGGGCAACCAC-------------------GGAACAAAAGTAATATCCACGACGACGAAAATAT--A-----ATTT-TCTAA------------GCCTAGAACCACAACTCTAAGCAC-----------TAGTAAAACTAACGTTAATAGACCCA-GC------ATCACTTGCTGACT----AACGAAACAAGTTACCCCAGGGATAACAGC-GCAATCCTTTCCACGAGCCCGAATCAACGAAAGGGTTTACGACCTCGATGTTGGATCGGGGCACCCCAATGGCGCAAAAGCTATTAAAGGTTCGTTTGTTCAACGATTAAAG-CCCCACGTGATCTGAGTTCAGACCGGAGTAATCC--       Lampetra_fluviatilis       CTCCAGATAAGAAT------------CGCCTATTGGAGGCAAGACCTGCCCAA--TGAT-TAATATTGAATGGCCGCGGTACTTTGACCGTGTAAAAGTAGCGTAATCACTTGTCTTGTAAATTAAGACTAGAATGAAAGGTTACACGAGGGCATAA-----------------------CTGTCTCCTTATCCCTATCAATGAAATTGACCTACCCGTGCAAAGGCGGGTATAAACCCATAAGACGAGAAGACCCTGTGGAGCTTCCAAACATTTAC--A-TCGCATAATCATT-----------------------------------ATTCACG--ATGCACAGTT------------------------------------------------------TTAGGTTGGGGCAACCAC-------------------GGAACAAAAGTAATATCCACGACGACGAAA--AT--A--CA-ATTT-TCTTA------------GCCTAGAGTTACAACTCTAAGCAC-----------TAGTAGAACTAACGTTAATAGACCCA-GC------ATCACTTG-CTG-C--CTAACGAAACAAGTTACCCCAGGGATAACAGC-GCAATCCTTTCCACGAGCCCGAATCAACGAAAGGGTTTACGACCTCGATGTTGGATCGGGGCACCCCAATGGCGCAAAAGCTATTAAAGGTTCGTTTGTTCAACGATTAAAG-CCCCACGTGATCTGAGTTCAGACCGGAGTAATCC--       Geotria_australis          CTCCTGTTATACAA---ATTAAACA-CACAAATGAGAGGTAAGACCTGCCCAAT-GAAA-AATATTTCAATGGCCGCGGTACTTTGACCGTGTAAAAGTAGCGTAATCACTTGTCTTGTAAATTAAGACTGGAATGAAAGGTTACACGAGGGCATAA-----------------------CTGTCTCCTTATCCTGATCAATGAAATTGACCTACCCGTGCAAAGGCAGGTATATGTACATAAGACGAGAAGACCCTGTGGAGCTTCCAAACATTA-----------------------------------GTGCC-----GAGTAGCCCCATCACC--GGTACGCAGT--------------------------------------------------T---TTAGGTTGGGGCAACCAC-------------------GGAACAAAAGTAACATCCATGACGATGAAA--AT--A--TA-ATTT-TCTCA------------GCTTAGAGTGACAACTCAAAATAC-----------TAGTATGACTAACGTTAATAGACCCA-GT------ATAACTTA-CTG-C--TTTACGAAACAAGTTACCCCAGGGATAACAGC-GCAATCCTTTCCACGAGCTCGAATCAACGAAAGGGTTTACGACCTCGATGTTGGATCGGGGCACCCCAATGGCGCAAAAGCTATTAAAGGTTCGTTTGTTCAACGATTAAAG-CCCCACGTGATCTGAGTTCAGACCGGAGTAATCC--     Ichthyomyzon_bdellium      ????????????????????????????????????????????????????????????????????????????????????????????????????????????????????????????????????????????????????????????????????????????????????????????????????????????????????????????????????????????????????????????????????????????????????????????????????????????????????????????????????????????????????????????????????????????????????????????????????????????????????????????????????????????????????????????????????????????????????????????????????????????????????????????????????????????????????????????????????????????????????????????????????????????????????????????????????????????????????????????????????????????????????????????????????????????????????????????????????????????????????????????????????????????????????????????????????     Ichthyomyzon_castaneus     ????????????????????????????????????????????????????????????????????????????????????????????????????????????????????????????????????????????????????????????????????????????????????????????????????????????????????????????????????????????????????????????????????????????????????????????????????????????????????????????????????????????????????????????????????????????????????????????????????????????????????????????????????????????????????????????????????????????????????????????????????????????????????????????????????????????????????????????????????????????????????????????????????????????????????????????????????????????????????????????????????????????????????????????????????????????????????????????????????????????????????????????????????????????????????????????????????     Ichthyomyzon_unicuspis     ????????????????????????????????????????????????????????????????????????????????????????????????????????????????????????????????????????????????????????????????????????????????????????????????????????????????????????????????????????????????????????????????????????????????????????????????????????????????????????????????????????????????????????????????????????????????????????????????????????????????????????????????????????????????????????????????????????????????????????????????????????????????????????????????????????????????????????????????????????????????????????????????????????????????????????????????????????????????????????????????????????????????????????????????????????????????????????????????????????????????????????????????????????????????????????????????????     Mordacia_mordax            ????????????????????????????????????????????????????????????????????????????????????????????????????????????????????????????????????????????????????????????????????????????????????????????????????????????????????????????????????????????????????????????????????????????????????????????????????????????????????????????????????????????????????????????????????????????????????????????????????????????????????????????????????????????????????????????????????????????????????????????????????????????????????????????????????????????????????????????????????????????????????????????????????????????????????????????????????????????????????????????????????????????????????????????????????????????????????????????????????????????????????????????????????????????????????????????????????     Mordacia_lapicida          ????????????????????????????????????????????????????????????????????????????????????????????????????????????????????????????????????????????????????????????????????????????????????????????????????????????????????????????????????????????????????????????????????????????????????????????????????????????????????????????????????????????????????????????????????????????????????????????????????????????????????????????????????????????????????????????????????????????????????????????????????????????????????????????????????????????????????????????????????????????????????????????????????????????????????????????????????????????????????????????????????????????????????????????????????????????????????????????????????????????????????????????????????????????????????????????????????     Caspiomyzon_wagneri        ????????????????????????????????????????????????????????????????????????????????????????????????????????????????????????????????????????????????????????????????????????????????????????????????????????????????????????????????????????????????????????????????????????????????????????????????????????????????????????????????????????????????????????????????????????????????????????????????????????????????????????????????????????????????????????????????????????????????????????????????????????????????????????????????????????????????????????????????????????????????????????????????????????????????????????????????????????????????????????????????????????????????????????????????????????????????????????????????????????????????????????????????????????????????????????????????????     Tetrapleurodon_spadiceus   ????????????????????????????????????????????????????????????????????????????????????????????????????????????????????????????????????????????????????????????????????????????????????????????????????????????????????????????????????????????????????????????????????????????????????????????????????????????????????????????????????????????????????????????????????????????????????????????????????????????????????????????????????????????????????????????????????????????????????????????????????????????????????????????????????????????????????????????????????????????????????????????????????????????????????????????????????????????????????????????????????????????????????????????????????????????????????????????????????????????????????????????????????????????????????????????????????     Entosphenus_macrostomus    ????????????????????????????????????????????????????????????????????????????????????????????????????????????????????????????????????????????????????????????????????????????????????????????????????????????????????????????????????????????????????????????????????????????????????????????????????????????????????????????????????????????????????????????????????????????????????????????????????????????????????????????????????????????????????????????????????????????????????????????????????????????????????????????????????????????????????????????????????????????????????????????????????????????????????????????????????????????????????????????????????????????????????????????????????????????????????????????????????????????????????????????????????????????????????????????????????     Entosphenus_minimus        ????????????????????????????????????????????????????????????????????????????????????????????????????????????????????????????????????????????????????????????????????????????????????????????????????????????????????????????????????????????????????????????????????????????????????????????????????????????????????????????????????????????????????????????????????????????????????????????????????????????????????????????????????????????????????????????????????????????????????????????????????????????????????????????????????????????????????????????????????????????????????????????????????????????????????????????????????????????????????????????????????????????????????????????????????????????????????????????????????????????????????????????????????????????????????????????????????     Entosphenus_similis        ????????????????????????????????????????????????????????????????????????????????????????????????????????????????????????????????????????????????????????????????????????????????????????????????????????????????????????????????????????????????????????????????????????????????????????????????????????????????????????????????????????????????????????????????????????????????????????????????????????????????????????????????????????????????????????????????????????????????????????????????????????????????????????????????????????????????????????????????????????????????????????????????????????????????????????????????????????????????????????????????????????????????????????????????????????????????????????????????????????????????????????????????????????????????????????????????????     Entosphenus_tridentatus    ????????????????????????????????????????????????????????????????????????????????????????????????????????????????????????????????????????????????????????????????????????????????????????????????????????????????????????????????????????????????????????????????????????????????????????????????????????????????????????????????????????????????????????????????????????????????????????????????????????????????????????????????????????????????????????????????????????????????????????????????????????????????????????????????????????????????????????????????????????????????????????????????????????????????????????????????????????????????????????????????????????????????????????????????????????????????????????????????????????????????????????????????????????????????????????????????????     Eudontomyzon_danfordi      ????????????????????????????????????????????????????????????????????????????????????????????????????????????????????????????????????????????????????????????????????????????????????????????????????????????????????????????????????????????????????????????????????????????????????????????????????????????????????????????????????????????????????????????????????????????????????????????????????????????????????????????????????????????????????????????????????????????????????????????????????????????????????????????????????????????????????????????????????????????????????????????????????????????????????????????????????????????????????????????????????????????????????????????????????????????????????????????????????????????????????????????????????????????????????????????????????     Eudontomyzon_morii         ????????????????????????????????????????????????????????????????????????????????????????????????????????????????????????????????????????????????????????????????????????????????????????????????????????????????????????????????????????????????????????????????????????????????????????????????????????????????????????????????????????????????????????????????????????????????????????????????????????????????????????????????????????????????????????????????????????????????????????????????????????????????????????????????????????????????????????????????????????????????????????????????????????????????????????????????????????????????????????????????????????????????????????????????????????????????????????????????????????????????????????????????????????????????????????????????????     Lampetra_ayresii           ????????????????????????????????????????????????????????????????????????????????????????????????????????????????????????????????????????????????????????????????????????????????????????????????????????????????????????????????????????????????????????????????????????????????????????????????????????????????????????????????????????????????????????????????????????????????????????????????????????????????????????????????????????????????????????????????????????????????????????????????????????????????????????????????????????????????????????????????????????????????????????????????????????????????????????????????????????????????????????????????????????????????????????????????????????????????????????????????????????????????????????????????????????????????????????????????????     Mesomyzon                  ????????????????????????????????????????????????????????????????????????????????????????????????????????????????????????????????????????????????????????????????????????????????????????????????????????????????????????????????????????????????????????????????????????????????????????????????????????????????????????????????????????????????????????????????????????????????????????????????????????????????????????????????????????????????????????????????????????????????????????????????????????????????????????????????????????????????????????????????????????????????????????????????????????????????????????????????????????????????????????????????????????????????????????????????????????????????????????????????????????????????????????????????????????????????????????????????????     Yanliaomyzon_ingensdentes  ????????????????????????????????????????????????????????????????????????????????????????????????????????????????????????????????????????????????????????????????????????????????????????????????????????????????????????????????????????????????????????????????????????????????????????????????????????????????????????????????????????????????????????????????????????????????????????????????????????????????????????????????????????????????????????????????????????????????????????????????????????????????????????????????????????????????????????????????????????????????????????????????????????????????????????????????????????????????????????????????????????????????????????????????????????????????????????????????????????????????????????????????????????????????????????????????????     Yanliaomyzon_occisor     ????????????????????????????????????????????????????????????????????????????????????????????????????????????????????????????????????????????????????????????????????????????????????????????????????????????????????????????????????????????????????????????????????????????????????????????????????????????????????????????????????????????????????????????????????????????????????????????????????????????????????????????????????????????????????????????????????????????????????????????????????????????????????????????????????????????????????????????????????????????????????????????????????????????????????????????????????????????????????????????????????????????????????????????????????????????????????????????????????????????????????????????????????????????????????????????????????     Priscomyzon                ????????????????????????????????????????????????????????????????????????????????????????????????????????????????????????????????????????????????????????????????????????????????????????????????????????????????????????????????????????????????????????????????????????????????????????????????????????????????????????????????????????????????????????????????????????????????????????????????????????????????????????????????????????????????????????????????????????????????????????????????????????????????????????????????????????????????????????????????????????????????????????????????????????????????????????????????????????????????????????????????????????????????????????????????????????????????????????????????????????????????????????????????????????????????????????????????????     Mayomyzon                  ????????????????????????????????????????????????????????????????????????????????????????????????????????????????????????????????????????????????????????????????????????????????????????????????????????????????????????????????????????????????????????????????????????????????????????????????????????????????????????????????????????????????????????????????????????????????????????????????????????????????????????????????????????????????????????????????????????????????????????????????????????????????????????????????????????????????????????????????????????????????????????????????????????????????????????????????????????????????????????????????????????????????????????????????????????????????????????????????????????????????????????????????????????????????????????????????????     Hardistiella               ????????????????????????????????????????????????????????????????????????????????????????????????????????????????????????????????????????????????????????????????????????????????????????????????????????????????????????????????????????????????????????????????????????????????????????????????????????????????????????????????????????????????????????????????????????????????????????????????????????????????????????????????????????????????????????????????????????????????????????????????????????????????????????????????????????????????????????????????????????????????????????????????????????????????????????????????????????????????????????????????????????????????????????????????????????????????????????????????????????????????????????????????????????????????????????????????????     Pipiscius                  ????????????????????????????????????????????????????????????????????????????????????????????????????????????????????????????????????????????????????????????????????????????????????????????????????????????????????????????????????????????????????????????????????????????????????????????????????????????????????????????????????????????????????????????????????????????????????????????????????????????????????????????????????????????????????????????????????????????????????????????????????????????????????????????????????????????????????????????????????????????????????????????????????????????????????????????????????????????????????????????????????????????????????????????????????????????????????????????????????????????????????????????????????????????????????????????????????              [CO1, 721 sites]     Euconodonta                ?????????????????????????????????????????????????????????????????????????????????????????????????????????????????????????????????????????????????????????????????????????????????????????????????????????????????????????????????????????????????????????????????????????????????????????????????????????????????????????????????????????????????????????????????????????????????????????????????????????????????????????????????????????????????????????????????????????????????????????????????????????????????????????????????????????????????????????????????????????????????????????????????????????????????????????????????????????????????????????????????????????????????????????????????????????????????????????????????????????????????     Jamoytius                  ?????????????????????????????????????????????????????????????????????????????????????????????????????????????????????????????????????????????????????????????????????????????????????????????????????????????????????????????????????????????????????????????????????????????????????????????????????????????????????????????????????????????????????????????????????????????????????????????????????????????????????????????????????????????????????????????????????????????????????????????????????????????????????????????????????????????????????????????????????????????????????????????????????????????????????????????????????????????????????????????????????????????????????????????????????????????????????????????????????????????????     Euphanerops                ?????????????????????????????????????????????????????????????????????????????????????????????????????????????????????????????????????????????????????????????????????????????????????????????????????????????????????????????????????????????????????????????????????????????????????????????????????????????????????????????????????????????????????????????????????????????????????????????????????????????????????????????????????????????????????????????????????????????????????????????????????????????????????????????????????????????????????????????????????????????????????????????????????????????????????????????????????????????????????????????????????????????????????????????????????????????????????????????????????????????????     Achanarella                ?????????????????????????????????????????????????????????????????????????????????????????????????????????????????????????????????????????????????????????????????????????????????????????????????????????????????????????????????????????????????????????????????????????????????????????????????????????????????????????????????????????????????????????????????????????????????????????????????????????????????????????????????????????????????????????????????????????????????????????????????????????????????????????????????????????????????????????????????????????????????????????????????????????????????????????????????????????????????????????????????????????????????????????????????????????????????????????????????????????????????     Ciderius                   ?????????????????????????????????????????????????????????????????????????????????????????????????????????????????????????????????????????????????????????????????????????????????????????????????????????????????????????????????????????????????????????????????????????????????????????????????????????????????????????????????????????????????????????????????????????????????????????????????????????????????????????????????????????????????????????????????????????????????????????????????????????????????????????????????????????????????????????????????????????????????????????????????????????????????????????????????????????????????????????????????????????????????????????????????????????????????????????????????????????????????     Cornovichthys              ?????????????????????????????????????????????????????????????????????????????????????????????????????????????????????????????????????????????????????????????????????????????????????????????????????????????????????????????????????????????????????????????????????????????????????????????????????????????????????????????????????????????????????????????????????????????????????????????????????????????????????????????????????????????????????????????????????????????????????????????????????????????????????????????????????????????????????????????????????????????????????????????????????????????????????????????????????????????????????????????????????????????????????????????????????????????????????????????????????????????????     Lasanius                   ?????????????????????????????????????????????????????????????????????????????????????????????????????????????????????????????????????????????????????????????????????????????????????????????????????????????????????????????????????????????????????????????????????????????????????????????????????????????????????????????????????????????????????????????????????????????????????????????????????????????????????????????????????????????????????????????????????????????????????????????????????????????????????????????????????????????????????????????????????????????????????????????????????????????????????????????????????????????????????????????????????????????????????????????????????????????????????????????????????????????????     Birkenia                   ?????????????????????????????????????????????????????????????????????????????????????????????????????????????????????????????????????????????????????????????????????????????????????????????????????????????????????????????????????????????????????????????????????????????????????????????????????????????????????????????????????????????????????????????????????????????????????????????????????????????????????????????????????????????????????????????????????????????????????????????????????????????????????????????????????????????????????????????????????????????????????????????????????????????????????????????????????????????????????????????????????????????????????????????????????????????????????????????????????????????????     Rhyncholepis               ?????????????????????????????????????????????????????????????????????????????????????????????????????????????????????????????????????????????????????????????????????????????????????????????????????????????????????????????????????????????????????????????????????????????????????????????????????????????????????????????????????????????????????????????????????????????????????????????????????????????????????????????????????????????????????????????????????????????????????????????????????????????????????????????????????????????????????????????????????????????????????????????????????????????????????????????????????????????????????????????????????????????????????????????????????????????????????????????????????????????????     Myxinikela                 ?????????????????????????????????????????????????????????????????????????????????????????????????????????????????????????????????????????????????????????????????????????????????????????????????????????????????????????????????????????????????????????????????????????????????????????????????????????????????????????????????????????????????????????????????????????????????????????????????????????????????????????????????????????????????????????????????????????????????????????????????????????????????????????????????????????????????????????????????????????????????????????????????????????????????????????????????????????????????????????????????????????????????????????????????????????????????????????????????????????????????     Tethymyxine                ?????????????????????????????????????????????????????????????????????????????????????????????????????????????????????????????????????????????????????????????????????????????????????????????????????????????????????????????????????????????????????????????????????????????????????????????????????????????????????????????????????????????????????????????????????????????????????????????????????????????????????????????????????????????????????????????????????????????????????????????????????????????????????????????????????????????????????????????????????????????????????????????????????????????????????????????????????????????????????????????????????????????????????????????????????????????????????????????????????????????????       Paramyxine_fernholmi       -------------------CACAAAGACATTGGCACCCTTTACCTAATTTTTGGTGCATGAGCCGGAATAATCGGAACAGCTTTAAGTGTAATTATTCGAACAGAACTAAGCCAACCAGGACCTTTAATTAACAATGACC----------AACTTTATAATACAATCATTACAGCCCATGCATTCATTATAATTTTCTTTATAGTTATACCAATTATAATTGGTGGTTTCGGAAACTGATTAGTACCATTAATAATTGGTGCACCAGACATAGCAT--TTCCACGAATAAACAATATAAGCTTCTGACTTCTTCCACCTTCACTCCTTCTTTTACTCTCATCTTCCATAATTAGCTCTG---GTGCAGGAACTGGGTGAACTGTTTACCCACCCCTTTCAAATCATATTTCACATATAGGTCCATCAGTAGACTTAACTATTTTCTCACTACACCTAGCAGGTGTTTCCTCCATTTTAGGAGCGATCAACTTTATCACTACTATTATTAACATAAAAATACAATCAATAACCATATACCACATCCCCTTATTTGTGTGATCAATTTTAATTACCACAATTTTACTTCTTCTTTCCCTGCCAGTTTTAGCTGCTGCTATTACTATGCTGCTCACTGATCGTAATCTCAACACTACCTTTTTTGACCCTTCTGGCGGAGGAGACCCTATCCTTTATCAACACCTTTTCTGATTCTTTGGCCAC       Eptatretus_burgeri         ATGATTATTTTCTACTAATCACAAAGACATTGGCACCCTTTACCTAATTTTTGGTGCATGAGCCGGAATAATCGGAACAGCTTTAAGTGTAATTATTCGAACAGAACTAAGCCAACCAGGACCCTTAATCAACAATGACC----------AGCTTTATAATACAATCATTACAGCCCATGCATTTATTATAATTTTCTTCATAGTTATACCAATTATGATTGGAGGTTTCGGAAATTGATTAGTCCCACTAATAATTGGTGCGCCAGACATAGCAT--TTCCACGAATAAATAATATAAGCTTTTGACTTTTACCACCTTCACTCCTTCTATTACTTTCATCTTCCATAATTAGTTCTG---GTGCAGGAACTGGGTGAACTGTCTACCCACCCCTTTCAAATCATATTTCACATATAGGCCCATCAGTAGACTTAACCATTTTCTCACTACACCTAGCAGGTGTTTCTTCAATTTTAGGAGCAATCAACTTTATCACTACTATCATTAACATAAAAATACAGTCAATAACCATATACCATATTCCATTATTTGTTTGATCAATTCTAATTACCACAATTTTACTTCTTCTTTCCCTTCCAGTTTTAGCCGCTGCTATCACTATACTACTTACTGATCGTAACCTTAATACCACTTTTTTTGATCCCTCCGGTGGAGGAGACCCTATCCTTTATCAACATCTTTTCTGATTTTTTGGACAT       Eptatretus_stoutii         ------------------------------------CCTTTATCTAATTTTTGGTGCATGAGCCGGAATAATCGGAACAGCTTTAAGTGTAATTATTCGAACAGAATTAAGCCAACCAGGGCCCTTAATTAACAATGACC----------AACTTTATAATACAATCATCACAGCCCATGCATTCATTATAATTTTCTTCATAGTTATACCAATTATAATTGGTGGTTTTGGAAACTGACTAGTACCATTAATAATTGGTGCACCAGATATAGCAT--TCCCACGAATAAACAATATAAGCTTCTGACTTCTTCCCCCTTCACTCCTTCTTCTACTTTCATCTTCCATAATTAGTTCTG---GTGCAGGAACTGGGTGAACTGTTTACCCACCCCTTTCAAATCATATTTCACATATAGGCCCATCAGTAGATTTAACTATTTTCTCACTACACCTAGCAGGTGTTTCTTCCATTTTAGGAGCAATCAACTTTATCACTACTATTATCAACATAAAAATACAATCAATAACCATATATCACATCCCATTATTTGTATGATCAATCCTAATCACCACAATTTTACTTCTCCTTTCCCTGCCAGTTTTAGCTGCTGCCATCACTATACTACTTACTGATCGTAATCTCAATACTACCTTTTTCGATCCTTCTGGTGGAGGAGATCCTATCCTTTATCAACACCT-------------------       Myxine_glutinosa           ATGATTTTTCTCCACCAATCATAAAGACATTGGTACCCTTTACCTTATTTTCGGGGCCTGAGCCGGAATAATTGGCACAGCCCTTAGCGTAATCATCCGAACAGAGTTGAGTCAGCCAGGATCCTTAATCAATAATGACC----------AACTCTATAATACAATTATCACAGCCCACGCATTTGTAATAATCTTTTTTATAGTAATACCTATCATAATCGGGGGCTTTGGGAACTGACTAGTCCCAATAATAATCGGCGCCCCTGATATAGCAT--TTCCCCGAATAAACAATATAAGCTTCTGACTTTTGCCCCCATCACTTATACTATTACTCTCCTCCTCACTAGTAAGCTCTG---GAGCAGGAACTGGATGAACAGTTTATCCCCCTCTCTCTAATCACATTTCTCACATAGGCCCTTCAGTAGACCTAGCTATTTTCTCCCTTCATCTAGCAGGAGTTTCCTCAATCTTAGGGGCAATCAACTTTATCACAACAATTATTAACATAAAAACACGGTCTATAGAAATATACCATATCCCATTATTTGTATGATCAATTCTAATCACCGCAATCCTACTTCTTTTATCCTTACCTGTTTTAGCCGCAGCTATCACTATACTCCTCACAGACCGTAATCTCAACACCACCTTCTTTGACCCCTCTGGTGGAGGGGATCCCATCCTCTATCAGCATTTATTTTGATTTTTTGGCCAC     Rubicundus_eos             ?????????????????????????????????????????????????????????????????????????????????????????????????????????????????????????????????????????????????????????????????????????????????????????????????????????????????????????????????????????????????????????????????????????????????????????????????????????????????????????????????????????????????????????????????????????????????????????????????????????????????????????????????????????????????????????????????????????????????????????????????????????????????????????????????????????????????????????????????????????????????????????????????????????????????????????????????????????????????????????????????????????????????????????????????????????????????????????????????????????????????       Rubicundus_lopheliae       -------------------CACAAAGACATTGGCACCCTCTATCTTATCTTTGGTGCATGAGCTGGAATAATTGGAACAGGTCTTAGTGTAATTATTCGTACAGAATTAAGCCAACCTGGACCCCTTATTAATAATGATC----------AACTTTATAATACAGTAATTACAGCTCATGCTTTTATTATAATTTTCTTTATAGTTATACCAGTCATAATTGGGGGATTTGGAAACTGATTAGTTCCTCTAATAATTGGAGCTCCAGATATAGCAT--TTCCTCGAATAAACAATATAAGCTTCTGACTTTTACCGCCTTCCCTCCTTCTTCTACTTTCTTCCTCTATAGTCGGATCAG---GGGCAGGCACAGGATGAACAGTATATCCGCCTCTTTCCAACCATATTTCACATATAGGACCATCAGTCGATCTAACTATTTTCTCCCTCCATTTAGCAGGTGTATCTTCTATTTTAGGAGCAATTAATTTTATTACCACAATCATCAACATGAAAATACAATCTATAACTATATATCATATCCCATTATTTGTTTGATCAGTCCTGATTACCGCAATTCTTCTTCTTTTATCCTTACCTGTTTTAGCAGCAGCTATTACTATACTTCTTACAGATCGAAATTTAAACACTACTTTTTTTGATCCTTCTGGTGGAGGAGACCCTATTCTTTACCAACACCTATTCTGATTCTTTGGCCAC       Neomyxine_biniplicata      -------------------CACAAAGACATTGGCACCCTTTACCTTATCTTTGGTGCTTGGGCCGGAATAATTGGCACAGCCCTTAGTGTAATTATTCGAACAGAGCTAAGTCAGCCTGGTGCCTTAATCGACAATGAGC----------AGCTTTATAACACAATTATTACAGCACATGCATTTATTATAATCTTCTTTATAGTTATACCGATCATAATCGGGGGGTTTGGAAACTGATTAGTCCCTTTAATAATTGGGGCACCAGACATAGCCT--TCCCTCGAATAAACAACATAAGCTTCTGATTACTTCCTCCATCACTCTTACTCTTACTTTCCTCTTCCCTTATCAGCACCG---GGGCAGGAACTGGTTGAACAGTTTACCCGCCTCTCTCTAACCATATCTCACATATAGGACCCTCGGTAGATTTAGCTATCTTTTCCTTACACTTAGCGGGAGTATCCTCAATCTTAGGGGCAATTAATTTTATTACTACAATTATTAACATAAAAATACAACCTATAGACATGTATAACACCCCTCTATTCGTATGATCAATCTTCATTACTGCTATTTTACTCCTACTCTCACTACCAGTATTAGCTGCTGCTGTTACAATACTACTTACAGACCGTAACCTAAATACTACATTCTTCGATCCTGCCGGCGGTGGGGATCCAATTCTCTACCAACACCTATTCTGATTCTTTGGCCAC     Myxineidus                 ?????????????????????????????????????????????????????????????????????????????????????????????????????????????????????????????????????????????????????????????????????????????????????????????????????????????????????????????????????????????????????????????????????????????????????????????????????????????????????????????????????????????????????????????????????????????????????????????????????????????????????????????????????????????????????????????????????????????????????????????????????????????????????????????????????????????????????????????????????????????????????????????????????????????????????????????????????????????????????????????????????????????????????????????????????????????????????????????????????????????????     Gilpichthys                ?????????????????????????????????????????????????????????????????????????????????????????????????????????????????????????????????????????????????????????????????????????????????????????????????????????????????????????????????????????????????????????????????????????????????????????????????????????????????????????????????????????????????????????????????????????????????????????????????????????????????????????????????????????????????????????????????????????????????????????????????????????????????????????????????????????????????????????????????????????????????????????????????????????????????????????????????????????????????????????????????????????????????????????????????????????????????????????????????????????????????       Lethenteron_camtschaticum  TTGATTATTCTCTACTAATCATAAAGACATCGGCACCCTATATCTAATTTTTGGGGCCTGAGCAGGAATAGTGGGAACCGCTTTAAGCATCCTAATTCGAGCAGAACTCAGTCAACCGGGCACTTTACTAGGAGATGACC----------AGATCTTTAATGTTATCGTAACCGCTCATGCTTTCGTTATAATTTTTTTTATAGTCATACCAATTATAATCGGAGGCTTCGGAAACTGACTTGTGCCTATAATACTTAGCGCCCCTGATATAGCCT--TCCCACGTATAAATAACATAAGCTTTTGACTGCTCCCACCATCCCTACTCTTACTTTTAGCTTCCGCAGGAGTTGAAGCAG---GAGCCGGAACTGGATGAACAGTATACCCACCTCTAGCAGGAAATTTAGCCCACACAGGGGCCTCTGTTGACTTAACAATTTTCTCCCTTCATCTAGCCGGTATTTCATCAATCCTTGGGGCAGTCAACTTTATTACAACAATTTTTAACATAAAACCTCCAACTATAACACAATACCAAACCCCATTATTTGTTTGATCCGTTTTAATTACTGCAGTCCTCCTTCTTCTATCACTTCCTGTACTTGCAGCTGCCATCACTATACTTTTAACAGATCGTAATTTAAATACATCCTTCTTTGACCCTGCAGGAGGAGGAGACCCAATCCTTTACCAACACCTGTTCTGATTCTTTGGGCAC       Petromyzon_marinus         TTGATTATTCTCTACTAATCACAAAGACATCGGCACCCTATATCTAATTTTCGGGGCCTGAGCAGGAATAGTAGGAACTGCTTTAAGTATTCTAATTCGAGCTGAACTAAGTCAGCCAGGCACTTTATTAGGAGACGACC----------AAATTTTTAATGTTATCGTAACTGCCCATGCCTTCGTCATAATCTTTTTTATAGTTATACCAATTATAATTGGAGGCTTTGGCAACTGACTTGTACCCCTAATACTTGGTGCTCCTGATATGGCCT--TCCCTCGTATAAACAACATAAGTTTTTGACTACTTCCGCCCTCTTTACTTTTACTCTTAGCCTCTGCAGGAGTTGAAGCTG---GGGCAGGAACAGGATGAACTGTATATCCTCCCTTAGCCGGAAACCTAGCCCACACCGGGGCCTCTGTCGACCTAACAATCTTTTCCTTACACTTAGCCGGAGTTTCATCAATTCTAGGAGCAGTTAATTTCATCACAACTATTTTTAACATGAAACCCCCAACTATGACTCAATACCAAACCCCCTTATTTGTTTGATCAGTCTTAATCACTGCAGTTCTTCTTCTTCTATCTCTACCAGTACTAGCAGCTGCTATCACAATACTTCTAACAGATCGTAACTTAAATACATCCTTCTTCGACCCTGCAGGAGGAGGAGACCCCATTCTTTACCAACACTTATTTTGATTCTTCGGACAC       Lampetra_fluviatilis       TTGATTATTCTCTACTAATCATAAAGACATCGGCACCCTATATCTAATCTTTGGGGCCTGAGCAGGAATAGTGGGAACTGCTTTAAGCATCCTAATTCGGGCAGAATTAAGTCAACCAGGCACTTTACTAGGAGACGATC----------AAATCTTTAACGTTATCGTAACCGCCCATGCTTTCGTTATAATCTTTTTTATAGTTATACCAATTATAATCGGAGGCTTCGGAAACTGACTTGTACCAATAATACTTAGCGCCCCAGATATAGCCT--TCCCACGTATAAACAACATAAGCTTTTGACTACTTCCACCCTCACTCCTTCTACTTTTAGCTTCCGCAGGAGTTGAAGCAG---GGGCCGGAACGGGATGAACCGTATACCCACCCCTAGCAGGAAATTTAGCCCACACAGGGGCCTCTGTTGACTTAACAATTTTCTCCCTTCACCTAGCTGGTATTTCATCAATTCTAGGGGCAGTCAACTTTATTACAACAATTTTTAATATAAAGCCCCCAACTATAACACAATACCAAATTCCTTTATTTGTTTGATCCGTTTTAATTACTGCAGTCCTCCTTCTTCTATCACTTCCTGTACTTGCAGCCGCCATTACTATACTTTTAACAGATCGTAATTTAAATACATCTTTCTTCGACCCTGCAGGGGGAGGAGACCCAATTCTTTACCAACACCTATTTTGATTCTTTGGACAT       Geotria_australis          TTGATTATTCTCCACTAATCACAAAGACATCGGCACCCTCTATCTTATCTTTGGGGCCTGAGCAGGAATAGTAGGAACCGCACTAAGTATCCTAATTCGAGCAGAACTAAGCCAACCCGGCACGCTATTAGGTGATGACC----------AAATCTACAATGTCATTGTTACAGCCCATGCTTTTGTTATAATTTTCTTTATAGTTATACCAATCATAATCGGAGGCTTTGGTAATTGATTAATTCCCCTAATACTTGGTGCCCCAGACATGGCAT--TCCCCCGAATAAATAACATAAGCTTCTGACTTCTACCCCCATCCCTTCTACTTCTTTTAGCCTCTTCAGGGGTCGAAGCCG---GAGCAGGTACAGGTTGGACAGTATACCCCCCGCTAGCAGGGAATCTTGCACACATAGGAGCCTCCGTTGACTTAACAATTTTCTCGCTACATCTTGCCGGGATCTCCTCAATTTTAGGAGCAATTAACTTCATTACTACTATTTTCAATATAAAACCCCCAACTATAACACAGTACCAAACACCCCTATTCGTGTGATCTGTATTAATTACCGCAGTTCTTCTCCTACTTGCACTACCAGTTCTAGCGGCTGCCATTACAATATTACTGACAGATCGAAATTTAAATACAGCCTTTTTCGACCCTGCTGGGGGAGGAGATCCCATTCTTTACCAACATTTATTCTGATTCTTCGGACAC     Ichthyomyzon_bdellium      ?????????????????????????????????????????????????????????????????????????????????????????????????????????????????????????????????????????????????????????????????????????????????????????????????????????????????????????????????????????????????????????????????????????????????????????????????????????????????????????????????????????????????????????????????????????????????????????????????????????????????????????????????????????????????????????????????????????????????????????????????????????????????????????????????????????????????????????????????????????????????????????????????????????????????????????????????????????????????????????????????????????????????????????????????????????????????????????????????????????????????     Ichthyomyzon_castaneus     ?????????????????????????????????????????????????????????????????????????????????????????????????????????????????????????????????????????????????????????????????????????????????????????????????????????????????????????????????????????????????????????????????????????????????????????????????????????????????????????????????????????????????????????????????????????????????????????????????????????????????????????????????????????????????????????????????????????????????????????????????????????????????????????????????????????????????????????????????????????????????????????????????????????????????????????????????????????????????????????????????????????????????????????????????????????????????????????????????????????????????     Ichthyomyzon_unicuspis     ?????????????????????????????????????????????????????????????????????????????????????????????????????????????????????????????????????????????????????????????????????????????????????????????????????????????????????????????????????????????????????????????????????????????????????????????????????????????????????????????????????????????????????????????????????????????????????????????????????????????????????????????????????????????????????????????????????????????????????????????????????????????????????????????????????????????????????????????????????????????????????????????????????????????????????????????????????????????????????????????????????????????????????????????????????????????????????????????????????????????????       Mordacia_mordax            ------------------------------------CCTTTATTTAATCTTCGGGGCTTGAGCAGGAATAGTAGGAACAGCATTAAGCATTCTAATCCGAGCAGAACTAAGTCAACCAGGAACGCTCCTAGGTGATGATC----------AAATCTACAATGTAATTGTTACAGCTCATGCTTTTATTATAATTTTTTTCATGGTAATACCCATCATAATAGGAGGCTTTGGGAATTGATTAACTCCTTTAATACTAGGTGCCCCAGATATAGCAT--TTCCTCGAATAAATAACATAAGTTTCTGACTTCTCCCTCCTTCCCTCCTTCTTCTCTTAGCATCTTCAGGTGTTGAAGCAG---GAGTAGGTACAGGCTGAACAGTCTATCCACCACTAGCAGGAAACTTAGCTCACACAGGAGCCTCTGTAGACTTGGCCATCTTTTCCCTACACCTTGCAGGTGTGTCATCAATTTTAGGAGCCATTAATTTTATCACAACAATTTTCAACATAAAACCCCCAACTATAACTATATATCACGTACCACTATTTATTTGATCAGTGTTAATTACAGCTGTTCTTCTTCTTCTTTCACTCCCAGTTTTAGCCGCAGCTATTACAATACTACTAACAGACCGTAATCTAAATACAACCTTCTTTGACCCAGCAGGTGGAGGAGACCCTATCTTATACCAACACCTA------------------     Mordacia_lapicida          ?????????????????????????????????????????????????????????????????????????????????????????????????????????????????????????????????????????????????????????????????????????????????????????????????????????????????????????????????????????????????????????????????????????????????????????????????????????????????????????????????????????????????????????????????????????????????????????????????????????????????????????????????????????????????????????????????????????????????????????????????????????????????????????????????????????????????????????????????????????????????????????????????????????????????????????????????????????????????????????????????????????????????????????????????????????????????????????????????????????????????     Caspiomyzon_wagneri        ?????????????????????????????????????????????????????????????????????????????????????????????????????????????????????????????????????????????????????????????????????????????????????????????????????????????????????????????????????????????????????????????????????????????????????????????????????????????????????????????????????????????????????????????????????????????????????????????????????????????????????????????????????????????????????????????????????????????????????????????????????????????????????????????????????????????????????????????????????????????????????????????????????????????????????????????????????????????????????????????????????????????????????????????????????????????????????????????????????????????????     Tetrapleurodon_spadiceus   ?????????????????????????????????????????????????????????????????????????????????????????????????????????????????????????????????????????????????????????????????????????????????????????????????????????????????????????????????????????????????????????????????????????????????????????????????????????????????????????????????????????????????????????????????????????????????????????????????????????????????????????????????????????????????????????????????????????????????????????????????????????????????????????????????????????????????????????????????????????????????????????????????????????????????????????????????????????????????????????????????????????????????????????????????????????????????????????????????????????????????     Entosphenus_macrostomus    ?????????????????????????????????????????????????????????????????????????????????????????????????????????????????????????????????????????????????????????????????????????????????????????????????????????????????????????????????????????????????????????????????????????????????????????????????????????????????????????????????????????????????????????????????????????????????????????????????????????????????????????????????????????????????????????????????????????????????????????????????????????????????????????????????????????????????????????????????????????????????????????????????????????????????????????????????????????????????????????????????????????????????????????????????????????????????????????????????????????????????     Entosphenus_minimus        ?????????????????????????????????????????????????????????????????????????????????????????????????????????????????????????????????????????????????????????????????????????????????????????????????????????????????????????????????????????????????????????????????????????????????????????????????????????????????????????????????????????????????????????????????????????????????????????????????????????????????????????????????????????????????????????????????????????????????????????????????????????????????????????????????????????????????????????????????????????????????????????????????????????????????????????????????????????????????????????????????????????????????????????????????????????????????????????????????????????????????     Entosphenus_similis        ?????????????????????????????????????????????????????????????????????????????????????????????????????????????????????????????????????????????????????????????????????????????????????????????????????????????????????????????????????????????????????????????????????????????????????????????????????????????????????????????????????????????????????????????????????????????????????????????????????????????????????????????????????????????????????????????????????????????????????????????????????????????????????????????????????????????????????????????????????????????????????????????????????????????????????????????????????????????????????????????????????????????????????????????????????????????????????????????????????????????????     Entosphenus_tridentatus    ?????????????????????????????????????????????????????????????????????????????????????????????????????????????????????????????????????????????????????????????????????????????????????????????????????????????????????????????????????????????????????????????????????????????????????????????????????????????????????????????????????????????????????????????????????????????????????????????????????????????????????????????????????????????????????????????????????????????????????????????????????????????????????????????????????????????????????????????????????????????????????????????????????????????????????????????????????????????????????????????????????????????????????????????????????????????????????????????????????????????????     Eudontomyzon_danfordi      ?????????????????????????????????????????????????????????????????????????????????????????????????????????????????????????????????????????????????????????????????????????????????????????????????????????????????????????????????????????????????????????????????????????????????????????????????????????????????????????????????????????????????????????????????????????????????????????????????????????????????????????????????????????????????????????????????????????????????????????????????????????????????????????????????????????????????????????????????????????????????????????????????????????????????????????????????????????????????????????????????????????????????????????????????????????????????????????????????????????????????     Eudontomyzon_morii         ?????????????????????????????????????????????????????????????????????????????????????????????????????????????????????????????????????????????????????????????????????????????????????????????????????????????????????????????????????????????????????????????????????????????????????????????????????????????????????????????????????????????????????????????????????????????????????????????????????????????????????????????????????????????????????????????????????????????????????????????????????????????????????????????????????????????????????????????????????????????????????????????????????????????????????????????????????????????????????????????????????????????????????????????????????????????????????????????????????????????????     Lampetra_ayresii           ?????????????????????????????????????????????????????????????????????????????????????????????????????????????????????????????????????????????????????????????????????????????????????????????????????????????????????????????????????????????????????????????????????????????????????????????????????????????????????????????????????????????????????????????????????????????????????????????????????????????????????????????????????????????????????????????????????????????????????????????????????????????????????????????????????????????????????????????????????????????????????????????????????????????????????????????????????????????????????????????????????????????????????????????????????????????????????????????????????????????????     Mesomyzon             ?????????????????????????????????????????????????????????????????????????????????????????????????????????????????????????????????????????????????????????????????????????????????????????????????????????????????????????????????????????????????????????????????????????????????????????????????????????????????????????????????????????????????????????????????????????????????????????????????????????????????????????????????????????????????????????????????????????????????????????????????????????????????????????????????????????????????????????????????????????????????????????????????????????????????????????????????????????????????????????????????????????????????????????????????????????????????????????????????????????????????     Yanliaomyzon_igensdentes  ?????????????????????????????????????????????????????????????????????????????????????????????????????????????????????????????????????????????????????????????????????????????????????????????????????????????????????????????????????????????????????????????????????????????????????????????????????????????????????????????????????????????????????????????????????????????????????????????????????????????????????????????????????????????????????????????????????????????????????????????????????????????????????????????????????????????????????????????????????????????????????????????????????????????????????????????????????????????????????????????????????????????????????????????????????????????????????????????????????????????????      Yanliaomyzon_occisor     ?????????????????????????????????????????????????????????????????????????????????????????????????????????????????????????????????????????????????????????????????????????????????????????????????????????????????????????????????????????????????????????????????????????????????????????????????????????????????????????????????????????????????????????????????????????????????????????????????????????????????????????????????????????????????????????????????????????????????????????????????????????????????????????????????????????????????????????????????????????????????????????????????????????????????????????????????????????????????????????????????????????????????????????????????????????????????????????????????????????????????     Priscomyzon                ?????????????????????????????????????????????????????????????????????????????????????????????????????????????????????????????????????????????????????????????????????????????????????????????????????????????????????????????????????????????????????????????????????????????????????????????????????????????????????????????????????????????????????????????????????????????????????????????????????????????????????????????????????????????????????????????????????????????????????????????????????????????????????????????????????????????????????????????????????????????????????????????????????????????????????????????????????????????????????????????????????????????????????????????????????????????????????????????????????????????????     Mayomyzon                  ?????????????????????????????????????????????????????????????????????????????????????????????????????????????????????????????????????????????????????????????????????????????????????????????????????????????????????????????????????????????????????????????????????????????????????????????????????????????????????????????????????????????????????????????????????????????????????????????????????????????????????????????????????????????????????????????????????????????????????????????????????????????????????????????????????????????????????????????????????????????????????????????????????????????????????????????????????????????????????????????????????????????????????????????????????????????????????????????????????????????????     Hardistiella               ?????????????????????????????????????????????????????????????????????????????????????????????????????????????????????????????????????????????????????????????????????????????????????????????????????????????????????????????????????????????????????????????????????????????????????????????????????????????????????????????????????????????????????????????????????????????????????????????????????????????????????????????????????????????????????????????????????????????????????????????????????????????????????????????????????????????????????????????????????????????????????????????????????????????????????????????????????????????????????????????????????????????????????????????????????????????????????????????????????????????????     Pipiscius                  ?????????????????????????????????????????????????????????????????????????????????????????????????????????????????????????????????????????????????????????????????????????????????????????????????????????????????????????????????????????????????????????????????????????????????????????????????????????????????????????????????????????????????????????????????????????????????????????????????????????????????????????????????????????????????????????????????????????????????????????????????????????????????????????????????????????????????????????????????????????????????????????????????????????????????????????????????????????????????????????????????????????????????????????????????????????????????????????????????????????????????       ;End; 
