# Supplementary material for: The rise of predation in Jurassic lampreys
Source: Nat Commun. 2023 Oct 31;14:6652. doi: 10.1038/s41467-023-42251-0 (PMC10618186; doi:10.1038/s41467-023-42251-0)
Supplement: Supplementary file 4 — Supplementary Code 1-8 [file 41467_2023_42251_MOESM4_ESM.zip › Supplementary Codes 1-8/Supplementary Code 2.rtf]

Supplementary Code 2: Total-evidence analyses command and log file                            MrBayes 3.2.7b x86_64                       (Bayesian Analysis of Phylogeny)                              (Parallel version)                         (8 processors available)               Distributed under the GNU General Public License                 Type "help" or "help <command>" for information                     on the commands that are available.                    Type "about" for authorship and general                       information about the program.    Executing file "run.nex"   UNIX line termination   Longest line length = 79   Parsing file   Expecting NEXUS formatted file   Reading mrbayes block       Executing file "../data/morph_molec.nex"...    UNIX line termination   Longest line length = 801   Parsing file   Expecting NEXUS formatted file   Reading data block      Allocated taxon set      Allocated matrix      Defining new matrix with 45 taxa and 1701 characters      Data is Mixed         Data for partition 1 is Standard         Data for partition 2 is Dna      There are a total of 2 default data divisions      Data matrix is interleaved      Gaps coded as -      Missing data coded as ?      Taxon  1 -> Euconodonta      Taxon  2 -> Jamoytius      Taxon  3 -> Euphanerops      Taxon  4 -> Achanarella      Taxon  5 -> Ciderius      Taxon  6 -> Cornovichthys      Taxon  7 -> Lasanius      Taxon  8 -> Birkenia      Taxon  9 -> Rhyncholepis      Taxon 10 -> Myxinikela      Taxon 11 -> Tethymyxine      Taxon 12 -> Paramyxine_fernholmi      Taxon 13 -> Eptatretus_burgeri      Taxon 14 -> Eptatretus_stoutii      Taxon 15 -> Myxine_glutinosa      Taxon 16 -> Rubicundus_eos      Taxon 17 -> Rubicundus_lopheliae      Taxon 18 -> Neomyxine_biniplicata      Taxon 19 -> Myxineidus      Taxon 20 -> Gilpichthys      Taxon 21 -> Lethenteron_camtschaticum      Taxon 22 -> Petromyzon_marinus      Taxon 23 -> Lampetra_fluviatilis      Taxon 24 -> Geotria_australis      Taxon 25 -> Ichthyomyzon_bdellium      Taxon 26 -> Ichthyomyzon_castaneus      Taxon 27 -> Ichthyomyzon_unicuspis      Taxon 28 -> Mordacia_mordax      Taxon 29 -> Mordacia_lapicida      Taxon 30 -> Caspiomyzon_wagneri      Taxon 31 -> Tetrapleurodon_spadiceus      Taxon 32 -> Entosphenus_macrostomus      Taxon 33 -> Entosphenus_minimus      Taxon 34 -> Entosphenus_similis      Taxon 35 -> Entosphenus_tridentatus      Taxon 36 -> Eudontomyzon_danfordi      Taxon 37 -> Eudontomyzon_morii      Taxon 38 -> Lampetra_ayresii      Taxon 39 -> Mesomyzon      Taxon 40 -> Yanliaomyzon_igensdentes      Taxon 41 -> Yanliaomyzon_occisor      Taxon 42 -> Priscomyzon      Taxon 43 -> Mayomyzon      Taxon 44 -> Hardistiella      Taxon 45 -> Pipiscius      Successfully read matrix      Setting default partition, dividing characters into 2 parts      Setting model defaults      Seed (for generating default start values) = 1654745601      WARNING: There are 76 characters incompatible with the specified               coding bias. These characters will be excluded.      Setting output file names to "../data/morph_molec.nex.run<i>.<p|t>"   Exiting data block   Reached end of file    Returning execution to calling file ...       Defining charset called 'MVothers'      Defining charset called 'BranchialApparatus'      Defining charset called 'FeedingMechanism'      Defining charset called '16S'      Defining charset called 'CO1'      Defining partition called 'five'      Setting five as the partition, dividing characters into 5 parts.      Setting model defaults      Seed (for generating default start values) = 235116259      WARNING: There are 61 characters incompatible with the specified               coding bias. These characters will be excluded.      WARNING: There are 10 characters incompatible with the specified               coding bias. These characters will be excluded.      WARNING: There are 5 characters incompatible with the specified               coding bias. These characters will be excluded.      Enabling Coding Variable for partition 1      Enabling Coding Variable for partition 2      Enabling Coding Variable for partition 3      Setting Rates to Gamma for partition 1      Setting Rates to Gamma for partition 2      Setting Rates to Gamma for partition 3      Successfully set likelihood model parameters to      partitions 1, 2, and 3 (if applicable)      WARNING: There are 61 characters incompatible with the specified               coding bias. These characters will be excluded.      WARNING: There are 10 characters incompatible with the specified               coding bias. These characters will be excluded.      WARNING: There are 5 characters incompatible with the specified               coding bias. These characters will be excluded.      Setting Nst to 2 for partition 4      Setting Nst to 2 for partition 5      Setting Rates to Gamma for partition 4      Setting Rates to Gamma for partition 5      Successfully set likelihood model parameters to      partitions 4 and 5 (if applicable)      WARNING: There are 61 characters incompatible with the specified               coding bias. These characters will be excluded.      WARNING: There are 10 characters incompatible with the specified               coding bias. These characters will be excluded.      WARNING: There are 5 characters incompatible with the specified               coding bias. These characters will be excluded.      Unlinking      WARNING: There are 61 characters incompatible with the specified               coding bias. These characters will be excluded.      WARNING: There are 10 characters incompatible with the specified               coding bias. These characters will be excluded.      WARNING: There are 5 characters incompatible with the specified               coding bias. These characters will be excluded.      Setting Ratepr to Variable [Dirichlet(..,1,..)] for partition 1      Setting Ratepr to Variable [Dirichlet(..,1,..)] for partition 2      Setting Ratepr to Variable [Dirichlet(..,1,..)] for partition 3      Setting Ratepr to Variable [Dirichlet(..,1,..)] for partition 4      Setting Ratepr to Variable [Dirichlet(..,1,..)] for partition 5      Successfully set prior model parameters to all      applicable data partitions       WARNING: There are 61 characters incompatible with the specified               coding bias. These characters will be excluded.      WARNING: There are 10 characters incompatible with the specified               coding bias. These characters will be excluded.      WARNING: There are 5 characters incompatible with the specified               coding bias. These characters will be excluded.      Defining constraint called 'ingroup'      Setting Clockratepr to Lognormal(-6.00,1.00)      Setting Clockratepr to Lognormal(-6.00,1.00)      Setting Clockratepr to Lognormal(-6.00,1.00)      Setting Clockratepr to Lognormal(-6.00,1.00)      Setting Clockratepr to Lognormal(-6.00,1.00)      Successfully set prior model parameters to all      applicable data partitions       WARNING: There are 61 characters incompatible with the specified               coding bias. These characters will be excluded.      WARNING: There are 10 characters incompatible with the specified               coding bias. These characters will be excluded.      WARNING: There are 5 characters incompatible with the specified               coding bias. These characters will be excluded.      Setting Clockvarpr to ILN for partition 1      Setting Clockvarpr to ILN for partition 2      Setting Clockvarpr to ILN for partition 3      Setting Clockvarpr to ILN for partition 4      Setting Clockvarpr to ILN for partition 5      Successfully set prior model parameters to all      applicable data partitions       WARNING: There are 61 characters incompatible with the specified               coding bias. These characters will be excluded.      WARNING: There are 10 characters incompatible with the specified               coding bias. These characters will be excluded.      WARNING: There are 5 characters incompatible with the specified               coding bias. These characters will be excluded.      Unlinking      WARNING: There are 61 characters incompatible with the specified               coding bias. These characters will be excluded.      WARNING: There are 10 characters incompatible with the specified               coding bias. These characters will be excluded.      WARNING: There are 5 characters incompatible with the specified               coding bias. These characters will be excluded.      Setting age of taxon 'Euconodonta' to Fixed(535.50)      Setting age of taxon 'Jamoytius' to Fixed(435.40)      Setting age of taxon 'Euphanerops' to Fixed(370.00)      Setting age of taxon 'Achanarella' to Fixed(385.00)      Setting age of taxon 'Ciderius' to Fixed(432.00)      Setting age of taxon 'Cornovichthys' to Fixed(385.00)      Setting age of taxon 'Lasanius' to Fixed(428.20)      Setting age of taxon 'Birkenia' to Fixed(435.40)      Setting age of taxon 'Rhyncholepis' to Fixed(431.95)      Setting age of taxon 'Myxinikela' to Fixed(310.00)      Setting age of taxon 'Tethymyxine' to Fixed(95.00)      Setting age of taxon 'Myxineidus' to Fixed(310.00)      Setting age of taxon 'Gilpichthys' to Fixed(310.00)      Setting age of taxon 'Mesomyzon' to Fixed(125.00)      Setting age of taxon 'Yanliaomyzon_occisor' to Fixed(158.00)      Setting age of taxon 'Yanliaomyzon_igensdentes' to Fixed(163.00)      Setting age of taxon 'Priscomyzon' to Fixed(360.00)      Setting age of taxon 'Mayomyzon' to Fixed(310.00)      Setting age of taxon 'Hardistiella' to Fixed(320.00)      Setting age of taxon 'Pipiscius' to Fixed(310.00)      Setting Nodeagepr to Calibrated for partition 1      Setting Nodeagepr to Calibrated for partition 2      Setting Nodeagepr to Calibrated for partition 3      Setting Nodeagepr to Calibrated for partition 4      Setting Nodeagepr to Calibrated for partition 5      Successfully set prior model parameters to all      applicable data partitions       WARNING: There are 61 characters incompatible with the specified               coding bias. These characters will be excluded.      WARNING: There are 10 characters incompatible with the specified               coding bias. These characters will be excluded.      WARNING: There are 5 characters incompatible with the specified               coding bias. These characters will be excluded.      Setting Brlenspr to Clock:Fossilization for partition 1      Setting Brlenspr to Clock:Fossilization for partition 2      Setting Brlenspr to Clock:Fossilization for partition 3      Setting Brlenspr to Clock:Fossilization for partition 4      Setting Brlenspr to Clock:Fossilization for partition 5      Successfully set prior model parameters to all      applicable data partitions       WARNING: There are 61 characters incompatible with the specified               coding bias. These characters will be excluded.      WARNING: There are 10 characters incompatible with the specified               coding bias. These characters will be excluded.      WARNING: There are 5 characters incompatible with the specified               coding bias. These characters will be excluded.      Setting SampleStrat to Random for partition 1      Setting SampleStrat to Random for partition 2      Setting SampleStrat to Random for partition 3      Setting SampleStrat to Random for partition 4      Setting SampleStrat to Random for partition 5      Successfully set prior model parameters to all      applicable data partitions       WARNING: There are 61 characters incompatible with the specified               coding bias. These characters will be excluded.      WARNING: There are 10 characters incompatible with the specified               coding bias. These characters will be excluded.      WARNING: There are 5 characters incompatible with the specified               coding bias. These characters will be excluded.      Setting Sampleprob to 0.30000000 for partition 1      Setting Sampleprob to 0.30000000 for partition 2      Setting Sampleprob to 0.30000000 for partition 3      Setting Sampleprob to 0.30000000 for partition 4      Setting Sampleprob to 0.30000000 for partition 5      Successfully set prior model parameters to all      applicable data partitions       WARNING: There are 61 characters incompatible with the specified               coding bias. These characters will be excluded.      WARNING: There are 10 characters incompatible with the specified               coding bias. These characters will be excluded.      WARNING: There are 5 characters incompatible with the specified               coding bias. These characters will be excluded.      Setting Speciationpr to Exponential(100.00) for partition 1      Setting Speciationpr to Exponential(100.00) for partition 2      Setting Speciationpr to Exponential(100.00) for partition 3      Setting Speciationpr to Exponential(100.00) for partition 4      Setting Speciationpr to Exponential(100.00) for partition 5      Successfully set prior model parameters to all      applicable data partitions       WARNING: There are 61 characters incompatible with the specified               coding bias. These characters will be excluded.      WARNING: There are 10 characters incompatible with the specified               coding bias. These characters will be excluded.      WARNING: There are 5 characters incompatible with the specified               coding bias. These characters will be excluded.      Setting Extinctionpr to Beta(2.00,1.00) for partition 1      Setting Extinctionpr to Beta(2.00,1.00) for partition 2      Setting Extinctionpr to Beta(2.00,1.00) for partition 3      Setting Extinctionpr to Beta(2.00,1.00) for partition 4      Setting Extinctionpr to Beta(2.00,1.00) for partition 5      Successfully set prior model parameters to all      applicable data partitions       WARNING: There are 61 characters incompatible with the specified               coding bias. These characters will be excluded.      WARNING: There are 10 characters incompatible with the specified               coding bias. These characters will be excluded.      WARNING: There are 5 characters incompatible with the specified               coding bias. These characters will be excluded.      Setting Fossilizationpr to Beta(1.00,9.00) for partition 1      Setting Fossilizationpr to Beta(1.00,9.00) for partition 2      Setting Fossilizationpr to Beta(1.00,9.00) for partition 3      Setting Fossilizationpr to Beta(1.00,9.00) for partition 4      Setting Fossilizationpr to Beta(1.00,9.00) for partition 5      Successfully set prior model parameters to all      applicable data partitions       WARNING: There are 61 characters incompatible with the specified               coding bias. These characters will be excluded.      WARNING: There are 10 characters incompatible with the specified               coding bias. These characters will be excluded.      WARNING: There are 5 characters incompatible with the specified               coding bias. These characters will be excluded.      Setting Treeagepr to Offsetexponential(500.00,600.00)      Setting Treeagepr to Offsetexponential(500.00,600.00)      Setting Treeagepr to Offsetexponential(500.00,600.00)      Setting Treeagepr to Offsetexponential(500.00,600.00)      Setting Treeagepr to Offsetexponential(500.00,600.00)      Successfully set prior model parameters to all      applicable data partitions       WARNING: There are 61 characters incompatible with the specified               coding bias. These characters will be excluded.      WARNING: There are 10 characters incompatible with the specified               coding bias. These characters will be excluded.      WARNING: There are 5 characters incompatible with the specified               coding bias. These characters will be excluded.      Setting Topologypr to Constraints for partition 1      Setting Topologypr to Constraints for partition 2      Setting Topologypr to Constraints for partition 3      Setting Topologypr to Constraints for partition 4      Setting Topologypr to Constraints for partition 5      Successfully set prior model parameters to all      applicable data partitions       WARNING: There are 61 characters incompatible with the specified               coding bias. These characters will be excluded.      WARNING: There are 10 characters incompatible with the specified               coding bias. These characters will be excluded.      WARNING: There are 5 characters incompatible with the specified               coding bias. These characters will be excluded.      Setting number of generations to 30000000      Setting sample frequency to 400      Setting print frequency to 10000      Setting diagnosing frequency to 50000      Successfully set chain parameters      Setting heating parameter to 0.070000      Setting chain output file names to "run.te.run<i>.<p/t>"      Successfully set chain parameters      Running Markov chain      MCMC stamp = 6291437938      Seed = 1816318358      Swapseed = 1654745601      Model settings:          Settings for partition 1 --            Datatype  = Standard            Coding    = Variable            # States  = Variable, up to 24                        State frequencies are fixed to be equal            Rates     = Gamma                        The distribution is approximated using 4 categories.                        Shape parameter is exponentially                        distributed with parameter (1.00).          Settings for partition 2 --            Datatype  = Standard            Coding    = Variable            # States  = Variable, up to 24                        State frequencies are fixed to be equal            Rates     = Gamma                        The distribution is approximated using 4 categories.                        Shape parameter is exponentially                        distributed with parameter (1.00).          Settings for partition 3 --            Datatype  = Standard            Coding    = Variable            # States  = Variable, up to 24                        State frequencies are fixed to be equal            Rates     = Gamma                        The distribution is approximated using 4 categories.                        Shape parameter is exponentially                        distributed with parameter (1.00).          Settings for partition 4 --            Datatype  = DNA            Nucmodel  = 4by4            Nst       = 2                        Transition and transversion  rates, expressed                        as proportions of the rate sum, have a                        Beta(1.00,1.00) prior            Covarion  = No            # States  = 4                        State frequencies have a Dirichlet prior                        (1.00,1.00,1.00,1.00)            Rates     = Gamma                        The distribution is approximated using 4 categories.                        Shape parameter is exponentially                        distributed with parameter (1.00).          Settings for partition 5 --            Datatype  = DNA            Nucmodel  = 4by4            Nst       = 2                        Transition and transversion  rates, expressed                        as proportions of the rate sum, have a                        Beta(1.00,1.00) prior            Covarion  = No            # States  = 4                        State frequencies have a Dirichlet prior                        (1.00,1.00,1.00,1.00)            Rates     = Gamma                        The distribution is approximated using 4 categories.                        Shape parameter is exponentially                        distributed with parameter (1.00).       Active parameters:                               Partition(s)         Parameters          1  2  3  4  5         ---------------------------------         Tratio              .  .  .  1  2         Statefreq           3  4  5  6  7         Shape               8  8  8  9 10         Ratemultiplier     11 11 11 11 11         Topology           12 12 12 12 12         Brlens             13 13 13 13 13         Speciationrate     14 14 14 14 14         Extinctionrate     15 15 15 15 15         Fossilizationrate  16 16 16 16 16         ILNvar             17 18 19 20 20         ILNbranchrates     21 22 23 24 24         Clockrate          25 25 25 25 25         ---------------------------------          Parameters can be linked or unlinked across partitions using 'link' and 'unlink'          1 --  Parameter  = Tratio{4}               Type       = Transition and transversion rates               Prior      = Beta(1.00,1.00)               Partition  = 4          2 --  Parameter  = Tratio{5}               Type       = Transition and transversion rates               Prior      = Beta(1.00,1.00)               Partition  = 5          3 --  Parameter  = Alpha_symdir{1}               Type       = Symmetric diricihlet/beta distribution alpha_i parameter               Prior      = Symmetric dirichlet with all parameters fixed to infinity               Partition  = 1          4 --  Parameter  = Alpha_symdir{2}               Type       = Symmetric diricihlet/beta distribution alpha_i parameter               Prior      = Symmetric dirichlet with all parameters fixed to infinity               Partition  = 2          5 --  Parameter  = Alpha_symdir{3}               Type       = Symmetric diricihlet/beta distribution alpha_i parameter               Prior      = Symmetric dirichlet with all parameters fixed to infinity               Partition  = 3          6 --  Parameter  = Pi{4}               Type       = Stationary state frequencies               Prior      = Dirichlet               Partition  = 4          7 --  Parameter  = Pi{5}               Type       = Stationary state frequencies               Prior      = Dirichlet               Partition  = 5          8 --  Parameter  = Alpha{1,2,3}               Type       = Shape of scaled gamma distribution of site rates               Prior      = Exponential(1.00)               Partitions = 1, 2, and 3          9 --  Parameter  = Alpha{4}               Type       = Shape of scaled gamma distribution of site rates               Prior      = Exponential(1.00)               Partition  = 4         10 --  Parameter  = Alpha{5}               Type       = Shape of scaled gamma distribution of site rates               Prior      = Exponential(1.00)               Partition  = 5         11 --  Parameter  = Ratemultiplier{all}               Type       = Partition-specific rate multiplier               Prior      = Dirichlet(1.00,1.00,1.00,1.00,1.00)               Partitions = All         12 --  Parameter  = Tau{all}               Type       = Topology               Prior      = Prior on topology obeys the following constraints:                            -- Hard constraint "ingroup"               Partitions = All               Subparam.  = V{all}         13 --  Parameter  = V{all}               Type       = Branch lengths               Prior      = Clock:Fossilization                            Node depths are constrained by the following age constraints:                            -- The age of terminal "Euconodonta" is Fixed(535.50)                            -- The age of terminal "Jamoytius" is Fixed(435.40)                            -- The age of terminal "Euphanerops" is Fixed(370.00)                            -- The age of terminal "Achanarella" is Fixed(385.00)                            -- The age of terminal "Ciderius" is Fixed(432.00)                            -- The age of terminal "Cornovichthys" is Fixed(385.00)                            -- The age of terminal "Lasanius" is Fixed(428.20)                            -- The age of terminal "Birkenia" is Fixed(435.40)                            -- The age of terminal "Rhyncholepis" is Fixed(431.95)                            -- The age of terminal "Myxinikela" is Fixed(310.00)                            -- The age of terminal "Tethymyxine" is Fixed(95.00)                            -- The age of terminal "Myxineidus" is Fixed(310.00)                            -- The age of terminal "Gilpichthys" is Fixed(310.00)                            -- The age of terminal "Mesomyzon" is Fixed(125.00)                            -- The age of terminal "Yanliaomyzon_igensdentes" is Fixed(163.00)                            -- The age of terminal "Yanliaomyzon_occisor" is Fixed(158.00)                            -- The age of terminal "Priscomyzon" is Fixed(360.00)                            -- The age of terminal "Mayomyzon" is Fixed(310.00)                            -- The age of terminal "Hardistiella" is Fixed(320.00)                            -- The age of terminal "Pipiscius" is Fixed(310.00)                            -- Tree age has a Offsetexponential(500.00,600.00) distribution               Partitions = All               Subparams  = IlnBrlens{1}, IlnBrlens{2}, IlnBrlens{3} and IlnBrlens{4,5}         14 --  Parameter  = Net_speciation{all}               Type       = Speciation rate               Prior      = Exponential(100.00)               Partitions = All         15 --  Parameter  = Relative_extinction{all}               Type       = Extinction rate               Prior      = Beta(2.00,1.00)               Partitions = All         16 --  Parameter  = Relative_fossilization{all}               Type       = Fossilization rate               Prior      = Beta(1.00,9.00)               Partitions = All         17 --  Parameter  = ILNvar{1}               Type       = Variance of ILN model branch rates               Prior      = Exponential(1.00)               Partition  = 1               Subparam.  = IlnBrlens{1}         18 --  Parameter  = ILNvar{2}               Type       = Variance of ILN model branch rates               Prior      = Exponential(1.00)               Partition  = 2               Subparam.  = IlnBrlens{2}         19 --  Parameter  = ILNvar{3}               Type       = Variance of ILN model branch rates               Prior      = Exponential(1.00)               Partition  = 3               Subparam.  = IlnBrlens{3}         20 --  Parameter  = ILNvar{4,5}               Type       = Variance of ILN model branch rates               Prior      = Exponential(1.00)               Partitions = 4 and 5               Subparam.  = IlnBrlens{4,5}         21 --  Parameter  = IlnBrlens{1}               Type       = Branch lengths of ILN relaxed clock               Prior      = LogNormal (expectation = 1.0, variance = ILNvar{1})                Partition  = 1         22 --  Parameter  = IlnBrlens{2}               Type       = Branch lengths of ILN relaxed clock               Prior      = LogNormal (expectation = 1.0, variance = ILNvar{2})                Partition  = 2         23 --  Parameter  = IlnBrlens{3}               Type       = Branch lengths of ILN relaxed clock               Prior      = LogNormal (expectation = 1.0, variance = ILNvar{3})                Partition  = 3         24 --  Parameter  = IlnBrlens{4,5}               Type       = Branch lengths of ILN relaxed clock               Prior      = LogNormal (expectation = 1.0, variance = ILNvar{4,5})                Partitions = 4 and 5         25 --  Parameter  = Clockrate{all}               Type       = Base rate of clock               Prior      = Lognormal(-6.00,1.00)                            The clock rate varies according to an independent lognormal model               Partitions = All        Number of chains per MPI processor = 1       The MCMC sampler will use the following moves:         With prob.  Chain will use move            0.45 %   Dirichlet(Tratio{4})            0.45 %   Dirichlet(Tratio{5})            0.22 %   Dirichlet(Pi{4})            0.22 %   Slider(Pi{4})            0.22 %   Dirichlet(Pi{5})            0.22 %   Slider(Pi{5})            0.45 %   Multiplier(Alpha{1,2,3})            0.45 %   Multiplier(Alpha{4})            0.45 %   Multiplier(Alpha{5})            0.34 %   Dirichlet(Ratemultiplier{all})            0.34 %   Slider(Ratemultiplier{all})            4.49 %   ExtSPRClock(Tau{all},V{all})            8.99 %   NNIClock(Tau{all},V{all})            4.49 %   ParsSPRClock(Tau{all},V{all})            4.49 %   AddBranch(V{all})            4.49 %   DelBranch(V{all})           13.48 %   NodesliderClock(V{all})            1.35 %   TreeStretch(V{all})            1.35 %   Multiplier(Net_speciation{all})            1.35 %   Slider(Relative_extinction{all})            1.35 %   Slider(Relative_fossilization{all})            0.90 %   Multiplier(ILNvar{1})            0.90 %   Multiplier(ILNvar{2})            0.90 %   Multiplier(ILNvar{3})            0.90 %   Multiplier(ILNvar{4,5})           11.24 %   Multiplier(IlnBrlens{1})           11.24 %   Multiplier(IlnBrlens{2})           11.24 %   Multiplier(IlnBrlens{3})           11.24 %   Multiplier(IlnBrlens{4,5})            1.80 %   Multiplier(Clockrate{all})       Division 1 has 67 unique site patterns      Division 2 has 14 unique site patterns      Division 3 has 47 unique site patterns      Division 4 has 244 unique site patterns      Division 5 has 290 unique site patterns      Initializing conditional likelihoods      Using standard non-SSE likelihood calculator for division 1 (single-precision)      Using standard non-SSE likelihood calculator for division 2 (single-precision)      Using standard non-SSE likelihood calculator for division 3 (single-precision)      Using standard AVX likelihood calculator for division 4 (single-precision)      Using standard AVX likelihood calculator for division 5 (single-precision)       Initial log likelihoods and log prior probs for run 1:         Chain 1 -- -13011.842873 -- -9309.200117       There are 7 more chains on other processor(s)       Overwriting file "run.te.mcmc"      Overwriting file "run.te.run1.p"      Overwriting file "run.te.run1.t"      Overwriting file "run.te.run2.p"      Overwriting file "run.te.run2.t"       Using a relative burnin of 25.0 % for diagnostics       Chain results (30000000 generations requested):          0 -- [-13011.843] [...7 remote chains...]       10000 -- (-7682.346) [...7 remote chains...] -- 2:29:57      20000 -- (-7638.304) [...7 remote chains...] -- 2:29:54      30000 -- (-7623.934) [...7 remote chains...] -- 2:29:51      40000 -- (-7623.441) [...7 remote chains...] -- 2:29:48      50000 -- (-7615.233) [...7 remote chains...] -- 2:29:45       Average standard deviation of split frequencies: 0.079969       60000 -- (-7626.585) [...7 remote chains...] -- 2:29:42      70000 -- (-7624.625) [...7 remote chains...] -- 2:29:39      80000 -- (-7597.828) [...7 remote chains...] -- 2:29:36      90000 -- (-7607.496) [...7 remote chains...] -- 2:29:33      100000 -- (-7607.036) [...7 remote chains...] -- 2:29:30       Average standard deviation of split frequencies: 0.051715       110000 -- (-7593.247) [...7 remote chains...] -- 2:29:27      120000 -- (-7598.622) [...7 remote chains...] -- 2:29:24      130000 -- (-7606.982) [...7 remote chains...] -- 2:29:21      140000 -- (-7601.663) [...7 remote chains...] -- 2:29:18      150000 -- (-7607.740) [...7 remote chains...] -- 2:29:15       Average standard deviation of split frequencies: 0.053986       160000 -- (-7619.128) [...7 remote chains...] -- 2:29:12      170000 -- (-7588.377) [...7 remote chains...] -- 2:32:04      180000 -- (-7615.555) [...7 remote chains...] -- 2:29:06      190000 -- (-7598.637) [...7 remote chains...] -- 2:29:03      200000 -- (-7594.577) [...7 remote chains...] -- 2:31:29       Average standard deviation of split frequencies: 0.039421       210000 -- (-7593.987) [...7 remote chains...] -- 2:31:18      220000 -- (-7591.238) [...7 remote chains...] -- 2:31:09      230000 -- (-7606.585) [...7 remote chains...] -- 2:31:00      240000 -- (-7593.834) [...7 remote chains...] -- 2:30:52      250000 -- (-7596.497) [...7 remote chains...] -- 2:30:44       Average standard deviation of split frequencies: 0.045629       260000 -- (-7604.291) [...7 remote chains...] -- 2:30:36      270000 -- (-7584.434) [...7 remote chains...] -- 2:30:29      280000 -- (-7575.931) [...7 remote chains...] -- 2:30:22      290000 -- (-7599.795) [...7 remote chains...] -- 2:30:15      300000 -- (-7579.356) [...7 remote chains...] -- 2:30:09       Average standard deviation of split frequencies: 0.044689       310000 -- (-7585.729) [...7 remote chains...] -- 2:30:02      320000 -- (-7583.159) [...7 remote chains...] -- 2:29:56      330000 -- (-7592.774) [...7 remote chains...] -- 2:29:50      340000 -- (-7596.062) [...7 remote chains...] -- 2:29:45      350000 -- (-7586.496) [...7 remote chains...] -- 2:29:39       Average standard deviation of split frequencies: 0.040956       360000 -- (-7591.190) [...7 remote chains...] -- 2:29:34      370000 -- (-7587.641) [...7 remote chains...] -- 2:29:29      380000 -- (-7579.912) [...7 remote chains...] -- 2:29:23      390000 -- (-7587.083) [...7 remote chains...] -- 2:29:18      400000 -- (-7584.670) [...7 remote chains...] -- 2:29:14       Average standard deviation of split frequencies: 0.040156       410000 -- (-7572.795) [...7 remote chains...] -- 2:29:09      420000 -- (-7584.787) [...7 remote chains...] -- 2:29:04      430000 -- (-7573.922) [...7 remote chains...] -- 2:30:08      440000 -- (-7592.125) [...7 remote chains...] -- 2:30:02      450000 -- (-7597.964) [...7 remote chains...] -- 2:29:56       Average standard deviation of split frequencies: 0.037329       460000 -- (-7580.500) [...7 remote chains...] -- 2:29:50      470000 -- (-7584.371) [...7 remote chains...] -- 2:29:44      480000 -- (-7594.150) [...7 remote chains...] -- 2:29:39      490000 -- (-7589.335) [...7 remote chains...] -- 2:29:33      500000 -- (-7583.961) [...7 remote chains...] -- 2:29:28       Average standard deviation of split frequencies: 0.033278       510000 -- (-7576.738) [...7 remote chains...] -- 2:29:22      520000 -- (-7598.278) [...7 remote chains...] -- 2:29:17      530000 -- (-7593.268) [...7 remote chains...] -- 2:29:12      540000 -- (-7594.579) [...7 remote chains...] -- 2:29:07      550000 -- (-7578.447) [...7 remote chains...] -- 2:29:55       Average standard deviation of split frequencies: 0.030074       560000 -- (-7565.072) [...7 remote chains...] -- 2:29:49      570000 -- (-7577.534) [...7 remote chains...] -- 2:29:43      580000 -- (-7568.902) [...7 remote chains...] -- 2:29:38      590000 -- (-7557.450) [...7 remote chains...] -- 2:29:32      600000 -- (-7577.858) [...7 remote chains...] -- 2:29:27       Average standard deviation of split frequencies: 0.029362       610000 -- (-7581.121) [...7 remote chains...] -- 2:29:21      620000 -- (-7568.089) [...7 remote chains...] -- 2:29:16      630000 -- (-7584.466) [...7 remote chains...] -- 2:29:10      640000 -- (-7596.782) [...7 remote chains...] -- 2:29:05      650000 -- (-7574.356) [...7 remote chains...] -- 2:29:00       Average standard deviation of split frequencies: 0.029616       660000 -- (-7575.071) [...7 remote chains...] -- 2:28:55      670000 -- (-7574.136) [...7 remote chains...] -- 2:29:34      680000 -- (-7572.287) [...7 remote chains...] -- 2:29:28      690000 -- (-7580.073) [...7 remote chains...] -- 2:29:22      700000 -- (-7572.081) [...7 remote chains...] -- 2:29:17       Average standard deviation of split frequencies: 0.030042       710000 -- (-7570.861) [...7 remote chains...] -- 2:29:12      720000 -- (-7586.453) [...7 remote chains...] -- 2:29:06      730000 -- (-7579.258) [...7 remote chains...] -- 2:29:01      740000 -- (-7576.904) [...7 remote chains...] -- 2:28:56      750000 -- (-7581.948) [...7 remote chains...] -- 2:28:51       Average standard deviation of split frequencies: 0.030637       760000 -- (-7597.500) [...7 remote chains...] -- 2:28:45      770000 -- (-7580.356) [...7 remote chains...] -- 2:28:40      780000 -- (-7565.366) [...7 remote chains...] -- 2:28:35      790000 -- (-7569.265) [...7 remote chains...] -- 2:28:30      800000 -- (-7576.538) [...7 remote chains...] -- 2:29:02       Average standard deviation of split frequencies: 0.030885       810000 -- (-7576.079) [...7 remote chains...] -- 2:28:57      820000 -- (-7571.484) [...7 remote chains...] -- 2:28:51      830000 -- (-7570.255) [...7 remote chains...] -- 2:28:46      840000 -- (-7588.459) [...7 remote chains...] -- 2:28:41      850000 -- (-7557.271) [...7 remote chains...] -- 2:28:36       Average standard deviation of split frequencies: 0.028426       860000 -- (-7570.284) [...7 remote chains...] -- 2:28:31      870000 -- (-7568.598) [...7 remote chains...] -- 2:28:26      880000 -- (-7567.108) [...7 remote chains...] -- 2:28:21      890000 -- (-7561.476) [...7 remote chains...] -- 2:28:16      900000 -- (-7578.133) [...7 remote chains...] -- 2:28:11       Average standard deviation of split frequencies: 0.027290       910000 -- (-7570.662) [...7 remote chains...] -- 2:28:06      920000 -- (-7586.541) [...7 remote chains...] -- 2:28:02      930000 -- (-7577.022) [...7 remote chains...] -- 2:28:28      940000 -- (-7575.260) [...7 remote chains...] -- 2:28:23      950000 -- (-7570.648) [...7 remote chains...] -- 2:28:18       Average standard deviation of split frequencies: 0.030025       960000 -- (-7587.606) [...7 remote chains...] -- 2:28:13      970000 -- (-7579.150) [...7 remote chains...] -- 2:28:08      980000 -- (-7581.586) [...7 remote chains...] -- 2:28:03      990000 -- (-7563.706) [...7 remote chains...] -- 2:27:58      1000000 -- (-7556.246) [...7 remote chains...] -- 2:27:54       Average standard deviation of split frequencies: 0.033624       1010000 -- (-7577.219) [...7 remote chains...] -- 2:27:49      1020000 -- (-7558.326) [...7 remote chains...] -- 2:27:44      1030000 -- (-7575.483) [...7 remote chains...] -- 2:28:07      1040000 -- (-7581.695) [...7 remote chains...] -- 2:28:02      1050000 -- (-7583.035) [...7 remote chains...] -- 2:27:58       Average standard deviation of split frequencies: 0.035240       1060000 -- (-7578.076) [...7 remote chains...] -- 2:27:53      1070000 -- (-7582.485) [...7 remote chains...] -- 2:27:48      1080000 -- (-7577.937) [...7 remote chains...] -- 2:27:43      1090000 -- (-7603.767) [...7 remote chains...] -- 2:27:38      1100000 -- (-7580.680) [...7 remote chains...] -- 2:27:33       Average standard deviation of split frequencies: 0.037356       1110000 -- (-7575.373) [...7 remote chains...] -- 2:27:29      1120000 -- (-7581.613) [...7 remote chains...] -- 2:27:24      1130000 -- (-7580.953) [...7 remote chains...] -- 2:27:45      1140000 -- (-7580.782) [...7 remote chains...] -- 2:27:40      1150000 -- (-7594.640) [...7 remote chains...] -- 2:27:35       Average standard deviation of split frequencies: 0.036653       1160000 -- (-7591.776) [...7 remote chains...] -- 2:27:30      1170000 -- (-7609.007) [...7 remote chains...] -- 2:27:26      1180000 -- (-7579.642) [...7 remote chains...] -- 2:27:21      1190000 -- (-7583.749) [...7 remote chains...] -- 2:27:16      1200000 -- (-7573.564) [...7 remote chains...] -- 2:27:12       Average standard deviation of split frequencies: 0.036414       1210000 -- (-7550.216) [...7 remote chains...] -- 2:27:31      1220000 -- (-7568.243) [...7 remote chains...] -- 2:27:26      1230000 -- (-7575.843) [...7 remote chains...] -- 2:27:21      1240000 -- (-7585.983) [...7 remote chains...] -- 2:27:16      1250000 -- (-7615.398) [...7 remote chains...] -- 2:27:12       Average standard deviation of split frequencies: 0.038713       1260000 -- (-7597.850) [...7 remote chains...] -- 2:27:07      1270000 -- (-7569.231) [...7 remote chains...] -- 2:27:02      1280000 -- (-7582.742) [...7 remote chains...] -- 2:26:57      1290000 -- (-7559.062) [...7 remote chains...] -- 2:27:15      1300000 -- (-7556.087) [...7 remote chains...] -- 2:27:10       Average standard deviation of split frequencies: 0.039591       1310000 -- (-7547.940) [...7 remote chains...] -- 2:27:06      1320000 -- (-7548.266) [...7 remote chains...] -- 2:27:01      1330000 -- (-7564.406) [...7 remote chains...] -- 2:26:56      1340000 -- (-7558.461) [...7 remote chains...] -- 2:26:51      1350000 -- (-7549.903) [...7 remote chains...] -- 2:26:47       Average standard deviation of split frequencies: 0.039184       1360000 -- (-7557.271) [...7 remote chains...] -- 2:26:42      1370000 -- (-7567.564) [...7 remote chains...] -- 2:26:37      1380000 -- (-7571.802) [...7 remote chains...] -- 2:26:33      1390000 -- (-7573.978) [...7 remote chains...] -- 2:26:49      1400000 -- (-7550.223) [...7 remote chains...] -- 2:26:44       Average standard deviation of split frequencies: 0.038637       1410000 -- (-7561.645) [...7 remote chains...] -- 2:26:40      1420000 -- (-7554.642) [...7 remote chains...] -- 2:26:35      1430000 -- (-7548.320) [...7 remote chains...] -- 2:26:30      1440000 -- (-7554.085) [...7 remote chains...] -- 2:26:26      1450000 -- (-7538.092) [...7 remote chains...] -- 2:26:21       Average standard deviation of split frequencies: 0.037597       1460000 -- (-7545.751) [...7 remote chains...] -- 2:26:17      1470000 -- (-7523.384) [...7 remote chains...] -- 2:26:12      1480000 -- (-7524.093) [...7 remote chains...] -- 2:26:27      1490000 -- (-7544.225) [...7 remote chains...] -- 2:26:22      1500000 -- (-7551.288) [...7 remote chains...] -- 2:26:18       Average standard deviation of split frequencies: 0.038030       1510000 -- (-7546.430) [...7 remote chains...] -- 2:26:13      1520000 -- (-7549.055) [...7 remote chains...] -- 2:26:08      1530000 -- (-7556.570) [...7 remote chains...] -- 2:26:04      1540000 -- (-7559.462) [...7 remote chains...] -- 2:25:59      1550000 -- (-7543.214) [...7 remote chains...] -- 2:26:13       Average standard deviation of split frequencies: 0.038277       1560000 -- (-7542.230) [...7 remote chains...] -- 2:26:09      1570000 -- (-7566.541) [...7 remote chains...] -- 2:26:04      1580000 -- (-7554.027) [...7 remote chains...] -- 2:25:59      1590000 -- (-7566.120) [...7 remote chains...] -- 2:25:55      1600000 -- (-7544.798) [...7 remote chains...] -- 2:25:50       Average standard deviation of split frequencies: 0.037951       1610000 -- (-7557.765) [...7 remote chains...] -- 2:25:46      1620000 -- (-7566.404) [...7 remote chains...] -- 2:25:59      1630000 -- (-7548.351) [...7 remote chains...] -- 2:25:54      1640000 -- (-7559.103) [...7 remote chains...] -- 2:25:50      1650000 -- (-7553.310) [...7 remote chains...] -- 2:25:45       Average standard deviation of split frequencies: 0.037978       1660000 -- (-7563.085) [...7 remote chains...] -- 2:25:41      1670000 -- (-7562.511) [...7 remote chains...] -- 2:25:36      1680000 -- (-7557.823) [...7 remote chains...] -- 2:25:32      1690000 -- (-7571.353) [...7 remote chains...] -- 2:25:44      1700000 -- (-7555.163) [...7 remote chains...] -- 2:25:39       Average standard deviation of split frequencies: 0.038629       1710000 -- (-7567.559) [...7 remote chains...] -- 2:25:35      1720000 -- (-7561.407) [...7 remote chains...] -- 2:25:30      1730000 -- (-7575.399) [...7 remote chains...] -- 2:25:26      1740000 -- (-7566.615) [...7 remote chains...] -- 2:25:21      1750000 -- (-7573.951) [...7 remote chains...] -- 2:25:17       Average standard deviation of split frequencies: 0.038105       1760000 -- (-7553.498) [...7 remote chains...] -- 2:25:12      1770000 -- (-7559.143) [...7 remote chains...] -- 2:25:24      1780000 -- [-7545.255] [...7 remote chains...] -- 2:25:19      1790000 -- (-7572.678) [...7 remote chains...] -- 2:25:15      1800000 -- (-7567.839) [...7 remote chains...] -- 2:25:10       Average standard deviation of split frequencies: 0.037890       1810000 -- (-7567.638) [...7 remote chains...] -- 2:25:06      1820000 -- (-7555.733) [...7 remote chains...] -- 2:25:01      1830000 -- [-7548.363] [...7 remote chains...] -- 2:24:57      1840000 -- [-7558.114] [...7 remote chains...] -- 2:25:08      1850000 -- [-7565.676] [...7 remote chains...] -- 2:25:03       Average standard deviation of split frequencies: 0.038823       1860000 -- [-7568.561] [...7 remote chains...] -- 2:24:59      1870000 -- [-7551.001] [...7 remote chains...] -- 2:24:54      1880000 -- [-7544.124] [...7 remote chains...] -- 2:24:50      1890000 -- [-7544.979] [...7 remote chains...] -- 2:24:45      1900000 -- [-7554.826] [...7 remote chains...] -- 2:24:56       Average standard deviation of split frequencies: 0.038204       1910000 -- [-7548.039] [...7 remote chains...] -- 2:24:51      1920000 -- (-7555.070) [...7 remote chains...] -- 2:24:47      1930000 -- [-7555.962] [...7 remote chains...] -- 2:24:42      1940000 -- (-7555.226) [...7 remote chains...] -- 2:24:38      1950000 -- (-7562.736) [...7 remote chains...] -- 2:24:33       Average standard deviation of split frequencies: 0.037662       1960000 -- (-7576.212) [...7 remote chains...] -- 2:24:29      1970000 -- (-7574.276) [...7 remote chains...] -- 2:24:39      1980000 -- (-7543.461) [...7 remote chains...] -- 2:24:34      1990000 -- (-7562.929) [...7 remote chains...] -- 2:24:30      2000000 -- (-7563.095) [...7 remote chains...] -- 2:24:26       Average standard deviation of split frequencies: 0.035726       2010000 -- (-7590.881) [...7 remote chains...] -- 2:24:21      2020000 -- (-7561.093) [...7 remote chains...] -- 2:24:17      2030000 -- (-7569.617) [...7 remote chains...] -- 2:24:12      2040000 -- (-7560.598) [...7 remote chains...] -- 2:24:08      2050000 -- (-7552.090) [...7 remote chains...] -- 2:24:17       Average standard deviation of split frequencies: 0.035096       2060000 -- (-7578.873) [...7 remote chains...] -- 2:24:13      2070000 -- (-7560.159) [...7 remote chains...] -- 2:24:08      2080000 -- (-7561.789) [...7 remote chains...] -- 2:24:04      2090000 -- (-7587.867) [...7 remote chains...] -- 2:24:00      2100000 -- (-7570.457) [...7 remote chains...] -- 2:23:55       Average standard deviation of split frequencies: 0.033412       2110000 -- (-7565.467) [...7 remote chains...] -- 2:23:51      2120000 -- (-7562.281) [...7 remote chains...] -- 2:23:47      2130000 -- (-7559.889) [...7 remote chains...] -- 2:23:55      2140000 -- (-7564.601) [...7 remote chains...] -- 2:23:51      2150000 -- (-7545.600) [...7 remote chains...] -- 2:23:47       Average standard deviation of split frequencies: 0.032405       2160000 -- (-7558.435) [...7 remote chains...] -- 2:23:42      2170000 -- (-7556.480) [...7 remote chains...] -- 2:23:38      2180000 -- (-7557.421) [...7 remote chains...] -- 2:23:33      2190000 -- (-7552.492) [...7 remote chains...] -- 2:23:42      2200000 -- (-7558.908) [...7 remote chains...] -- 2:23:38       Average standard deviation of split frequencies: 0.033459       2210000 -- (-7559.491) [...7 remote chains...] -- 2:23:33      2220000 -- (-7547.709) [...7 remote chains...] -- 2:23:29      2230000 -- (-7578.473) [...7 remote chains...] -- 2:23:24      2240000 -- (-7579.228) [...7 remote chains...] -- 2:23:20      2250000 -- (-7573.696) [...7 remote chains...] -- 2:23:16       Average standard deviation of split frequencies: 0.033270       2260000 -- (-7549.040) [...7 remote chains...] -- 2:23:24      2270000 -- (-7558.065) [...7 remote chains...] -- 2:23:19      2280000 -- [-7540.903] [...7 remote chains...] -- 2:23:15      2290000 -- [-7548.280] [...7 remote chains...] -- 2:23:11      2300000 -- [-7545.737] [...7 remote chains...] -- 2:23:07       Average standard deviation of split frequencies: 0.033292       2310000 -- [-7561.205] [...7 remote chains...] -- 2:23:02      2320000 -- [-7551.831] [...7 remote chains...] -- 2:22:58      2330000 -- [-7545.883] [...7 remote chains...] -- 2:23:06      2340000 -- (-7543.545) [...7 remote chains...] -- 2:23:01      2350000 -- (-7549.660) [...7 remote chains...] -- 2:22:57       Average standard deviation of split frequencies: 0.033162       2360000 -- (-7541.949) [...7 remote chains...] -- 2:22:53      2370000 -- (-7562.032) [...7 remote chains...] -- 2:22:48      2380000 -- (-7547.882) [...7 remote chains...] -- 2:22:44      2390000 -- (-7534.648) [...7 remote chains...] -- 2:22:40      2400000 -- (-7558.916) [...7 remote chains...] -- 2:22:47       Average standard deviation of split frequencies: 0.032365       2410000 -- (-7556.860) [...7 remote chains...] -- 2:22:43      2420000 -- (-7535.572) [...7 remote chains...] -- 2:22:38      2430000 -- (-7548.043) [...7 remote chains...] -- 2:22:34      2440000 -- (-7551.927) [...7 remote chains...] -- 2:22:30      2450000 -- (-7541.013) [...7 remote chains...] -- 2:22:26       Average standard deviation of split frequencies: 0.031733       2460000 -- (-7551.113) [...7 remote chains...] -- 2:22:21      2470000 -- (-7543.819) [...7 remote chains...] -- 2:22:28      2480000 -- [-7551.371] [...7 remote chains...] -- 2:22:24      2490000 -- (-7541.924) [...7 remote chains...] -- 2:22:20      2500000 -- (-7548.889) [...7 remote chains...] -- 2:22:16       Average standard deviation of split frequencies: 0.031853       2510000 -- (-7557.469) [...7 remote chains...] -- 2:22:11      2520000 -- (-7574.578) [...7 remote chains...] -- 2:22:07      2530000 -- (-7555.991) [...7 remote chains...] -- 2:22:03      2540000 -- (-7579.079) [...7 remote chains...] -- 2:21:59      2550000 -- (-7571.454) [...7 remote chains...] -- 2:22:05       Average standard deviation of split frequencies: 0.031465       2560000 -- (-7556.157) [...7 remote chains...] -- 2:22:01      2570000 -- (-7560.325) [...7 remote chains...] -- 2:21:57      2580000 -- (-7549.446) [...7 remote chains...] -- 2:21:52      2590000 -- (-7553.202) [...7 remote chains...] -- 2:21:48      2600000 -- (-7562.511) [...7 remote chains...] -- 2:21:44       Average standard deviation of split frequencies: 0.030033       2610000 -- (-7575.541) [...7 remote chains...] -- 2:21:40      2620000 -- (-7563.425) [...7 remote chains...] -- 2:21:36      2630000 -- (-7559.571) [...7 remote chains...] -- 2:21:42      2640000 -- (-7549.500) [...7 remote chains...] -- 2:21:38      2650000 -- (-7557.074) [...7 remote chains...] -- 2:21:33       Average standard deviation of split frequencies: 0.029947       2660000 -- (-7530.982) [...7 remote chains...] -- 2:21:29      2670000 -- (-7559.272) [...7 remote chains...] -- 2:21:25      2680000 -- (-7550.741) [...7 remote chains...] -- 2:21:21      2690000 -- (-7564.926) [...7 remote chains...] -- 2:21:17      2700000 -- (-7566.726) [...7 remote chains...] -- 2:21:13       Average standard deviation of split frequencies: 0.029550       2710000 -- (-7557.519) [...7 remote chains...] -- 2:21:19      2720000 -- (-7596.478) [...7 remote chains...] -- 2:21:14      2730000 -- (-7558.102) [...7 remote chains...] -- 2:21:10      2740000 -- (-7558.547) [...7 remote chains...] -- 2:21:06      2750000 -- (-7545.213) [...7 remote chains...] -- 2:21:02       Average standard deviation of split frequencies: 0.028691       2760000 -- (-7551.942) [...7 remote chains...] -- 2:20:58      2770000 -- (-7553.672) [...7 remote chains...] -- 2:20:54      2780000 -- (-7543.819) [...7 remote chains...] -- 2:20:49      2790000 -- (-7550.083) [...7 remote chains...] -- 2:20:45      2800000 -- [-7542.009] [...7 remote chains...] -- 2:20:51       Average standard deviation of split frequencies: 0.028044       2810000 -- [-7546.040] [...7 remote chains...] -- 2:20:47      2820000 -- (-7559.436) [...7 remote chains...] -- 2:20:43      2830000 -- (-7547.544) [...7 remote chains...] -- 2:20:39      2840000 -- (-7560.337) [...7 remote chains...] -- 2:20:34      2850000 -- (-7574.002) [...7 remote chains...] -- 2:20:30       Average standard deviation of split frequencies: 0.027933       2860000 -- (-7561.357) [...7 remote chains...] -- 2:20:26      2870000 -- (-7567.481) [...7 remote chains...] -- 2:20:32      2880000 -- (-7554.812) [...7 remote chains...] -- 2:20:27      2890000 -- (-7549.346) [...7 remote chains...] -- 2:20:23      2900000 -- (-7547.478) [...7 remote chains...] -- 2:20:19       Average standard deviation of split frequencies: 0.028467       2910000 -- (-7553.047) [...7 remote chains...] -- 2:20:15      2920000 -- (-7588.848) [...7 remote chains...] -- 2:20:11      2930000 -- (-7565.018) [...7 remote chains...] -- 2:20:07      2940000 -- (-7584.334) [...7 remote chains...] -- 2:20:12      2950000 -- (-7559.753) [...7 remote chains...] -- 2:20:08       Average standard deviation of split frequencies: 0.027919       2960000 -- (-7583.868) [...7 remote chains...] -- 2:20:04      2970000 -- [-7588.144] [...7 remote chains...] -- 2:20:00      2980000 -- (-7546.721) [...7 remote chains...] -- 2:19:56      2990000 -- (-7535.421) [...7 remote chains...] -- 2:19:52      3000000 -- (-7584.871) [...7 remote chains...] -- 2:19:48       Average standard deviation of split frequencies: 0.026922       3010000 -- (-7564.290) [...7 remote chains...] -- 2:19:52      3020000 -- (-7571.367) [...7 remote chains...] -- 2:19:48      3030000 -- (-7554.623) [...7 remote chains...] -- 2:19:44      3040000 -- (-7534.373) [...7 remote chains...] -- 2:19:40      3050000 -- (-7568.372) [...7 remote chains...] -- 2:19:36       Average standard deviation of split frequencies: 0.026674       3060000 -- (-7551.468) [...7 remote chains...] -- 2:19:32      3070000 -- (-7542.524) [...7 remote chains...] -- 2:19:28      3080000 -- (-7559.975) [...7 remote chains...] -- 2:19:33      3090000 -- (-7552.894) [...7 remote chains...] -- 2:19:29      3100000 -- (-7554.804) [...7 remote chains...] -- 2:19:25       Average standard deviation of split frequencies: 0.026845       3110000 -- (-7548.130) [...7 remote chains...] -- 2:19:20      3120000 -- (-7551.249) [...7 remote chains...] -- 2:19:16      3130000 -- (-7552.063) [...7 remote chains...] -- 2:19:12      3140000 -- [-7543.057] [...7 remote chains...] -- 2:19:08      3150000 -- (-7556.409) [...7 remote chains...] -- 2:19:13       Average standard deviation of split frequencies: 0.026228       3160000 -- [-7541.711] [...7 remote chains...] -- 2:19:09      3170000 -- (-7548.362) [...7 remote chains...] -- 2:19:05      3180000 -- (-7559.004) [...7 remote chains...] -- 2:19:01      3190000 -- [-7562.944] [...7 remote chains...] -- 2:18:57      3200000 -- [-7546.305] [...7 remote chains...] -- 2:18:53       Average standard deviation of split frequencies: 0.026255       3210000 -- (-7559.313) [...7 remote chains...] -- 2:18:49      3220000 -- [-7539.821] [...7 remote chains...] -- 2:18:53      3230000 -- [-7549.028] [...7 remote chains...] -- 2:18:49      3240000 -- [-7560.017] [...7 remote chains...] -- 2:18:45      3250000 -- [-7559.044] [...7 remote chains...] -- 2:18:41       Average standard deviation of split frequencies: 0.026307       3260000 -- [-7534.311] [...7 remote chains...] -- 2:18:37      3270000 -- [-7554.989] [...7 remote chains...] -- 2:18:33      3280000 -- (-7562.658) [...7 remote chains...] -- 2:18:29      3290000 -- [-7546.242] [...7 remote chains...] -- 2:18:25      3300000 -- [-7537.363] [...7 remote chains...] -- 2:18:21       Average standard deviation of split frequencies: 0.026316       3310000 -- [-7540.341] [...7 remote chains...] -- 2:18:25      3320000 -- [-7537.785] [...7 remote chains...] -- 2:18:21      3330000 -- [-7548.572] [...7 remote chains...] -- 2:18:17      3340000 -- [-7538.758] [...7 remote chains...] -- 2:18:13      3350000 -- (-7573.216) [...7 remote chains...] -- 2:18:09       Average standard deviation of split frequencies: 0.025612       3360000 -- [-7547.176] [...7 remote chains...] -- 2:18:05      3370000 -- [-7551.554] [...7 remote chains...] -- 2:18:01      3380000 -- [-7548.602] [...7 remote chains...] -- 2:17:57      3390000 -- (-7547.504) [...7 remote chains...] -- 2:18:01      3400000 -- [-7539.520] [...7 remote chains...] -- 2:17:57       Average standard deviation of split frequencies: 0.025361       3410000 -- [-7537.402] [...7 remote chains...] -- 2:17:53      3420000 -- [-7551.549] [...7 remote chains...] -- 2:17:49      3430000 -- [-7547.510] [...7 remote chains...] -- 2:17:45      3440000 -- [-7550.028] [...7 remote chains...] -- 2:17:41      3450000 -- (-7561.132) [...7 remote chains...] -- 2:17:37       Average standard deviation of split frequencies: 0.025511       3460000 -- [-7544.204] [...7 remote chains...] -- 2:17:33      3470000 -- [-7562.901] [...7 remote chains...] -- 2:17:37      3480000 -- [-7554.904] [...7 remote chains...] -- 2:17:33      3490000 -- (-7545.596) [...7 remote chains...] -- 2:17:29      3500000 -- (-7561.994) [...7 remote chains...] -- 2:17:25       Average standard deviation of split frequencies: 0.025311       3510000 -- (-7551.669) [...7 remote chains...] -- 2:17:21      3520000 -- (-7551.261) [...7 remote chains...] -- 2:17:17      3530000 -- (-7555.002) [...7 remote chains...] -- 2:17:13      3540000 -- [-7541.744] [...7 remote chains...] -- 2:17:16      3550000 -- (-7542.904) [...7 remote chains...] -- 2:17:13       Average standard deviation of split frequencies: 0.024559       3560000 -- [-7527.569] [...7 remote chains...] -- 2:17:09      3570000 -- (-7543.157) [...7 remote chains...] -- 2:17:05      3580000 -- (-7550.986) [...7 remote chains...] -- 2:17:01      3590000 -- (-7545.078) [...7 remote chains...] -- 2:16:57      3600000 -- (-7542.629) [...7 remote chains...] -- 2:16:53       Average standard deviation of split frequencies: 0.024268       3610000 -- (-7550.424) [...7 remote chains...] -- 2:16:49      3620000 -- [-7541.868] [...7 remote chains...] -- 2:16:52      3630000 -- (-7536.929) [...7 remote chains...] -- 2:16:48      3640000 -- (-7548.105) [...7 remote chains...] -- 2:16:44      3650000 -- (-7538.798) [...7 remote chains...] -- 2:16:40       Average standard deviation of split frequencies: 0.024359       3660000 -- [-7549.361] [...7 remote chains...] -- 2:16:37      3670000 -- [-7548.675] [...7 remote chains...] -- 2:16:33      3680000 -- [-7560.589] [...7 remote chains...] -- 2:16:29      3690000 -- [-7572.545] [...7 remote chains...] -- 2:16:25      3700000 -- [-7537.951] [...7 remote chains...] -- 2:16:28       Average standard deviation of split frequencies: 0.023962       3710000 -- [-7535.470] [...7 remote chains...] -- 2:16:24      3720000 -- [-7539.359] [...7 remote chains...] -- 2:16:20      3730000 -- [-7556.341] [...7 remote chains...] -- 2:16:16      3740000 -- [-7538.842] [...7 remote chains...] -- 2:16:12      3750000 -- [-7556.599] [...7 remote chains...] -- 2:16:08       Average standard deviation of split frequencies: 0.023791       3760000 -- (-7535.863) [...7 remote chains...] -- 2:16:05      3770000 -- (-7548.824) [...7 remote chains...] -- 2:16:08      3780000 -- [-7555.231] [...7 remote chains...] -- 2:16:04      3790000 -- [-7531.654] [...7 remote chains...] -- 2:16:00      3800000 -- (-7563.329) [...7 remote chains...] -- 2:15:56       Average standard deviation of split frequencies: 0.023196       3810000 -- (-7555.781) [...7 remote chains...] -- 2:15:52      3820000 -- (-7572.164) [...7 remote chains...] -- 2:15:48      3830000 -- [-7557.395] [...7 remote chains...] -- 2:15:44      3840000 -- [-7557.533] [...7 remote chains...] -- 2:15:47      3850000 -- [-7551.035] [...7 remote chains...] -- 2:15:43       Average standard deviation of split frequencies: 0.022233       3860000 -- (-7553.043) [...7 remote chains...] -- 2:15:39      3870000 -- [-7547.225] [...7 remote chains...] -- 2:15:36      3880000 -- [-7518.414] [...7 remote chains...] -- 2:15:32      3890000 -- [-7546.713] [...7 remote chains...] -- 2:15:28      3900000 -- [-7543.327] [...7 remote chains...] -- 2:15:24       Average standard deviation of split frequencies: 0.021382       3910000 -- (-7540.464) [...7 remote chains...] -- 2:15:20      3920000 -- (-7555.645) [...7 remote chains...] -- 2:15:23      3930000 -- (-7557.534) [...7 remote chains...] -- 2:15:19      3940000 -- (-7557.979) [...7 remote chains...] -- 2:15:15      3950000 -- [-7552.302] [...7 remote chains...] -- 2:15:11       Average standard deviation of split frequencies: 0.021358       3960000 -- [-7543.867] [...7 remote chains...] -- 2:15:07      3970000 -- (-7552.863) [...7 remote chains...] -- 2:15:04      3980000 -- (-7526.870) [...7 remote chains...] -- 2:15:00      3990000 -- [-7542.524] [...7 remote chains...] -- 2:14:56      4000000 -- [-7547.934] [...7 remote chains...] -- 2:14:52       Average standard deviation of split frequencies: 0.021620       4010000 -- (-7542.426) [...7 remote chains...] -- 2:14:55      4020000 -- [-7558.814] [...7 remote chains...] -- 2:14:51      4030000 -- [-7542.789] [...7 remote chains...] -- 2:14:47      4040000 -- [-7552.673] [...7 remote chains...] -- 2:14:43      4050000 -- [-7555.782] [...7 remote chains...] -- 2:14:39       Average standard deviation of split frequencies: 0.021373       4060000 -- [-7551.587] [...7 remote chains...] -- 2:14:35      4070000 -- [-7546.577] [...7 remote chains...] -- 2:14:32      4080000 -- [-7555.994] [...7 remote chains...] -- 2:14:28      4090000 -- [-7540.025] [...7 remote chains...] -- 2:14:24      4100000 -- [-7565.078] [...7 remote chains...] -- 2:14:26       Average standard deviation of split frequencies: 0.021214       4110000 -- [-7567.581] [...7 remote chains...] -- 2:14:23      4120000 -- (-7562.926) [...7 remote chains...] -- 2:14:19      4130000 -- [-7560.670] [...7 remote chains...] -- 2:14:15      4140000 -- [-7560.133] [...7 remote chains...] -- 2:14:11      4150000 -- [-7540.987] [...7 remote chains...] -- 2:14:07       Average standard deviation of split frequencies: 0.021923       4160000 -- [-7544.867] [...7 remote chains...] -- 2:14:03      4170000 -- (-7545.381) [...7 remote chains...] -- 2:14:00      4180000 -- (-7542.038) [...7 remote chains...] -- 2:14:02      4190000 -- (-7563.494) [...7 remote chains...] -- 2:13:58      4200000 -- (-7560.822) [...7 remote chains...] -- 2:13:54       Average standard deviation of split frequencies: 0.022200       4210000 -- (-7548.277) [...7 remote chains...] -- 2:13:51      4220000 -- (-7542.315) [...7 remote chains...] -- 2:13:47      4230000 -- (-7548.629) [...7 remote chains...] -- 2:13:43      4240000 -- (-7578.341) [...7 remote chains...] -- 2:13:39      4250000 -- (-7564.380) [...7 remote chains...] -- 2:13:41       Average standard deviation of split frequencies: 0.021916       4260000 -- (-7555.775) [...7 remote chains...] -- 2:13:38      4270000 -- (-7548.831) [...7 remote chains...] -- 2:13:34      4280000 -- (-7560.289) [...7 remote chains...] -- 2:13:30      4290000 -- (-7566.184) [...7 remote chains...] -- 2:13:26      4300000 -- (-7563.017) [...7 remote chains...] -- 2:13:22       Average standard deviation of split frequencies: 0.021217       4310000 -- (-7570.342) [...7 remote chains...] -- 2:13:19      4320000 -- (-7553.666) [...7 remote chains...] -- 2:13:15      4330000 -- (-7562.623) [...7 remote chains...] -- 2:13:17      4340000 -- (-7581.782) [...7 remote chains...] -- 2:13:13      4350000 -- (-7574.619) [...7 remote chains...] -- 2:13:09       Average standard deviation of split frequencies: 0.021691       4360000 -- (-7568.994) [...7 remote chains...] -- 2:13:06      4370000 -- (-7569.016) [...7 remote chains...] -- 2:13:02      4380000 -- (-7546.411) [...7 remote chains...] -- 2:12:58      4390000 -- (-7568.951) [...7 remote chains...] -- 2:12:54      4400000 -- (-7562.704) [...7 remote chains...] -- 2:12:50       Average standard deviation of split frequencies: 0.021606       4410000 -- (-7569.867) [...7 remote chains...] -- 2:12:52      4420000 -- (-7573.702) [...7 remote chains...] -- 2:12:49      4430000 -- (-7575.504) [...7 remote chains...] -- 2:12:45      4440000 -- (-7547.521) [...7 remote chains...] -- 2:12:41      4450000 -- (-7570.984) [...7 remote chains...] -- 2:12:37       Average standard deviation of split frequencies: 0.021517       4460000 -- (-7549.307) [...7 remote chains...] -- 2:12:34      4470000 -- [-7533.321] [...7 remote chains...] -- 2:12:30      4480000 -- (-7558.048) [...7 remote chains...] -- 2:12:26      4490000 -- (-7552.717) [...7 remote chains...] -- 2:12:22      4500000 -- (-7536.008) [...7 remote chains...] -- 2:12:24       Average standard deviation of split frequencies: 0.020927       4510000 -- (-7571.558) [...7 remote chains...] -- 2:12:20      4520000 -- (-7555.488) [...7 remote chains...] -- 2:12:17      4530000 -- (-7552.719) [...7 remote chains...] -- 2:12:13      4540000 -- (-7562.541) [...7 remote chains...] -- 2:12:09      4550000 -- (-7579.853) [...7 remote chains...] -- 2:12:05       Average standard deviation of split frequencies: 0.020304       4560000 -- (-7560.259) [...7 remote chains...] -- 2:12:02      4570000 -- (-7580.334) [...7 remote chains...] -- 2:11:58      4580000 -- (-7542.833) [...7 remote chains...] -- 2:12:00      4590000 -- (-7568.747) [...7 remote chains...] -- 2:11:56      4600000 -- (-7564.775) [...7 remote chains...] -- 2:11:52       Average standard deviation of split frequencies: 0.020234       4610000 -- [-7553.158] [...7 remote chains...] -- 2:11:48      4620000 -- (-7566.154) [...7 remote chains...] -- 2:11:45      4630000 -- [-7539.399] [...7 remote chains...] -- 2:11:41      4640000 -- (-7550.191) [...7 remote chains...] -- 2:11:37      4650000 -- (-7545.660) [...7 remote chains...] -- 2:11:33       Average standard deviation of split frequencies: 0.019637       4660000 -- [-7557.231] [...7 remote chains...] -- 2:11:35      4670000 -- [-7551.024] [...7 remote chains...] -- 2:11:31      4680000 -- [-7550.225] [...7 remote chains...] -- 2:11:28      4690000 -- [-7554.705] [...7 remote chains...] -- 2:11:24      4700000 -- [-7562.664] [...7 remote chains...] -- 2:11:20       Average standard deviation of split frequencies: 0.019260       4710000 -- (-7557.795) [...7 remote chains...] -- 2:11:16      4720000 -- [-7544.798] [...7 remote chains...] -- 2:11:13      4730000 -- (-7569.330) [...7 remote chains...] -- 2:11:14      4740000 -- [-7545.130] [...7 remote chains...] -- 2:11:11      4750000 -- [-7552.791] [...7 remote chains...] -- 2:11:07       Average standard deviation of split frequencies: 0.019115       4760000 -- (-7555.220) [...7 remote chains...] -- 2:11:03      4770000 -- [-7537.579] [...7 remote chains...] -- 2:10:59      4780000 -- (-7537.199) [...7 remote chains...] -- 2:10:56      4790000 -- (-7556.510) [...7 remote chains...] -- 2:10:52      4800000 -- (-7548.787) [...7 remote chains...] -- 2:10:54       Average standard deviation of split frequencies: 0.018408       4810000 -- (-7545.226) [...7 remote chains...] -- 2:10:50      4820000 -- (-7559.156) [...7 remote chains...] -- 2:10:46      4830000 -- (-7569.322) [...7 remote chains...] -- 2:10:42      4840000 -- (-7542.410) [...7 remote chains...] -- 2:10:39      4850000 -- (-7557.489) [...7 remote chains...] -- 2:10:35       Average standard deviation of split frequencies: 0.017606       4860000 -- (-7548.742) [...7 remote chains...] -- 2:10:36      4870000 -- (-7574.667) [...7 remote chains...] -- 2:10:33      4880000 -- [-7575.741] [...7 remote chains...] -- 2:10:29      4890000 -- (-7561.494) [...7 remote chains...] -- 2:10:25      4900000 -- [-7543.431] [...7 remote chains...] -- 2:10:21       Average standard deviation of split frequencies: 0.017958       4910000 -- (-7568.634) [...7 remote chains...] -- 2:10:18      4920000 -- (-7560.714) [...7 remote chains...] -- 2:10:14      4930000 -- (-7556.930) [...7 remote chains...] -- 2:10:10      4940000 -- (-7537.840) [...7 remote chains...] -- 2:10:12      4950000 -- (-7554.234) [...7 remote chains...] -- 2:10:08       Average standard deviation of split frequencies: 0.018049       4960000 -- (-7568.115) [...7 remote chains...] -- 2:10:04      4970000 -- (-7551.860) [...7 remote chains...] -- 2:10:01      4980000 -- (-7556.207) [...7 remote chains...] -- 2:09:57      4990000 -- (-7551.775) [...7 remote chains...] -- 2:09:53      5000000 -- (-7539.649) [...7 remote chains...] -- 2:09:50       Average standard deviation of split frequencies: 0.017635       5010000 -- (-7576.705) [...7 remote chains...] -- 2:09:51      5020000 -- (-7544.061) [...7 remote chains...] -- 2:09:47      5030000 -- (-7564.168) [...7 remote chains...] -- 2:09:43      5040000 -- (-7557.660) [...7 remote chains...] -- 2:09:40      5050000 -- (-7550.783) [...7 remote chains...] -- 2:09:36       Average standard deviation of split frequencies: 0.016712       5060000 -- (-7536.989) [...7 remote chains...] -- 2:09:32      5070000 -- (-7539.797) [...7 remote chains...] -- 2:09:29      5080000 -- (-7555.738) [...7 remote chains...] -- 2:09:25      5090000 -- (-7567.505) [...7 remote chains...] -- 2:09:21      5100000 -- (-7560.804) [...7 remote chains...] -- 2:09:22       Average standard deviation of split frequencies: 0.015536       5110000 -- (-7557.750) [...7 remote chains...] -- 2:09:19      5120000 -- (-7566.121) [...7 remote chains...] -- 2:09:15      5130000 -- (-7566.567) [...7 remote chains...] -- 2:09:11      5140000 -- (-7566.924) [...7 remote chains...] -- 2:09:08      5150000 -- (-7556.698) [...7 remote chains...] -- 2:09:04       Average standard deviation of split frequencies: 0.015413       5160000 -- (-7580.774) [...7 remote chains...] -- 2:09:00      5170000 -- (-7555.939) [...7 remote chains...] -- 2:08:57      5180000 -- (-7537.679) [...7 remote chains...] -- 2:08:58      5190000 -- (-7552.281) [...7 remote chains...] -- 2:08:54      5200000 -- (-7558.560) [...7 remote chains...] -- 2:08:50       Average standard deviation of split frequencies: 0.015604       5210000 -- (-7551.118) [...7 remote chains...] -- 2:08:47      5220000 -- (-7572.885) [...7 remote chains...] -- 2:08:43      5230000 -- (-7545.557) [...7 remote chains...] -- 2:08:39      5240000 -- (-7555.611) [...7 remote chains...] -- 2:08:36      5250000 -- (-7544.041) [...7 remote chains...] -- 2:08:32       Average standard deviation of split frequencies: 0.015372       5260000 -- (-7537.178) [...7 remote chains...] -- 2:08:33      5270000 -- (-7570.821) [...7 remote chains...] -- 2:08:29      5280000 -- (-7575.862) [...7 remote chains...] -- 2:08:26      5290000 -- (-7567.298) [...7 remote chains...] -- 2:08:22      5300000 -- (-7551.093) [...7 remote chains...] -- 2:08:18       Average standard deviation of split frequencies: 0.015375       5310000 -- (-7569.975) [...7 remote chains...] -- 2:08:15      5320000 -- (-7547.897) [...7 remote chains...] -- 2:08:11      5330000 -- (-7548.890) [...7 remote chains...] -- 2:08:07      5340000 -- (-7559.188) [...7 remote chains...] -- 2:08:08      5350000 -- (-7561.883) [...7 remote chains...] -- 2:08:05       Average standard deviation of split frequencies: 0.015192       5360000 -- (-7544.911) [...7 remote chains...] -- 2:08:01      5370000 -- (-7556.959) [...7 remote chains...] -- 2:07:57      5380000 -- (-7557.558) [...7 remote chains...] -- 2:07:54      5390000 -- (-7542.709) [...7 remote chains...] -- 2:07:50      5400000 -- (-7562.404) [...7 remote chains...] -- 2:07:47       Average standard deviation of split frequencies: 0.015168       5410000 -- (-7567.926) [...7 remote chains...] -- 2:07:43      5420000 -- (-7569.398) [...7 remote chains...] -- 2:07:44      5430000 -- (-7569.388) [...7 remote chains...] -- 2:07:40      5440000 -- (-7566.049) [...7 remote chains...] -- 2:07:36      5450000 -- (-7540.033) [...7 remote chains...] -- 2:07:33       Average standard deviation of split frequencies: 0.015525       5460000 -- (-7546.818) [...7 remote chains...] -- 2:07:29      5470000 -- (-7560.286) [...7 remote chains...] -- 2:07:26      5480000 -- (-7542.517) [...7 remote chains...] -- 2:07:22      5490000 -- (-7569.710) [...7 remote chains...] -- 2:07:18      5500000 -- (-7548.872) [...7 remote chains...] -- 2:07:19       Average standard deviation of split frequencies: 0.015747       5510000 -- (-7563.645) [...7 remote chains...] -- 2:07:15      5520000 -- (-7563.026) [...7 remote chains...] -- 2:07:12      5530000 -- (-7567.032) [...7 remote chains...] -- 2:07:08      5540000 -- (-7560.570) [...7 remote chains...] -- 2:07:04      5550000 -- (-7572.624) [...7 remote chains...] -- 2:07:01       Average standard deviation of split frequencies: 0.015662       5560000 -- (-7565.570) [...7 remote chains...] -- 2:06:57      5570000 -- (-7567.778) [...7 remote chains...] -- 2:06:54      5580000 -- (-7551.557) [...7 remote chains...] -- 2:06:50      5590000 -- (-7568.306) [...7 remote chains...] -- 2:06:51      5600000 -- (-7558.962) [...7 remote chains...] -- 2:06:47       Average standard deviation of split frequencies: 0.015757       5610000 -- (-7564.460) [...7 remote chains...] -- 2:06:43      5620000 -- (-7561.374) [...7 remote chains...] -- 2:06:40      5630000 -- (-7534.502) [...7 remote chains...] -- 2:06:36      5640000 -- (-7542.071) [...7 remote chains...] -- 2:06:33      5650000 -- (-7543.196) [...7 remote chains...] -- 2:06:29       Average standard deviation of split frequencies: 0.014865       5660000 -- (-7546.062) [...7 remote chains...] -- 2:06:25      5670000 -- (-7559.894) [...7 remote chains...] -- 2:06:26      5680000 -- (-7574.359) [...7 remote chains...] -- 2:06:22      5690000 -- (-7557.944) [...7 remote chains...] -- 2:06:19      5700000 -- (-7552.389) [...7 remote chains...] -- 2:06:15       Average standard deviation of split frequencies: 0.014064       5710000 -- (-7566.957) [...7 remote chains...] -- 2:06:12      5720000 -- (-7565.117) [...7 remote chains...] -- 2:06:08      5730000 -- (-7571.278) [...7 remote chains...] -- 2:06:04      5740000 -- (-7549.750) [...7 remote chains...] -- 2:06:01      5750000 -- (-7546.204) [...7 remote chains...] -- 2:05:57       Average standard deviation of split frequencies: 0.014029       5760000 -- (-7566.718) [...7 remote chains...] -- 2:05:53      5770000 -- (-7547.597) [...7 remote chains...] -- 2:05:54      5780000 -- (-7568.732) [...7 remote chains...] -- 2:05:50      5790000 -- (-7553.962) [...7 remote chains...] -- 2:05:47      5800000 -- (-7577.875) [...7 remote chains...] -- 2:05:43       Average standard deviation of split frequencies: 0.013851       5810000 -- (-7568.823) [...7 remote chains...] -- 2:05:40      5820000 -- (-7562.280) [...7 remote chains...] -- 2:05:36      5830000 -- (-7567.453) [...7 remote chains...] -- 2:05:32      5840000 -- (-7551.455) [...7 remote chains...] -- 2:05:29      5850000 -- (-7541.332) [...7 remote chains...] -- 2:05:25       Average standard deviation of split frequencies: 0.013797       5860000 -- (-7583.204) [...7 remote chains...] -- 2:05:22      5870000 -- (-7587.676) [...7 remote chains...] -- 2:05:22      5880000 -- (-7575.812) [...7 remote chains...] -- 2:05:19      5890000 -- (-7565.347) [...7 remote chains...] -- 2:05:15      5900000 -- (-7555.032) [...7 remote chains...] -- 2:05:11       Average standard deviation of split frequencies: 0.014300       5910000 -- (-7564.224) [...7 remote chains...] -- 2:05:08      5920000 -- (-7546.481) [...7 remote chains...] -- 2:05:04      5930000 -- (-7548.778) [...7 remote chains...] -- 2:05:01      5940000 -- (-7560.804) [...7 remote chains...] -- 2:04:57      5950000 -- (-7556.750) [...7 remote chains...] -- 2:04:53       Average standard deviation of split frequencies: 0.014059       5960000 -- (-7557.893) [...7 remote chains...] -- 2:04:54      5970000 -- (-7563.936) [...7 remote chains...] -- 2:04:50      5980000 -- (-7562.687) [...7 remote chains...] -- 2:04:47      5990000 -- (-7557.000) [...7 remote chains...] -- 2:04:43      6000000 -- (-7564.014) [...7 remote chains...] -- 2:04:40       Average standard deviation of split frequencies: 0.013504       6010000 -- (-7573.472) [...7 remote chains...] -- 2:04:36      6020000 -- (-7575.027) [...7 remote chains...] -- 2:04:32      6030000 -- (-7566.932) [...7 remote chains...] -- 2:04:29      6040000 -- (-7557.512) [...7 remote chains...] -- 2:04:29      6050000 -- (-7540.683) [...7 remote chains...] -- 2:04:26       Average standard deviation of split frequencies: 0.013086       6060000 -- (-7568.359) [...7 remote chains...] -- 2:04:22      6070000 -- (-7561.881) [...7 remote chains...] -- 2:04:18      6080000 -- (-7561.319) [...7 remote chains...] -- 2:04:15      6090000 -- (-7572.510) [...7 remote chains...] -- 2:04:11      6100000 -- (-7577.740) [...7 remote chains...] -- 2:04:08       Average standard deviation of split frequencies: 0.013186       6110000 -- (-7564.943) [...7 remote chains...] -- 2:04:04      6120000 -- (-7569.603) [...7 remote chains...] -- 2:04:01      6130000 -- (-7568.126) [...7 remote chains...] -- 2:03:57      6140000 -- (-7561.579) [...7 remote chains...] -- 2:03:57      6150000 -- (-7555.689) [...7 remote chains...] -- 2:03:54       Average standard deviation of split frequencies: 0.013462       6160000 -- (-7548.512) [...7 remote chains...] -- 2:03:50      6170000 -- (-7525.615) [...7 remote chains...] -- 2:03:47      6180000 -- (-7546.953) [...7 remote chains...] -- 2:03:43      6190000 -- (-7549.535) [...7 remote chains...] -- 2:03:39      6200000 -- (-7565.751) [...7 remote chains...] -- 2:03:36       Average standard deviation of split frequencies: 0.013622       6210000 -- (-7553.274) [...7 remote chains...] -- 2:03:36      6220000 -- (-7553.683) [...7 remote chains...] -- 2:03:33      6230000 -- (-7528.081) [...7 remote chains...] -- 2:03:29      6240000 -- (-7568.396) [...7 remote chains...] -- 2:03:25      6250000 -- (-7578.308) [...7 remote chains...] -- 2:03:22       Average standard deviation of split frequencies: 0.013667       6260000 -- (-7556.488) [...7 remote chains...] -- 2:03:18      6270000 -- (-7557.528) [...7 remote chains...] -- 2:03:15      6280000 -- (-7550.384) [...7 remote chains...] -- 2:03:11      6290000 -- (-7562.735) [...7 remote chains...] -- 2:03:08      6300000 -- (-7564.178) [...7 remote chains...] -- 2:03:08       Average standard deviation of split frequencies: 0.013451       6310000 -- (-7550.667) [...7 remote chains...] -- 2:03:04      6320000 -- (-7532.969) [...7 remote chains...] -- 2:03:01      6330000 -- (-7539.951) [...7 remote chains...] -- 2:02:57      6340000 -- (-7550.178) [...7 remote chains...] -- 2:02:54      6350000 -- (-7552.468) [...7 remote chains...] -- 2:02:50       Average standard deviation of split frequencies: 0.012724       6360000 -- (-7547.897) [...7 remote chains...] -- 2:02:47      6370000 -- (-7568.217) [...7 remote chains...] -- 2:02:43      6380000 -- (-7542.911) [...7 remote chains...] -- 2:02:43      6390000 -- (-7550.916) [...7 remote chains...] -- 2:02:40      6400000 -- (-7560.614) [...7 remote chains...] -- 2:02:36       Average standard deviation of split frequencies: 0.012454       6410000 -- (-7551.287) [...7 remote chains...] -- 2:02:33      6420000 -- (-7550.521) [...7 remote chains...] -- 2:02:29      6430000 -- (-7597.223) [...7 remote chains...] -- 2:02:25      6440000 -- (-7562.818) [...7 remote chains...] -- 2:02:22      6450000 -- (-7556.592) [...7 remote chains...] -- 2:02:18       Average standard deviation of split frequencies: 0.012263       6460000 -- (-7557.169) [...7 remote chains...] -- 2:02:15      6470000 -- (-7581.583) [...7 remote chains...] -- 2:02:11      6480000 -- (-7588.071) [...7 remote chains...] -- 2:02:08      6490000 -- (-7579.284) [...7 remote chains...] -- 2:02:04      6500000 -- (-7569.922) [...7 remote chains...] -- 2:02:01       Average standard deviation of split frequencies: 0.012054       6510000 -- (-7564.310) [...7 remote chains...] -- 2:02:01      6520000 -- (-7578.285) [...7 remote chains...] -- 2:01:57      6530000 -- (-7567.609) [...7 remote chains...] -- 2:01:54      6540000 -- (-7543.848) [...7 remote chains...] -- 2:01:50      6550000 -- (-7555.160) [...7 remote chains...] -- 2:01:47       Average standard deviation of split frequencies: 0.012076       6560000 -- (-7570.148) [...7 remote chains...] -- 2:01:43      6570000 -- (-7550.725) [...7 remote chains...] -- 2:01:40      6580000 -- (-7551.153) [...7 remote chains...] -- 2:01:36      6590000 -- (-7564.358) [...7 remote chains...] -- 2:01:32      6600000 -- (-7546.851) [...7 remote chains...] -- 2:01:29       Average standard deviation of split frequencies: 0.012068       6610000 -- (-7558.720) [...7 remote chains...] -- 2:01:25      6620000 -- (-7548.585) [...7 remote chains...] -- 2:01:25      6630000 -- (-7592.483) [...7 remote chains...] -- 2:01:22      6640000 -- (-7573.619) [...7 remote chains...] -- 2:01:18      6650000 -- (-7558.909) [...7 remote chains...] -- 2:01:15       Average standard deviation of split frequencies: 0.012136       6660000 -- (-7565.731) [...7 remote chains...] -- 2:01:11      6670000 -- (-7553.912) [...7 remote chains...] -- 2:01:08      6680000 -- (-7568.331) [...7 remote chains...] -- 2:01:04      6690000 -- (-7568.409) [...7 remote chains...] -- 2:01:01      6700000 -- (-7597.587) [...7 remote chains...] -- 2:00:57       Average standard deviation of split frequencies: 0.012120       6710000 -- (-7581.679) [...7 remote chains...] -- 2:00:57      6720000 -- (-7567.434) [...7 remote chains...] -- 2:00:54      6730000 -- (-7536.566) [...7 remote chains...] -- 2:00:50      6740000 -- (-7562.787) [...7 remote chains...] -- 2:00:47      6750000 -- (-7550.122) [...7 remote chains...] -- 2:00:43       Average standard deviation of split frequencies: 0.012232       6760000 -- (-7565.932) [...7 remote chains...] -- 2:00:40      6770000 -- (-7563.732) [...7 remote chains...] -- 2:00:36      6780000 -- (-7576.951) [...7 remote chains...] -- 2:00:33      6790000 -- (-7556.331) [...7 remote chains...] -- 2:00:29      6800000 -- (-7537.973) [...7 remote chains...] -- 2:00:29       Average standard deviation of split frequencies: 0.012256       6810000 -- (-7542.644) [...7 remote chains...] -- 2:00:26      6820000 -- (-7548.850) [...7 remote chains...] -- 2:00:22      6830000 -- (-7548.454) [...7 remote chains...] -- 2:00:18      6840000 -- (-7593.667) [...7 remote chains...] -- 2:00:15      6850000 -- (-7563.998) [...7 remote chains...] -- 2:00:11       Average standard deviation of split frequencies: 0.012210       6860000 -- (-7545.396) [...7 remote chains...] -- 2:00:08      6870000 -- (-7565.573) [...7 remote chains...] -- 2:00:04      6880000 -- (-7579.184) [...7 remote chains...] -- 2:00:01      6890000 -- (-7554.692) [...7 remote chains...] -- 1:59:57      6900000 -- (-7559.634) [...7 remote chains...] -- 1:59:54       Average standard deviation of split frequencies: 0.012187       6910000 -- (-7556.317) [...7 remote chains...] -- 1:59:54      6920000 -- (-7571.224) [...7 remote chains...] -- 1:59:50      6930000 -- (-7535.551) [...7 remote chains...] -- 1:59:47      6940000 -- (-7543.709) [...7 remote chains...] -- 1:59:43      6950000 -- (-7546.770) [...7 remote chains...] -- 1:59:40       Average standard deviation of split frequencies: 0.012399       6960000 -- (-7575.061) [...7 remote chains...] -- 1:59:36      6970000 -- (-7548.780) [...7 remote chains...] -- 1:59:33      6980000 -- (-7572.563) [...7 remote chains...] -- 1:59:29      6990000 -- (-7556.670) [...7 remote chains...] -- 1:59:29      7000000 -- (-7552.235) [...7 remote chains...] -- 1:59:26       Average standard deviation of split frequencies: 0.012384       7010000 -- (-7564.735) [...7 remote chains...] -- 1:59:22      7020000 -- (-7543.029) [...7 remote chains...] -- 1:59:19      7030000 -- (-7555.429) [...7 remote chains...] -- 1:59:15      7040000 -- (-7557.633) [...7 remote chains...] -- 1:59:12      7050000 -- (-7539.498) [...7 remote chains...] -- 1:59:08       Average standard deviation of split frequencies: 0.012342       7060000 -- (-7573.945) [...7 remote chains...] -- 1:59:05      7070000 -- (-7570.944) [...7 remote chains...] -- 1:59:01      7080000 -- (-7550.756) [...7 remote chains...] -- 1:59:01      7090000 -- (-7567.441) [...7 remote chains...] -- 1:58:57      7100000 -- (-7561.296) [...7 remote chains...] -- 1:58:54       Average standard deviation of split frequencies: 0.012275       7110000 -- (-7553.437) [...7 remote chains...] -- 1:58:50      7120000 -- (-7564.873) [...7 remote chains...] -- 1:58:47      7130000 -- (-7570.782) [...7 remote chains...] -- 1:58:44      7140000 -- (-7554.137) [...7 remote chains...] -- 1:58:40      7150000 -- (-7547.874) [...7 remote chains...] -- 1:58:37       Average standard deviation of split frequencies: 0.012234       7160000 -- (-7563.914) [...7 remote chains...] -- 1:58:36      7170000 -- (-7564.358) [...7 remote chains...] -- 1:58:33      7180000 -- (-7604.306) [...7 remote chains...] -- 1:58:29      7190000 -- (-7587.107) [...7 remote chains...] -- 1:58:26      7200000 -- (-7571.000) [...7 remote chains...] -- 1:58:22       Average standard deviation of split frequencies: 0.012269       7210000 -- (-7556.579) [...7 remote chains...] -- 1:58:19      7220000 -- (-7558.544) [...7 remote chains...] -- 1:58:15      7230000 -- (-7556.962) [...7 remote chains...] -- 1:58:12      7240000 -- (-7555.010) [...7 remote chains...] -- 1:58:12      7250000 -- (-7553.055) [...7 remote chains...] -- 1:58:08       Average standard deviation of split frequencies: 0.012802       7260000 -- (-7558.313) [...7 remote chains...] -- 1:58:05      7270000 -- (-7562.713) [...7 remote chains...] -- 1:58:01      7280000 -- (-7564.481) [...7 remote chains...] -- 1:57:58      7290000 -- (-7548.620) [...7 remote chains...] -- 1:57:54      7300000 -- (-7552.352) [...7 remote chains...] -- 1:57:51       Average standard deviation of split frequencies: 0.012910       7310000 -- (-7557.087) [...7 remote chains...] -- 1:57:50      7320000 -- (-7559.212) [...7 remote chains...] -- 1:57:47      7330000 -- (-7554.315) [...7 remote chains...] -- 1:57:43      7340000 -- (-7539.783) [...7 remote chains...] -- 1:57:40      7350000 -- (-7548.953) [...7 remote chains...] -- 1:57:36       Average standard deviation of split frequencies: 0.012944       7360000 -- (-7541.016) [...7 remote chains...] -- 1:57:36      7370000 -- (-7555.553) [...7 remote chains...] -- 1:57:33      7380000 -- (-7552.656) [...7 remote chains...] -- 1:57:29      7390000 -- (-7566.250) [...7 remote chains...] -- 1:57:26      7400000 -- (-7572.197) [...7 remote chains...] -- 1:57:22       Average standard deviation of split frequencies: 0.012765       7410000 -- (-7570.349) [...7 remote chains...] -- 1:57:19      7420000 -- (-7566.035) [...7 remote chains...] -- 1:57:15      7430000 -- (-7563.496) [...7 remote chains...] -- 1:57:15      7440000 -- (-7538.574) [...7 remote chains...] -- 1:57:11      7450000 -- (-7538.599) [...7 remote chains...] -- 1:57:08       Average standard deviation of split frequencies: 0.012682       7460000 -- (-7549.414) [...7 remote chains...] -- 1:57:04      7470000 -- (-7552.090) [...7 remote chains...] -- 1:57:01      7480000 -- (-7556.018) [...7 remote chains...] -- 1:56:57      7490000 -- (-7559.241) [...7 remote chains...] -- 1:56:54      7500000 -- (-7574.873) [...7 remote chains...] -- 1:56:51       Average standard deviation of split frequencies: 0.012987       7510000 -- (-7565.188) [...7 remote chains...] -- 1:56:47      7520000 -- (-7545.444) [...7 remote chains...] -- 1:56:44      7530000 -- (-7563.849) [...7 remote chains...] -- 1:56:40      7540000 -- (-7553.539) [...7 remote chains...] -- 1:56:40      7550000 -- (-7559.447) [...7 remote chains...] -- 1:56:36       Average standard deviation of split frequencies: 0.013068       7560000 -- (-7555.779) [...7 remote chains...] -- 1:56:33      7570000 -- (-7558.490) [...7 remote chains...] -- 1:56:29      7580000 -- (-7542.039) [...7 remote chains...] -- 1:56:26      7590000 -- (-7545.505) [...7 remote chains...] -- 1:56:22      7600000 -- (-7561.054) [...7 remote chains...] -- 1:56:19       Average standard deviation of split frequencies: 0.013215       7610000 -- (-7547.472) [...7 remote chains...] -- 1:56:15      7620000 -- (-7545.841) [...7 remote chains...] -- 1:56:12      7630000 -- (-7562.029) [...7 remote chains...] -- 1:56:09      7640000 -- (-7545.477) [...7 remote chains...] -- 1:56:05      7650000 -- (-7566.521) [...7 remote chains...] -- 1:56:05       Average standard deviation of split frequencies: 0.013177       7660000 -- (-7567.947) [...7 remote chains...] -- 1:56:01      7670000 -- (-7564.426) [...7 remote chains...] -- 1:55:55      7680000 -- (-7555.955) [...7 remote chains...] -- 1:55:54      7690000 -- (-7555.638) [...7 remote chains...] -- 1:55:51      7700000 -- (-7555.400) [...7 remote chains...] -- 1:55:47       Average standard deviation of split frequencies: 0.013293       7710000 -- (-7557.820) [...7 remote chains...] -- 1:55:44      7720000 -- (-7552.362) [...7 remote chains...] -- 1:55:40      7730000 -- (-7558.421) [...7 remote chains...] -- 1:55:37      7740000 -- (-7558.680) [...7 remote chains...] -- 1:55:33      7750000 -- (-7578.676) [...7 remote chains...] -- 1:55:30       Average standard deviation of split frequencies: 0.013436       7760000 -- (-7571.015) [...7 remote chains...] -- 1:55:27      7770000 -- (-7542.469) [...7 remote chains...] -- 1:55:23      7780000 -- (-7591.973) [...7 remote chains...] -- 1:55:20      7790000 -- (-7561.727) [...7 remote chains...] -- 1:55:16      7800000 -- (-7563.049) [...7 remote chains...] -- 1:55:13       Average standard deviation of split frequencies: 0.013644       7810000 -- (-7569.598) [...7 remote chains...] -- 1:55:12      7820000 -- (-7552.417) [...7 remote chains...] -- 1:55:09      7830000 -- (-7565.301) [...7 remote chains...] -- 1:55:05      7840000 -- (-7554.794) [...7 remote chains...] -- 1:55:02      7850000 -- (-7562.874) [...7 remote chains...] -- 1:54:58       Average standard deviation of split frequencies: 0.013626       7860000 -- (-7564.176) [...7 remote chains...] -- 1:54:55      7870000 -- (-7578.359) [...7 remote chains...] -- 1:54:52      7880000 -- (-7549.902) [...7 remote chains...] -- 1:54:48      7890000 -- (-7562.261) [...7 remote chains...] -- 1:54:45      7900000 -- (-7567.826) [...7 remote chains...] -- 1:54:41       Average standard deviation of split frequencies: 0.013664       7910000 -- (-7565.165) [...7 remote chains...] -- 1:54:41      7920000 -- (-7568.144) [...7 remote chains...] -- 1:54:37      7930000 -- (-7567.465) [...7 remote chains...] -- 1:54:34      7940000 -- (-7548.165) [...7 remote chains...] -- 1:54:30      7950000 -- (-7560.033) [...7 remote chains...] -- 1:54:27       Average standard deviation of split frequencies: 0.013674       7960000 -- (-7557.415) [...7 remote chains...] -- 1:54:23      7970000 -- (-7561.646) [...7 remote chains...] -- 1:54:20      7980000 -- (-7543.544) [...7 remote chains...] -- 1:54:17      7990000 -- (-7556.845) [...7 remote chains...] -- 1:54:13      8000000 -- (-7558.992) [...7 remote chains...] -- 1:54:10       Average standard deviation of split frequencies: 0.013408       8010000 -- (-7547.395) [...7 remote chains...] -- 1:54:06      8020000 -- (-7553.632) [...7 remote chains...] -- 1:54:03      8030000 -- (-7547.300) [...7 remote chains...] -- 1:53:59      8040000 -- (-7560.509) [...7 remote chains...] -- 1:53:59      8050000 -- (-7557.600) [...7 remote chains...] -- 1:53:55       Average standard deviation of split frequencies: 0.013090       8060000 -- (-7561.814) [...7 remote chains...] -- 1:53:52      8070000 -- (-7555.367) [...7 remote chains...] -- 1:53:49      8080000 -- (-7580.536) [...7 remote chains...] -- 1:53:45      8090000 -- (-7570.886) [...7 remote chains...] -- 1:53:42      8100000 -- (-7551.227) [...7 remote chains...] -- 1:53:38       Average standard deviation of split frequencies: 0.012753       8110000 -- (-7563.779) [...7 remote chains...] -- 1:53:35      8120000 -- (-7548.616) [...7 remote chains...] -- 1:53:31      8130000 -- (-7551.039) [...7 remote chains...] -- 1:53:31      8140000 -- (-7572.274) [...7 remote chains...] -- 1:53:27      8150000 -- (-7559.191) [...7 remote chains...] -- 1:53:24       Average standard deviation of split frequencies: 0.012703       8160000 -- (-7566.955) [...7 remote chains...] -- 1:53:20      8170000 -- (-7566.397) [...7 remote chains...] -- 1:53:17      8180000 -- (-7543.072) [...7 remote chains...] -- 1:53:14      8190000 -- (-7554.644) [...7 remote chains...] -- 1:53:10      8200000 -- (-7548.971) [...7 remote chains...] -- 1:53:07       Average standard deviation of split frequencies: 0.012879       8210000 -- (-7560.968) [...7 remote chains...] -- 1:53:03      8220000 -- (-7553.937) [...7 remote chains...] -- 1:53:00      8230000 -- (-7564.123) [...7 remote chains...] -- 1:52:59      8240000 -- (-7550.395) [...7 remote chains...] -- 1:52:56      8250000 -- (-7542.408) [...7 remote chains...] -- 1:52:52       Average standard deviation of split frequencies: 0.013044       8260000 -- (-7562.952) [...7 remote chains...] -- 1:52:49      8270000 -- (-7577.770) [...7 remote chains...] -- 1:52:45      8280000 -- (-7544.079) [...7 remote chains...] -- 1:52:42      8290000 -- (-7565.282) [...7 remote chains...] -- 1:52:39      8300000 -- (-7545.516) [...7 remote chains...] -- 1:52:35       Average standard deviation of split frequencies: 0.012989       8310000 -- (-7549.435) [...7 remote chains...] -- 1:52:32      8320000 -- (-7580.602) [...7 remote chains...] -- 1:52:28      8330000 -- (-7560.959) [...7 remote chains...] -- 1:52:25      8340000 -- (-7562.261) [...7 remote chains...] -- 1:52:24      8350000 -- (-7565.845) [...7 remote chains...] -- 1:52:21       Average standard deviation of split frequencies: 0.013054       8360000 -- (-7546.374) [...7 remote chains...] -- 1:52:17      8370000 -- (-7562.948) [...7 remote chains...] -- 1:52:14      8380000 -- (-7551.065) [...7 remote chains...] -- 1:52:11      8390000 -- (-7545.688) [...7 remote chains...] -- 1:52:07      8400000 -- (-7578.249) [...7 remote chains...] -- 1:52:04       Average standard deviation of split frequencies: 0.012882       8410000 -- (-7560.778) [...7 remote chains...] -- 1:52:00      8420000 -- (-7551.800) [...7 remote chains...] -- 1:51:57      8430000 -- (-7553.353) [...7 remote chains...] -- 1:51:54      8440000 -- (-7546.596) [...7 remote chains...] -- 1:51:53      8450000 -- (-7565.585) [...7 remote chains...] -- 1:51:49       Average standard deviation of split frequencies: 0.012750       8460000 -- (-7560.712) [...7 remote chains...] -- 1:51:46      8470000 -- (-7563.192) [...7 remote chains...] -- 1:51:43      8480000 -- (-7556.295) [...7 remote chains...] -- 1:51:39      8490000 -- (-7554.325) [...7 remote chains...] -- 1:51:36      8500000 -- (-7568.899) [...7 remote chains...] -- 1:51:32       Average standard deviation of split frequencies: 0.012893       8510000 -- (-7551.509) [...7 remote chains...] -- 1:51:29      8520000 -- (-7555.571) [...7 remote chains...] -- 1:51:26      8530000 -- [-7554.112] [...7 remote chains...] -- 1:51:22      8540000 -- (-7562.520) [...7 remote chains...] -- 1:51:19      8550000 -- (-7561.846) [...7 remote chains...] -- 1:51:18       Average standard deviation of split frequencies: 0.012701       8560000 -- (-7552.324) [...7 remote chains...] -- 1:51:14      8570000 -- (-7579.259) [...7 remote chains...] -- 1:51:11      8580000 -- (-7560.971) [...7 remote chains...] -- 1:51:08      8590000 -- (-7564.291) [...7 remote chains...] -- 1:51:04      8600000 -- (-7561.916) [...7 remote chains...] -- 1:51:01       Average standard deviation of split frequencies: 0.012598       8610000 -- (-7562.628) [...7 remote chains...] -- 1:50:57      8620000 -- (-7573.803) [...7 remote chains...] -- 1:50:54      8630000 -- (-7564.283) [...7 remote chains...] -- 1:50:51      8640000 -- (-7563.820) [...7 remote chains...] -- 1:50:47      8650000 -- (-7570.098) [...7 remote chains...] -- 1:50:44       Average standard deviation of split frequencies: 0.012220       8660000 -- (-7576.905) [...7 remote chains...] -- 1:50:43      8670000 -- (-7591.851) [...7 remote chains...] -- 1:50:40      8680000 -- (-7566.567) [...7 remote chains...] -- 1:50:36      8690000 -- (-7569.320) [...7 remote chains...] -- 1:50:33      8700000 -- (-7555.271) [...7 remote chains...] -- 1:50:29       Average standard deviation of split frequencies: 0.012062       8710000 -- (-7582.346) [...7 remote chains...] -- 1:50:26      8720000 -- (-7540.145) [...7 remote chains...] -- 1:50:23      8730000 -- (-7563.085) [...7 remote chains...] -- 1:50:19      8740000 -- (-7560.078) [...7 remote chains...] -- 1:50:16      8750000 -- (-7568.082) [...7 remote chains...] -- 1:50:12       Average standard deviation of split frequencies: 0.011956       8760000 -- (-7551.019) [...7 remote chains...] -- 1:50:12      8770000 -- (-7552.124) [...7 remote chains...] -- 1:50:08      8780000 -- [-7541.090] [...7 remote chains...] -- 1:50:05      8790000 -- (-7543.859) [...7 remote chains...] -- 1:50:01      8800000 -- [-7544.613] [...7 remote chains...] -- 1:49:58       Average standard deviation of split frequencies: 0.011768       8810000 -- [-7557.403] [...7 remote chains...] -- 1:49:55      8820000 -- [-7554.360] [...7 remote chains...] -- 1:49:51      8830000 -- [-7553.489] [...7 remote chains...] -- 1:49:48      8840000 -- [-7525.457] [...7 remote chains...] -- 1:49:47      8850000 -- [-7543.305] [...7 remote chains...] -- 1:49:43       Average standard deviation of split frequencies: 0.011700       8860000 -- [-7554.062] [...7 remote chains...] -- 1:49:40      8870000 -- [-7566.305] [...7 remote chains...] -- 1:49:37      8880000 -- (-7567.162) [...7 remote chains...] -- 1:49:33      8890000 -- [-7534.418] [...7 remote chains...] -- 1:49:30      8900000 -- [-7559.271] [...7 remote chains...] -- 1:49:27       Average standard deviation of split frequencies: 0.011795       8910000 -- [-7555.459] [...7 remote chains...] -- 1:49:23      8920000 -- (-7541.681) [...7 remote chains...] -- 1:49:20      8930000 -- [-7537.918] [...7 remote chains...] -- 1:49:19      8940000 -- [-7546.166] [...7 remote chains...] -- 1:49:15      8950000 -- (-7559.184) [...7 remote chains...] -- 1:49:12       Average standard deviation of split frequencies: 0.011721       8960000 -- [-7552.959] [...7 remote chains...] -- 1:49:09      8970000 -- (-7554.683) [...7 remote chains...] -- 1:49:05      8980000 -- (-7545.831) [...7 remote chains...] -- 1:49:02      8990000 -- (-7554.460) [...7 remote chains...] -- 1:48:59      9000000 -- (-7565.983) [...7 remote chains...] -- 1:48:55       Average standard deviation of split frequencies: 0.011752       9010000 -- (-7561.666) [...7 remote chains...] -- 1:48:52      9020000 -- [-7562.437] [...7 remote chains...] -- 1:48:48      9030000 -- (-7572.425) [...7 remote chains...] -- 1:48:45      9040000 -- (-7561.407) [...7 remote chains...] -- 1:48:42      9050000 -- (-7566.744) [...7 remote chains...] -- 1:48:38       Average standard deviation of split frequencies: 0.011544       9060000 -- (-7547.908) [...7 remote chains...] -- 1:48:37      9070000 -- (-7546.295) [...7 remote chains...] -- 1:48:34      9080000 -- (-7547.468) [...7 remote chains...] -- 1:48:31      9090000 -- (-7543.249) [...7 remote chains...] -- 1:48:27      9100000 -- (-7552.782) [...7 remote chains...] -- 1:48:24       Average standard deviation of split frequencies: 0.011667       9110000 -- [-7558.572] [...7 remote chains...] -- 1:48:20      9120000 -- [-7538.332] [...7 remote chains...] -- 1:48:17      9130000 -- (-7536.427) [...7 remote chains...] -- 1:48:14      9140000 -- [-7536.829] [...7 remote chains...] -- 1:48:10      9150000 -- (-7543.581) [...7 remote chains...] -- 1:48:07       Average standard deviation of split frequencies: 0.011720       9160000 -- (-7538.244) [...7 remote chains...] -- 1:48:04      9170000 -- (-7548.932) [...7 remote chains...] -- 1:48:00      9180000 -- (-7562.677) [...7 remote chains...] -- 1:47:57      9190000 -- (-7565.041) [...7 remote chains...] -- 1:47:56      9200000 -- (-7552.771) [...7 remote chains...] -- 1:47:52       Average standard deviation of split frequencies: 0.011719       9210000 -- (-7553.960) [...7 remote chains...] -- 1:47:49      9220000 -- (-7548.692) [...7 remote chains...] -- 1:47:46      9230000 -- (-7567.027) [...7 remote chains...] -- 1:47:42      9240000 -- (-7571.682) [...7 remote chains...] -- 1:47:39      9250000 -- (-7575.211) [...7 remote chains...] -- 1:47:36       Average standard deviation of split frequencies: 0.011826       9260000 -- (-7556.441) [...7 remote chains...] -- 1:47:32      9270000 -- (-7567.369) [...7 remote chains...] -- 1:47:29      9280000 -- (-7581.146) [...7 remote chains...] -- 1:47:25      9290000 -- (-7567.957) [...7 remote chains...] -- 1:47:24      9300000 -- (-7566.923) [...7 remote chains...] -- 1:47:21       Average standard deviation of split frequencies: 0.011847       9310000 -- (-7560.654) [...7 remote chains...] -- 1:47:18      9320000 -- (-7558.970) [...7 remote chains...] -- 1:47:14      9330000 -- (-7577.595) [...7 remote chains...] -- 1:47:11      9340000 -- (-7553.541) [...7 remote chains...] -- 1:47:08      9350000 -- [-7546.746] [...7 remote chains...] -- 1:47:04       Average standard deviation of split frequencies: 0.011953       9360000 -- (-7563.473) [...7 remote chains...] -- 1:47:03      9370000 -- (-7566.914) [...7 remote chains...] -- 1:47:00      9380000 -- (-7572.112) [...7 remote chains...] -- 1:46:56      9390000 -- (-7537.808) [...7 remote chains...] -- 1:46:53      9400000 -- (-7554.111) [...7 remote chains...] -- 1:46:50       Average standard deviation of split frequencies: 0.011999       9410000 -- (-7544.737) [...7 remote chains...] -- 1:46:46      9420000 -- (-7579.417) [...7 remote chains...] -- 1:46:43      9430000 -- (-7559.504) [...7 remote chains...] -- 1:46:40      9440000 -- (-7578.847) [...7 remote chains...] -- 1:46:38      9450000 -- (-7564.458) [...7 remote chains...] -- 1:46:35       Average standard deviation of split frequencies: 0.011843       9460000 -- (-7582.257) [...7 remote chains...] -- 1:46:32      9470000 -- (-7542.130) [...7 remote chains...] -- 1:46:28      9480000 -- (-7559.974) [...7 remote chains...] -- 1:46:25      9490000 -- (-7546.997) [...7 remote chains...] -- 1:46:22      9500000 -- (-7572.359) [...7 remote chains...] -- 1:46:18       Average standard deviation of split frequencies: 0.011803       9510000 -- (-7543.642) [...7 remote chains...] -- 1:46:15      9520000 -- (-7533.592) [...7 remote chains...] -- 1:46:12      9530000 -- [-7545.199] [...7 remote chains...] -- 1:46:08      9540000 -- (-7556.185) [...7 remote chains...] -- 1:46:07      9550000 -- [-7555.955] [...7 remote chains...] -- 1:46:04       Average standard deviation of split frequencies: 0.011850       9560000 -- [-7547.127] [...7 remote chains...] -- 1:46:00      9570000 -- [-7561.144] [...7 remote chains...] -- 1:45:57      9580000 -- [-7546.864] [...7 remote chains...] -- 1:45:54      9590000 -- [-7550.485] [...7 remote chains...] -- 1:45:50      9600000 -- (-7557.874) [...7 remote chains...] -- 1:45:47       Average standard deviation of split frequencies: 0.011973       9610000 -- (-7555.930) [...7 remote chains...] -- 1:45:44      9620000 -- (-7564.893) [...7 remote chains...] -- 1:45:40      9630000 -- (-7563.492) [...7 remote chains...] -- 1:45:37      9640000 -- (-7556.253) [...7 remote chains...] -- 1:45:36      9650000 -- (-7551.522) [...7 remote chains...] -- 1:45:32       Average standard deviation of split frequencies: 0.012049       9660000 -- (-7553.975) [...7 remote chains...] -- 1:45:29      9670000 -- (-7540.995) [...7 remote chains...] -- 1:45:26      9680000 -- [-7543.867] [...7 remote chains...] -- 1:45:22      9690000 -- [-7550.338] [...7 remote chains...] -- 1:45:19      9700000 -- [-7553.448] [...7 remote chains...] -- 1:45:16       Average standard deviation of split frequencies: 0.012041       9710000 -- (-7551.969) [...7 remote chains...] -- 1:45:12      9720000 -- [-7559.988] [...7 remote chains...] -- 1:45:09      9730000 -- [-7554.253] [...7 remote chains...] -- 1:45:05      9740000 -- [-7534.027] [...7 remote chains...] -- 1:45:04      9750000 -- (-7565.155) [...7 remote chains...] -- 1:45:01       Average standard deviation of split frequencies: 0.012018       9760000 -- (-7564.355) [...7 remote chains...] -- 1:44:58      9770000 -- (-7555.364) [...7 remote chains...] -- 1:44:54      9780000 -- (-7556.081) [...7 remote chains...] -- 1:44:51      9790000 -- (-7556.160) [...7 remote chains...] -- 1:44:48      9800000 -- (-7553.634) [...7 remote chains...] -- 1:44:44       Average standard deviation of split frequencies: 0.011986       9810000 -- [-7542.613] [...7 remote chains...] -- 1:44:41      9820000 -- (-7557.866) [...7 remote chains...] -- 1:44:37      9830000 -- (-7551.011) [...7 remote chains...] -- 1:44:34      9840000 -- (-7568.618) [...7 remote chains...] -- 1:44:31      9850000 -- (-7568.747) [...7 remote chains...] -- 1:44:27       Average standard deviation of split frequencies: 0.011891       9860000 -- (-7562.219) [...7 remote chains...] -- 1:44:24      9870000 -- (-7553.321) [...7 remote chains...] -- 1:44:23      9880000 -- (-7573.680) [...7 remote chains...] -- 1:44:20      9890000 -- (-7544.248) [...7 remote chains...] -- 1:44:16      9900000 -- (-7567.653) [...7 remote chains...] -- 1:44:13       Average standard deviation of split frequencies: 0.011834       9910000 -- (-7561.487) [...7 remote chains...] -- 1:44:09      9920000 -- (-7565.256) [...7 remote chains...] -- 1:44:06      9930000 -- (-7565.499) [...7 remote chains...] -- 1:44:03      9940000 -- (-7557.492) [...7 remote chains...] -- 1:43:59      9950000 -- (-7560.977) [...7 remote chains...] -- 1:43:56       Average standard deviation of split frequencies: 0.012042       9960000 -- (-7557.674) [...7 remote chains...] -- 1:43:53      9970000 -- (-7556.542) [...7 remote chains...] -- 1:43:49      9980000 -- (-7551.036) [...7 remote chains...] -- 1:43:46      9990000 -- (-7579.997) [...7 remote chains...] -- 1:43:45      10000000 -- (-7546.233) [...7 remote chains...] -- 1:43:42       Average standard deviation of split frequencies: 0.011912       10010000 -- (-7584.206) [...7 remote chains...] -- 1:43:38      10020000 -- (-7559.155) [...7 remote chains...] -- 1:43:35      10030000 -- (-7605.851) [...7 remote chains...] -- 1:43:32      10040000 -- (-7567.377) [...7 remote chains...] -- 1:43:28      10050000 -- (-7568.433) [...7 remote chains...] -- 1:43:25       Average standard deviation of split frequencies: 0.011654       10060000 -- (-7565.615) [...7 remote chains...] -- 1:43:22      10070000 -- (-7568.975) [...7 remote chains...] -- 1:43:18      10080000 -- (-7564.527) [...7 remote chains...] -- 1:43:15      10090000 -- (-7560.220) [...7 remote chains...] -- 1:43:12      10100000 -- (-7551.360) [...7 remote chains...] -- 1:43:08       Average standard deviation of split frequencies: 0.011641       10110000 -- (-7562.995) [...7 remote chains...] -- 1:43:05      10120000 -- (-7561.500) [...7 remote chains...] -- 1:43:02      10130000 -- (-7556.829) [...7 remote chains...] -- 1:43:00      10140000 -- (-7551.171) [...7 remote chains...] -- 1:42:57      10150000 -- (-7543.851) [...7 remote chains...] -- 1:42:54       Average standard deviation of split frequencies: 0.011802       10160000 -- (-7546.158) [...7 remote chains...] -- 1:42:50      10170000 -- [-7546.753] [...7 remote chains...] -- 1:42:47      10180000 -- (-7543.066) [...7 remote chains...] -- 1:42:44      10190000 -- (-7554.812) [...7 remote chains...] -- 1:42:40      10200000 -- (-7569.618) [...7 remote chains...] -- 1:42:37       Average standard deviation of split frequencies: 0.011669       10210000 -- (-7556.766) [...7 remote chains...] -- 1:42:34      10220000 -- (-7564.751) [...7 remote chains...] -- 1:42:30      10230000 -- (-7569.295) [...7 remote chains...] -- 1:42:27      10240000 -- (-7553.686) [...7 remote chains...] -- 1:42:24      10250000 -- (-7564.386) [...7 remote chains...] -- 1:42:20       Average standard deviation of split frequencies: 0.011450       10260000 -- (-7568.464) [...7 remote chains...] -- 1:42:17      10270000 -- (-7575.747) [...7 remote chains...] -- 1:42:14      10280000 -- (-7552.494) [...7 remote chains...] -- 1:42:10      10290000 -- (-7550.869) [...7 remote chains...] -- 1:42:07      10300000 -- (-7555.914) [...7 remote chains...] -- 1:42:06       Average standard deviation of split frequencies: 0.011253       10310000 -- (-7557.436) [...7 remote chains...] -- 1:42:02      10320000 -- (-7543.760) [...7 remote chains...] -- 1:41:59      10330000 -- (-7560.135) [...7 remote chains...] -- 1:41:56      10340000 -- (-7542.901) [...7 remote chains...] -- 1:41:52      10350000 -- (-7554.714) [...7 remote chains...] -- 1:41:49       Average standard deviation of split frequencies: 0.011410       10360000 -- (-7563.555) [...7 remote chains...] -- 1:41:46      10370000 -- (-7554.526) [...7 remote chains...] -- 1:41:42      10380000 -- (-7566.770) [...7 remote chains...] -- 1:41:41      10390000 -- (-7555.414) [...7 remote chains...] -- 1:41:38      10400000 -- (-7557.364) [...7 remote chains...] -- 1:41:34       Average standard deviation of split frequencies: 0.011334       10410000 -- (-7575.083) [...7 remote chains...] -- 1:41:31      10420000 -- (-7559.486) [...7 remote chains...] -- 1:41:28      10430000 -- (-7547.238) [...7 remote chains...] -- 1:41:24      10440000 -- (-7575.059) [...7 remote chains...] -- 1:41:21      10450000 -- (-7555.631) [...7 remote chains...] -- 1:41:18       Average standard deviation of split frequencies: 0.011630       10460000 -- (-7566.965) [...7 remote chains...] -- 1:41:14      10470000 -- (-7549.541) [...7 remote chains...] -- 1:41:11      10480000 -- (-7561.671) [...7 remote chains...] -- 1:41:08      10490000 -- (-7547.880) [...7 remote chains...] -- 1:41:05      10500000 -- (-7559.339) [...7 remote chains...] -- 1:41:01       Average standard deviation of split frequencies: 0.011665       10510000 -- (-7539.001) [...7 remote chains...] -- 1:40:58      10520000 -- (-7560.717) [...7 remote chains...] -- 1:40:56      10530000 -- (-7557.585) [...7 remote chains...] -- 1:40:53      10540000 -- (-7569.916) [...7 remote chains...] -- 1:40:50      10550000 -- (-7549.972) [...7 remote chains...] -- 1:40:47       Average standard deviation of split frequencies: 0.011471       10560000 -- (-7586.038) [...7 remote chains...] -- 1:40:43      10570000 -- (-7544.742) [...7 remote chains...] -- 1:40:40      10580000 -- (-7569.496) [...7 remote chains...] -- 1:40:37      10590000 -- (-7569.667) [...7 remote chains...] -- 1:40:33      10600000 -- (-7559.317) [...7 remote chains...] -- 1:40:30       Average standard deviation of split frequencies: 0.011270       10610000 -- (-7554.805) [...7 remote chains...] -- 1:40:27      10620000 -- (-7546.965) [...7 remote chains...] -- 1:40:23      10630000 -- (-7554.143) [...7 remote chains...] -- 1:40:20      10640000 -- (-7556.163) [...7 remote chains...] -- 1:40:17      10650000 -- (-7555.809) [...7 remote chains...] -- 1:40:13       Average standard deviation of split frequencies: 0.011185       10660000 -- (-7548.455) [...7 remote chains...] -- 1:40:10      10670000 -- (-7560.522) [...7 remote chains...] -- 1:40:07      10680000 -- (-7556.353) [...7 remote chains...] -- 1:40:04      10690000 -- (-7563.422) [...7 remote chains...] -- 1:40:00      10700000 -- (-7567.543) [...7 remote chains...] -- 1:39:59       Average standard deviation of split frequencies: 0.011048       10710000 -- (-7562.002) [...7 remote chains...] -- 1:39:55      10720000 -- (-7575.787) [...7 remote chains...] -- 1:39:52      10730000 -- (-7576.454) [...7 remote chains...] -- 1:39:49      10740000 -- (-7562.946) [...7 remote chains...] -- 1:39:46      10750000 -- (-7572.918) [...7 remote chains...] -- 1:39:42       Average standard deviation of split frequencies: 0.010880       10760000 -- (-7581.950) [...7 remote chains...] -- 1:39:39      10770000 -- (-7554.974) [...7 remote chains...] -- 1:39:36      10780000 -- (-7574.901) [...7 remote chains...] -- 1:39:32      10790000 -- (-7563.296) [...7 remote chains...] -- 1:39:29      10800000 -- (-7567.491) [...7 remote chains...] -- 1:39:26       Average standard deviation of split frequencies: 0.010863       10810000 -- (-7573.524) [...7 remote chains...] -- 1:39:22      10820000 -- (-7580.863) [...7 remote chains...] -- 1:39:19      10830000 -- (-7573.429) [...7 remote chains...] -- 1:39:16      10840000 -- (-7550.582) [...7 remote chains...] -- 1:39:13      10850000 -- (-7559.221) [...7 remote chains...] -- 1:39:09       Average standard deviation of split frequencies: 0.011030       10860000 -- (-7559.310) [...7 remote chains...] -- 1:39:06      10870000 -- (-7576.625) [...7 remote chains...] -- 1:39:03      10880000 -- (-7555.976) [...7 remote chains...] -- 1:38:59      10890000 -- (-7548.874) [...7 remote chains...] -- 1:38:56      10900000 -- (-7534.335) [...7 remote chains...] -- 1:38:55       Average standard deviation of split frequencies: 0.010970       10910000 -- (-7552.964) [...7 remote chains...] -- 1:38:51      10920000 -- (-7559.036) [...7 remote chains...] -- 1:38:48      10930000 -- (-7579.581) [...7 remote chains...] -- 1:38:45      10940000 -- (-7571.035) [...7 remote chains...] -- 1:38:41      10950000 -- (-7571.650) [...7 remote chains...] -- 1:38:38       Average standard deviation of split frequencies: 0.010709       10960000 -- (-7556.594) [...7 remote chains...] -- 1:38:35      10970000 -- (-7562.335) [...7 remote chains...] -- 1:38:31      10980000 -- (-7576.247) [...7 remote chains...] -- 1:38:28      10990000 -- (-7544.029) [...7 remote chains...] -- 1:38:25      11000000 -- [-7532.843] [...7 remote chains...] -- 1:38:22       Average standard deviation of split frequencies: 0.010415       11010000 -- [-7557.098] [...7 remote chains...] -- 1:38:20      11020000 -- [-7575.212] [...7 remote chains...] -- 1:38:17      11030000 -- [-7576.069] [...7 remote chains...] -- 1:38:13      11040000 -- [-7545.410] [...7 remote chains...] -- 1:38:10      11050000 -- (-7574.674) [...7 remote chains...] -- 1:38:07       Average standard deviation of split frequencies: 0.010303       11060000 -- (-7570.194) [...7 remote chains...] -- 1:38:04      11070000 -- (-7567.363) [...7 remote chains...] -- 1:38:00      11080000 -- (-7552.083) [...7 remote chains...] -- 1:37:57      11090000 -- (-7554.035) [...7 remote chains...] -- 1:37:54      11100000 -- (-7557.945) [...7 remote chains...] -- 1:37:50       Average standard deviation of split frequencies: 0.010350       11110000 -- (-7579.255) [...7 remote chains...] -- 1:37:47      11120000 -- (-7557.292) [...7 remote chains...] -- 1:37:44      11130000 -- (-7553.430) [...7 remote chains...] -- 1:37:41      11140000 -- (-7577.790) [...7 remote chains...] -- 1:37:37      11150000 -- (-7560.493) [...7 remote chains...] -- 1:37:34       Average standard deviation of split frequencies: 0.010446       11160000 -- (-7549.461) [...7 remote chains...] -- 1:37:32      11170000 -- (-7541.422) [...7 remote chains...] -- 1:37:29      11180000 -- (-7563.151) [...7 remote chains...] -- 1:37:26      11190000 -- (-7575.077) [...7 remote chains...] -- 1:37:23      11200000 -- (-7578.102) [...7 remote chains...] -- 1:37:19       Average standard deviation of split frequencies: 0.010200       11210000 -- (-7553.003) [...7 remote chains...] -- 1:37:16      11220000 -- (-7564.339) [...7 remote chains...] -- 1:37:13      11230000 -- [-7549.866] [...7 remote chains...] -- 1:37:09      11240000 -- [-7559.578] [...7 remote chains...] -- 1:37:06      11250000 -- [-7529.122] [...7 remote chains...] -- 1:37:03       Average standard deviation of split frequencies: 0.010046       11260000 -- [-7539.382] [...7 remote chains...] -- 1:37:00      11270000 -- [-7527.410] [...7 remote chains...] -- 1:36:56      11280000 -- (-7543.202) [...7 remote chains...] -- 1:36:53      11290000 -- (-7542.360) [...7 remote chains...] -- 1:36:50      11300000 -- (-7547.550) [...7 remote chains...] -- 1:36:46       Average standard deviation of split frequencies: 0.009988       11310000 -- (-7562.301) [...7 remote chains...] -- 1:36:43      11320000 -- (-7565.013) [...7 remote chains...] -- 1:36:40      11330000 -- (-7584.823) [...7 remote chains...] -- 1:36:37      11340000 -- (-7549.221) [...7 remote chains...] -- 1:36:33      11350000 -- (-7555.961) [...7 remote chains...] -- 1:36:30       Average standard deviation of split frequencies: 0.009953       11360000 -- (-7565.535) [...7 remote chains...] -- 1:36:27      11370000 -- [-7542.730] [...7 remote chains...] -- 1:36:23      11380000 -- [-7553.855] [...7 remote chains...] -- 1:36:20      11390000 -- (-7554.358) [...7 remote chains...] -- 1:36:17      11400000 -- (-7538.789) [...7 remote chains...] -- 1:36:14       Average standard deviation of split frequencies: 0.009561       11410000 -- (-7557.872) [...7 remote chains...] -- 1:36:12      11420000 -- (-7563.084) [...7 remote chains...] -- 1:36:07      11430000 -- (-7559.425) [...7 remote chains...] -- 1:36:05      11440000 -- (-7547.250) [...7 remote chains...] -- 1:36:02      11450000 -- (-7548.434) [...7 remote chains...] -- 1:35:59       Average standard deviation of split frequencies: 0.009614       11460000 -- (-7540.458) [...7 remote chains...] -- 1:35:56      11470000 -- (-7555.884) [...7 remote chains...] -- 1:35:52      11480000 -- (-7554.363) [...7 remote chains...] -- 1:35:49      11490000 -- (-7556.850) [...7 remote chains...] -- 1:35:46      11500000 -- (-7550.428) [...7 remote chains...] -- 1:35:43       Average standard deviation of split frequencies: 0.009582       11510000 -- (-7555.643) [...7 remote chains...] -- 1:35:39      11520000 -- (-7540.630) [...7 remote chains...] -- 1:35:36      11530000 -- (-7557.286) [...7 remote chains...] -- 1:35:33      11540000 -- (-7575.673) [...7 remote chains...] -- 1:35:29      11550000 -- (-7567.825) [...7 remote chains...] -- 1:35:26       Average standard deviation of split frequencies: 0.009409       11560000 -- (-7557.794) [...7 remote chains...] -- 1:35:23      11570000 -- (-7562.913) [...7 remote chains...] -- 1:35:20      11580000 -- (-7556.929) [...7 remote chains...] -- 1:35:16      11590000 -- (-7584.902) [...7 remote chains...] -- 1:35:13      11600000 -- (-7552.311) [...7 remote chains...] -- 1:35:10       Average standard deviation of split frequencies: 0.009318       11610000 -- (-7552.070) [...7 remote chains...] -- 1:35:07      11620000 -- (-7588.646) [...7 remote chains...] -- 1:35:05      11630000 -- (-7570.515) [...7 remote chains...] -- 1:35:02      11640000 -- (-7558.463) [...7 remote chains...] -- 1:34:58      11650000 -- (-7568.874) [...7 remote chains...] -- 1:34:55       Average standard deviation of split frequencies: 0.009626       11660000 -- (-7572.941) [...7 remote chains...] -- 1:34:52      11670000 -- (-7568.418) [...7 remote chains...] -- 1:34:49      11680000 -- (-7594.825) [...7 remote chains...] -- 1:34:45      11690000 -- (-7560.828) [...7 remote chains...] -- 1:34:42      11700000 -- (-7552.214) [...7 remote chains...] -- 1:34:39       Average standard deviation of split frequencies: 0.009535       11710000 -- (-7568.858) [...7 remote chains...] -- 1:34:35      11720000 -- (-7554.216) [...7 remote chains...] -- 1:34:32      11730000 -- (-7568.373) [...7 remote chains...] -- 1:34:29      11740000 -- (-7547.140) [...7 remote chains...] -- 1:34:26      11750000 -- (-7546.669) [...7 remote chains...] -- 1:34:22       Average standard deviation of split frequencies: 0.009502       11760000 -- (-7564.991) [...7 remote chains...] -- 1:34:19      11770000 -- (-7549.856) [...7 remote chains...] -- 1:34:16      11780000 -- (-7543.378) [...7 remote chains...] -- 1:34:13      11790000 -- (-7546.345) [...7 remote chains...] -- 1:34:11      11800000 -- (-7562.348) [...7 remote chains...] -- 1:34:08       Average standard deviation of split frequencies: 0.009408       11810000 -- (-7561.630) [...7 remote chains...] -- 1:34:04      11820000 -- (-7562.531) [...7 remote chains...] -- 1:34:01      11830000 -- (-7550.670) [...7 remote chains...] -- 1:33:58      11840000 -- (-7575.754) [...7 remote chains...] -- 1:33:55      11850000 -- (-7553.397) [...7 remote chains...] -- 1:33:51       Average standard deviation of split frequencies: 0.009399       11860000 -- (-7571.741) [...7 remote chains...] -- 1:33:48      11870000 -- (-7557.718) [...7 remote chains...] -- 1:33:45      11880000 -- (-7585.600) [...7 remote chains...] -- 1:33:42      11890000 -- (-7548.242) [...7 remote chains...] -- 1:33:38      11900000 -- (-7551.989) [...7 remote chains...] -- 1:33:35       Average standard deviation of split frequencies: 0.009348       11910000 -- (-7551.256) [...7 remote chains...] -- 1:33:32      11920000 -- (-7545.387) [...7 remote chains...] -- 1:33:29      11930000 -- (-7539.298) [...7 remote chains...] -- 1:33:25      11940000 -- (-7547.757) [...7 remote chains...] -- 1:33:22      11950000 -- (-7579.992) [...7 remote chains...] -- 1:33:19       Average standard deviation of split frequencies: 0.009248       11960000 -- (-7552.034) [...7 remote chains...] -- 1:33:17      11970000 -- (-7559.806) [...7 remote chains...] -- 1:33:14      11980000 -- (-7556.401) [...7 remote chains...] -- 1:33:11      11990000 -- (-7545.830) [...7 remote chains...] -- 1:33:07      12000000 -- (-7530.705) [...7 remote chains...] -- 1:33:04       Average standard deviation of split frequencies: 0.009392       12010000 -- (-7536.804) [...7 remote chains...] -- 1:33:01      12020000 -- (-7551.612) [...7 remote chains...] -- 1:32:57      12030000 -- (-7558.835) [...7 remote chains...] -- 1:32:54      12040000 -- (-7545.697) [...7 remote chains...] -- 1:32:51      12050000 -- (-7567.826) [...7 remote chains...] -- 1:32:48       Average standard deviation of split frequencies: 0.009552       12060000 -- (-7569.481) [...7 remote chains...] -- 1:32:44      12070000 -- (-7555.701) [...7 remote chains...] -- 1:32:41      12080000 -- (-7550.097) [...7 remote chains...] -- 1:32:38      12090000 -- (-7567.782) [...7 remote chains...] -- 1:32:35      12100000 -- (-7554.224) [...7 remote chains...] -- 1:32:33       Average standard deviation of split frequencies: 0.009621       12110000 -- (-7553.894) [...7 remote chains...] -- 1:32:30      12120000 -- [-7553.347] [...7 remote chains...] -- 1:32:26      12130000 -- [-7556.200] [...7 remote chains...] -- 1:32:23      12140000 -- (-7559.675) [...7 remote chains...] -- 1:32:20      12150000 -- [-7545.336] [...7 remote chains...] -- 1:32:17       Average standard deviation of split frequencies: 0.009598       12160000 -- (-7554.718) [...7 remote chains...] -- 1:32:13      12170000 -- (-7571.942) [...7 remote chains...] -- 1:32:10      12180000 -- (-7556.036) [...7 remote chains...] -- 1:32:07      12190000 -- (-7569.547) [...7 remote chains...] -- 1:32:04      12200000 -- (-7567.375) [...7 remote chains...] -- 1:32:00       Average standard deviation of split frequencies: 0.009789       12210000 -- (-7575.363) [...7 remote chains...] -- 1:31:57      12220000 -- (-7563.720) [...7 remote chains...] -- 1:31:54      12230000 -- (-7578.508) [...7 remote chains...] -- 1:31:51      12240000 -- (-7579.026) [...7 remote chains...] -- 1:31:47      12250000 -- (-7570.469) [...7 remote chains...] -- 1:31:44       Average standard deviation of split frequencies: 0.009952       12260000 -- (-7552.293) [...7 remote chains...] -- 1:31:41      12270000 -- (-7549.902) [...7 remote chains...] -- 1:31:38      12280000 -- (-7565.248) [...7 remote chains...] -- 1:31:34      12290000 -- (-7553.205) [...7 remote chains...] -- 1:31:31      12300000 -- (-7556.463) [...7 remote chains...] -- 1:31:28       Average standard deviation of split frequencies: 0.010149       12310000 -- (-7569.262) [...7 remote chains...] -- 1:31:25      12320000 -- (-7551.004) [...7 remote chains...] -- 1:31:21      12330000 -- (-7547.935) [...7 remote chains...] -- 1:31:18      12340000 -- (-7556.451) [...7 remote chains...] -- 1:31:15      12350000 -- (-7542.164) [...7 remote chains...] -- 1:31:12       Average standard deviation of split frequencies: 0.010131       12360000 -- (-7560.025) [...7 remote chains...] -- 1:31:10      12370000 -- (-7564.297) [...7 remote chains...] -- 1:31:07      12380000 -- (-7579.893) [...7 remote chains...] -- 1:31:03      12390000 -- (-7565.606) [...7 remote chains...] -- 1:31:00      12400000 -- (-7581.140) [...7 remote chains...] -- 1:30:57       Average standard deviation of split frequencies: 0.010559       12410000 -- (-7572.795) [...7 remote chains...] -- 1:30:54      12420000 -- (-7565.834) [...7 remote chains...] -- 1:30:50      12430000 -- (-7595.364) [...7 remote chains...] -- 1:30:47      12440000 -- (-7554.854) [...7 remote chains...] -- 1:30:44      12450000 -- (-7577.627) [...7 remote chains...] -- 1:30:41       Average standard deviation of split frequencies: 0.010421       12460000 -- (-7568.156) [...7 remote chains...] -- 1:30:37      12470000 -- (-7567.207) [...7 remote chains...] -- 1:30:34      12480000 -- (-7550.812) [...7 remote chains...] -- 1:30:32      12490000 -- (-7562.921) [...7 remote chains...] -- 1:30:29      12500000 -- (-7545.415) [...7 remote chains...] -- 1:30:26       Average standard deviation of split frequencies: 0.010180       12510000 -- (-7540.964) [...7 remote chains...] -- 1:30:23      12520000 -- (-7542.211) [...7 remote chains...] -- 1:30:19      12530000 -- (-7564.696) [...7 remote chains...] -- 1:30:16      12540000 -- (-7562.100) [...7 remote chains...] -- 1:30:13      12550000 -- (-7571.382) [...7 remote chains...] -- 1:30:10       Average standard deviation of split frequencies: 0.010125       12560000 -- (-7568.086) [...7 remote chains...] -- 1:30:06      12570000 -- (-7562.819) [...7 remote chains...] -- 1:30:03      12580000 -- (-7557.524) [...7 remote chains...] -- 1:30:00      12590000 -- (-7545.799) [...7 remote chains...] -- 1:29:57      12600000 -- (-7546.833) [...7 remote chains...] -- 1:29:54       Average standard deviation of split frequencies: 0.010140       12610000 -- (-7556.607) [...7 remote chains...] -- 1:29:50      12620000 -- (-7552.991) [...7 remote chains...] -- 1:29:47      12630000 -- (-7570.396) [...7 remote chains...] -- 1:29:44      12640000 -- (-7565.561) [...7 remote chains...] -- 1:29:41      12650000 -- (-7556.703) [...7 remote chains...] -- 1:29:37       Average standard deviation of split frequencies: 0.010186       12660000 -- (-7561.169) [...7 remote chains...] -- 1:29:34      12670000 -- (-7555.462) [...7 remote chains...] -- 1:29:32      12680000 -- (-7579.872) [...7 remote chains...] -- 1:29:29      12690000 -- (-7573.206) [...7 remote chains...] -- 1:29:26      12700000 -- (-7568.892) [...7 remote chains...] -- 1:29:23       Average standard deviation of split frequencies: 0.010055       12710000 -- (-7562.583) [...7 remote chains...] -- 1:29:19      12720000 -- (-7555.084) [...7 remote chains...] -- 1:29:16      12730000 -- (-7559.045) [...7 remote chains...] -- 1:29:13      12740000 -- (-7557.723) [...7 remote chains...] -- 1:29:10      12750000 -- (-7567.706) [...7 remote chains...] -- 1:29:06       Average standard deviation of split frequencies: 0.009950       12760000 -- (-7569.142) [...7 remote chains...] -- 1:29:03      12770000 -- (-7564.792) [...7 remote chains...] -- 1:29:00      12780000 -- (-7563.525) [...7 remote chains...] -- 1:28:57      12790000 -- (-7554.884) [...7 remote chains...] -- 1:28:55      12800000 -- (-7563.380) [...7 remote chains...] -- 1:28:52       Average standard deviation of split frequencies: 0.010153       12810000 -- (-7550.081) [...7 remote chains...] -- 1:28:48      12820000 -- (-7535.072) [...7 remote chains...] -- 1:28:45      12830000 -- (-7560.408) [...7 remote chains...] -- 1:28:42      12840000 -- (-7577.886) [...7 remote chains...] -- 1:28:39      12850000 -- (-7564.707) [...7 remote chains...] -- 1:28:35       Average standard deviation of split frequencies: 0.010356       12860000 -- (-7557.184) [...7 remote chains...] -- 1:28:32      12870000 -- (-7562.008) [...7 remote chains...] -- 1:28:29      12880000 -- (-7566.765) [...7 remote chains...] -- 1:28:26      12890000 -- (-7574.828) [...7 remote chains...] -- 1:28:22      12900000 -- (-7541.353) [...7 remote chains...] -- 1:28:19       Average standard deviation of split frequencies: 0.010468       12910000 -- (-7559.825) [...7 remote chains...] -- 1:28:16      12920000 -- (-7570.252) [...7 remote chains...] -- 1:28:13      12930000 -- (-7566.456) [...7 remote chains...] -- 1:28:09      12940000 -- (-7571.406) [...7 remote chains...] -- 1:28:06      12950000 -- (-7569.168) [...7 remote chains...] -- 1:28:03       Average standard deviation of split frequencies: 0.010370       12960000 -- (-7570.005) [...7 remote chains...] -- 1:28:00      12970000 -- (-7538.975) [...7 remote chains...] -- 1:27:57      12980000 -- (-7547.672) [...7 remote chains...] -- 1:27:53      12990000 -- (-7560.892) [...7 remote chains...] -- 1:27:50      13000000 -- (-7587.857) [...7 remote chains...] -- 1:27:47       Average standard deviation of split frequencies: 0.010397       13010000 -- (-7568.836) [...7 remote chains...] -- 1:27:44      13020000 -- (-7568.704) [...7 remote chains...] -- 1:27:40      13030000 -- (-7563.286) [...7 remote chains...] -- 1:27:37      13040000 -- (-7555.096) [...7 remote chains...] -- 1:27:35      13050000 -- (-7553.625) [...7 remote chains...] -- 1:27:32       Average standard deviation of split frequencies: 0.010457       13060000 -- (-7568.903) [...7 remote chains...] -- 1:27:29      13070000 -- (-7575.440) [...7 remote chains...] -- 1:27:26      13080000 -- (-7561.707) [...7 remote chains...] -- 1:27:22      13090000 -- (-7557.394) [...7 remote chains...] -- 1:27:19      13100000 -- (-7570.662) [...7 remote chains...] -- 1:27:16       Average standard deviation of split frequencies: 0.010292       13110000 -- (-7578.178) [...7 remote chains...] -- 1:27:13      13120000 -- (-7562.041) [...7 remote chains...] -- 1:27:09      13130000 -- (-7575.800) [...7 remote chains...] -- 1:27:06      13140000 -- (-7548.984) [...7 remote chains...] -- 1:27:03      13150000 -- (-7560.070) [...7 remote chains...] -- 1:27:00       Average standard deviation of split frequencies: 0.010133       13160000 -- (-7553.486) [...7 remote chains...] -- 1:26:57      13170000 -- (-7543.327) [...7 remote chains...] -- 1:26:53      13180000 -- (-7550.442) [...7 remote chains...] -- 1:26:50      13190000 -- (-7550.660) [...7 remote chains...] -- 1:26:47      13200000 -- (-7568.781) [...7 remote chains...] -- 1:26:44       Average standard deviation of split frequencies: 0.010060       13210000 -- (-7556.569) [...7 remote chains...] -- 1:26:40      13220000 -- (-7580.885) [...7 remote chains...] -- 1:26:37      13230000 -- (-7555.459) [...7 remote chains...] -- 1:26:34      13240000 -- (-7554.026) [...7 remote chains...] -- 1:26:31      13250000 -- (-7546.759) [...7 remote chains...] -- 1:26:28       Average standard deviation of split frequencies: 0.009828       13260000 -- (-7574.727) [...7 remote chains...] -- 1:26:24      13270000 -- (-7564.818) [...7 remote chains...] -- 1:26:21      13280000 -- (-7570.359) [...7 remote chains...] -- 1:26:18      13290000 -- (-7542.544) [...7 remote chains...] -- 1:26:16      13300000 -- (-7557.967) [...7 remote chains...] -- 1:26:13       Average standard deviation of split frequencies: 0.009872       13310000 -- (-7552.226) [...7 remote chains...] -- 1:26:10      13320000 -- (-7537.526) [...7 remote chains...] -- 1:26:06      13330000 -- (-7559.663) [...7 remote chains...] -- 1:26:03      13340000 -- (-7549.955) [...7 remote chains...] -- 1:26:00      13350000 -- (-7568.201) [...7 remote chains...] -- 1:25:57       Average standard deviation of split frequencies: 0.009980       13360000 -- [-7552.044] [...7 remote chains...] -- 1:25:53      13370000 -- (-7548.213) [...7 remote chains...] -- 1:25:50      13380000 -- [-7539.738] [...7 remote chains...] -- 1:25:47      13390000 -- [-7552.774] [...7 remote chains...] -- 1:25:44      13400000 -- (-7558.466) [...7 remote chains...] -- 1:25:41       Average standard deviation of split frequencies: 0.010105       13410000 -- (-7553.013) [...7 remote chains...] -- 1:25:37      13420000 -- (-7556.766) [...7 remote chains...] -- 1:25:34      13430000 -- (-7553.751) [...7 remote chains...] -- 1:25:31      13440000 -- (-7566.649) [...7 remote chains...] -- 1:25:28      13450000 -- (-7548.292) [...7 remote chains...] -- 1:25:24       Average standard deviation of split frequencies: 0.010170       13460000 -- (-7555.607) [...7 remote chains...] -- 1:25:21      13470000 -- (-7543.698) [...7 remote chains...] -- 1:25:18      13480000 -- (-7570.062) [...7 remote chains...] -- 1:25:15      13490000 -- (-7566.204) [...7 remote chains...] -- 1:25:12      13500000 -- (-7560.635) [...7 remote chains...] -- 1:25:10       Average standard deviation of split frequencies: 0.010095       13510000 -- (-7563.020) [...7 remote chains...] -- 1:25:06      13520000 -- (-7576.516) [...7 remote chains...] -- 1:25:03      13530000 -- (-7589.723) [...7 remote chains...] -- 1:25:00      13540000 -- (-7544.406) [...7 remote chains...] -- 1:24:57      13550000 -- (-7550.799) [...7 remote chains...] -- 1:24:54       Average standard deviation of split frequencies: 0.010016       13560000 -- (-7555.609) [...7 remote chains...] -- 1:24:50      13570000 -- (-7561.738) [...7 remote chains...] -- 1:24:47      13580000 -- (-7581.811) [...7 remote chains...] -- 1:24:44      13590000 -- (-7561.785) [...7 remote chains...] -- 1:24:41      13600000 -- (-7570.918) [...7 remote chains...] -- 1:24:37       Average standard deviation of split frequencies: 0.010050       13610000 -- (-7553.362) [...7 remote chains...] -- 1:24:34      13620000 -- (-7566.045) [...7 remote chains...] -- 1:24:31      13630000 -- (-7555.563) [...7 remote chains...] -- 1:24:28      13640000 -- (-7543.210) [...7 remote chains...] -- 1:24:25      13650000 -- (-7550.071) [...7 remote chains...] -- 1:24:21       Average standard deviation of split frequencies: 0.010021       13660000 -- (-7557.471) [...7 remote chains...] -- 1:24:18      13670000 -- (-7531.264) [...7 remote chains...] -- 1:24:15      13680000 -- (-7524.806) [...7 remote chains...] -- 1:24:12      13690000 -- (-7570.278) [...7 remote chains...] -- 1:24:09      13700000 -- (-7572.554) [...7 remote chains...] -- 1:24:05       Average standard deviation of split frequencies: 0.010044       13710000 -- (-7564.166) [...7 remote chains...] -- 1:24:02      13720000 -- (-7550.584) [...7 remote chains...] -- 1:23:59      13730000 -- (-7551.522) [...7 remote chains...] -- 1:23:56      13740000 -- (-7546.686) [...7 remote chains...] -- 1:23:53      13750000 -- (-7556.080) [...7 remote chains...] -- 1:23:51       Average standard deviation of split frequencies: 0.010209       13760000 -- (-7547.523) [...7 remote chains...] -- 1:23:47      13770000 -- (-7563.482) [...7 remote chains...] -- 1:23:44      13780000 -- (-7554.311) [...7 remote chains...] -- 1:23:41      13790000 -- (-7562.801) [...7 remote chains...] -- 1:23:38      13800000 -- (-7544.765) [...7 remote chains...] -- 1:23:34       Average standard deviation of split frequencies: 0.010359       13810000 -- (-7564.931) [...7 remote chains...] -- 1:23:31      13820000 -- (-7558.348) [...7 remote chains...] -- 1:23:28      13830000 -- (-7574.674) [...7 remote chains...] -- 1:23:25      13840000 -- (-7544.440) [...7 remote chains...] -- 1:23:22      13850000 -- (-7558.707) [...7 remote chains...] -- 1:23:18       Average standard deviation of split frequencies: 0.010575       13860000 -- (-7552.497) [...7 remote chains...] -- 1:23:15      13870000 -- (-7549.311) [...7 remote chains...] -- 1:23:12      13880000 -- [-7553.795] [...7 remote chains...] -- 1:23:09      13890000 -- [-7549.395] [...7 remote chains...] -- 1:23:06      13900000 -- (-7560.783) [...7 remote chains...] -- 1:23:02       Average standard deviation of split frequencies: 0.010520       13910000 -- (-7546.160) [...7 remote chains...] -- 1:23:00      13920000 -- (-7555.001) [...7 remote chains...] -- 1:22:57      13930000 -- (-7565.730) [...7 remote chains...] -- 1:22:54      13940000 -- (-7554.705) [...7 remote chains...] -- 1:22:51      13950000 -- (-7550.169) [...7 remote chains...] -- 1:22:48       Average standard deviation of split frequencies: 0.010664       13960000 -- (-7555.609) [...7 remote chains...] -- 1:22:44      13970000 -- (-7541.610) [...7 remote chains...] -- 1:22:41      13980000 -- (-7554.884) [...7 remote chains...] -- 1:22:38      13990000 -- (-7557.871) [...7 remote chains...] -- 1:22:35      14000000 -- (-7557.283) [...7 remote chains...] -- 1:22:32       Average standard deviation of split frequencies: 0.010606       14010000 -- (-7545.927) [...7 remote chains...] -- 1:22:28      14020000 -- (-7551.595) [...7 remote chains...] -- 1:22:25      14030000 -- (-7548.496) [...7 remote chains...] -- 1:22:22      14040000 -- (-7544.196) [...7 remote chains...] -- 1:22:19      14050000 -- (-7542.633) [...7 remote chains...] -- 1:22:15       Average standard deviation of split frequencies: 0.010521       14060000 -- (-7545.577) [...7 remote chains...] -- 1:22:12      14070000 -- (-7563.972) [...7 remote chains...] -- 1:22:09      14080000 -- (-7542.307) [...7 remote chains...] -- 1:22:06      14090000 -- [-7543.303] [...7 remote chains...] -- 1:22:03      14100000 -- [-7541.256] [...7 remote chains...] -- 1:21:59       Average standard deviation of split frequencies: 0.010483       14110000 -- [-7544.874] [...7 remote chains...] -- 1:21:56      14120000 -- [-7557.924] [...7 remote chains...] -- 1:21:53      14130000 -- [-7561.249] [...7 remote chains...] -- 1:21:50      14140000 -- [-7553.841] [...7 remote chains...] -- 1:21:47      14150000 -- [-7555.244] [...7 remote chains...] -- 1:21:43       Average standard deviation of split frequencies: 0.010640       14160000 -- [-7553.909] [...7 remote chains...] -- 1:21:41      14170000 -- [-7545.126] [...7 remote chains...] -- 1:21:38      14180000 -- [-7554.650] [...7 remote chains...] -- 1:21:35      14190000 -- [-7541.111] [...7 remote chains...] -- 1:21:32      14200000 -- [-7542.568] [...7 remote chains...] -- 1:21:29       Average standard deviation of split frequencies: 0.010612       14210000 -- [-7548.904] [...7 remote chains...] -- 1:21:25      14220000 -- [-7549.051] [...7 remote chains...] -- 1:21:22      14230000 -- [-7549.755] [...7 remote chains...] -- 1:21:19      14240000 -- (-7559.465) [...7 remote chains...] -- 1:21:16      14250000 -- [-7564.634] [...7 remote chains...] -- 1:21:13       Average standard deviation of split frequencies: 0.010425       14260000 -- [-7555.190] [...7 remote chains...] -- 1:21:09      14270000 -- [-7561.898] [...7 remote chains...] -- 1:21:06      14280000 -- [-7547.094] [...7 remote chains...] -- 1:21:03      14290000 -- [-7545.313] [...7 remote chains...] -- 1:21:00      14300000 -- [-7542.930] [...7 remote chains...] -- 1:20:57       Average standard deviation of split frequencies: 0.010420       14310000 -- [-7559.999] [...7 remote chains...] -- 1:20:53      14320000 -- [-7570.733] [...7 remote chains...] -- 1:20:50      14330000 -- (-7561.466) [...7 remote chains...] -- 1:20:47      14340000 -- (-7561.495) [...7 remote chains...] -- 1:20:44      14350000 -- (-7546.303) [...7 remote chains...] -- 1:20:42       Average standard deviation of split frequencies: 0.010457       14360000 -- (-7549.775) [...7 remote chains...] -- 1:20:39      14370000 -- (-7566.820) [...7 remote chains...] -- 1:20:35      14380000 -- (-7571.177) [...7 remote chains...] -- 1:20:32      14390000 -- (-7570.226) [...7 remote chains...] -- 1:20:29      14400000 -- (-7540.210) [...7 remote chains...] -- 1:20:26       Average standard deviation of split frequencies: 0.010448       14410000 -- (-7576.441) [...7 remote chains...] -- 1:20:23      14420000 -- (-7562.879) [...7 remote chains...] -- 1:20:19      14430000 -- (-7565.566) [...7 remote chains...] -- 1:20:16      14440000 -- (-7568.263) [...7 remote chains...] -- 1:20:13      14450000 -- (-7536.913) [...7 remote chains...] -- 1:20:10       Average standard deviation of split frequencies: 0.010464       14460000 -- (-7560.062) [...7 remote chains...] -- 1:20:07      14470000 -- (-7548.199) [...7 remote chains...] -- 1:20:03      14480000 -- (-7572.767) [...7 remote chains...] -- 1:20:00      14490000 -- (-7567.986) [...7 remote chains...] -- 1:19:57      14500000 -- (-7556.994) [...7 remote chains...] -- 1:19:54       Average standard deviation of split frequencies: 0.010499       14510000 -- (-7537.675) [...7 remote chains...] -- 1:19:51      14520000 -- (-7568.610) [...7 remote chains...] -- 1:19:47      14530000 -- (-7562.000) [...7 remote chains...] -- 1:19:44      14540000 -- (-7561.309) [...7 remote chains...] -- 1:19:41      14550000 -- (-7551.621) [...7 remote chains...] -- 1:19:38       Average standard deviation of split frequencies: 0.010552       14560000 -- (-7552.967) [...7 remote chains...] -- 1:19:35      14570000 -- (-7541.908) [...7 remote chains...] -- 1:19:31      14580000 -- (-7559.415) [...7 remote chains...] -- 1:19:28      14590000 -- (-7558.980) [...7 remote chains...] -- 1:19:25      14600000 -- (-7551.567) [...7 remote chains...] -- 1:19:23       Average standard deviation of split frequencies: 0.010500       14610000 -- (-7574.801) [...7 remote chains...] -- 1:19:20      14620000 -- (-7566.100) [...7 remote chains...] -- 1:19:17      14630000 -- (-7580.383) [...7 remote chains...] -- 1:19:13      14640000 -- (-7574.936) [...7 remote chains...] -- 1:19:10      14650000 -- (-7541.031) [...7 remote chains...] -- 1:19:07       Average standard deviation of split frequencies: 0.010496       14660000 -- (-7543.714) [...7 remote chains...] -- 1:19:04      14670000 -- (-7528.310) [...7 remote chains...] -- 1:19:01      14680000 -- (-7558.942) [...7 remote chains...] -- 1:18:57      14690000 -- (-7557.992) [...7 remote chains...] -- 1:18:54      14700000 -- (-7540.708) [...7 remote chains...] -- 1:18:51       Average standard deviation of split frequencies: 0.010553       14710000 -- (-7571.374) [...7 remote chains...] -- 1:18:48      14720000 -- (-7548.272) [...7 remote chains...] -- 1:18:45      14730000 -- (-7552.114) [...7 remote chains...] -- 1:18:41      14740000 -- (-7568.817) [...7 remote chains...] -- 1:18:38      14750000 -- (-7556.305) [...7 remote chains...] -- 1:18:35       Average standard deviation of split frequencies: 0.010468       14760000 -- (-7538.584) [...7 remote chains...] -- 1:18:32      14770000 -- (-7522.147) [...7 remote chains...] -- 1:18:29      14780000 -- (-7552.261) [...7 remote chains...] -- 1:18:26      14790000 -- (-7560.584) [...7 remote chains...] -- 1:18:22      14800000 -- (-7553.031) [...7 remote chains...] -- 1:18:19       Average standard deviation of split frequencies: 0.010581       14810000 -- (-7564.182) [...7 remote chains...] -- 1:18:16      14820000 -- (-7554.062) [...7 remote chains...] -- 1:18:13      14830000 -- (-7551.596) [...7 remote chains...] -- 1:18:10      14840000 -- (-7562.885) [...7 remote chains...] -- 1:18:06      14850000 -- (-7550.892) [...7 remote chains...] -- 1:18:03       Average standard deviation of split frequencies: 0.010368       14860000 -- (-7570.054) [...7 remote chains...] -- 1:18:00      14870000 -- (-7597.481) [...7 remote chains...] -- 1:17:57      14880000 -- (-7571.299) [...7 remote chains...] -- 1:17:54      14890000 -- (-7561.403) [...7 remote chains...] -- 1:17:51      14900000 -- (-7565.450) [...7 remote chains...] -- 1:17:47       Average standard deviation of split frequencies: 0.010408       14910000 -- (-7549.708) [...7 remote chains...] -- 1:17:44      14920000 -- (-7555.727) [...7 remote chains...] -- 1:17:42      14930000 -- (-7535.718) [...7 remote chains...] -- 1:17:39      14940000 -- (-7552.097) [...7 remote chains...] -- 1:17:36      14950000 -- (-7566.138) [...7 remote chains...] -- 1:17:32       Average standard deviation of split frequencies: 0.010433       14960000 -- (-7570.554) [...7 remote chains...] -- 1:17:29      14970000 -- (-7552.401) [...7 remote chains...] -- 1:17:26      14980000 -- (-7571.737) [...7 remote chains...] -- 1:17:23      14990000 -- (-7543.478) [...7 remote chains...] -- 1:17:20      15000000 -- (-7527.870) [...7 remote chains...] -- 1:17:17       Average standard deviation of split frequencies: 0.010530       15010000 -- (-7566.985) [...7 remote chains...] -- 1:17:13      15020000 -- (-7568.034) [...7 remote chains...] -- 1:17:10      15030000 -- (-7541.913) [...7 remote chains...] -- 1:17:07      15040000 -- (-7545.476) [...7 remote chains...] -- 1:17:04      15050000 -- (-7561.397) [...7 remote chains...] -- 1:17:01       Average standard deviation of split frequencies: 0.010435       15060000 -- [-7542.461] [...7 remote chains...] -- 1:16:57      15070000 -- [-7535.317] [...7 remote chains...] -- 1:16:54      15080000 -- (-7557.032) [...7 remote chains...] -- 1:16:51      15090000 -- (-7563.769) [...7 remote chains...] -- 1:16:48      15100000 -- (-7542.080) [...7 remote chains...] -- 1:16:45       Average standard deviation of split frequencies: 0.010176       15110000 -- (-7555.519) [...7 remote chains...] -- 1:16:42      15120000 -- (-7549.372) [...7 remote chains...] -- 1:16:38      15130000 -- (-7558.616) [...7 remote chains...] -- 1:16:35      15140000 -- (-7555.539) [...7 remote chains...] -- 1:16:32      15150000 -- (-7552.295) [...7 remote chains...] -- 1:16:29       Average standard deviation of split frequencies: 0.009781       15160000 -- (-7524.381) [...7 remote chains...] -- 1:16:26      15170000 -- (-7552.160) [...7 remote chains...] -- 1:16:22      15180000 -- (-7569.652) [...7 remote chains...] -- 1:16:19      15190000 -- (-7549.738) [...7 remote chains...] -- 1:16:16      15200000 -- (-7546.097) [...7 remote chains...] -- 1:16:14       Average standard deviation of split frequencies: 0.009822       15210000 -- (-7539.602) [...7 remote chains...] -- 1:16:11      15220000 -- (-7550.131) [...7 remote chains...] -- 1:16:08      15230000 -- (-7534.165) [...7 remote chains...] -- 1:16:04      15240000 -- (-7557.267) [...7 remote chains...] -- 1:16:01      15250000 -- (-7564.395) [...7 remote chains...] -- 1:15:58       Average standard deviation of split frequencies: 0.009468       15260000 -- (-7568.495) [...7 remote chains...] -- 1:15:55      15270000 -- (-7571.865) [...7 remote chains...] -- 1:15:52      15280000 -- (-7564.061) [...7 remote chains...] -- 1:15:48      15290000 -- (-7550.347) [...7 remote chains...] -- 1:15:45      15300000 -- (-7552.668) [...7 remote chains...] -- 1:15:42       Average standard deviation of split frequencies: 0.008971       15310000 -- (-7554.788) [...7 remote chains...] -- 1:15:39      15320000 -- (-7575.392) [...7 remote chains...] -- 1:15:36      15330000 -- [-7573.059] [...7 remote chains...] -- 1:15:33      15340000 -- (-7558.436) [...7 remote chains...] -- 1:15:29      15350000 -- (-7568.265) [...7 remote chains...] -- 1:15:26       Average standard deviation of split frequencies: 0.008765       15360000 -- (-7536.115) [...7 remote chains...] -- 1:15:23      15370000 -- (-7564.746) [...7 remote chains...] -- 1:15:20      15380000 -- (-7560.523) [...7 remote chains...] -- 1:15:17      15390000 -- (-7554.473) [...7 remote chains...] -- 1:15:14      15400000 -- (-7555.569) [...7 remote chains...] -- 1:15:10       Average standard deviation of split frequencies: 0.008487       15410000 -- (-7555.944) [...7 remote chains...] -- 1:15:07      15420000 -- (-7556.938) [...7 remote chains...] -- 1:15:05      15430000 -- [-7538.665] [...7 remote chains...] -- 1:15:02      15440000 -- [-7553.603] [...7 remote chains...] -- 1:14:59      15450000 -- [-7545.121] [...7 remote chains...] -- 1:14:55       Average standard deviation of split frequencies: 0.008547       15460000 -- (-7564.199) [...7 remote chains...] -- 1:14:52      15470000 -- [-7549.874] [...7 remote chains...] -- 1:14:49      15480000 -- (-7559.247) [...7 remote chains...] -- 1:14:46      15490000 -- (-7546.197) [...7 remote chains...] -- 1:14:43      15500000 -- (-7548.364) [...7 remote chains...] -- 1:14:40       Average standard deviation of split frequencies: 0.008232       15510000 -- [-7550.567] [...7 remote chains...] -- 1:14:36      15520000 -- (-7566.577) [...7 remote chains...] -- 1:14:33      15530000 -- (-7553.037) [...7 remote chains...] -- 1:14:30      15540000 -- (-7529.383) [...7 remote chains...] -- 1:14:27      15550000 -- (-7550.188) [...7 remote chains...] -- 1:14:24       Average standard deviation of split frequencies: 0.008331       15560000 -- (-7556.841) [...7 remote chains...] -- 1:14:20      15570000 -- [-7546.040] [...7 remote chains...] -- 1:14:17      15580000 -- [-7546.708] [...7 remote chains...] -- 1:14:14      15590000 -- [-7552.832] [...7 remote chains...] -- 1:14:11      15600000 -- [-7540.282] [...7 remote chains...] -- 1:14:08       Average standard deviation of split frequencies: 0.008308       15610000 -- (-7561.035) [...7 remote chains...] -- 1:14:05      15620000 -- (-7559.288) [...7 remote chains...] -- 1:14:01      15630000 -- (-7577.148) [...7 remote chains...] -- 1:13:58      15640000 -- (-7544.265) [...7 remote chains...] -- 1:13:55      15650000 -- (-7575.791) [...7 remote chains...] -- 1:13:52       Average standard deviation of split frequencies: 0.008275       15660000 -- (-7589.633) [...7 remote chains...] -- 1:13:49      15670000 -- (-7552.198) [...7 remote chains...] -- 1:13:46      15680000 -- (-7573.923) [...7 remote chains...] -- 1:13:42      15690000 -- (-7556.205) [...7 remote chains...] -- 1:13:39      15700000 -- (-7541.522) [...7 remote chains...] -- 1:13:36       Average standard deviation of split frequencies: 0.007920       15710000 -- (-7552.449) [...7 remote chains...] -- 1:13:34      15720000 -- (-7555.212) [...7 remote chains...] -- 1:13:31      15730000 -- [-7561.763] [...7 remote chains...] -- 1:13:28      15740000 -- [-7553.068] [...7 remote chains...] -- 1:13:24      15750000 -- [-7552.964] [...7 remote chains...] -- 1:13:21       Average standard deviation of split frequencies: 0.007809       15760000 -- (-7567.184) [...7 remote chains...] -- 1:13:18      15770000 -- (-7572.592) [...7 remote chains...] -- 1:13:15      15780000 -- (-7552.862) [...7 remote chains...] -- 1:13:12      15790000 -- [-7555.556] [...7 remote chains...] -- 1:13:08      15800000 -- (-7567.917) [...7 remote chains...] -- 1:13:05       Average standard deviation of split frequencies: 0.007819       15810000 -- (-7543.071) [...7 remote chains...] -- 1:13:02      15820000 -- (-7536.645) [...7 remote chains...] -- 1:12:59      15830000 -- (-7553.150) [...7 remote chains...] -- 1:12:56      15840000 -- (-7555.569) [...7 remote chains...] -- 1:12:53      15850000 -- (-7555.041) [...7 remote chains...] -- 1:12:49       Average standard deviation of split frequencies: 0.007927       15860000 -- (-7543.119) [...7 remote chains...] -- 1:12:46      15870000 -- [-7538.052] [...7 remote chains...] -- 1:12:43      15880000 -- (-7555.050) [...7 remote chains...] -- 1:12:40      15890000 -- (-7542.723) [...7 remote chains...] -- 1:12:37      15900000 -- (-7558.270) [...7 remote chains...] -- 1:12:34       Average standard deviation of split frequencies: 0.007952       15910000 -- (-7539.986) [...7 remote chains...] -- 1:12:30      15920000 -- (-7547.049) [...7 remote chains...] -- 1:12:27      15930000 -- (-7545.525) [...7 remote chains...] -- 1:12:24      15940000 -- (-7576.578) [...7 remote chains...] -- 1:12:21      15950000 -- (-7569.532) [...7 remote chains...] -- 1:12:18       Average standard deviation of split frequencies: 0.007923       15960000 -- (-7566.532) [...7 remote chains...] -- 1:12:15      15970000 -- (-7556.831) [...7 remote chains...] -- 1:12:11      15980000 -- (-7550.234) [...7 remote chains...] -- 1:12:08      15990000 -- (-7537.945) [...7 remote chains...] -- 1:12:06      16000000 -- (-7572.491) [...7 remote chains...] -- 1:12:03       Average standard deviation of split frequencies: 0.007903       16010000 -- (-7555.233) [...7 remote chains...] -- 1:12:00      16020000 -- (-7538.691) [...7 remote chains...] -- 1:11:57      16030000 -- (-7550.573) [...7 remote chains...] -- 1:11:53      16040000 -- (-7573.223) [...7 remote chains...] -- 1:11:50      16050000 -- (-7568.407) [...7 remote chains...] -- 1:11:46       Average standard deviation of split frequencies: 0.007812       16060000 -- (-7581.546) [...7 remote chains...] -- 1:11:43      16070000 -- (-7562.584) [...7 remote chains...] -- 1:11:41      16080000 -- (-7554.909) [...7 remote chains...] -- 1:11:38      16090000 -- (-7543.890) [...7 remote chains...] -- 1:11:34      16100000 -- (-7565.041) [...7 remote chains...] -- 1:11:31       Average standard deviation of split frequencies: 0.007788       16110000 -- (-7583.091) [...7 remote chains...] -- 1:11:28      16120000 -- (-7569.565) [...7 remote chains...] -- 1:11:25      16130000 -- (-7563.401) [...7 remote chains...] -- 1:11:22      16140000 -- (-7541.042) [...7 remote chains...] -- 1:11:19      16150000 -- (-7553.405) [...7 remote chains...] -- 1:11:15       Average standard deviation of split frequencies: 0.007773       16160000 -- (-7561.370) [...7 remote chains...] -- 1:11:12      16170000 -- (-7598.192) [...7 remote chains...] -- 1:11:09      16180000 -- (-7560.423) [...7 remote chains...] -- 1:11:06      16190000 -- (-7548.365) [...7 remote chains...] -- 1:11:03      16200000 -- (-7564.204) [...7 remote chains...] -- 1:11:00       Average standard deviation of split frequencies: 0.007563       16210000 -- (-7558.198) [...7 remote chains...] -- 1:10:56      16220000 -- (-7551.032) [...7 remote chains...] -- 1:10:53      16230000 -- (-7555.181) [...7 remote chains...] -- 1:10:50      16240000 -- (-7558.465) [...7 remote chains...] -- 1:10:47      16250000 -- (-7554.059) [...7 remote chains...] -- 1:10:44       Average standard deviation of split frequencies: 0.007433       16260000 -- [-7544.342] [...7 remote chains...] -- 1:10:41      16270000 -- [-7554.103] [...7 remote chains...] -- 1:10:37      16280000 -- (-7548.413) [...7 remote chains...] -- 1:10:34      16290000 -- (-7567.934) [...7 remote chains...] -- 1:10:31      16300000 -- (-7559.092) [...7 remote chains...] -- 1:10:28       Average standard deviation of split frequencies: 0.007392       16310000 -- (-7541.630) [...7 remote chains...] -- 1:10:25      16320000 -- (-7542.075) [...7 remote chains...] -- 1:10:22      16330000 -- (-7549.646) [...7 remote chains...] -- 1:10:19      16340000 -- (-7567.129) [...7 remote chains...] -- 1:10:16      16350000 -- (-7549.856) [...7 remote chains...] -- 1:10:13       Average standard deviation of split frequencies: 0.007251       16360000 -- (-7570.134) [...7 remote chains...] -- 1:10:10      16370000 -- (-7553.853) [...7 remote chains...] -- 1:10:07      16380000 -- (-7558.566) [...7 remote chains...] -- 1:10:04      16390000 -- (-7571.721) [...7 remote chains...] -- 1:10:00      16400000 -- [-7539.958] [...7 remote chains...] -- 1:09:57       Average standard deviation of split frequencies: 0.007173       16410000 -- (-7537.788) [...7 remote chains...] -- 1:09:54      16420000 -- (-7553.159) [...7 remote chains...] -- 1:09:51      16430000 -- [-7539.503] [...7 remote chains...] -- 1:09:48      16440000 -- [-7564.169] [...7 remote chains...] -- 1:09:45      16450000 -- [-7539.289] [...7 remote chains...] -- 1:09:41       Average standard deviation of split frequencies: 0.007006       16460000 -- [-7535.148] [...7 remote chains...] -- 1:09:38      16470000 -- [-7543.493] [...7 remote chains...] -- 1:09:35      16480000 -- (-7548.525) [...7 remote chains...] -- 1:09:32      16490000 -- (-7575.531) [...7 remote chains...] -- 1:09:29      16500000 -- (-7570.172) [...7 remote chains...] -- 1:09:26       Average standard deviation of split frequencies: 0.006885       16510000 -- (-7570.649) [...7 remote chains...] -- 1:09:23      16520000 -- (-7564.782) [...7 remote chains...] -- 1:09:19      16530000 -- (-7572.338) [...7 remote chains...] -- 1:09:16      16540000 -- (-7540.188) [...7 remote chains...] -- 1:09:13      16550000 -- (-7545.814) [...7 remote chains...] -- 1:09:10       Average standard deviation of split frequencies: 0.006916       16560000 -- (-7547.324) [...7 remote chains...] -- 1:09:07      16570000 -- (-7556.511) [...7 remote chains...] -- 1:09:04      16580000 -- (-7562.501) [...7 remote chains...] -- 1:09:00      16590000 -- (-7561.738) [...7 remote chains...] -- 1:08:57      16600000 -- (-7546.211) [...7 remote chains...] -- 1:08:54       Average standard deviation of split frequencies: 0.006947       16610000 -- (-7551.462) [...7 remote chains...] -- 1:08:51      16620000 -- (-7558.799) [...7 remote chains...] -- 1:08:48      16630000 -- (-7553.925) [...7 remote chains...] -- 1:08:45      16640000 -- (-7571.421) [...7 remote chains...] -- 1:08:42      16650000 -- (-7554.608) [...7 remote chains...] -- 1:08:38       Average standard deviation of split frequencies: 0.007003       16660000 -- (-7554.231) [...7 remote chains...] -- 1:08:35      16670000 -- (-7565.613) [...7 remote chains...] -- 1:08:32      16680000 -- (-7569.804) [...7 remote chains...] -- 1:08:29      16690000 -- (-7540.116) [...7 remote chains...] -- 1:08:26      16700000 -- (-7561.297) [...7 remote chains...] -- 1:08:23       Average standard deviation of split frequencies: 0.007088       16710000 -- (-7553.887) [...7 remote chains...] -- 1:08:19      16720000 -- [-7547.857] [...7 remote chains...] -- 1:08:17      16730000 -- (-7559.520) [...7 remote chains...] -- 1:08:14      16740000 -- (-7565.688) [...7 remote chains...] -- 1:08:11      16750000 -- (-7569.701) [...7 remote chains...] -- 1:08:08       Average standard deviation of split frequencies: 0.007016       16760000 -- (-7553.797) [...7 remote chains...] -- 1:08:04      16770000 -- (-7546.424) [...7 remote chains...] -- 1:08:01      16780000 -- (-7561.765) [...7 remote chains...] -- 1:07:58      16790000 -- (-7554.355) [...7 remote chains...] -- 1:07:55      16800000 -- (-7581.174) [...7 remote chains...] -- 1:07:52       Average standard deviation of split frequencies: 0.007110       16810000 -- (-7574.531) [...7 remote chains...] -- 1:07:49      16820000 -- (-7572.471) [...7 remote chains...] -- 1:07:46      16830000 -- (-7561.367) [...7 remote chains...] -- 1:07:42      16840000 -- (-7547.335) [...7 remote chains...] -- 1:07:39      16850000 -- (-7555.317) [...7 remote chains...] -- 1:07:36       Average standard deviation of split frequencies: 0.007143       16860000 -- (-7549.895) [...7 remote chains...] -- 1:07:33      16870000 -- (-7553.427) [...7 remote chains...] -- 1:07:30      16880000 -- [-7558.138] [...7 remote chains...] -- 1:07:27      16890000 -- [-7542.384] [...7 remote chains...] -- 1:07:23      16900000 -- [-7562.163] [...7 remote chains...] -- 1:07:20       Average standard deviation of split frequencies: 0.007116       16910000 -- [-7554.147] [...7 remote chains...] -- 1:07:18      16920000 -- (-7561.958) [...7 remote chains...] -- 1:07:15      16930000 -- (-7564.995) [...7 remote chains...] -- 1:07:12      16940000 -- [-7549.343] [...7 remote chains...] -- 1:07:09      16950000 -- [-7565.652] [...7 remote chains...] -- 1:07:05       Average standard deviation of split frequencies: 0.007139       16960000 -- [-7556.268] [...7 remote chains...] -- 1:07:02      16970000 -- (-7566.120) [...7 remote chains...] -- 1:06:59      16980000 -- (-7570.406) [...7 remote chains...] -- 1:06:56      16990000 -- (-7575.214) [...7 remote chains...] -- 1:06:53      17000000 -- (-7559.249) [...7 remote chains...] -- 1:06:50       Average standard deviation of split frequencies: 0.006966       17010000 -- [-7547.359] [...7 remote chains...] -- 1:06:46      17020000 -- [-7540.857] [...7 remote chains...] -- 1:06:43      17030000 -- [-7538.062] [...7 remote chains...] -- 1:06:40      17040000 -- (-7551.872) [...7 remote chains...] -- 1:06:37      17050000 -- (-7557.581) [...7 remote chains...] -- 1:06:34       Average standard deviation of split frequencies: 0.007160       17060000 -- (-7542.218) [...7 remote chains...] -- 1:06:31      17070000 -- (-7543.962) [...7 remote chains...] -- 1:06:28      17080000 -- (-7549.874) [...7 remote chains...] -- 1:06:24      17090000 -- (-7555.049) [...7 remote chains...] -- 1:06:21      17100000 -- (-7569.200) [...7 remote chains...] -- 1:06:18       Average standard deviation of split frequencies: 0.007224       17110000 -- (-7566.862) [...7 remote chains...] -- 1:06:15      17120000 -- (-7551.569) [...7 remote chains...] -- 1:06:12      17130000 -- (-7553.741) [...7 remote chains...] -- 1:06:09      17140000 -- (-7548.276) [...7 remote chains...] -- 1:06:06      17150000 -- (-7574.563) [...7 remote chains...] -- 1:06:02       Average standard deviation of split frequencies: 0.007061       17160000 -- (-7567.771) [...7 remote chains...] -- 1:05:59      17170000 -- (-7575.992) [...7 remote chains...] -- 1:05:56      17180000 -- (-7554.724) [...7 remote chains...] -- 1:05:54      17190000 -- (-7574.877) [...7 remote chains...] -- 1:05:50      17200000 -- (-7542.672) [...7 remote chains...] -- 1:05:47       Average standard deviation of split frequencies: 0.006805       17210000 -- (-7553.670) [...7 remote chains...] -- 1:05:44      17220000 -- (-7562.973) [...7 remote chains...] -- 1:05:41      17230000 -- (-7568.934) [...7 remote chains...] -- 1:05:38      17240000 -- (-7551.895) [...7 remote chains...] -- 1:05:35      17250000 -- (-7562.932) [...7 remote chains...] -- 1:05:32       Average standard deviation of split frequencies: 0.006829       17260000 -- (-7558.051) [...7 remote chains...] -- 1:05:29      17270000 -- (-7556.869) [...7 remote chains...] -- 1:05:25      17280000 -- (-7552.047) [...7 remote chains...] -- 1:05:22      17290000 -- (-7554.716) [...7 remote chains...] -- 1:05:19      17300000 -- (-7572.177) [...7 remote chains...] -- 1:05:16       Average standard deviation of split frequencies: 0.006848       17310000 -- (-7552.843) [...7 remote chains...] -- 1:05:13      17320000 -- (-7555.298) [...7 remote chains...] -- 1:05:10      17330000 -- (-7544.609) [...7 remote chains...] -- 1:05:07      17340000 -- (-7535.373) [...7 remote chains...] -- 1:05:03      17350000 -- (-7564.435) [...7 remote chains...] -- 1:05:00       Average standard deviation of split frequencies: 0.006835       17360000 -- (-7578.449) [...7 remote chains...] -- 1:04:57      17370000 -- (-7552.829) [...7 remote chains...] -- 1:04:54      17380000 -- (-7566.025) [...7 remote chains...] -- 1:04:51      17390000 -- (-7553.450) [...7 remote chains...] -- 1:04:48      17400000 -- (-7576.644) [...7 remote chains...] -- 1:04:45       Average standard deviation of split frequencies: 0.006861       17410000 -- (-7574.295) [...7 remote chains...] -- 1:04:42      17420000 -- (-7558.973) [...7 remote chains...] -- 1:04:39      17430000 -- (-7550.817) [...7 remote chains...] -- 1:04:36      17440000 -- (-7562.801) [...7 remote chains...] -- 1:04:33      17450000 -- (-7582.038) [...7 remote chains...] -- 1:04:30       Average standard deviation of split frequencies: 0.007022       17460000 -- (-7575.872) [...7 remote chains...] -- 1:04:26      17470000 -- (-7543.892) [...7 remote chains...] -- 1:04:23      17480000 -- (-7552.231) [...7 remote chains...] -- 1:04:20      17490000 -- (-7552.338) [...7 remote chains...] -- 1:04:17      17500000 -- (-7551.343) [...7 remote chains...] -- 1:04:14       Average standard deviation of split frequencies: 0.007166       17510000 -- (-7551.288) [...7 remote chains...] -- 1:04:11      17520000 -- (-7556.667) [...7 remote chains...] -- 1:04:07      17530000 -- (-7550.967) [...7 remote chains...] -- 1:04:04      17540000 -- (-7581.229) [...7 remote chains...] -- 1:04:01      17550000 -- (-7562.784) [...7 remote chains...] -- 1:03:58       Average standard deviation of split frequencies: 0.007226       17560000 -- (-7553.586) [...7 remote chains...] -- 1:03:55      17570000 -- (-7593.920) [...7 remote chains...] -- 1:03:52      17580000 -- (-7562.722) [...7 remote chains...] -- 1:03:49      17590000 -- (-7573.371) [...7 remote chains...] -- 1:03:46      17600000 -- (-7560.537) [...7 remote chains...] -- 1:03:42       Average standard deviation of split frequencies: 0.007254       17610000 -- (-7556.857) [...7 remote chains...] -- 1:03:39      17620000 -- (-7570.940) [...7 remote chains...] -- 1:03:36      17630000 -- (-7583.360) [...7 remote chains...] -- 1:03:33      17640000 -- (-7551.193) [...7 remote chains...] -- 1:03:30      17650000 -- (-7542.526) [...7 remote chains...] -- 1:03:27       Average standard deviation of split frequencies: 0.007381       17660000 -- (-7580.047) [...7 remote chains...] -- 1:03:24      17670000 -- (-7568.109) [...7 remote chains...] -- 1:03:20      17680000 -- [-7559.742] [...7 remote chains...] -- 1:03:17      17690000 -- (-7546.858) [...7 remote chains...] -- 1:03:14      17700000 -- (-7564.493) [...7 remote chains...] -- 1:03:11       Average standard deviation of split frequencies: 0.007554       17710000 -- (-7549.505) [...7 remote chains...] -- 1:03:08      17720000 -- [-7561.743] [...7 remote chains...] -- 1:03:05      17730000 -- [-7540.520] [...7 remote chains...] -- 1:03:02      17740000 -- (-7536.516) [...7 remote chains...] -- 1:02:58      17750000 -- (-7537.033) [...7 remote chains...] -- 1:02:55       Average standard deviation of split frequencies: 0.007629       17760000 -- (-7552.779) [...7 remote chains...] -- 1:02:52      17770000 -- (-7538.847) [...7 remote chains...] -- 1:02:49      17780000 -- (-7575.449) [...7 remote chains...] -- 1:02:46      17790000 -- (-7556.529) [...7 remote chains...] -- 1:02:43      17800000 -- (-7556.923) [...7 remote chains...] -- 1:02:40       Average standard deviation of split frequencies: 0.007618       17810000 -- (-7561.480) [...7 remote chains...] -- 1:02:36      17820000 -- (-7546.072) [...7 remote chains...] -- 1:02:34      17830000 -- (-7529.713) [...7 remote chains...] -- 1:02:31      17840000 -- (-7541.115) [...7 remote chains...] -- 1:02:28      17850000 -- [-7524.285] [...7 remote chains...] -- 1:02:25       Average standard deviation of split frequencies: 0.007546       17860000 -- (-7546.790) [...7 remote chains...] -- 1:02:21      17870000 -- (-7553.367) [...7 remote chains...] -- 1:02:18      17880000 -- (-7546.707) [...7 remote chains...] -- 1:02:15      17890000 -- (-7568.906) [...7 remote chains...] -- 1:02:12      17900000 -- (-7579.145) [...7 remote chains...] -- 1:02:09       Average standard deviation of split frequencies: 0.007365       17910000 -- (-7553.238) [...7 remote chains...] -- 1:02:06      17920000 -- (-7538.465) [...7 remote chains...] -- 1:02:03      17930000 -- (-7555.192) [...7 remote chains...] -- 1:01:59      17940000 -- [-7566.032] [...7 remote chains...] -- 1:01:56      17950000 -- (-7575.826) [...7 remote chains...] -- 1:01:53       Average standard deviation of split frequencies: 0.007153       17960000 -- (-7579.599) [...7 remote chains...] -- 1:01:50      17970000 -- (-7571.969) [...7 remote chains...] -- 1:01:47      17980000 -- (-7539.524) [...7 remote chains...] -- 1:01:44      17990000 -- (-7566.597) [...7 remote chains...] -- 1:01:41      18000000 -- (-7551.494) [...7 remote chains...] -- 1:01:38       Average standard deviation of split frequencies: 0.007216       18010000 -- (-7555.211) [...7 remote chains...] -- 1:01:34      18020000 -- (-7564.689) [...7 remote chains...] -- 1:01:31      18030000 -- (-7549.565) [...7 remote chains...] -- 1:01:28      18040000 -- (-7572.368) [...7 remote chains...] -- 1:01:25      18050000 -- (-7566.981) [...7 remote chains...] -- 1:01:22       Average standard deviation of split frequencies: 0.007255       18060000 -- (-7557.889) [...7 remote chains...] -- 1:01:19      18070000 -- (-7569.310) [...7 remote chains...] -- 1:01:16      18080000 -- (-7571.229) [...7 remote chains...] -- 1:01:12      18090000 -- (-7545.212) [...7 remote chains...] -- 1:01:09      18100000 -- (-7561.080) [...7 remote chains...] -- 1:01:06       Average standard deviation of split frequencies: 0.007091       18110000 -- [-7552.787] [...7 remote chains...] -- 1:01:03      18120000 -- [-7544.671] [...7 remote chains...] -- 1:01:00      18130000 -- [-7523.162] [...7 remote chains...] -- 1:00:57      18140000 -- (-7542.445) [...7 remote chains...] -- 1:00:54      18150000 -- (-7565.444) [...7 remote chains...] -- 1:00:50       Average standard deviation of split frequencies: 0.006864       18160000 -- (-7553.473) [...7 remote chains...] -- 1:00:47      18170000 -- (-7562.289) [...7 remote chains...] -- 1:00:44      18180000 -- (-7551.725) [...7 remote chains...] -- 1:00:41      18190000 -- (-7550.324) [...7 remote chains...] -- 1:00:38      18200000 -- (-7547.008) [...7 remote chains...] -- 1:00:35       Average standard deviation of split frequencies: 0.006905       18210000 -- (-7553.574) [...7 remote chains...] -- 1:00:32      18220000 -- (-7573.325) [...7 remote chains...] -- 1:00:29      18230000 -- (-7554.694) [...7 remote chains...] -- 1:00:25      18240000 -- (-7555.826) [...7 remote chains...] -- 1:00:22      18250000 -- [-7540.929] [...7 remote chains...] -- 1:00:19       Average standard deviation of split frequencies: 0.006928       18260000 -- [-7556.011] [...7 remote chains...] -- 1:00:16      18270000 -- (-7561.715) [...7 remote chains...] -- 1:00:13      18280000 -- [-7522.535] [...7 remote chains...] -- 1:00:10      18290000 -- (-7551.410) [...7 remote chains...] -- 1:00:07      18300000 -- (-7557.995) [...7 remote chains...] -- 1:00:03       Average standard deviation of split frequencies: 0.007011       18310000 -- (-7583.174) [...7 remote chains...] -- 1:00:00      18320000 -- (-7567.727) [...7 remote chains...] -- 0:59:57      18330000 -- (-7560.059) [...7 remote chains...] -- 0:59:54      18340000 -- (-7552.020) [...7 remote chains...] -- 0:59:51      18350000 -- (-7552.971) [...7 remote chains...] -- 0:59:48       Average standard deviation of split frequencies: 0.007011       18360000 -- [-7555.176] [...7 remote chains...] -- 0:59:45      18370000 -- [-7544.686] [...7 remote chains...] -- 0:59:42      18380000 -- [-7553.008] [...7 remote chains...] -- 0:59:39      18390000 -- [-7537.031] [...7 remote chains...] -- 0:59:36      18400000 -- [-7542.497] [...7 remote chains...] -- 0:59:32       Average standard deviation of split frequencies: 0.007011       18410000 -- [-7549.021] [...7 remote chains...] -- 0:59:29      18420000 -- [-7539.660] [...7 remote chains...] -- 0:59:26      18430000 -- [-7532.201] [...7 remote chains...] -- 0:59:23      18440000 -- [-7536.977] [...7 remote chains...] -- 0:59:20      18450000 -- [-7555.909] [...7 remote chains...] -- 0:59:17       Average standard deviation of split frequencies: 0.007051       18460000 -- [-7563.012] [...7 remote chains...] -- 0:59:13      18470000 -- (-7551.136) [...7 remote chains...] -- 0:59:10      18480000 -- (-7550.458) [...7 remote chains...] -- 0:59:07      18490000 -- [-7536.101] [...7 remote chains...] -- 0:59:04      18500000 -- [-7541.998] [...7 remote chains...] -- 0:59:01       Average standard deviation of split frequencies: 0.007032       18510000 -- [-7536.017] [...7 remote chains...] -- 0:58:58      18520000 -- [-7543.717] [...7 remote chains...] -- 0:58:55      18530000 -- (-7556.801) [...7 remote chains...] -- 0:58:52      18540000 -- (-7562.346) [...7 remote chains...] -- 0:58:49      18550000 -- (-7550.150) [...7 remote chains...] -- 0:58:46       Average standard deviation of split frequencies: 0.007006       18560000 -- (-7538.950) [...7 remote chains...] -- 0:58:43      18570000 -- (-7564.488) [...7 remote chains...] -- 0:58:40      18580000 -- (-7548.253) [...7 remote chains...] -- 0:58:36      18590000 -- (-7559.359) [...7 remote chains...] -- 0:58:33      18600000 -- (-7545.719) [...7 remote chains...] -- 0:58:30       Average standard deviation of split frequencies: 0.007030       18610000 -- (-7579.711) [...7 remote chains...] -- 0:58:27      18620000 -- (-7556.083) [...7 remote chains...] -- 0:58:24      18630000 -- (-7569.519) [...7 remote chains...] -- 0:58:21      18640000 -- (-7531.458) [...7 remote chains...] -- 0:58:18      18650000 -- (-7564.178) [...7 remote chains...] -- 0:58:15       Average standard deviation of split frequencies: 0.007162       18660000 -- (-7556.900) [...7 remote chains...] -- 0:58:11      18670000 -- (-7545.314) [...7 remote chains...] -- 0:58:08      18680000 -- (-7565.386) [...7 remote chains...] -- 0:58:05      18690000 -- (-7546.808) [...7 remote chains...] -- 0:58:02      18700000 -- (-7550.777) [...7 remote chains...] -- 0:57:59       Average standard deviation of split frequencies: 0.007165       18710000 -- (-7550.009) [...7 remote chains...] -- 0:57:56      18720000 -- [-7548.953] [...7 remote chains...] -- 0:57:53      18730000 -- [-7540.216] [...7 remote chains...] -- 0:57:50      18740000 -- (-7552.328) [...7 remote chains...] -- 0:57:46      18750000 -- [-7549.796] [...7 remote chains...] -- 0:57:43       Average standard deviation of split frequencies: 0.007160       18760000 -- (-7534.996) [...7 remote chains...] -- 0:57:41      18770000 -- (-7547.391) [...7 remote chains...] -- 0:57:38      18780000 -- (-7557.582) [...7 remote chains...] -- 0:57:35      18790000 -- (-7558.990) [...7 remote chains...] -- 0:57:31      18800000 -- [-7552.625] [...7 remote chains...] -- 0:57:28       Average standard deviation of split frequencies: 0.007134       18810000 -- (-7568.114) [...7 remote chains...] -- 0:57:25      18820000 -- [-7549.589] [...7 remote chains...] -- 0:57:22      18830000 -- [-7558.924] [...7 remote chains...] -- 0:57:19      18840000 -- (-7547.307) [...7 remote chains...] -- 0:57:16      18850000 -- [-7544.846] [...7 remote chains...] -- 0:57:13       Average standard deviation of split frequencies: 0.007041       18860000 -- [-7551.202] [...7 remote chains...] -- 0:57:10      18870000 -- (-7559.481) [...7 remote chains...] -- 0:57:06      18880000 -- [-7556.356] [...7 remote chains...] -- 0:57:03      18890000 -- [-7540.664] [...7 remote chains...] -- 0:57:00      18900000 -- (-7550.623) [...7 remote chains...] -- 0:56:57       Average standard deviation of split frequencies: 0.007045       18910000 -- (-7527.936) [...7 remote chains...] -- 0:56:54      18920000 -- [-7556.641] [...7 remote chains...] -- 0:56:51      18930000 -- (-7563.287) [...7 remote chains...] -- 0:56:48      18940000 -- (-7556.845) [...7 remote chains...] -- 0:56:45      18950000 -- (-7565.809) [...7 remote chains...] -- 0:56:41       Average standard deviation of split frequencies: 0.007122       18960000 -- (-7534.407) [...7 remote chains...] -- 0:56:38      18970000 -- (-7563.134) [...7 remote chains...] -- 0:56:35      18980000 -- [-7528.468] [...7 remote chains...] -- 0:56:32      18990000 -- (-7548.623) [...7 remote chains...] -- 0:56:29      19000000 -- [-7540.174] [...7 remote chains...] -- 0:56:26       Average standard deviation of split frequencies: 0.007074       19010000 -- [-7552.229] [...7 remote chains...] -- 0:56:23      19020000 -- [-7558.209] [...7 remote chains...] -- 0:56:20      19030000 -- [-7537.266] [...7 remote chains...] -- 0:56:16      19040000 -- (-7535.985) [...7 remote chains...] -- 0:56:13      19050000 -- [-7550.622] [...7 remote chains...] -- 0:56:11       Average standard deviation of split frequencies: 0.007010       19060000 -- [-7550.035] [...7 remote chains...] -- 0:56:08      19070000 -- [-7545.588] [...7 remote chains...] -- 0:56:04      19080000 -- [-7557.080] [...7 remote chains...] -- 0:56:01      19090000 -- [-7551.509] [...7 remote chains...] -- 0:55:58      19100000 -- [-7539.577] [...7 remote chains...] -- 0:55:55       Average standard deviation of split frequencies: 0.007268       19110000 -- [-7534.634] [...7 remote chains...] -- 0:55:52      19120000 -- [-7545.113] [...7 remote chains...] -- 0:55:49      19130000 -- [-7570.099] [...7 remote chains...] -- 0:55:46      19140000 -- [-7550.981] [...7 remote chains...] -- 0:55:43      19150000 -- (-7573.383) [...7 remote chains...] -- 0:55:39       Average standard deviation of split frequencies: 0.007263       19160000 -- (-7549.996) [...7 remote chains...] -- 0:55:36      19170000 -- [-7546.680] [...7 remote chains...] -- 0:55:33      19180000 -- (-7542.595) [...7 remote chains...] -- 0:55:30      19190000 -- (-7553.952) [...7 remote chains...] -- 0:55:27      19200000 -- (-7572.051) [...7 remote chains...] -- 0:55:24       Average standard deviation of split frequencies: 0.007373       19210000 -- (-7536.717) [...7 remote chains...] -- 0:55:21      19220000 -- (-7541.141) [...7 remote chains...] -- 0:55:18      19230000 -- (-7561.723) [...7 remote chains...] -- 0:55:15      19240000 -- (-7541.184) [...7 remote chains...] -- 0:55:11      19250000 -- (-7576.467) [...7 remote chains...] -- 0:55:08       Average standard deviation of split frequencies: 0.007542       19260000 -- (-7565.788) [...7 remote chains...] -- 0:55:05      19270000 -- (-7570.223) [...7 remote chains...] -- 0:55:02      19280000 -- (-7551.993) [...7 remote chains...] -- 0:54:59      19290000 -- [-7548.015] [...7 remote chains...] -- 0:54:56      19300000 -- (-7567.809) [...7 remote chains...] -- 0:54:53       Average standard deviation of split frequencies: 0.007596       19310000 -- [-7537.514] [...7 remote chains...] -- 0:54:50      19320000 -- [-7544.942] [...7 remote chains...] -- 0:54:46      19330000 -- (-7544.898) [...7 remote chains...] -- 0:54:43      19340000 -- (-7578.306) [...7 remote chains...] -- 0:54:40      19350000 -- (-7542.781) [...7 remote chains...] -- 0:54:37       Average standard deviation of split frequencies: 0.007549       19360000 -- (-7542.867) [...7 remote chains...] -- 0:54:34      19370000 -- [-7543.249] [...7 remote chains...] -- 0:54:31      19380000 -- [-7557.318] [...7 remote chains...] -- 0:54:28      19390000 -- (-7544.359) [...7 remote chains...] -- 0:54:25      19400000 -- (-7572.958) [...7 remote chains...] -- 0:54:21       Average standard deviation of split frequencies: 0.007512       19410000 -- [-7542.357] [...7 remote chains...] -- 0:54:18      19420000 -- (-7543.106) [...7 remote chains...] -- 0:54:15      19430000 -- [-7539.632] [...7 remote chains...] -- 0:54:12      19440000 -- [-7551.751] [...7 remote chains...] -- 0:54:09      19450000 -- (-7561.151) [...7 remote chains...] -- 0:54:06       Average standard deviation of split frequencies: 0.007417       19460000 -- [-7554.962] [...7 remote chains...] -- 0:54:03      19470000 -- [-7550.021] [...7 remote chains...] -- 0:54:00      19480000 -- [-7556.309] [...7 remote chains...] -- 0:53:57      19490000 -- [-7537.106] [...7 remote chains...] -- 0:53:54      19500000 -- [-7520.729] [...7 remote chains...] -- 0:53:51       Average standard deviation of split frequencies: 0.007284       19510000 -- (-7548.505) [...7 remote chains...] -- 0:53:48      19520000 -- (-7583.136) [...7 remote chains...] -- 0:53:45      19530000 -- (-7561.273) [...7 remote chains...] -- 0:53:41      19540000 -- (-7551.918) [...7 remote chains...] -- 0:53:38      19550000 -- (-7550.782) [...7 remote chains...] -- 0:53:35       Average standard deviation of split frequencies: 0.007257       19560000 -- (-7532.083) [...7 remote chains...] -- 0:53:32      19570000 -- [-7554.673] [...7 remote chains...] -- 0:53:29      19580000 -- [-7565.882] [...7 remote chains...] -- 0:53:26      19590000 -- [-7556.735] [...7 remote chains...] -- 0:53:23      19600000 -- (-7540.312) [...7 remote chains...] -- 0:53:20       Average standard deviation of split frequencies: 0.007213       19610000 -- (-7545.350) [...7 remote chains...] -- 0:53:17      19620000 -- (-7545.211) [...7 remote chains...] -- 0:53:13      19630000 -- (-7556.977) [...7 remote chains...] -- 0:53:10      19640000 -- (-7556.343) [...7 remote chains...] -- 0:53:07      19650000 -- (-7562.919) [...7 remote chains...] -- 0:53:04       Average standard deviation of split frequencies: 0.007297       19660000 -- (-7545.546) [...7 remote chains...] -- 0:53:01      19670000 -- (-7550.299) [...7 remote chains...] -- 0:52:58      19680000 -- (-7547.506) [...7 remote chains...] -- 0:52:55      19690000 -- (-7566.697) [...7 remote chains...] -- 0:52:52      19700000 -- (-7566.920) [...7 remote chains...] -- 0:52:48       Average standard deviation of split frequencies: 0.007453       19710000 -- (-7565.086) [...7 remote chains...] -- 0:52:45      19720000 -- (-7558.578) [...7 remote chains...] -- 0:52:42      19730000 -- (-7550.279) [...7 remote chains...] -- 0:52:39      19740000 -- (-7569.182) [...7 remote chains...] -- 0:52:36      19750000 -- (-7568.824) [...7 remote chains...] -- 0:52:33       Average standard deviation of split frequencies: 0.007480       19760000 -- (-7564.069) [...7 remote chains...] -- 0:52:30      19770000 -- (-7578.878) [...7 remote chains...] -- 0:52:27      19780000 -- (-7577.802) [...7 remote chains...] -- 0:52:24      19790000 -- (-7565.708) [...7 remote chains...] -- 0:52:20      19800000 -- (-7543.544) [...7 remote chains...] -- 0:52:17       Average standard deviation of split frequencies: 0.007394       19810000 -- (-7551.874) [...7 remote chains...] -- 0:52:14      19820000 -- [-7546.103] [...7 remote chains...] -- 0:52:11      19830000 -- (-7567.967) [...7 remote chains...] -- 0:52:08      19840000 -- [-7544.611] [...7 remote chains...] -- 0:52:05      19850000 -- (-7547.987) [...7 remote chains...] -- 0:52:02       Average standard deviation of split frequencies: 0.007409       19860000 -- [-7549.453] [...7 remote chains...] -- 0:51:59      19870000 -- [-7546.389] [...7 remote chains...] -- 0:51:55      19880000 -- [-7542.138] [...7 remote chains...] -- 0:51:52      19890000 -- (-7546.098) [...7 remote chains...] -- 0:51:49      19900000 -- (-7556.494) [...7 remote chains...] -- 0:51:46       Average standard deviation of split frequencies: 0.007361       19910000 -- (-7546.043) [...7 remote chains...] -- 0:51:44      19920000 -- [-7558.399] [...7 remote chains...] -- 0:51:40      19930000 -- [-7543.943] [...7 remote chains...] -- 0:51:37      19940000 -- (-7549.038) [...7 remote chains...] -- 0:51:34      19950000 -- [-7552.931] [...7 remote chains...] -- 0:51:31       Average standard deviation of split frequencies: 0.007311       19960000 -- [-7560.270] [...7 remote chains...] -- 0:51:28      19970000 -- [-7552.805] [...7 remote chains...] -- 0:51:25      19980000 -- [-7535.914] [...7 remote chains...] -- 0:51:22      19990000 -- [-7551.572] [...7 remote chains...] -- 0:51:19      20000000 -- [-7561.639] [...7 remote chains...] -- 0:51:16       Average standard deviation of split frequencies: 0.007277       20010000 -- [-7563.018] [...7 remote chains...] -- 0:51:12      20020000 -- [-7544.916] [...7 remote chains...] -- 0:51:09      20030000 -- [-7564.984] [...7 remote chains...] -- 0:51:06      20040000 -- [-7572.519] [...7 remote chains...] -- 0:51:03      20050000 -- [-7563.827] [...7 remote chains...] -- 0:51:00       Average standard deviation of split frequencies: 0.007342       20060000 -- [-7567.210] [...7 remote chains...] -- 0:50:57      20070000 -- [-7562.884] [...7 remote chains...] -- 0:50:54      20080000 -- [-7563.903] [...7 remote chains...] -- 0:50:51      20090000 -- [-7562.964] [...7 remote chains...] -- 0:50:47      20100000 -- [-7541.406] [...7 remote chains...] -- 0:50:44       Average standard deviation of split frequencies: 0.007271       20110000 -- [-7542.905] [...7 remote chains...] -- 0:50:41      20120000 -- [-7544.532] [...7 remote chains...] -- 0:50:38      20130000 -- [-7557.649] [...7 remote chains...] -- 0:50:35      20140000 -- [-7544.689] [...7 remote chains...] -- 0:50:32      20150000 -- [-7550.586] [...7 remote chains...] -- 0:50:29       Average standard deviation of split frequencies: 0.007312       20160000 -- [-7566.326] [...7 remote chains...] -- 0:50:26      20170000 -- [-7549.635] [...7 remote chains...] -- 0:50:23      20180000 -- [-7550.010] [...7 remote chains...] -- 0:50:19      20190000 -- [-7550.644] [...7 remote chains...] -- 0:50:16      20200000 -- [-7552.720] [...7 remote chains...] -- 0:50:13       Average standard deviation of split frequencies: 0.007436       20210000 -- (-7549.681) [...7 remote chains...] -- 0:50:10      20220000 -- [-7555.182] [...7 remote chains...] -- 0:50:07      20230000 -- [-7562.770] [...7 remote chains...] -- 0:50:04      20240000 -- [-7545.746] [...7 remote chains...] -- 0:50:01      20250000 -- [-7553.183] [...7 remote chains...] -- 0:49:58       Average standard deviation of split frequencies: 0.007429       20260000 -- (-7543.088) [...7 remote chains...] -- 0:49:55      20270000 -- (-7560.804) [...7 remote chains...] -- 0:49:51      20280000 -- (-7541.981) [...7 remote chains...] -- 0:49:48      20290000 -- (-7559.315) [...7 remote chains...] -- 0:49:46      20300000 -- (-7552.034) [...7 remote chains...] -- 0:49:43       Average standard deviation of split frequencies: 0.007301       20310000 -- (-7554.661) [...7 remote chains...] -- 0:49:39      20320000 -- (-7539.549) [...7 remote chains...] -- 0:49:36      20330000 -- (-7537.925) [...7 remote chains...] -- 0:49:33      20340000 -- (-7554.810) [...7 remote chains...] -- 0:49:30      20350000 -- (-7566.896) [...7 remote chains...] -- 0:49:27       Average standard deviation of split frequencies: 0.007344       20360000 -- (-7544.290) [...7 remote chains...] -- 0:49:24      20370000 -- (-7560.268) [...7 remote chains...] -- 0:49:21      20380000 -- (-7563.925) [...7 remote chains...] -- 0:49:18      20390000 -- (-7570.235) [...7 remote chains...] -- 0:49:15      20400000 -- (-7549.171) [...7 remote chains...] -- 0:49:12       Average standard deviation of split frequencies: 0.007408       20410000 -- (-7564.345) [...7 remote chains...] -- 0:49:08      20420000 -- (-7568.872) [...7 remote chains...] -- 0:49:05      20430000 -- (-7572.803) [...7 remote chains...] -- 0:49:02      20440000 -- (-7570.140) [...7 remote chains...] -- 0:48:59      20450000 -- (-7591.022) [...7 remote chains...] -- 0:48:56       Average standard deviation of split frequencies: 0.007449       20460000 -- (-7570.653) [...7 remote chains...] -- 0:48:53      20470000 -- (-7591.233) [...7 remote chains...] -- 0:48:50      20480000 -- (-7553.779) [...7 remote chains...] -- 0:48:47      20490000 -- (-7559.417) [...7 remote chains...] -- 0:48:44      20500000 -- (-7576.301) [...7 remote chains...] -- 0:48:41       Average standard deviation of split frequencies: 0.007513       20510000 -- (-7559.178) [...7 remote chains...] -- 0:48:38      20520000 -- (-7570.933) [...7 remote chains...] -- 0:48:35      20530000 -- (-7582.819) [...7 remote chains...] -- 0:48:32      20540000 -- (-7562.148) [...7 remote chains...] -- 0:48:28      20550000 -- (-7561.733) [...7 remote chains...] -- 0:48:25       Average standard deviation of split frequencies: 0.007477       20560000 -- (-7573.337) [...7 remote chains...] -- 0:48:22      20570000 -- (-7579.240) [...7 remote chains...] -- 0:48:19      20580000 -- (-7580.124) [...7 remote chains...] -- 0:48:16      20590000 -- (-7562.720) [...7 remote chains...] -- 0:48:13      20600000 -- (-7558.404) [...7 remote chains...] -- 0:48:10       Average standard deviation of split frequencies: 0.007489       20610000 -- (-7575.511) [...7 remote chains...] -- 0:48:07      20620000 -- (-7572.356) [...7 remote chains...] -- 0:48:04      20630000 -- (-7570.845) [...7 remote chains...] -- 0:48:01      20640000 -- (-7567.531) [...7 remote chains...] -- 0:47:58      20650000 -- (-7561.021) [...7 remote chains...] -- 0:47:55       Average standard deviation of split frequencies: 0.007334       20660000 -- (-7575.378) [...7 remote chains...] -- 0:47:52      20670000 -- (-7548.745) [...7 remote chains...] -- 0:47:48      20680000 -- (-7560.057) [...7 remote chains...] -- 0:47:45      20690000 -- (-7556.209) [...7 remote chains...] -- 0:47:42      20700000 -- (-7562.424) [...7 remote chains...] -- 0:47:39       Average standard deviation of split frequencies: 0.007299       20710000 -- (-7550.791) [...7 remote chains...] -- 0:47:36      20720000 -- (-7559.242) [...7 remote chains...] -- 0:47:33      20730000 -- (-7573.666) [...7 remote chains...] -- 0:47:30      20740000 -- (-7559.065) [...7 remote chains...] -- 0:47:27      20750000 -- (-7553.984) [...7 remote chains...] -- 0:47:24       Average standard deviation of split frequencies: 0.007329       20760000 -- (-7549.359) [...7 remote chains...] -- 0:47:20      20770000 -- (-7567.011) [...7 remote chains...] -- 0:47:17      20780000 -- (-7551.986) [...7 remote chains...] -- 0:47:14      20790000 -- (-7557.688) [...7 remote chains...] -- 0:47:11      20800000 -- (-7574.977) [...7 remote chains...] -- 0:47:08       Average standard deviation of split frequencies: 0.007289       20810000 -- (-7561.977) [...7 remote chains...] -- 0:47:05      20820000 -- (-7548.979) [...7 remote chains...] -- 0:47:02      20830000 -- (-7541.475) [...7 remote chains...] -- 0:46:59      20840000 -- (-7568.280) [...7 remote chains...] -- 0:46:56      20850000 -- (-7555.131) [...7 remote chains...] -- 0:46:53       Average standard deviation of split frequencies: 0.007171       20860000 -- (-7579.369) [...7 remote chains...] -- 0:46:49      20870000 -- (-7560.811) [...7 remote chains...] -- 0:46:46      20880000 -- (-7569.513) [...7 remote chains...] -- 0:46:43      20890000 -- (-7563.080) [...7 remote chains...] -- 0:46:40      20900000 -- (-7547.883) [...7 remote chains...] -- 0:46:37       Average standard deviation of split frequencies: 0.007082       20910000 -- (-7557.666) [...7 remote chains...] -- 0:46:34      20920000 -- (-7570.417) [...7 remote chains...] -- 0:46:31      20930000 -- (-7565.315) [...7 remote chains...] -- 0:46:28      20940000 -- (-7529.418) [...7 remote chains...] -- 0:46:25      20950000 -- (-7552.406) [...7 remote chains...] -- 0:46:22       Average standard deviation of split frequencies: 0.007049       20960000 -- (-7568.977) [...7 remote chains...] -- 0:46:19      20970000 -- (-7557.992) [...7 remote chains...] -- 0:46:16      20980000 -- (-7573.239) [...7 remote chains...] -- 0:46:13      20990000 -- (-7552.730) [...7 remote chains...] -- 0:46:09      21000000 -- (-7558.913) [...7 remote chains...] -- 0:46:06       Average standard deviation of split frequencies: 0.007063       21010000 -- (-7552.254) [...7 remote chains...] -- 0:46:03      21020000 -- (-7549.028) [...7 remote chains...] -- 0:46:00      21030000 -- (-7568.799) [...7 remote chains...] -- 0:45:57      21040000 -- (-7569.420) [...7 remote chains...] -- 0:45:54      21050000 -- (-7553.233) [...7 remote chains...] -- 0:45:51       Average standard deviation of split frequencies: 0.007056       21060000 -- (-7556.580) [...7 remote chains...] -- 0:45:48      21070000 -- (-7557.360) [...7 remote chains...] -- 0:45:45      21080000 -- (-7552.030) [...7 remote chains...] -- 0:45:42      21090000 -- (-7528.015) [...7 remote chains...] -- 0:45:38      21100000 -- (-7547.330) [...7 remote chains...] -- 0:45:35       Average standard deviation of split frequencies: 0.007037       21110000 -- (-7537.835) [...7 remote chains...] -- 0:45:32      21120000 -- (-7551.739) [...7 remote chains...] -- 0:45:29      21130000 -- (-7572.925) [...7 remote chains...] -- 0:45:26      21140000 -- (-7566.085) [...7 remote chains...] -- 0:45:23      21150000 -- (-7587.020) [...7 remote chains...] -- 0:45:20       Average standard deviation of split frequencies: 0.006961       21160000 -- (-7577.627) [...7 remote chains...] -- 0:45:17      21170000 -- [-7546.352] [...7 remote chains...] -- 0:45:14      21180000 -- (-7559.457) [...7 remote chains...] -- 0:45:10      21190000 -- (-7562.142) [...7 remote chains...] -- 0:45:07      21200000 -- (-7554.321) [...7 remote chains...] -- 0:45:04       Average standard deviation of split frequencies: 0.006881       21210000 -- (-7563.516) [...7 remote chains...] -- 0:45:02      21220000 -- (-7554.770) [...7 remote chains...] -- 0:44:58      21230000 -- (-7548.658) [...7 remote chains...] -- 0:44:55      21240000 -- (-7562.411) [...7 remote chains...] -- 0:44:52      21250000 -- (-7582.220) [...7 remote chains...] -- 0:44:49       Average standard deviation of split frequencies: 0.006812       21260000 -- (-7552.077) [...7 remote chains...] -- 0:44:46      21270000 -- (-7572.729) [...7 remote chains...] -- 0:44:43      21280000 -- [-7553.105] [...7 remote chains...] -- 0:44:40      21290000 -- (-7539.166) [...7 remote chains...] -- 0:44:37      21300000 -- (-7547.378) [...7 remote chains...] -- 0:44:34       Average standard deviation of split frequencies: 0.006781       21310000 -- [-7532.849] [...7 remote chains...] -- 0:44:31      21320000 -- [-7549.582] [...7 remote chains...] -- 0:44:27      21330000 -- (-7543.402) [...7 remote chains...] -- 0:44:24      21340000 -- [-7551.631] [...7 remote chains...] -- 0:44:21      21350000 -- [-7558.204] [...7 remote chains...] -- 0:44:18       Average standard deviation of split frequencies: 0.006751       21360000 -- (-7559.468) [...7 remote chains...] -- 0:44:15      21370000 -- (-7556.388) [...7 remote chains...] -- 0:44:12      21380000 -- (-7545.753) [...7 remote chains...] -- 0:44:09      21390000 -- (-7546.663) [...7 remote chains...] -- 0:44:06      21400000 -- [-7538.448] [...7 remote chains...] -- 0:44:03       Average standard deviation of split frequencies: 0.006677       21410000 -- [-7550.954] [...7 remote chains...] -- 0:43:59      21420000 -- [-7553.516] [...7 remote chains...] -- 0:43:56      21430000 -- (-7540.143) [...7 remote chains...] -- 0:43:54      21440000 -- [-7549.976] [...7 remote chains...] -- 0:43:51      21450000 -- [-7545.337] [...7 remote chains...] -- 0:43:47       Average standard deviation of split frequencies: 0.006470       21460000 -- (-7540.071) [...7 remote chains...] -- 0:43:44      21470000 -- (-7557.497) [...7 remote chains...] -- 0:43:41      21480000 -- (-7556.726) [...7 remote chains...] -- 0:43:38      21490000 -- (-7556.922) [...7 remote chains...] -- 0:43:35      21500000 -- (-7555.452) [...7 remote chains...] -- 0:43:32       Average standard deviation of split frequencies: 0.006475       21510000 -- (-7534.110) [...7 remote chains...] -- 0:43:29      21520000 -- (-7528.528) [...7 remote chains...] -- 0:43:26      21530000 -- (-7559.604) [...7 remote chains...] -- 0:43:23      21540000 -- (-7545.577) [...7 remote chains...] -- 0:43:20      21550000 -- (-7542.727) [...7 remote chains...] -- 0:43:16       Average standard deviation of split frequencies: 0.006595       21560000 -- (-7568.067) [...7 remote chains...] -- 0:43:13      21570000 -- (-7566.714) [...7 remote chains...] -- 0:43:10      21580000 -- [-7544.948] [...7 remote chains...] -- 0:43:07      21590000 -- (-7535.395) [...7 remote chains...] -- 0:43:04      21600000 -- [-7541.128] [...7 remote chains...] -- 0:43:01       Average standard deviation of split frequencies: 0.006592       21610000 -- [-7546.353] [...7 remote chains...] -- 0:42:58      21620000 -- (-7559.826) [...7 remote chains...] -- 0:42:55      21630000 -- [-7549.422] [...7 remote chains...] -- 0:42:52      21640000 -- (-7546.858) [...7 remote chains...] -- 0:42:49      21650000 -- (-7575.605) [...7 remote chains...] -- 0:42:45       Average standard deviation of split frequencies: 0.006461       21660000 -- (-7558.186) [...7 remote chains...] -- 0:42:42      21670000 -- (-7574.875) [...7 remote chains...] -- 0:42:39      21680000 -- (-7562.756) [...7 remote chains...] -- 0:42:36      21690000 -- (-7565.794) [...7 remote chains...] -- 0:42:33      21700000 -- (-7561.152) [...7 remote chains...] -- 0:42:30       Average standard deviation of split frequencies: 0.006490       21710000 -- (-7537.883) [...7 remote chains...] -- 0:42:27      21720000 -- (-7564.420) [...7 remote chains...] -- 0:42:24      21730000 -- (-7569.505) [...7 remote chains...] -- 0:42:21      21740000 -- (-7551.561) [...7 remote chains...] -- 0:42:18      21750000 -- (-7543.313) [...7 remote chains...] -- 0:42:14       Average standard deviation of split frequencies: 0.006530       21760000 -- (-7555.520) [...7 remote chains...] -- 0:42:11      21770000 -- (-7576.731) [...7 remote chains...] -- 0:42:08      21780000 -- (-7550.179) [...7 remote chains...] -- 0:42:05      21790000 -- (-7535.379) [...7 remote chains...] -- 0:42:02      21800000 -- (-7558.514) [...7 remote chains...] -- 0:41:59       Average standard deviation of split frequencies: 0.006570       21810000 -- (-7545.333) [...7 remote chains...] -- 0:41:56      21820000 -- (-7534.059) [...7 remote chains...] -- 0:41:53      21830000 -- (-7566.323) [...7 remote chains...] -- 0:41:50      21840000 -- (-7536.249) [...7 remote chains...] -- 0:41:47      21850000 -- (-7578.459) [...7 remote chains...] -- 0:41:43       Average standard deviation of split frequencies: 0.006513       21860000 -- (-7546.244) [...7 remote chains...] -- 0:41:40      21870000 -- (-7555.559) [...7 remote chains...] -- 0:41:38      21880000 -- (-7534.560) [...7 remote chains...] -- 0:41:35      21890000 -- (-7551.694) [...7 remote chains...] -- 0:41:31      21900000 -- (-7550.432) [...7 remote chains...] -- 0:41:28       Average standard deviation of split frequencies: 0.006531       21910000 -- (-7568.668) [...7 remote chains...] -- 0:41:25      21920000 -- (-7559.223) [...7 remote chains...] -- 0:41:22      21930000 -- (-7551.548) [...7 remote chains...] -- 0:41:19      21940000 -- (-7562.081) [...7 remote chains...] -- 0:41:16      21950000 -- (-7544.316) [...7 remote chains...] -- 0:41:13       Average standard deviation of split frequencies: 0.006382       21960000 -- (-7562.634) [...7 remote chains...] -- 0:41:10      21970000 -- (-7554.540) [...7 remote chains...] -- 0:41:07      21980000 -- (-7558.716) [...7 remote chains...] -- 0:41:04      21990000 -- [-7542.130] [...7 remote chains...] -- 0:41:00      22000000 -- (-7554.222) [...7 remote chains...] -- 0:40:57       Average standard deviation of split frequencies: 0.006403       22010000 -- (-7570.942) [...7 remote chains...] -- 0:40:54      22020000 -- [-7537.940] [...7 remote chains...] -- 0:40:51      22030000 -- [-7550.818] [...7 remote chains...] -- 0:40:48      22040000 -- (-7540.989) [...7 remote chains...] -- 0:40:45      22050000 -- (-7575.817) [...7 remote chains...] -- 0:40:42       Average standard deviation of split frequencies: 0.006654       22060000 -- (-7544.866) [...7 remote chains...] -- 0:40:39      22070000 -- (-7553.574) [...7 remote chains...] -- 0:40:36      22080000 -- (-7541.533) [...7 remote chains...] -- 0:40:33      22090000 -- (-7562.865) [...7 remote chains...] -- 0:40:29      22100000 -- [-7555.032] [...7 remote chains...] -- 0:40:26       Average standard deviation of split frequencies: 0.006716       22110000 -- (-7544.089) [...7 remote chains...] -- 0:40:23      22120000 -- (-7558.160) [...7 remote chains...] -- 0:40:20      22130000 -- (-7568.648) [...7 remote chains...] -- 0:40:17      22140000 -- (-7531.584) [...7 remote chains...] -- 0:40:14      22150000 -- (-7542.691) [...7 remote chains...] -- 0:40:11       Average standard deviation of split frequencies: 0.006884       22160000 -- (-7557.974) [...7 remote chains...] -- 0:40:08      22170000 -- (-7577.395) [...7 remote chains...] -- 0:40:05      22180000 -- (-7561.112) [...7 remote chains...] -- 0:40:02      22190000 -- (-7540.581) [...7 remote chains...] -- 0:39:59      22200000 -- (-7542.527) [...7 remote chains...] -- 0:39:56       Average standard deviation of split frequencies: 0.007052       22210000 -- (-7554.416) [...7 remote chains...] -- 0:39:53      22220000 -- (-7528.663) [...7 remote chains...] -- 0:39:50      22230000 -- (-7560.508) [...7 remote chains...] -- 0:39:46      22240000 -- [-7544.317] [...7 remote chains...] -- 0:39:43      22250000 -- [-7545.805] [...7 remote chains...] -- 0:39:40       Average standard deviation of split frequencies: 0.007121       22260000 -- (-7565.988) [...7 remote chains...] -- 0:39:37      22270000 -- [-7557.293] [...7 remote chains...] -- 0:39:34      22280000 -- (-7544.792) [...7 remote chains...] -- 0:39:31      22290000 -- (-7541.614) [...7 remote chains...] -- 0:39:28      22300000 -- (-7542.921) [...7 remote chains...] -- 0:39:25       Average standard deviation of split frequencies: 0.007192       22310000 -- (-7579.890) [...7 remote chains...] -- 0:39:22      22320000 -- (-7556.290) [...7 remote chains...] -- 0:39:19      22330000 -- (-7556.727) [...7 remote chains...] -- 0:39:15      22340000 -- (-7565.934) [...7 remote chains...] -- 0:39:12      22350000 -- (-7570.119) [...7 remote chains...] -- 0:39:09       Average standard deviation of split frequencies: 0.007201       22360000 -- (-7555.197) [...7 remote chains...] -- 0:39:06      22370000 -- (-7560.000) [...7 remote chains...] -- 0:39:03      22380000 -- (-7569.684) [...7 remote chains...] -- 0:39:00      22390000 -- (-7582.505) [...7 remote chains...] -- 0:38:57      22400000 -- (-7553.434) [...7 remote chains...] -- 0:38:54       Average standard deviation of split frequencies: 0.007136       22410000 -- (-7561.813) [...7 remote chains...] -- 0:38:51      22420000 -- (-7556.925) [...7 remote chains...] -- 0:38:48      22430000 -- (-7559.380) [...7 remote chains...] -- 0:38:45      22440000 -- (-7557.387) [...7 remote chains...] -- 0:38:42      22450000 -- (-7566.273) [...7 remote chains...] -- 0:38:39       Average standard deviation of split frequencies: 0.007156       22460000 -- (-7562.535) [...7 remote chains...] -- 0:38:36      22470000 -- (-7570.320) [...7 remote chains...] -- 0:38:32      22480000 -- (-7566.813) [...7 remote chains...] -- 0:38:29      22490000 -- [-7554.048] [...7 remote chains...] -- 0:38:26      22500000 -- (-7564.796) [...7 remote chains...] -- 0:38:23       Average standard deviation of split frequencies: 0.007169       22510000 -- (-7546.885) [...7 remote chains...] -- 0:38:20      22520000 -- (-7536.511) [...7 remote chains...] -- 0:38:17      22530000 -- [-7565.402] [...7 remote chains...] -- 0:38:14      22540000 -- (-7567.068) [...7 remote chains...] -- 0:38:11      22550000 -- (-7548.763) [...7 remote chains...] -- 0:38:08       Average standard deviation of split frequencies: 0.007292       22560000 -- (-7550.500) [...7 remote chains...] -- 0:38:05      22570000 -- (-7565.025) [...7 remote chains...] -- 0:38:02      22580000 -- (-7553.887) [...7 remote chains...] -- 0:37:58      22590000 -- (-7543.978) [...7 remote chains...] -- 0:37:55      22600000 -- (-7569.970) [...7 remote chains...] -- 0:37:52       Average standard deviation of split frequencies: 0.007276       22610000 -- (-7553.153) [...7 remote chains...] -- 0:37:49      22620000 -- (-7569.513) [...7 remote chains...] -- 0:37:46      22630000 -- (-7555.436) [...7 remote chains...] -- 0:37:43      22640000 -- (-7542.719) [...7 remote chains...] -- 0:37:40      22650000 -- (-7563.063) [...7 remote chains...] -- 0:37:37       Average standard deviation of split frequencies: 0.007287       22660000 -- (-7562.924) [...7 remote chains...] -- 0:37:34      22670000 -- (-7571.926) [...7 remote chains...] -- 0:37:31      22680000 -- (-7564.024) [...7 remote chains...] -- 0:37:27      22690000 -- (-7569.184) [...7 remote chains...] -- 0:37:24      22700000 -- (-7541.249) [...7 remote chains...] -- 0:37:21       Average standard deviation of split frequencies: 0.007297       22710000 -- (-7568.146) [...7 remote chains...] -- 0:37:18      22720000 -- (-7541.125) [...7 remote chains...] -- 0:37:15      22730000 -- (-7558.733) [...7 remote chains...] -- 0:37:12      22740000 -- (-7571.456) [...7 remote chains...] -- 0:37:09      22750000 -- (-7587.193) [...7 remote chains...] -- 0:37:06       Average standard deviation of split frequencies: 0.007309       22760000 -- (-7557.637) [...7 remote chains...] -- 0:37:03      22770000 -- (-7567.105) [...7 remote chains...] -- 0:37:00      22780000 -- [-7544.466] [...7 remote chains...] -- 0:36:57      22790000 -- (-7539.042) [...7 remote chains...] -- 0:36:54      22800000 -- (-7563.210) [...7 remote chains...] -- 0:36:50       Average standard deviation of split frequencies: 0.007275       22810000 -- (-7574.147) [...7 remote chains...] -- 0:36:47      22820000 -- (-7555.768) [...7 remote chains...] -- 0:36:44      22830000 -- (-7555.786) [...7 remote chains...] -- 0:36:41      22840000 -- [-7553.446] [...7 remote chains...] -- 0:36:38      22850000 -- (-7560.288) [...7 remote chains...] -- 0:36:35       Average standard deviation of split frequencies: 0.007255       22860000 -- [-7545.188] [...7 remote chains...] -- 0:36:32      22870000 -- [-7560.620] [...7 remote chains...] -- 0:36:29      22880000 -- (-7561.286) [...7 remote chains...] -- 0:36:26      22890000 -- [-7545.349] [...7 remote chains...] -- 0:36:23      22900000 -- [-7569.563] [...7 remote chains...] -- 0:36:20       Average standard deviation of split frequencies: 0.007247       22910000 -- [-7573.644] [...7 remote chains...] -- 0:36:17      22920000 -- [-7542.660] [...7 remote chains...] -- 0:36:14      22930000 -- (-7549.014) [...7 remote chains...] -- 0:36:10      22940000 -- (-7557.257) [...7 remote chains...] -- 0:36:07      22950000 -- (-7551.862) [...7 remote chains...] -- 0:36:04       Average standard deviation of split frequencies: 0.007330       22960000 -- (-7551.172) [...7 remote chains...] -- 0:36:01      22970000 -- (-7581.664) [...7 remote chains...] -- 0:35:58      22980000 -- (-7562.091) [...7 remote chains...] -- 0:35:55      22990000 -- (-7589.189) [...7 remote chains...] -- 0:35:52      23000000 -- (-7550.935) [...7 remote chains...] -- 0:35:49       Average standard deviation of split frequencies: 0.007542       23010000 -- (-7573.772) [...7 remote chains...] -- 0:35:46      23020000 -- (-7551.007) [...7 remote chains...] -- 0:35:43      23030000 -- (-7541.752) [...7 remote chains...] -- 0:35:40      23040000 -- (-7547.431) [...7 remote chains...] -- 0:35:36      23050000 -- (-7545.330) [...7 remote chains...] -- 0:35:33       Average standard deviation of split frequencies: 0.007597       23060000 -- (-7574.649) [...7 remote chains...] -- 0:35:30      23070000 -- (-7575.790) [...7 remote chains...] -- 0:35:27      23080000 -- (-7536.224) [...7 remote chains...] -- 0:35:24      23090000 -- (-7555.995) [...7 remote chains...] -- 0:35:21      23100000 -- (-7554.550) [...7 remote chains...] -- 0:35:18       Average standard deviation of split frequencies: 0.007684       23110000 -- (-7560.618) [...7 remote chains...] -- 0:35:15      23120000 -- (-7561.944) [...7 remote chains...] -- 0:35:12      23130000 -- (-7561.142) [...7 remote chains...] -- 0:35:09      23140000 -- (-7560.809) [...7 remote chains...] -- 0:35:06      23150000 -- (-7544.292) [...7 remote chains...] -- 0:35:03       Average standard deviation of split frequencies: 0.007695       23160000 -- (-7550.981) [...7 remote chains...] -- 0:35:00      23170000 -- (-7545.513) [...7 remote chains...] -- 0:34:57      23180000 -- (-7557.843) [...7 remote chains...] -- 0:34:53      23190000 -- (-7550.368) [...7 remote chains...] -- 0:34:50      23200000 -- (-7553.490) [...7 remote chains...] -- 0:34:47       Average standard deviation of split frequencies: 0.007732       23210000 -- (-7553.695) [...7 remote chains...] -- 0:34:44      23220000 -- (-7555.404) [...7 remote chains...] -- 0:34:41      23230000 -- (-7568.214) [...7 remote chains...] -- 0:34:38      23240000 -- [-7540.839] [...7 remote chains...] -- 0:34:35      23250000 -- [-7543.861] [...7 remote chains...] -- 0:34:32       Average standard deviation of split frequencies: 0.007834       23260000 -- [-7546.253] [...7 remote chains...] -- 0:34:29      23270000 -- [-7564.362] [...7 remote chains...] -- 0:34:26      23280000 -- [-7548.759] [...7 remote chains...] -- 0:34:23      23290000 -- [-7542.196] [...7 remote chains...] -- 0:34:19      23300000 -- [-7533.050] [...7 remote chains...] -- 0:34:16       Average standard deviation of split frequencies: 0.007790       23310000 -- [-7532.694] [...7 remote chains...] -- 0:34:13      23320000 -- [-7542.678] [...7 remote chains...] -- 0:34:10      23330000 -- [-7539.756] [...7 remote chains...] -- 0:34:07      23340000 -- [-7552.794] [...7 remote chains...] -- 0:34:04      23350000 -- [-7557.007] [...7 remote chains...] -- 0:34:01       Average standard deviation of split frequencies: 0.007731       23360000 -- [-7549.835] [...7 remote chains...] -- 0:33:58      23370000 -- [-7552.309] [...7 remote chains...] -- 0:33:55      23380000 -- [-7554.839] [...7 remote chains...] -- 0:33:52      23390000 -- [-7556.509] [...7 remote chains...] -- 0:33:49      23400000 -- [-7540.811] [...7 remote chains...] -- 0:33:45       Average standard deviation of split frequencies: 0.007545       23410000 -- (-7555.067) [...7 remote chains...] -- 0:33:42      23420000 -- (-7587.076) [...7 remote chains...] -- 0:33:39      23430000 -- [-7580.517] [...7 remote chains...] -- 0:33:36      23440000 -- [-7558.231] [...7 remote chains...] -- 0:33:33      23450000 -- [-7569.722] [...7 remote chains...] -- 0:33:30       Average standard deviation of split frequencies: 0.007442       23460000 -- [-7549.875] [...7 remote chains...] -- 0:33:27      23470000 -- [-7529.679] [...7 remote chains...] -- 0:33:24      23480000 -- [-7535.760] [...7 remote chains...] -- 0:33:21      23490000 -- [-7549.413] [...7 remote chains...] -- 0:33:18      23500000 -- (-7546.577) [...7 remote chains...] -- 0:33:15       Average standard deviation of split frequencies: 0.007413       23510000 -- (-7568.429) [...7 remote chains...] -- 0:33:11      23520000 -- (-7555.318) [...7 remote chains...] -- 0:33:08      23530000 -- (-7518.259) [...7 remote chains...] -- 0:33:05      23540000 -- [-7547.927] [...7 remote chains...] -- 0:33:02      23550000 -- (-7573.740) [...7 remote chains...] -- 0:32:59       Average standard deviation of split frequencies: 0.007448       23560000 -- [-7550.049] [...7 remote chains...] -- 0:32:56      23570000 -- (-7539.894) [...7 remote chains...] -- 0:32:53      23580000 -- [-7542.536] [...7 remote chains...] -- 0:32:50      23590000 -- (-7570.215) [...7 remote chains...] -- 0:32:47      23600000 -- (-7559.860) [...7 remote chains...] -- 0:32:44       Average standard deviation of split frequencies: 0.007445       23610000 -- (-7560.209) [...7 remote chains...] -- 0:32:41      23620000 -- (-7548.104) [...7 remote chains...] -- 0:32:38      23630000 -- (-7596.277) [...7 remote chains...] -- 0:32:35      23640000 -- (-7560.530) [...7 remote chains...] -- 0:32:32      23650000 -- (-7552.804) [...7 remote chains...] -- 0:32:29       Average standard deviation of split frequencies: 0.007569       23660000 -- (-7568.306) [...7 remote chains...] -- 0:32:25      23670000 -- (-7548.233) [...7 remote chains...] -- 0:32:22      23680000 -- (-7555.334) [...7 remote chains...] -- 0:32:19      23690000 -- (-7566.536) [...7 remote chains...] -- 0:32:16      23700000 -- (-7557.288) [...7 remote chains...] -- 0:32:13       Average standard deviation of split frequencies: 0.007427       23710000 -- (-7557.765) [...7 remote chains...] -- 0:32:10      23720000 -- (-7565.867) [...7 remote chains...] -- 0:32:07      23730000 -- (-7555.518) [...7 remote chains...] -- 0:32:04      23740000 -- (-7560.319) [...7 remote chains...] -- 0:32:01      23750000 -- (-7573.776) [...7 remote chains...] -- 0:31:58       Average standard deviation of split frequencies: 0.007367       23760000 -- (-7568.054) [...7 remote chains...] -- 0:31:55      23770000 -- (-7574.195) [...7 remote chains...] -- 0:31:51      23780000 -- (-7559.477) [...7 remote chains...] -- 0:31:48      23790000 -- (-7563.787) [...7 remote chains...] -- 0:31:45      23800000 -- (-7575.266) [...7 remote chains...] -- 0:31:42       Average standard deviation of split frequencies: 0.007403       23810000 -- (-7558.984) [...7 remote chains...] -- 0:31:39      23820000 -- (-7579.816) [...7 remote chains...] -- 0:31:36      23830000 -- (-7561.762) [...7 remote chains...] -- 0:31:33      23840000 -- (-7578.808) [...7 remote chains...] -- 0:31:30      23850000 -- (-7564.190) [...7 remote chains...] -- 0:31:27       Average standard deviation of split frequencies: 0.007502       23860000 -- (-7538.257) [...7 remote chains...] -- 0:31:24      23870000 -- (-7561.212) [...7 remote chains...] -- 0:31:21      23880000 -- (-7557.372) [...7 remote chains...] -- 0:31:18      23890000 -- (-7558.779) [...7 remote chains...] -- 0:31:14      23900000 -- (-7546.883) [...7 remote chains...] -- 0:31:11       Average standard deviation of split frequencies: 0.007571       23910000 -- (-7542.422) [...7 remote chains...] -- 0:31:08      23920000 -- (-7554.703) [...7 remote chains...] -- 0:31:05      23930000 -- (-7562.470) [...7 remote chains...] -- 0:31:02      23940000 -- (-7553.002) [...7 remote chains...] -- 0:30:59      23950000 -- (-7549.567) [...7 remote chains...] -- 0:30:56       Average standard deviation of split frequencies: 0.007620       23960000 -- (-7550.333) [...7 remote chains...] -- 0:30:53      23970000 -- (-7544.073) [...7 remote chains...] -- 0:30:50      23980000 -- (-7555.309) [...7 remote chains...] -- 0:30:47      23990000 -- (-7566.256) [...7 remote chains...] -- 0:30:44      24000000 -- (-7575.477) [...7 remote chains...] -- 0:30:41       Average standard deviation of split frequencies: 0.007739       24010000 -- (-7550.499) [...7 remote chains...] -- 0:30:38      24020000 -- (-7548.369) [...7 remote chains...] -- 0:30:35      24030000 -- (-7539.028) [...7 remote chains...] -- 0:30:31      24040000 -- (-7560.690) [...7 remote chains...] -- 0:30:28      24050000 -- (-7541.205) [...7 remote chains...] -- 0:30:25       Average standard deviation of split frequencies: 0.007671       24060000 -- (-7555.275) [...7 remote chains...] -- 0:30:22      24070000 -- (-7557.897) [...7 remote chains...] -- 0:30:19      24080000 -- (-7546.412) [...7 remote chains...] -- 0:30:16      24090000 -- (-7566.844) [...7 remote chains...] -- 0:30:13      24100000 -- (-7574.314) [...7 remote chains...] -- 0:30:10       Average standard deviation of split frequencies: 0.007685       24110000 -- (-7552.076) [...7 remote chains...] -- 0:30:07      24120000 -- (-7532.735) [...7 remote chains...] -- 0:30:04      24130000 -- (-7555.844) [...7 remote chains...] -- 0:30:01      24140000 -- (-7566.197) [...7 remote chains...] -- 0:29:58      24150000 -- (-7558.584) [...7 remote chains...] -- 0:29:54       Average standard deviation of split frequencies: 0.007802       24160000 -- (-7555.631) [...7 remote chains...] -- 0:29:51      24170000 -- (-7569.348) [...7 remote chains...] -- 0:29:48      24180000 -- (-7547.678) [...7 remote chains...] -- 0:29:45      24190000 -- (-7556.150) [...7 remote chains...] -- 0:29:42      24200000 -- (-7553.723) [...7 remote chains...] -- 0:29:39       Average standard deviation of split frequencies: 0.007694       24210000 -- (-7564.167) [...7 remote chains...] -- 0:29:36      24220000 -- (-7576.420) [...7 remote chains...] -- 0:29:33      24230000 -- (-7551.105) [...7 remote chains...] -- 0:29:30      24240000 -- (-7570.200) [...7 remote chains...] -- 0:29:27      24250000 -- (-7585.010) [...7 remote chains...] -- 0:29:24       Average standard deviation of split frequencies: 0.007658       24260000 -- (-7561.174) [...7 remote chains...] -- 0:29:21      24270000 -- (-7549.603) [...7 remote chains...] -- 0:29:18      24280000 -- (-7556.506) [...7 remote chains...] -- 0:29:15      24290000 -- (-7562.108) [...7 remote chains...] -- 0:29:12      24300000 -- (-7536.949) [...7 remote chains...] -- 0:29:08       Average standard deviation of split frequencies: 0.007491       24310000 -- (-7549.627) [...7 remote chains...] -- 0:29:05      24320000 -- [-7539.944] [...7 remote chains...] -- 0:29:02      24330000 -- (-7554.046) [...7 remote chains...] -- 0:28:59      24340000 -- (-7568.091) [...7 remote chains...] -- 0:28:56      24350000 -- [-7560.538] [...7 remote chains...] -- 0:28:53       Average standard deviation of split frequencies: 0.007514       24360000 -- [-7550.588] [...7 remote chains...] -- 0:28:50      24370000 -- (-7547.073) [...7 remote chains...] -- 0:28:47      24380000 -- (-7570.422) [...7 remote chains...] -- 0:28:44      24390000 -- (-7549.942) [...7 remote chains...] -- 0:28:41      24400000 -- (-7547.236) [...7 remote chains...] -- 0:28:38       Average standard deviation of split frequencies: 0.007272       24410000 -- [-7551.038] [...7 remote chains...] -- 0:28:35      24420000 -- [-7546.531] [...7 remote chains...] -- 0:28:31      24430000 -- [-7551.448] [...7 remote chains...] -- 0:28:28      24440000 -- [-7555.407] [...7 remote chains...] -- 0:28:25      24450000 -- [-7540.344] [...7 remote chains...] -- 0:28:22       Average standard deviation of split frequencies: 0.007251       24460000 -- [-7544.232] [...7 remote chains...] -- 0:28:19      24470000 -- [-7550.182] [...7 remote chains...] -- 0:28:16      24480000 -- [-7548.518] [...7 remote chains...] -- 0:28:13      24490000 -- [-7541.642] [...7 remote chains...] -- 0:28:10      24500000 -- [-7545.738] [...7 remote chains...] -- 0:28:07       Average standard deviation of split frequencies: 0.007217       24510000 -- [-7561.510] [...7 remote chains...] -- 0:28:04      24520000 -- [-7550.386] [...7 remote chains...] -- 0:28:01      24530000 -- (-7545.555) [...7 remote chains...] -- 0:27:58      24540000 -- (-7546.321) [...7 remote chains...] -- 0:27:54      24550000 -- (-7560.045) [...7 remote chains...] -- 0:27:51       Average standard deviation of split frequencies: 0.007107       24560000 -- (-7603.217) [...7 remote chains...] -- 0:27:48      24570000 -- (-7582.999) [...7 remote chains...] -- 0:27:45      24580000 -- (-7535.437) [...7 remote chains...] -- 0:27:42      24590000 -- (-7547.676) [...7 remote chains...] -- 0:27:39      24600000 -- (-7541.736) [...7 remote chains...] -- 0:27:36       Average standard deviation of split frequencies: 0.007062       24610000 -- (-7556.614) [...7 remote chains...] -- 0:27:33      24620000 -- (-7567.142) [...7 remote chains...] -- 0:27:30      24630000 -- (-7534.012) [...7 remote chains...] -- 0:27:27      24640000 -- (-7572.585) [...7 remote chains...] -- 0:27:24      24650000 -- [-7558.545] [...7 remote chains...] -- 0:27:21       Average standard deviation of split frequencies: 0.007128       24660000 -- [-7531.219] [...7 remote chains...] -- 0:27:17      24670000 -- [-7545.073] [...7 remote chains...] -- 0:27:14      24680000 -- [-7548.988] [...7 remote chains...] -- 0:27:11      24690000 -- (-7534.868) [...7 remote chains...] -- 0:27:08      24700000 -- [-7547.165] [...7 remote chains...] -- 0:27:05       Average standard deviation of split frequencies: 0.007191       24710000 -- [-7545.144] [...7 remote chains...] -- 0:27:02      24720000 -- [-7558.205] [...7 remote chains...] -- 0:26:59      24730000 -- (-7554.439) [...7 remote chains...] -- 0:26:56      24740000 -- [-7552.974] [...7 remote chains...] -- 0:26:53      24750000 -- [-7537.239] [...7 remote chains...] -- 0:26:50       Average standard deviation of split frequencies: 0.007313       24760000 -- (-7565.082) [...7 remote chains...] -- 0:26:47      24770000 -- (-7561.115) [...7 remote chains...] -- 0:26:44      24780000 -- (-7551.117) [...7 remote chains...] -- 0:26:41      24790000 -- (-7543.667) [...7 remote chains...] -- 0:26:38      24800000 -- (-7570.520) [...7 remote chains...] -- 0:26:35       Average standard deviation of split frequencies: 0.007264       24810000 -- (-7554.404) [...7 remote chains...] -- 0:26:31      24820000 -- (-7551.126) [...7 remote chains...] -- 0:26:28      24830000 -- (-7556.739) [...7 remote chains...] -- 0:26:25      24840000 -- (-7553.646) [...7 remote chains...] -- 0:26:22      24850000 -- (-7546.971) [...7 remote chains...] -- 0:26:19       Average standard deviation of split frequencies: 0.007368       24860000 -- (-7549.862) [...7 remote chains...] -- 0:26:16      24870000 -- (-7555.009) [...7 remote chains...] -- 0:26:13      24880000 -- (-7552.776) [...7 remote chains...] -- 0:26:10      24890000 -- (-7554.821) [...7 remote chains...] -- 0:26:07      24900000 -- (-7562.804) [...7 remote chains...] -- 0:26:04       Average standard deviation of split frequencies: 0.007432       24910000 -- (-7535.676) [...7 remote chains...] -- 0:26:01      24920000 -- (-7550.173) [...7 remote chains...] -- 0:25:58      24930000 -- (-7558.234) [...7 remote chains...] -- 0:25:54      24940000 -- (-7551.311) [...7 remote chains...] -- 0:25:51      24950000 -- (-7538.296) [...7 remote chains...] -- 0:25:48       Average standard deviation of split frequencies: 0.007420       24960000 -- (-7546.127) [...7 remote chains...] -- 0:25:45      24970000 -- (-7557.990) [...7 remote chains...] -- 0:25:42      24980000 -- (-7557.090) [...7 remote chains...] -- 0:25:39      24990000 -- (-7565.318) [...7 remote chains...] -- 0:25:36      25000000 -- (-7555.832) [...7 remote chains...] -- 0:25:33       Average standard deviation of split frequencies: 0.007333       25010000 -- (-7546.942) [...7 remote chains...] -- 0:25:30      25020000 -- (-7535.869) [...7 remote chains...] -- 0:25:27      25030000 -- (-7539.124) [...7 remote chains...] -- 0:25:24      25040000 -- (-7565.846) [...7 remote chains...] -- 0:25:21      25050000 -- (-7539.276) [...7 remote chains...] -- 0:25:18       Average standard deviation of split frequencies: 0.007267       25060000 -- (-7553.104) [...7 remote chains...] -- 0:25:15      25070000 -- (-7549.922) [...7 remote chains...] -- 0:25:12      25080000 -- [-7562.610] [...7 remote chains...] -- 0:25:08      25090000 -- [-7550.770] [...7 remote chains...] -- 0:25:05      25100000 -- (-7566.196) [...7 remote chains...] -- 0:25:02       Average standard deviation of split frequencies: 0.007208       25110000 -- (-7550.089) [...7 remote chains...] -- 0:24:59      25120000 -- (-7556.772) [...7 remote chains...] -- 0:24:56      25130000 -- (-7572.649) [...7 remote chains...] -- 0:24:53      25140000 -- (-7561.620) [...7 remote chains...] -- 0:24:50      25150000 -- (-7553.921) [...7 remote chains...] -- 0:24:47       Average standard deviation of split frequencies: 0.007127       25160000 -- (-7564.806) [...7 remote chains...] -- 0:24:44      25170000 -- (-7570.529) [...7 remote chains...] -- 0:24:41      25180000 -- (-7570.758) [...7 remote chains...] -- 0:24:38      25190000 -- (-7563.227) [...7 remote chains...] -- 0:24:35      25200000 -- (-7552.904) [...7 remote chains...] -- 0:24:32       Average standard deviation of split frequencies: 0.006991       25210000 -- (-7543.496) [...7 remote chains...] -- 0:24:28      25220000 -- (-7548.371) [...7 remote chains...] -- 0:24:25      25230000 -- (-7546.494) [...7 remote chains...] -- 0:24:22      25240000 -- (-7581.730) [...7 remote chains...] -- 0:24:19      25250000 -- (-7554.041) [...7 remote chains...] -- 0:24:16       Average standard deviation of split frequencies: 0.006861       25260000 -- (-7541.596) [...7 remote chains...] -- 0:24:13      25270000 -- (-7571.988) [...7 remote chains...] -- 0:24:10      25280000 -- (-7561.432) [...7 remote chains...] -- 0:24:07      25290000 -- (-7553.881) [...7 remote chains...] -- 0:24:04      25300000 -- (-7542.739) [...7 remote chains...] -- 0:24:01       Average standard deviation of split frequencies: 0.006869       25310000 -- [-7543.275] [...7 remote chains...] -- 0:23:58      25320000 -- (-7545.347) [...7 remote chains...] -- 0:23:55      25330000 -- (-7557.353) [...7 remote chains...] -- 0:23:52      25340000 -- (-7555.105) [...7 remote chains...] -- 0:23:49      25350000 -- (-7540.406) [...7 remote chains...] -- 0:23:46       Average standard deviation of split frequencies: 0.006842       25360000 -- (-7550.661) [...7 remote chains...] -- 0:23:42      25370000 -- [-7543.454] [...7 remote chains...] -- 0:23:39      25380000 -- (-7561.483) [...7 remote chains...] -- 0:23:36      25390000 -- [-7563.904] [...7 remote chains...] -- 0:23:33      25400000 -- (-7533.915) [...7 remote chains...] -- 0:23:30       Average standard deviation of split frequencies: 0.006876       25410000 -- (-7553.481) [...7 remote chains...] -- 0:23:27      25420000 -- (-7541.709) [...7 remote chains...] -- 0:23:24      25430000 -- (-7547.422) [...7 remote chains...] -- 0:23:21      25440000 -- (-7543.891) [...7 remote chains...] -- 0:23:18      25450000 -- (-7536.924) [...7 remote chains...] -- 0:23:15       Average standard deviation of split frequencies: 0.006956       25460000 -- (-7550.399) [...7 remote chains...] -- 0:23:12      25470000 -- [-7547.616] [...7 remote chains...] -- 0:23:09      25480000 -- (-7568.936) [...7 remote chains...] -- 0:23:05      25490000 -- (-7546.550) [...7 remote chains...] -- 0:23:02      25500000 -- (-7544.269) [...7 remote chains...] -- 0:22:59       Average standard deviation of split frequencies: 0.007017       25510000 -- (-7551.251) [...7 remote chains...] -- 0:22:56      25520000 -- (-7574.108) [...7 remote chains...] -- 0:22:53      25530000 -- (-7569.052) [...7 remote chains...] -- 0:22:50      25540000 -- (-7549.952) [...7 remote chains...] -- 0:22:47      25550000 -- (-7551.745) [...7 remote chains...] -- 0:22:44       Average standard deviation of split frequencies: 0.006981       25560000 -- (-7572.562) [...7 remote chains...] -- 0:22:41      25570000 -- (-7550.838) [...7 remote chains...] -- 0:22:38      25580000 -- (-7557.092) [...7 remote chains...] -- 0:22:35      25590000 -- (-7559.822) [...7 remote chains...] -- 0:22:32      25600000 -- (-7562.394) [...7 remote chains...] -- 0:22:29       Average standard deviation of split frequencies: 0.006962       25610000 -- (-7551.451) [...7 remote chains...] -- 0:22:25      25620000 -- (-7565.157) [...7 remote chains...] -- 0:22:22      25630000 -- (-7572.183) [...7 remote chains...] -- 0:22:19      25640000 -- (-7566.013) [...7 remote chains...] -- 0:22:16      25650000 -- (-7557.461) [...7 remote chains...] -- 0:22:13       Average standard deviation of split frequencies: 0.006899       25660000 -- (-7558.763) [...7 remote chains...] -- 0:22:10      25670000 -- (-7565.018) [...7 remote chains...] -- 0:22:07      25680000 -- (-7538.075) [...7 remote chains...] -- 0:22:04      25690000 -- (-7548.459) [...7 remote chains...] -- 0:22:01      25700000 -- [-7556.030] [...7 remote chains...] -- 0:21:58       Average standard deviation of split frequencies: 0.006965       25710000 -- (-7539.530) [...7 remote chains...] -- 0:21:55      25720000 -- (-7552.839) [...7 remote chains...] -- 0:21:52      25730000 -- (-7554.758) [...7 remote chains...] -- 0:21:49      25740000 -- (-7572.425) [...7 remote chains...] -- 0:21:46      25750000 -- (-7562.681) [...7 remote chains...] -- 0:21:43       Average standard deviation of split frequencies: 0.006888       25760000 -- (-7551.615) [...7 remote chains...] -- 0:21:39      25770000 -- (-7578.057) [...7 remote chains...] -- 0:21:36      25780000 -- (-7573.095) [...7 remote chains...] -- 0:21:33      25790000 -- (-7565.689) [...7 remote chains...] -- 0:21:30      25800000 -- (-7538.685) [...7 remote chains...] -- 0:21:27       Average standard deviation of split frequencies: 0.006949       25810000 -- (-7571.523) [...7 remote chains...] -- 0:21:24      25820000 -- (-7547.897) [...7 remote chains...] -- 0:21:21      25830000 -- (-7549.194) [...7 remote chains...] -- 0:21:18      25840000 -- (-7554.049) [...7 remote chains...] -- 0:21:15      25850000 -- [-7568.351] [...7 remote chains...] -- 0:21:12       Average standard deviation of split frequencies: 0.006829       25860000 -- [-7545.690] [...7 remote chains...] -- 0:21:09      25870000 -- [-7552.296] [...7 remote chains...] -- 0:21:06      25880000 -- (-7551.487) [...7 remote chains...] -- 0:21:03      25890000 -- (-7563.517) [...7 remote chains...] -- 0:20:59      25900000 -- (-7556.024) [...7 remote chains...] -- 0:20:56       Average standard deviation of split frequencies: 0.006660       25910000 -- (-7551.202) [...7 remote chains...] -- 0:20:53      25920000 -- (-7538.236) [...7 remote chains...] -- 0:20:50      25930000 -- (-7592.446) [...7 remote chains...] -- 0:20:47      25940000 -- (-7569.998) [...7 remote chains...] -- 0:20:44      25950000 -- (-7563.998) [...7 remote chains...] -- 0:20:41       Average standard deviation of split frequencies: 0.006619       25960000 -- (-7538.969) [...7 remote chains...] -- 0:20:38      25970000 -- (-7558.960) [...7 remote chains...] -- 0:20:35      25980000 -- (-7580.586) [...7 remote chains...] -- 0:20:32      25990000 -- (-7548.473) [...7 remote chains...] -- 0:20:29      26000000 -- (-7542.155) [...7 remote chains...] -- 0:20:26       Average standard deviation of split frequencies: 0.006628       26010000 -- (-7565.462) [...7 remote chains...] -- 0:20:23      26020000 -- (-7570.359) [...7 remote chains...] -- 0:20:20      26030000 -- (-7563.261) [...7 remote chains...] -- 0:20:16      26040000 -- (-7559.967) [...7 remote chains...] -- 0:20:13      26050000 -- (-7563.499) [...7 remote chains...] -- 0:20:10       Average standard deviation of split frequencies: 0.006603       26060000 -- (-7550.244) [...7 remote chains...] -- 0:20:07      26070000 -- (-7529.128) [...7 remote chains...] -- 0:20:04      26080000 -- (-7548.526) [...7 remote chains...] -- 0:20:01      26090000 -- (-7570.333) [...7 remote chains...] -- 0:19:58      26100000 -- (-7559.515) [...7 remote chains...] -- 0:19:55       Average standard deviation of split frequencies: 0.006510       26110000 -- (-7560.450) [...7 remote chains...] -- 0:19:52      26120000 -- (-7553.729) [...7 remote chains...] -- 0:19:49      26130000 -- (-7549.259) [...7 remote chains...] -- 0:19:46      26140000 -- (-7557.560) [...7 remote chains...] -- 0:19:43      26150000 -- (-7566.667) [...7 remote chains...] -- 0:19:40       Average standard deviation of split frequencies: 0.006477       26160000 -- (-7553.878) [...7 remote chains...] -- 0:19:37      26170000 -- (-7548.115) [...7 remote chains...] -- 0:19:34      26180000 -- (-7553.266) [...7 remote chains...] -- 0:19:30      26190000 -- (-7544.949) [...7 remote chains...] -- 0:19:27      26200000 -- (-7552.220) [...7 remote chains...] -- 0:19:24       Average standard deviation of split frequencies: 0.006460       26210000 -- (-7567.246) [...7 remote chains...] -- 0:19:21      26220000 -- (-7557.909) [...7 remote chains...] -- 0:19:18      26230000 -- (-7554.449) [...7 remote chains...] -- 0:19:15      26240000 -- (-7553.761) [...7 remote chains...] -- 0:19:12      26250000 -- (-7580.243) [...7 remote chains...] -- 0:19:09       Average standard deviation of split frequencies: 0.006370       26260000 -- (-7584.170) [...7 remote chains...] -- 0:19:06      26270000 -- (-7580.798) [...7 remote chains...] -- 0:19:03      26280000 -- (-7562.903) [...7 remote chains...] -- 0:19:00      26290000 -- (-7566.610) [...7 remote chains...] -- 0:18:57      26300000 -- (-7557.882) [...7 remote chains...] -- 0:18:54       Average standard deviation of split frequencies: 0.006354       26310000 -- (-7570.853) [...7 remote chains...] -- 0:18:50      26320000 -- (-7560.535) [...7 remote chains...] -- 0:18:47      26330000 -- (-7549.801) [...7 remote chains...] -- 0:18:44      26340000 -- (-7561.881) [...7 remote chains...] -- 0:18:41      26350000 -- (-7536.266) [...7 remote chains...] -- 0:18:38       Average standard deviation of split frequencies: 0.006289       26360000 -- (-7547.227) [...7 remote chains...] -- 0:18:35      26370000 -- [-7549.523] [...7 remote chains...] -- 0:18:32      26380000 -- [-7545.230] [...7 remote chains...] -- 0:18:29      26390000 -- [-7545.239] [...7 remote chains...] -- 0:18:26      26400000 -- [-7544.806] [...7 remote chains...] -- 0:18:23       Average standard deviation of split frequencies: 0.006212       26410000 -- [-7552.881] [...7 remote chains...] -- 0:18:20      26420000 -- (-7561.526) [...7 remote chains...] -- 0:18:17      26430000 -- [-7551.589] [...7 remote chains...] -- 0:18:14      26440000 -- (-7550.735) [...7 remote chains...] -- 0:18:11      26450000 -- [-7556.368] [...7 remote chains...] -- 0:18:07       Average standard deviation of split frequencies: 0.006135       26460000 -- [-7550.926] [...7 remote chains...] -- 0:18:04      26470000 -- [-7550.044] [...7 remote chains...] -- 0:18:01      26480000 -- [-7561.843] [...7 remote chains...] -- 0:17:58      26490000 -- [-7567.939] [...7 remote chains...] -- 0:17:55      26500000 -- [-7566.532] [...7 remote chains...] -- 0:17:52       Average standard deviation of split frequencies: 0.006058       26510000 -- [-7557.277] [...7 remote chains...] -- 0:17:49      26520000 -- (-7570.203) [...7 remote chains...] -- 0:17:46      26530000 -- [-7563.691] [...7 remote chains...] -- 0:17:43      26540000 -- [-7553.803] [...7 remote chains...] -- 0:17:40      26550000 -- [-7570.234] [...7 remote chains...] -- 0:17:37       Average standard deviation of split frequencies: 0.006031       26560000 -- (-7571.207) [...7 remote chains...] -- 0:17:34      26570000 -- (-7567.375) [...7 remote chains...] -- 0:17:31      26580000 -- (-7552.713) [...7 remote chains...] -- 0:17:28      26590000 -- (-7554.005) [...7 remote chains...] -- 0:17:25      26600000 -- (-7584.773) [...7 remote chains...] -- 0:17:21       Average standard deviation of split frequencies: 0.006063       26610000 -- (-7563.403) [...7 remote chains...] -- 0:17:18      26620000 -- (-7564.648) [...7 remote chains...] -- 0:17:15      26630000 -- (-7554.040) [...7 remote chains...] -- 0:17:12      26640000 -- (-7541.492) [...7 remote chains...] -- 0:17:09      26650000 -- (-7562.950) [...7 remote chains...] -- 0:17:06       Average standard deviation of split frequencies: 0.006136       26660000 -- (-7555.915) [...7 remote chains...] -- 0:17:03      26670000 -- (-7545.679) [...7 remote chains...] -- 0:17:00      26680000 -- (-7547.564) [...7 remote chains...] -- 0:16:57      26690000 -- (-7569.748) [...7 remote chains...] -- 0:16:54      26700000 -- (-7567.152) [...7 remote chains...] -- 0:16:51       Average standard deviation of split frequencies: 0.006142       26710000 -- (-7554.859) [...7 remote chains...] -- 0:16:48      26720000 -- (-7545.785) [...7 remote chains...] -- 0:16:45      26730000 -- (-7554.515) [...7 remote chains...] -- 0:16:42      26740000 -- (-7553.513) [...7 remote chains...] -- 0:16:38      26750000 -- (-7568.904) [...7 remote chains...] -- 0:16:35       Average standard deviation of split frequencies: 0.006117       26760000 -- (-7544.174) [...7 remote chains...] -- 0:16:32      26770000 -- (-7558.074) [...7 remote chains...] -- 0:16:29      26780000 -- (-7582.420) [...7 remote chains...] -- 0:16:26      26790000 -- (-7566.142) [...7 remote chains...] -- 0:16:23      26800000 -- (-7565.431) [...7 remote chains...] -- 0:16:20       Average standard deviation of split frequencies: 0.006079       26810000 -- (-7554.196) [...7 remote chains...] -- 0:16:17      26820000 -- (-7556.207) [...7 remote chains...] -- 0:16:14      26830000 -- (-7554.987) [...7 remote chains...] -- 0:16:11      26840000 -- [-7537.878] [...7 remote chains...] -- 0:16:08      26850000 -- [-7544.185] [...7 remote chains...] -- 0:16:05       Average standard deviation of split frequencies: 0.005976       26860000 -- [-7535.943] [...7 remote chains...] -- 0:16:02      26870000 -- [-7556.152] [...7 remote chains...] -- 0:15:59      26880000 -- [-7546.984] [...7 remote chains...] -- 0:15:55      26890000 -- [-7537.982] [...7 remote chains...] -- 0:15:52      26900000 -- (-7545.460) [...7 remote chains...] -- 0:15:49       Average standard deviation of split frequencies: 0.006018       26910000 -- [-7543.497] [...7 remote chains...] -- 0:15:46      26920000 -- [-7555.194] [...7 remote chains...] -- 0:15:43      26930000 -- [-7548.897] [...7 remote chains...] -- 0:15:40      26940000 -- [-7560.316] [...7 remote chains...] -- 0:15:37      26950000 -- [-7541.087] [...7 remote chains...] -- 0:15:34       Average standard deviation of split frequencies: 0.006044       26960000 -- [-7548.193] [...7 remote chains...] -- 0:15:31      26970000 -- [-7547.888] [...7 remote chains...] -- 0:15:28      26980000 -- [-7545.023] [...7 remote chains...] -- 0:15:25      26990000 -- [-7535.511] [...7 remote chains...] -- 0:15:22      27000000 -- (-7541.752) [...7 remote chains...] -- 0:15:19       Average standard deviation of split frequencies: 0.006038       27010000 -- [-7541.598] [...7 remote chains...] -- 0:15:16      27020000 -- [-7556.789] [...7 remote chains...] -- 0:15:12      27030000 -- (-7554.692) [...7 remote chains...] -- 0:15:10      27040000 -- [-7547.508] [...7 remote chains...] -- 0:15:06      27050000 -- [-7550.360] [...7 remote chains...] -- 0:15:03       Average standard deviation of split frequencies: 0.006091       27060000 -- [-7547.578] [...7 remote chains...] -- 0:15:00      27070000 -- [-7548.565] [...7 remote chains...] -- 0:14:57      27080000 -- [-7546.919] [...7 remote chains...] -- 0:14:54      27090000 -- [-7549.479] [...7 remote chains...] -- 0:14:51      27100000 -- [-7556.438] [...7 remote chains...] -- 0:14:48       Average standard deviation of split frequencies: 0.006176       27110000 -- [-7566.666] [...7 remote chains...] -- 0:14:45      27120000 -- (-7561.657) [...7 remote chains...] -- 0:14:42      27130000 -- [-7532.661] [...7 remote chains...] -- 0:14:39      27140000 -- [-7531.290] [...7 remote chains...] -- 0:14:36      27150000 -- (-7555.192) [...7 remote chains...] -- 0:14:33       Average standard deviation of split frequencies: 0.006198       27160000 -- [-7539.278] [...7 remote chains...] -- 0:14:30      27170000 -- [-7538.780] [...7 remote chains...] -- 0:14:27      27180000 -- [-7554.129] [...7 remote chains...] -- 0:14:23      27190000 -- [-7569.752] [...7 remote chains...] -- 0:14:20      27200000 -- [-7548.443] [...7 remote chains...] -- 0:14:17       Average standard deviation of split frequencies: 0.006231       27210000 -- [-7542.738] [...7 remote chains...] -- 0:14:14      27220000 -- [-7567.035] [...7 remote chains...] -- 0:14:11      27230000 -- [-7567.164] [...7 remote chains...] -- 0:14:08      27240000 -- [-7569.723] [...7 remote chains...] -- 0:14:05      27250000 -- [-7558.422] [...7 remote chains...] -- 0:14:02       Average standard deviation of split frequencies: 0.006130       27260000 -- [-7554.391] [...7 remote chains...] -- 0:13:59      27270000 -- [-7540.434] [...7 remote chains...] -- 0:13:56      27280000 -- [-7547.459] [...7 remote chains...] -- 0:13:53      27290000 -- [-7555.301] [...7 remote chains...] -- 0:13:50      27300000 -- [-7552.649] [...7 remote chains...] -- 0:13:47       Average standard deviation of split frequencies: 0.006053       27310000 -- [-7551.270] [...7 remote chains...] -- 0:13:44      27320000 -- [-7551.349] [...7 remote chains...] -- 0:13:40      27330000 -- [-7557.432] [...7 remote chains...] -- 0:13:37      27340000 -- [-7573.330] [...7 remote chains...] -- 0:13:34      27350000 -- [-7560.508] [...7 remote chains...] -- 0:13:31       Average standard deviation of split frequencies: 0.006036       27360000 -- [-7536.939] [...7 remote chains...] -- 0:13:28      27370000 -- [-7572.113] [...7 remote chains...] -- 0:13:25      27380000 -- [-7554.986] [...7 remote chains...] -- 0:13:22      27390000 -- [-7570.335] [...7 remote chains...] -- 0:13:19      27400000 -- [-7548.295] [...7 remote chains...] -- 0:13:16       Average standard deviation of split frequencies: 0.006025       27410000 -- [-7564.570] [...7 remote chains...] -- 0:13:13      27420000 -- [-7568.253] [...7 remote chains...] -- 0:13:10      27430000 -- [-7579.492] [...7 remote chains...] -- 0:13:07      27440000 -- [-7555.990] [...7 remote chains...] -- 0:13:04      27450000 -- [-7555.153] [...7 remote chains...] -- 0:13:01       Average standard deviation of split frequencies: 0.005980       27460000 -- [-7557.812] [...7 remote chains...] -- 0:12:58      27470000 -- [-7542.480] [...7 remote chains...] -- 0:12:55      27480000 -- [-7563.745] [...7 remote chains...] -- 0:12:51      27490000 -- [-7539.643] [...7 remote chains...] -- 0:12:48      27500000 -- (-7535.343) [...7 remote chains...] -- 0:12:45       Average standard deviation of split frequencies: 0.005874       27510000 -- (-7564.183) [...7 remote chains...] -- 0:12:42      27520000 -- (-7566.429) [...7 remote chains...] -- 0:12:39      27530000 -- (-7547.689) [...7 remote chains...] -- 0:12:36      27540000 -- (-7545.665) [...7 remote chains...] -- 0:12:33      27550000 -- (-7558.319) [...7 remote chains...] -- 0:12:30       Average standard deviation of split frequencies: 0.005900       27560000 -- (-7541.034) [...7 remote chains...] -- 0:12:27      27570000 -- (-7546.946) [...7 remote chains...] -- 0:12:24      27580000 -- (-7556.514) [...7 remote chains...] -- 0:12:21      27590000 -- (-7564.456) [...7 remote chains...] -- 0:12:18      27600000 -- (-7556.113) [...7 remote chains...] -- 0:12:15       Average standard deviation of split frequencies: 0.005944       27610000 -- [-7544.552] [...7 remote chains...] -- 0:12:12      27620000 -- [-7548.366] [...7 remote chains...] -- 0:12:08      27630000 -- [-7558.462] [...7 remote chains...] -- 0:12:05      27640000 -- (-7565.935) [...7 remote chains...] -- 0:12:02      27650000 -- (-7534.064) [...7 remote chains...] -- 0:11:59       Average standard deviation of split frequencies: 0.005961       27660000 -- (-7569.279) [...7 remote chains...] -- 0:11:56      27670000 -- (-7569.003) [...7 remote chains...] -- 0:11:53      27680000 -- (-7602.915) [...7 remote chains...] -- 0:11:50      27690000 -- (-7549.097) [...7 remote chains...] -- 0:11:47      27700000 -- (-7571.386) [...7 remote chains...] -- 0:11:44       Average standard deviation of split frequencies: 0.005945       27710000 -- (-7560.504) [...7 remote chains...] -- 0:11:41      27720000 -- (-7580.681) [...7 remote chains...] -- 0:11:38      27730000 -- (-7556.585) [...7 remote chains...] -- 0:11:35      27740000 -- (-7573.139) [...7 remote chains...] -- 0:11:32      27750000 -- (-7544.503) [...7 remote chains...] -- 0:11:29       Average standard deviation of split frequencies: 0.005977       27760000 -- [-7554.333] [...7 remote chains...] -- 0:11:26      27770000 -- [-7567.565] [...7 remote chains...] -- 0:11:23      27780000 -- [-7544.064] [...7 remote chains...] -- 0:11:19      27790000 -- [-7566.692] [...7 remote chains...] -- 0:11:16      27800000 -- [-7537.669] [...7 remote chains...] -- 0:11:13       Average standard deviation of split frequencies: 0.006048       27810000 -- (-7568.573) [...7 remote chains...] -- 0:11:10      27820000 -- [-7555.947] [...7 remote chains...] -- 0:11:07      27830000 -- (-7566.249) [...7 remote chains...] -- 0:11:04      27840000 -- (-7564.097) [...7 remote chains...] -- 0:11:01      27850000 -- (-7561.307) [...7 remote chains...] -- 0:10:58       Average standard deviation of split frequencies: 0.006132       27860000 -- (-7570.948) [...7 remote chains...] -- 0:10:55      27870000 -- (-7567.757) [...7 remote chains...] -- 0:10:52      27880000 -- (-7554.383) [...7 remote chains...] -- 0:10:49      27890000 -- (-7573.275) [...7 remote chains...] -- 0:10:46      27900000 -- (-7558.037) [...7 remote chains...] -- 0:10:43       Average standard deviation of split frequencies: 0.006084       27910000 -- (-7556.505) [...7 remote chains...] -- 0:10:40      27920000 -- (-7534.990) [...7 remote chains...] -- 0:10:37      27930000 -- (-7545.846) [...7 remote chains...] -- 0:10:33      27940000 -- (-7540.737) [...7 remote chains...] -- 0:10:30      27950000 -- (-7571.721) [...7 remote chains...] -- 0:10:27       Average standard deviation of split frequencies: 0.005993       27960000 -- (-7568.525) [...7 remote chains...] -- 0:10:24      27970000 -- (-7540.869) [...7 remote chains...] -- 0:10:21      27980000 -- (-7557.121) [...7 remote chains...] -- 0:10:18      27990000 -- (-7567.686) [...7 remote chains...] -- 0:10:15      28000000 -- (-7565.890) [...7 remote chains...] -- 0:10:12       Average standard deviation of split frequencies: 0.006107       28010000 -- (-7548.698) [...7 remote chains...] -- 0:10:09      28020000 -- (-7541.149) [...7 remote chains...] -- 0:10:06      28030000 -- (-7559.608) [...7 remote chains...] -- 0:10:03      28040000 -- (-7587.386) [...7 remote chains...] -- 0:10:00      28050000 -- (-7573.852) [...7 remote chains...] -- 0:09:57       Average standard deviation of split frequencies: 0.006023       28060000 -- (-7564.251) [...7 remote chains...] -- 0:09:54      28070000 -- (-7544.642) [...7 remote chains...] -- 0:09:51      28080000 -- (-7560.523) [...7 remote chains...] -- 0:09:48      28090000 -- (-7573.323) [...7 remote chains...] -- 0:09:44      28100000 -- (-7554.970) [...7 remote chains...] -- 0:09:41       Average standard deviation of split frequencies: 0.005973       28110000 -- (-7585.491) [...7 remote chains...] -- 0:09:38      28120000 -- (-7545.750) [...7 remote chains...] -- 0:09:35      28130000 -- (-7558.844) [...7 remote chains...] -- 0:09:32      28140000 -- (-7547.078) [...7 remote chains...] -- 0:09:29      28150000 -- (-7564.026) [...7 remote chains...] -- 0:09:26       Average standard deviation of split frequencies: 0.006082       28160000 -- (-7568.158) [...7 remote chains...] -- 0:09:23      28170000 -- (-7547.513) [...7 remote chains...] -- 0:09:20      28180000 -- [-7561.473] [...7 remote chains...] -- 0:09:17      28190000 -- [-7546.575] [...7 remote chains...] -- 0:09:14      28200000 -- [-7549.606] [...7 remote chains...] -- 0:09:11       Average standard deviation of split frequencies: 0.006083       28210000 -- [-7566.662] [...7 remote chains...] -- 0:09:08      28220000 -- (-7558.465) [...7 remote chains...] -- 0:09:05      28230000 -- (-7553.027) [...7 remote chains...] -- 0:09:02      28240000 -- (-7565.883) [...7 remote chains...] -- 0:08:58      28250000 -- (-7541.962) [...7 remote chains...] -- 0:08:55       Average standard deviation of split frequencies: 0.006083       28260000 -- (-7553.052) [...7 remote chains...] -- 0:08:52      28270000 -- (-7568.925) [...7 remote chains...] -- 0:08:49      28280000 -- (-7575.242) [...7 remote chains...] -- 0:08:46      28290000 -- (-7574.975) [...7 remote chains...] -- 0:08:43      28300000 -- (-7569.086) [...7 remote chains...] -- 0:08:40       Average standard deviation of split frequencies: 0.006023       28310000 -- (-7563.672) [...7 remote chains...] -- 0:08:37      28320000 -- (-7549.428) [...7 remote chains...] -- 0:08:34      28330000 -- (-7575.306) [...7 remote chains...] -- 0:08:31      28340000 -- (-7552.551) [...7 remote chains...] -- 0:08:28      28350000 -- (-7555.461) [...7 remote chains...] -- 0:08:25       Average standard deviation of split frequencies: 0.005896       28360000 -- (-7543.528) [...7 remote chains...] -- 0:08:22      28370000 -- (-7550.749) [...7 remote chains...] -- 0:08:19      28380000 -- (-7565.122) [...7 remote chains...] -- 0:08:16      28390000 -- [-7543.441] [...7 remote chains...] -- 0:08:13      28400000 -- (-7554.083) [...7 remote chains...] -- 0:08:09       Average standard deviation of split frequencies: 0.005927       28410000 -- (-7561.744) [...7 remote chains...] -- 0:08:06      28420000 -- [-7554.290] [...7 remote chains...] -- 0:08:03      28430000 -- [-7537.417] [...7 remote chains...] -- 0:08:00      28440000 -- [-7562.812] [...7 remote chains...] -- 0:07:57      28450000 -- (-7544.939) [...7 remote chains...] -- 0:07:54       Average standard deviation of split frequencies: 0.005908       28460000 -- [-7546.162] [...7 remote chains...] -- 0:07:51      28470000 -- [-7524.842] [...7 remote chains...] -- 0:07:48      28480000 -- [-7549.279] [...7 remote chains...] -- 0:07:45      28490000 -- [-7562.086] [...7 remote chains...] -- 0:07:42      28500000 -- [-7553.375] [...7 remote chains...] -- 0:07:39       Average standard deviation of split frequencies: 0.005824       28510000 -- [-7555.713] [...7 remote chains...] -- 0:07:36      28520000 -- [-7549.765] [...7 remote chains...] -- 0:07:33      28530000 -- [-7550.337] [...7 remote chains...] -- 0:07:30      28540000 -- (-7556.291) [...7 remote chains...] -- 0:07:27      28550000 -- [-7539.573] [...7 remote chains...] -- 0:07:23       Average standard deviation of split frequencies: 0.005841       28560000 -- (-7560.235) [...7 remote chains...] -- 0:07:20      28570000 -- (-7562.356) [...7 remote chains...] -- 0:07:17      28580000 -- [-7547.532] [...7 remote chains...] -- 0:07:14      28590000 -- (-7561.813) [...7 remote chains...] -- 0:07:11      28600000 -- (-7541.553) [...7 remote chains...] -- 0:07:08       Average standard deviation of split frequencies: 0.005827       28610000 -- [-7540.057] [...7 remote chains...] -- 0:07:05      28620000 -- [-7537.744] [...7 remote chains...] -- 0:07:02      28630000 -- (-7537.881) [...7 remote chains...] -- 0:06:59      28640000 -- (-7562.958) [...7 remote chains...] -- 0:06:56      28650000 -- (-7540.779) [...7 remote chains...] -- 0:06:53       Average standard deviation of split frequencies: 0.005820       28660000 -- (-7589.527) [...7 remote chains...] -- 0:06:50      28670000 -- [-7535.268] [...7 remote chains...] -- 0:06:47      28680000 -- (-7561.985) [...7 remote chains...] -- 0:06:44      28690000 -- [-7570.596] [...7 remote chains...] -- 0:06:41      28700000 -- [-7569.615] [...7 remote chains...] -- 0:06:38       Average standard deviation of split frequencies: 0.005822       28710000 -- [-7547.138] [...7 remote chains...] -- 0:06:34      28720000 -- (-7558.692) [...7 remote chains...] -- 0:06:31      28730000 -- [-7565.300] [...7 remote chains...] -- 0:06:28      28740000 -- (-7550.306) [...7 remote chains...] -- 0:06:25      28750000 -- (-7550.542) [...7 remote chains...] -- 0:06:22       Average standard deviation of split frequencies: 0.005770       28760000 -- (-7541.223) [...7 remote chains...] -- 0:06:19      28770000 -- (-7548.510) [...7 remote chains...] -- 0:06:16      28780000 -- [-7540.933] [...7 remote chains...] -- 0:06:13      28790000 -- [-7549.824] [...7 remote chains...] -- 0:06:10      28800000 -- [-7540.612] [...7 remote chains...] -- 0:06:07       Average standard deviation of split frequencies: 0.005727       28810000 -- [-7548.980] [...7 remote chains...] -- 0:06:04      28820000 -- (-7557.487) [...7 remote chains...] -- 0:06:01      28830000 -- [-7539.211] [...7 remote chains...] -- 0:05:58      28840000 -- [-7552.938] [...7 remote chains...] -- 0:05:55      28850000 -- [-7561.770] [...7 remote chains...] -- 0:05:52       Average standard deviation of split frequencies: 0.005658       28860000 -- [-7557.979] [...7 remote chains...] -- 0:05:49      28870000 -- [-7549.873] [...7 remote chains...] -- 0:05:45      28880000 -- [-7540.971] [...7 remote chains...] -- 0:05:42      28890000 -- [-7536.428] [...7 remote chains...] -- 0:05:39      28900000 -- [-7563.786] [...7 remote chains...] -- 0:05:36       Average standard deviation of split frequencies: 0.005641       28910000 -- [-7541.523] [...7 remote chains...] -- 0:05:33      28920000 -- (-7547.409) [...7 remote chains...] -- 0:05:30      28930000 -- [-7542.755] [...7 remote chains...] -- 0:05:27      28940000 -- [-7551.457] [...7 remote chains...] -- 0:05:24      28950000 -- (-7559.517) [...7 remote chains...] -- 0:05:21       Average standard deviation of split frequencies: 0.005571       28960000 -- (-7556.591) [...7 remote chains...] -- 0:05:18      28970000 -- (-7564.745) [...7 remote chains...] -- 0:05:15      28980000 -- [-7557.041] [...7 remote chains...] -- 0:05:12      28990000 -- [-7531.992] [...7 remote chains...] -- 0:05:09      29000000 -- (-7537.577) [...7 remote chains...] -- 0:05:06       Average standard deviation of split frequencies: 0.005540       29010000 -- (-7549.517) [...7 remote chains...] -- 0:05:03      29020000 -- (-7565.455) [...7 remote chains...] -- 0:05:00      29030000 -- [-7544.995] [...7 remote chains...] -- 0:04:56      29040000 -- [-7575.666] [...7 remote chains...] -- 0:04:53      29050000 -- [-7540.050] [...7 remote chains...] -- 0:04:50       Average standard deviation of split frequencies: 0.005452       29060000 -- [-7562.912] [...7 remote chains...] -- 0:04:47      29070000 -- (-7573.815) [...7 remote chains...] -- 0:04:44      29080000 -- (-7561.401) [...7 remote chains...] -- 0:04:41      29090000 -- (-7581.654) [...7 remote chains...] -- 0:04:38      29100000 -- (-7563.741) [...7 remote chains...] -- 0:04:35       Average standard deviation of split frequencies: 0.005401       29110000 -- (-7533.076) [...7 remote chains...] -- 0:04:32      29120000 -- (-7580.601) [...7 remote chains...] -- 0:04:29      29130000 -- (-7557.425) [...7 remote chains...] -- 0:04:26      29140000 -- (-7558.901) [...7 remote chains...] -- 0:04:23      29150000 -- [-7544.742] [...7 remote chains...] -- 0:04:20       Average standard deviation of split frequencies: 0.005441       29160000 -- (-7552.791) [...7 remote chains...] -- 0:04:17      29170000 -- (-7565.408) [...7 remote chains...] -- 0:04:14      29180000 -- (-7559.508) [...7 remote chains...] -- 0:04:11      29190000 -- (-7537.280) [...7 remote chains...] -- 0:04:07      29200000 -- (-7567.515) [...7 remote chains...] -- 0:04:04       Average standard deviation of split frequencies: 0.005499       29210000 -- (-7541.717) [...7 remote chains...] -- 0:04:01      29220000 -- (-7559.006) [...7 remote chains...] -- 0:03:58      29230000 -- (-7559.964) [...7 remote chains...] -- 0:03:55      29240000 -- (-7557.499) [...7 remote chains...] -- 0:03:52      29250000 -- (-7562.935) [...7 remote chains...] -- 0:03:49       Average standard deviation of split frequencies: 0.005540       29260000 -- (-7554.109) [...7 remote chains...] -- 0:03:46      29270000 -- (-7564.512) [...7 remote chains...] -- 0:03:43      29280000 -- (-7555.313) [...7 remote chains...] -- 0:03:40      29290000 -- (-7561.226) [...7 remote chains...] -- 0:03:37      29300000 -- (-7562.595) [...7 remote chains...] -- 0:03:34       Average standard deviation of split frequencies: 0.005535       29310000 -- (-7566.688) [...7 remote chains...] -- 0:03:31      29320000 -- (-7544.153) [...7 remote chains...] -- 0:03:28      29330000 -- (-7557.949) [...7 remote chains...] -- 0:03:25      29340000 -- (-7547.762) [...7 remote chains...] -- 0:03:22      29350000 -- (-7558.884) [...7 remote chains...] -- 0:03:18       Average standard deviation of split frequencies: 0.005630       29360000 -- (-7558.652) [...7 remote chains...] -- 0:03:15      29370000 -- (-7558.071) [...7 remote chains...] -- 0:03:12      29380000 -- (-7561.799) [...7 remote chains...] -- 0:03:09      29390000 -- (-7546.719) [...7 remote chains...] -- 0:03:06      29400000 -- (-7546.390) [...7 remote chains...] -- 0:03:03       Average standard deviation of split frequencies: 0.005560       29410000 -- (-7547.747) [...7 remote chains...] -- 0:03:00      29420000 -- (-7581.516) [...7 remote chains...] -- 0:02:57      29430000 -- (-7576.616) [...7 remote chains...] -- 0:02:54      29440000 -- (-7576.811) [...7 remote chains...] -- 0:02:51      29450000 -- (-7559.430) [...7 remote chains...] -- 0:02:48       Average standard deviation of split frequencies: 0.005359       29460000 -- (-7557.397) [...7 remote chains...] -- 0:02:45      29470000 -- (-7551.025) [...7 remote chains...] -- 0:02:42      29480000 -- (-7566.052) [...7 remote chains...] -- 0:02:39      29490000 -- (-7556.989) [...7 remote chains...] -- 0:02:36      29500000 -- (-7576.560) [...7 remote chains...] -- 0:02:33       Average standard deviation of split frequencies: 0.005258       29510000 -- (-7552.573) [...7 remote chains...] -- 0:02:29      29520000 -- (-7544.535) [...7 remote chains...] -- 0:02:26      29530000 -- (-7558.280) [...7 remote chains...] -- 0:02:23      29540000 -- (-7558.703) [...7 remote chains...] -- 0:02:20      29550000 -- (-7559.673) [...7 remote chains...] -- 0:02:17       Average standard deviation of split frequencies: 0.005263       29560000 -- (-7570.144) [...7 remote chains...] -- 0:02:14      29570000 -- (-7565.188) [...7 remote chains...] -- 0:02:11      29580000 -- (-7576.191) [...7 remote chains...] -- 0:02:08      29590000 -- (-7574.746) [...7 remote chains...] -- 0:02:05      29600000 -- (-7583.873) [...7 remote chains...] -- 0:02:02       Average standard deviation of split frequencies: 0.005315       29610000 -- (-7575.684) [...7 remote chains...] -- 0:01:59      29620000 -- (-7567.280) [...7 remote chains...] -- 0:01:56      29630000 -- (-7552.273) [...7 remote chains...] -- 0:01:53      29640000 -- (-7569.244) [...7 remote chains...] -- 0:01:50      29650000 -- (-7555.381) [...7 remote chains...] -- 0:01:47       Average standard deviation of split frequencies: 0.005351       29660000 -- (-7543.651) [...7 remote chains...] -- 0:01:44      29670000 -- (-7569.097) [...7 remote chains...] -- 0:01:41      29680000 -- (-7538.641) [...7 remote chains...] -- 0:01:37      29690000 -- (-7564.524) [...7 remote chains...] -- 0:01:34      29700000 -- (-7551.033) [...7 remote chains...] -- 0:01:31       Average standard deviation of split frequencies: 0.005313       29710000 -- (-7559.362) [...7 remote chains...] -- 0:01:28      29720000 -- (-7554.655) [...7 remote chains...] -- 0:01:25      29730000 -- (-7557.708) [...7 remote chains...] -- 0:01:22      29740000 -- (-7560.975) [...7 remote chains...] -- 0:01:19      29750000 -- (-7569.668) [...7 remote chains...] -- 0:01:16       Average standard deviation of split frequencies: 0.005327       29760000 -- (-7567.553) [...7 remote chains...] -- 0:01:13      29770000 -- (-7541.298) [...7 remote chains...] -- 0:01:10      29780000 -- (-7537.239) [...7 remote chains...] -- 0:01:07      29790000 -- (-7545.785) [...7 remote chains...] -- 0:01:04      29800000 -- (-7554.683) [...7 remote chains...] -- 0:01:01       Average standard deviation of split frequencies: 0.005362       29810000 -- (-7550.938) [...7 remote chains...] -- 0:00:58      29820000 -- (-7558.611) [...7 remote chains...] -- 0:00:55      29830000 -- (-7561.774) [...7 remote chains...] -- 0:00:52      29840000 -- (-7584.014) [...7 remote chains...] -- 0:00:48      29850000 -- (-7577.951) [...7 remote chains...] -- 0:00:45       Average standard deviation of split frequencies: 0.005379       29860000 -- (-7565.803) [...7 remote chains...] -- 0:00:42      29870000 -- (-7588.930) [...7 remote chains...] -- 0:00:39      29880000 -- (-7576.091) [...7 remote chains...] -- 0:00:36      29890000 -- (-7574.912) [...7 remote chains...] -- 0:00:33      29900000 -- (-7561.337) [...7 remote chains...] -- 0:00:30       Average standard deviation of split frequencies: 0.005302       29910000 -- (-7527.329) [...7 remote chains...] -- 0:00:27      29920000 -- (-7552.147) [...7 remote chains...] -- 0:00:24      29930000 -- (-7544.401) [...7 remote chains...] -- 0:00:21      29940000 -- (-7546.481) [...7 remote chains...] -- 0:00:18      29950000 -- (-7562.447) [...7 remote chains...] -- 0:00:15       Average standard deviation of split frequencies: 0.005310       29960000 -- (-7550.541) [...7 remote chains...] -- 0:00:12      29970000 -- (-7574.267) [...7 remote chains...] -- 0:00:09      29980000 -- (-7569.047) [...7 remote chains...] -- 0:00:06      29990000 -- (-7561.589) [...7 remote chains...] -- 0:00:03      30000000 -- (-7551.672) [...7 remote chains...] -- 0:00:00       Average standard deviation of split frequencies: 0.005206       Analysis completed in 2 hours 33 mins 2 seconds      Analysis used 9179.29 seconds of CPU time on processor 0      Likelihood of best state for "cold" chain of run 1 was -7501.53      Likelihood of best state for "cold" chain of run 2 was -7508.41       Acceptance rates for the moves in the "cold" chain of run 1:         With prob.   (last 1000)   chain accepted proposals by move            31.4 %     ( 21 %)     Dirichlet(Tratio{4})            24.6 %     ( 25 %)     Dirichlet(Tratio{5})            24.9 %     ( 26 %)     Dirichlet(Pi{4})            26.6 %     ( 26 %)     Slider(Pi{4})            22.0 %     ( 28 %)     Dirichlet(Pi{5})            24.8 %     ( 25 %)     Slider(Pi{5})            32.1 %     ( 20 %)     Multiplier(Alpha{1,2,3})            29.9 %     ( 25 %)     Multiplier(Alpha{4})            25.3 %     ( 23 %)     Multiplier(Alpha{5})            25.0 %     ( 22 %)     Dirichlet(Ratemultiplier{all})            69.6 %     ( 60 %)     Slider(Ratemultiplier{all})             8.3 %     (  7 %)     ExtSPRClock(Tau{all},V{all})            25.3 %     ( 27 %)     NNIClock(Tau{all},V{all})             3.2 %     (  3 %)     ParsSPRClock(Tau{all},V{all})             7.3 %     ( 10 %)     AddBranch(V{all})             7.2 %     (  8 %)     DelBranch(V{all})            47.4 %     ( 52 %)     NodesliderClock(V{all})            34.7 %     ( 21 %)     TreeStretch(V{all})            43.8 %     ( 25 %)     Multiplier(Net_speciation{all})            24.5 %     ( 28 %)     Slider(Relative_extinction{all})            23.0 %     ( 26 %)     Slider(Relative_fossilization{all})            60.9 %     ( 26 %)     Multiplier(ILNvar{1})            59.3 %     ( 25 %)     Multiplier(ILNvar{2})            61.8 %     ( 25 %)     Multiplier(ILNvar{3})            59.2 %     ( 22 %)     Multiplier(ILNvar{4,5})            30.5 %     ( 26 %)     Multiplier(IlnBrlens{1})            30.8 %     ( 14 %)     Multiplier(IlnBrlens{2})            30.9 %     ( 24 %)     Multiplier(IlnBrlens{3})            30.1 %     ( 15 %)     Multiplier(IlnBrlens{4,5})            25.0 %     ( 26 %)     Multiplier(Clockrate{all})       Acceptance rates for the moves in the "cold" chain of run 2:         With prob.   (last 1000)   chain accepted proposals by move            31.4 %     ( 26 %)     Dirichlet(Tratio{4})            24.6 %     ( 23 %)     Dirichlet(Tratio{5})            24.7 %     ( 25 %)     Dirichlet(Pi{4})            26.5 %     ( 25 %)     Slider(Pi{4})            21.9 %     ( 25 %)     Dirichlet(Pi{5})            25.0 %     ( 24 %)     Slider(Pi{5})            31.8 %     ( 23 %)     Multiplier(Alpha{1,2,3})            29.8 %     ( 26 %)     Multiplier(Alpha{4})            25.4 %     ( 26 %)     Multiplier(Alpha{5})            25.0 %     ( 24 %)     Dirichlet(Ratemultiplier{all})            69.9 %     ( 62 %)     Slider(Ratemultiplier{all})             8.3 %     (  8 %)     ExtSPRClock(Tau{all},V{all})            25.3 %     ( 22 %)     NNIClock(Tau{all},V{all})             3.2 %     (  2 %)     ParsSPRClock(Tau{all},V{all})             7.2 %     (  7 %)     AddBranch(V{all})             7.1 %     (  6 %)     DelBranch(V{all})            47.4 %     ( 46 %)     NodesliderClock(V{all})            36.3 %     ( 22 %)     TreeStretch(V{all})            43.7 %     ( 25 %)     Multiplier(Net_speciation{all})            24.6 %     ( 18 %)     Slider(Relative_extinction{all})            23.2 %     ( 20 %)     Slider(Relative_fossilization{all})            60.8 %     ( 24 %)     Multiplier(ILNvar{1})            59.1 %     ( 26 %)     Multiplier(ILNvar{2})            62.0 %     ( 24 %)     Multiplier(ILNvar{3})            59.1 %     ( 21 %)     Multiplier(ILNvar{4,5})            30.5 %     ( 27 %)     Multiplier(IlnBrlens{1})            29.9 %     ( 19 %)     Multiplier(IlnBrlens{2})            31.1 %     ( 20 %)     Multiplier(IlnBrlens{3})            29.8 %     ( 20 %)     Multiplier(IlnBrlens{4,5})            25.1 %     ( 26 %)     Multiplier(Clockrate{all})       Chain swap information for run 1:                     1        2        3        4            --------------------------------------         1 |              0.16     0.01     0.00          2 |  4999218              0.23     0.02          3 |  4998918  4997575              0.28          4 |  5001888  5001690  5000711                 Chain swap information for run 2:                     1        2        3        4            --------------------------------------         1 |              0.18     0.01     0.00          2 |  5002923              0.24     0.02          3 |  4999917  4997287              0.26          4 |  5001555  5000895  4997423                 Upper diagonal: Proportion of successful state exchanges between chains      Lower diagonal: Number of attempted state exchanges between chains       Chain information:         ID -- Heat        -----------         1 -- 1.00  (cold chain)         2 -- 0.93          3 -- 0.88          4 -- 0.83        Heat = 1 / (1 + T * (ID - 1))         (where T = 0.07 is the temperature and ID is the chain number)       Setting sumt output file name to "run.te.maj"      Summarizing trees in files "run.te.run1.t" and "run.te.run2.t"      Using relative burnin ('relburnin=yes'), discarding the first 25 % of sampled trees      Writing statistics to files run.te.maj.<parts|tstat|vstat|trprobs|con>      Examining first file ...      Found one tree block in file "run.te.run1.t" with 75001 trees in last block      Expecting the same number of trees in the last tree block of all files       Tree reading status:       0      10      20      30      40      50      60      70      80      90     100      v-------v-------v-------v-------v-------v-------v-------v-------v-------v-------v      *********************************************************************************       Read a total of 150002 trees in 2 files (sampling 112502 of them)         (Each file contained 75001 trees of which 56251 were sampled)                                                                                         General explanation:                                                                                                                                                   In an unrooted tree, a taxon bipartition (split) is specified by removing a         branch, thereby dividing the species into those to the left and those to the        right of the branch. Here, taxa to one side of the removed branch are denoted       '.' and those to the other side are denoted '*'. Specifically, the '.' symbol       is used for the taxa on the same side as the outgroup.                                                                                                                 In a rooted or clock tree, the tree is rooted using the model and not by            reference to an outgroup. Each bipartition therefore corresponds to a clade,        that is, a group that includes all the descendants of a particular branch in        the tree.  Taxa that are included in each clade are denoted using '*', and          taxa that are not included are denoted using the '.' symbol.                                                                                                           The output first includes a key to all the bipartitions with frequency larger       or equal to (Minpartfreq) in at least one run. Minpartfreq is a parameter to        sumt command and currently it is set to 0.10.  This is followed by a table        with statistics for the informative bipartitions (those including at least          two taxa), sorted from highest to lowest probability. For each bipartition,         the table gives the number of times the partition or split was observed in all      runs (#obs) and the posterior probability of the bipartition (Probab.), which       is the same as the split frequency. If several runs are summarized, this is         followed by the minimum split frequency (Min(s)), the maximum frequency             (Max(s)), and the standard deviation of frequencies (Stddev(s)) across runs.        The latter value should approach 0 for all bipartitions as MCMC runs converge.                                                                                         This is followed by a table summarizing branch lengths, node heights (if a          clock model was used) and relaxed clock parameters (if a relaxed clock model        was used). The mean, variance, and 95 % credible interval are given for each       of these parameters. If several runs are summarized, the potential scale            reduction factor (PSRF) is also given; it should approach 1 as runs converge.       Node heights will take calibration points into account, if such points were         used in the analysis.                                                                                                                                                   Note that Stddev may be unreliable if the partition is not present in all           runs (the last column indicates the number of runs that sampled the partition       if more than one run is summarized). The PSRF is not calculated at all if           the partition is not present in all runs.The PSRF is also sensitive to small        sample sizes and it should only be considered a rough guide to convergence          since some of the assumptions allowing one to interpret it as a true potential      scale reduction factor are violated in MrBayes.                                                                                                                         List of taxa in bipartitions:                                                                                                                                             1 -- Euconodonta         2 -- Jamoytius         3 -- Euphanerops         4 -- Achanarella         5 -- Ciderius         6 -- Cornovichthys         7 -- Lasanius         8 -- Birkenia         9 -- Rhyncholepis        10 -- Myxinikela        11 -- Tethymyxine        12 -- Paramyxine_fernholmi        13 -- Eptatretus_burgeri        14 -- Eptatretus_stoutii        15 -- Myxine_glutinosa        16 -- Rubicundus_eos        17 -- Rubicundus_lopheliae        18 -- Neomyxine_biniplicata        19 -- Myxineidus        20 -- Gilpichthys        21 -- Lethenteron_camtschaticum        22 -- Petromyzon_marinus        23 -- Lampetra_fluviatilis        24 -- Geotria_australis        25 -- Ichthyomyzon_bdellium        26 -- Ichthyomyzon_castaneus        27 -- Ichthyomyzon_unicuspis        28 -- Mordacia_mordax        29 -- Mordacia_lapicida        30 -- Caspiomyzon_wagneri        31 -- Tetrapleurodon_spadiceus        32 -- Entosphenus_macrostomus        33 -- Entosphenus_minimus        34 -- Entosphenus_similis        35 -- Entosphenus_tridentatus        36 -- Eudontomyzon_danfordi        37 -- Eudontomyzon_morii        38 -- Lampetra_ayresii        39 -- Mesomyzon        40 -- Yanliaomyzon_igensdentes        41 -- Yanliaomyzon_occisor        42 -- Priscomyzon        43 -- Mayomyzon        44 -- Hardistiella        45 -- Pipiscius       Key to taxon bipartitions (saved to file "run.te.maj.parts"):        ID -- Partition      ----------------------------------------------------        0 -- *********************************************        1 -- *............................................        2 -- .*...........................................        3 -- ..*..........................................        4 -- ...*.........................................        5 -- ....*........................................        6 -- .....*.......................................        7 -- ......*......................................        8 -- .......*.....................................        9 -- ........*....................................       10 -- .........*...................................       11 -- ..........*..................................       12 -- ...........*.................................       13 -- ............*................................       14 -- .............*...............................       15 -- ..............*..............................       16 -- ...............*.............................       17 -- ................*............................       18 -- .................*...........................       19 -- ..................*..........................       20 -- ...................*.........................       21 -- ....................*........................       22 -- .....................*.......................       23 -- ......................*......................       24 -- .......................*.....................       25 -- ........................*....................       26 -- .........................*...................       27 -- ..........................*..................       28 -- ...........................*.................       29 -- ............................*................       30 -- .............................*...............       31 -- ..............................*..............       32 -- ...............................*.............       33 -- ................................*............       34 -- .................................*...........       35 -- ..................................*..........       36 -- ...................................*.........       37 -- ....................................*........       38 -- .....................................*.......       39 -- ......................................*......       40 -- .......................................*.....       41 -- ........................................*....       42 -- .........................................*...       43 -- ..........................................*..       44 -- ...........................................*.       45 -- ............................................*       46 -- ..........********...........................       47 -- ...........................**................       48 -- ...........***...............................       49 -- .********************************************       50 -- ...............**............................       51 -- ..............*..*...........................       52 -- ....................*********************....       53 -- ....................***.***..*********.......       54 -- ....................******************.......       55 -- ......................*..............*.......       56 -- .......**....................................       57 -- ...................................**........       58 -- ........................***..................       59 -- .........*********...........................       60 -- ....................*************************       61 -- .**..........................................       62 -- ...........****..*...........................       63 -- ...............................*..*..........       64 -- ...........*******...........................       65 -- ....................*.*............***.......       66 -- .....................*..***..................       67 -- .....................*..***..*...............       68 -- ....................*.*.......********.......       69 -- .**...***....................................       70 -- ......***....................................       71 -- ....................***.**************.......       72 -- ...............................****..........       73 -- ...........**................................       74 -- ...***...************************************       75 -- ..............................*****..........       76 -- ....................*********************..*.       77 -- ....................******************.**....       78 -- ...**........................................       79 -- ...............................*.**..........       80 -- ....................******************..*....       81 -- ....................*..............**........       82 -- ........................*.*..................       83 -- ........................**...................       84 -- ....................*********************.**.       85 -- ..................**.........................       86 -- .........************************************       87 -- ...***.......................................       88 -- ....................*********************.***       89 -- ...........*.*...............................       90 -- .......................................**....       91 -- ...*.*.......................................       92 -- ....................*.*..............*.......       93 -- ....................************************.       94 -- .........*********.*.........................       95 -- ....................*******************......       96 -- ......................*............***.......       97 -- .........*********.**************************       98 -- ................................**...........       99 -- ....................**********************...      100 -- .........***********.........................      101 -- .........................**..................      102 -- ..........................................**.      103 -- ..........................................*.*      104 -- ....................*.*.......*....***.......      105 -- ....................*******************.*....      106 -- .....*............*..........................      107 -- ...................**************************      108 -- .**...*......................................      109 -- ...***............*..........................      110 -- ..............................**..*..........      111 -- ......................................***....      112 -- ....................*******..*********.......      113 -- ...............................**.*..........      114 -- ..................***************************      115 -- .........**********..........................      116 -- .......................*...**................      117 -- ....................*.*......*********.......      118 -- ....................*.*.......*****..*.......      119 -- .********....................................      120 -- ..........................................***      121 -- .....*...************************************      122 -- .....................*.......*...............      123 -- ....................**********************.*.      ----------------------------------------------------       Summary statistics for informative taxon bipartitions (clades)         (saved to file "run.te.maj.tstat"):        ID   #obs      Probab.     Sd(s)+      Min(s)      Max(s)   Nruns       -------------------------------------------------------------------       46  112502    1.000000    0.000000    1.000000    1.000000    2       47  112502    1.000000    0.000000    1.000000    1.000000    2       48  112502    1.000000    0.000000    1.000000    1.000000    2       49  112502    1.000000    0.000000    1.000000    1.000000    2       50  112482    0.999822    0.000226    0.999662    0.999982    2       51  112467    0.999689    0.000113    0.999609    0.999769    2       52  112447    0.999511    0.000239    0.999342    0.999680    2       53  112303    0.998231    0.000314    0.998009    0.998453    2       54  112251    0.997769    0.000189    0.997636    0.997902    2       55  112138    0.996765    0.001031    0.996036    0.997493    2       56  111927    0.994889    0.000641    0.994436    0.995342    2       57  111094    0.987485    0.001282    0.986578    0.988391    2       58  105687    0.939423    0.001270    0.938526    0.940321    2       59  105633    0.938943    0.001244    0.938063    0.939823    2       60  103466    0.919681    0.001911    0.918330    0.921033    2       61  102259    0.908953    0.006122    0.904624    0.913282    2       62  100885    0.896740    0.011653    0.888500    0.904979    2       63   98871    0.878838    0.001571    0.877727    0.879949    2       64   97230    0.864251    0.009780    0.857336    0.871167    2       65   93968    0.835256    0.008070    0.829550    0.840963    2       66   91444    0.812821    0.006662    0.808110    0.817532    2       67   90103    0.800901    0.004513    0.797710    0.804092    2       68   87968    0.781924    0.002087    0.780448    0.783399    2       69   85161    0.756973    0.016027    0.745640    0.768306    2       70   84105    0.747587    0.005594    0.743631    0.751542    2       71   81870    0.727720    0.015839    0.716521    0.738920    2       72   77879    0.692245    0.003155    0.690014    0.694477    2       73   76244    0.677712    0.009554    0.670957    0.684468    2       74   75183    0.668281    0.026939    0.649233    0.687330    2       75   68582    0.609607    0.000352    0.609358    0.609856    2       76   67716    0.601909    0.012219    0.593269    0.610549    2       77   63618    0.565483    0.011691    0.557217    0.573750    2       78   53787    0.478098    0.007379    0.472880    0.483316    2       79   52208    0.464063    0.004827    0.460650    0.467476    2       80   46065    0.409459    0.004010    0.406624    0.412295    2       81   45783    0.406953    0.001999    0.405539    0.408366    2       82   43618    0.387709    0.004375    0.384615    0.390802    2       83   42477    0.377567    0.003633    0.374998    0.380135    2       84   41442    0.368367    0.009528    0.361629    0.375104    2       85   37573    0.333976    0.004940    0.330483    0.337470    2       86   37352    0.332012    0.016719    0.320190    0.343834    2       87   37262    0.331212    0.007190    0.326128    0.336296    2       88   35202    0.312901    0.017121    0.300795    0.325008    2       89   35126    0.312226    0.009805    0.305292    0.319159    2       90   34480    0.306483    0.004249    0.303479    0.309488    2       91   34041    0.302581    0.004337    0.299515    0.305648    2       92   32242    0.286590    0.002539    0.284795    0.288386    2       93   31578    0.280688    0.010358    0.273364    0.288013    2       94   28786    0.255871    0.002288    0.254253    0.257489    2       95   27536    0.244760    0.000805    0.244191    0.245329    2       96   27495    0.244396    0.008485    0.238396    0.250396    2       97   26080    0.231818    0.003796    0.229134    0.234502    2       98   25885    0.230085    0.003407    0.227676    0.232494    2       99   25747    0.228858    0.007354    0.223658    0.234058    2      100   25712    0.228547    0.007341    0.223356    0.233738    2      101   24891    0.221249    0.000817    0.220672    0.221827    2      102   21688    0.192779    0.005254    0.189063    0.196494    2      103   21623    0.192201    0.001798    0.190930    0.193472    2      104   20881    0.185606    0.006801    0.180797    0.190414    2      105   20806    0.184939    0.004073    0.182059    0.187819    2      106   20530    0.182486    0.003042    0.180335    0.184637    2      107   20246    0.179961    0.001684    0.178770    0.181152    2      108   18650    0.165775    0.004752    0.162415    0.169135    2      109   17876    0.158895    0.002615    0.157046    0.160744    2      110   17345    0.154175    0.000013    0.154166    0.154184    2      111   16310    0.144975    0.004073    0.142095    0.147855    2      112   15829    0.140700    0.000465    0.140371    0.141029    2      113   15520    0.137953    0.001131    0.137153    0.138753    2      114   15427    0.137126    0.000365    0.136869    0.137384    2      115   15344    0.136389    0.005355    0.132602    0.140175    2      116   14780    0.131375    0.016317    0.119838    0.142913    2      117   14603    0.129802    0.003130    0.127589    0.132015    2      118   13651    0.121340    0.005342    0.117562    0.125118    2      119   13291    0.118140    0.017687    0.105634    0.130647    2      120   13086    0.116318    0.000151    0.116211    0.116425    2      121   13002    0.115571    0.003796    0.112887    0.118256    2      122   12552    0.111571    0.004852    0.108140    0.115002    2      123   11569    0.102834    0.005795    0.098736    0.106931    2      -------------------------------------------------------------------      + Convergence diagnostic (standard deviation of split frequencies)        should approach 0.0 as runs converge.        Summary statistics for branch and node parameters         (saved to file "run.te.maj.vstat"):                                                               95% HPD Interval                                                            --------------------      Parameter                      Mean       Variance     Lower       Upper       Median     PSRF+  Nruns      ------------------------------------------------------------------------------------------------------      length{all}[1]                0.230897    0.018137    0.004061    0.486892    0.207148    1.001    2      length{all}[2]                0.020679    0.002768    0.000000    0.131083    0.000000    1.000    2      length{all}[3]                0.234319    0.006327    0.104474    0.414164    0.221727    1.000    2      length{all}[4]                0.142900    0.006844    0.000000    0.282846    0.137322    1.000    2      length{all}[5]                0.029983    0.004592    0.000000    0.172771    0.000000    1.001    2      length{all}[6]                0.161876    0.018943    0.000000    0.424137    0.132726    1.001    2      length{all}[7]                0.142863    0.005188    0.037558    0.294240    0.128463    1.000    2      length{all}[8]                0.001900    0.000067    0.000000    0.013622    0.000000    1.001    2      length{all}[9]                0.013671    0.000077    0.005672    0.027643    0.011730    1.000    2      length{all}[10]               0.030390    0.010991    0.000000    0.225770    0.000000    1.000    2      length{all}[11]               0.005084    0.000423    0.000000    0.036546    0.000000    1.000    2      length{all}[12]               0.037451    0.000221    0.013325    0.067000    0.034869    1.003    2      length{all}[13]               0.043549    0.000356    0.013542    0.081188    0.040043    1.003    2      length{all}[14]               0.049591    0.000407    0.016912    0.089608    0.046283    1.001    2      length{all}[15]               0.116430    0.001763    0.045184    0.200597    0.110642    1.000    2      length{all}[16]               0.021094    0.000437    0.000186    0.059968    0.014902    1.000    2      length{all}[17]               0.021093    0.000437    0.000186    0.059968    0.014902    1.000    2      length{all}[18]               0.116425    0.001762    0.045181    0.200576    0.110641    1.000    2      length{all}[19]               0.313996    0.032292    0.029841    0.675308    0.281387    1.000    2      length{all}[20]               0.305863    0.030520    0.000000    0.621571    0.286369    1.000    2      length{all}[21]               0.024779    0.000105    0.006442    0.044369    0.023634    1.001    2      length{all}[22]               0.029196    0.000299    0.003883    0.063165    0.025344    1.000    2      length{all}[23]               0.008820    0.000039    0.000372    0.020878    0.007369    1.001    2      length{all}[24]               0.252382    0.005523    0.122709    0.402047    0.241872    1.000    2      length{all}[25]               0.005758    0.000028    0.000020    0.015954    0.004240    1.000    2      length{all}[26]               0.007048    0.000041    0.000045    0.019157    0.005251    1.000    2      length{all}[27]               0.006931    0.000040    0.000021    0.018790    0.005166    1.000    2      length{all}[28]               0.019639    0.000244    0.001287    0.050301    0.015363    1.000    2      length{all}[29]               0.019639    0.000244    0.001287    0.050301    0.015363    1.000    2      length{all}[30]               0.050736    0.000688    0.008712    0.101740    0.046036    1.000    2      length{all}[31]               0.033459    0.000420    0.001636    0.071080    0.030571    1.000    2      length{all}[32]               0.004175    0.000027    0.000000    0.013961    0.002445    1.000    2      length{all}[33]               0.020761    0.000219    0.001638    0.050312    0.016855    1.000    2      length{all}[34]               0.015532    0.000148    0.000326    0.039557    0.012235    1.000    2      length{all}[35]               0.004430    0.000033    0.000000    0.015061    0.002519    1.000    2      length{all}[36]               0.009931    0.000093    0.000246    0.026413    0.007313    1.001    2      length{all}[37]               0.009953    0.000096    0.000246    0.026415    0.007312    1.001    2      length{all}[38]               0.008821    0.000039    0.000360    0.020828    0.007369    1.001    2      length{all}[39]               0.186089    0.011233    0.020573    0.395858    0.167202    1.000    2      length{all}[40]               0.001091    0.000028    0.000000    0.006572    0.000000    1.000    2      length{all}[41]               0.007927    0.000111    0.000000    0.025038    0.000000    1.000    2      length{all}[42]               0.074971    0.004977    0.000000    0.205531    0.065933    1.000    2      length{all}[43]               0.177844    0.008761    0.000000    0.348033    0.164770    1.000    2      length{all}[44]               0.074543    0.009028    0.000000    0.263247    0.033361    1.000    2      length{all}[45]               0.245660    0.013839    0.053903    0.477372    0.228612    1.000    2      length{all}[46]               0.744293    0.044576    0.371517    1.167416    0.713793    1.000    2      length{all}[47]               0.192614    0.004222    0.082982    0.323352    0.182353    1.000    2      length{all}[48]               0.137717    0.002754    0.043029    0.242177    0.132021    1.000    2      length{all}[49]               0.135870    0.011604    0.000002    0.342751    0.112086    1.000    2      length{all}[50]               0.239394    0.004842    0.111861    0.379346    0.232457    1.000    2      length{all}[51]               0.077629    0.001558    0.016745    0.156833    0.069940    1.000    2      length{all}[52]               0.526697    0.034034    0.208518    0.909239    0.502194    1.000    2      length{all}[53]               0.120619    0.002716    0.033170    0.226599    0.113074    1.000    2      length{all}[54]               0.293442    0.019168    0.042534    0.558519    0.275259    1.000    2      length{all}[55]               0.018486    0.000092    0.002348    0.036745    0.017042    1.001    2      length{all}[56]               0.132033    0.007053    0.016420    0.300076    0.112482    1.000    2      length{all}[57]               0.019020    0.000222    0.000864    0.048535    0.015075    1.001    2      length{all}[58]               0.021699    0.000276    0.000000    0.053012    0.017659    1.000    2      length{all}[59]               0.404726    0.044871    0.063049    0.831574    0.371122    1.000    2      length{all}[60]               0.192011    0.012927    0.019739    0.420041    0.168710    1.000    2      length{all}[61]               0.192139    0.010918    0.025258    0.401838    0.174602    1.000    2      length{all}[62]               0.078771    0.002638    0.000016    0.173280    0.068750    1.000    2      length{all}[63]               0.011692    0.000113    0.000004    0.032376    0.008645    1.000    2      length{all}[64]               0.079568    0.004331    0.000001    0.206019    0.064313    1.001    2      length{all}[65]               0.022722    0.000282    0.000551    0.056191    0.018445    1.000    2      length{all}[66]               0.029965    0.000491    0.000000    0.072787    0.024614    1.000    2      length{all}[67]               0.036328    0.000460    0.000003    0.075623    0.033143    1.000    2      length{all}[68]               0.033691    0.000416    0.000030    0.071029    0.030551    1.000    2      length{all}[69]               0.195010    0.012995    0.005455    0.412294    0.175790    1.000    2      length{all}[70]               0.133929    0.009249    0.000001    0.316547    0.113842    1.000    2      length{all}[71]               0.066471    0.002292    0.000007    0.158497    0.055072    1.001    2      length{all}[72]               0.019802    0.000256    0.000000    0.050694    0.015619    1.001    2      length{all}[73]               0.018051    0.000170    0.000649    0.043724    0.014864    1.000    2      length{all}[74]               0.202210    0.015987    0.000332    0.438150    0.181163    1.000    2      length{all}[75]               0.020532    0.000276    0.000000    0.052158    0.016354    1.000    2      length{all}[76]               0.136999    0.006909    0.000740    0.291765    0.123488    1.001    2      length{all}[77]               0.104325    0.007218    0.000004    0.264433    0.084341    1.000    2      length{all}[78]               0.151390    0.011759    0.000012    0.359014    0.128010    1.001    2      length{all}[79]               0.012225    0.000145    0.000002    0.035550    0.008579    1.000    2      length{all}[80]               0.015683    0.000050    0.000000    0.026128    0.015526    1.000    2      length{all}[81]               0.009848    0.000059    0.000001    0.024701    0.008261    1.000    2      length{all}[82]               0.005221    0.000034    0.000000    0.016052    0.003431    1.000    2      length{all}[83]               0.005142    0.000034    0.000000    0.016010    0.003338    1.000    2      length{all}[84]               0.088141    0.004602    0.000004    0.216979    0.074282    1.000    2      length{all}[85]               0.235603    0.024727    0.000008    0.526628    0.211301    1.000    2      length{all}[86]               0.197073    0.022404    0.000018    0.490709    0.161798    1.003    2      length{all}[87]               0.150899    0.012538    0.000010    0.364552    0.127500    1.000    2      length{all}[88]               0.066515    0.002964    0.000004    0.172951    0.054255    1.000    2      length{all}[89]               0.019830    0.000172    0.000011    0.045080    0.017119    1.000    2      length{all}[90]               0.040944    0.001513    0.000003    0.116674    0.030014    1.000    2      length{all}[91]               0.107971    0.007306    0.000004    0.276806    0.086922    1.000    2      length{all}[92]               0.010601    0.000115    0.000000    0.031771    0.007271    1.001    2      length{all}[93]               0.097282    0.010317    0.000007    0.299998    0.065174    1.000    2      length{all}[94]               0.167395    0.017087    0.000002    0.418697    0.139966    1.000    2      length{all}[95]               0.051708    0.001892    0.000006    0.132193    0.041940    1.000    2      length{all}[96]               0.006632    0.000034    0.000001    0.017817    0.005134    1.000    2      length{all}[97]               0.184727    0.020134    0.000010    0.463461    0.152647    1.001    2      length{all}[98]               0.008473    0.000083    0.000002    0.026185    0.005606    1.000    2      length{all}[99]               0.099468    0.004138    0.000027    0.220738    0.087157    1.000    2      length{all}[100]              0.168798    0.018644    0.000020    0.432366    0.138117    1.000    2      length{all}[101]              0.003867    0.000021    0.000000    0.012718    0.002349    1.000    2      length{all}[102]              0.096991    0.005719    0.000004    0.243557    0.079785    1.000    2      length{all}[103]              0.069109    0.003978    0.000002    0.192984    0.051140    1.001    2      length{all}[104]              0.013916    0.000173    0.000002    0.039635    0.010146    1.000    2      length{all}[105]              0.015448    0.000064    0.000015    0.026783    0.015726    1.002    2      length{all}[106]              0.178400    0.016948    0.000005    0.427008    0.150481    1.000    2      length{all}[107]              0.121999    0.010949    0.000002    0.328669    0.095056    1.000    2      length{all}[108]              0.085757    0.005470    0.000013    0.227378    0.067706    1.000    2      length{all}[109]              0.164736    0.015213    0.000024    0.400202    0.140128    1.000    2      length{all}[110]              0.011346    0.000118    0.000009    0.032624    0.008011    1.003    2      length{all}[111]              0.062228    0.003855    0.000011    0.181705    0.043972    1.000    2      length{all}[112]              0.058880    0.002619    0.000015    0.159320    0.046168    1.000    2      length{all}[113]              0.007679    0.000072    0.000000    0.024499    0.005058    1.000    2      length{all}[114]              0.120362    0.009713    0.000098    0.310259    0.096618    1.000    2      length{all}[115]              0.186519    0.020414    0.000049    0.462243    0.156557    1.002    2      length{all}[116]              0.045205    0.001408    0.000008    0.120823    0.035989    1.000    2      length{all}[117]              0.020854    0.000276    0.000006    0.052469    0.017159    1.000    2      length{all}[118]              0.019550    0.000222    0.000033    0.048486    0.015708    1.000    2      length{all}[119]              0.169075    0.010732    0.000167    0.363689    0.151950    1.002    2      length{all}[120]              0.074313    0.004113    0.000006    0.196510    0.058460    1.000    2      length{all}[121]              0.121381    0.011927    0.000003    0.335115    0.090852    1.000    2      length{all}[122]              0.013299    0.000165    0.000001    0.037327    0.009776    1.003    2      length{all}[123]              0.049836    0.002196    0.000007    0.141281    0.036527    1.000    2      height{all}[0]                2.065942    0.266486    1.170176    3.092430    1.990418    1.000    2      height{all}[1]                1.835045    0.219084    1.043306    2.781548    1.765154    1.000    2      height{all}[2]                1.492023    0.144833    0.848283    2.261598    1.435197    1.000    2      height{all}[3]                1.267911    0.104591    0.720865    1.921891    1.219621    1.000    2      height{all}[4]                1.319313    0.113243    0.750089    1.999805    1.269065    1.000    2      height{all}[5]                1.480372    0.142580    0.841658    2.243938    1.423990    1.000    2      height{all}[6]                1.319313    0.113243    0.750089    1.999805    1.269065    1.000    2      height{all}[7]                1.467350    0.140083    0.834255    2.224199    1.411464    1.000    2      height{all}[8]                1.492023    0.144833    0.848283    2.261598    1.435197    1.000    2      height{all}[9]                1.480201    0.142547    0.841561    2.243678    1.423825    1.000    2      height{all}[10]               1.062304    0.073420    0.603968    1.610233    1.021845    1.000    2      height{all}[11]               0.325545    0.006895    0.185087    0.493459    0.313146    1.000    2      height{all}[12]               0.000000    0.000000    0.000000    0.000000    0.000000    1.000    2      height{all}[13]               0.000000    0.000000    0.000000    0.000000    0.000000    1.000    2      height{all}[14]               0.000000    0.000000    0.000000    0.000000    0.000000    1.000    2      height{all}[15]               0.000000    0.000000    0.000000    0.000000    0.000000    1.000    2      height{all}[16]               0.000000    0.000000    0.000000    0.000000    0.000000    1.000    2      height{all}[17]               0.000000    0.000000    0.000000    0.000000    0.000000    1.000    2      height{all}[18]               0.000000    0.000000    0.000000    0.000000    0.000000    1.000    2      height{all}[19]               1.062304    0.073420    0.603968    1.610233    1.021845    1.000    2      height{all}[20]               1.062304    0.073420    0.603968    1.610233    1.021845    1.000    2      height{all}[21]               0.000000    0.000000    0.000000    0.000000    0.000000    1.000    2      height{all}[22]               0.000000    0.000000    0.000000    0.000000    0.000000    1.000    2      height{all}[23]               0.000000    0.000000    0.000000    0.000000    0.000000    1.000    2      height{all}[24]               0.000000    0.000000    0.000000    0.000000    0.000000    1.000    2      height{all}[25]               0.000000    0.000000    0.000000    0.000000    0.000000    1.000    2      height{all}[26]               0.000000    0.000000    0.000000    0.000000    0.000000    1.000    2      height{all}[27]               0.000000    0.000000    0.000000    0.000000    0.000000    1.000    2      height{all}[28]               0.000000    0.000000    0.000000    0.000000    0.000000    1.000    2      height{all}[29]               0.000000    0.000000    0.000000    0.000000    0.000000    1.000    2      height{all}[30]               0.000000    0.000000    0.000000    0.000000    0.000000    1.000    2      height{all}[31]               0.000000    0.000000    0.000000    0.000000    0.000000    1.000    2      height{all}[32]               0.000000    0.000000    0.000000    0.000000    0.000000    1.000    2      height{all}[33]               0.000000    0.000000    0.000000    0.000000    0.000000    1.000    2      height{all}[34]               0.000000    0.000000    0.000000    0.000000    0.000000    1.000    2      height{all}[35]               0.000000    0.000000    0.000000    0.000000    0.000000    1.000    2      height{all}[36]               0.000000    0.000000    0.000000    0.000000    0.000000    1.000    2      height{all}[37]               0.000000    0.000000    0.000000    0.000000    0.000000    1.000    2      height{all}[38]               0.000000    0.000000    0.000000    0.000000    0.000000    1.000    2      height{all}[39]               0.428348    0.011937    0.243535    0.649287    0.412034    1.000    2      height{all}[40]               0.558566    0.020299    0.317570    0.846671    0.537292    1.000    2      height{all}[41]               0.541432    0.019072    0.307829    0.820699    0.520811    1.000    2      height{all}[42]               1.233643    0.099014    0.701382    1.869948    1.186658    1.000    2      height{all}[43]               1.062304    0.073420    0.603968    1.610233    1.021845    1.000    2      height{all}[44]               1.096572    0.078233    0.623451    1.662176    1.054807    1.000    2      height{all}[45]               1.062304    0.073420    0.603968    1.610233    1.021845    1.000    2      height{all}[46]               0.335884    0.007109    0.197146    0.506492    0.322022    1.001    2      height{all}[47]               0.019639    0.000244    0.001287    0.050301    0.015363    1.000    2      height{all}[48]               0.055876    0.000392    0.024433    0.096293    0.052336    1.002    2      height{all}[49]               1.930072    0.228363    1.121930    2.895695    1.858060    1.000    2      height{all}[50]               0.021056    0.000429    0.000096    0.059826    0.014900    1.000    2      height{all}[51]               0.116399    0.001760    0.045333    0.200597    0.110629    1.000    2      height{all}[52]               0.640543    0.030053    0.349966    0.982424    0.613132    1.000    2      height{all}[53]               0.089432    0.000583    0.050648    0.139057    0.085435    1.001    2      height{all}[54]               0.266266    0.005272    0.144253    0.410906    0.255360    1.000    2      height{all}[55]               0.008754    0.000037    0.000471    0.020696    0.007348    1.000    2      height{all}[56]               1.494027    0.144957    0.848516    2.262118    1.437322    1.000    2      height{all}[57]               0.009397    0.000066    0.000246    0.024216    0.007219    1.001    2      height{all}[58]               0.009326    0.000044    0.000700    0.022032    0.007693    1.000    2      height{all}[59]               1.071255    0.076343    0.595616    1.616308    1.029784    1.000    2      height{all}[60]               1.363387    0.113956    0.784617    2.038019    1.313212    1.000    2      height{all}[61]               1.503487    0.148341    0.856606    2.287222    1.446342    1.000    2      height{all}[62]               0.190477    0.002618    0.100838    0.293333    0.184223    1.000    2      height{all}[63]               0.003420    0.000016    0.000000    0.011199    0.002083    1.000    2      height{all}[64]               0.259326    0.004142    0.144463    0.388515    0.251531    1.000    2      height{all}[65]               0.030579    0.000113    0.013024    0.051640    0.028769    1.002    2      height{all}[66]               0.027760    0.000228    0.005912    0.058272    0.024450    1.000    2      height{all}[67]               0.047526    0.000394    0.015198    0.086714    0.044222    1.000    2      height{all}[68]               0.052568    0.000290    0.023828    0.086343    0.049998    1.001    2      height{all}[69]               1.716139    0.186048    0.971878    2.568879    1.651655    1.000    2      height{all}[70]               1.604642    0.164468    0.907292    2.407140    1.543101    1.000    2      height{all}[71]               0.201300    0.002973    0.106607    0.309170    0.193235    1.000    2      height{all}[72]               0.021355    0.000141    0.003674    0.044677    0.018699    1.000    2      height{all}[73]               0.037395    0.000229    0.012533    0.066943    0.034659    1.002    2      height{all}[74]               1.736127    0.181150    0.999873    2.569306    1.672901    1.000    2      height{all}[75]               0.030791    0.000216    0.007601    0.059580    0.028151    1.000    2      height{all}[76]               1.117185    0.082345    0.628800    1.695158    1.073110    1.000    2      height{all}[77]               0.565675    0.020396    0.319704    0.854860    0.543636    1.000    2      height{all}[78]               1.466379    0.140564    0.826439    2.223546    1.411161    1.001    2      height{all}[79]               0.011515    0.000066    0.000670    0.027324    0.009488    1.000    2      height{all}[80]               0.545492    0.018663    0.313206    0.824493    0.524640    1.000    2      height{all}[81]               0.019806    0.000075    0.005471    0.036843    0.018569    1.001    2      height{all}[82]               0.004600    0.000017    0.000019    0.012407    0.003466    1.001    2      height{all}[83]               0.004621    0.000017    0.000039    0.012600    0.003448    1.000    2      height{all}[84]               1.224442    0.095924    0.691638    1.848235    1.177072    1.000    2      height{all}[85]               1.272257    0.109835    0.695221    1.922200    1.221602    1.000    2      height{all}[86]               1.611846    0.152021    0.967480    2.402860    1.551078    1.000    2      height{all}[87]               1.529895    0.149062    0.851985    2.286484    1.473265    1.000    2      height{all}[88]               1.299717    0.100323    0.753522    1.932502    1.255849    1.000    2      height{all}[89]               0.037150    0.000194    0.012516    0.063253    0.034964    1.006    2      height{all}[90]               0.546075    0.020397    0.302988    0.829487    0.524532    1.000    2      height{all}[91]               1.440227    0.130829    0.815522    2.161818    1.384480    1.000    2      height{all}[92]               0.026514    0.000089    0.010515    0.044168    0.025232    1.007    2      height{all}[93]               1.316366    0.112561    0.745711    1.988170    1.263930    1.000    2      height{all}[94]               1.351615    0.121414    0.757991    2.060625    1.297376    1.000    2      height{all}[95]               0.533302    0.019158    0.297532    0.798752    0.513295    1.002    2      height{all}[96]               0.022625    0.000073    0.008033    0.039522    0.021339    1.001    2      height{all}[97]               1.569153    0.153680    0.912899    2.349652    1.506066    1.000    2      height{all}[98]               0.014664    0.000095    0.001618    0.033829    0.012221    1.000    2      height{all}[99]               1.230912    0.103250    0.711045    1.888687    1.182699    1.000    2      height{all}[100]              1.429878    0.127379    0.822261    2.147093    1.376427    1.000    2      height{all}[101]              0.005466    0.000019    0.000135    0.013989    0.004316    1.000    2      height{all}[102]              1.213320    0.100198    0.692965    1.850306    1.162482    1.000    2      height{all}[103]              1.248742    0.097684    0.715806    1.874257    1.204588    1.000    2      height{all}[104]              0.042755    0.000226    0.018044    0.072672    0.040417    1.000    2      height{all}[105]              0.571249    0.020065    0.324011    0.834866    0.551410    1.003    2      height{all}[106]              1.368201    0.127202    0.756484    2.084418    1.317961    1.000    2      height{all}[107]              1.480188    0.131687    0.853185    2.216389    1.427128    1.000    2      height{all}[108]              1.617915    0.166821    0.919001    2.428177    1.559530    1.001    2      height{all}[109]              1.562940    0.164385    0.853208    2.362687    1.503761    1.000    2      height{all}[110]              0.013809    0.000093    0.001233    0.033142    0.011370    1.000    2      height{all}[111]              0.556820    0.021269    0.309884    0.851778    0.533164    1.000    2      height{all}[112]              0.221235    0.003296    0.123601    0.328966    0.214193    1.001    2      height{all}[113]              0.014179    0.000085    0.001745    0.032518    0.011892    1.000    2      height{all}[114]              1.497179    0.135523    0.858435    2.212063    1.444569    1.000    2      height{all}[115]              1.329129    0.113943    0.731059    1.977709    1.282679    1.000    2      height{all}[116]              0.200479    0.003459    0.099106    0.321076    0.193585    1.001    2      height{all}[117]              0.068888    0.000428    0.030933    0.109756    0.066450    1.002    2      height{all}[118]              0.039593    0.000191    0.017355    0.067482    0.036852    1.001    2      height{all}[119]              1.755417    0.180405    0.997933    2.582788    1.693728    1.001    2      height{all}[120]              1.295867    0.107009    0.751405    1.951319    1.244922    1.000    2      height{all}[121]              1.664141    0.161186    0.987112    2.490860    1.599117    1.000    2      height{all}[122]              0.029844    0.000243    0.005997    0.061407    0.026732    1.001    2      height{all}[123]              1.283070    0.102372    0.740970    1.932439    1.238368    1.000    2      age{all}[0]                 604.922016  1586.564804  535.553387  680.241385  598.638267    1.002    2      age{all}[1]                 535.500020    0.000000  535.499919  535.500123  535.500020    1.000    2      age{all}[2]                 435.400020    0.000000  435.399931  435.400110  435.400020    1.000    2      age{all}[3]                 370.000020    0.000000  369.999937  370.000103  370.000020    1.000    2      age{all}[4]                 385.000020    0.000000  384.999940  385.000102  385.000019    1.000    2      age{all}[5]                 432.000020    0.000000  431.999935  432.000106  432.000020    1.000    2      age{all}[6]                 385.000020    0.000000  384.999941  385.000101  385.000019    1.000    2      age{all}[7]                 428.200020    0.000000  428.199931  428.200108  428.200019    1.000    2      age{all}[8]                 435.400020    0.000000  435.399931  435.400109  435.400019    1.000    2      age{all}[9]                 431.950020    0.000000  431.949932  431.950109  431.950019    1.000    2      age{all}[10]                310.000020    0.000000  309.999956  310.000088  310.000019    1.000    2      age{all}[11]                 95.000020    0.000000   94.999985   95.000066   95.000016    1.000    2      age{all}[12]                  0.000020    0.000000    0.000000    0.000062    0.000014    1.000    2      age{all}[13]                  0.000020    0.000000    0.000000    0.000062    0.000014    1.000    2      age{all}[14]                  0.000020    0.000000    0.000000    0.000062    0.000014    1.000    2      age{all}[15]                  0.000020    0.000000    0.000000    0.000062    0.000015    1.000    2      age{all}[16]                  0.000020    0.000000    0.000000    0.000062    0.000015    1.000    2      age{all}[17]                  0.000020    0.000000    0.000000    0.000062    0.000015    1.000    2      age{all}[18]                  0.000020    0.000000    0.000000    0.000062    0.000015    1.000    2      age{all}[19]                310.000020    0.000000  309.999950  310.000092  310.000019    1.000    2      age{all}[20]                310.000020    0.000000  309.999952  310.000090  310.000019    1.000    2      age{all}[21]                  0.000020    0.000000    0.000000    0.000061    0.000014    1.000    2      age{all}[22]                  0.000020    0.000000    0.000000    0.000061    0.000014    1.000    2      age{all}[23]                  0.000020    0.000000    0.000000    0.000061    0.000014    1.000    2      age{all}[24]                  0.000020    0.000000    0.000000    0.000061    0.000015    1.000    2      age{all}[25]                  0.000020    0.000000    0.000000    0.000061    0.000014    1.000    2      age{all}[26]                  0.000020    0.000000    0.000000    0.000061    0.000014    1.000    2      age{all}[27]                  0.000020    0.000000    0.000000    0.000061    0.000014    1.000    2      age{all}[28]                  0.000020    0.000000    0.000000    0.000062    0.000015    1.000    2      age{all}[29]                  0.000020    0.000000    0.000000    0.000062    0.000015    1.000    2      age{all}[30]                  0.000020    0.000000    0.000000    0.000061    0.000014    1.000    2      age{all}[31]                  0.000020    0.000000    0.000000    0.000061    0.000014    1.000    2      age{all}[32]                  0.000020    0.000000    0.000000    0.000060    0.000014    1.000    2      age{all}[33]                  0.000020    0.000000    0.000000    0.000060    0.000014    1.000    2      age{all}[34]                  0.000020    0.000000    0.000000    0.000060    0.000014    1.000    2      age{all}[35]                  0.000020    0.000000    0.000000    0.000060    0.000014    1.000    2      age{all}[36]                  0.000020    0.000000    0.000000    0.000060    0.000014    1.000    2      age{all}[37]                  0.000020    0.000000    0.000000    0.000060    0.000014    1.000    2      age{all}[38]                  0.000020    0.000000    0.000000    0.000061    0.000014    1.000    2      age{all}[39]                125.000020    0.000000  124.999976  125.000069  125.000018    1.000    2      age{all}[40]                163.000020    0.000000  162.999974  163.000069  163.000018    1.000    2      age{all}[41]                158.000020    0.000000  157.999975  158.000068  158.000018    1.000    2      age{all}[42]                360.000020    0.000000  359.999949  360.000095  360.000019    1.000    2      age{all}[43]                310.000020    0.000000  309.999953  310.000090  310.000019    1.000    2      age{all}[44]                320.000020    0.000000  319.999954  320.000090  320.000019    1.000    2      age{all}[45]                310.000020    0.000000  309.999954  310.000092  310.000019    1.000    2      age{all}[46]                 98.550997   94.286352   94.999970  119.168785   95.000022    1.000    2      age{all}[47]                  5.907731   22.081822    0.388212   15.263551    4.598273    1.000    2      age{all}[48]                 16.930560   38.846171    6.495838   29.495135   15.888508    1.002    2      age{all}[49]                565.545650  1476.493904  496.457013  642.509688  561.899966    1.002    2      age{all}[50]                  6.370155   39.884838    0.033957   18.281932    4.514850    1.000    2      age{all}[51]                 35.541257  204.940088   11.771068   62.837297   33.461798    1.000    2      age{all}[52]                187.754362  604.909690  162.999947  236.122921  182.092818    1.000    2      age{all}[53]                 27.125613   64.614648   13.465493   43.583233   25.855064    1.001    2      age{all}[54]                 80.526193  534.404118   39.863055  126.906975   77.920768    1.000    2      age{all}[55]                  2.649552    3.477914    0.119346    6.281899    2.213898    1.001    2      age{all}[56]                435.982638    6.240101  435.399816  439.578260  435.400027    1.001    2      age{all}[57]                  2.837893    6.051057    0.079991    7.355942    2.167693    1.001    2      age{all}[58]                  2.813717    4.086346    0.162864    6.634216    2.311812    1.000    2      age{all}[59]                312.884817  154.210090  309.999884  329.981693  310.000024    1.000    2      age{all}[60]                400.495189  576.240366  359.999925  444.326122  397.718013    1.001    2      age{all}[61]                439.505833  127.087913  435.399837  463.006935  435.400039    1.000    2      age{all}[62]                 57.346343  255.458809   28.973296   88.020969   55.994354    1.000    2      age{all}[63]                  1.032720    1.494617    0.000012    3.387033    0.628863    1.000    2      age{all}[64]                 75.085549  203.577795   46.419711   94.999446   76.642804    1.001    2      age{all}[65]                  9.316673   12.417697    3.489374   16.261234    8.764578    1.002    2      age{all}[66]                  8.401282   21.084269    1.514335   17.569499    7.386597    1.000    2      age{all}[67]                 14.418791   39.337706    4.379502   27.118451   13.269401    1.001    2      age{all}[68]                 16.004332   31.273685    6.660585   27.348108   15.119710    1.001    2      age{all}[69]                502.374875  721.468151  457.206752  555.672608  498.234531    1.000    2      age{all}[70]                468.176340  320.747048  440.930619  504.008051  464.761808    1.000    2      age{all}[71]                 60.649451  340.117152   27.889700   96.423824   58.406726    1.000    2      age{all}[72]                  6.502159   13.865400    1.099400   13.973698    5.642207    1.000    2      age{all}[73]                 11.311854   23.179869    3.463660   20.281152   10.578138    1.002    2      age{all}[74]                503.694970  1267.268379  441.032416  574.287625  499.632545    1.001    2      age{all}[75]                  9.323443   21.081907    2.079933   18.400612    8.432360    1.000    2      age{all}[76]                326.745698  202.440278  319.999884  359.044475  320.000040    1.000    2      age{all}[77]                166.558299   59.972676  162.999941  183.236647  163.000036    1.000    2      age{all}[78]                435.932612  112.054936  431.999850  457.294034  432.000041    1.001    2      age{all}[79]                  3.495330    6.121522    0.260633    8.360577    2.874952    1.000    2      age{all}[80]                158.577615    2.010016  157.999943  161.985875  158.000025    1.000    2      age{all}[81]                  6.031538    7.465221    1.377405   11.308275    5.613361    1.001    2      age{all}[82]                  1.391820    1.555610    0.006651    3.758219    1.043446    1.001    2      age{all}[83]                  1.395071    1.597198    0.006535    3.779221    1.034734    1.000    2      age{all}[84]                356.716633  410.539451  319.999940  393.265525  354.031434    1.002    2      age{all}[85]                372.812638  1161.149853  318.899349  439.672507  367.039804    1.001    2      age{all}[86]                462.514685  1540.540083  394.405733  542.837413  456.974373    1.002    2      age{all}[87]                449.946007  585.378554  431.999851  499.501701  438.895671    1.001    2      age{all}[88]                371.140701  481.309591  332.094667  411.944423  370.185996    1.004    2      age{all}[89]                 11.423237   21.235250    3.532939   20.452297   10.739783    1.005    2      age{all}[90]                163.443986    3.105738  162.999922  166.028194  163.000023    1.000    2      age{all}[91]                415.862917  274.688193  389.499294  448.130356  413.348028    1.000    2      age{all}[92]                  7.993887    9.089428    3.071160   14.228019    7.555110    1.008    2      age{all}[93]                389.565571  378.837228  359.999916  425.079848  387.377050    1.000    2      age{all}[94]                395.709437  1407.996921  329.989085  468.341077  391.427930    1.000    2      age{all}[95]                150.816821  157.234563  124.999972  174.127942  149.824589    1.000    2      age{all}[96]                  6.924888    7.712364    2.241001   12.501524    6.518741    1.002    2      age{all}[97]                453.537254  1334.104217  391.774480  527.350880  448.757436    1.001    2      age{all}[98]                  4.431628    8.887327    0.390418   10.288143    3.690299    1.001    2      age{all}[99]                365.534401  137.819635  359.999897  391.536884  360.000042    1.000    2      age{all}[100]               415.893010  1443.852627  345.605780  489.194571  411.946503    1.001    2      age{all}[101]                 1.652146    1.792590    0.036739    4.272104    1.293366    1.000    2      age{all}[102]               354.542860  429.926088  319.999919  392.018225  352.215062    1.000    2      age{all}[103]               361.318950  490.272450  322.215803  403.261842  359.075258    1.001    2      age{all}[104]                13.103041   25.646357    4.622084   23.481190   12.325891    1.000    2      age{all}[105]               159.042320    3.693280  157.999951  162.590650  158.000035    1.000    2      age{all}[106]               403.550187  806.451205  384.999866  463.632908  385.000081    1.000    2      age{all}[107]               438.933993  1029.705144  381.654766  500.545336  434.957955    1.000    2      age{all}[108]               474.806718  372.765354  442.956035  512.761949  471.477814    1.000    2      age{all}[109]               460.111383  824.711902  431.999871  515.322889  453.474309    1.000    2      age{all}[110]                 4.136285    8.488528    0.360453    9.833819    3.415009    1.000    2      age{all}[111]               169.234763   93.802751  162.999942  189.256821  163.801121    1.001    2      age{all}[112]                68.746352  448.841961   34.376554  109.634495   65.788755    1.001    2      age{all}[113]                 4.296821    8.112864    0.452276    9.992890    3.596548    1.000    2      age{all}[114]               447.575279  1088.511909  389.423894  514.392708  443.549935    1.005    2      age{all}[115]               387.384419  1344.256537  326.482929  459.108937  381.930813    1.001    2      age{all}[116]                63.048051  457.147835   26.674259  109.297999   60.419720    1.003    2      age{all}[117]                20.715159   45.199284    9.631183   33.887063   19.724097    1.000    2      age{all}[118]                11.824480   17.717910    4.705079   20.199871   11.035633    1.002    2      age{all}[119]               518.981307  943.021833  464.980633  579.461029  514.902304    1.004    2      age{all}[120]               375.660441  438.389815  334.973219  415.315326  373.789898    1.000    2      age{all}[121]               474.399671  1319.141854  411.982099  551.192570  469.918369    1.005    2      age{all}[122]                 8.881867   21.617824    1.634251   18.322065    7.918888    1.001    2      age{all}[123]               383.572043  302.827957  359.999905  414.621262  381.688951    1.000    2      IlnBrlens{1}_length[1]        0.500937    0.320051    0.000019    1.502505    0.340735    1.000    2      IlnBrlens{1}_length[2]        0.010395    0.001118    0.000000    0.062768    0.000000    1.000    2      IlnBrlens{1}_length[3]        0.154832    0.015060    0.008853    0.391330    0.123043    1.000    2      IlnBrlens{1}_length[4]        0.132059    0.019823    0.000000    0.386506    0.091798    1.000    2      IlnBrlens{1}_length[5]        0.015611    0.001940    0.000000    0.089481    0.000000    1.001    2      IlnBrlens{1}_length[6]        0.134765    0.030159    0.000000    0.455437    0.079201    1.001    2      IlnBrlens{1}_length[7]        0.065478    0.004265    0.000000    0.188567    0.046241    1.000    2      IlnBrlens{1}_length[8]        0.001169    0.000030    0.000000    0.007553    0.000000    1.000    2      IlnBrlens{1}_length[9]        0.010972    0.000098    0.000000    0.029045    0.008267    1.000    2      IlnBrlens{1}_length[10]       0.050227    0.065317    0.000000    0.292756    0.000000    1.000    2      IlnBrlens{1}_length[11]       0.004211    0.000419    0.000000    0.023288    0.000000    1.000    2      IlnBrlens{1}_length[12]       0.023553    0.000413    0.000436    0.063077    0.017920    1.001    2      IlnBrlens{1}_length[13]       0.019423    0.000347    0.000221    0.054218    0.014034    1.002    2      IlnBrlens{1}_length[14]       0.026408    0.000551    0.000502    0.070542    0.020003    1.001    2      IlnBrlens{1}_length[15]       0.050266    0.001718    0.000938    0.129185    0.039093    1.002    2      IlnBrlens{1}_length[16]       0.013705    0.000276    0.000002    0.045230    0.008346    1.000    2      IlnBrlens{1}_length[17]       0.015293    0.000348    0.000011    0.049869    0.009308    1.000    2      IlnBrlens{1}_length[18]       0.046877    0.001510    0.001189    0.121091    0.036708    1.000    2      IlnBrlens{1}_length[19]       0.447258    0.227615    0.000000    1.278400    0.314609    1.000    2      IlnBrlens{1}_length[20]       0.231807    0.063644    0.000000    0.671081    0.160869    1.000    2      IlnBrlens{1}_length[21]       0.015423    0.000182    0.000130    0.041470    0.011663    1.000    2      IlnBrlens{1}_length[22]       0.026278    0.000440    0.000703    0.066816    0.020791    1.000    2      IlnBrlens{1}_length[23]       0.007985    0.000061    0.000041    0.022759    0.005689    1.000    2      IlnBrlens{1}_length[24]       0.135429    0.009775    0.005135    0.325266    0.112365    1.001    2      IlnBrlens{1}_length[25]       0.003524    0.000022    0.000001    0.012001    0.001978    1.000    2      IlnBrlens{1}_length[26]       0.004237    0.000030    0.000004    0.014182    0.002447    1.000    2      IlnBrlens{1}_length[27]       0.004068    0.000028    0.000002    0.013630    0.002346    1.000    2      IlnBrlens{1}_length[28]       0.021534    0.000541    0.000037    0.065226    0.014425    1.000    2      IlnBrlens{1}_length[29]       0.016085    0.000321    0.000033    0.049299    0.010565    1.000    2      IlnBrlens{1}_length[30]       0.019709    0.000337    0.000183    0.054314    0.014539    1.001    2      IlnBrlens{1}_length[31]       0.013987    0.000198    0.000062    0.040827    0.009719    1.000    2      IlnBrlens{1}_length[32]       0.002906    0.000024    0.000000    0.011135    0.001245    1.001    2      IlnBrlens{1}_length[33]       0.011256    0.000131    0.000075    0.032922    0.007775    1.001    2      IlnBrlens{1}_length[34]       0.014877    0.000257    0.000020    0.044904    0.009909    1.000    2      IlnBrlens{1}_length[35]       0.002914    0.000022    0.000000    0.011211    0.001272    1.000    2      IlnBrlens{1}_length[36]       0.006252    0.000059    0.000006    0.019971    0.003835    1.000    2      IlnBrlens{1}_length[37]       0.010023    0.000145    0.000012    0.031324    0.006304    1.000    2      IlnBrlens{1}_length[38]       0.005477    0.000041    0.000005    0.017265    0.003426    1.000    2      IlnBrlens{1}_length[39]       0.200352    0.027942    0.000000    0.515630    0.156744    1.000    2      IlnBrlens{1}_length[40]       0.000562    0.000012    0.000000    0.002488    0.000000    1.000    2      IlnBrlens{1}_length[41]       0.004531    0.000079    0.000000    0.020791    0.000000    1.000    2      IlnBrlens{1}_length[42]       0.119773    0.024840    0.000000    0.402336    0.075824    1.000    2      IlnBrlens{1}_length[43]       0.160947    0.023424    0.000000    0.438937    0.119096    1.000    2      IlnBrlens{1}_length[44]       0.120905    0.039913    0.000000    0.501785    0.028960    1.000    2      IlnBrlens{1}_length[45]       0.127508    0.016087    0.000000    0.362095    0.090744    1.000    2      IlnBrlens{1}_length[46]       4.107029   27.628795    0.170046   12.215980    2.573029    1.000    2      IlnBrlens{1}_length[47]       0.126984    0.008150    0.003305    0.297351    0.106216    1.000    2      IlnBrlens{1}_length[48]       0.054262    0.002251    0.000953    0.146149    0.041178    1.000    2      IlnBrlens{1}_length[49]       0.265063    0.172296    0.000001    1.010754    0.113705    1.000    2      IlnBrlens{1}_length[50]       0.088010    0.004949    0.002296    0.224997    0.069152    1.000    2      IlnBrlens{1}_length[51]       0.068034    0.003334    0.001631    0.178256    0.052376    1.000    2      IlnBrlens{1}_length[52]       0.667000    0.282739    0.030163    1.660986    0.529365    1.000    2      IlnBrlens{1}_length[53]       0.083524    0.004769    0.001081    0.215696    0.065246    1.000    2      IlnBrlens{1}_length[54]       0.394894    0.089633    0.013076    0.954324    0.319532    1.000    2      IlnBrlens{1}_length[55]       0.008578    0.000070    0.000048    0.024501    0.006082    1.001    2      IlnBrlens{1}_length[56]       0.189151    0.027931    0.001602    0.500228    0.141648    1.000    2      IlnBrlens{1}_length[57]       0.008912    0.000104    0.000045    0.027807    0.005699    1.000    2      IlnBrlens{1}_length[58]       0.022935    0.000478    0.000000    0.064813    0.016759    1.000    2      IlnBrlens{1}_length[59]       3.190556   30.112116    0.000170   11.533750    1.481186    1.000    2      IlnBrlens{1}_length[60]       0.160615    0.051481    0.000032    0.542978    0.089461    1.000    2      IlnBrlens{1}_length[61]       0.126019    0.017291    0.000002    0.375188    0.086216    1.000    2      IlnBrlens{1}_length[62]       0.060140    0.003126    0.000000    0.167747    0.044466    1.000    2      IlnBrlens{1}_length[63]       0.007519    0.000081    0.000000    0.024572    0.004567    1.000    2      IlnBrlens{1}_length[64]       0.047047    0.003434    0.000000    0.157177    0.027614    1.000    2      IlnBrlens{1}_length[65]       0.012749    0.000170    0.000001    0.037562    0.008857    1.001    2      IlnBrlens{1}_length[66]       0.020743    0.000431    0.000000    0.060089    0.014764    1.000    2      IlnBrlens{1}_length[67]       0.015147    0.000251    0.000002    0.044347    0.010425    1.001    2      IlnBrlens{1}_length[68]       0.015015    0.000233    0.000003    0.044130    0.010337    1.000    2      IlnBrlens{1}_length[69]       0.210393    0.060191    0.000012    0.670440    0.131673    1.000    2      IlnBrlens{1}_length[70]       0.097617    0.015118    0.000001    0.323122    0.058516    1.000    2      IlnBrlens{1}_length[71]       0.042922    0.002352    0.000003    0.134812    0.027514    1.000    2      IlnBrlens{1}_length[72]       0.009673    0.000113    0.000000    0.029870    0.006287    1.001    2      IlnBrlens{1}_length[73]       0.012137    0.000201    0.000017    0.038841    0.007554    1.000    2      IlnBrlens{1}_length[74]       0.235371    0.072252    0.000004    0.699315    0.159083    1.000    2      IlnBrlens{1}_length[75]       0.009122    0.000105    0.000000    0.028185    0.005892    1.001    2      IlnBrlens{1}_length[76]       0.322237    0.113980    0.000015    0.928051    0.227247    1.000    2      IlnBrlens{1}_length[77]       0.074429    0.010070    0.000004    0.252060    0.042391    1.000    2      IlnBrlens{1}_length[78]       0.130465    0.023897    0.000004    0.417701    0.080953    1.000    2      IlnBrlens{1}_length[79]       0.008927    0.000102    0.000000    0.027882    0.005715    1.001    2      IlnBrlens{1}_length[80]       0.010617    0.000144    0.000000    0.032897    0.006869    1.000    2      IlnBrlens{1}_length[81]       0.007330    0.000084    0.000000    0.024426    0.004267    1.000    2      IlnBrlens{1}_length[82]       0.003219    0.000022    0.000000    0.011703    0.001620    1.001    2      IlnBrlens{1}_length[83]       0.003309    0.000025    0.000000    0.012202    0.001613    1.000    2      IlnBrlens{1}_length[84]       0.101698    0.024887    0.000003    0.357566    0.052053    1.001    2      IlnBrlens{1}_length[85]       0.193259    0.059819    0.000001    0.632809    0.116643    1.000    2      IlnBrlens{1}_length[86]       0.228000    0.072392    0.000003    0.722872    0.145141    1.001    2      IlnBrlens{1}_length[87]       0.138583    0.032819    0.000008    0.471315    0.079757    1.000    2      IlnBrlens{1}_length[88]       0.064409    0.008450    0.000001    0.231979    0.033171    1.001    2      IlnBrlens{1}_length[89]       0.011611    0.000183    0.000001    0.036926    0.007225    1.000    2      IlnBrlens{1}_length[90]       0.032851    0.002462    0.000000    0.119447    0.016153    1.000    2      IlnBrlens{1}_length[91]       0.081301    0.011727    0.000001    0.274413    0.046873    1.000    2      IlnBrlens{1}_length[92]       0.005446    0.000051    0.000000    0.019025    0.003026    1.000    2      IlnBrlens{1}_length[93]       0.118773    0.057584    0.000002    0.493643    0.041216    1.000    2      IlnBrlens{1}_length[94]       0.198948    0.260650    0.000001    0.714510    0.076564    1.000    2      IlnBrlens{1}_length[95]       0.056063    0.006763    0.000003    0.202960    0.027533    1.000    2      IlnBrlens{1}_length[96]       0.003944    0.000025    0.000000    0.013352    0.002246    1.000    2      IlnBrlens{1}_length[97]       0.213070    0.065166    0.000002    0.677674    0.133935    1.000    2      IlnBrlens{1}_length[98]       0.004782    0.000045    0.000001    0.016562    0.002558    1.001    2      IlnBrlens{1}_length[99]       0.227584    0.069903    0.000022    0.714138    0.148011    1.000    2      IlnBrlens{1}_length[100]      0.245149    0.507028    0.000014    0.893803    0.085797    1.000    2      IlnBrlens{1}_length[101]      0.002508    0.000015    0.000000    0.009630    0.001145    1.000    2      IlnBrlens{1}_length[102]      0.089384    0.016916    0.000004    0.303402    0.048393    1.000    2      IlnBrlens{1}_length[103]      0.045362    0.003846    0.000000    0.156058    0.024558    1.001    2      IlnBrlens{1}_length[104]      0.008865    0.000120    0.000000    0.030117    0.005122    1.000    2      IlnBrlens{1}_length[105]      0.011325    0.000204    0.000000    0.037384    0.006697    1.000    2      IlnBrlens{1}_length[106]      0.159196    0.046585    0.000003    0.535267    0.091594    1.000    2      IlnBrlens{1}_length[107]      0.147334    0.041047    0.000001    0.520428    0.079158    1.000    2      IlnBrlens{1}_length[108]      0.075611    0.011695    0.000003    0.267485    0.039874    1.000    2      IlnBrlens{1}_length[109]      0.133847    0.029392    0.000003    0.444486    0.079118    1.000    2      IlnBrlens{1}_length[110]      0.005981    0.000054    0.000001    0.020006    0.003466    1.001    2      IlnBrlens{1}_length[111]      0.095211    0.021974    0.000009    0.339759    0.048728    1.000    2      IlnBrlens{1}_length[112]      0.034694    0.002025    0.000004    0.117209    0.019697    1.002    2      IlnBrlens{1}_length[113]      0.005887    0.000060    0.000000    0.020707    0.003250    1.000    2      IlnBrlens{1}_length[114]      0.142344    0.037758    0.000026    0.481689    0.080103    1.000    2      IlnBrlens{1}_length[115]      0.743489    5.978453    0.000011    2.923191    0.223569    1.002    2      IlnBrlens{1}_length[116]      0.042539    0.003025    0.000001    0.147196    0.023638    1.000    2      IlnBrlens{1}_length[117]      0.014471    0.000270    0.000001    0.046182    0.009062    1.001    2      IlnBrlens{1}_length[118]      0.008770    0.000090    0.000009    0.026894    0.005624    1.000    2      IlnBrlens{1}_length[119]      0.299345    0.104474    0.000015    0.921026    0.194860    1.000    2      IlnBrlens{1}_length[120]      0.048569    0.004247    0.000004    0.166108    0.026877    1.000    2      IlnBrlens{1}_length[121]      0.121948    0.032790    0.000002    0.449700    0.058805    1.000    2      IlnBrlens{1}_length[122]      0.007352    0.000089    0.000000    0.025439    0.004179    1.003    2      IlnBrlens{1}_length[123]      0.069590    0.018080    0.000005    0.306534    0.024038    1.000    2      IlnBrlens{1}_rate[1]          2.430049    8.448816    0.014700    7.338215    1.567105    1.000    2      IlnBrlens{1}_rate[2]          0.877814    0.112550    0.104361    1.000682    1.000000    1.000    2      IlnBrlens{1}_rate[3]          0.696927    0.358441    0.046209    1.724795    0.549355    1.000    2      IlnBrlens{1}_rate[4]          1.059349    1.379208    0.011369    2.996621    0.771178    1.000    2      IlnBrlens{1}_rate[5]          0.860524    0.126879    0.044948    1.000597    1.000000    1.000    2      IlnBrlens{1}_rate[6]          0.965467    1.058132    0.005123    2.595226    0.778028    1.000    2      IlnBrlens{1}_rate[7]          0.495206    0.211625    0.008986    1.343852    0.362896    1.000    2      IlnBrlens{1}_rate[8]          0.974213    0.035422    0.370334    1.000242    1.000000    1.000    2      IlnBrlens{1}_rate[9]          0.864511    0.512030    0.024647    2.245427    0.679464    1.000    2      IlnBrlens{1}_rate[10]         1.088708    0.526753    0.102995    1.689577    1.000000    1.000    2      IlnBrlens{1}_rate[11]         0.994611    0.087928    0.097351    1.000000    1.000000    1.000    2      IlnBrlens{1}_rate[12]         0.670064    0.330484    0.016815    1.768395    0.516276    1.000    2      IlnBrlens{1}_rate[13]         0.472719    0.182142    0.006074    1.278501    0.355106    1.000    2      IlnBrlens{1}_rate[14]         0.576044    0.266922    0.009227    1.512607    0.437009    1.000    2      IlnBrlens{1}_rate[15]         0.463701    0.146868    0.011246    1.203799    0.362028    1.002    2      IlnBrlens{1}_rate[16]         0.829806    0.865925    0.002888    2.435184    0.570580    1.000    2      IlnBrlens{1}_rate[17]         0.941479    1.264900    0.017664    2.727172    0.631723    1.000    2      IlnBrlens{1}_rate[18]         0.433831    0.132014    0.010044    1.128560    0.336764    1.000    2      IlnBrlens{1}_rate[19]         1.575000    2.682199    0.027426    4.318033    1.133803    1.000    2      IlnBrlens{1}_rate[20]         0.840866    0.824186    0.009958    2.272250    0.610968    1.000    2      IlnBrlens{1}_rate[21]         0.659116    0.320065    0.024643    1.728687    0.505859    1.000    2      IlnBrlens{1}_rate[22]         1.082638    0.997206    0.019550    2.862214    0.811520    1.000    2      IlnBrlens{1}_rate[23]         1.091057    1.189649    0.018188    3.010448    0.791314    1.000    2      IlnBrlens{1}_rate[24]         0.560508    0.166557    0.014732    1.334530    0.467257    1.001    2      IlnBrlens{1}_rate[25]         0.711488    0.583664    0.009437    2.070064    0.489819    1.000    2      IlnBrlens{1}_rate[26]         0.697476    0.550379    0.004790    1.985042    0.487025    1.000    2      IlnBrlens{1}_rate[27]         0.683164    0.549765    0.004134    1.963765    0.476237    1.000    2      IlnBrlens{1}_rate[28]         1.359030    2.415623    0.007149    4.029374    0.920057    1.000    2      IlnBrlens{1}_rate[29]         1.021832    1.449750    0.008503    3.107861    0.669042    1.000    2      IlnBrlens{1}_rate[30]         0.449283    0.206807    0.007262    1.252626    0.320714    1.000    2      IlnBrlens{1}_rate[31]         0.504565    0.283930    0.007277    1.418728    0.356454    1.001    2      IlnBrlens{1}_rate[32]         0.829266    0.977212    0.001171    2.476572    0.546188    1.001    2      IlnBrlens{1}_rate[33]         0.667057    0.521486    0.004887    1.932144    0.463475    1.000    2      IlnBrlens{1}_rate[34]         1.134498    1.380284    0.013124    3.174391    0.809080    1.000    2      IlnBrlens{1}_rate[35]         0.805620    0.897338    0.007081    2.342021    0.543847    1.000    2      IlnBrlens{1}_rate[36]         0.780743    0.817081    0.004839    2.350280    0.522796    1.000    2      IlnBrlens{1}_rate[37]         1.224869    1.783438    0.007135    3.562717    0.838471    1.000    2      IlnBrlens{1}_rate[38]         0.702979    0.579344    0.008713    2.031376    0.488891    1.000    2      IlnBrlens{1}_rate[39]         1.236310    1.130698    0.044798    3.136425    0.964577    1.000    2      IlnBrlens{1}_rate[40]         0.955399    0.042414    0.322247    1.000061    1.000000    1.000    2      IlnBrlens{1}_rate[41]         0.794124    0.196901    0.020420    1.182868    1.000000    1.000    2      IlnBrlens{1}_rate[42]         1.574361    2.394525    0.074081    4.252449    1.000000    1.000    2      IlnBrlens{1}_rate[43]         1.043565    1.740085    0.020501    2.719252    0.752101    1.000    2      IlnBrlens{1}_rate[44]         1.423786    1.785174    0.063836    3.559619    1.000000    1.000    2      IlnBrlens{1}_rate[45]         0.565172    0.330871    0.005161    1.522600    0.415862    1.000    2      IlnBrlens{1}_rate[46]         5.537906   43.610507    0.302038   16.216923    3.590016    1.000    2      IlnBrlens{1}_rate[47]         0.707057    0.276844    0.014769    1.690360    0.577125    1.000    2      IlnBrlens{1}_rate[48]         0.414559    0.119972    0.015184    1.092934    0.323589    1.000    2      IlnBrlens{1}_rate[49]         1.988662    9.601465    0.007150    6.697109    1.038321    1.000    2      IlnBrlens{1}_rate[50]         0.384288    0.094292    0.010958    0.970133    0.300824    1.000    2      IlnBrlens{1}_rate[51]         0.958696    0.632488    0.043237    2.427949    0.761452    1.000    2      IlnBrlens{1}_rate[52]         1.321629    1.053609    0.091330    3.266960    1.065889    1.000    2      IlnBrlens{1}_rate[53]         0.761811    0.427738    0.019899    1.961202    0.591705    1.000    2      IlnBrlens{1}_rate[54]         1.541336    1.752101    0.059489    3.787235    1.214249    1.000    2      IlnBrlens{1}_rate[55]         0.509699    0.231944    0.007234    1.360286    0.372881    1.000    2      IlnBrlens{1}_rate[56]         1.695217    2.311657    0.032747    4.462697    1.282666    1.000    2      IlnBrlens{1}_rate[57]         0.539379    0.280161    0.007805    1.491104    0.384762    1.000    2      IlnBrlens{1}_rate[58]         1.281924    1.730459    0.021142    3.445058    0.928961    1.000    2      IlnBrlens{1}_rate[59]         7.630884  140.095026    0.033414   25.433095    4.033889    1.000    2      IlnBrlens{1}_rate[60]         0.824138    0.921349    0.007486    2.385998    0.557824    1.000    2      IlnBrlens{1}_rate[61]         0.711888    0.501699    0.006759    1.986621    0.516878    1.000    2      IlnBrlens{1}_rate[62]         0.873140    0.654327    0.013410    2.320498    0.661036    1.000    2      IlnBrlens{1}_rate[63]         0.774140    0.695968    0.007951    2.248345    0.542674    1.000    2      IlnBrlens{1}_rate[64]         0.670848    0.478516    0.010806    1.899360    0.479431    1.000    2      IlnBrlens{1}_rate[65]         0.681426    0.468669    0.012851    1.897492    0.489739    1.001    2      IlnBrlens{1}_rate[66]         0.856830    0.789670    0.010800    2.374959    0.614979    1.000    2      IlnBrlens{1}_rate[67]         0.475711    0.223913    0.003474    1.324010    0.341760    1.000    2      IlnBrlens{1}_rate[68]         0.502604    0.227722    0.006760    1.370890    0.368954    1.000    2      IlnBrlens{1}_rate[69]         1.168153    1.803947    0.009522    3.502039    0.789357    1.000    2      IlnBrlens{1}_rate[70]         0.795447    0.730361    0.002376    2.293971    0.550664    1.000    2      IlnBrlens{1}_rate[71]         0.727938    0.566526    0.007730    2.065968    0.518403    1.000    2      IlnBrlens{1}_rate[72]         0.609795    0.409822    0.005515    1.727268    0.430640    1.000    2      IlnBrlens{1}_rate[73]         0.754754    0.632297    0.008025    2.184796    0.532036    1.000    2      IlnBrlens{1}_rate[74]         1.289316    1.900716    0.024048    3.641030    0.902288    1.000    2      IlnBrlens{1}_rate[75]         0.553993    0.352270    0.005985    1.587544    0.391785    1.000    2      IlnBrlens{1}_rate[76]         2.646398    8.607412    0.018250    7.247921    1.870348    1.000    2      IlnBrlens{1}_rate[77]         0.810618    0.857507    0.013997    2.399119    0.541774    1.000    2      IlnBrlens{1}_rate[78]         0.952074    1.021070    0.006693    2.704750    0.667559    1.000    2      IlnBrlens{1}_rate[79]         0.979304    1.261429    0.018102    2.801848    0.673289    1.000    2      IlnBrlens{1}_rate[80]         0.672463    0.439013    0.006428    1.913168    0.478873    1.000    2      IlnBrlens{1}_rate[81]         0.836688    0.807209    0.003792    2.433254    0.573665    1.000    2      IlnBrlens{1}_rate[82]         0.746347    0.697769    0.003528    2.122190    0.515496    1.001    2      IlnBrlens{1}_rate[83]         0.764868    0.725365    0.009558    2.187861    0.523346    1.000    2      IlnBrlens{1}_rate[84]         1.215173    2.528757    0.011583    3.766137    0.760371    1.001    2      IlnBrlens{1}_rate[85]         0.861187    0.837657    0.004568    2.408076    0.599394    1.000    2      IlnBrlens{1}_rate[86]         1.280756    1.919971    0.006472    3.575287    0.890867    1.000    2      IlnBrlens{1}_rate[87]         1.002418    1.429206    0.010560    2.956206    0.668077    1.000    2      IlnBrlens{1}_rate[88]         1.110049    2.201309    0.004127    3.662021    0.641727    1.001    2      IlnBrlens{1}_rate[89]         0.640633    0.398888    0.009847    1.807549    0.452834    1.000    2      IlnBrlens{1}_rate[90]         0.882514    1.019975    0.008256    2.644441    0.584710    1.000    2      IlnBrlens{1}_rate[91]         0.817148    0.839121    0.009561    2.319834    0.576294    1.000    2      IlnBrlens{1}_rate[92]         0.656028    0.506350    0.005581    1.893315    0.457138    1.000    2      IlnBrlens{1}_rate[93]         1.110780    1.784857    0.004795    3.375283    0.707976    1.000    2      IlnBrlens{1}_rate[94]         1.059035    2.426903    0.006038    3.291448    0.631747    1.000    2      IlnBrlens{1}_rate[95]         1.096504    1.688313    0.003453    3.309707    0.708663    1.000    2      IlnBrlens{1}_rate[96]         0.704870    0.591787    0.003028    1.974453    0.485949    1.000    2      IlnBrlens{1}_rate[97]         1.285897    2.052057    0.023322    3.682544    0.874232    1.001    2      IlnBrlens{1}_rate[98]         0.708185    0.685797    0.009453    2.066859    0.472871    1.000    2      IlnBrlens{1}_rate[99]         2.477194    7.778760    0.017892    7.239002    1.672199    1.001    2      IlnBrlens{1}_rate[100]        1.248532    5.216887    0.009717    3.836819    0.720128    1.000    2      IlnBrlens{1}_rate[101]        0.779199    0.837661    0.008863    2.281778    0.530207    1.000    2      IlnBrlens{1}_rate[102]        0.994652    1.398853    0.010050    2.888099    0.659581    1.000    2      IlnBrlens{1}_rate[103]        0.772942    0.786197    0.010370    2.271945    0.516208    1.000    2      IlnBrlens{1}_rate[104]        0.785427    0.782983    0.010739    2.290858    0.539693    1.000    2      IlnBrlens{1}_rate[105]        0.727207    0.625848    0.004180    2.157571    0.489198    1.000    2      IlnBrlens{1}_rate[106]        0.994052    1.528134    0.006928    2.924750    0.653079    1.001    2      IlnBrlens{1}_rate[107]        1.265489    2.160051    0.008170    3.678079    0.850595    1.000    2      IlnBrlens{1}_rate[108]        0.954651    1.311363    0.008399    2.778315    0.634814    1.000    2      IlnBrlens{1}_rate[109]        0.878996    0.963664    0.016391    2.482348    0.610192    1.001    2      IlnBrlens{1}_rate[110]        0.666915    0.603852    0.008475    1.917693    0.452885    1.000    2      IlnBrlens{1}_rate[111]        1.703872    4.587524    0.004560    5.245065    1.008228    1.001    2      IlnBrlens{1}_rate[112]        0.695645    0.556026    0.015934    1.985769    0.480343    1.001    2      IlnBrlens{1}_rate[113]        0.980678    1.470039    0.012447    2.869086    0.647526    1.000    2      IlnBrlens{1}_rate[114]        1.286309    2.283136    0.018308    3.956757    0.841542    1.000    2      IlnBrlens{1}_rate[115]        3.225790   44.731104    0.007629   11.895512    1.424196    1.001    2      IlnBrlens{1}_rate[116]        1.011173    1.215514    0.024844    2.884803    0.688992    1.000    2      IlnBrlens{1}_rate[117]        0.811120    0.753187    0.009506    2.261708    0.573473    1.002    2      IlnBrlens{1}_rate[118]        0.535121    0.262364    0.010821    1.475373    0.392819    1.000    2      IlnBrlens{1}_rate[119]        1.862659    3.642566    0.027441    5.526638    1.269801    1.001    2      IlnBrlens{1}_rate[120]        0.748585    0.653687    0.007255    2.171247    0.510699    1.000    2      IlnBrlens{1}_rate[121]        1.038422    1.409649    0.012932    3.154052    0.690105    1.000    2      IlnBrlens{1}_rate[122]        0.669722    0.502759    0.011778    1.910918    0.482323    1.000    2      IlnBrlens{1}_rate[123]        1.285284    3.103799    0.014642    4.312248    0.728025    1.002    2      IlnBrlens{2}_length[1]        0.226535    0.070739    0.000023    0.636245    0.157888    1.000    2      IlnBrlens{2}_length[2]        0.019894    0.004050    0.000000    0.119709    0.000000    1.000    2      IlnBrlens{2}_length[3]        0.351301    0.126928    0.015441    0.908239    0.259565    1.000    2      IlnBrlens{2}_length[4]        0.154175    0.033072    0.000000    0.427732    0.111262    1.000    2      IlnBrlens{2}_length[5]        0.028410    0.006058    0.000000    0.163182    0.000000    1.001    2      IlnBrlens{2}_length[6]        0.182445    0.051786    0.000000    0.572919    0.119633    1.000    2      IlnBrlens{2}_length[7]        0.126029    0.012680    0.000000    0.329946    0.097103    1.000    2      IlnBrlens{2}_length[8]        0.001606    0.000057    0.000000    0.010121    0.000000    1.001    2      IlnBrlens{2}_length[9]        0.017206    0.000287    0.000000    0.045298    0.012622    1.000    2      IlnBrlens{2}_length[10]       0.030532    0.015337    0.000000    0.203347    0.000000    1.000    2      IlnBrlens{2}_length[11]       0.004325    0.000418    0.000000    0.026390    0.000000    1.000    2      IlnBrlens{2}_length[12]       0.049565    0.002039    0.000818    0.129202    0.037810    1.000    2      IlnBrlens{2}_length[13]       0.043689    0.001489    0.000726    0.108343    0.034248    1.001    2      IlnBrlens{2}_length[14]       0.063024    0.003560    0.001612    0.165895    0.047464    1.000    2      IlnBrlens{2}_length[15]       0.106371    0.008164    0.003580    0.261203    0.085148    1.000    2      IlnBrlens{2}_length[16]       0.016759    0.000450    0.000015    0.053534    0.010231    1.000    2      IlnBrlens{2}_length[17]       0.016927    0.000461    0.000011    0.054093    0.010307    1.000    2      IlnBrlens{2}_length[18]       0.094595    0.005814    0.003074    0.229891    0.076970    1.000    2      IlnBrlens{2}_length[19]       0.358234    0.195955    0.000000    1.011518    0.250429    1.000    2      IlnBrlens{2}_length[20]       0.264776    0.073173    0.000000    0.714539    0.196546    1.000    2      IlnBrlens{2}_length[21]       0.020980    0.000329    0.000349    0.052674    0.016769    1.001    2      IlnBrlens{2}_length[22]       0.024281    0.000534    0.000238    0.065358    0.018217    1.001    2      IlnBrlens{2}_length[23]       0.008029    0.000086    0.000014    0.023707    0.005360    1.001    2      IlnBrlens{2}_length[24]       0.148821    0.012179    0.002625    0.347801    0.124266    1.002    2      IlnBrlens{2}_length[25]       0.005410    0.000052    0.000005    0.017817    0.003168    1.000    2      IlnBrlens{2}_length[26]       0.006451    0.000075    0.000003    0.020692    0.003875    1.000    2      IlnBrlens{2}_length[27]       0.006411    0.000070    0.000005    0.020787    0.003821    1.000    2      IlnBrlens{2}_length[28]       0.016602    0.000348    0.000063    0.049907    0.011057    1.000    2      IlnBrlens{2}_length[29]       0.016821    0.000374    0.000081    0.050878    0.011024    1.000    2      IlnBrlens{2}_length[30]       0.039114    0.001139    0.000596    0.101284    0.030462    1.000    2      IlnBrlens{2}_length[31]       0.026881    0.000657    0.000181    0.073644    0.019832    1.000    2      IlnBrlens{2}_length[32]       0.003936    0.000044    0.000000    0.014507    0.001797    1.000    2      IlnBrlens{2}_length[33]       0.017976    0.000375    0.000058    0.052322    0.012283    1.000    2      IlnBrlens{2}_length[34]       0.013526    0.000242    0.000038    0.041301    0.008822    1.000    2      IlnBrlens{2}_length[35]       0.004119    0.000051    0.000000    0.015451    0.001816    1.000    2      IlnBrlens{2}_length[36]       0.008929    0.000148    0.000026    0.027989    0.005413    1.001    2      IlnBrlens{2}_length[37]       0.008973    0.000170    0.000006    0.028183    0.005385    1.001    2      IlnBrlens{2}_length[38]       0.008221    0.000091    0.000019    0.024449    0.005467    1.000    2      IlnBrlens{2}_length[39]       0.132821    0.013170    0.000000    0.346676    0.103643    1.000    2      IlnBrlens{2}_length[40]       0.000871    0.000022    0.000000    0.004590    0.000000    1.000    2      IlnBrlens{2}_length[41]       0.006694    0.000131    0.000000    0.027284    0.000000    1.000    2      IlnBrlens{2}_length[42]       0.067899    0.006903    0.000000    0.218153    0.046086    1.000    2      IlnBrlens{2}_length[43]       0.120953    0.011014    0.000000    0.316284    0.094358    1.000    2      IlnBrlens{2}_length[44]       0.052479    0.007060    0.000000    0.217393    0.014013    1.001    2      IlnBrlens{2}_length[45]       0.329010    0.099370    0.000000    0.824955    0.250918    1.000    2      IlnBrlens{2}_length[46]       0.657662    0.244082    0.039135    1.518277    0.553112    1.000    2      IlnBrlens{2}_length[47]       0.118321    0.007862    0.001894    0.281230    0.099202    1.001    2      IlnBrlens{2}_length[48]       0.137364    0.017943    0.001746    0.360140    0.105008    1.000    2      IlnBrlens{2}_length[49]       0.134534    0.030803    0.000002    0.431044    0.082377    1.000    2      IlnBrlens{2}_length[50]       0.257778    0.042719    0.009920    0.613003    0.212117    1.000    2      IlnBrlens{2}_length[51]       0.101862    0.014746    0.001312    0.272411    0.072438    1.000    2      IlnBrlens{2}_length[52]       0.442723    0.125598    0.017388    1.032473    0.371229    1.000    2      IlnBrlens{2}_length[53]       0.080973    0.003964    0.001317    0.198988    0.066631    1.001    2      IlnBrlens{2}_length[54]       0.195522    0.025995    0.001833    0.486348    0.156398    1.001    2      IlnBrlens{2}_length[55]       0.016035    0.000236    0.000098    0.042568    0.012149    1.001    2      IlnBrlens{2}_length[56]       0.131017    0.017895    0.001360    0.358013    0.094894    1.000    2      IlnBrlens{2}_length[57]       0.016277    0.000333    0.000092    0.048336    0.010991    1.001    2      IlnBrlens{2}_length[58]       0.018334    0.000407    0.000000    0.054406    0.012382    1.000    2      IlnBrlens{2}_length[59]       0.493849    0.257869    0.004951    1.310876    0.365113    1.001    2      IlnBrlens{2}_length[60]       0.168270    0.030167    0.000647    0.470972    0.120684    1.000    2      IlnBrlens{2}_length[61]       0.313692    0.114513    0.000001    0.876329    0.222772    1.000    2      IlnBrlens{2}_length[62]       0.104487    0.017002    0.000000    0.317555    0.067822    1.000    2      IlnBrlens{2}_length[63]       0.010551    0.000190    0.000000    0.033770    0.006314    1.000    2      IlnBrlens{2}_length[64]       0.083776    0.012089    0.000001    0.265762    0.051020    1.000    2      IlnBrlens{2}_length[65]       0.019338    0.000442    0.000004    0.057708    0.013068    1.000    2      IlnBrlens{2}_length[66]       0.024041    0.000666    0.000000    0.071671    0.016341    1.000    2      IlnBrlens{2}_length[67]       0.028901    0.000718    0.000002    0.077972    0.021915    1.001    2      IlnBrlens{2}_length[68]       0.027059    0.000677    0.000002    0.075108    0.019949    1.000    2      IlnBrlens{2}_length[69]       0.209671    0.071515    0.000006    0.595450    0.141993    1.000    2      IlnBrlens{2}_length[70]       0.201200    0.071261    0.000001    0.618485    0.129074    1.000    2      IlnBrlens{2}_length[71]       0.049337    0.002505    0.000005    0.143122    0.034524    1.001    2      IlnBrlens{2}_length[72]       0.016599    0.000356    0.000000    0.051185    0.010733    1.001    2      IlnBrlens{2}_length[73]       0.017718    0.000568    0.000005    0.053560    0.011258    1.000    2      IlnBrlens{2}_length[74]       0.216842    0.068541    0.000009    0.633049    0.146034    1.000    2      IlnBrlens{2}_length[75]       0.017597    0.000416    0.000000    0.054035    0.011397    1.001    2      IlnBrlens{2}_length[76]       0.117599    0.016870    0.000009    0.329773    0.083811    1.001    2      IlnBrlens{2}_length[77]       0.087346    0.010202    0.000003    0.264498    0.057697    1.000    2      IlnBrlens{2}_length[78]       0.217433    0.089198    0.000006    0.663884    0.137830    1.000    2      IlnBrlens{2}_length[79]       0.010711    0.000207    0.000001    0.035258    0.006249    1.000    2      IlnBrlens{2}_length[80]       0.014544    0.000198    0.000000    0.038594    0.011186    1.000    2      IlnBrlens{2}_length[81]       0.008837    0.000101    0.000000    0.027263    0.005728    1.000    2      IlnBrlens{2}_length[82]       0.004760    0.000051    0.000000    0.016700    0.002449    1.000    2      IlnBrlens{2}_length[83]       0.004781    0.000052    0.000000    0.017005    0.002432    1.000    2      IlnBrlens{2}_length[84]       0.072414    0.006117    0.000001    0.213789    0.049466    1.001    2      IlnBrlens{2}_length[85]       0.262999    0.112212    0.000004    0.781797    0.175047    1.000    2      IlnBrlens{2}_length[86]       0.183595    0.042533    0.000017    0.537772    0.124169    1.001    2      IlnBrlens{2}_length[87]       0.212606    0.077596    0.000002    0.684746    0.131226    1.001    2      IlnBrlens{2}_length[88]       0.060665    0.004822    0.000001    0.186286    0.040185    1.000    2      IlnBrlens{2}_length[89]       0.022281    0.000709    0.000007    0.065223    0.015132    1.000    2      IlnBrlens{2}_length[90]       0.044252    0.003268    0.000003    0.144004    0.026947    1.000    2      IlnBrlens{2}_length[91]       0.136688    0.043290    0.000001    0.403270    0.087189    1.000    2      IlnBrlens{2}_length[92]       0.009485    0.000162    0.000000    0.032900    0.005236    1.001    2      IlnBrlens{2}_length[93]       0.092668    0.018612    0.000005    0.324412    0.048926    1.000    2      IlnBrlens{2}_length[94]       0.161091    0.043586    0.000002    0.494762    0.101788    1.000    2      IlnBrlens{2}_length[95]       0.049164    0.002838    0.000002    0.146345    0.033484    1.000    2      IlnBrlens{2}_length[96]       0.006216    0.000061    0.000000    0.020639    0.003648    1.000    2      IlnBrlens{2}_length[97]       0.182976    0.050998    0.000013    0.551769    0.118320    1.001    2      IlnBrlens{2}_length[98]       0.007682    0.000130    0.000001    0.026989    0.004020    1.000    2      IlnBrlens{2}_length[99]       0.109727    0.014395    0.000022    0.318512    0.076133    1.000    2      IlnBrlens{2}_length[100]      0.166436    0.048142    0.000014    0.515076    0.105191    1.000    2      IlnBrlens{2}_length[101]      0.003607    0.000034    0.000000    0.013018    0.001695    1.000    2      IlnBrlens{2}_length[102]      0.076391    0.007139    0.000003    0.232946    0.050814    1.001    2      IlnBrlens{2}_length[103]      0.057104    0.005265    0.000001    0.192066    0.033635    1.000    2      IlnBrlens{2}_length[104]      0.012225    0.000272    0.000001    0.041814    0.006905    1.002    2      IlnBrlens{2}_length[105]      0.014098    0.000192    0.000001    0.038058    0.010654    1.000    2      IlnBrlens{2}_length[106]      0.262418    0.131439    0.000003    0.881446    0.149697    1.000    2      IlnBrlens{2}_length[107]      0.127669    0.027414    0.000002    0.416538    0.076382    1.000    2      IlnBrlens{2}_length[108]      0.078365    0.017466    0.000008    0.240972    0.046694    1.000    2      IlnBrlens{2}_length[109]      0.259664    0.165193    0.000020    0.899389    0.137818    1.000    2      IlnBrlens{2}_length[110]      0.010041    0.000163    0.000004    0.033679    0.005749    1.000    2      IlnBrlens{2}_length[111]      0.055845    0.005634    0.000007    0.193224    0.031368    1.000    2      IlnBrlens{2}_length[112]      0.042932    0.002356    0.000004    0.132310    0.027913    1.002    2      IlnBrlens{2}_length[113]      0.006893    0.000103    0.000000    0.024679    0.003671    1.000    2      IlnBrlens{2}_length[114]      0.120759    0.023386    0.000020    0.388517    0.073993    1.001    2      IlnBrlens{2}_length[115]      0.174144    0.045487    0.000034    0.520560    0.114219    1.000    2      IlnBrlens{2}_length[116]      0.034608    0.001344    0.000002    0.107599    0.023134    1.003    2      IlnBrlens{2}_length[117]      0.017913    0.000436    0.000007    0.055620    0.011402    1.000    2      IlnBrlens{2}_length[118]      0.016411    0.000362    0.000003    0.048530    0.010936    1.000    2      IlnBrlens{2}_length[119]      0.165670    0.030800    0.000077    0.447692    0.120558    1.001    2      IlnBrlens{2}_length[120]      0.064384    0.006338    0.000004    0.202078    0.039821    1.000    2      IlnBrlens{2}_length[121]      0.114017    0.024610    0.000001    0.373402    0.066640    1.000    2      IlnBrlens{2}_length[122]      0.011958    0.000293    0.000000    0.039147    0.006861    1.005    2      IlnBrlens{2}_length[123]      0.046703    0.003979    0.000006    0.158092    0.025866    1.000    2      IlnBrlens{2}_rate[1]          0.980756    0.799265    0.017830    2.332908    0.838133    1.000    2      IlnBrlens{2}_rate[2]          0.990908    0.091895    0.254641    1.317673    1.000000    1.000    2      IlnBrlens{2}_rate[3]          1.506081    1.944694    0.100717    3.771675    1.133124    1.000    2      IlnBrlens{2}_rate[4]          1.048227    0.765645    0.044000    2.399907    0.910977    1.000    2      IlnBrlens{2}_rate[5]          0.980979    0.126488    0.180392    1.372314    1.000000    1.000    2      IlnBrlens{2}_rate[6]          1.130180    0.787375    0.059562    2.544029    1.000000    1.000    2      IlnBrlens{2}_rate[7]          0.906444    0.463437    0.037366    2.027263    0.800810    1.000    2      IlnBrlens{2}_rate[8]          0.985537    0.019881    0.708827    1.106988    1.000000    1.000    2      IlnBrlens{2}_rate[9]          1.305809    1.291699    0.050584    3.187171    1.028628    1.000    2      IlnBrlens{2}_rate[10]         0.996700    0.069119    0.437543    1.238162    1.000000    1.000    2      IlnBrlens{2}_rate[11]         0.981267    0.033888    0.530351    1.093189    1.000000    1.000    2      IlnBrlens{2}_rate[12]         1.364321    1.465523    0.061826    3.442504    1.051314    1.001    2      IlnBrlens{2}_rate[13]         1.037998    0.727903    0.031418    2.466303    0.884123    1.000    2      IlnBrlens{2}_rate[14]         1.305773    1.412170    0.070927    3.268597    1.015687    1.000    2      IlnBrlens{2}_rate[15]         0.925326    0.473151    0.027534    2.054734    0.819755    1.000    2      IlnBrlens{2}_rate[16]         0.860868    0.437315    0.025669    1.959567    0.767471    1.000    2      IlnBrlens{2}_rate[17]         0.862195    0.406551    0.021028    1.950822    0.770415    1.000    2      IlnBrlens{2}_rate[18]         0.830238    0.357674    0.027875    1.864937    0.739896    1.000    2      IlnBrlens{2}_rate[19]         1.153474    1.446046    0.024789    2.831025    0.923801    1.000    2      IlnBrlens{2}_rate[20]         0.892404    0.501060    0.019721    1.990443    0.798500    1.000    2      IlnBrlens{2}_rate[21]         0.861733    0.420352    0.024356    1.917687    0.759775    1.000    2      IlnBrlens{2}_rate[22]         0.863018    0.437974    0.015822    1.946383    0.770108    1.000    2      IlnBrlens{2}_rate[23]         0.932024    0.632976    0.016473    2.142276    0.812001    1.000    2      IlnBrlens{2}_rate[24]         0.599741    0.159754    0.022699    1.284240    0.528509    1.002    2      IlnBrlens{2}_rate[25]         0.966973    0.658654    0.018964    2.271682    0.821271    1.000    2      IlnBrlens{2}_rate[26]         0.942226    0.672283    0.023416    2.150823    0.811118    1.000    2      IlnBrlens{2}_rate[27]         0.955740    0.685078    0.013899    2.241614    0.817251    1.000    2      IlnBrlens{2}_rate[28]         0.891765    0.493530    0.023157    2.061509    0.785323    1.000    2      IlnBrlens{2}_rate[29]         0.890994    0.479373    0.022980    2.050980    0.784520    1.000    2      IlnBrlens{2}_rate[30]         0.800795    0.318310    0.025478    1.768961    0.720440    1.001    2      IlnBrlens{2}_rate[31]         0.838366    0.390574    0.020494    1.862086    0.746173    1.000    2      IlnBrlens{2}_rate[32]         0.973328    0.716475    0.026560    2.290778    0.823729    1.000    2      IlnBrlens{2}_rate[33]         0.900910    0.507199    0.027723    2.053561    0.789647    1.000    2      IlnBrlens{2}_rate[34]         0.909891    0.563528    0.010098    2.113174    0.783029    1.000    2      IlnBrlens{2}_rate[35]         0.961630    0.713775    0.016932    2.266886    0.817462    1.000    2      IlnBrlens{2}_rate[36]         0.930948    0.563630    0.025010    2.144245    0.809802    1.000    2      IlnBrlens{2}_rate[37]         0.937468    0.670905    0.024442    2.168460    0.813732    1.000    2      IlnBrlens{2}_rate[38]         0.953311    0.662238    0.030570    2.223215    0.821094    1.000    2      IlnBrlens{2}_rate[39]         0.752247    0.243742    0.021740    1.605301    0.686798    1.001    2      IlnBrlens{2}_rate[40]         0.981589    0.025935    0.661605    1.107846    1.000000    1.000    2      IlnBrlens{2}_rate[41]         0.920316    0.159864    0.065726    1.409063    1.000000    1.000    2      IlnBrlens{2}_rate[42]         0.953678    0.295232    0.045420    1.803833    1.000000    1.000    2      IlnBrlens{2}_rate[43]         0.704088    0.221215    0.028005    1.476762    0.646388    1.000    2      IlnBrlens{2}_rate[44]         0.844960    0.135520    0.037000    1.270110    1.000000    1.000    2      IlnBrlens{2}_rate[45]         1.400054    1.435851    0.112428    3.362673    1.085840    1.001    2      IlnBrlens{2}_rate[46]         0.885941    0.359595    0.065608    1.881587    0.792903    1.000    2      IlnBrlens{2}_rate[47]         0.631277    0.182849    0.011256    1.362757    0.566360    1.001    2      IlnBrlens{2}_rate[48]         0.995555    0.689003    0.035125    2.386004    0.858044    1.000    2      IlnBrlens{2}_rate[49]         0.997580    0.766592    0.024101    2.383866    0.840718    1.000    2      IlnBrlens{2}_rate[50]         1.096957    0.692229    0.050344    2.555424    0.935571    1.000    2      IlnBrlens{2}_rate[51]         1.331698    1.629133    0.044972    3.263947    1.027518    1.000    2      IlnBrlens{2}_rate[52]         0.855272    0.401245    0.048733    1.832860    0.758294    1.000    2      IlnBrlens{2}_rate[53]         0.696994    0.218453    0.017515    1.478331    0.635413    1.001    2      IlnBrlens{2}_rate[54]         0.686374    0.208413    0.029456    1.449945    0.622463    1.002    2      IlnBrlens{2}_rate[55]         0.887534    0.484359    0.012722    1.997316    0.782373    1.000    2      IlnBrlens{2}_rate[56]         1.034590    0.657630    0.035532    2.408092    0.898428    1.000    2      IlnBrlens{2}_rate[57]         0.893540    0.495476    0.025951    2.026119    0.785134    1.000    2      IlnBrlens{2}_rate[58]         0.884976    0.457988    0.028891    2.035570    0.779263    1.000    2      IlnBrlens{2}_rate[59]         1.312040    1.610161    0.033762    3.292873    1.013041    1.001    2      IlnBrlens{2}_rate[60]         0.893887    0.492550    0.022193    2.015261    0.786244    1.000    2      IlnBrlens{2}_rate[61]         1.804232    4.177858    0.036433    5.132055    1.203095    1.000    2      IlnBrlens{2}_rate[62]         1.327108    1.873407    0.009800    3.478803    1.004358    1.000    2      IlnBrlens{2}_rate[63]         0.943462    0.654522    0.022593    2.182633    0.805351    1.000    2      IlnBrlens{2}_rate[64]         1.058632    1.133388    0.029276    2.484270    0.877042    1.000    2      IlnBrlens{2}_rate[65]         0.887764    0.480079    0.026834    2.032162    0.780224    1.000    2      IlnBrlens{2}_rate[66]         0.851916    0.423049    0.021138    1.885248    0.751755    1.000    2      IlnBrlens{2}_rate[67]         0.834991    0.384982    0.028826    1.839193    0.749392    1.000    2      IlnBrlens{2}_rate[68]         0.839561    0.414762    0.022434    1.863205    0.747500    1.000    2      IlnBrlens{2}_rate[69]         1.082143    1.105141    0.016217    2.725137    0.876476    1.000    2      IlnBrlens{2}_rate[70]         1.561114    3.803380    0.026541    4.296566    1.076059    1.000    2      IlnBrlens{2}_rate[71]         0.788994    0.319516    0.023223    1.797922    0.692920    1.000    2      IlnBrlens{2}_rate[72]         0.885957    0.465079    0.026312    2.041917    0.777423    1.000    2      IlnBrlens{2}_rate[73]         0.996582    0.937015    0.033959    2.396635    0.830232    1.000    2      IlnBrlens{2}_rate[74]         1.083147    1.156590    0.030332    2.675748    0.872407    1.000    2      IlnBrlens{2}_rate[75]         0.897697    0.500759    0.020433    2.068907    0.792442    1.000    2      IlnBrlens{2}_rate[76]         0.866260    0.466357    0.022797    1.946380    0.759628    1.000    2      IlnBrlens{2}_rate[77]         0.893704    0.462500    0.018008    2.063062    0.776002    1.000    2      IlnBrlens{2}_rate[78]         1.472121    2.408698    0.068071    3.804540    1.080774    1.001    2      IlnBrlens{2}_rate[79]         0.928728    0.630091    0.022134    2.098189    0.809549    1.000    2      IlnBrlens{2}_rate[80]         0.915617    0.516647    0.031473    2.125443    0.781747    1.000    2      IlnBrlens{2}_rate[81]         0.935340    0.599934    0.022414    2.190741    0.817604    1.000    2      IlnBrlens{2}_rate[82]         0.952532    0.663238    0.024879    2.285678    0.806855    1.000    2      IlnBrlens{2}_rate[83]         0.958247    0.642933    0.027741    2.270819    0.818842    1.000    2      IlnBrlens{2}_rate[84]         0.866169    0.406691    0.012906    1.921451    0.776795    1.001    2      IlnBrlens{2}_rate[85]         1.120865    1.250296    0.028681    2.729371    0.906396    1.000    2      IlnBrlens{2}_rate[86]         0.969797    0.627691    0.018920    2.146193    0.846236    1.000    2      IlnBrlens{2}_rate[87]         1.389255    1.874652    0.026090    3.752553    1.032906    1.001    2      IlnBrlens{2}_rate[88]         0.959179    0.541060    0.019566    2.194223    0.830130    1.000    2      IlnBrlens{2}_rate[89]         1.132066    1.058142    0.024977    2.714540    0.932681    1.000    2      IlnBrlens{2}_rate[90]         1.163197    1.169706    0.024590    2.863164    0.960564    1.000    2      IlnBrlens{2}_rate[91]         1.292743    2.051516    0.056705    3.077609    1.005927    1.000    2      IlnBrlens{2}_rate[92]         0.956696    0.681305    0.025677    2.270904    0.823859    1.000    2      IlnBrlens{2}_rate[93]         0.982294    0.745497    0.026761    2.213734    0.835630    1.000    2      IlnBrlens{2}_rate[94]         0.984282    0.752234    0.028427    2.322829    0.829918    1.000    2      IlnBrlens{2}_rate[95]         1.037742    0.719429    0.022321    2.466130    0.891391    1.000    2      IlnBrlens{2}_rate[96]         0.969293    0.667592    0.029966    2.344373    0.836481    1.000    2      IlnBrlens{2}_rate[97]         1.025468    0.769563    0.017490    2.435209    0.863544    1.000    2      IlnBrlens{2}_rate[98]         0.947295    0.698241    0.025412    2.193469    0.812224    1.000    2      IlnBrlens{2}_rate[99]         1.138173    0.972375    0.061252    2.845738    0.912506    1.000    2      IlnBrlens{2}_rate[100]        1.011603    1.195103    0.030513    2.330428    0.856417    1.000    2      IlnBrlens{2}_rate[101]        0.963710    0.685233    0.019978    2.273020    0.822892    1.000    2      IlnBrlens{2}_rate[102]        0.832603    0.379981    0.012139    1.908854    0.740473    1.000    2      IlnBrlens{2}_rate[103]        0.870107    0.527345    0.027840    2.009828    0.752890    1.000    2      IlnBrlens{2}_rate[104]        0.923134    0.622614    0.010239    2.113033    0.787115    1.001    2      IlnBrlens{2}_rate[105]        0.904897    0.465049    0.018284    2.107491    0.787890    1.000    2      IlnBrlens{2}_rate[106]        1.461523    2.842052    0.039470    3.970425    1.028731    1.000    2      IlnBrlens{2}_rate[107]        1.057398    0.939562    0.032569    2.579192    0.877431    1.000    2      IlnBrlens{2}_rate[108]        0.938837    0.716402    0.013541    2.303841    0.782287    1.000    2      IlnBrlens{2}_rate[109]        1.466210    2.837803    0.031967    4.112575    1.017733    1.000    2      IlnBrlens{2}_rate[110]        0.930292    0.591699    0.039214    2.170917    0.822339    1.002    2      IlnBrlens{2}_rate[111]        0.952472    0.558523    0.018986    2.244212    0.842834    1.000    2      IlnBrlens{2}_rate[112]        0.806617    0.345590    0.025497    1.816565    0.730057    1.001    2      IlnBrlens{2}_rate[113]        0.958028    0.824041    0.021327    2.231518    0.807179    1.000    2      IlnBrlens{2}_rate[114]        1.015638    0.796796    0.028505    2.408870    0.855276    1.000    2      IlnBrlens{2}_rate[115]        0.957988    0.642715    0.016907    2.146960    0.845773    1.000    2      IlnBrlens{2}_rate[116]        0.828722    0.360703    0.025497    1.794212    0.743160    1.003    2      IlnBrlens{2}_rate[117]        0.900874    0.545623    0.028322    2.064983    0.785494    1.000    2      IlnBrlens{2}_rate[118]        0.875047    0.438232    0.043650    1.962654    0.769880    1.005    2      IlnBrlens{2}_rate[119]        0.996723    0.705379    0.034170    2.280420    0.864744    1.000    2      IlnBrlens{2}_rate[120]        0.925454    0.784263    0.019382    2.155160    0.779449    1.000    2      IlnBrlens{2}_rate[121]        0.971358    0.698490    0.024339    2.314741    0.825949    1.000    2      IlnBrlens{2}_rate[122]        0.932324    0.626005    0.049880    2.131300    0.814625    1.002    2      IlnBrlens{2}_rate[123]        0.953416    0.564324    0.032311    2.229681    0.816824    1.000    2      IlnBrlens{3}_length[1]        0.160286    0.074686    0.000006    0.579688    0.075647    1.000    2      IlnBrlens{3}_length[2]        0.010064    0.001622    0.000000    0.055137    0.000000    1.000    2      IlnBrlens{3}_length[3]        0.104702    0.017986    0.000000    0.338461    0.062265    1.000    2      IlnBrlens{3}_length[4]        0.094133    0.026152    0.000000    0.339417    0.045665    1.000    2      IlnBrlens{3}_length[5]        0.013896    0.004295    0.000000    0.069379    0.000000    1.000    2      IlnBrlens{3}_length[6]        0.093619    0.034195    0.000000    0.357408    0.038724    1.000    2      IlnBrlens{3}_length[7]        0.095018    0.024231    0.000000    0.329979    0.048761    1.000    2      IlnBrlens{3}_length[8]        0.001106    0.000046    0.000000    0.005033    0.000000    1.000    2      IlnBrlens{3}_length[9]        0.011591    0.000442    0.000000    0.042158    0.005329    1.000    2      IlnBrlens{3}_length[10]       0.013783    0.005610    0.000000    0.066771    0.000000    1.000    2      IlnBrlens{3}_length[11]       0.001812    0.000142    0.000000    0.008017    0.000000    1.000    2      IlnBrlens{3}_length[12]       0.022013    0.000967    0.000076    0.074654    0.012142    1.000    2      IlnBrlens{3}_length[13]       0.024536    0.001356    0.000066    0.082476    0.013405    1.000    2      IlnBrlens{3}_length[14]       0.026998    0.001500    0.000031    0.090509    0.014992    1.000    2      IlnBrlens{3}_length[15]       0.052878    0.004588    0.000072    0.173785    0.030999    1.001    2      IlnBrlens{3}_length[16]       0.013210    0.000556    0.000003    0.050667    0.005549    1.000    2      IlnBrlens{3}_length[17]       0.013088    0.000585    0.000003    0.049162    0.005443    1.000    2      IlnBrlens{3}_length[18]       0.052964    0.004905    0.000189    0.171786    0.031408    1.000    2      IlnBrlens{3}_length[19]       0.259795    0.122281    0.000000    0.869984    0.148108    1.000    2      IlnBrlens{3}_length[20]       0.170765    0.130486    0.000000    0.574104    0.083375    1.000    2      IlnBrlens{3}_length[21]       0.005908    0.000038    0.000041    0.017607    0.003988    1.001    2      IlnBrlens{3}_length[22]       0.014110    0.000140    0.000133    0.036831    0.011019    1.000    2      IlnBrlens{3}_length[23]       0.002943    0.000013    0.000002    0.009480    0.001796    1.000    2      IlnBrlens{3}_length[24]       0.023261    0.000787    0.000444    0.059410    0.017219    1.001    2      IlnBrlens{3}_length[25]       0.002033    0.000007    0.000001    0.007003    0.001131    1.000    2      IlnBrlens{3}_length[26]       0.004560    0.000022    0.000013    0.013563    0.003105    1.000    2      IlnBrlens{3}_length[27]       0.004608    0.000023    0.000010    0.013738    0.003106    1.000    2      IlnBrlens{3}_length[28]       0.005558    0.000032    0.000020    0.016534    0.003803    1.000    2      IlnBrlens{3}_length[29]       0.005570    0.000033    0.000021    0.016298    0.003822    1.001    2      IlnBrlens{3}_length[30]       0.059217    0.002618    0.000301    0.147052    0.048000    1.000    2      IlnBrlens{3}_length[31]       0.014459    0.000148    0.000166    0.037688    0.011243    1.000    2      IlnBrlens{3}_length[32]       0.001559    0.000006    0.000000    0.005909    0.000708    1.000    2      IlnBrlens{3}_length[33]       0.016840    0.000146    0.000974    0.040335    0.013841    1.001    2      IlnBrlens{3}_length[34]       0.003921    0.000018    0.000014    0.011950    0.002579    1.001    2      IlnBrlens{3}_length[35]       0.001580    0.000006    0.000000    0.005945    0.000727    1.000    2      IlnBrlens{3}_length[36]       0.005382    0.000030    0.000015    0.016057    0.003703    1.000    2      IlnBrlens{3}_length[37]       0.003127    0.000015    0.000007    0.010364    0.001843    1.000    2      IlnBrlens{3}_length[38]       0.005270    0.000027    0.000014    0.015181    0.003733    1.000    2      IlnBrlens{3}_length[39]       0.153976    0.022957    0.000000    0.419048    0.114411    1.000    2      IlnBrlens{3}_length[40]       0.000217    0.000001    0.000000    0.001059    0.000000    1.000    2      IlnBrlens{3}_length[41]       0.001885    0.000013    0.000000    0.008726    0.000000    1.000    2      IlnBrlens{3}_length[42]       0.052737    0.012507    0.000000    0.220760    0.017416    1.000    2      IlnBrlens{3}_length[43]       0.131480    0.033348    0.000000    0.436874    0.074423    1.000    2      IlnBrlens{3}_length[44]       0.039951    0.010282    0.000000    0.185964    0.004793    1.000    2      IlnBrlens{3}_length[45]       0.608521    0.906403    0.000000    1.954569    0.344505    1.000    2      IlnBrlens{3}_length[46]       0.428790    0.317739    0.000659    1.352514    0.260496    1.000    2      IlnBrlens{3}_length[47]       0.419273    0.153661    0.001921    1.137865    0.310219    1.000    2      IlnBrlens{3}_length[48]       0.065620    0.006909    0.000161    0.212901    0.039576    1.000    2      IlnBrlens{3}_length[49]       0.103745    0.043429    0.000000    0.405052    0.040744    1.000    2      IlnBrlens{3}_length[50]       0.085711    0.010238    0.000471    0.269587    0.053152    1.000    2      IlnBrlens{3}_length[51]       0.064589    0.007477    0.000157    0.216479    0.036675    1.000    2      IlnBrlens{3}_length[52]       0.256518    0.087195    0.000220    0.755430    0.175622    1.000    2      IlnBrlens{3}_length[53]       0.192259    0.052399    0.000024    0.643825    0.106652    1.000    2      IlnBrlens{3}_length[54]       0.023548    0.000844    0.000492    0.060691    0.017461    1.002    2      IlnBrlens{3}_length[55]       0.025214    0.000300    0.000173    0.057801    0.021225    1.000    2      IlnBrlens{3}_length[56]       0.221223    0.113918    0.000040    0.748630    0.119245    1.000    2      IlnBrlens{3}_length[57]       0.026829    0.000366    0.000004    0.062964    0.022561    1.000    2      IlnBrlens{3}_length[58]       0.007979    0.000070    0.000000    0.024299    0.005371    1.000    2      IlnBrlens{3}_length[59]       0.327565    0.277176    0.000033    1.142665    0.162250    1.000    2      IlnBrlens{3}_length[60]       2.565068    8.192347    0.000036    7.124911    1.784931    1.000    2      IlnBrlens{3}_length[61]       0.243477    0.144525    0.000004    0.832756    0.129046    1.000    2      IlnBrlens{3}_length[62]       0.040421    0.003416    0.000000    0.144299    0.020909    1.001    2      IlnBrlens{3}_length[63]       0.004827    0.000028    0.000000    0.014884    0.003178    1.000    2      IlnBrlens{3}_length[64]       0.038566    0.003705    0.000000    0.141183    0.018480    1.000    2      IlnBrlens{3}_length[65]       0.024944    0.000458    0.000002    0.065266    0.019636    1.001    2      IlnBrlens{3}_length[66]       0.034511    0.002033    0.000000    0.109715    0.021372    1.000    2      IlnBrlens{3}_length[67]       0.127334    0.014544    0.000000    0.352903    0.101559    1.000    2      IlnBrlens{3}_length[68]       0.108115    0.012626    0.000001    0.319058    0.079768    1.000    2      IlnBrlens{3}_length[69]       0.722323    0.835761    0.000003    2.267745    0.457406    1.000    2      IlnBrlens{3}_length[70]       0.158155    0.110139    0.000000    0.625297    0.057594    1.000    2      IlnBrlens{3}_length[71]       0.405113    0.128187    0.000001    1.043560    0.324450    1.000    2      IlnBrlens{3}_length[72]       0.013907    0.000171    0.000000    0.039123    0.010281    1.000    2      IlnBrlens{3}_length[73]       0.012732    0.000529    0.000002    0.046969    0.005626    1.000    2      IlnBrlens{3}_length[74]       0.165526    0.118044    0.000001    0.615080    0.071567    1.000    2      IlnBrlens{3}_length[75]       0.017935    0.000388    0.000000    0.056620    0.011761    1.000    2      IlnBrlens{3}_length[76]       0.104665    0.025056    0.000002    0.373621    0.052403    1.000    2      IlnBrlens{3}_length[77]       0.134321    0.031489    0.000000    0.452928    0.077919    1.000    2      IlnBrlens{3}_length[78]       0.111878    0.036188    0.000001    0.413776    0.049988    1.000    2      IlnBrlens{3}_length[79]       0.003553    0.000022    0.000000    0.012149    0.002014    1.000    2      IlnBrlens{3}_length[80]       0.004966    0.000036    0.000000    0.016106    0.003011    1.000    2      IlnBrlens{3}_length[81]       0.004770    0.000045    0.000000    0.016654    0.002567    1.000    2      IlnBrlens{3}_length[82]       0.002179    0.000010    0.000000    0.008028    0.001071    1.000    2      IlnBrlens{3}_length[83]       0.002038    0.000009    0.000000    0.007617    0.000983    1.000    2      IlnBrlens{3}_length[84]       0.066697    0.015609    0.000005    0.257528    0.027318    1.000    2      IlnBrlens{3}_length[85]       0.139382    0.068904    0.000001    0.498644    0.067205    1.000    2      IlnBrlens{3}_length[86]       0.174048    0.138444    0.000003    0.698822    0.062761    1.001    2      IlnBrlens{3}_length[87]       0.107280    0.044414    0.000003    0.397127    0.044901    1.000    2      IlnBrlens{3}_length[88]       0.057838    0.018334    0.000001    0.227819    0.021363    1.000    2      IlnBrlens{3}_length[89]       0.013772    0.000514    0.000004    0.051708    0.006396    1.000    2      IlnBrlens{3}_length[90]       0.010086    0.000266    0.000000    0.032412    0.005860    1.001    2      IlnBrlens{3}_length[91]       0.070948    0.016914    0.000001    0.262650    0.030520    1.000    2      IlnBrlens{3}_length[92]       0.005645    0.000078    0.000000    0.021508    0.002517    1.000    2      IlnBrlens{3}_length[93]       0.655245    3.073883    0.000000    3.350385    0.048081    1.000    2      IlnBrlens{3}_length[94]       0.156079    0.092259    0.000000    0.592995    0.060631    1.000    2      IlnBrlens{3}_length[95]       0.012121    0.000483    0.000000    0.036198    0.006928    1.001    2      IlnBrlens{3}_length[96]       0.004259    0.000031    0.000000    0.014746    0.002330    1.000    2      IlnBrlens{3}_length[97]       0.174179    0.132762    0.000003    0.655697    0.067593    1.001    2      IlnBrlens{3}_length[98]       0.003899    0.000024    0.000000    0.012998    0.002269    1.000    2      IlnBrlens{3}_length[99]       0.115752    0.040533    0.000004    0.430153    0.052624    1.000    2      IlnBrlens{3}_length[100]      0.134268    0.094893    0.000005    0.525742    0.048217    1.001    2      IlnBrlens{3}_length[101]      0.001525    0.000005    0.000000    0.005866    0.000693    1.000    2      IlnBrlens{3}_length[102]      0.069568    0.014754    0.000001    0.271861    0.029991    1.000    2      IlnBrlens{3}_length[103]      0.055505    0.011952    0.000000    0.219778    0.020487    1.001    2      IlnBrlens{3}_length[104]      0.007689    0.000115    0.000000    0.027713    0.003853    1.001    2      IlnBrlens{3}_length[105]      0.004404    0.000026    0.000001    0.013992    0.002831    1.000    2      IlnBrlens{3}_length[106]      0.166245    0.096955    0.000001    0.641482    0.068556    1.000    2      IlnBrlens{3}_length[107]      0.179815    0.573721    0.000000    0.604690    0.033937    1.001    2      IlnBrlens{3}_length[108]      0.100020    0.050652    0.000001    0.418297    0.030079    1.000    2      IlnBrlens{3}_length[109]      0.117662    0.037359    0.000005    0.455195    0.050845    1.000    2      IlnBrlens{3}_length[110]      0.005303    0.000031    0.000000    0.016083    0.003571    1.000    2      IlnBrlens{3}_length[111]      0.013611    0.001222    0.000003    0.040180    0.007897    1.001    2      IlnBrlens{3}_length[112]      0.010916    0.000171    0.000001    0.034656    0.006800    1.000    2      IlnBrlens{3}_length[113]      0.002451    0.000012    0.000000    0.008832    0.001286    1.000    2      IlnBrlens{3}_length[114]      0.106125    0.060932    0.000001    0.413112    0.036131    1.002    2      IlnBrlens{3}_length[115]      0.124876    0.049867    0.000012    0.476496    0.054269    1.000    2      IlnBrlens{3}_length[116]      0.010393    0.000251    0.000002    0.032064    0.006403    1.000    2      IlnBrlens{3}_length[117]      0.026632    0.002883    0.000000    0.110027    0.008892    1.000    2      IlnBrlens{3}_length[118]      0.025604    0.000539    0.000006    0.067138    0.020307    1.001    2      IlnBrlens{3}_length[119]      0.626649    0.688498    0.000030    2.104905    0.371153    1.002    2      IlnBrlens{3}_length[120]      0.056872    0.015203    0.000004    0.211185    0.022019    1.000    2      IlnBrlens{3}_length[121]      0.097173    0.057879    0.000000    0.378368    0.033905    1.000    2      IlnBrlens{3}_length[122]      0.006618    0.000066    0.000000    0.022481    0.003831    1.000    2      IlnBrlens{3}_length[123]      0.064757    0.019925    0.000002    0.266162    0.019208    1.000    2      IlnBrlens{3}_rate[1]          0.736804    1.330040    0.002411    2.483404    0.388846    1.000    2      IlnBrlens{3}_rate[2]          0.877116    0.183694    0.053539    1.000000    1.000000    1.000    2      IlnBrlens{3}_rate[3]          0.457893    0.319302    0.001661    1.459329    0.286000    1.001    2      IlnBrlens{3}_rate[4]          0.697828    1.135840    0.001417    2.327542    0.393423    1.000    2      IlnBrlens{3}_rate[5]          0.825580    0.263946    0.040842    1.000000    1.000000    1.000    2      IlnBrlens{3}_rate[6]          0.676320    0.819724    0.004422    1.904829    0.430362    1.000    2      IlnBrlens{3}_rate[7]          0.698603    1.145452    0.002086    2.290179    0.384575    1.000    2      IlnBrlens{3}_rate[8]          0.961665    0.069944    0.264931    1.000000    1.000000    1.000    2      IlnBrlens{3}_rate[9]          0.870625    2.033552    0.001690    3.059072    0.438010    1.000    2      IlnBrlens{3}_rate[10]         0.914641    0.104527    0.114868    1.000000    1.000000    1.000    2      IlnBrlens{3}_rate[11]         0.926992    0.073864    0.154388    1.000000    1.000000    1.000    2      IlnBrlens{3}_rate[12]         0.612334    0.704410    0.002928    2.040046    0.346698    1.000    2      IlnBrlens{3}_rate[13]         0.582893    0.660339    0.002413    1.937698    0.330283    1.000    2      IlnBrlens{3}_rate[14]         0.565436    0.611737    0.001266    1.878561    0.327107    1.000    2      IlnBrlens{3}_rate[15]         0.470968    0.342641    0.001130    1.515417    0.285383    1.001    2      IlnBrlens{3}_rate[16]         0.722446    1.165805    0.002197    2.465582    0.381236    1.000    2      IlnBrlens{3}_rate[17]         0.720561    1.234171    0.002701    2.529266    0.376914    1.000    2      IlnBrlens{3}_rate[18]         0.471331    0.386116    0.002607    1.471530    0.284535    1.000    2      IlnBrlens{3}_rate[19]         0.957896    1.669800    0.004420    3.140949    0.555645    1.000    2      IlnBrlens{3}_rate[20]         0.668227    3.120410    0.003367    2.026252    0.329865    1.000    2      IlnBrlens{3}_rate[21]         0.258468    0.076502    0.002305    0.766990    0.174680    1.000    2      IlnBrlens{3}_rate[22]         0.619953    0.411780    0.005003    1.787960    0.431020    1.000    2      IlnBrlens{3}_rate[23]         0.410461    0.272107    0.004475    1.307901    0.249743    1.000    2      IlnBrlens{3}_rate[24]         0.095917    0.015281    0.001746    0.242540    0.070870    1.001    2      IlnBrlens{3}_rate[25]         0.464914    0.396877    0.002386    1.486099    0.269849    1.000    2      IlnBrlens{3}_rate[26]         0.961066    1.529594    0.003974    2.968929    0.608483    1.000    2      IlnBrlens{3}_rate[27]         0.979500    1.445570    0.007413    3.065294    0.612556    1.000    2      IlnBrlens{3}_rate[28]         0.386054    0.206994    0.004241    1.202155    0.240965    1.000    2      IlnBrlens{3}_rate[29]         0.388526    0.223049    0.002487    1.201177    0.243477    1.001    2      IlnBrlens{3}_rate[30]         1.654550    6.683928    0.004761    4.685916    1.050231    1.000    2      IlnBrlens{3}_rate[31]         0.716024    1.050350    0.006490    2.527556    0.395261    1.000    2      IlnBrlens{3}_rate[32]         0.562795    0.926908    0.001977    1.918248    0.299936    1.000    2      IlnBrlens{3}_rate[33]         1.159227    1.401970    0.017313    3.196179    0.815393    1.000    2      IlnBrlens{3}_rate[34]         0.367524    0.309828    0.002521    1.185018    0.208500    1.000    2      IlnBrlens{3}_rate[35]         0.546604    0.690098    0.001037    1.829640    0.301412    1.000    2      IlnBrlens{3}_rate[36]         0.784432    0.926349    0.004903    2.352869    0.498324    1.000    2      IlnBrlens{3}_rate[37]         0.421913    0.316726    0.001260    1.361166    0.251071    1.000    2      IlnBrlens{3}_rate[38]         0.782109    0.799310    0.009301    2.348561    0.522301    1.000    2      IlnBrlens{3}_rate[39]         1.156336    2.278601    0.003942    3.385178    0.755180    1.001    2      IlnBrlens{3}_rate[40]         0.927968    0.060330    0.159433    1.000000    1.000000    1.000    2      IlnBrlens{3}_rate[41]         0.637782    0.175466    0.039212    1.000000    1.000000    1.000    2      IlnBrlens{3}_rate[42]         0.808626    0.891972    0.000819    2.131984    0.693173    1.000    2      IlnBrlens{3}_rate[43]         0.807253    1.130928    0.004777    2.573317    0.481679    1.000    2      IlnBrlens{3}_rate[44]         0.746224    0.393004    0.003485    1.321463    1.000000    1.000    2      IlnBrlens{3}_rate[45]         2.576472   12.278000    0.011980    7.935437    1.584510    1.000    2      IlnBrlens{3}_rate[46]         0.579150    0.523846    0.004315    1.795863    0.361458    1.000    2      IlnBrlens{3}_rate[47]         2.246607    4.268900    0.007194    5.903411    1.700723    1.001    2      IlnBrlens{3}_rate[48]         0.504896    0.385197    0.002822    1.623428    0.308588    1.000    2      IlnBrlens{3}_rate[49]         0.807723    1.634038    0.001055    2.784864    0.413399    1.000    2      IlnBrlens{3}_rate[50]         0.368661    0.183231    0.003937    1.134876    0.230278    1.000    2      IlnBrlens{3}_rate[51]         0.898487    1.375438    0.003072    2.873055    0.530531    1.000    2      IlnBrlens{3}_rate[52]         0.498627    0.284140    0.000790    1.409013    0.345561    1.000    2      IlnBrlens{3}_rate[53]         1.599183    3.561444    0.001326    5.144492    0.965017    1.000    2      IlnBrlens{3}_rate[54]         0.095118    0.012304    0.002479    0.269712    0.065154    1.001    2      IlnBrlens{3}_rate[55]         1.702670    2.412673    0.034115    4.534081    1.255838    1.000    2      IlnBrlens{3}_rate[56]         1.833421    6.327886    0.001883    5.904486    1.083088    1.000    2      IlnBrlens{3}_rate[57]         2.188724    5.782441    0.009310    6.063966    1.574117    1.000    2      IlnBrlens{3}_rate[58]         0.497063    0.406527    0.001924    1.585573    0.306422    1.000    2      IlnBrlens{3}_rate[59]         0.859586    1.787956    0.004257    2.882344    0.454605    1.000    2      IlnBrlens{3}_rate[60]        16.142819  379.856031    0.004266   45.066291   10.883793    1.000    2      IlnBrlens{3}_rate[61]         1.298188    2.977693    0.005685    4.222813    0.759412    1.000    2      IlnBrlens{3}_rate[62]         0.572146    0.606790    0.002374    1.898750    0.327390    1.001    2      IlnBrlens{3}_rate[63]         0.618289    0.654210    0.001275    1.987284    0.372085    1.000    2      IlnBrlens{3}_rate[64]         0.586638    0.749836    0.003405    1.945091    0.323167    1.000    2      IlnBrlens{3}_rate[65]         1.641659    4.026729    0.007883    5.043580    1.064123    1.001    2      IlnBrlens{3}_rate[66]         1.409857    3.443734    0.005612    4.713660    0.829709    1.000    2      IlnBrlens{3}_rate[67]         4.422177   32.561568    0.005581   13.799495    2.903553    1.000    2      IlnBrlens{3}_rate[68]         3.867522   26.635693    0.008038   12.202279    2.349548    1.000    2      IlnBrlens{3}_rate[69]         4.105047   28.423853    0.003625   12.830036    2.583633    1.000    2      IlnBrlens{3}_rate[70]         1.156409    3.597068    0.002210    4.131282    0.563339    1.000    2      IlnBrlens{3}_rate[71]         7.855949   76.404441    0.006627   22.182684    5.459267    1.000    2      IlnBrlens{3}_rate[72]         1.087674    2.201615    0.004214    3.381403    0.657024    1.000    2      IlnBrlens{3}_rate[73]         0.754949    1.405885    0.000447    2.600896    0.396779    1.000    2      IlnBrlens{3}_rate[74]         0.880074    2.575998    0.002938    3.109150    0.426057    1.000    2      IlnBrlens{3}_rate[75]         1.296356    3.609463    0.002547    4.555957    0.654076    1.000    2      IlnBrlens{3}_rate[76]         0.792403    1.211098    0.002744    2.529670    0.459718    1.000    2      IlnBrlens{3}_rate[77]         1.565500    5.864593    0.004699    5.267339    0.893952    1.000    2      IlnBrlens{3}_rate[78]         0.795523    1.353281    0.001439    2.676884    0.424321    1.000    2      IlnBrlens{3}_rate[79]         0.419493    0.382616    0.002005    1.339187    0.241747    1.000    2      IlnBrlens{3}_rate[80]         0.331334    0.169592    0.003149    1.012429    0.209882    1.001    2      IlnBrlens{3}_rate[81]         0.609537    0.828274    0.002258    2.009619    0.340177    1.001    2      IlnBrlens{3}_rate[82]         0.597484    0.853522    0.001838    1.986975    0.326025    1.000    2      IlnBrlens{3}_rate[83]         0.571663    0.747821    0.001832    1.960491    0.307184    1.000    2      IlnBrlens{3}_rate[84]         0.817921    1.881164    0.002285    2.861115    0.417874    1.000    2      IlnBrlens{3}_rate[85]         0.672845    1.670138    0.001354    2.261323    0.354577    1.000    2      IlnBrlens{3}_rate[86]         0.943206    3.495589    0.003881    3.538170    0.414427    1.000    2      IlnBrlens{3}_rate[87]         0.758067    1.385303    0.001644    2.575776    0.386475    1.000    2      IlnBrlens{3}_rate[88]         0.909118    2.212878    0.004491    3.317248    0.444432    1.000    2      IlnBrlens{3}_rate[89]         0.739618    1.223819    0.004112    2.465346    0.396060    1.000    2      IlnBrlens{3}_rate[90]         0.363673    0.275222    0.002337    1.196688    0.206777    1.000    2      IlnBrlens{3}_rate[91]         0.717290    1.284309    0.003896    2.519530    0.370784    1.000    2      IlnBrlens{3}_rate[92]         0.699960    1.250434    0.002604    2.375203    0.378290    1.000    2      IlnBrlens{3}_rate[93]         4.650159  160.434753    0.001446   19.895164    1.032673    1.000    2      IlnBrlens{3}_rate[94]         1.040826    3.900503    0.001849    3.696831    0.479138    1.001    2      IlnBrlens{3}_rate[95]         0.305179    0.159291    0.003183    0.992423    0.181137    1.000    2      IlnBrlens{3}_rate[96]         0.819597    1.266237    0.004368    2.725906    0.473925    1.000    2      IlnBrlens{3}_rate[97]         0.991796    2.814722    0.004034    3.505103    0.478209    1.000    2      IlnBrlens{3}_rate[98]         0.718762    1.171929    0.002285    2.382348    0.395358    1.000    2      IlnBrlens{3}_rate[99]         1.166340    2.823862    0.004700    4.084632    0.633905    1.000    2      IlnBrlens{3}_rate[100]        0.898484    4.066706
[truncated: 232,432 more chars]
