# Supplementary material for: The rise of predation in Jurassic lampreys
Source: Nat Commun. 2023 Oct 31;14:6652. doi: 10.1038/s41467-023-42251-0 (PMC10618186; doi:10.1038/s41467-023-42251-0)
Supplement: Supplementary file 4 — Supplementary Code 1-8 [file 41467_2023_42251_MOESM4_ESM.zip › Supplementary Codes 1-8/Supplementary Code 3.rtf]

Supplementary Code 3: All-compatible tree file#NEXUS[ID: 6291437938]begin taxa;       dimensions ntax=45;       taxlabels              Euconodonta              Jamoytius              Euphanerops              Achanarella              Ciderius              Cornovichthys              Lasanius              Birkenia              Rhyncholepis              Myxinikela              Tethymyxine              Paramyxine_fernholmi              Eptatretus_burgeri              Eptatretus_stoutii              Myxine_glutinosa              Rubicundus_eos              Rubicundus_lopheliae              Neomyxine_biniplicata              Myxineidus              Gilpichthys              Lethenteron_camtschaticum              Petromyzon_marinus              Lampetra_fluviatilis              Geotria_australis              Ichthyomyzon_bdellium              Ichthyomyzon_castaneus              Ichthyomyzon_unicuspis              Mordacia_mordax              Mordacia_lapicida              Caspiomyzon_wagneri              Tetrapleurodon_spadiceus              Entosphenus_macrostomus              Entosphenus_minimus              Entosphenus_similis              Entosphenus_tridentatus              Eudontomyzon_danfordi              Eudontomyzon_morii              Lampetra_ayresii              Mesomyzon              Yanliaomyzon_igensdentes              Yanliaomyzon_occisor              Priscomyzon              Mayomyzon              Hardistiella              Pipiscius              ;end;begin trees;       translate              1     Euconodonta,              2     Jamoytius,              3     Euphanerops,              4     Achanarella,              5     Ciderius,              6     Cornovichthys,              7     Lasanius,              8     Birkenia,              9     Rhyncholepis,              10   Myxinikela,              11   Tethymyxine,              12   Paramyxine_fernholmi,              13   Eptatretus_burgeri,              14   Eptatretus_stoutii,              15   Myxine_glutinosa,              16   Rubicundus_eos,              17   Rubicundus_lopheliae,              18   Neomyxine_biniplicata,              19   Myxineidus,              20   Gilpichthys,              21   Lethenteron_camtschaticum,              22   Petromyzon_marinus,              23   Lampetra_fluviatilis,              24   Geotria_australis,              25   Ichthyomyzon_bdellium,              26   Ichthyomyzon_castaneus,              27   Ichthyomyzon_unicuspis,              28   Mordacia_mordax,              29   Mordacia_lapicida,              30   Caspiomyzon_wagneri,              31   Tetrapleurodon_spadiceus,              32   Entosphenus_macrostomus,              33   Entosphenus_minimus,              34   Entosphenus_similis,              35   Entosphenus_tridentatus,              36   Eudontomyzon_danfordi,              37   Eudontomyzon_morii,              38   Lampetra_ayresii,              39   Mesomyzon,              40   Yanliaomyzon_igensdentes,              41   Yanliaomyzon_occisor,              42   Priscomyzon,              43   Mayomyzon,              44   Hardistiella,              45   Pipiscius              ;   tree con_all_compat = [&R] (1[&prob=1.00000000e+00,prob_stddev=0.00000000e+00,prob_range={1.00000000e+00,1.00000000e+00},prob(percent)="100",prob+-sd="100+-0",height_mean=1.83504461e+00,height_median=1.76515402e+00,height_95%HPD={1.04330569e+00,2.78154754e+00},age_mean=5.35500020e+02,age_median=5.35500020e+02,age_95%HPD={5.35499919e+02,5.35500123e+02}]:6.313825e+01[&length_mean=2.30897375e-01,length_median=2.07147600e-01,length_95%HPD={4.06082200e-03,4.86892300e-01},brlenIlnBrlens{1}_mean=0.500937,brlenIlnBrlens{1}_median=0.340735,brlenIlnBrlens{1}_95%HPD={0.000019,1.502505},rateIlnBrlens{1}_mean=2.430049,rateIlnBrlens{1}_median=1.567105,rateIlnBrlens{1}_95%HPD={0.014700,7.338215},brlenIlnBrlens{2}_mean=0.226535,brlenIlnBrlens{2}_median=0.157888,brlenIlnBrlens{2}_95%HPD={0.000023,0.636245},rateIlnBrlens{2}_mean=0.980756,rateIlnBrlens{2}_median=0.838133,rateIlnBrlens{2}_95%HPD={0.017830,2.332908},brlenIlnBrlens{3}_mean=0.160286,brlenIlnBrlens{3}_median=0.075647,brlenIlnBrlens{3}_95%HPD={0.000006,0.579688},rateIlnBrlens{3}_mean=0.736804,rateIlnBrlens{3}_median=0.388846,rateIlnBrlens{3}_95%HPD={0.002411,2.483404},brlenIlnBrlens{4,5}_mean=0.227494,brlenIlnBrlens{4,5}_median=0.154715,brlenIlnBrlens{4,5}_95%HPD={0.000010,0.665436},rateIlnBrlens{4,5}_mean=0.987887,rateIlnBrlens{4,5}_median=0.798620,rateIlnBrlens{4,5}_95%HPD={0.055397,2.374800}],(((2[&prob=1.00000000e+00,prob_stddev=0.00000000e+00,prob_range={1.00000000e+00,1.00000000e+00},prob(percent)="100",prob+-sd="100+-0",height_mean=1.49202321e+00,height_median=1.43519712e+00,height_95%HPD={8.48282569e-01,2.26159807e+00},age_mean=4.35400020e+02,age_median=4.35400020e+02,age_95%HPD={4.35399931e+02,4.35400110e+02}]:1.975078e-05[&length_mean=2.06791040e-02,length_median=0.00000000e+00,length_95%HPD={0.00000000e+00,1.31082700e-01},brlenIlnBrlens{1}_mean=0.010395,brlenIlnBrlens{1}_median=0.000000,brlenIlnBrlens{1}_95%HPD={0.000000,0.062768},rateIlnBrlens{1}_mean=0.877814,rateIlnBrlens{1}_median=1.000000,rateIlnBrlens{1}_95%HPD={0.104361,1.000682},brlenIlnBrlens{2}_mean=0.019894,brlenIlnBrlens{2}_median=0.000000,brlenIlnBrlens{2}_95%HPD={0.000000,0.119709},rateIlnBrlens{2}_mean=0.990908,rateIlnBrlens{2}_median=1.000000,rateIlnBrlens{2}_95%HPD={0.254641,1.317673},brlenIlnBrlens{3}_mean=0.010064,brlenIlnBrlens{3}_median=0.000000,brlenIlnBrlens{3}_95%HPD={0.000000,0.055137},rateIlnBrlens{3}_mean=0.877116,rateIlnBrlens{3}_median=1.000000,rateIlnBrlens{3}_95%HPD={0.053539,1.000000},brlenIlnBrlens{4,5}_mean=0.016875,brlenIlnBrlens{4,5}_median=0.000000,brlenIlnBrlens{4,5}_95%HPD={0.000000,0.099532},rateIlnBrlens{4,5}_mean=0.948739,rateIlnBrlens{4,5}_median=1.000000,rateIlnBrlens{4,5}_95%HPD={0.179001,1.188516}],3[&prob=1.00000000e+00,prob_stddev=0.00000000e+00,prob_range={1.00000000e+00,1.00000000e+00},prob(percent)="100",prob+-sd="100+-0",height_mean=1.26791133e+00,height_median=1.21962092e+00,height_95%HPD={7.20864869e-01,1.92189087e+00},age_mean=3.70000020e+02,age_median=3.70000020e+02,age_95%HPD={3.69999937e+02,3.70000103e+02}]:6.540002e+01[&length_mean=2.34319084e-01,length_median=2.21727200e-01,length_95%HPD={1.04474400e-01,4.14163700e-01},brlenIlnBrlens{1}_mean=0.154832,brlenIlnBrlens{1}_median=0.123043,brlenIlnBrlens{1}_95%HPD={0.008853,0.391330},rateIlnBrlens{1}_mean=0.696927,rateIlnBrlens{1}_median=0.549355,rateIlnBrlens{1}_95%HPD={0.046209,1.724795},brlenIlnBrlens{2}_mean=0.351301,brlenIlnBrlens{2}_median=0.259565,brlenIlnBrlens{2}_95%HPD={0.015441,0.908239},rateIlnBrlens{2}_mean=1.506081,rateIlnBrlens{2}_median=1.133124,rateIlnBrlens{2}_95%HPD={0.100717,3.771675},brlenIlnBrlens{3}_mean=0.104702,brlenIlnBrlens{3}_median=0.062265,brlenIlnBrlens{3}_95%HPD={0.000000,0.338461},rateIlnBrlens{3}_mean=0.457893,rateIlnBrlens{3}_median=0.286000,rateIlnBrlens{3}_95%HPD={0.001661,1.459329},brlenIlnBrlens{4,5}_mean=0.232548,brlenIlnBrlens{4,5}_median=0.172744,brlenIlnBrlens{4,5}_95%HPD={0.007533,0.605075},rateIlnBrlens{4,5}_mean=0.994654,rateIlnBrlens{4,5}_median=0.792929,rateIlnBrlens{4,5}_95%HPD={0.055302,2.378603}])[&prob=9.08952730e-01,prob_stddev=6.12186454e-03,prob_range={9.04623918e-01,9.13281542e-01},prob(percent)="91",prob+-sd="91+-1",height_mean=1.50348664e+00,height_median=1.44634236e+00,height_95%HPD={8.56605521e-01,2.28722223e+00},age_mean=4.39505833e+02,age_median=4.35400039e+02,age_95%HPD={4.35399837e+02,4.63006935e+02}]:6.283449e+01[&length_mean=1.92138909e-01,length_median=1.74602000e-01,length_95%HPD={2.52575600e-02,4.01838000e-01},brlenIlnBrlens{1}_mean=0.126019,brlenIlnBrlens{1}_median=0.086216,brlenIlnBrlens{1}_95%HPD={0.000002,0.375188},rateIlnBrlens{1}_mean=0.711888,rateIlnBrlens{1}_median=0.516878,rateIlnBrlens{1}_95%HPD={0.006759,1.986621},brlenIlnBrlens{2}_mean=0.313692,brlenIlnBrlens{2}_median=0.222772,brlenIlnBrlens{2}_95%HPD={0.000001,0.876329},rateIlnBrlens{2}_mean=1.804232,rateIlnBrlens{2}_median=1.203095,rateIlnBrlens{2}_95%HPD={0.036433,5.132055},brlenIlnBrlens{3}_mean=0.243477,brlenIlnBrlens{3}_median=0.129046,brlenIlnBrlens{3}_95%HPD={0.000004,0.832756},rateIlnBrlens{3}_mean=1.298188,rateIlnBrlens{3}_median=0.759412,rateIlnBrlens{3}_95%HPD={0.005685,4.222813},brlenIlnBrlens{4,5}_mean=0.195424,brlenIlnBrlens{4,5}_median=0.133342,brlenIlnBrlens{4,5}_95%HPD={0.000008,0.558899},rateIlnBrlens{4,5}_mean=1.009597,rateIlnBrlens{4,5}_median=0.810017,rateIlnBrlens{4,5}_95%HPD={0.058054,2.439874}],(7[&prob=1.00000000e+00,prob_stddev=0.00000000e+00,prob_range={1.00000000e+00,1.00000000e+00},prob(percent)="100",prob+-sd="100+-0",height_mean=1.46735034e+00,height_median=1.41146396e+00,height_95%HPD={8.34254929e-01,2.22419914e+00},age_mean=4.28200020e+02,age_median=4.28200019e+02,age_95%HPD={4.28199931e+02,4.28200108e+02}]:3.656179e+01[&length_mean=1.42862558e-01,length_median=1.28462700e-01,length_95%HPD={3.75577800e-02,2.94239900e-01},brlenIlnBrlens{1}_mean=0.065478,brlenIlnBrlens{1}_median=0.046241,brlenIlnBrlens{1}_95%HPD={0.000000,0.188567},rateIlnBrlens{1}_mean=0.495206,rateIlnBrlens{1}_median=0.362896,rateIlnBrlens{1}_95%HPD={0.008986,1.343852},brlenIlnBrlens{2}_mean=0.126029,brlenIlnBrlens{2}_median=0.097103,brlenIlnBrlens{2}_95%HPD={0.000000,0.329946},rateIlnBrlens{2}_mean=0.906444,rateIlnBrlens{2}_median=0.800810,rateIlnBrlens{2}_95%HPD={0.037366,2.027263},brlenIlnBrlens{3}_mean=0.095018,brlenIlnBrlens{3}_median=0.048761,brlenIlnBrlens{3}_95%HPD={0.000000,0.329979},rateIlnBrlens{3}_mean=0.698603,rateIlnBrlens{3}_median=0.384575,rateIlnBrlens{3}_95%HPD={0.002086,2.290179},brlenIlnBrlens{4,5}_mean=0.142425,brlenIlnBrlens{4,5}_median=0.100350,brlenIlnBrlens{4,5}_95%HPD={0.000000,0.394472},rateIlnBrlens{4,5}_mean=0.995549,rateIlnBrlens{4,5}_median=0.810255,rateIlnBrlens{4,5}_95%HPD={0.035734,2.371436}],(8[&prob=1.00000000e+00,prob_stddev=0.00000000e+00,prob_range={1.00000000e+00,1.00000000e+00},prob(percent)="100",prob+-sd="100+-0",height_mean=1.49202321e+00,height_median=1.43519713e+00,height_95%HPD={8.48282569e-01,2.26159814e+00},age_mean=4.35400020e+02,age_median=4.35400019e+02,age_95%HPD={4.35399931e+02,4.35400109e+02}]:7.250804e-06[&length_mean=1.90021228e-03,length_median=0.00000000e+00,length_95%HPD={0.00000000e+00,1.36216000e-02},brlenIlnBrlens{1}_mean=0.001169,brlenIlnBrlens{1}_median=0.000000,brlenIlnBrlens{1}_95%HPD={0.000000,0.007553},rateIlnBrlens{1}_mean=0.974213,rateIlnBrlens{1}_median=1.000000,rateIlnBrlens{1}_95%HPD={0.370334,1.000242},brlenIlnBrlens{2}_mean=0.001606,brlenIlnBrlens{2}_median=0.000000,brlenIlnBrlens{2}_95%HPD={0.000000,0.010121},rateIlnBrlens{2}_mean=0.985537,rateIlnBrlens{2}_median=1.000000,rateIlnBrlens{2}_95%HPD={0.708827,1.106988},brlenIlnBrlens{3}_mean=0.001106,brlenIlnBrlens{3}_median=0.000000,brlenIlnBrlens{3}_95%HPD={0.000000,0.005033},rateIlnBrlens{3}_mean=0.961665,rateIlnBrlens{3}_median=1.000000,rateIlnBrlens{3}_95%HPD={0.264931,1.000000},brlenIlnBrlens{4,5}_mean=0.001477,brlenIlnBrlens{4,5}_median=0.000000,brlenIlnBrlens{4,5}_95%HPD={0.000000,0.008829},rateIlnBrlens{4,5}_mean=0.978229,rateIlnBrlens{4,5}_median=1.000000,rateIlnBrlens{4,5}_95%HPD={0.494287,1.000000}],9[&prob=1.00000000e+00,prob_stddev=0.00000000e+00,prob_range={1.00000000e+00,1.00000000e+00},prob(percent)="100",prob+-sd="100+-0",height_mean=1.48020080e+00,height_median=1.42382499e+00,height_95%HPD={8.41560991e-01,2.24367781e+00},age_mean=4.31950020e+02,age_median=4.31950019e+02,age_95%HPD={4.31949932e+02,4.31950109e+02}]:3.450007e+00[&length_mean=1.36710163e-02,length_median=1.17295000e-02,length_95%HPD={5.67236400e-03,2.76434700e-02},brlenIlnBrlens{1}_mean=0.010972,brlenIlnBrlens{1}_median=0.008267,brlenIlnBrlens{1}_95%HPD={0.000000,0.029045},rateIlnBrlens{1}_mean=0.864511,rateIlnBrlens{1}_median=0.679464,rateIlnBrlens{1}_95%HPD={0.024647,2.245427},brlenIlnBrlens{2}_mean=0.017206,brlenIlnBrlens{2}_median=0.012622,brlenIlnBrlens{2}_95%HPD={0.000000,0.045298},rateIlnBrlens{2}_mean=1.305809,rateIlnBrlens{2}_median=1.028628,rateIlnBrlens{2}_95%HPD={0.050584,3.187171},brlenIlnBrlens{3}_mean=0.011591,brlenIlnBrlens{3}_median=0.005329,brlenIlnBrlens{3}_95%HPD={0.000000,0.042158},rateIlnBrlens{3}_mean=0.870625,rateIlnBrlens{3}_median=0.438010,rateIlnBrlens{3}_95%HPD={0.001690,3.059072},brlenIlnBrlens{4,5}_mean=0.013624,brlenIlnBrlens{4,5}_median=0.009480,brlenIlnBrlens{4,5}_95%HPD={0.000000,0.037168},rateIlnBrlens{4,5}_mean=1.000221,rateIlnBrlens{4,5}_median=0.807058,rateIlnBrlens{4,5}_95%HPD={0.055595,2.428102}])[&prob=9.94888980e-01,prob_stddev=6.41098751e-04,prob_range={9.94435654e-01,9.95342305e-01},prob(percent)="99",prob+-sd="99+-0",height_mean=1.49402715e+00,height_median=1.43732175e+00,height_95%HPD={8.48515979e-01,2.26211779e+00},age_mean=4.35982638e+02,age_median=4.35400027e+02,age_95%HPD={4.35399816e+02,4.39578260e+02}]:2.936178e+01[&length_mean=1.32033126e-01,length_median=1.12481700e-01,length_95%HPD={1.64197300e-02,3.00076000e-01},brlenIlnBrlens{1}_mean=0.189151,brlenIlnBrlens{1}_median=0.141648,brlenIlnBrlens{1}_95%HPD={0.001602,0.500228},rateIlnBrlens{1}_mean=1.695217,rateIlnBrlens{1}_median=1.282666,rateIlnBrlens{1}_95%HPD={0.032747,4.462697},brlenIlnBrlens{2}_mean=0.131017,brlenIlnBrlens{2}_median=0.094894,brlenIlnBrlens{2}_95%HPD={0.001360,0.358013},rateIlnBrlens{2}_mean=1.034590,rateIlnBrlens{2}_median=0.898428,rateIlnBrlens{2}_95%HPD={0.035532,2.408092},brlenIlnBrlens{3}_mean=0.221223,brlenIlnBrlens{3}_median=0.119245,brlenIlnBrlens{3}_95%HPD={0.000040,0.748630},rateIlnBrlens{3}_mean=1.833421,rateIlnBrlens{3}_median=1.083088,rateIlnBrlens{3}_95%HPD={0.001883,5.904486},brlenIlnBrlens{4,5}_mean=0.132268,brlenIlnBrlens{4,5}_median=0.086779,brlenIlnBrlens{4,5}_95%HPD={0.001914,0.390703},rateIlnBrlens{4,5}_mean=1.002989,rateIlnBrlens{4,5}_median=0.801630,rateIlnBrlens{4,5}_95%HPD={0.051507,2.399140}])[&prob=7.47586710e-01,prob_stddev=5.59390087e-03,prob_range={7.43631224e-01,7.51542195e-01},prob(percent)="75",prob+-sd="75+-1",height_mean=1.60464184e+00,height_median=1.54310140e+00,height_95%HPD={9.07292265e-01,2.40713975e+00},age_mean=4.68176340e+02,age_median=4.64761808e+02,age_95%HPD={4.40930619e+02,5.04008051e+02}]:3.347272e+01[&length_mean=1.33929316e-01,length_median=1.13841700e-01,length_95%HPD={1.42253500e-06,3.16547500e-01},brlenIlnBrlens{1}_mean=0.097617,brlenIlnBrlens{1}_median=0.058516,brlenIlnBrlens{1}_95%HPD={0.000001,0.323122},rateIlnBrlens{1}_mean=0.795447,rateIlnBrlens{1}_median=0.550664,rateIlnBrlens{1}_95%HPD={0.002376,2.293971},brlenIlnBrlens{2}_mean=0.201200,brlenIlnBrlens{2}_median=0.129074,brlenIlnBrlens{2}_95%HPD={0.000001,0.618485},rateIlnBrlens{2}_mean=1.561114,rateIlnBrlens{2}_median=1.076059,rateIlnBrlens{2}_95%HPD={0.026541,4.296566},brlenIlnBrlens{3}_mean=0.158155,brlenIlnBrlens{3}_median=0.057594,brlenIlnBrlens{3}_95%HPD={0.000000,0.625297},rateIlnBrlens{3}_mean=1.156409,rateIlnBrlens{3}_median=0.563339,rateIlnBrlens{3}_95%HPD={0.002210,4.131282},brlenIlnBrlens{4,5}_mean=0.133425,brlenIlnBrlens{4,5}_median=0.084972,brlenIlnBrlens{4,5}_95%HPD={0.000001,0.409212},rateIlnBrlens{4,5}_mean=0.993059,rateIlnBrlens{4,5}_median=0.804368,rateIlnBrlens{4,5}_95%HPD={0.034655,2.368411}])[&prob=7.56973209e-01,prob_stddev=1.60274688e-02,prob_range={7.45640078e-01,7.68306341e-01},prob(percent)="76",prob+-sd="76+-2",height_mean=1.71613912e+00,height_median=1.65165454e+00,height_95%HPD={9.71877978e-01,2.56887881e+00},age_mean=5.02374875e+02,age_median=4.98234531e+02,age_95%HPD={4.57206752e+02,5.55672608e+02}]:6.366543e+01[&length_mean=1.95010010e-01,length_median=1.75789900e-01,length_95%HPD={5.45522400e-03,4.12293800e-01},brlenIlnBrlens{1}_mean=0.210393,brlenIlnBrlens{1}_median=0.131673,brlenIlnBrlens{1}_95%HPD={0.000012,0.670440},rateIlnBrlens{1}_mean=1.168153,rateIlnBrlens{1}_median=0.789357,rateIlnBrlens{1}_95%HPD={0.009522,3.502039},brlenIlnBrlens{2}_mean=0.209671,brlenIlnBrlens{2}_median=0.141993,brlenIlnBrlens{2}_95%HPD={0.000006,0.595450},rateIlnBrlens{2}_mean=1.082143,rateIlnBrlens{2}_median=0.876476,rateIlnBrlens{2}_95%HPD={0.016217,2.725137},brlenIlnBrlens{3}_mean=0.722323,brlenIlnBrlens{3}_median=0.457406,brlenIlnBrlens{3}_95%HPD={0.000003,2.267745},rateIlnBrlens{3}_mean=4.105047,rateIlnBrlens{3}_median=2.583633,rateIlnBrlens{3}_95%HPD={0.003625,12.830036},brlenIlnBrlens{4,5}_mean=0.197903,brlenIlnBrlens{4,5}_median=0.132454,brlenIlnBrlens{4,5}_95%HPD={0.000008,0.574585},rateIlnBrlens{4,5}_mean=1.010985,rateIlnBrlens{4,5}_median=0.817049,rateIlnBrlens{4,5}_95%HPD={0.033320,2.462792}],(((4[&prob=1.00000000e+00,prob_stddev=0.00000000e+00,prob_range={1.00000000e+00,1.00000000e+00},prob(percent)="100",prob+-sd="100+-0",height_mean=1.31931314e+00,height_median=1.26906499e+00,height_95%HPD={7.50089089e-01,1.99980541e+00},age_mean=3.85000020e+02,age_median=3.85000019e+02,age_95%HPD={3.84999940e+02,3.85000102e+02}]:4.700002e+01[&length_mean=1.42900282e-01,length_median=1.37322500e-01,length_95%HPD={0.00000000e+00,2.82846200e-01},brlenIlnBrlens{1}_mean=0.132059,brlenIlnBrlens{1}_median=0.091798,brlenIlnBrlens{1}_95%HPD={0.000000,0.386506},rateIlnBrlens{1}_mean=1.059349,rateIlnBrlens{1}_median=0.771178,rateIlnBrlens{1}_95%HPD={0.011369,2.996621},brlenIlnBrlens{2}_mean=0.154175,brlenIlnBrlens{2}_median=0.111262,brlenIlnBrlens{2}_95%HPD={0.000000,0.427732},rateIlnBrlens{2}_mean=1.048227,rateIlnBrlens{2}_median=0.910977,rateIlnBrlens{2}_95%HPD={0.044000,2.399907},brlenIlnBrlens{3}_mean=0.094133,brlenIlnBrlens{3}_median=0.045665,brlenIlnBrlens{3}_95%HPD={0.000000,0.339417},rateIlnBrlens{3}_mean=0.697828,rateIlnBrlens{3}_median=0.393423,rateIlnBrlens{3}_95%HPD={0.001417,2.327542},brlenIlnBrlens{4,5}_mean=0.139631,brlenIlnBrlens{4,5}_median=0.098329,brlenIlnBrlens{4,5}_95%HPD={0.000000,0.393035},rateIlnBrlens{4,5}_mean=0.975493,rateIlnBrlens{4,5}_median=0.816864,rateIlnBrlens{4,5}_95%HPD={0.062461,2.323336}],5[&prob=1.00000000e+00,prob_stddev=0.00000000e+00,prob_range={1.00000000e+00,1.00000000e+00},prob(percent)="100",prob+-sd="100+-0",height_mean=1.48037213e+00,height_median=1.42398981e+00,height_95%HPD={8.41658409e-01,2.24393750e+00},age_mean=4.32000020e+02,age_median=4.32000020e+02,age_95%HPD={4.31999935e+02,4.32000106e+02}]:2.186286e-05[&length_mean=2.99834509e-02,length_median=0.00000000e+00,length_95%HPD={0.00000000e+00,1.72770600e-01},brlenIlnBrlens{1}_mean=0.015611,brlenIlnBrlens{1}_median=0.000000,brlenIlnBrlens{1}_95%HPD={0.000000,0.089481},rateIlnBrlens{1}_mean=0.860524,rateIlnBrlens{1}_median=1.000000,rateIlnBrlens{1}_95%HPD={0.044948,1.000597},brlenIlnBrlens{2}_mean=0.028410,brlenIlnBrlens{2}_median=0.000000,brlenIlnBrlens{2}_95%HPD={0.000000,0.163182},rateIlnBrlens{2}_mean=0.980979,rateIlnBrlens{2}_median=1.000000,rateIlnBrlens{2}_95%HPD={0.180392,1.372314},brlenIlnBrlens{3}_mean=0.013896,brlenIlnBrlens{3}_median=0.000000,brlenIlnBrlens{3}_95%HPD={0.000000,0.069379},rateIlnBrlens{3}_mean=0.825580,rateIlnBrlens{3}_median=1.000000,rateIlnBrlens{3}_95%HPD={0.040842,1.000000},brlenIlnBrlens{4,5}_mean=0.024707,brlenIlnBrlens{4,5}_median=0.000000,brlenIlnBrlens{4,5}_95%HPD={0.000000,0.138263},rateIlnBrlens{4,5}_mean=0.940086,rateIlnBrlens{4,5}_median=1.000000,rateIlnBrlens{4,5}_95%HPD={0.142095,1.271953}])[&prob=4.78098167e-01,prob_stddev=7.37892092e-03,prob_range={4.72880482e-01,4.83315852e-01},prob(percent)="48",prob+-sd="48+-1",height_mean=1.46637912e+00,height_median=1.41116097e+00,height_95%HPD={8.26439440e-01,2.22354637e+00},age_mean=4.35932612e+02,age_median=4.32000041e+02,age_95%HPD={4.31999850e+02,4.57294034e+02}]:6.895629e+00[&length_mean=1.51390076e-01,length_median=1.28010500e-01,length_95%HPD={1.16061600e-05,3.59013700e-01},brlenIlnBrlens{1}_mean=0.130465,brlenIlnBrlens{1}_median=0.080953,brlenIlnBrlens{1}_95%HPD={0.000004,0.417701},rateIlnBrlens{1}_mean=0.952074,rateIlnBrlens{1}_median=0.667559,rateIlnBrlens{1}_95%HPD={0.006693,2.704750},brlenIlnBrlens{2}_mean=0.217433,brlenIlnBrlens{2}_median=0.137830,brlenIlnBrlens{2}_95%HPD={0.000006,0.663884},rateIlnBrlens{2}_mean=1.472121,rateIlnBrlens{2}_median=1.080774,rateIlnBrlens{2}_95%HPD={0.068071,3.804540},brlenIlnBrlens{3}_mean=0.111878,brlenIlnBrlens{3}_median=0.049988,brlenIlnBrlens{3}_95%HPD={0.000001,0.413776},rateIlnBrlens{3}_mean=0.795523,rateIlnBrlens{3}_median=0.424321,rateIlnBrlens{3}_95%HPD={0.001439,2.676884},brlenIlnBrlens{4,5}_mean=0.157067,brlenIlnBrlens{4,5}_median=0.097172,brlenIlnBrlens{4,5}_95%HPD={0.000006,0.489584},rateIlnBrlens{4,5}_mean=1.024407,rateIlnBrlens{4,5}_median=0.828497,rateIlnBrlens{4,5}_95%HPD={0.061093,2.467512}],6[&prob=1.00000000e+00,prob_stddev=0.00000000e+00,prob_range={1.00000000e+00,1.00000000e+00},prob(percent)="100",prob+-sd="100+-0",height_mean=1.31931314e+00,height_median=1.26906499e+00,height_95%HPD={7.50089089e-01,1.99980541e+00},age_mean=3.85000020e+02,age_median=3.85000019e+02,age_95%HPD={3.84999941e+02,3.85000101e+02}]:5.389565e+01[&length_mean=1.61876089e-01,length_median=1.32726000e-01,length_95%HPD={0.00000000e+00,4.24137400e-01},brlenIlnBrlens{1}_mean=0.134765,brlenIlnBrlens{1}_median=0.079201,brlenIlnBrlens{1}_95%HPD={0.000000,0.455437},rateIlnBrlens{1}_mean=0.965467,rateIlnBrlens{1}_median=0.778028,rateIlnBrlens{1}_95%HPD={0.005123,2.595226},brlenIlnBrlens{2}_mean=0.182445,brlenIlnBrlens{2}_median=0.119633,brlenIlnBrlens{2}_95%HPD={0.000000,0.572919},rateIlnBrlens{2}_mean=1.130180,rateIlnBrlens{2}_median=1.000000,rateIlnBrlens{2}_95%HPD={0.059562,2.544029},brlenIlnBrlens{3}_mean=0.093619,brlenIlnBrlens{3}_median=0.038724,brlenIlnBrlens{3}_95%HPD={0.000000,0.357408},rateIlnBrlens{3}_mean=0.676320,rateIlnBrlens{3}_median=0.430362,rateIlnBrlens{3}_95%HPD={0.004422,1.904829},brlenIlnBrlens{4,5}_mean=0.154530,brlenIlnBrlens{4,5}_median=0.094454,brlenIlnBrlens{4,5}_95%HPD={0.000000,0.502109},rateIlnBrlens{4,5}_mean=0.957881,rateIlnBrlens{4,5}_median=0.882253,rateIlnBrlens{4,5}_95%HPD={0.068787,2.139923}])[&prob=3.31211890e-01,prob_stddev=7.19036246e-03,prob_range={3.26127536e-01,3.36296244e-01},prob(percent)="33",prob+-sd="33+-1",height_mean=1.52989511e+00,height_median=1.47326538e+00,height_95%HPD={8.51985287e-01,2.28648369e+00},age_mean=4.49946007e+02,age_median=4.38895671e+02,age_95%HPD={4.31999851e+02,4.99501701e+02}]:6.073687e+01[&length_mean=1.50898632e-01,length_median=1.27499800e-01,length_95%HPD={9.52038600e-06,3.64551700e-01},brlenIlnBrlens{1}_mean=0.138583,brlenIlnBrlens{1}_median=0.079757,brlenIlnBrlens{1}_95%HPD={0.000008,0.471315},rateIlnBrlens{1}_mean=1.002418,rateIlnBrlens{1}_median=0.668077,rateIlnBrlens{1}_95%HPD={0.010560,2.956206},brlenIlnBrlens{2}_mean=0.212606,brlenIlnBrlens{2}_median=0.131226,brlenIlnBrlens{2}_95%HPD={0.000002,0.684746},rateIlnBrlens{2}_mean=1.389255,rateIlnBrlens{2}_median=1.032906,rateIlnBrlens{2}_95%HPD={0.026090,3.752553},brlenIlnBrlens{3}_mean=0.107280,brlenIlnBrlens{3}_median=0.044901,brlenIlnBrlens{3}_95%HPD={0.000003,0.397127},rateIlnBrlens{3}_mean=0.758067,rateIlnBrlens{3}_median=0.386475,rateIlnBrlens{3}_95%HPD={0.001644,2.575776},brlenIlnBrlens{4,5}_mean=0.156550,brlenIlnBrlens{4,5}_median=0.095743,brlenIlnBrlens{4,5}_95%HPD={0.000002,0.494073},rateIlnBrlens{4,5}_mean=1.017377,rateIlnBrlens{4,5}_median=0.819077,rateIlnBrlens{4,5}_95%HPD={0.053179,2.500411}],(((10[&prob=1.00000000e+00,prob_stddev=0.00000000e+00,prob_range={1.00000000e+00,1.00000000e+00},prob(percent)="100",prob+-sd="100+-0",height_mean=1.06230410e+00,height_median=1.02184452e+00,height_95%HPD={6.03967859e-01,1.61023293e+00},age_mean=3.10000020e+02,age_median=3.10000019e+02,age_95%HPD={3.09999956e+02,3.10000088e+02}]:5.128953e-06[&length_mean=3.03898775e-02,length_median=0.00000000e+00,length_95%HPD={0.00000000e+00,2.25769500e-01},brlenIlnBrlens{1}_mean=0.050227,brlenIlnBrlens{1}_median=0.000000,brlenIlnBrlens{1}_95%HPD={0.000000,0.292756},rateIlnBrlens{1}_mean=1.088708,rateIlnBrlens{1}_median=1.000000,rateIlnBrlens{1}_95%HPD={0.102995,1.689577},brlenIlnBrlens{2}_mean=0.030532,brlenIlnBrlens{2}_median=0.000000,brlenIlnBrlens{2}_95%HPD={0.000000,0.203347},rateIlnBrlens{2}_mean=0.996700,rateIlnBrlens{2}_median=1.000000,rateIlnBrlens{2}_95%HPD={0.437543,1.238162},brlenIlnBrlens{3}_mean=0.013783,brlenIlnBrlens{3}_median=0.000000,brlenIlnBrlens{3}_95%HPD={0.000000,0.066771},rateIlnBrlens{3}_mean=0.914641,rateIlnBrlens{3}_median=1.000000,rateIlnBrlens{3}_95%HPD={0.114868,1.000000},brlenIlnBrlens{4,5}_mean=0.025846,brlenIlnBrlens{4,5}_median=0.000000,brlenIlnBrlens{4,5}_95%HPD={0.000000,0.156181},rateIlnBrlens{4,5}_mean=0.966142,rateIlnBrlens{4,5}_median=1.000000,rateIlnBrlens{4,5}_95%HPD={0.257480,1.000000}],(11[&prob=1.00000000e+00,prob_stddev=0.00000000e+00,prob_range={1.00000000e+00,1.00000000e+00},prob(percent)="100",prob+-sd="100+-0",height_mean=3.25544853e-01,height_median=3.13145920e-01,height_95%HPD={1.85086930e-01,4.93458931e-01},age_mean=9.50000202e+01,age_median=9.50000162e+01,age_95%HPD={9.49999847e+01,9.50000658e+01}]:5.880694e-06[&length_mean=5.08432521e-03,length_median=0.00000000e+00,length_95%HPD={0.00000000e+00,3.65464000e-02},brlenIlnBrlens{1}_mean=0.004211,brlenIlnBrlens{1}_median=0.000000,brlenIlnBrlens{1}_95%HPD={0.000000,0.023288},rateIlnBrlens{1}_mean=0.994611,rateIlnBrlens{1}_median=1.000000,rateIlnBrlens{1}_95%HPD={0.097351,1.000000},brlenIlnBrlens{2}_mean=0.004325,brlenIlnBrlens{2}_median=0.000000,brlenIlnBrlens{2}_95%HPD={0.000000,0.026390},rateIlnBrlens{2}_mean=0.981267,rateIlnBrlens{2}_median=1.000000,rateIlnBrlens{2}_95%HPD={0.530351,1.093189},brlenIlnBrlens{3}_mean=0.001812,brlenIlnBrlens{3}_median=0.000000,brlenIlnBrlens{3}_95%HPD={0.000000,0.008017},rateIlnBrlens{3}_mean=0.926992,rateIlnBrlens{3}_median=1.000000,rateIlnBrlens{3}_95%HPD={0.154388,1.000000},brlenIlnBrlens{4,5}_mean=0.003521,brlenIlnBrlens{4,5}_median=0.000000,brlenIlnBrlens{4,5}_95%HPD={0.000000,0.020815},rateIlnBrlens{4,5}_mean=0.962856,rateIlnBrlens{4,5}_median=1.000000,rateIlnBrlens{4,5}_95%HPD={0.407184,1.000000}],((((12[&prob=1.00000000e+00,prob_stddev=0.00000000e+00,prob_range={1.00000000e+00,1.00000000e+00},prob(percent)="100",prob+-sd="100+-0",height_mean=6.97795325e-08,height_median=4.90000003e-08,height_95%HPD={0.00000000e+00,2.07999998e-07},age_mean=2.01858522e-05,age_median=1.44332405e-05,age_95%HPD={0.00000000e+00,6.19344089e-05}]:1.057812e+01[&length_mean=3.74509781e-02,length_median=3.48691700e-02,length_95%HPD={1.33253700e-02,6.69999000e-02},brlenIlnBrlens{1}_mean=0.023553,brlenIlnBrlens{1}_median=0.017920,brlenIlnBrlens{1}_95%HPD={0.000436,0.063077},rateIlnBrlens{1}_mean=0.670064,rateIlnBrlens{1}_median=0.516276,rateIlnBrlens{1}_95%HPD={0.016815,1.768395},brlenIlnBrlens{2}_mean=0.049565,brlenIlnBrlens{2}_median=0.037810,brlenIlnBrlens{2}_95%HPD={0.000818,0.129202},rateIlnBrlens{2}_mean=1.364321,rateIlnBrlens{2}_median=1.051314,rateIlnBrlens{2}_95%HPD={0.061826,3.442504},brlenIlnBrlens{3}_mean=0.022013,brlenIlnBrlens{3}_median=0.012142,brlenIlnBrlens{3}_95%HPD={0.000076,0.074654},rateIlnBrlens{3}_mean=0.612334,rateIlnBrlens{3}_median=0.346698,rateIlnBrlens{3}_95%HPD={0.002928,2.040046},brlenIlnBrlens{4,5}_mean=0.024287,brlenIlnBrlens{4,5}_median=0.023652,brlenIlnBrlens{4,5}_95%HPD={0.011813,0.037425},rateIlnBrlens{4,5}_mean=0.734114,rateIlnBrlens{4,5}_median=0.676056,rateIlnBrlens{4,5}_95%HPD={0.229619,1.378628}],13[&prob=1.00000000e+00,prob_stddev=0.00000000e+00,prob_range={1.00000000e+00,1.00000000e+00},prob(percent)="100",prob+-sd="100+-0",height_mean=6.97620814e-08,height_median=4.99999993e-08,height_95%HPD={0.00000000e+00,2.08000000e-07},age_mean=2.01805454e-05,age_median=1.44441541e-05,age_95%HPD={0.00000000e+00,6.19110082e-05}]:1.057812e+01[&length_mean=4.35488400e-02,length_median=4.00434000e-02,length_95%HPD={1.35424300e-02,8.11882100e-02},brlenIlnBrlens{1}_mean=0.019423,brlenIlnBrlens{1}_median=0.014034,brlenIlnBrlens{1}_95%HPD={0.000221,0.054218},rateIlnBrlens{1}_mean=0.472719,rateIlnBrlens{1}_median=0.355106,rateIlnBrlens{1}_95%HPD={0.006074,1.278501},brlenIlnBrlens{2}_mean=0.043689,brlenIlnBrlens{2}_median=0.034248,brlenIlnBrlens{2}_95%HPD={0.000726,0.108343},rateIlnBrlens{2}_mean=1.037998,rateIlnBrlens{2}_median=0.884123,rateIlnBrlens{2}_95%HPD={0.031418,2.466303},brlenIlnBrlens{3}_mean=0.024536,brlenIlnBrlens{3}_median=0.013405,brlenIlnBrlens{3}_95%HPD={0.000066,0.082476},rateIlnBrlens{3}_mean=0.582893,rateIlnBrlens{3}_median=0.330283,rateIlnBrlens{3}_95%HPD={0.002413,1.937698},brlenIlnBrlens{4,5}_mean=0.046210,brlenIlnBrlens{4,5}_median=0.045483,brlenIlnBrlens{4,5}_95%HPD={0.025481,0.067777},rateIlnBrlens{4,5}_mean=1.270803,rateIlnBrlens{4,5}_median=1.155853,rateIlnBrlens{4,5}_95%HPD={0.258621,2.489893}])[&prob=6.77712396e-01,prob_stddev=9.55362845e-03,prob_range={6.70956961e-01,6.84467832e-01},prob(percent)="68",prob+-sd="68+-1",height_mean=3.73945805e-02,height_median=3.46590600e-02,height_95%HPD={1.25330800e-02,6.69428000e-02},age_mean=1.13118543e+01,age_median=1.05781380e+01,age_95%HPD={3.46365954e+00,2.02811519e+01}]:5.310370e+00[&length_mean=1.80512162e-02,length_median=1.48639600e-02,length_95%HPD={6.48899400e-04,4.37244300e-02},brlenIlnBrlens{1}_mean=0.012137,brlenIlnBrlens{1}_median=0.007554,brlenIlnBrlens{1}_95%HPD={0.000017,0.038841},rateIlnBrlens{1}_mean=0.754754,rateIlnBrlens{1}_median=0.532036,rateIlnBrlens{1}_95%HPD={0.008025,2.184796},brlenIlnBrlens{2}_mean=0.017718,brlenIlnBrlens{2}_median=0.011258,brlenIlnBrlens{2}_95%HPD={0.000005,0.053560},rateIlnBrlens{2}_mean=0.996582,rateIlnBrlens{2}_median=0.830232,rateIlnBrlens{2}_95%HPD={0.033959,2.396635},brlenIlnBrlens{3}_mean=0.012732,brlenIlnBrlens{3}_median=0.005626,brlenIlnBrlens{3}_95%HPD={0.000002,0.046969},rateIlnBrlens{3}_mean=0.754949,rateIlnBrlens{3}_median=0.396779,rateIlnBrlens{3}_95%HPD={0.000447,2.600896},brlenIlnBrlens{4,5}_mean=0.012738,brlenIlnBrlens{4,5}_median=0.011707,brlenIlnBrlens{4,5}_95%HPD={0.000838,0.026087},rateIlnBrlens{4,5}_mean=0.919167,rateIlnBrlens{4,5}_median=0.774359,rateIlnBrlens{4,5}_95%HPD={0.090799,2.053284}],14[&prob=1.00000000e+00,prob_stddev=0.00000000e+00,prob_range={1.00000000e+00,1.00000000e+00},prob(percent)="100",prob+-sd="100+-0",height_mean=6.97493555e-08,height_median=4.99999997e-08,height_95%HPD={0.00000000e+00,2.09000000e-07},age_mean=2.01774414e-05,age_median=1.44104300e-05,age_95%HPD={0.00000000e+00,6.18932948e-05}]:1.588849e+01[&length_mean=4.95908893e-02,length_median=4.62831600e-02,length_95%HPD={1.69120800e-02,8.96077100e-02},brlenIlnBrlens{1}_mean=0.026408,brlenIlnBrlens{1}_median=0.020003,brlenIlnBrlens{1}_95%HPD={0.000502,0.070542},rateIlnBrlens{1}_mean=0.576044,rateIlnBrlens{1}_median=0.437009,rateIlnBrlens{1}_95%HPD={0.009227,1.512607},brlenIlnBrlens{2}_mean=0.063024,brlenIlnBrlens{2}_median=0.047464,brlenIlnBrlens{2}_95%HPD={0.001612,0.165895},rateIlnBrlens{2}_mean=1.305773,rateIlnBrlens{2}_median=1.015687,rateIlnBrlens{2}_95%HPD={0.070927,3.268597},brlenIlnBrlens{3}_mean=0.026998,brlenIlnBrlens{3}_median=0.014992,brlenIlnBrlens{3}_95%HPD={0.000031,0.090509},rateIlnBrlens{3}_mean=0.565436,rateIlnBrlens{3}_median=0.327107,rateIlnBrlens{3}_95%HPD={0.001266,1.878561},brlenIlnBrlens{4,5}_mean=0.032386,brlenIlnBrlens{4,5}_median=0.031848,brlenIlnBrlens{4,5}_95%HPD={0.014576,0.051143},rateIlnBrlens{4,5}_mean=0.770751,rateIlnBrlens{4,5}_median=0.681054,rateIlnBrlens{4,5}_95%HPD={0.150833,1.572967}])[&prob=1.00000000e+00,prob_stddev=0.00000000e+00,prob_range={1.00000000e+00,1.00000000e+00},prob(percent)="100",prob+-sd="100+-0",height_mean=5.58760645e-02,height_median=5.23359300e-02,height_95%HPD={2.44333110e-02,9.62932100e-02},age_mean=1.69305598e+01,age_median=1.58885081e+01,age_95%HPD={6.49583789e+00,2.94951352e+01}]:4.010585e+01[&length_mean=1.37717429e-01,length_median=1.32021300e-01,length_95%HPD={4.30287000e-02,2.42176600e-01},brlenIlnBrlens{1}_mean=0.054262,brlenIlnBrlens{1}_median=0.041178,brlenIlnBrlens{1}_95%HPD={0.000953,0.146149},rateIlnBrlens{1}_mean=0.414559,rateIlnBrlens{1}_median=0.323589,rateIlnBrlens{1}_95%HPD={0.015184,1.092934},brlenIlnBrlens{2}_mean=0.137364,brlenIlnBrlens{2}_median=0.105008,brlenIlnBrlens{2}_95%HPD={0.001746,0.360140},rateIlnBrlens{2}_mean=0.995555,rateIlnBrlens{2}_median=0.858044,rateIlnBrlens{2}_95%HPD={0.035125,2.386004},brlenIlnBrlens{3}_mean=0.065620,brlenIlnBrlens{3}_median=0.039576,brlenIlnBrlens{3}_95%HPD={0.000161,0.212901},rateIlnBrlens{3}_mean=0.504896,rateIlnBrlens{3}_median=0.308588,rateIlnBrlens{3}_95%HPD={0.002822,1.623428},brlenIlnBrlens{4,5}_mean=0.131589,brlenIlnBrlens{4,5}_median=0.128137,brlenIlnBrlens{4,5}_95%HPD={0.070946,0.198421},rateIlnBrlens{4,5}_mean=1.091132,rateIlnBrlens{4,5}_median=0.979621,rateIlnBrlens{4,5}_95%HPD={0.404654,2.132790}],(15[&prob=1.00000000e+00,prob_stddev=0.00000000e+00,prob_range={1.00000000e+00,1.00000000e+00},prob(percent)="100",prob+-sd="100+-0",height_mean=6.96598825e-08,height_median=4.99999997e-08,height_95%HPD={0.00000000e+00,2.08000000e-07},age_mean=2.01705926e-05,age_median=1.45280167e-05,age_95%HPD={0.00000000e+00,6.17992688e-05}]:3.346178e+01[&length_mean=1.16430470e-01,length_median=1.10642300e-01,length_95%HPD={4.51840300e-02,2.00597000e-01},brlenIlnBrlens{1}_mean=0.050266,brlenIlnBrlens{1}_median=0.039093,brlenIlnBrlens{1}_95%HPD={0.000938,0.129185},rateIlnBrlens{1}_mean=0.463701,rateIlnBrlens{1}_median=0.362028,rateIlnBrlens{1}_95%HPD={0.011246,1.203799},brlenIlnBrlens{2}_mean=0.106371,brlenIlnBrlens{2}_median=0.085148,brlenIlnBrlens{2}_95%HPD={0.003580,0.261203},rateIlnBrlens{2}_mean=0.925326,rateIlnBrlens{2}_median=0.819755,rateIlnBrlens{2}_95%HPD={0.027534,2.054734},brlenIlnBrlens{3}_mean=0.052878,brlenIlnBrlens{3}_median=0.030999,brlenIlnBrlens{3}_95%HPD={0.000072,0.173785},rateIlnBrlens{3}_mean=0.470968,rateIlnBrlens{3}_median=0.285383,rateIlnBrlens{3}_95%HPD={0.001130,1.515417},brlenIlnBrlens{4,5}_mean=0.301904,brlenIlnBrlens{4,5}_median=0.295298,brlenIlnBrlens{4,5}_95%HPD={0.193588,0.419645},rateIlnBrlens{4,5}_mean=2.893772,rateIlnBrlens{4,5}_median=2.666667,rateIlnBrlens{4,5}_95%HPD={1.209297,5.060064}],18[&prob=1.00000000e+00,prob_stddev=0.00000000e+00,prob_range={1.00000000e+00,1.00000000e+00},prob(percent)="100",prob+-sd="100+-0",height_mean=6.96589403e-08,height_median=4.99999997e-08,height_95%HPD={0.00000000e+00,2.08000000e-07},age_mean=2.01702872e-05,age_median=1.45270267e-05,age_95%HPD={0.00000000e+00,6.18012084e-05}]:3.346178e+01[&length_mean=1.16425152e-01,length_median=1.10641100e-01,length_95%HPD={4.51806500e-02,2.00576300e-01},brlenIlnBrlens{1}_mean=0.046877,brlenIlnBrlens{1}_median=0.036708,brlenIlnBrlens{1}_95%HPD={0.001189,0.121091},rateIlnBrlens{1}_mean=0.433831,rateIlnBrlens{1}_median=0.336764,rateIlnBrlens{1}_95%HPD={0.010044,1.128560},brlenIlnBrlens{2}_mean=0.094595,brlenIlnBrlens{2}_median=0.076970,brlenIlnBrlens{2}_95%HPD={0.003074,0.229891},rateIlnBrlens{2}_mean=0.830238,rateIlnBrlens{2}_median=0.739896,rateIlnBrlens{2}_95%HPD={0.027875,1.864937},brlenIlnBrlens{3}_mean=0.052964,brlenIlnBrlens{3}_median=0.031408,brlenIlnBrlens{3}_95%HPD={0.000189,0.171786},rateIlnBrlens{3}_mean=0.471331,rateIlnBrlens{3}_median=0.284535,rateIlnBrlens{3}_95%HPD={0.002607,1.471530},brlenIlnBrlens{4,5}_mean=0.276596,brlenIlnBrlens{4,5}_median=0.270561,brlenIlnBrlens{4,5}_95%HPD={0.176297,0.389391},rateIlnBrlens{4,5}_mean=2.644035,rateIlnBrlens{4,5}_median=2.448384,rateIlnBrlens{4,5}_95%HPD={1.061869,4.541874}])[&prob=9.99688894e-01,prob_stddev=1.13135074e-04,prob_range={9.99608896e-01,9.99768893e-01},prob(percent)="100",prob+-sd="100+-0",height_mean=1.16399035e-01,height_median=1.10628680e-01,height_95%HPD={4.53326280e-02,2.00597227e-01},age_mean=3.55412574e+01,age_median=3.34617981e+01,age_95%HPD={1.17710678e+01,6.28372974e+01}]:2.253256e+01[&length_mean=7.76288798e-02,length_median=6.99398800e-02,length_95%HPD={1.67446900e-02,1.56832800e-01},brlenIlnBrlens{1}_mean=0.068034,brlenIlnBrlens{1}_median=0.052376,brlenIlnBrlens{1}_95%HPD={0.001631,0.178256},rateIlnBrlens{1}_mean=0.958696,rateIlnBrlens{1}_median=0.761452,rateIlnBrlens{1}_95%HPD={0.043237,2.427949},brlenIlnBrlens{2}_mean=0.101862,brlenIlnBrlens{2}_median=0.072438,brlenIlnBrlens{2}_95%HPD={0.001312,0.272411},rateIlnBrlens{2}_mean=1.331698,rateIlnBrlens{2}_median=1.027518,rateIlnBrlens{2}_95%HPD={0.044972,3.263947},brlenIlnBrlens{3}_mean=0.064589,brlenIlnBrlens{3}_median=0.036675,brlenIlnBrlens{3}_95%HPD={0.000157,0.216479},rateIlnBrlens{3}_mean=0.898487,rateIlnBrlens{3}_median=0.530531,rateIlnBrlens{3}_95%HPD={0.003072,2.873055},brlenIlnBrlens{4,5}_mean=0.059148,brlenIlnBrlens{4,5}_median=0.056373,brlenIlnBrlens{4,5}_95%HPD={0.018278,0.106682},rateIlnBrlens{4,5}_mean=0.903325,rateIlnBrlens{4,5}_median=0.805139,rateIlnBrlens{4,5}_95%HPD={0.198178,1.836945}])[&prob=8.96739614e-01,prob_stddev=1.16529126e-02,prob_range={8.88499760e-01,9.04979467e-01},prob(percent)="90",prob+-sd="90+-1",height_mean=1.90476569e-01,height_median=1.84223460e-01,height_95%HPD={1.00837580e-01,2.93333000e-01},age_mean=5.73463426e+01,age_median=5.59943539e+01,age_95%HPD={2.89732959e+01,8.80209689e+01}]:2.064845e+01[&length_mean=7.87714778e-02,length_median=6.87501800e-02,length_95%HPD={1.55295800e-05,1.73280300e-01},brlenIlnBrlens{1}_mean=0.060140,brlenIlnBrlens{1}_median=0.044466,brlenIlnBrlens{1}_95%HPD={0.000000,0.167747},rateIlnBrlens{1}_mean=0.873140,rateIlnBrlens{1}_median=0.661036,rateIlnBrlens{1}_95%HPD={0.013410,2.320498},brlenIlnBrlens{2}_mean=0.104487,brlenIlnBrlens{2}_median=0.067822,brlenIlnBrlens{2}_95%HPD={0.000000,0.317555},rateIlnBrlens{2}_mean=1.327108,rateIlnBrlens{2}_median=1.004358,rateIlnBrlens{2}_95%HPD={0.009800,3.478803},brlenIlnBrlens{3}_mean=0.040421,brlenIlnBrlens{3}_median=0.020909,brlenIlnBrlens{3}_95%HPD={0.000000,0.144299},rateIlnBrlens{3}_mean=0.572146,rateIlnBrlens{3}_median=0.327390,rateIlnBrlens{3}_95%HPD={0.002374,1.898750},brlenIlnBrlens{4,5}_mean=0.057974,brlenIlnBrlens{4,5}_median=0.052181,brlenIlnBrlens{4,5}_95%HPD={0.000012,0.124767},rateIlnBrlens{4,5}_mean=0.881532,rateIlnBrlens{4,5}_median=0.759905,rateIlnBrlens{4,5}_95%HPD={0.105333,1.938798}],(16[&prob=1.00000000e+00,prob_stddev=0.00000000e+00,prob_range={1.00000000e+00,1.00000000e+00},prob(percent)="100",prob+-sd="100+-0",height_mean=6.95714678e-08,height_median=4.99999997e-08,height_95%HPD={0.00000000e+00,2.06000000e-07},age_mean=2.01312962e-05,age_median=1.46229853e-05,age_95%HPD={0.00000000e+00,6.21861853e-05}]:4.514836e+00[&length_mean=2.10938842e-02,length_median=1.49024900e-02,length_95%HPD={1.86385200e-04,5.99683900e-02},brlenIlnBrlens{1}_mean=0.013705,brlenIlnBrlens{1}_median=0.008346,brlenIlnBrlens{1}_95%HPD={0.000002,0.045230},rateIlnBrlens{1}_mean=0.829806,rateIlnBrlens{1}_median=0.570580,rateIlnBrlens{1}_95%HPD={0.002888,2.435184},brlenIlnBrlens{2}_mean=0.016759,brlenIlnBrlens{2}_median=0.010231,brlenIlnBrlens{2}_95%HPD={0.000015,0.053534},rateIlnBrlens{2}_mean=0.860868,rateIlnBrlens{2}_median=0.767471,rateIlnBrlens{2}_95%HPD={0.025669,1.959567},brlenIlnBrlens{3}_mean=0.013210,brlenIlnBrlens{3}_median=0.005549,brlenIlnBrlens{3}_95%HPD={0.000003,0.050667},rateIlnBrlens{3}_mean=0.722446,rateIlnBrlens{3}_median=0.381236,rateIlnBrlens{3}_95%HPD={0.002197,2.465582},brlenIlnBrlens{4,5}_mean=0.021528,brlenIlnBrlens{4,5}_median=0.011597,brlenIlnBrlens{4,5}_95%HPD={0.000012,0.071922},rateIlnBrlens{4,5}_mean=1.015724,rateIlnBrlens{4,5}_median=0.804700,rateIlnBrlens{4,5}_95%HPD={0.051955,2.450599}],17[&prob=1.00000000e+00,prob_stddev=0.00000000e+00,prob_range={1.00000000e+00,1.00000000e+00},prob(percent)="100",prob+-sd="100+-0",height_mean=6.95708349e-08,height_median=4.99999997e-08,height_95%HPD={0.00000000e+00,2.06000000e-07},age_mean=2.01312719e-05,age_median=1.46229853e-05,age_95%HPD={0.00000000e+00,6.21861853e-05}]:4.514836e+00[&length_mean=2.10928644e-02,length_median=1.49024900e-02,length_95%HPD={1.86385200e-04,5.99683900e-02},brlenIlnBrlens{1}_mean=0.015293,brlenIlnBrlens{1}_median=0.009308,brlenIlnBrlens{1}_95%HPD={0.000011,0.049869},rateIlnBrlens{1}_mean=0.941479,rateIlnBrlens{1}_median=0.631723,rateIlnBrlens{1}_95%HPD={0.017664,2.727172},brlenIlnBrlens{2}_mean=0.016927,brlenIlnBrlens{2}_median=0.010307,brlenIlnBrlens{2}_95%HPD={0.000011,0.054093},rateIlnBrlens{2}_mean=0.862195,rateIlnBrlens{2}_median=0.770415,rateIlnBrlens{2}_95%HPD={0.021028,1.950822},brlenIlnBrlens{3}_mean=0.013088,brlenIlnBrlens{3}_median=0.005443,brlenIlnBrlens{3}_95%HPD={0.000003,0.049162},rateIlnBrlens{3}_mean=0.720561,rateIlnBrlens{3}_median=0.376914,rateIlnBrlens{3}_95%HPD={0.002701,2.529266},brlenIlnBrlens{4,5}_mean=0.019505,brlenIlnBrlens{4,5}_median=0.011582,brlenIlnBrlens{4,5}_95%HPD={0.000041,0.065389},rateIlnBrlens{4,5}_mean=0.983228,rateIlnBrlens{4,5}_median=0.793873,rateIlnBrlens{4,5}_95%HPD={0.039775,2.337172}])[&prob=9.99822225e-01,prob_stddev=2.26270147e-04,prob_range={9.99662228e-01,9.99982223e-01},prob(percent)="100",prob+-sd="100+-0",height_mean=2.10555794e-02,height_median=1.49003300e-02,height_95%HPD={9.64320000e-05,5.98261800e-02},age_mean=6.37015487e+00,age_median=4.51485014e+00,age_95%HPD={3.39574786e-02,1.82819325e+01}]:7.212795e+01[&length_mean=2.39394050e-01,length_median=2.32456800e-01,length_95%HPD={1.11861100e-01,3.79345600e-01},brlenIlnBrlens{1}_mean=0.088010,brlenIlnBrlens{1}_median=0.069152,brlenIlnBrlens{1}_95%HPD={0.002296,0.224997},rateIlnBrlens{1}_mean=0.384288,rateIlnBrlens{1}_median=0.300824,rateIlnBrlens{1}_95%HPD={0.010958,0.970133},brlenIlnBrlens{2}_mean=0.257778,brlenIlnBrlens{2}_median=0.212117,brlenIlnBrlens{2}_95%HPD={0.009920,0.613003},rateIlnBrlens{2}_mean=1.096957,rateIlnBrlens{2}_median=0.935571,rateIlnBrlens{2}_95%HPD={0.050344,2.555424},brlenIlnBrlens{3}_mean=0.085711,brlenIlnBrlens{3}_median=0.053152,brlenIlnBrlens{3}_95%HPD={0.000471,0.269587},rateIlnBrlens{3}_mean=0.368661,rateIlnBrlens{3}_median=0.230278,rateIlnBrlens{3}_95%HPD={0.003937,1.134876},brlenIlnBrlens{4,5}_mean=0.148844,brlenIlnBrlens{4,5}_median=0.145609,brlenIlnBrlens{4,5}_95%HPD={0.042581,0.255047},rateIlnBrlens{4,5}_mean=0.670420,rateIlnBrlens{4,5}_median=0.615058,rateIlnBrlens{4,5}_95%HPD={0.148317,1.296172}])[&prob=8.64251302e-01,prob_stddev=9.77989859e-03,prob_range={8.57335870e-01,8.71166735e-01},prob(percent)="86",prob+-sd="86+-1",height_mean=2.59325519e-01,height_median=2.51530830e-01,height_95%HPD={1.44463460e-01,3.88514761e-01},age_mean=7.50855491e+01,age_median=7.66428036e+01,age_95%HPD={4.64197110e+01,9.49994459e+01}]:1.835722e+01[&length_mean=7.95680482e-02,length_median=6.43135000e-02,length_95%HPD={1.31501600e-06,2.06019400e-01},brlenIlnBrlens{1}_mean=0.047047,brlenIlnBrlens{1}_median=0.027614,brlenIlnBrlens{1}_95%HPD={0.000000,0.157177},rateIlnBrlens{1}_mean=0.670848,rateIlnBrlens{1}_median=0.479431,rateIlnBrlens{1}_95%HPD={0.010806,1.899360},brlenIlnBrlens{2}_mean=0.083776,brlenIlnBrlens{2}_median=0.051020,brlenIlnBrlens{2}_95%HPD={0.000001,0.265762},rateIlnBrlens{2}_mean=1.058632,rateIlnBrlens{2}_median=0.877042,rateIlnBrlens{2}_95%HPD={0.029276,2.484270},brlenIlnBrlens{3}_mean=0.038566,brlenIlnBrlens{3}_median=0.018480,brlenIlnBrlens{3}_95%HPD={0.000000,0.141183},rateIlnBrlens{3}_mean=0.586638,rateIlnBrlens{3}_median=0.323167,rateIlnBrlens{3}_95%HPD={0.003405,1.945091},brlenIlnBrlens{4,5}_mean=0.064794,brlenIlnBrlens{4,5}_median=0.042848,brlenIlnBrlens{4,5}_95%HPD={0.000001,0.199694},rateIlnBrlens{4,5}_mean=0.881992,rateIlnBrlens{4,5}_median=0.742364,rateIlnBrlens{4,5}_95%HPD={0.053497,2.042074}])[&prob=1.00000000e+00,prob_stddev=0.00000000e+00,prob_range={1.00000000e+00,1.00000000e+00},prob(percent)="100",prob+-sd="100+-0",height_mean=3.35883502e-01,height_median=3.22022030e-01,height_95%HPD={1.97145799e-01,5.06491520e-01},age_mean=9.85509975e+01,age_median=9.50000221e+01,age_95%HPD={9.49999697e+01,1.19168785e+02}]:2.150000e+02[&length_mean=7.44293332e-01,length_median=7.13792900e-01,length_95%HPD={3.71517200e-01,1.16741600e+00},brlenIlnBrlens{1}_mean=4.107029,brlenIlnBrlens{1}_median=2.573029,brlenIlnBrlens{1}_95%HPD={0.170046,12.215980},rateIlnBrlens{1}_mean=5.537906,rateIlnBrlens{1}_median=3.590016,rateIlnBrlens{1}_95%HPD={0.302038,16.216923},brlenIlnBrlens{2}_mean=0.657662,brlenIlnBrlens{2}_median=0.553112,brlenIlnBrlens{2}_95%HPD={0.039135,1.518277},rateIlnBrlens{2}_mean=0.885941,rateIlnBrlens{2}_median=0.792903,rateIlnBrlens{2}_95%HPD={0.065608,1.881587},brlenIlnBrlens{3}_mean=0.428790,brlenIlnBrlens{3}_median=0.260496,brlenIlnBrlens{3}_95%HPD={0.000659,1.352514},rateIlnBrlens{3}_mean=0.579150,rateIlnBrlens{3}_median=0.361458,rateIlnBrlens{3}_95%HPD={0.004315,1.795863},brlenIlnBrlens{4,5}_mean=0.391514,brlenIlnBrlens{4,5}_median=0.353601,brlenIlnBrlens{4,5}_95%HPD={0.050349,0.809205},rateIlnBrlens{4,5}_mean=0.553879,rateIlnBrlens{4,5}_median=0.495991,rateIlnBrlens{4,5}_95%HPD={0.058829,1.163264}])[&prob=9.38943308e-01,prob_stddev=1.24448581e-03,prob_range={9.38063323e-01,9.39823292e-01},prob(percent)="94",prob+-sd="94+-0",height_mean=1.07125514e+00,height_median=1.02978418e+00,height_95%HPD={5.95615990e-01,1.61630833e+00},age_mean=3.12884817e+02,age_median=3.10000024e+02,age_95%HPD={3.09999884e+02,3.29981693e+02}]:1.019465e+02[&length_mean=4.04725742e-01,length_median=3.71122300e-01,length_95%HPD={6.30488300e-02,8.31573600e-01},brlenIlnBrlens{1}_mean=3.190556,brlenIlnBrlens{1}_median=1.481186,brlenIlnBrlens{1}_95%HPD={0.000170,11.533750},rateIlnBrlens{1}_mean=7.630884,rateIlnBrlens{1}_median=4.033889,rateIlnBrlens{1}_95%HPD={0.033414,25.433095},brlenIlnBrlens{2}_mean=0.493849,brlenIlnBrlens{2}_median=0.365113,brlenIlnBrlens{2}_95%HPD={0.004951,1.310876},rateIlnBrlens{2}_mean=1.312040,rateIlnBrlens{2}_median=1.013041,rateIlnBrlens{2}_95%HPD={0.033762,3.292873},brlenIlnBrlens{3}_mean=0.327565,brlenIlnBrlens{3}_median=0.162250,brlenIlnBrlens{3}_95%HPD={0.000033,1.142665},rateIlnBrlens{3}_mean=0.859586,rateIlnBrlens{3}_median=0.454605,rateIlnBrlens{3}_95%HPD={0.004257,2.882344},brlenIlnBrlens{4,5}_mean=0.251312,brlenIlnBrlens{4,5}_median=0.212334,brlenIlnBrlens{4,5}_95%HPD={0.009085,0.589549},rateIlnBrlens{4,5}_mean=0.685121,rateIlnBrlens{4,5}_median=0.597532,rateIlnBrlens{4,5}_95%HPD={0.049119,1.491112}],(19[&prob=1.00000000e+00,prob_stddev=0.00000000e+00,prob_range={1.00000000e+00,1.00000000e+00},prob(percent)="100",prob+-sd="100+-0",height_mean=1.06230410e+00,height_median=1.02184452e+00,height_95%HPD={6.03967809e-01,1.61023293e+00},age_mean=3.10000020e+02,age_median=3.10000019e+02,age_95%HPD={3.09999950e+02,3.10000092e+02}]:5.703979e+01[&length_mean=3.13996017e-01,length_median=2.81387200e-01,length_95%HPD={2.98409900e-02,6.75308000e-01},brlenIlnBrlens{1}_mean=0.447258,brlenIlnBrlens{1}_median=0.314609,brlenIlnBrlens{1}_95%HPD={0.000000,1.278400},rateIlnBrlens{1}_mean=1.575000,rateIlnBrlens{1}_median=1.133803,rateIlnBrlens{1}_95%HPD={0.027426,4.318033},brlenIlnBrlens{2}_mean=0.358234,brlenIlnBrlens{2}_median=0.250429,brlenIlnBrlens{2}_95%HPD={0.000000,1.011518},rateIlnBrlens{2}_mean=1.153474,rateIlnBrlens{2}_median=0.923801,rateIlnBrlens{2}_95%HPD={0.024789,2.831025},brlenIlnBrlens{3}_mean=0.259795,brlenIlnBrlens{3}_median=0.148108,brlenIlnBrlens{3}_95%HPD={0.000000,0.869984},rateIlnBrlens{3}_mean=0.957896,rateIlnBrlens{3}_median=0.555645,rateIlnBrlens{3}_95%HPD={0.004420,3.140949},brlenIlnBrlens{4,5}_mean=0.310415,brlenIlnBrlens{4,5}_median=0.212629,brlenIlnBrlens{4,5}_95%HPD={0.000000,0.890369},rateIlnBrlens{4,5}_mean=0.986976,rateIlnBrlens{4,5}_median=0.800453,rateIlnBrlens{4,5}_95%HPD={0.050499,2.352413}],20[&prob=1.00000000e+00,prob_stddev=0.00000000e+00,prob_range={1.00000000e+00,1.00000000e+00},prob(percent)="100",prob+-sd="100+-0",height_mean=1.06230410e+00,height_median=1.02184452e+00,height_95%HPD={6.03967809e-01,1.61023293e+00},age_mean=3.10000020e+02,age_median=3.10000019e+02,age_95%HPD={3.09999952e+02,3.10000090e+02}]:5.703979e+01[&length_mean=3.05862706e-01,length_median=2.86369200e-01,length_95%HPD={0.00000000e+00,6.21570800e-01},brlenIlnBrlens{1}_mean=0.231807,brlenIlnBrlens{1}_median=0.160869,brlenIlnBrlens{1}_95%HPD={0.000000,0.671081},rateIlnBrlens{1}_mean=0.840866,rateIlnBrlens{1}_median=0.610968,rateIlnBrlens{1}_95%HPD={0.009958,2.272250},brlenIlnBrlens{2}_mean=0.264776,brlenIlnBrlens{2}_median=0.196546,brlenIlnBrlens{2}_95%HPD={0.000000,0.714539},rateIlnBrlens{2}_mean=0.892404,rateIlnBrlens{2}_median=0.798500,rateIlnBrlens{2}_95%HPD={0.019721,1.990443},brlenIlnBrlens{3}_mean=0.170765,brlenIlnBrlens{3}_median=0.083375,brlenIlnBrlens{3}_95%HPD={0.000000,0.574104},rateIlnBrlens{3}_mean=0.668227,rateIlnBrlens{3}_median=0.329865,rateIlnBrlens{3}_95%HPD={0.003367,2.026252},brlenIlnBrlens{4,5}_mean=0.303641,brlenIlnBrlens{4,5}_median=0.209871,brlenIlnBrlens{4,5}_95%HPD={0.000000,0.879215},rateIlnBrlens{4,5}_mean=0.991229,rateIlnBrlens{4,5}_median=0.806390,rateIlnBrlens{4,5}_95%HPD={0.062901,2.379159}])[&prob=3.33976285e-01,prob_stddev=4.94023155e-03,prob_range={3.30483014e-01,3.37469556e-01},prob(percent)="33",prob+-sd="33+-0",height_mean=1.27225665e+00,height_median=1.22160188e+00,height_95%HPD={6.95220940e-01,1.92220024e+00},age_mean=3.72812638e+02,age_median=3.67039804e+02,age_95%HPD={3.18899349e+02,4.39672507e+02}]:4.490670e+01[&length_mean=2.35602617e-01,length_median=2.11300500e-01,length_95%HPD={8.29364200e-06,5.26628400e-01},brlenIlnBrlens{1}_mean=0.193259,brlenIlnBrlens{1}_median=0.116643,brlenIlnBrlens{1}_95%HPD={0.000001,0.632809},rateIlnBrlens{1}_mean=0.861187,rateIlnBrlens{1}_median=0.599394,rateIlnBrlens{1}_95%HPD={0.004568,2.408076},brlenIlnBrlens{2}_mean=0.262999,brlenIlnBrlens{2}_median=0.175047,brlenIlnBrlens{2}_95%HPD={0.000004,0.781797},rateIlnBrlens{2}_mean=1.120865,rateIlnBrlens{2}_median=0.906396,rateIlnBrlens{2}_95%HPD={0.028681,2.729371},brlenIlnBrlens{3}_mean=0.139382,brlenIlnBrlens{3}_median=0.067205,brlenIlnBrlens{3}_95%HPD={0.000001,0.498644},rateIlnBrlens{3}_mean=0.672845,rateIlnBrlens{3}_median=0.354577,rateIlnBrlens{3}_95%HPD={0.001354,2.261323},brlenIlnBrlens{4,5}_mean=0.226724,brlenIlnBrlens{4,5}_median=0.149900,brlenIlnBrlens{4,5}_95%HPD={0.000008,0.686267},rateIlnBrlens{4,5}_mean=0.967246,rateIlnBrlens{4,5}_median=0.781481,rateIlnBrlens{4,5}_95%HPD={0.058275,2.313451}])[&prob=2.28547048e-01,prob_stddev=7.34120923e-03,prob_range={2.23356029e-01,2.33738067e-01},prob(percent)="23",prob+-sd="23+-1",height_mean=1.42987797e+00,height_median=1.37642712e+00,height_95%HPD={8.22260732e-01,2.14709315e+00},age_mean=4.15893010e+02,age_median=4.11946503e+02,age_95%HPD={3.45605780e+02,4.89194571e+02}]:4.502787e+01[&length_mean=1.68797759e-01,length_median=1.38116500e-01,length_95%HPD={1.97418400e-05,4.32366300e-01},brlenIlnBrlens{1}_mean=0.245149,brlenIlnBrlens{1}_median=0.085797,brlenIlnBrlens{1}_95%HPD={0.000014,0.893803},rateIlnBrlens{1}_mean=1.248532,rateIlnBrlens{1}_median=0.720128,rateIlnBrlens{1}_95%HPD={0.009717,3.836819},brlenIlnBrlens{2}_mean=0.166436,brlenIlnBrlens{2}_median=0.105191,brlenIlnBrlens{2}_95%HPD={0.000014,0.515076},rateIlnBrlens{2}_mean=1.011603,rateIlnBrlens{2}_median=0.856417,rateIlnBrlens{2}_95%HPD={0.030513,2.330428},brlenIlnBrlens{3}_mean=0.134268,brlenIlnBrlens{3}_median=0.048217,brlenIlnBrlens{3}_95%HPD={0.000005,0.525742},rateIlnBrlens{3}_mean=0.898484,rateIlnBrlens{3}_median=0.390505,rateIlnBrlens{3}_95%HPD={0.001699,3.137876},brlenIlnBrlens{4,5}_mean=0.121194,brlenIlnBrlens{4,5}_median=0.088912,brlenIlnBrlens{4,5}_95%HPD={0.000009,0.342885},rateIlnBrlens{4,5}_mean=0.816728,rateIlnBrlens{4,5}_median=0.693420,rateIlnBrlens{4,5}_95%HPD={0.066038,1.848140}],(((((((((((((21[&prob=1.00000000e+00,prob_stddev=0.00000000e+00,prob_range={1.00000000e+00,1.00000000e+00},prob(percent)="100",prob+-sd="100+-0",height_mean=6.95388846e-08,height_median=4.60000003e-08,height_95%HPD={0.00000000e+00,2.03760000e-07},age_mean=2.01355838e-05,age_median=1.41266394e-05,age_95%HPD={0.00000000e+00,6.05872343e-05}]:5.613347e+00[&length_mean=2.47793921e-02,length_median=2.36343100e-02,length_95%HPD={6.44165800e-03,4.43693400e-02},brlenIlnBrlens{1}_mean=0.015423,brlenIlnBrlens{1}_median=0.011663,brlenIlnBrlens{1}_95%HPD={0.000130,0.041470},rateIlnBrlens{1}_mean=0.659116,rateIlnBrlens{1}_median=0.505859,rateIlnBrlens{1}_95%HPD={0.024643,1.728687},brlenIlnBrlens{2}_mean=0.020980,brlenIlnBrlens{2}_median=0.016769,brlenIlnBrlens{2}_95%HPD={0.000349,0.052674},rateIlnBrlens{2}_mean=0.861733,rateIlnBrlens{2}_median=0.759775,rateIlnBrlens{2}_95%HPD={0.024356,1.917687},brlenIlnBrlens{3}_mean=0.005908,brlenIlnBrlens{3}_median=0.003988,brlenIlnBrlens{3}_95%HPD={0.000041,0.017607},rateIlnBrlens{3}_mean=0.258468,rateIlnBrlens{3}_median=0.174680,rateIlnBrlens{3}_95%HPD={0.002305,0.766990},brlenIlnBrlens{4,5}_mean=0.024458,brlenIlnBrlens{4,5}_median=0.024539,brlenIlnBrlens{4,5}_95%HPD={0.003903,0.042005},rateIlnBrlens{4,5}_mean=1.084059,rateIlnBrlens{4,5}_median=0.995321,rateIlnBrlens{4,5}_95%HPD={0.246293,2.194936}],(36[&prob=1.00000000e+00,prob_stddev=0.00000000e+00,prob_range={1.00000000e+00,1.00000000e+00},prob(percent)="100",prob+-sd="100+-0",height_mean=6.95326519e-08,height_median=4.60000000e-08,height_95%HPD={0.00000000e+00,2.03700000e-07},age_mean=2.01322484e-05,age_median=1.41011971e-05,age_95%HPD={0.00000000e+00,6.04880012e-05}]:2.167679e+00[&length_mean=9.93149766e-03,length_median=7.31306900e-03,length_95%HPD={2.46209200e-04,2.64125300e-02},brlenIlnBrlens{1}_mean=0.006252,brlenIlnBrlens{1}_median=0.003835,brlenIlnBrlens{1}_95%HPD={0.000006,0.019971},rateIlnBrlens{1}_mean=0.780743,rateIlnBrlens{1}_median=0.522796,rateIlnBrlens{1}_95%HPD={0.004839,2.350280},brlenIlnBrlens{2}_mean=0.008929,brlenIlnBrlens{2}_median=0.005413,brlenIlnBrlens{2}_95%HPD={0.000026,0.027989},rateIlnBrlens{2}_mean=0.930948,rateIlnBrlens{2}_median=0.809802,rateIlnBrlens{2}_95%HPD={0.025010,2.144245},brlenIlnBrlens{3}_mean=0.005382,brlenIlnBrlens{3}_median=0.003703,brlenIlnBrlens{3}_95%HPD={0.000015,0.016057},rateIlnBrlens{3}_mean=0.784432,rateIlnBrlens{3}_median=0.498324,rateIlnBrlens{3}_95%HPD={0.004903,2.352869},brlenIlnBrlens{4,5}_mean=0.009873,brlenIlnBrlens{4,5}_median=0.005613,brlenIlnBrlens{4,5}_95%HPD={0.000028,0.032071},rateIlnBrlens{4,5}_mean=0.985981,rateIlnBrlens{4,5}_median=0.799073,rateIlnBrlens{4,5}_95%HPD={0.041901,2.357826}],37[&prob=1.00000000e+00,prob_stddev=0.00000000e+00,prob_range={1.00000000e+00,1.00000000e+00},prob(percent)="100",prob+-sd="100+-0",height_mean=6.95380641e-08,height_median=4.60000000e-08,height_95%HPD={0.00000000e+00,2.03700000e-07},age_mean=2.01336168e-05,age_median=1.41033362e-05,age_95%HPD={0.00000000e+00,6.04908577e-05}]:2.167679e+00[&length_mean=9.95332816e-03,length_median=7.31172300e-03,length_95%HPD={2.46209200e-04,2.64146700e-02},brlenIlnBrlens{1}_mean=0.010023,brlenIlnBrlens{1}_median=0.006304,brlenIlnBrlens{1}_95%HPD={0.000012,0.031324},rateIlnBrlens{1}_mean=1.224869,rateIlnBrlens{1}_median=0.838471,rateIlnBrlens{1}_95%HPD={0.007135,3.562717},brlenIlnBrlens{2}_mean=0.008973,brlenIlnBrlens{2}_median=0.005385,brlenIlnBrlens{2}_95%HPD={0.000006,0.028183},rateIlnBrlens{2}_mean=0.937468,rateIlnBrlens{2}_median=0.813732,rateIlnBrlens{2}_95%HPD={0.024442,2.168460},brlenIlnBrlens{3}_mean=0.003127,brlenIlnBrlens{3}_median=0.001843,brlenIlnBrlens{3}_95%HPD={0.000007,0.010364},rateIlnBrlens{3}_mean=0.421913,rateIlnBrlens{3}_median=0.251071,rateIlnBrlens{3}_95%HPD={0.001260,1.361166},brlenIlnBrlens{4,5}_mean=0.009850,brlenIlnBrlens{4,5}_median=0.005683,brlenIlnBrlens{4,5}_95%HPD={0.000045,0.031757},rateIlnBrlens{4,5}_mean=0.998975,rateIlnBrlens{4,5}_median=0.802765,rateIlnBrlens{4,5}_95%HPD={0.049497,2.379613}])[&prob=9.87484667e-01,prob_stddev=1.28219750e-03,prob_range={9.86578016e-01,9.88391317e-01},prob(percent)="99",prob+-sd="99+-0",height_mean=9.39742374e-03,height_median=7.21932500e-03,height_95%HPD={2.46324000e-04,2.42156900e-02},age_mean=2.83789258e+00,age_median=2.16769278e+00,age_95%HPD={7.99905445e-02,7.35594226e+00}]:3.445669e+00[&length_mean=1.90203495e-02,length_median=1.50753700e-02,length_95%HPD={8.63641500e-04,4.85348700e-02},brlenIlnBrlens{1}_mean=0.008912,brlenIlnBrlens{1}_median=0.005699,brlenIlnBrlens{1}_95%HPD={0.000045,0.027807},rateIlnBrlens{1}_mean=0.539379,rateIlnBrlens{1}_median=0.384762,rateIlnBrlens{1}_95%HPD={0.007805,1.491104},brlenIlnBrlens{2}_mean=0.016277,brlenIlnBrlens{2}_median=0.010991,brlenIlnBrlens{2}_95%HPD={0.000092,0.048336},rateIlnBrlens{2}_mean=0.893540,rateIlnBrlens{2}_median=0.785134,rateIlnBrlens{2}_95%HPD={0.025951,2.026119},brlenIlnBrlens{3}_mean=0.026829,brlenIlnBrlens{3}_median=0.022561,brlenIlnBrlens{3}_95%HPD={0.000004,0.062964},rateIlnBrlens{3}_mean=2.188724,rateIlnBrlens{3}_median=1.574117,rateIlnBrlens{3}_95%HPD={0.009310,6.063966},brlenIlnBrlens{4,5}_mean=0.019043,brlenIlnBrlens{4,5}_median=0.011739,brlenIlnBrlens{4,5}_95%HPD={0.000059,0.058377},rateIlnBrlens{4,5}_mean=1.002869,rateIlnBrlens{4,5}_median=0.806500,rateIlnBrlens{4,5}_95%HPD={0.045285,2.420159}])[&prob=4.06952765e-01,prob_stddev=1.99871964e-03,prob_range={4.05539457e-01,4.08366073e-01},prob(percent)="41",prob+-sd="41+-0",height_mean=1.98060747e-02,height_median=1.85693340e-02,height_95%HPD={5.47090000e-03,3.68432600e-02},age_mean=6.03153829e+00,age_median=5.61336139e+00,age_95%HPD={1.37740503e+00,1.13082750e+01}]:3.151216e+00[&length_mean=9.84817248e-03,length_median=8.26124700e-03,length_95%HPD={8.50492600e-07,2.47007900e-02},brlenIlnBrlens{1}_mean=0.007330,brlenIlnBrlens{1}_median=0.004267,brlenIlnBrlens{1}_95%HPD={0.000000,0.024426},rateIlnBrlens{1}_mean=0.836688,rateIlnBrlens{1}_median=0.573665,rateIlnBrlens{1}_95%HPD={0.003792,2.433254},brlenIlnBrlens{2}_mean=0.008837,brlenIlnBrlens{2}_median=0.005728,brlenIlnBrlens{2}_95%HPD={0.000000,0.027263},rateIlnBrlens{2}_mean=0.935340,rateIlnBrlens{2}_median=0.817604,rateIlnBrlens{2}_95%HPD={0.022414,2.190741},brlenIlnBrlens{3}_mean=0.004770,brlenIlnBrlens{3}_median=0.002567,brlenIlnBrlens{3}_95%HPD={0.000000,0.016654},rateIlnBrlens{3}_mean=0.609537,rateIlnBrlens{3}_median=0.340177,rateIlnBrlens{3}_95%HPD={0.002258,2.009619},brlenIlnBrlens{4,5}_mean=0.009382,brlenIlnBrlens{4,5}_median=0.007289,brlenIlnBrlens{4,5}_95%HPD={0.000001,0.025344},rateIlnBrlens{4,5}_mean=1.056035,rateIlnBrlens{4,5}_median=0.877537,rateIlnBrlens{4,5}_95%HPD={0.092965,2.471682}],(23[&prob=1.00000000e+00,prob_stddev=0.00000000e+00,prob_range={1.00000000e+00,1.00000000e+00},prob(percent)="100",prob+-sd="100+-0",height_mean=6.95531697e-08,height_median=4.60000000e-08,height_95%HPD={0.00000000e+00,2.04000000e-07},age_mean=2.01403684e-05,age_median=1.41287454e-05,age_95%HPD={0.00000000e+00,6.05186477e-05}]:2.213884e+00[&length_mean=8.81959690e-03,length_median=7.36895100e-03,length_95%HPD={3.71735600e-04,2.08782500e-02},brlenIlnBrlens{1}_mean=0.007985,brlenIlnBrlens{1}_median=0.005689,brlenIlnBrlens{1}_95%HPD={0.000041,0.022759},rateIlnBrlens{1}_mean=1.091057,rateIlnBrlens{1}_median=0.791314,rateIlnBrlens{1}_95%HPD={0.018188,3.010448},brlenIlnBrlens{2}_mean=0.008029,brlenIlnBrlens{2}_median=0.005360,brlenIlnBrlens{2}_95%HPD={0.000014,0.023707},rateIlnBrlens{2}_mean=0.932024,rateIlnBrlens{2}_median=0.812001,rateIlnBrlens{2}_95%HPD={0.016473,2.142276},brlenIlnBrlens{3}_mean=0.002943,brlenIlnBrlens{3}_median=0.001796,brlenIlnBrlens{3}_95%HPD={0.000002,0.009480},rateIlnBrlens{3}_mean=0.410461,rateIlnBrlens{3}_median=0.249743,rateIlnBrlens{3}_95%HPD={0.004475,1.307901},brlenIlnBrlens{4,5}_mean=0.008083,brlenIlnBrlens{4,5}_median=0.006201,brlenIlnBrlens{4,5}_95%HPD={0.000090,0.021467},rateIlnBrlens{4,5}_mean=0.990462,rateIlnBrlens{4,5}_median=0.846371,rateIlnBrlens{4,5}_95%HPD={0.079780,2.295890}],38[&prob=1.00000000e+00,prob_stddev=0.00000000e+00,prob_range={1.00000000e+00,1.00000000e+00},prob(percent)="100",prob+-sd="100+-0",height_mean=6.95529589e-08,height_median=4.60000000e-08,height_95%HPD={0.00000000e+00,2.04000000e-07},age_mean=2.01403069e-05,age_median=1.41285191e-05,age_95%HPD={0.00000000e+00,6.05186477e-05}]:2.213884e+00[&length_mean=8.82099510e-03,length_median=7.36893200e-03,length_95%HPD={3.59984500e-04,2.08281600e-02},brlenIlnBrlens{1}_mean=0.005477,brlenIlnBrlens{1}_median=0.003426,brlenIlnBrlens{1}_95%HPD={0.000005,0.017265},rateIlnBrlens{1}_mean=0.702979,rateIlnBrlens{1}_median=0.488891,rateIlnBrlens{1}_95%HPD={0.008713,2.031376},brlenIlnBrlens{2}_mean=0.008221,brlenIlnBrlens{2}_median=0.005467,brlenIlnBrlens{2}_95%HPD={0.000019,0.024449},rateIlnBrlens{2}_mean=0.953311,rateIlnBrlens{2}_median=0.821094,rateIlnBrlens{2}_95%HPD={0.030570,2.223215},brlenIlnBrlens{3}_mean=0.005270,brlenIlnBrlens{3}_median=0.003733,brlenIlnBrlens{3}_95%HPD={0.000014,0.015181},rateIlnBrlens{3}_mean=0.782109,rateIlnBrlens{3}_median=0.522301,rateIlnBrlens{3}_95%HPD={0.009301,2.348561},brlenIlnBrlens{4,5}_mean=0.008877,brlenIlnBrlens{4,5}_median=0.005670,brlenIlnBrlens{4,5}_95%HPD={0.000045,0.026877},rateIlnBrlens{4,5}_mean=1.012059,rateIlnBrlens{4,5}_median=0.811803,rateIlnBrlens{4,5}_95%HPD={0.041452,2.415120}])[&prob=9.96764502e-01,prob_stddev=1.03078623e-03,prob_range={9.96035626e-01,9.97493378e-01},prob(percent)="100",prob+-sd="100+-0",height_mean=8.75384250e-03,height_median=7.34837300e-03,height_95%HPD={4.70902000e-04,2.06957870e-02},age_mean=2.64955230e+00,age_median=2.21389788e+00,age_95%HPD={1.19345954e-01,6.28189879e+00}]:6.550680e+00[&length_mean=1.84856867e-02,length_median=1.70415200e-02,length_95%HPD={2.34776800e-03,3.67447600e-02},brlenIlnBrlens{1}_mean=0.008578,brlenIlnBrlens{1}_median=0.006082,brlenIlnBrlens{1}_95%HPD={0.000048,0.024501},rateIlnBrlens{1}_mean=0.509699,rateIlnBrlens{1}_median=0.372881,rateIlnBrlens{1}_95%HPD={0.007234,1.360286},brlenIlnBrlens{2}_mean=0.016035,brlenIlnBrlens{2}_median=0.012149,brlenIlnBrlens{2}_95%HPD={0.000098,0.042568},rateIlnBrlens{2}_mean=0.887534,rateIlnBrlens{2}_median=0.782373,rateIlnBrlens{2}_95%HPD={0.012722,1.997316},brlenIlnBrlens{3}_mean=0.025214,brlenIlnBrlens{3}_median=0.021225,brlenIlnBrlens{3}_95%HPD={0.000173,0.057801},rateIlnBrlens{3}_mean=1.702670,rateIlnBrlens{3}_median=1.255838,rateIlnBrlens{3}_95%HPD={0.034115,4.534081},brlenIlnBrlens{4,5}_mean=0.015572,brlenIlnBrlens{4,5}_median=0.014823,brlenIlnBrlens{4,5}_95%HPD={0.000970,0.031024},rateIlnBrlens{4,5}_mean=0.939892,rateIlnBrlens{4,5}_median=0.832916,rateIlnBrlens{4,5}_95%HPD={0.124938,1.985730}])[&prob=8.35256262e-01,prob_stddev=8.07030192e-03,prob_range={8.29549697e-01,8.40962827e-01},prob(percent)="84",prob+-sd="84+-1",height_mean=3.05794018e-02,height_median=2.87689140e-02,height_95%HPD={1.30238400e-02,5.16403760e-02},age_mean=9.31667321e+00,age_median=8.76457771e+00,age_95%HPD={3.48937393e+00,1.62612340e+01}]:6.355132e+00[&length_mean=2.27221227e-02,length_median=1.84450100e-02,length_95%HPD={5.50640700e-04,5.61912700e-02},brlenIlnBrlens{1}_mean=0.012749,brlenIlnBrlens{1}_median=0.008857,brlenIlnBrlens{1}_95%HPD={0.000001,0.037562},rateIlnBrlens{1}_mean=0.681426,rateIlnBrlens{1}_median=0.489739,rateIlnBrlens{1}_95%HPD={0.012851,1.897492},brlenIlnBrlens{2}_mean=0.019338,brlenIlnBrlens{2}_median=0.013068,brlenIlnBrlens{2}_95%HPD={0.000004,0.057708},rateIlnBrlens{2}_mean=0.887764,rateIlnBrlens{2}_median=0.780224,rateIlnBrlens{2}_95%HPD={0.026834,2.032162},brlenIlnBrlens{3}_mean=0.024944,brlenIlnBrlens{3}_median=0.019636,brlenIlnBrlens{3}_95%HPD={0.000002,0.065266},rateIlnBrlens{3}_mean=1.641659,rateIlnBrlens{3}_median=1.064123,rateIlnBrlens{3}_95%HPD={0.007883,5.043580},brlenIlnBrlens{4,5}_mean=0.022277,brlenIlnBrlens{4,5}_median=0.016890,brlenIlnBrlens{4,5}_95%HPD={0.000021,0.058693},rateIlnBrlens{4,5}_mean=1.041445,rateIlnBrlens{4,5}_median=0.894367,rateIlnBrlens{4,5}_95%HPD={0.081253,2.287043}],(31[&prob=1.00000000e+00,prob_stddev=0.00000000e+00,prob_range={1.00000000e+00,1.00000000e+00},prob(percent)="100",prob+-sd="100+-0",height_mean=6.95758292e-08,height_median=4.63900001e-08,height_95%HPD={0.00000000e+00,2.03499999e-07},age_mean=2.01453709e-05,age_median=1.41439367e-05,age_95%HPD={0.00000000e+00,6.05009260e-05}]:8.432346e+00[&length_mean=3.34587445e-02,length_median=3.05711400e-02,length_95%HPD={1.63585300e-03,7.10801400e-02},brlenIlnBrlens{1}_mean=0.013987,brlenIlnBrlens{1}_median=0.009719,brlenIlnBrlens{1}_95%HPD={0.000062,0.040827},rateIlnBrlens{1}_mean=0.504565,rateIlnBrlens{1}_median=0.356454,rateIlnBrlens{1}_95%HPD={0.007277,1.418728},brlenIlnBrlens{2}_mean=0.026881,brlenIlnBrlens{2}_median=0.019832,brlenIlnBrlens{2}_95%HPD={0.000181,0.073644},rateIlnBrlens{2}_mean=0.838366,rateIlnBrlens{2}_median=0.746173,rateIlnBrlens{2}_95%HPD={0.020494,1.862086},brlenIlnBrlens{3}_mean=0.014459,brlenIlnBrlens{3}_median=0.011243,brlenIlnBrlens{3}_95%HPD={0.000166,0.037688},rateIlnBrlens{3}_mean=0.716024,rateIlnBrlens{3}_median=0.395261,rateIlnBrlens{3}_95%HPD={0.006490,2.527556},brlenIlnBrlens{4,5}_mean=0.033312,brlenIlnBrlens{4,5}_median=0.022490,brlenIlnBrlens{4,5}_95%HPD={0.000174,0.097570},rateIlnBrlens{4,5}_mean=1.001565,rateIlnBrlens{4,5}_median=0.803043,rateIlnBrlens{4,5}_95%HPD={0.040643,2.416489}],(((32[&prob=1.00000000e+00,prob_stddev=0.00000000e+00,prob_range={1.00000000e+00,1.00000000e+00},prob(percent)="100",prob+-sd="100+-0",height_mean=6.95282581e-08,height_median=4.60000003e-08,height_95%HPD={0.00000000e+00,2.04000000e-07},age_mean=2.01294956e-05,age_median=1.41306932e-05,age_95%HPD={0.00000000e+00,6.04299869e-05}]:6.288491e-01[&length_mean=4.17531718e-03,length_median=2.44471800e-03,length_95%HPD={2.87686000e-08,1.39614900e-02},brlenIlnBrlens{1}_mean=0.002906,brlenIlnBrlens{1}_median=0.001245,brlenIlnBrlens{1}_95%HPD={0.000000,0.011135},rateIlnBrlens{1}_mean=0.829266,rateIlnBrlens{1}_median=0.546188,rateIlnBrlens{1}_95%HPD={0.001171,2.476572},brlenIlnBrlens{2}_mean=0.003936,brlenIlnBrlens{2}_median=0.001797,brlenIlnBrlens{2}_95%HPD={0.000000,0.014507},rateIlnBrlens{2}_mean=0.973328,rateIlnBrlens{2}_median=0.823729,rateIlnBrlens{2}_95%HPD={0.026560,2.290778},brlenIlnBrlens{3}_mean=0.001559,brlenIlnBrlens{3}_median=0.000708,brlenIlnBrlens{3}_95%HPD={0.000000,0.005909},rateIlnBrlens{3}_mean=0.562795,rateIlnBrlens{3}_median=0.299936,rateIlnBrlens{3}_95%HPD={0.001977,1.918248},brlenIlnBrlens{4,5}_mean=0.004146,brlenIlnBrlens{4,5}_median=0.001838,brlenIlnBrlens{4,5}_95%HPD={0.000000,0.015185},rateIlnBrlens{4,5}_mean=0.998477,rateIlnBrlens{4,5}_median=0.798955,rateIlnBrlens{4,5}_95%HPD={0.042273,2.380806}],35[&prob=1.00000000e+00,prob_stddev=0.00000000e+00,prob_range={1.00000000e+00,1.00000000e+00},prob(percent)="100",prob+-sd="100+-0",height_mean=6.95304023e-08,height_median=4.60000003e-08,height_95%HPD={0.00000000e+00,2.04000000e-07},age_mean=2.01300905e-05,age_median=1.41349650e-05,age_95%HPD={0.00000000e+00,6.04353858e-05}]:6.288491e-01[&length_mean=4.42957449e-03,length_median=2.51890500e-03,length_95%HPD={2.87686000e-08,1.50605400e-02},brlenIlnBrlens{1}_mean=0.002914,brlenIlnBrlens{1}_median=0.001272,brlenIlnBrlens{1}_95%HPD={0.000000,0.011211},rateIlnBrlens{1}_mean=0.805620,rateIlnBrlens{1}_median=0.543847,rateIlnBrlens{1}_95%HPD={0.007081,2.342021},brlenIlnBrlens{2}_mean=0.004119,brlenIlnBrlens{2}_median=0.001816,brlenIlnBrlens{2}_95%HPD={0.000000,0.015451},rateIlnBrlens{2}_mean=0.961630,rateIlnBrlens{2}_median=0.817462,rateIlnBrlens{2}_95%HPD={0.016932,2.266886},brlenIlnBrlens{3}_mean=0.001580,brlenIlnBrlens{3}_median=0.000727,brlenIlnBrlens{3}_95%HPD={0.000000,0.005945},rateIlnBrlens{3}_mean=0.546604,rateIlnBrlens{3}_median=0.301412,rateIlnBrlens{3}_95%HPD={0.001037,1.829640},brlenIlnBrlens{4,5}_mean=0.004423,brlenIlnBrlens{4,5}_median=0.001905,brlenIlnBrlens{4,5}_95%HPD={0.000000,0.016559},rateIlnBrlens{4,5}_mean=0.999893,rateIlnBrlens{4,5}_median=0.810491,rateIlnBrlens{4,5}_95%HPD={0.044466,2.385063}])[&prob=8.78837710e-01,prob_stddev=1.57132047e-03,prob_range={8.77726618e-01,8.79948801e-01},prob(percent)="88",prob+-sd="88+-0",height_mean=3.42044989e-03,height_median=2.08287000e-03,height_95%HPD={4.80000000e-08,1.11988730e-02},age_mean=1.03271964e+00,age_median=6.28863227e-01,age_95%HPD={1.23142258e-05,3.38703305e+00}]:2.246089e+00[&length_mean=1.16917286e-02,length_median=8.64483400e-03,length_95%HPD={4.02758800e-06,3.23755300e-02},brlenIlnBrlens{1}_mean=0.007519,brlenIlnBrlens{1}_median=0.004567,brlenIlnBrlens{1}_95%HPD={0.000000,0.024572},rateIlnBrlens{1}_mean=0.774140,rateIlnBrlens{1}_median=0.542674,rateIlnBrlens{1}_95%HPD={0.007951,2.248345},brlenIlnBrlens{2}_mean=0.010551,brlenIlnBrlens{2}_median=0.006314,brlenIlnBrlens{2}_95%HPD={0.000000,0.033770},rateIlnBrlens{2}_mean=0.943462,rateIlnBrlens{2}_median=0.805351,rateIlnBrlens{2}_95%HPD={0.022593,2.182633},brlenIlnBrlens{3}_mean=0.004827,brlenIlnBrlens{3}_median=0.003178,brlenIlnBrlens{3}_95%HPD={0.000000,0.014884},rateIlnBrlens{3}_mean=0.618289,rateIlnBrlens{3}_median=0.372085,rateIlnBrlens{3}_95%HPD={0.001275,1.987284},brlenIlnBrlens{4,5}_mean=0.011603,brlenIlnBrlens{4,5}_median=0.006639,brlenIlnBrlens{4,5}_95%HPD={0.000000,0.038134},rateIlnBrlens{4,5}_mean=0.993839,rateIlnBrlens{4,5}_median=0.810736,rateIlnBrlens{4,5}_95%HPD={0.052561,2.376719}],34[&prob=1.00000000e+00,prob_stddev=0.00000000e+00,prob_range={1.00000000e+00,1.00000000e+00},prob(percent)="100",prob+-sd="100+-0",height_mean=6.95283429e-08,height_median=4.60000003e-08,height_95%HPD={0.00000000e+00,2.04000000e-07},age_mean=2.01298555e-05,age_median=1.41179927e-05,age_95%HPD={0.00000000e+00,6.04344259e-05}]:2.874938e+00[&length_mean=1.55316908e-02,length_median=1.22351400e-02,length_95%HPD={3.26419500e-04,3.95569900e-02},brlenIlnBrlens{1}_mean=0.014877,brlenIlnBrlens{1}_median=0.009909,brlenIlnBrlens{1}_95%HPD={0.000020,0.044904},rateIlnBrlens{1}_mean=1.134498,rateIlnBrlens{1}_median=0.809080,rateIlnBrlens{1}_95%HPD={0.013124,3.174391},brlenIlnBrlens{2}_mean=0.013526,brlenIlnBrlens{2}_median=0.008822,brlenIlnBrlens{2}_95%HPD={0.000038,0.041301},rateIlnBrlens{2}_mean=0.909891,rateIlnBrlens{2}_median=0.783029,rateIlnBrlens{2}_95%HPD={0.010098,2.113174},brlenIlnBrlens{3}_mean=0.003921,brlenIlnBrlens{3}_median=0.002579,brlenIlnBrlens{3}_95%HPD={0.000014,0.011950},rateIlnBrlens{3}_mean=0.367524,rateIlnBrlens{3}_median=0.208500,rateIlnBrlens{3}_95%HPD={0.002521,1.185018},brlenIlnBrlens{4,5}_mean=0.015458,brlenIlnBrlens{4,5}_median=0.009369,brlenIlnBrlens{4,5}_95%HPD={0.000038,0.048926},rateIlnBrlens{4,5}_mean=0.992499,rateIlnBrlens{4,5}_median=0.797552,rateIlnBrlens{4,5}_95%HPD={0.053508,2.392464}])[&prob=4.64062861e-01,prob_stddev=4.82709648e-03,prob_range={4.60649588e-01,4.67476134e-01},prob(percent)="46",prob+-sd="46+-0",height_mean=1.15147239e-02,height_median=9.48754660e-03,height_95%HPD={6.69533000e-04,2.73235080e-02},age_mean=3.49532964e+00,age_median=2.87495209e+00,age_95%HPD={2.60633263e-01,8.36057719e+00}]:2.767255e+00[&length_mean=1.22254108e-02,length_median=8.57881900e-03,length_95%HPD={2.28204100e-06,3.55501600e-02},brlenIlnBrlens{1}_mean=0.008927,brlenIlnBrlens{1}_median=0.005715,brlenIlnBrlens{1}_95%HPD={0.000000,0.027882},rateIlnBrlens{1}_mean=0.979304,rateIlnBrlens{1}_median=0.673289,rateIlnBrlens{1}_95%HPD={0.018102,2.801848},brlenIlnBrlens{2}_mean=0.010711,brlenIlnBrlens{2}_median=0.006249,brlenIlnBrlens{2}_95%HPD={0.000001,0.035258},rateIlnBrlens{2}_mean=0.928728,rateIlnBrlens{2}_median=0.809549,rateIlnBrlens{2}_95%HPD={0.022134,2.098189},brlenIlnBrlens{3}_mean=0.003553,brlenIlnBrlens{3}_median=0.002014,brlenIlnBrlens{3}_95%HPD={0.000000,0.012149},rateIlnBrlens{3}_mean=0.419493,rateIlnBrlens{3}_median=0.241747,rateIlnBrlens{3}_95%HPD={0.002005,1.339187},brlenIlnBrlens{4,5}_mean=0.012094,brlenIlnBrlens{4,5}_median=0.006422,brlenIlnBrlens{4,5}_95%HPD={0.000001,0.041482},rateIlnBrlens{4,5}_mean=0.996067,rateIlnBrlens{4,5}_median=0.802603,rateIlnBrlens{4,5}_95%HPD={0.068641,2.389919}],33[&prob=1.00000000e+00,prob_stddev=0.00000000e+00,prob_range={1.00000000e+00,1.00000000e+00},prob(percent)="100",prob+-sd="100+-0",height_mean=6.95375829e-08,height_median=4.60000003e-08,height_95%HPD={0.00000000e+00,2.04000000e-07},age_mean=2.01321015e-05,age_median=1.41255688e-05,age_95%HPD={0.00000000e+00,6.04012949e-05}]:5.642193e+00[&length_mean=2.07606373e-02,length_median=1.68546100e-02,length_95%HPD={1.63816800e-03,5.03118100e-02},brlenIlnBrlens{1}_mean=0.011256,brlenIlnBrlens{1}_median=0.007775,brlenIlnBrlens{1}_95%HPD={0.000075,0.032922},rateIlnBrlens{1}_mean=0.667057,rateIlnBrlens{1}_median=0.463475,rateIlnBrlens{1}_95%HPD={0.004887,1.932144},brlenIlnBrlens{2}_mean=0.017976,brlenIlnBrlens{2}_median=0.012283,brlenIlnBrlens{2}_95%HPD={0.000058,0.052322},rateIlnBrlens{2}_mean=0.900910,rateIlnBrlens{2}_median=0.789647,rateIlnBrlens{2}_95%HPD={0.027723,2.053561},brlenIlnBrlens{3}_mean=0.016840,brlenIlnBrlens{3}_median=0.013841,brlenIlnBrlens{3}_95%HPD={0.000974,0.040335},rateIlnBrlens{3}_mean=1.159227,rateIlnBrlens{3}_median=0.815393,rateIlnBrlens{3}_95%HPD={0.017313,3.196179},brlenIlnBrlens{4,5}_mean=0.020603,brlenIlnBrlens{4,5}_median=0.013167,brlenIlnBrlens{4,5}_95%HPD={0.000198,0.063236},rateIlnBrlens{4,5}_mean=0.993884,rateIlnBrlens{4,5}_median=0.809724,rateIlnBrlens{4,5}_95%HPD={0.052896,2.398823}])[&prob=6.92245471e-01,prob_stddev=3.15521150e-03,prob_range={6.90014400e-01,6.94476543e-01},prob(percent)="69",prob+-sd="69+-0",height_mean=2.13545276e-02,height_median=1.86987510e-02,height_95%HPD={3.67355600e-03,4.46772900e-02},age_mean=6.50215911e+00,age_median=5.64220692e+00,age_95%HPD={1.09940010e+00,1.39736985e+01}]:2.790153e+00[&length_mean=1.98024048e-02,length_median=1.56191200e-02,length_95%HPD={2.09141900e-07,5.06937300e-02},brlenIlnBrlens{1}_mean=0.009673,brlenIlnBrlens{1}_median=0.006287,brlenIlnBrlens{1}_95%HPD={0.000000,0.029870},rateIlnBrlens{1}_mean=0.609795,rateIlnBrlens{1}_median=0.430640,rateIlnBrlens{1}_95%HPD={0.005515,1.727268},brlenIlnBrlens{2}_mean=0.016599,brlenIlnBrlens{2}_median=0.010733,brlenIlnBrlens{2}_95%HPD={0.000000,0.051185},rateIlnBrlens{2}_mean=0.885957,rateIlnBrlens{2}_median=0.777423,rateIlnBrlens{2}_95%HPD={0.026312,2.041917},brlenIlnBrlens{3}_mean=0.013907,brlenIlnBrlens{3}_median=0.010281,brlenIlnBrlens{3}_95%HPD={0.000000,0.039123},rateIlnBrlens{3}_mean=1.087674,rateIlnBrlens{3}_median=0.657024,rateIlnBrlens{3}_95%HPD={0.004214,3.381403},brlenIlnBrlens{4,5}_mean=0.019694,brlenIlnBrlens{4,5}_median=0.011833,brlenIlnBrlens{4,5}_95%HPD={0.000000,0.063129},rateIlnBrlens{4,5}_mean=0.995125,rateIlnBrlens{4,5}_median=0.800658,rateIlnBrlens{4,5}_95%HPD={0.060981,2.441684}])[&prob=6.09606940e-01,prob_stddev=3.51975785e-04,prob_range={6.09358056e-01,6.09855825e-01},prob(percent)="61",prob+-sd="61+-0",height_mean=3.07906560e-02,height_median=2.81512740e-02,height_95%HPD={7.60054210e-03,5.95798970e-02},age_mean=9.32344318e+00,age_median=8.43236009e+00,age_95%HPD={2.07993301e+00,1.84006118e+01}]:6.687350e+00[&length_mean=2.05321170e-02,length_median=1.63540500e-02,length_95%HPD={1.12965800e-07,5.21580500e-02},brlenIlnBrlens{1}_mean=0.009122,brlenIlnBrlens{1}_median=0.005892,brlenIlnBrlens{1}_95%HPD={0.000000,0.028185},rateIlnBrlens{1}_mean=0.553993,rateIlnBrlens{1}_median=0.391785,rateIlnBrlens{1}_95%HPD={0.005985,1.587544},brlenIlnBrlens{2}_mean=0.017597,brlenIlnBrlens{2}_median=0.011397,brlenIlnBrlens{2}_95%HPD={0.000000,0.054035},rateIlnBrlens{2}_mean=0.897697,rateIlnBrlens{2}_median=0.792442,rateIlnBrlens{2}_95%HPD={0.020433,2.068907},brlenIlnBrlens{3}_mean=0.017935,brlenIlnBrlens{3}_median=0.011761,brlenIlnBrlens{3}_95%HPD={0.000000,0.056620},rateIlnBrlens{3}_mean=1.296356,rateIlnBrlens{3}_median=0.654076,rateIlnBrlens{3}_95%HPD={0.002547,4.555957},brlenIlnBrlens{4,5}_mean=0.020498,brlenIlnBrlens{4,5}_median=0.012159,brlenIlnBrlens{4,5}_95%HPD={0.000000,0.064657},rateIlnBrlens{4,5}_mean=0.998315,rateIlnBrlens{4,5}_median=0.800735,rateIlnBrlens{4,5}_95%HPD={0.058184,2.394333}])[&prob=7.81923877e-01,prob_stddev=2.08671358e-03,prob_range={7.80448348e-01,7.83399406e-01},prob(percent)="78",prob+-sd="78+-0",height_mean=5.25683325e-02,height_median=4.99983700e-02,height_95%HPD={2.38276400e-02,8.63428670e-02},age_mean=1.60043317e+01,age_median=1.51197098e+01,age_95%HPD={6.66058475e+00,2.73481084e+01}]:1.073535e+01[&length_mean=3.36906093e-02,length_median=3.05507500e-02,length_95%HPD={3.02808400e-05,7.10288600e-02},brlenIlnBrlens{1}_mean=0.015015,brlenIlnBrlens{1}_median=0.010337,brlenIlnBrlens{1}_95%HPD={0.000003,0.044130},rateIlnBrlens{1}_mean=0.502604,rateIlnBrlens{1}_median=0.368954,rateIlnBrlens{1}_95%HPD={0.006760,1.370890},brlenIlnBrlens{2}_mean=0.027059,brlenIlnBrlens{2}_median=0.019949,brlenIlnBrlens{2}_95%HPD={0.000002,0.075108},rateIlnBrlens{2}_mean=0.839561,rateIlnBrlens{2}_median=0.747500,rateIlnBrlens{2}_95%HPD={0.022434,1.863205},brlenIlnBrlens{3}_mean=0.108115,brlenIlnBrlens{3}_median=0.079768,brlenIlnBrlens{3}_95%HPD={0.000001,0.319058},rateIlnBrlens{3}_mean=3.867522,rateIlnBrlens{3}_median=2.349548,rateIlnBrlens{3}_95%HPD={0.008038,12.202279},brlenIlnBrlens{4,5}_mean=0.028742,brlenIlnBrlens{4,5}_median=0.026488,brlenIlnBrlens{4,5}_95%HPD={0.000008,0.061226},rateIlnBrlens{4,5}_mean=0.955267,rateIlnBrlens{4,5}_median=0.845485,rateIlnBrlens{4,5}_95%HPD={0.094613,2.023964}],((22[&prob=1.00000000e+00,prob_stddev=0.00000000e+00,prob_range={1.00000000e+00,1.00000000e+00},prob(percent)="100",prob+-sd="100+-0",height_mean=6.95340307e-08,height_median=4.69999999e-08,height_95%HPD={0.00000000e+00,2.03400000e-07},age_mean=2.01324607e-05,age_median=1.41761363e-05,age_95%HPD={0.00000000e+00,6.05738841e-05}]:7.386583e+00[&length_mean=2.91962218e-02,length_median=2.53435700e-02,length_95%HPD={3.88298700e-03,6.31654700e-02},brlenIlnBrlens{1}_mean=0.026278,brlenIlnBrlens{1}_median=0.020791,brlenIlnBrlens{1}_95%HPD={0.000703,0.066816},rateIlnBrlens{1}_mean=1.082638,rateIlnBrlens{1}_median=0.811520,rateIlnBrlens{1}_95%HPD={0.019550,2.862214},brlenIlnBrlens{2}_mean=0.024281,brlenIlnBrlens{2}_median=0.018217,brlenIlnBrlens{2}_95%HPD={0.000238,0.065358},rateIlnBrlens{2}_mean=0.863018,rateIlnBrlens{2}_median=0.770108,rateIlnBrlens{2}_95%HPD={0.015822,1.946383},brlenIlnBrlens{3}_mean=0.014110,brlenIlnBrlens{3}_median=0.011019,brlenIlnBrlens{3}_95%HPD={0.000133,0.036831},rateIlnBrlens{3}_mean=0.619953,rateIlnBrlens{3}_median=0.431020,rateIlnBrlens{3}_95%HPD={0.005003,1.787960},brlenIlnBrlens{4,5}_mean=0.026399,brlenIlnBrlens{4,5}_median=0.021397,brlenIlnBrlens{4,5}_95%HPD={0.000850,0.065791},rateIlnBrlens{4,5}_mean=0.957733,rateIlnBrlens{4,5}_median=0.835123,rateIlnBrlens{4,5}_95%HPD={0.081171,2.069981}],((25[&prob=1.00000000e+00,prob_stddev=0.00000000e+00,prob_range={1.00000000e+00,1.00000000e+00},prob(percent)="100",prob+-sd="100+-0",height_mean=6.95428489e-08,height_median=4.60000000e-08,height_95%HPD={0.00000000e+00,2.03700000e-07},age_mean=2.01346238e-05,age_median=1.41453191e-05,age_95%HPD={0.00000000e+00,6.05204152e-05}]:1.043432e+00[&length_mean=5.75847705e-03,length_median=4.24027800e-03,length_95%HPD={2.04089900e-05,1.59543500e-02},brlenIlnBrlens{1}_mean=0.003524,brlenIlnBrlens{1}_median=0.001978,brlenIlnBrlens{1}_95%HPD={0.000001,0.012001},rateIlnBrlens{1}_mean=0.711488,rateIlnBrlens{1}_median=0.489819,rateIlnBrlens{1}_95%HPD={0.009437,2.070064},brlenIlnBrlens{2}_mean=0.005410,brlenIlnBrlens{2}_median=0.003168,brlenIlnBrlens{2}_95%HPD={0.000005,0.017817},rateIlnBrlens{2}_mean=0.966973,rateIlnBrlens{2}_median=0.821271,rateIlnBrlens{2}_95%HPD={0.018964,2.271682},brlenIlnBrlens{3}_mean=0.002033,brlenIlnBrlens{3}_median=0.001131,brlenIlnBrlens{3}_95%HPD={0.000001,0.007003},rateIlnBrlens{3}_mean=0.464914,rateIlnBrlens{3}_median=0.269849,rateIlnBrlens{3}_95%HPD={0.002386,1.486099},brlenIlnBrlens{4,5}_mean=0.005790,brlenIlnBrlens{4,5}_median=0.003290,brlenIlnBrlens{4,5}_95%HPD={0.000005,0.019069},rateIlnBrlens{4,5}_mean=1.012307,rateIlnBrlens{4,5}_median=0.821077,rateIlnBrlens{4,5}_95%HPD={0.055835,2.407252}],27[&prob=1.00000000e+00,prob_stddev=0.00000000e+00,prob_range={1.00000000e+00,1.00000000e+00},prob(percent)="100",prob+-sd="100+-0",height_mean=6.95432963e-08,height_median=4.60000000e-08,height_95%HPD={0.00000000e+00,2.03500000e-07},age_mean=2.01346797e-05,age_median=1.41382942e-05,age_95%HPD={0.00000000e+00,6.05622044e-05}]:1.043432e+00[&length_mean=6.93110888e-03,length_median=5.16601000e-03,length_95%HPD={2.14173800e-05,1.87895700e-02},brlenIlnBrlens{1}_mean=0.004068,brlenIlnBrlens{1}_median=0.002346,brlenIlnBrlens{1}_95%HPD={0.000002,0.013630},rateIlnBrlens{1}_mean=0.683164,rateIlnBrlens{1}_median=0.476237,rateIlnBrlens{1}_95%HPD={0.004134,1.963765},brlenIlnBrlens{2}_mean=0.006411,brlenIlnBrlens{2}_median=0.003821,brlenIlnBrlens{2}_95%HPD={0.000005,0.020787},rateIlnBrlens{2}_mean=0.955740,rateIlnBrlens{2}_median=0.817251,rateIlnBrlens{2}_95%HPD={0.013899,2.241614},brlenIlnBrlens{3}_mean=0.004608,brlenIlnBrlens{3}_median=0.003106,brlenIlnBrlens{3}_95%HPD={0.000010,0.013738},rateIlnBrlens{3}_mean=0.979500,rateIlnBrlens{3}_median=0.612556,rateIlnBrlens{3}_95%HPD={0.007413,3.065294},brlenIlnBrlens{4,5}_mean=0.006832,brlenIlnBrlens{4,5}_median=0.003929,brlenIlnBrlens{4,5}_95%HPD={0.000004,0.022111},rateIlnBrlens{4,5}_mean=0.993282,rateIlnBrlens{4,5}_median=0.797908,rateIlnBrlens{4,5}_95%HPD={0.041426,2.362054}])[&prob=3.87708663e-01,prob_stddev=4.37455618e-03,prob_range={3.84615385e-01,3.90801941e-01},prob(percent)="39",prob+-sd="39+-0",height_mean=4.60032771e-03,height_median=3.46617000e-03,height_95%HPD={1.86792000e-05,1.24074070e-02},age_mean=1.39181999e+00,age_median=1.04344596e+00,age_95%HPD={6.65119029e-03,3.75821889e+00}]:1.268366e+00[&length_mean=5.22067142e-03,length_median=3.43110300e-03,length_95%HPD={2.90075700e-07,1.60519200e-02},brlenIlnBrlens{1}_mean=0.003219,brlenIlnBrlens{1}_median=0.001620,brlenIlnBrlens{1}_95%HPD={0.000000,0.011703},rateIlnBrlens{1}_mean=0.746347,rateIlnBrlens{1}_median=0.515496,rateIlnBrlens{1}_95%HPD={0.003528,2.122190},brlenIlnBrlens{2}_mean=0.004760,brlenIlnBrlens{2}_median=0.002449,brlenIlnBrlens{2}_95%HPD={0.000000,0.016700},rateIlnBrlens{2}_mean=0.952532,rateIlnBrlens{2}_median=0.806855,rateIlnBrlens{2}_95%HPD={0.024879,2.285678},brlenIlnBrlens{3}_mean=0.002179,brlenIlnBrlens{3}_median=0.001071,brlenIlnBrlens{3}_95%HPD={0.000000,0.008028},rateIlnBrlens{3}_mean=0.597484,rateIlnBrlens{3}_median=0.326025,rateIlnBrlens{3}_95%HPD={0.001838,1.986975},brlenIlnBrlens{4,5}_mean=0.005147,brlenIlnBrlens{4,5}_median=0.002577,brlenIlnBrlens{4,5}_95%HPD={0.000000,0.018174},rateIlnBrlens{4,5}_mean=0.990670,rateIlnBrlens{4,5}_median=0.805006,rateIlnBrlens{4,5}_95%HPD={0.065738,2.349819}],26[&prob=1.00000000e+00,prob_stddev=0.00000000e+00,prob_range={1.00000000e+00,1.00000000e+00},prob(percent)="100",prob+-sd="100+-0",height_mean=6.95420716e-08,height_median=4.60000000e-08,height_95%HPD={0.00000000e+00,2.03200000e-07},age_mean=2.01348403e-05,age_median=1.41276422e-05,age_95%HPD={0.00000000e+00,6.05168703e-05}]:2.311798e+00[&length_mean=7.04825222e-03,length_median=5.25054100e-03,length_95%HPD={4.47846500e-05,1.91565300e-02},brlenIlnBrlens{1}_mean=0.004237,brlenIlnBrlens{1}_median=0.002447,brlenIlnBrlens{1}_95%HPD={0.000004,0.014182},rateIlnBrlens{1}_mean=0.697476,rateIlnBrlens{1}_median=0.487025,rateIlnBrlens{1}_95%HPD={0.004790,1.985042},brlenIlnBrlens{2}_mean=0.006451,brlenIlnBrlens{2}_median=0.003875,brlenIlnBrlens{2}_95%HPD={0.000003,0.020692},rateIlnBrlens{2}_mean=0.942226,rateIlnBrlens{2}_median=0.811118,rateIlnBrlens{2}_95%HPD={0.023416,2.150823},brlenIlnBrlens{3}_mean=0.004560,brlenIlnBrlens{3}_median=0.003105,brlenIlnBrlens{3}_95%HPD={0.000013,0.013563},rateIlnBrlens{3}_mean=0.961066,rateIlnBrlens{3}_median=0.608483,rateIlnBrlens{3}_95%HPD={0.003974,2.968929},brlenIlnBrlens{4,5}_mean=0.007083,brlenIlnBrlens{4,5}_median=0.004047,brlenIlnBrlens{4,5}_95%HPD={0.000012,0.022881},rateIlnBrlens{4,5}_mean=1.007138,rateIlnBrlens{4,5}_median=0.810997,rateIlnBrlens{4,5}_95%HPD={0.033280,2.427461}])[&prob=9.39423299e-01,prob_stddev=1.26962694e-03,prob_range={9.38525537e-01,9.40321061e-01},prob(percent)="94",prob+-sd="94+-0",height_mean=9.32570252e-03,height_median=7.69274100e-03,height_95%HPD={7.00168000e-04,2.20323200e-02},age_mean=2.81371744e+00,age_median=2.31181163e+00,age_95%HPD={1.62864365e-01,6.63421557e+00}]:5.074786e+00[&length_mean=2.16994776e-02,length_median=1.76592400e-02,length_95%HPD={4.32588400e-07,5.30115800e-02},brlenIlnBrlens{1}_mean=0.022935,brlenIlnBrlens{1}_median=0.016759,brlenIlnBrlens{1}_95%HPD={0.000000,0.064813},rateIlnBrlens{1}_mean=1.281924,rateIlnBrlens{1}_median=0.928961,rateIlnBrlens{1}_95%HPD={0.021142,3.445058},brlenIlnBrlens{2}_mean=0.018334,brlenIlnBrlens{2}_median=0.012382,brlenIlnBrlens{2}_95%HPD={0.000000,0.054406},rateIlnBrlens{2}_mean=0.884976,rateIlnBrlens{2}_median=0.779263,rateIlnBrlens{2}_95%HPD={0.028891,2.035570},brlenIlnBrlens{3}_mean=0.007979,brlenIlnBrlens{3}_median=0.005371,brlenIlnBrlens{3}_95%HPD={0.000000,0.024299},rateIlnBrlens{3}_mean=0.497063,rateIlnBrlens{3}_median=0.306422,rateIlnBrlens{3}_95%HPD={0.001924,1.585573},brlenIlnBrlens{4,5}_mean=0.021685,brlenIlnBrlens{4,5}_median=0.013545,brlenIlnBrlens{4,5}_95%HPD={0.000000,0.066501},rateIlnBrlens{4,5}_mean=0.998935,rateIlnBrlens{4,5}_median=0.808065,rateIlnBrlens{4,5}_95%HPD={0.054407,2.395041}])[&prob=8.12821105e-01,prob_stddev=6.66239878e-03,prob_range={8.08110078e-01,8.17532133e-01},prob(percent)="81",prob+-sd="81+-1",height_mean=2.77602850e-02,height_median=2.44496000e-02,height_95%HPD={5.91246500e-03,5.82715200e-02},age_mean=8.40128242e+00,age_median=7.38659743e+00,age_95%HPD={1.51433549e+00,1.75694990e+01}]:5.882804e+00[&length_mean=2.99648485e-02,length_median=2.46143000e-02,length_95%HPD={3.66914500e-07,7.27872800e-02},brlenIlnBrlens{1}_mean=0.020743,brlenIlnBrlens{1}_median=0.014764,brlenIlnBrlens{1}_95%HPD={0.000000,0.060089},rateIlnBrlens{1}_mean=0.856830,rateIlnBrlens{1}_median=0.614979,rateIlnBrlens{1}_95%HPD={0.010800,2.374959},brlenIlnBrlens{2}_mean=0.024041,brlenIlnBrlens{2}_median=0.016341,brlenIlnBrlens{2}_95%HPD={0.000000,0.071671},rateIlnBrlens{2}_mean=0.851916,rateIlnBrlens{2}_median=0.751755,rateIlnBrlens{2}_95%HPD={0.021138,1.885248},brlenIlnBrlens{3}_mean=0.034511,brlenIlnBrlens{3}_median=0.021372,brlenIlnBrlens{3}_95%HPD={0.000000,0.109715},rateIlnBrlens{3}_mean=1.409857,rateIlnBrlens{3}_median=0.829709,rateIlnBrlens{3}_95%HPD={0.005612,4.713660},brlenIlnBrlens{4,5}_mean=0.026288,brlenIlnBrlens{4,5}_median=0.020587,brlenIlnBrlens{4,5}_95%HPD={0.000000,0.068099},rateIlnBrlens{4,5}_mean=0.960811,rateIlnBrlens{4,5}_median=0.836723,rateIlnBrlens{4,5}_95%HPD={0.100500,2.118063}],30[&prob=1.00000000e+00,prob_stddev=0.00000000e+00,prob_range={1.00000000e+00,1.00000000e+00},prob(percent)="100",prob+-sd="100+-0",height_mean=6.95511733e-08,height_median=4.69999999e-08,height_95%HPD={0.00000000e+00,2.04000000e-07},age_mean=2.01350618e-05,age_median=1.41645859e-05,age_95%HPD={0.00000000e+00,6.06904023e-05}]:1.326939e+01[&length_mean=5.07359137e-02,length_median=4.60362500e-02,length_95%HPD={8.71191500e-03,1.01740300e-01},brlenIlnBrlens{1}_mean=0.019709,brlenIlnBrlens{1}_median=0.014539,brlenIlnBrlens{1}_95%HPD={0.000183,0.054314},rateIlnBrlens{1}_mean=0.449283,rateIlnBrlens{1}_median=0.320714,rateIlnBrlens{1}_95%HPD={0.007262,1.252626},brlenIlnBrlens{2}_mean=0.039114,brlenIlnBrlens{2}_median=0.030462,brlenIlnBrlens{2}_95%HPD={0.000596,0.101284},rateIlnBrlens{2}_mean=0.800795,rateIlnBrlens{2}_median=0.720440,rateIlnBrlens{2}_95%HPD={0.025478,1.768961},brlenIlnBrlens{3}_mean=0.059217,brlenIlnBrlens{3}_median=0.048000,brlenIlnBrlens{3}_95%HPD={0.000301,0.147052},rateIlnBrlens{3}_mean=1.654550,rateIlnBrlens{3}_median=1.050231,rateIlnBrlens{3}_95%HPD={0.004761,4.685916},brlenIlnBrlens{4,5}_mean=0.050052,brlenIlnBrlens{4,5}_median=0.035452,brlenIlnBrlens{4,5}_95%HPD={0.000902,0.139545},rateIlnBrlens{4,5}_mean=0.989824,rateIlnBrlens{4,5}_median=0.798091,rateIlnBrlens{4,5}_95%HPD={0.033265,2.359339}])[&prob=8.00901317e-01,prob_stddev=4.51283238e-03,prob_range={7.97710263e-01,8.04092372e-01},prob(percent)="80",prob+-sd="80+-0",height_mean=4.75258685e-02,height_median=4.42217000e-02,height_95%HPD={1.51977700e-02,8.67139850e-02},age_mean=1.44187909e+01,age_median=1.32694013e+01,age_95%HPD={4.37950174e+00,2.71184512e+01}]:1.258566e+01[&length_mean=3.63279841e-02,length_median=3.31427700e-02,length_95%HPD={3.36172700e-06,7.56234100e-02},brlenIlnBrlens{1}_mean=0.015147,brlenIlnBrlens{1}_median=0.010425,brlenIlnBrlens{1}_95%HPD={0.000002,0.044347},rateIlnBrlens{1}_mean=0.475711,rateIlnBrlens{1}_median=0.341760,rateIlnBrlens{1}_95%HPD={0.003474,1.324010},brlenIlnBrlens{2}_mean=0.028901,brlenIlnBrlens{2}_median=0.021915,brlenIlnBrlens{2}_95%HPD={0.000002,0.077972},rateIlnBrlens{2}_mean=0.834991,rateIlnBrlens{2}_median=0.749392,rateIlnBrlens{2}_95%HPD={0.028826,1.839193},brlenIlnBrlens{3}_mean=0.127334,brlenIlnBrlens{3}_median=0.101559,brlenIlnBrlens{3}_95%HPD={0.000000,0.352903},rateIlnBrlens{3}_mean=4.422177,rateIlnBrlens{3}_median=2.903553,rateIlnBrlens{3}_95%HPD={0.005581,13.799495},brlenIlnBrlens{4,5}_mean=0.031037,brlenIlnBrlens{4,5}_median=0.027591,brlenIlnBrlens{4,5}_95%HPD={0.000003,0.069335},rateIlnBrlens{4,5}_mean=0.935686,rateIlnBrlens{4,5}_median=0.828002,rateIlnBrlens{4,5}_95%HPD={0.105194,2.028488}])[&prob=9.98231143e-01,prob_stddev=3.14264094e-04,prob_range={9.98008924e-01,9.98453361e-01},prob(percent)="100",prob+-sd="100+-0",height_mean=8.94319464e-02,height_median=8.54347850e-02,height_95%HPD={5.06482200e-02,1.39057390e-01},age_mean=2.71256132e+01,age_median=2.58550640e+01,age_95%HPD={1.34654932e+01,4.35832335e+01}]:3.255166e+01[&length_mean=1.20619180e-01,length_median=1.13073900e-01,length_95%HPD={3.31704400e-02,2.26598600e-01},brlenIlnBrlens{1}_mean=0.083524,brlenIlnBrlens{1}_median=0.065246,brlenIlnBrlens{1}_95%HPD={0.001081,0.215696},rateIlnBrlens{1}_mean=0.761811,rateIlnBrlens{1}_median=0.591705,rateIlnBrlens{1}_95%HPD={0.019899,1.961202},brlenIlnBrlens{2}_mean=0.080973,brlenIlnBrlens{2}_median=0.066631,brlenIlnBrlens{2}_95%HPD={0.001317,0.198988},rateIlnBrlens{2}_mean=0.696994,rateIlnBrlens{2}_median=0.635413,rateIlnBrlens{2}_95%HPD={0.017515,1.478331},brlenIlnBrlens{3}_mean=0.192259,brlenIlnBrlens{3}_median=0.106652,brlenIlnBrlens{3}_95%HPD={0.000024,0.643825},rateIlnBrlens{3}_mean=1.599183,rateIlnBrlens{3}_median=0.965017,rateIlnBrlens{3}_95%HPD={0.001326,5.144492},brlenIlnBrlens{4,5}_mean=0.180081,brlenIlnBrlens{4,5}_median=0.176508,brlenIlnBrlens{4,5}_95%HPD={0.094096,0.277046},rateIlnBrlens{4,5}_mean=1.778793,rateIlnBrlens{4,5}_median=1.556277,rateIlnBrlens{4,5}_95%HPD={0.519981,3.649010}],(28[&prob=1.00000000e+00,prob_stddev=0.00000000e+00,prob_range={1.00000000e+00,1.00000000e+00},prob(percent)="100",prob+-sd="100+-0",height_mean=6.94718838e-08,height_median=4.99999997e-08,height_95%HPD={0.00000000e+00,2.09610000e-07},age_mean=2.01160019e-05,age_median=1.46385129e-05,age_95%HPD={0.00000000e+00,6.16814590e-05}]:4.598259e+00[&length_mean=1.96389340e-02,length_median=1.53630000e-02,length_95%HPD={1.28658000e-03,5.03010000e-02},brlenIlnBrlens{1}_mean=0.021534,brlenIlnBrlens{1}_median=0.014425,brlenIlnBrlens{1}_95%HPD={0.000037,0.065226},rateIlnBrlens{1}_mean=1.359030,rateIlnBrlens{1}_median=0.920057,rateIlnBrlens{1}_95%HPD={0.007149,4.029374},brlenIlnBrlens{2}_mean=0.016602,brlenIlnBrlens{2}_median=0.011057,brlenIlnBrlens{2}_95%HPD={0.000063,0.049907},rateIlnBrlens{2}_mean=0.891765,rateIlnBrlens{2}_median=0.785323,rateIlnBrlens{2}_95%HPD={0.023157,2.061509},brlenIlnBrlens{3}_mean=0.005558,brlenIlnBrlens{3}_median=0.003803,brlenIlnBrlens{3}_95%HPD={0.000020,0.016534},rateIlnBrlens{3}_mean=0.386054,rateIlnBrlens{3}_median=0.240965,rateIlnBrlens{3}_95%HPD={0.004241,1.202155},brlenIlnBrlens{4,5}_mean=0.023614,brlenIlnBrlens{4,5}_median=0.012887,brlenIlnBrlens{4,5}_95%HPD={0.000132,0.082800},rateIlnBrlens{4,5}_mean=1.148830,rateIlnBrlens{4,5}_median=0.884551,rateIlnBrlens{4,5}_95%HPD={0.053219,2.903717}],29[&prob=1.00000000e+00,prob_stddev=0.00000000e+00,prob_range={1.00000000e+00,1.00000000e+00},prob(percent)="100",prob+-sd="100+-0",height_mean=6.94718838e-08,height_median=4.99999997e-08,height_95%HPD={0.00000000e+00,2.09610000e-07},age_mean=2.01160019e-05,age_median=1.46385129e-05,age_95%HPD={0.00000000e+00,6.16814590e-05}]:4.598259e+00[&length_mean=1.96389340e-02,length_median=1.53630000e-02,length_95%HPD={1.28658000e-03,5.03010000e-02},brlenIlnBrlens{1}_mean=0.016085,brlenIlnBrlens{1}_median=0.010565,brlenIlnBrlens{1}_95%HPD={0.000033,0.049299},rateIlnBrlens{1}_mean=1.021832,rateIlnBrlens{1}_median=0.669042,rateIlnBrlens{1}_95%HPD={0.008503,3.107861},brlenIlnBrlens{2}_mean=0.016821,brlenIlnBrlens{2}_median=0.011024,brlenIlnBrlens{2}_95%HPD={0.000081,0.050878},rateIlnBrlens{2}_mean=0.890994,rateIlnBrlens{2}_median=0.784520,rateIlnBrlens{2}_95%HPD={0.022980,2.050980},brlenIlnBrlens{3}_mean=0.005570,brlenIlnBrlens{3}_median=0.003822,brlenIlnBrlens{3}_95%HPD={0.000021,0.016298},rateIlnBrlens{3}_mean=0.388526,rateIlnBrlens{3}_median=0.243477,rateIlnBrlens{3}_95%HPD={0.002487,1.201177},brlenIlnBrlens{4,5}_mean=0.019725,brlenIlnBrlens{4,5}_median=0.011847,brlenIlnBrlens{4,5}_95%HPD={0.000128,0.061823},rateIlnBrlens{4,5}_mean=1.000749,rateIlnBrlens{4,5}_median=0.806114,rateIlnBrlens{4,5}_95%HPD={0.064110,2.376456}])[&prob=1.00000000e+00,prob_stddev=0.00000000e+00,prob_range={1.00000000e+00,1.00000000e+00},prob(percent)="100",prob+-sd="100+-0",height_mean=1.96390035e-02,height_median=1.53630600e-02,height_95%HPD={1.28668100e-03,5.03012700e-02},age_mean=5.90773076e+00,age_median=4.59827317e+00,age_95%HPD={3.88212340e-01,1.52635512e+01}]:5.380845e+01[&length_mean=1.92614054e-01,length_median=1.82352800e-01,length_95%HPD={8.29820800e-02,3.23352400e-01},brlenIlnBrlens{1}_mean=0.126984,brlenIlnBrlens{1}_median=0.106216,brlenIlnBrlens{1}_95%HPD={0.003305,0.297351},rateIlnBrlens{1}_mean=0.707057,rateIlnBrlens{1}_median=0.577125,rateIlnBrlens{1}_95%HPD={0.014769,1.690360},brlenIlnBrlens{2}_mean=0.118321,brlenIlnBrlens{2}_median=0.099202,brlenIlnBrlens{2}_95%HPD={0.001894,0.281230},rateIlnBrlens{2}_mean=0.631277,rateIlnBrlens{2}_median=0.566360,rateIlnBrlens{2}_95%HPD={0.011256,1.362757},brlenIlnBrlens{3}_mean=0.419273,brlenIlnBrlens{3}_median=0.310219,brlenIlnBrlens{3}_95%HPD={0.001921,1.137865},rateIlnBrlens{3}_mean=2.246607,rateIlnBrlens{3}_median=1.700723,rateIlnBrlens{3}_95%HPD={0.007194,5.903411},brlenIlnBrlens{4,5}_mean=0.271419,brlenIlnBrlens{4,5}_median=0.267083,brlenIlnBrlens{4,5}_95%HPD={0.107277,0.439741},rateIlnBrlens{4,5}_mean=1.535655,rateIlnBrlens{4,5}_median=1.461854,rateIlnBrlens{4,5}_95%HPD={0.364679,2.708724}])[&prob=7.27720396e-01,prob_stddev=1.58389103e-02,prob_range={7.16520595e-01,7.38920197e-01},prob(percent)="73",prob+-sd="73+-2",height_mean=2.01299625e-01,height_median=1.93234600e-01,height_95%HPD={1.06606880e-01,3.09169790e-01},age_mean=6.06494508e+01,age_median=5.84067261e+01,age_95%HPD={2.78897004e+01,9.64238235e+01}]:1.951404e+01[&length_mean=6.64713994e-02,length_median=5.50717000e-02,length_95%HPD={7.41601200e-06,1.58496900e-01},brlenIlnBrlens{1}_mean=0.042922,brlenIlnBrlens{1}_median=0.027514,brlenIlnBrlens{1}_95%HPD={0.000003,0.134812},rateIlnBrlens{1}_mean=0.727938,rateIlnBrlens{1}_median=0.518403,rateIlnBrlens{1}_95%HPD={0.007730,2.065968},brlenIlnBrlens{2}_mean=0.049337,brlenIlnBrlens{2}_median=0.034524,brlenIlnBrlens{2}_95%HPD={0.000005,0.143122},rateIlnBrlens{2}_mean=0.788994,rateIlnBrlens{2}_median=0.692920,rateIlnBrlens{2}_95%HPD={0.023223,1.797922},brlenIlnBrlens{3}_mean=0.405113,brlenIlnBrlens{3}_median=0.324450,brlenIlnBrlens{3}_95%HPD={0.000001,1.043560},rateIlnBrlens{3}_mean=7.855949,rateIlnBrlens{3}_median=5.459267,rateIlnBrlens{3}_95%HPD={0.006627,22.182684},brlenIlnBrlens{4,5}_mean=0.033503,brlenIlnBrlens{4,5}_median=0.028280,brlenIlnBrlens{4,5}_95%HPD={0.000003,0.079638},rateIlnBrlens{4,5}_mean=0.617576,rateIlnBrlens{4,5}_median=0.527191,rateIlnBrlens{4,5}_95%HPD={0.032727,1.376560}],24[&prob=1.00000000e+00,prob_stddev=0.00000000e+00,prob_range={1.00000000e+00,1.00000000e+00},prob(percent)="100",prob+-sd="100+-0",height_mean=6.94249954e-08,height_median=4.99999999e-08,height_95%HPD={0.00000000e+00,2.07000000e-07},age_mean=2.00819959e-05,age_median=1.46949790e-05,age_95%HPD={0.00000000e+00,6.13230611e-05}]:7.792075e+01[&length_mean=2.52381667e-01,length_median=2.41871800e-01,length_95%HPD={1.22709000e-01,4.02046900e-01},brlenIlnBrlens{1}_mean=0.135429,brlenIlnBrlens{1}_median=0.112365,brlenIlnBrlens{1}_95%HPD={0.005135,0.325266},rateIlnBrlens{1}_mean=0.560508,rateIlnBrlens{1}_median=0.467257,rateIlnBrlens{1}_95%HPD={0.014732,1.334530},brlenIlnBrlens{2}_mean=0.148821,brlenIlnBrlens{2}_median=0.124266,brlenIlnBrlens{2}_95%HPD={0.002625,0.347801},rateIlnBrlens{2}_mean=0.599741,rateIlnBrlens{2}_median=0.528509,rateIlnBrlens{2}_95%HPD={0.022699,1.284240},brlenIlnBrlens{3}_mean=0.023261,brlenIlnBrlens{3}_median=0.017219,brlenIlnBrlens{3}_95%HPD={0.000444,0.059410},rateIlnBrlens{3}_mean=0.095917,rateIlnBrlens{3}_median=0.070870,rateIlnBrlens{3}_95%HPD={0.001746,0.242540},brlenIlnBrlens{4,5}_mean=0.137008,brlenIlnBrlens{4,5}_median=0.133602,brlenIlnBrlens{4,5}_95%HPD={0.060514,0.217550},rateIlnBrlens{4,5}_mean=0.582087,rateIlnBrlens{4,5}_median=0.556202,rateIlnBrlens{4,5}_95%HPD={0.186506,1.009199}])[&prob=9.97768929e-01,prob_stddev=1.88558456e-04,prob_range={9.97635598e-01,9.97902260e-01},prob(percent)="100",prob+-sd="100+-0",height_mean=2.66266123e-01,height_median=2.55359960e-01,height_95%HPD={1.44253332e-01,4.10905500e-01},age_mean=8.05261930e+01,age_median=7.79207679e+01,age_95%HPD={3.98630547e+01,1.26906975e+02}]:8.007926e+01[&length_mean=2.93442028e-01,length_median=2.75258900e-01,length_95%HPD={4.25337400e-02,5.58519500e-01},brlenIlnBrlens{1}_mean=0.394894,brlenIlnBrlens{1}_median=0.319532,brlenIlnBrlens{1}_95%HPD={0.013076,0.954324},rateIlnBrlens{1}_mean=1.541336,rateIlnBrlens{1}_median=1.214249,rateIlnBrlens{1}_95%HPD={0.059489,3.787235},brlenIlnBrlens{2}_mean=0.195522,brlenIlnBrlens{2}_median=0.156398,brlenIlnBrlens{2}_95%HPD={0.001833,0.486348},rateIlnBrlens{2}_mean=0.686374,rateIlnBrlens{2}_median=0.622463,rateIlnBrlens{2}_95%HPD={0.029456,1.449945},brlenIlnBrlens{3}_mean=0.023548,brlenIlnBrlens{3}_median=0.017461,brlenIlnBrlens{3}_95%HPD={0.000492,0.060691},rateIlnBrlens{3}_mean=0.095118,rateIlnBrlens{3}_median=0.065154,rateIlnBrlens{3}_95%HPD={0.002479,0.269712},brlenIlnBrlens{4,5}_mean=0.197933,brlenIlnBrlens{4,5}_median=0.165156,brlenIlnBrlens{4,5}_95%HPD={0.010899,0.470735},rateIlnBrlens{4,5}_mean=0.731701,rateIlnBrlens{4,5}_median=0.636614,rateIlnBrlens{4,5}_95%HPD={0.045771,1.589106}],41[&prob=1.00000000e+00,prob_stddev=0.00000000e+00,prob_range={1.00000000e+00,1.00000000e+00},prob(percent)="100",prob+-sd="100+-0",height_mean=5.41432445e-01,height_median=5.20811124e-01,height_95%HPD={3.07828800e-01,8.20699374e-01},age_mean=1.58000020e+02,age_median=1.58000018e+02,age_95%HPD={1.57999975e+02,1.58000068e+02}]:7.809001e-06[&length_mean=7.92704444e-03,length_median=0.00000000e+00,length_95%HPD={0.00000000e+00,2.50376900e-02},brlenIlnBrlens{1}_mean=0.004531,brlenIlnBrlens{1}_median=0.000000,brlenIlnBrlens{1}_95%HPD={0.000000,0.020791},rateIlnBrlens{1}_mean=0.794124,rateIlnBrlens{1}_median=1.000000,rateIlnBrlens{1}_95%HPD={0.020420,1.182868},brlenIlnBrlens{2}_mean=0.006694,brlenIlnBrlens{2}_median=0.000000,brlenIlnBrlens{2}_95%HPD={0.000000,0.027284},rateIlnBrlens{2}_mean=0.920316,rateIlnBrlens{2}_median=1.000000,rateIlnBrlens{2}_95%HPD={0.065726,1.409063},brlenIlnBrlens{3}_mean=0.001885,brlenIlnBrlens{3}_median=0.000000,brlenIlnBrlens{3}_95%HPD={0.000000,0.008726},rateIlnBrlens{3}_mean=0.637782,rateIlnBrlens{3}_median=1.000000,rateIlnBrlens{3}_95%HPD={0.039212,1.000000},brlenIlnBrlens{4,5}_mean=0.007316,brlenIlnBrlens{4,5}_median=0.000000,brlenIlnBrlens{4,5}_95%HPD={0.000000,0.029648},rateIlnBrlens{4,5}_mean=0.953173,rateIlnBrlens{4,5}_median=1.000000,rateIlnBrlens{4,5}_95%HPD={0.107082,1.633970}])[&prob=4.09459387e-01,prob_stddev=4.01000983e-03,prob_range={4.06623882e-01,4.12294893e-01},prob(percent)="41",prob+-sd="41+-0",height_mean=5.45491597e-01,height_median=5.24639910e-01,height_95%HPD={3.13206108e-01,8.24493400e-01},age_mean=1.58577615e+02,age_median=1.58000025e+02,age_95%HPD={1.57999943e+02,1.61985875e+02}]:5.000010e+00[&length_mean=1.56831398e-02,length_median=1.55260100e-02,length_95%HPD={3.50997900e-07,2.61277600e-02},brlenIlnBrlens{1}_mean=0.010617,brlenIlnBrlens{1}_median=0.006869,brlenIlnBrlens{1}_95%HPD={0.000000,0.032897},rateIlnBrlens{1}_mean=0.672463,rateIlnBrlens{1}_median=0.478873,rateIlnBrlens{1}_95%HPD={0.006428,1.913168},brlenIlnBrlens{2}_mean=0.014544,brlenIlnBrlens{2}_median=0.011186,brlenIlnBrlens{2}_95%HPD={0.000000,0.038594},rateIlnBrlens{2}_mean=0.915617,rateIlnBrlens{2}_median=0.781747,rateIlnBrlens{2}_95%HPD={0.031473,2.125443},brlenIlnBrlens{3}_mean=0.004966,brlenIlnBrlens{3}_median=0.003011,brlenIlnBrlens{3}_95%HPD={0.000000,0.016106},rateIlnBrlens{3}_mean=0.331334,rateIlnBrlens{3}_median=0.209882,rateIlnBrlens{3}_95%HPD={0.003149,1.012429},brlenIlnBrlens{4,5}_mean=0.015695,brlenIlnBrlens{4,5}_median=0.011343,brlenIlnBrlens{4,5}_95%HPD={0.000000,0.042339},rateIlnBrlens{4,5}_mean=0.976712,rateIlnBrlens{4,5}_median=0.780751,rateIlnBrlens{4,5}_95%HPD={0.051379,2.335261}],40[&prob=1.00000000e+00,prob_stddev=0.00000000e+00,prob_range={1.00000000e+00,1.00000000e+00},prob(percent)="100",prob+-sd="100+-0",height_mean=5.58566381e-01,height_median=5.37292484e-01,height_95%HPD={3.17570217e-01,8.46670874e-01},age_mean=1.63000020e+02,age_median=1.63000018e+02,age_95%HPD={1.62999974e+02,1.63000069e+02}]:1.806676e-05[&length_mean=1.09126319e-03,length_median=0.00000000e+00,length_95%HPD={0.00000000e+00,6.57212600e-03},brlenIlnBrlens{1}_mean=0.000562,brlenIlnBrlens{1}_median=0.000000,brlenIlnBrlens{1}_95%HPD={0.000000,0.002488},rateIlnBrlens{1}_mean=0.955399,rateIlnBrlens{1}_median=1.000000,rateIlnBrlens{1}_95%HPD={0.322247,1.000061},brlenIlnBrlens{2}_mean=0.000871,brlenIlnBrlens{2}_median=0.000000,brlenIlnBrlens{2}_95%HPD={0.000000,0.004590},rateIlnBrlens{2}_mean=0.981589,rateIlnBrlens{2}_median=1.000000,rateIlnBrlens{2}_95%HPD={0.661605,1.107846},brlenIlnBrlens{3}_mean=0.000217,brlenIlnBrlens{3}_median=0.000000,brlenIlnBrlens{3}_95%HPD={0.000000,0.001059},rateIlnBrlens{3}_mean=0.927968,rateIlnBrlens{3}_median=1.000000,rateIlnBrlens{3}_95%HPD={0.159433,1.000000},brlenIlnBrlens{4,5}_mean=0.000822,brlenIlnBrlens{4,5}_median=0.000000,brlenIlnBrlens{4,5}_95%HPD={0.000000,0.004110},rateIlnBrlens{4,5}_mean=0.975342,rateIlnBrlens{4,5}_median=1.000000,rateIlnBrlens{4,5}_95%HPD={0.486973,1.000000}])[&prob=5.65483280e-01,prob_stddev=1.16906243e-02,prob_range={5.57216761e-01,5.73749800e-01},prob(percent)="57",prob+-sd="57+-1",height_mean=5.65675290e-01,height_median=5.43636428e-01,height_95%HPD={3.19704222e-01,8.54859753e-01},age_mean=1.66558299e+02,age_median=1.63000036e+02,age_95%HPD={1.62999941e+02,1.83236647e+02}]:1.909278e+01[&length_mean=1.04325139e-01,length_median=8.43409300e-02,length_95%HPD={4.15005700e-06,2.64432700e-01},brlenIlnBrlens{1}_mean=0.074429,brlenIlnBrlens{1}_median=0.042391,brlenIlnBrlens{1}_95%HPD={0.000004,0.252060},rateIlnBrlens{1}_mean=0.810618,rateIlnBrlens{1}_median=0.541774,rateIlnBrlens{1}_95%HPD={0.013997,2.399119},brlenIlnBrlens{2}_mean=0.087346,brlenIlnBrlens{2}_median=0.057697,brlenIlnBrlens{2}_95%HPD={0.000003,0.264498},rateIlnBrlens{2}_mean=0.893704,rateIlnBrlens{2}_median=0.776002,rateIlnBrlens{2}_95%HPD={0.018008,2.063062},brlenIlnBrlens{3}_mean=0.134321,brlenIlnBrlens{3}_median=0.077919,brlenIlnBrlens{3}_95%HPD={0.000000,0.452928},rateIlnBrlens{3}_mean=1.565500,rateIlnBrlens{3}_median=0.893952,rateIlnBrlens{3}_95%HPD={0.004699,5.267339},brlenIlnBrlens{4,5}_mean=0.084738,brlenIlnBrlens{4,5}_median=0.058152,brlenIlnBrlens{4,5}_95%HPD={0.000001,0.252594},rateIlnBrlens{4,5}_mean=0.882742,rateIlnBrlens{4,5}_median=0.736353,rateIlnBrlens{4,5}_95%HPD={0.048895,2.045603}],39[&prob=1.00000000e+00,prob_stddev=0.00000000e+00,prob_range={1.00000000e+00,1.00000000e+00},prob(percent)="100",prob+-sd="100+-0",height_mean=4.28348468e-01,height_median=4.12034124e-01,height_95%HPD={2.43535437e-01,6.49287490e-01},age_mean=1.25000020e+02,age_median=1.25000018e+02,age_95%HPD={1.24999976e+02,1.25000069e+02}]:5.709280e+01[&length_mean=1.86088915e-01,length_median=1.67201800e-01,length_95%HPD={2.05726800e-02,3.95857600e-01},brlenIlnBrlens{1}_mean=0.200352,brlenIlnBrlens{1}_median=0.156744,brlenIlnBrlens{1}_95%HPD={0.000000,0.515630},rateIlnBrlens{1}_mean=1.236310,rateIlnBrlens{1}_median=0.964577,rateIlnBrlens{1}_95%HPD={0.044798,3.136425},brlenIlnBrlens{2}_mean=0.132821,brlenIlnBrlens{2}_median=0.103643,brlenIlnBrlens{2}_95%HPD={0.000000,0.346676},rateIlnBrlens{2}_mean=0.752247,rateIlnBrlens{2}_median=0.686798,rateIlnBrlens{2}_95%HPD={0.021740,1.605301},brlenIlnBrlens{3}_mean=0.153976,brlenIlnBrlens{3}_median=0.114411,brlenIlnBrlens{3}_95%HPD={0.000000,0.419048},rateIlnBrlens{3}_mean=1.156336,rateIlnBrlens{3}_median=0.755180,rateIlnBrlens{3}_95%HPD={0.003942,3.385178},brlenIlnBrlens{4,5}_mean=0.182512,brlenIlnBrlens{4,5}_median=0.126922,brlenIlnBrlens{4,5}_95%HPD={0.000000,0.513518},rateIlnBrlens{4,5}_mean=0.983229,rateIlnBrlens{4,5}_median=0.797889,rateIlnBrlens{4,5}_95%HPD={0.047115,2.333228}])[&prob=9.99511120e-01,prob_stddev=2.38840711e-04,prob_range={9.99342234e-01,9.99680006e-01},prob(percent)="100",prob+-sd="100+-0",height_mean=6.40543060e-01,height_median=6.13132130e-01,height_95%HPD={3.49965614e-01,9.82423510e-01},age_mean=1.87754362e+02,age_median=1.82092818e+02,age_95%HPD={1.62999947e+02,2.36122921e+02}]:1.379072e+02[&length_mean=5.26697021e-01,length_median=5.02194400e-01,length_95%HPD={2.08518500e-01,9.09238800e-01},brlenIlnBrlens{1}_mean=0.667000,brlenIlnBrlens{1}_median=0.529365,brlenIlnBrlens{1}_95%HPD={0.030163,1.660986},rateIlnBrlens{1}_mean=1.321629,rateIlnBrlens{1}_median=1.065889,rateIlnBrlens{1}_95%HPD={0.091330,3.266960},brlenIlnBrlens{2}_mean=0.442723,brlenIlnBrlens{2}_median=0.371229,brlenIlnBrlens{2}_95%HPD={0.017388,1.032473},rateIlnBrlens{2}_mean=0.855272,rateIlnBrlens{2}_median=0.758294,rateIlnBrlens{2}_95%HPD={0.048733,1.832860},brlenIlnBrlens{3}_mean=0.256518,brlenIlnBrlens{3}_median=0.175622,brlenIlnBrlens{3}_95%HPD={0.000220,0.755430},rateIlnBrlens{3}_mean=0.498627,rateIlnBrlens{3}_median=0.345561,rateIlnBrlens{3}_95%HPD={0.000790,1.409013},brlenIlnBrlens{4,5}_mean=0.308375,brlenIlnBrlens{4,5}_median=0.272680,brlenIlnBrlens{4,5}_95%HPD={0.028864,0.659259},rateIlnBrlens{4,5}_mean=0.622238,rateIlnBrlens{4,5}_median=0.554244,rateIlnBrlens{4,5}_95%HPD={0.059668,1.328875}],44[&prob=1.00000000e+00,prob_stddev=0.00000000e+00,prob_range={1.00000000e+00,1.00000000e+00},prob(percent)="100",prob+-sd="100+-0",height_mean=1.09657197e+00,height_median=1.05480728e+00,height_95%HPD={6.23450677e-01,1.66217595e+00},age_mean=3.20000020e+02,age_median=3.20000019e+02,age_95%HPD={3.19999954e+02,3.20000090e+02}]:2.053352e-05[&length_mean=7.45427270e-02,length_median=3.33609400e-02,length_95%HPD={0.00000000e+00,2.63246800e-01},brlenIlnBrlens{1}_mean=0.120905,brlenIlnBrlens{1}_median=0.028960,brlenIlnBrlens{1}_95%HPD={0.000000,0.501785},rateIlnBrlens{1}_mean=1.423786,rateIlnBrlens{1}_median=1.000000,rateIlnBrlens{1}_95%HPD={0.063836,3.559619},brlenIlnBrlens{2}_mean=0.052479,brlenIlnBrlens{2}_median=0.014013,brlenIlnBrlens{2}_95%HPD={0.000000,0.217393},rateIlnBrlens{2}_mean=0.844960,rateIlnBrlens{2}_median=1.000000,rateIlnBrlens{2}_95%HPD={0.037000,1.270110},brlenIlnBrlens{3}_mean=0.039951,brlenIlnBrlens{3}_median=0.004793,brlenIlnBrlens{3}_95%HPD={0.000000,0.185964},rateIlnBrlens{3}_mean=0.746224,rateIlnBrlens{3}_median=1.000000,rateIlnBrlens{3}_95%HPD={0.003485,1.321463},brlenIlnBrlens{4,5}_mean=0.066338,brlenIlnBrlens{4,5}_median=0.017167,brlenIlnBrlens{4,5}_95%HPD={0.000000,0.271301},rateIlnBrlens{4,5}_mean=0.927878,rateIlnBrlens{4,5}_median=1.000000,rateIlnBrlens{4,5}_95%HPD={0.088573,1.644038}])[&prob=6.01909299e-01,prob_stddev=1.22185880e-02,prob_range={5.93269453e-01,6.10549146e-01},prob(percent)="60",prob+-sd="60+-1",height_mean=1.11718472e+00,height_median=1.07310971e+00,height_95%HPD={6.28799821e-01,1.69515807e+00},age_mean=3.26745698e+02,age_median=3.20000040e+02,age_95%HPD={3.19999884e+02,3.59044475e+02}]:3.403139e+01[&length_mean=1.36998707e-01,length_median=1.23488300e-01,length_95%HPD={7.39707200e-04,2.91765100e-01},brlenIlnBrlens{1}_mean=0.322237,brlenIlnBrlens{1}_median=0.227247,brlenIlnBrlens{1}_95%HPD={0.000015,0.928051},rateIlnBrlens{1}_mean=2.646398,rateIlnBrlens{1}_median=1.870348,rateIlnBrlens{1}_95%HPD={0.018250,7.247921},brlenIlnBrlens{2}_mean=0.117599,brlenIlnBrlens{2}_median=0.083811,brlenIlnBrlens{2}_95%HPD={0.000009,0.329773},rateIlnBrlens{2}_mean=0.866260,rateIlnBrlens{2}_median=0.759628,rateIlnBrlens{2}_95%HPD={0.022797,1.946380},brlenIlnBrlens{3}_mean=0.104665,brlenIlnBrlens{3}_median=0.052403,brlenIlnBrlens{3}_95%HPD={0.000002,0.373621},rateIlnBrlens{3}_mean=0.792403,rateIlnBrlens{3}_median=0.459718,rateIlnBrlens{3}_95%HPD={0.002744,2.529670},brlenIlnBrlens{4,5}_mean=0.112380,brlenIlnBrlens{4,5}_median=0.083975,brlenIlnBrlens{4,5}_95%HPD={0.000006,0.307682},rateIlnBrlens{4,5}_mean=0.865132,rateIlnBrlens{4,5}_median=0.728137,rateIlnBrlens{4,5}_95%HPD={0.060679,1.985838}],43[&prob=1.00000000e+00,prob_stddev=0.00000000e+00,prob_range={1.00000000e+00,1.00000000e+00},prob(percent)="100",prob+-sd="100+-0",height_mean=1.06230410e+00,height_median=1.02184458e+00,height_95%HPD={6.03967849e-01,1.61023292e+00},age_mean=3.10000020e+02,age_median=3.10000019e+02,age_95%HPD={3.09999953e+02,3.10000090e+02}]:4.403141e+01[&length_mean=1.77843884e-01,length_median=1.64770400e-01,length_95%HPD={0.00000000e+00,3.48033400e-01},brlenIlnBrlens{1}_mean=0.160947,brlenIlnBrlens{1}_median=0.119096,brlenIlnBrlens{1}_95%HPD={0.000000,0.438937},rateIlnBrlens{1}_mean=1.043565,rateIlnBrlens{1}_median=0.752101,rateIlnBrlens{1}_95%HPD={0.020501,2.719252},brlenIlnBrlens{2}_mean=0.120953,brlenIlnBrlens{2}_median=0.094358,brlenIlnBrlens{2}_95%HPD={0.000000,0.316284},rateIlnBrlens{2}_mean=0.704088,rateIlnBrlens{2}_median=0.646388,rateIlnBrlens{2}_95%HPD={0.028005,1.476762},brlenIlnBrlens{3}_mean=0.131480,brlenIlnBrlens{3}_median=0.074423,brlenIlnBrlens{3}_95%HPD={0.000000,0.436874},rateIlnBrlens{3}_mean=0.807253,rateIlnBrlens{3}_median=0.481679,rateIlnBrlens{3}_95%HPD={0.004777,2.573317},brlenIlnBrlens{4,5}_mean=0.172177,brlenIlnBrlens{4,5}_median=0.120797,brlenIlnBrlens{4,5}_95%HPD={0.000000,0.483768},rateIlnBrlens{4,5}_mean=0.966862,rateIlnBrlens{4,5}_median=0.796806,rateIlnBrlens{4,5}_95%HPD={0.051933,2.297001}])[&prob=3.68366785e-01,prob_stddev=9.52848732e-03,prob_range={3.61629127e-01,3.75104443e-01},prob(percent)="37",prob+-sd="37+-1",height_mean=1.22444211e+00,height_median=1.17707197e+00,height_95%HPD={6.91637551e-01,1.84823467e+00},age_mean=3.56716633e+02,age_median=3.54031434e+02,age_95%HPD={3.19999940e+02,3.93265525e+02}]:1.615456e+01[&length_mean=8.81409750e-02,length_median=7.42816800e-02,length_95%HPD={3.74342900e-06,2.16978600e-01},brlenIlnBrlens{1}_mean=0.101698,brlenIlnBrlens{1}_median=0.052053,brlenIlnBrlens{1}_95%HPD={0.000003,0.357566},rateIlnBrlens{1}_mean=1.215173,rateIlnBrlens{1}_median=0.760371,rateIlnBrlens{1}_95%HPD={0.011583,3.766137},brlenIlnBrlens{2}_mean=0.072414,brlenIlnBrlens{2}_median=0.049466,brlenIlnBrlens{2}_95%HPD={0.000001,0.213789},rateIlnBrlens{2}_mean=0.866169,rateIlnBrlens{2}_median=0.776795,rateIlnBrlens{2}_95%HPD={0.012906,1.921451},brlenIlnBrlens{3}_mean=0.066697,brlenIlnBrlens{3}_median=0.027318,brlenIlnBrlens{3}_95%HPD={0.000005,0.257528},rateIlnBrlens{3}_mean=0.817921,rateIlnBrlens{3}_median=0.417874,rateIlnBrlens{3}_95%HPD={0.002285,2.861115},brlenIlnBrlens{4,5}_mean=0.071986,brlenIlnBrlens{4,5}_median=0.049820,brlenIlnBrlens{4,5}_95%HPD={0.000003,0.212770},rateIlnBrlens{4,5}_mean=0.873545,rateIlnBrlens{4,5}_median=0.726080,rateIlnBrlens{4,5}_95%HPD={0.054116,2.069454}],45[&prob=1.00000000e+00,prob_stddev=0.00000000e+00,prob_range={1.00000000e+00,1.00000000e+00},prob(percent)="100",prob+-sd="100+-0",height_mean=1.06230410e+00,height_median=1.02184450e+00,height_95%HPD={6.03967847e-01,1.61023295e+00},age_mean=3.10000020e+02,age_median=3.10000019e+02,age_95%HPD={3.09999954e+02,3.10000092e+02}]:6.018598e+01[&length_mean=2.45660255e-01,length_median=2.28612000e-01,length_95%HPD={5.39030500e-02,4.77372200e-01},brlenIlnBrlens{1}_mean=0.127508,brlenIlnBrlens{1}_median=0.090744,brlenIlnBrlens{1}_95%HPD={0.000000,0.362095},rateIlnBrlens{1}_mean=0.565172,rateIlnBrlens{1}_median=0.415862,rateIlnBrlens{1}_95%HPD={0.005161,1.522600},brlenIlnBrlens{2}_mean=0.329010,brlenIlnBrlens{2}_median=0.250918,brlenIlnBrlens{2}_95%HPD={0.000000,0.824955},rateIlnBrlens{2}_mean=1.400054,rateIlnBrlens{2}_median=1.085840,rateIlnBrlens{2}_95%HPD={0.112428,3.362673},brlenIlnBrlens{3}_mean=0.608521,brlenIlnBrlens{3}_median=0.344505,brlenIlnBrlens{3}_95%HPD={0.000000,1.954569},rateIlnBrlens{3}_mean=2.576472,rateIlnBrlens{3}_median=1.584510,rateIlnBrlens{3}_95%HPD={0.011980,7.935437},brlenIlnBrlens{4,5}_mean=0.248044,brlenIlnBrlens{4,5}_median=0.173214,brlenIlnBrlens{4,5}_95%HPD={0.000000,0.680123},rateIlnBrlens{4,5}_mean=1.004115,rateIlnBrlens{4,5}_median=0.802257,rateIlnBrlens{4,5}_95%HPD={0.047403,2.400428}])[&prob=3.12901104e-01,prob_stddev=1.71211078e-02,prob_range={3.00794653e-01,3.25007555e-01},prob(percent)="31",prob+-sd="31+-2",height_mean=1.29971708e+00,height_median=1.25584864e+00,height_95%HPD={7.53522020e-01,1.93250180e+00},age_mean=3.71140701e+02,age_median=3.70185996e+02,age_95%HPD={3.32094667e+02,4.11944423e+02}]:2.753202e+01[&length_mean=6.65146262e-02,length_median=5.42551600e-02,length_95%HPD={3.89540600e-06,1.72951200e-01},brlenIlnBrlens{1}_mean=0.064409,brlenIlnBrlens{1}_median=0.033171,brlenIlnBrlens{1}_95%HPD={0.000001,0.231979},rateIlnBrlens{1}_mean=1.110049,rateIlnBrlens{1}_median=0.641727,rateIlnBrlens{1}_95%HPD={0.004127,3.662021},brlenIlnBrlens{2}_mean=0.060665,brlenIlnBrlens{2}_median=0.040185,brlenIlnBrlens{2}_95%HPD={0.000001,0.186286},rateIlnBrlens{2}_mean=0.959179,rateIlnBrlens{2}_median=0.830130,rateIlnBrlens{2}_95%HPD={0.019566,2.194223},brlenIlnBrlens{3}_mean=0.057838,brlenIlnBrlens{3}_median=0.021363,brlenIlnBrlens{3}_95%HPD={0.000001,0.227819},rateIlnBrlens{3}_mean=0.909118,rateIlnBrlens{3}_median=0.444432,rateIlnBrlens{3}_95%HPD={0.004491,3.317248},brlenIlnBrlens{4,5}_mean=0.055811,brlenIlnBrlens{4,5}_median=0.037506,brlenIlnBrlens{4,5}_95%HPD={0.000001,0.171652},rateIlnBrlens{4,5}_mean=0.900549,rateIlnBrlens{4,5}_median=0.740369,rateIlnBrlens{4,5}_95%HPD={0.044223,2.091144}],42[&prob=1.00000000e+00,prob_stddev=0.00000000e+00,prob_range={1.00000000e+00,1.00000000e+00},prob(percent)="100",prob+-sd="100+-0",height_mean=1.23364346e+00,height_median=1.18665817e+00,height_95%HPD={7.01382099e-01,1.86994794e+00},age_mean=3.60000020e+02,age_median=3.60000019e+02,age_95%HPD={3.59999949e+02,3.60000095e+02}]:3.771799e+01[&length_mean=7.49712398e-02,length_median=6.59333000e-02,length_95%HPD={0.00000000e+00,2.05530700e-01},brlenIlnBrlens{1}_mean=0.119773,brlenIlnBrlens{1}_median=0.075824,brlenIlnBrlens{1}_95%HPD={0.000000,0.402336},rateIlnBrlens{1}_mean=1.574361,rateIlnBrlens{1}_median=1.000000,rateIlnBrlens{1}_95%HPD={0.074081,4.252449},brlenIlnBrlens{2}_mean=0.067899,brlenIlnBrlens{2}_median=0.046086,brlenIlnBrlens{2}_95%HPD={0.000000,0.218153},rateIlnBrlens{2}_mean=0.953678,rateIlnBrlens{2}_median=1.000000,rateIlnBrlens{2}_95%HPD={0.045420,1.803833},brlenIlnBrlens{3}_mean=0.052737,brlenIlnBrlens{3}_median=0.017416,brlenIlnBrlens{3}_95%HPD={0.000000,0.220760},rateIlnBrlens{3}_mean=0.808626,rateIlnBrlens{3}_median=0.693173,rateIlnBrlens{3}_95%HPD={0.000819,2.131984},brlenIlnBrlens{4,5}_mean=0.068442,brlenIlnBrlens{4,5}_median=0.041251,brlenIlnBrlens{4,5}_95%HPD={0.000000,0.230474},rateIlnBrlens{4,5}_mean=0.932309,rateIlnBrlens{4,5}_median=1.000000,rateIlnBrlens{4,5}_95%HPD={0.082121,1.882717}])[&prob=9.19681428e-01,prob_stddev=1.91072569e-03,prob_range={9.18330341e-01,9.21032515e-01},prob(percent)="92",prob+-sd="92+-0",height_mean=1.36338716e+00,height_median=1.31321210e+00,height_95%HPD={7.84616829e-01,2.03801939e+00},age_mean=4.00495189e+02,age_median=3.97718013e+02,age_95%HPD={3.59999925e+02,4.44326122e+02}]:5.925636e+01[&length_mean=1.92011257e-01,length_median=1.68709800e-01,length_95%HPD={1.97394400e-02,4.20041200e-01},brlenIlnBrlens{1}_mean=0.160615,brlenIlnBrlens{1}_median=0.089461,brlenIlnBrlens{1}_95%HPD={0.000032,0.542978},rateIlnBrlens{1}_mean=0.824138,rateIlnBrlens{1}_median=0.557824,rateIlnBrlens{1}_95%HPD={0.007486,2.385998},brlenIlnBrlens{2}_mean=0.168270,brlenIlnBrlens{2}_median=0.120684,brlenIlnBrlens{2}_95%HPD={0.000647,0.470972},rateIlnBrlens{2}_mean=0.893887,rateIlnBrlens{2}_median=0.786244,rateIlnBrlens{2}_95%HPD={0.022193,2.015261},brlenIlnBrlens{3}_mean=2.565068,brlenIlnBrlens{3}_median=1.784931,brlenIlnBrlens{3}_95%HPD={0.000036,7.124911},rateIlnBrlens{3}_mean=16.142819,rateIlnBrlens{3}_median=10.883793,rateIlnBrlens{3}_95%HPD={0.004266,45.066291},brlenIlnBrlens{4,5}_mean=0.140708,brlenIlnBrlens{4,5}_median=0.109750,brlenIlnBrlens{4,5}_95%HPD={0.002464,0.364418},rateIlnBrlens{4,5}_mean=0.794956,rateIlnBrlens{4,5}_median=0.675402,rateIlnBrlens{4,5}_95%HPD={0.045638,1.806245}])[&prob=3.32011875e-01,prob_stddev=1.67188498e-02,prob_range={3.20189863e-01,3.43833887e-01},prob(percent)="33",prob+-sd="33+-2",height_mean=1.61184564e+00,height_median=1.55107803e+00,height_95%HPD={9.67479704e-01,2.40285975e+00},age_mean=4.62514685e+02,age_median=4.56974373e+02,age_95%HPD={3.94405733e+02,5.42837413e+02}]:4.265817e+01[&length_mean=1.97073204e-01,length_median=1.61798100e-01,length_95%HPD={1.76928500e-05,4.90709200e-01},brlenIlnBrlens{1}_mean=0.228000,brlenIlnBrlens{1}_median=0.145141,brlenIlnBrlens{1}_95%HPD={0.000003,0.722872},rateIlnBrlens{1}_mean=1.280756,rateIlnBrlens{1}_median=0.890867,rateIlnBrlens{1}_95%HPD={0.006472,3.575287},brlenIlnBrlens{2}_mean=0.183595,brlenIlnBrlens{2}_median=0.124169,brlenIlnBrlens{2}_95%HPD={0.000017,0.537772},rateIlnBrlens{2}_mean=0.969797,rateIlnBrlens{2}_median=0.846236,rateIlnBrlens{2}_95%HPD={0.018920,2.146193},brlenIlnBrlens{3}_mean=0.174048,brlenIlnBrlens{3}_median=0.062761,brlenIlnBrlens{3}_95%HPD={0.000003,0.698822},rateIlnBrlens{3}_mean=0.943206,rateIlnBrlens{3}_median=0.414427,rateIlnBrlens{3}_95%HPD={0.003881,3.538170},brlenIlnBrlens{4,5}_mean=0.194273,brlenIlnBrlens{4,5}_median=0.120894,brlenIlnBrlens{4,5}_95%HPD={0.000012,0.599459},rateIlnBrlens{4,5}_mean=0.987854,rateIlnBrlens{4,5}_median=0.808446,rateIlnBrlens{4,5}_95%HPD={0.032396,2.294149}])[&prob=6.68281453e-01,prob_stddev=2.69387181e-02,prob_range={6.49232903e-01,6.87330003e-01},prob(percent)="67",prob+-sd="67+-3",height_mean=1.73612703e+00,height_median=1.67290109e+00,height_95%HPD={9.99872600e-01,2.56930560e+00},age_mean=5.03694970e+02,age_median=4.99632545e+02,age_95%HPD={4.41032416e+02,5.74287625e+02}]:6.226742e+01[&length_mean=2.02210248e-01,length_median=1.81163000e-01,length_95%HPD={3.32388300e-04,4.38150400e-01},brlenIlnBrlens{1}_mean=0.235371,brlenIlnBrlens{1}_median=0.159083,brlenIlnBrlens{1}_95%HPD={0.000004,0.699315},rateIlnBrlens{1}_mean=1.289316,rateIlnBrlens{1}_median=0.902288,rateIlnBrlens{1}_95%HPD={0.024048,3.641030},brlenIlnBrlens{2}_mean=0.216842,brlenIlnBrlens{2}_median=0.146034,brlenIlnBrlens{2}_95%HPD={0.000009,0.633049},rateIlnBrlens{2}_mean=1.083147,rateIlnBrlens{2}_median=0.872407,rateIlnBrlens{2}_95%HPD={0.030332,2.675748},brlenIlnBrlens{3}_mean=0.165526,brlenIlnBrlens{3}_median=0.071567,brlenIlnBrlens{3}_95%HPD={0.000001,0.615080},rateIlnBrlens{3}_mean=0.880074,rateIlnBrlens{3}_median=0.426057,rateIlnBrlens{3}_95%HPD={0.002938,3.109150},brlenIlnBrlens{4,5}_mean=0.197694,brlenIlnBrlens{4,5}_median=0.133606,brlenIlnBrlens{4,5}_95%HPD={0.000009,0.579727},rateIlnBrlens{4,5}_mean=0.984171,rateIlnBrlens{4,5}_median=0.791901,rateIlnBrlens{4,5}_95%HPD={0.047016,2.378194}])[&prob=1.00000000e+00,prob_stddev=0.00000000e+00,prob_range={1.00000000e+00,1.00000000e+00},prob(percent)="100",prob+-sd="100+-0",height_mean=1.93007243e+00,height_median=1.85806017e+00,height_95%HPD={1.12192998e+00,2.89569455e+00},age_mean=5.65545650e+02,age_median=5.61899966e+02,age_95%HPD={4.96457013e+02,6.42509688e+02}]:3.673830e+01[&length_mean=1.35869550e-01,length_median=1.12085600e-01,length_95%HPD={2.49087300e-06,3.42751200e-01},brlenIlnBrlens{1}_mean=0.265063,brlenIlnBrlens{1}_median=0.113705,brlenIlnBrlens{1}_95%HPD={0.000001,1.010754},rateIlnBrlens{1}_mean=1.988662,rateIlnBrlens{1}_median=1.038321,rateIlnBrlens{1}_95%HPD={0.007150,6.697109},brlenIlnBrlens{2}_mean=0.134534,brlenIlnBrlens{2}_median=0.082377,brlenIlnBrlens{2}_95%HPD={0.000002,0.431044},rateIlnBrlens{2}_mean=0.997580,rateIlnBrlens{2}_median=0.840718,rateIlnBrlens{2}_95%HPD={0.024101,2.383866},brlenIlnBrlens{3}_mean=0.103745,brlenIlnBrlens{3}_median=0.040744,brlenIlnBrlens{3}_95%HPD={0.000000,0.405052},rateIlnBrlens{3}_mean=0.807723,rateIlnBrlens{3}_median=0.413399,rateIlnBrlens{3}_95%HPD={0.001055,2.784864},brlenIlnBrlens{4,5}_mean=0.137068,brlenIlnBrlens{4,5}_median=0.082518,brlenIlnBrlens{4,5}_95%HPD={0.000001,0.441700},rateIlnBrlens{4,5}_mean=1.011041,rateIlnBrlens{4,5}_median=0.811378,rateIlnBrlens{4,5}_95%HPD={0.051910,2.477158}])[&prob=1.00000000e+00,prob_stddev=0.00000000e+00,prob_range={1.00000000e+00,1.00000000e+00},prob(percent)="100",prob+-sd="100+-0",height_mean=2.06594198e+00,height_median=1.99041780e+00,height_95%HPD={1.17017576e+00,3.09243002e+00},age_mean=6.04922016e+02,age_median=5.98638267e+02,age_95%HPD={5.35553387e+02,6.80241385e+02}][&length_mean=0.00000000e+00,length_median=0.00000000e+00,length_95%HPD={0.00000000e+00,0.00000000e+00},brlenIlnBrlens{1}_mean=1.000000,brlenIlnBrlens{1}_median=1.000000,brlenIlnBrlens{1}_95%HPD={1.000000,1.000000},rateIlnBrlens{1}_mean=1.000000,rateIlnBrlens{1}_median=1.000000,rateIlnBrlens{1}_95%HPD={1.000000,1.000000},brlenIlnBrlens{2}_mean=1.000000,brlenIlnBrlens{2}_median=1.000000,brlenIlnBrlens{2}_95%HPD={1.000000,1.000000},rateIlnBrlens{2}_mean=1.000000,rateIlnBrlens{2}_median=1.000000,rateIlnBrlens{2}_95%HPD={1.000000,1.000000},brlenIlnBrlens{3}_mean=1.000000,brlenIlnBrlens{3}_median=1.000000,brlenIlnBrlens{3}_95%HPD={1.000000,1.000000},rateIlnBrlens{3}_mean=1.000000,rateIlnBrlens{3}_median=1.000000,rateIlnBrlens{3}_95%HPD={1.000000,1.000000},brlenIlnBrlens{4,5}_mean=1.000000,brlenIlnBrlens{4,5}_median=1.000000,brlenIlnBrlens{4,5}_95%HPD={1.000000,1.000000},rateIlnBrlens{4,5}_mean=1.000000,rateIlnBrlens{4,5}_median=1.000000,rateIlnBrlens{4,5}_95%HPD={1.000000,1.000000}];end; 
