# Supplementary material for: The rise of predation in Jurassic lampreys
Source: Nat Commun. 2023 Oct 31;14:6652. doi: 10.1038/s41467-023-42251-0 (PMC10618186; doi:10.1038/s41467-023-42251-0)
Supplement: Supplementary file 4 — Supplementary Code 1-8 [file 41467_2023_42251_MOESM4_ESM.zip › Supplementary Codes 1-8/Supplementary Code 4.rtf]

Supplementary Code 4: Ancestral state reconstruction                              MrBayes 3.2.7b x86_64                       (Bayesian Analysis of Phylogeny)                              (Parallel version)                         (8 processors available)               Distributed under the GNU General Public License                 Type "help" or "help <command>" for information                     on the commands that are available.                    Type "about" for authorship and general                       information about the program.      Executing file "run.nex"   UNIX line termination   Longest line length = 134   Parsing file   Expecting NEXUS formatted file   Reading mrbayes block       Executing file "../data/morph_molec.nex"...    UNIX line termination   Longest line length = 801   Parsing file   Expecting NEXUS formatted file   Reading data block      Allocated taxon set      Allocated matrix      Defining new matrix with 45 taxa and 1701 characters      Data is Mixed         Data for partition 1 is Standard         Data for partition 2 is Dna      There are a total of 2 default data divisions      Data matrix is interleaved      Gaps coded as -      Missing data coded as ?      Taxon  1 -> Euconodonta      Taxon  2 -> Jamoytius      Taxon  3 -> Euphanerops      Taxon  4 -> Achanarella      Taxon  5 -> Ciderius      Taxon  6 -> Cornovichthys      Taxon  7 -> Lasanius      Taxon  8 -> Birkenia      Taxon  9 -> Rhyncholepis      Taxon 10 -> Myxinikela      Taxon 11 -> Tethymyxine      Taxon 12 -> Paramyxine_fernholmi      Taxon 13 -> Eptatretus_burgeri      Taxon 14 -> Eptatretus_stoutii      Taxon 15 -> Myxine_glutinosa      Taxon 16 -> Rubicundus_eos      Taxon 17 -> Rubicundus_lopheliae      Taxon 18 -> Neomyxine_biniplicata      Taxon 19 -> Myxineidus      Taxon 20 -> Gilpichthys      Taxon 21 -> Lethenteron_camtschaticum      Taxon 22 -> Petromyzon_marinus      Taxon 23 -> Lampetra_fluviatilis      Taxon 24 -> Geotria_australis      Taxon 25 -> Ichthyomyzon_bdellium      Taxon 26 -> Ichthyomyzon_castaneus      Taxon 27 -> Ichthyomyzon_unicuspis      Taxon 28 -> Mordacia_mordax      Taxon 29 -> Mordacia_lapicida      Taxon 30 -> Caspiomyzon_wagneri      Taxon 31 -> Tetrapleurodon_spadiceus      Taxon 32 -> Entosphenus_macrostomus      Taxon 33 -> Entosphenus_minimus      Taxon 34 -> Entosphenus_similis      Taxon 35 -> Entosphenus_tridentatus      Taxon 36 -> Eudontomyzon_danfordi      Taxon 37 -> Eudontomyzon_morii      Taxon 38 -> Lampetra_ayresii      Taxon 39 -> Mesomyzon      Taxon 40 -> Yanliaomyzon_ingensdentes      Taxon 41 -> Yanliaomyzon_occisor      Taxon 42 -> Priscomyzon      Taxon 43 -> Mayomyzon      Taxon 44 -> Hardistiella      Taxon 45 -> Pipiscius      Successfully read matrix      Setting default partition, dividing characters into 2 parts      Setting model defaults      Seed (for generating default start values) = 1648603866      WARNING: There are 76 characters incompatible with the specified               coding bias. These characters will be excluded.      Setting output file names to "../data/morph_molec.nex.run<i>.<p|t>"   Exiting data block   Reached end of file    Returning execution to calling file ...       Defining charset called 'MVothers'      Defining charset called 'BranchialApparatus'      Defining charset called 'FeedingMechanism'      Defining charset called '16S'      Defining charset called 'CO1'      Defining partition called 'five'      Setting five as the partition, dividing characters into 5 parts.      Setting model defaults      Seed (for generating default start values) = 1158291438      WARNING: There are 61 characters incompatible with the specified               coding bias. These characters will be excluded.      WARNING: There are 10 characters incompatible with the specified               coding bias. These characters will be excluded.      WARNING: There are 5 characters incompatible with the specified               coding bias. These characters will be excluded.      Excluding character(s)      Enabling Coding Variable for partition 1      Enabling Coding Variable for partition 2      Enabling Coding Variable for partition 3      Setting Rates to Gamma for partition 1      Setting Rates to Gamma for partition 2      Setting Rates to Gamma for partition 3      Successfully set likelihood model parameters to      partitions 1, 2, and 3 (if applicable)      Setting Nst to 2 for partition 4      Setting Nst to 2 for partition 5      Setting Rates to Gamma for partition 4      Setting Rates to Gamma for partition 5      Successfully set likelihood model parameters to      partitions 4 and 5 (if applicable)      Unlinking      Setting Ratepr to Variable [Dirichlet(..,1,..)] for partition 1      Setting Ratepr to Variable [Dirichlet(..,1,..)] for partition 2      Setting Ratepr to Variable [Dirichlet(..,1,..)] for partition 3      Setting Ratepr to Variable [Dirichlet(..,1,..)] for partition 4      Setting Ratepr to Variable [Dirichlet(..,1,..)] for partition 5      Successfully set prior model parameters to all      applicable data partitions       Defining constraint called 'ingroup'      Defining constraint called 'crown_lamprey'      Defining constraint called 'north_lamprey'      Setting Clockratepr to Lognormal(-6.00,1.00)      Setting Clockratepr to Lognormal(-6.00,1.00)      Setting Clockratepr to Lognormal(-6.00,1.00)      Setting Clockratepr to Lognormal(-6.00,1.00)      Setting Clockratepr to Lognormal(-6.00,1.00)      Successfully set prior model parameters to all      applicable data partitions       Setting Clockvarpr to ILN for partition 1      Setting Clockvarpr to ILN for partition 2      Setting Clockvarpr to ILN for partition 3      Setting Clockvarpr to ILN for partition 4      Setting Clockvarpr to ILN for partition 5      Successfully set prior model parameters to all      applicable data partitions       Unlinking      Setting age of taxon 'Euconodonta' to Fixed(535.50)      Setting age of taxon 'Jamoytius' to Fixed(435.40)      Setting age of taxon 'Euphanerops' to Fixed(370.00)      Setting age of taxon 'Achanarella' to Fixed(385.00)      Setting age of taxon 'Ciderius' to Fixed(432.00)      Setting age of taxon 'Cornovichthys' to Fixed(385.00)      Setting age of taxon 'Lasanius' to Fixed(428.20)      Setting age of taxon 'Birkenia' to Fixed(435.40)      Setting age of taxon 'Rhyncholepis' to Fixed(431.95)      Setting age of taxon 'Myxinikela' to Fixed(280.00)      Setting age of taxon 'Tethymyxine' to Fixed(95.00)      Setting age of taxon 'Myxineidus' to Fixed(280.00)      Setting age of taxon 'Gilpichthys' to Fixed(280.00)      Setting age of taxon 'Mesomyzon' to Fixed(125.00)      Setting age of taxon 'Yanliaomyzon_occisor' to Fixed(158.00)      Setting age of taxon 'Yanliaomyzon_ingensdentes' to Fixed(163.00)      Setting age of taxon 'Priscomyzon' to Fixed(360.00)      Setting age of taxon 'Mayomyzon' to Fixed(280.00)      Setting age of taxon 'Hardistiella' to Fixed(320.00)      Setting age of taxon 'Pipiscius' to Fixed(280.00)      Setting Nodeagepr to Calibrated for partition 1      Setting Nodeagepr to Calibrated for partition 2      Setting Nodeagepr to Calibrated for partition 3      Setting Nodeagepr to Calibrated for partition 4      Setting Nodeagepr to Calibrated for partition 5      Successfully set prior model parameters to all      applicable data partitions       Setting Brlenspr to Clock:Fossilization for partition 1      Setting Brlenspr to Clock:Fossilization for partition 2      Setting Brlenspr to Clock:Fossilization for partition 3      Setting Brlenspr to Clock:Fossilization for partition 4      Setting Brlenspr to Clock:Fossilization for partition 5      Successfully set prior model parameters to all      applicable data partitions       Setting SampleStrat to Random for partition 1      Setting SampleStrat to Random for partition 2      Setting SampleStrat to Random for partition 3      Setting SampleStrat to Random for partition 4      Setting SampleStrat to Random for partition 5      Successfully set prior model parameters to all      applicable data partitions       Setting Sampleprob to 0.30000000 for partition 1      Setting Sampleprob to 0.30000000 for partition 2      Setting Sampleprob to 0.30000000 for partition 3      Setting Sampleprob to 0.30000000 for partition 4      Setting Sampleprob to 0.30000000 for partition 5      Successfully set prior model parameters to all      applicable data partitions       Setting Speciationpr to Exponential(100.00) for partition 1      Setting Speciationpr to Exponential(100.00) for partition 2      Setting Speciationpr to Exponential(100.00) for partition 3      Setting Speciationpr to Exponential(100.00) for partition 4      Setting Speciationpr to Exponential(100.00) for partition 5      Successfully set prior model parameters to all      applicable data partitions       Setting Extinctionpr to Beta(2.00,1.00) for partition 1      Setting Extinctionpr to Beta(2.00,1.00) for partition 2      Setting Extinctionpr to Beta(2.00,1.00) for partition 3      Setting Extinctionpr to Beta(2.00,1.00) for partition 4      Setting Extinctionpr to Beta(2.00,1.00) for partition 5      Successfully set prior model parameters to all      applicable data partitions       Setting Fossilizationpr to Beta(1.00,9.00) for partition 1      Setting Fossilizationpr to Beta(1.00,9.00) for partition 2      Setting Fossilizationpr to Beta(1.00,9.00) for partition 3      Setting Fossilizationpr to Beta(1.00,9.00) for partition 4      Setting Fossilizationpr to Beta(1.00,9.00) for partition 5      Successfully set prior model parameters to all      applicable data partitions       Setting Treeagepr to Offsetexponential(500.00,600.00)      Setting Treeagepr to Offsetexponential(500.00,600.00)      Setting Treeagepr to Offsetexponential(500.00,600.00)      Setting Treeagepr to Offsetexponential(500.00,600.00)      Setting Treeagepr to Offsetexponential(500.00,600.00)      Successfully set prior model parameters to all      applicable data partitions       Setting Topologypr to Constraints for partition 1      Setting Topologypr to Constraints for partition 2      Setting Topologypr to Constraints for partition 3      Setting Topologypr to Constraints for partition 4      Setting Topologypr to Constraints for partition 5      Successfully set prior model parameters to all      applicable data partitions       Reporting ancestral states for partition 1 (if applicable)      Reporting ancestral states for partition 2 (if applicable)      Reporting ancestral states for partition 3 (if applicable)      Setting number of generations to 30000000      Setting sample frequency to 400      Setting print frequency to 10000      Setting diagnosing frequency to 50000      Successfully set chain parameters      Setting heating parameter to 0.070000      Setting chain output file names to "run.te.run<i>.<p/t>"      Successfully set chain parameters      Running Markov chain      MCMC stamp = 5036201787      Seed = 651447811      Swapseed = 1648603866      Model settings:          Settings for partition 1 --            Datatype  = Standard            Coding    = Variable            # States  = Variable, up to 24                        State frequencies are fixed to be equal            Rates     = Gamma                        The distribution is approximated using 4 categories.                        Shape parameter is exponentially                        distributed with parameter (1.00).          Settings for partition 2 --            Datatype  = Standard            Coding    = Variable            # States  = Variable, up to 24                        State frequencies are fixed to be equal            Rates     = Gamma                        The distribution is approximated using 4 categories.                        Shape parameter is exponentially                        distributed with parameter (1.00).          Settings for partition 3 --            Datatype  = Standard            Coding    = Variable            # States  = Variable, up to 24                        State frequencies are fixed to be equal            Rates     = Gamma                        The distribution is approximated using 4 categories.                        Shape parameter is exponentially                        distributed with parameter (1.00).          Settings for partition 4 --            Datatype  = DNA            Nucmodel  = 4by4            Nst       = 2                        Transition and transversion  rates, expressed                        as proportions of the rate sum, have a                        Beta(1.00,1.00) prior            Covarion  = No            # States  = 4                        State frequencies have a Dirichlet prior                        (1.00,1.00,1.00,1.00)            Rates     = Gamma                        The distribution is approximated using 4 categories.                        Shape parameter is exponentially                        distributed with parameter (1.00).          Settings for partition 5 --            Datatype  = DNA            Nucmodel  = 4by4            Nst       = 2                        Transition and transversion  rates, expressed                        as proportions of the rate sum, have a                        Beta(1.00,1.00) prior            Covarion  = No            # States  = 4                        State frequencies have a Dirichlet prior                        (1.00,1.00,1.00,1.00)            Rates     = Gamma                        The distribution is approximated using 4 categories.                        Shape parameter is exponentially                        distributed with parameter (1.00).       Active parameters:                               Partition(s)         Parameters          1  2  3  4  5         ---------------------------------         Tratio              .  .  .  1  2         Statefreq           3  4  5  6  7         Shape               8  8  8  9 10         Ratemultiplier     11 11 11 11 11         Topology           12 12 12 12 12         Brlens             13 13 13 13 13         Speciationrate     14 14 14 14 14         Extinctionrate     15 15 15 15 15         Fossilizationrate  16 16 16 16 16         ILNvar             17 18 19 20 20         ILNbranchrates     21 22 23 24 24         Clockrate          25 25 25 25 25         ---------------------------------          Parameters can be linked or unlinked across partitions using 'link' and 'unlink'          1 --  Parameter  = Tratio{4}               Type       = Transition and transversion rates               Prior      = Beta(1.00,1.00)               Partition  = 4          2 --  Parameter  = Tratio{5}               Type       = Transition and transversion rates               Prior      = Beta(1.00,1.00)               Partition  = 5          3 --  Parameter  = Alpha_symdir{1}               Type       = Symmetric diricihlet/beta distribution alpha_i parameter               Prior      = Symmetric dirichlet with all parameters fixed to infinity               Partition  = 1          4 --  Parameter  = Alpha_symdir{2}               Type       = Symmetric diricihlet/beta distribution alpha_i parameter               Prior      = Symmetric dirichlet with all parameters fixed to infinity               Partition  = 2          5 --  Parameter  = Alpha_symdir{3}               Type       = Symmetric diricihlet/beta distribution alpha_i parameter               Prior      = Symmetric dirichlet with all parameters fixed to infinity               Partition  = 3          6 --  Parameter  = Pi{4}               Type       = Stationary state frequencies               Prior      = Dirichlet               Partition  = 4          7 --  Parameter  = Pi{5}               Type       = Stationary state frequencies               Prior      = Dirichlet               Partition  = 5          8 --  Parameter  = Alpha{1,2,3}               Type       = Shape of scaled gamma distribution of site rates               Prior      = Exponential(1.00)               Partitions = 1, 2, and 3          9 --  Parameter  = Alpha{4}               Type       = Shape of scaled gamma distribution of site rates               Prior      = Exponential(1.00)               Partition  = 4         10 --  Parameter  = Alpha{5}               Type       = Shape of scaled gamma distribution of site rates               Prior      = Exponential(1.00)               Partition  = 5         11 --  Parameter  = Ratemultiplier{all}               Type       = Partition-specific rate multiplier               Prior      = Dirichlet(1.00,1.00,1.00,1.00,1.00)               Partitions = All         12 --  Parameter  = Tau{all}               Type       = Topology               Prior      = Prior on topology obeys the following constraints:                            -- Hard constraint "ingroup"                            -- Hard constraint "crown_lamprey"                            -- Hard constraint "north_lamprey"               Partitions = All               Subparam.  = V{all}         13 --  Parameter  = V{all}               Type       = Branch lengths               Prior      = Clock:Fossilization                            Node depths are constrained by the following age constraints:                            -- The age of terminal "Euconodonta" is Fixed(535.50)                            -- The age of terminal "Jamoytius" is Fixed(435.40)                            -- The age of terminal "Euphanerops" is Fixed(370.00)                            -- The age of terminal "Achanarella" is Fixed(385.00)                            -- The age of terminal "Ciderius" is Fixed(432.00)                            -- The age of terminal "Cornovichthys" is Fixed(385.00)                            -- The age of terminal "Lasanius" is Fixed(428.20)                            -- The age of terminal "Birkenia" is Fixed(435.40)                            -- The age of terminal "Rhyncholepis" is Fixed(431.95)                            -- The age of terminal "Myxinikela" is Fixed(280.00)                            -- The age of terminal "Tethymyxine" is Fixed(95.00)                            -- The age of terminal "Myxineidus" is Fixed(280.00)                            -- The age of terminal "Gilpichthys" is Fixed(280.00)                            -- The age of terminal "Mesomyzon" is Fixed(125.00)                            -- The age of terminal "Yanliaomyzon_ingensdentes" is Fixed(163.00)                            -- The age of terminal "Yanliaomyzon_occisor" is Fixed(158.00)                            -- The age of terminal "Priscomyzon" is Fixed(360.00)                            -- The age of terminal "Mayomyzon" is Fixed(280.00)                            -- The age of terminal "Hardistiella" is Fixed(320.00)                            -- The age of terminal "Pipiscius" is Fixed(280.00)                            -- Tree age has a Offsetexponential(500.00,600.00) distribution               Partitions = All               Subparams  = IlnBrlens{1}, IlnBrlens{2}, IlnBrlens{3} and IlnBrlens{4,5}         14 --  Parameter  = Net_speciation{all}               Type       = Speciation rate               Prior      = Exponential(100.00)               Partitions = All         15 --  Parameter  = Relative_extinction{all}               Type       = Extinction rate               Prior      = Beta(2.00,1.00)               Partitions = All         16 --  Parameter  = Relative_fossilization{all}               Type       = Fossilization rate               Prior      = Beta(1.00,9.00)               Partitions = All         17 --  Parameter  = ILNvar{1}               Type       = Variance of ILN model branch rates               Prior      = Exponential(1.00)               Partition  = 1               Subparam.  = IlnBrlens{1}         18 --  Parameter  = ILNvar{2}               Type       = Variance of ILN model branch rates               Prior      = Exponential(1.00)               Partition  = 2               Subparam.  = IlnBrlens{2}         19 --  Parameter  = ILNvar{3}               Type       = Variance of ILN model branch rates               Prior      = Exponential(1.00)               Partition  = 3               Subparam.  = IlnBrlens{3}         20 --  Parameter  = ILNvar{4,5}               Type       = Variance of ILN model branch rates               Prior      = Exponential(1.00)               Partitions = 4 and 5               Subparam.  = IlnBrlens{4,5}         21 --  Parameter  = IlnBrlens{1}               Type       = Branch lengths of ILN relaxed clock               Prior      = LogNormal (expectation = 1.0, variance = ILNvar{1})                Partition  = 1         22 --  Parameter  = IlnBrlens{2}               Type       = Branch lengths of ILN relaxed clock               Prior      = LogNormal (expectation = 1.0, variance = ILNvar{2})                Partition  = 2         23 --  Parameter  = IlnBrlens{3}               Type       = Branch lengths of ILN relaxed clock               Prior      = LogNormal (expectation = 1.0, variance = ILNvar{3})                Partition  = 3         24 --  Parameter  = IlnBrlens{4,5}               Type       = Branch lengths of ILN relaxed clock               Prior      = LogNormal (expectation = 1.0, variance = ILNvar{4,5})                Partitions = 4 and 5         25 --  Parameter  = Clockrate{all}               Type       = Base rate of clock               Prior      = Lognormal(-6.00,1.00)                            The clock rate varies according to an independent lognormal model               Partitions = All        Number of chains per MPI processor = 1       The MCMC sampler will use the following moves:         With prob.  Chain will use move            0.45 %   Dirichlet(Tratio{4})            0.45 %   Dirichlet(Tratio{5})            0.22 %   Dirichlet(Pi{4})            0.22 %   Slider(Pi{4})            0.22 %   Dirichlet(Pi{5})            0.22 %   Slider(Pi{5})            0.45 %   Multiplier(Alpha{1,2,3})            0.45 %   Multiplier(Alpha{4})            0.45 %   Multiplier(Alpha{5})            0.34 %   Dirichlet(Ratemultiplier{all})            0.34 %   Slider(Ratemultiplier{all})            4.49 %   ExtSPRClock(Tau{all},V{all})            8.99 %   NNIClock(Tau{all},V{all})            4.49 %   ParsSPRClock(Tau{all},V{all})            4.49 %   AddBranch(V{all})            4.49 %   DelBranch(V{all})           13.48 %   NodesliderClock(V{all})            1.35 %   TreeStretch(V{all})            1.35 %   Multiplier(Net_speciation{all})            1.35 %   Slider(Relative_extinction{all})            1.35 %   Slider(Relative_fossilization{all})            0.90 %   Multiplier(ILNvar{1})            0.90 %   Multiplier(ILNvar{2})            0.90 %   Multiplier(ILNvar{3})            0.90 %   Multiplier(ILNvar{4,5})           11.24 %   Multiplier(IlnBrlens{1})           11.24 %   Multiplier(IlnBrlens{2})           11.24 %   Multiplier(IlnBrlens{3})           11.24 %   Multiplier(IlnBrlens{4,5})            1.80 %   Multiplier(Clockrate{all})       Division 1 has 67 unique site patterns      Division 2 has 14 unique site patterns      Division 3 has 47 unique site patterns      Division 4 has 244 unique site patterns      Division 5 has 290 unique site patterns      Initializing conditional likelihoods      Using standard non-SSE likelihood calculator for division 1 (single-precision)      Using standard non-SSE likelihood calculator for division 2 (single-precision)      Using standard non-SSE likelihood calculator for division 3 (single-precision)      Using standard AVX likelihood calculator for division 4 (single-precision)      Using standard AVX likelihood calculator for division 5 (single-precision)       Initial log likelihoods and log prior probs for run 1:         Chain 1 -- -11289.880512 -- -7955.313504       There are 7 more chains on other processor(s)        Using a relative burnin of 25.0 % for diagnostics       Chain results (30000000 generations requested):          0 -- [-11289.881] [...7 remote chains...]       10000 -- (-7648.830) [...7 remote chains...] -- 2:29:57      20000 -- (-7627.974) [...7 remote chains...] -- 2:29:54      30000 -- (-7636.722) [...7 remote chains...] -- 2:29:51      40000 -- (-7629.076) [...7 remote chains...] -- 2:29:48      50000 -- (-7609.949) [...7 remote chains...] -- 2:29:45       Average standard deviation of split frequencies: 0.084765       60000 -- (-7616.495) [...7 remote chains...] -- 2:29:42      70000 -- (-7626.297) [...7 remote chains...] -- 2:36:46      80000 -- (-7627.991) [...7 remote chains...] -- 2:35:50      90000 -- (-7619.233) [...7 remote chains...] -- 2:35:05      100000 -- (-7611.501) [...7 remote chains...] -- 2:34:29       Average standard deviation of split frequencies: 0.074688       110000 -- (-7608.920) [...7 remote chains...] -- 2:38:30      120000 -- (-7615.955) [...7 remote chains...] -- 2:37:42      130000 -- (-7612.493) [...7 remote chains...] -- 2:37:00      140000 -- (-7620.581) [...7 remote chains...] -- 2:36:24      150000 -- (-7609.351) [...7 remote chains...] -- 2:39:12       Average standard deviation of split frequencies: 0.075668       160000 -- (-7605.323) [...7 remote chains...] -- 2:38:31      170000 -- (-7598.117) [...7 remote chains...] -- 2:37:55      180000 -- (-7616.229) [...7 remote chains...] -- 2:37:23      190000 -- (-7612.459) [...7 remote chains...] -- 2:36:53      200000 -- (-7601.140) [...7 remote chains...] -- 2:38:56       Average standard deviation of split frequencies: 0.064763       210000 -- (-7612.702) [...7 remote chains...] -- 2:38:24      220000 -- (-7615.330) [...7 remote chains...] -- 2:37:55      230000 -- (-7614.756) [...7 remote chains...] -- 2:37:28      240000 -- (-7596.453) [...7 remote chains...] -- 2:39:08      250000 -- (-7601.085) [...7 remote chains...] -- 2:38:40       Average standard deviation of split frequencies: 0.054033       260000 -- (-7600.928) [...7 remote chains...] -- 2:38:13      270000 -- (-7591.532) [...7 remote chains...] -- 2:39:39      280000 -- (-7591.333) [...7 remote chains...] -- 2:39:12      290000 -- (-7596.560) [...7 remote chains...] -- 2:38:47      300000 -- (-7622.029) [...7 remote chains...] -- 2:38:24       Average standard deviation of split frequencies: 0.049498       310000 -- (-7595.332) [...7 remote chains...] -- 2:38:01      320000 -- (-7601.194) [...7 remote chains...] -- 2:39:13      330000 -- (-7599.435) [...7 remote chains...] -- 2:38:50      340000 -- (-7599.729) [...7 remote chains...] -- 2:38:28      350000 -- (-7584.728) [...7 remote chains...] -- 2:38:08       Average standard deviation of split frequencies: 0.052272       360000 -- (-7594.400) [...7 remote chains...] -- 2:37:48      370000 -- (-7589.321) [...7 remote chains...] -- 2:38:49      380000 -- (-7606.992) [...7 remote chains...] -- 2:38:29      390000 -- (-7582.275) [...7 remote chains...] -- 2:38:10      400000 -- (-7595.685) [...7 remote chains...] -- 2:39:06       Average standard deviation of split frequencies: 0.048428       410000 -- (-7589.705) [...7 remote chains...] -- 2:38:46      420000 -- (-7602.504) [...7 remote chains...] -- 2:38:27      430000 -- (-7602.584) [...7 remote chains...] -- 2:38:09      440000 -- (-7600.790) [...7 remote chains...] -- 2:38:59      450000 -- (-7589.509) [...7 remote chains...] -- 2:38:41       Average standard deviation of split frequencies: 0.045738       460000 -- (-7593.753) [...7 remote chains...] -- 2:38:24      470000 -- (-7595.285) [...7 remote chains...] -- 2:39:10      480000 -- (-7594.969) [...7 remote chains...] -- 2:38:52      490000 -- (-7597.839) [...7 remote chains...] -- 2:38:35      500000 -- (-7606.537) [...7 remote chains...] -- 2:38:19       Average standard deviation of split frequencies: 0.046823       510000 -- (-7605.222) [...7 remote chains...] -- 2:39:00      520000 -- (-7596.457) [...7 remote chains...] -- 2:38:44      530000 -- (-7599.068) [...7 remote chains...] -- 2:38:28      540000 -- (-7593.639) [...7 remote chains...] -- 2:39:07      550000 -- (-7589.958) [...7 remote chains...] -- 2:38:51       Average standard deviation of split frequencies: 0.049455       560000 -- (-7574.409) [...7 remote chains...] -- 2:38:35      570000 -- (-7579.190) [...7 remote chains...] -- 2:39:11      580000 -- (-7572.794) [...7 remote chains...] -- 2:38:56      590000 -- (-7584.413) [...7 remote chains...] -- 2:38:40      600000 -- (-7565.693) [...7 remote chains...] -- 2:38:26       Average standard deviation of split frequencies: 0.051257       610000 -- (-7584.953) [...7 remote chains...] -- 2:38:59      620000 -- (-7585.669) [...7 remote chains...] -- 2:38:44      630000 -- (-7588.987) [...7 remote chains...] -- 2:38:30      640000 -- (-7578.045) [...7 remote chains...] -- 2:39:02      650000 -- (-7571.912) [...7 remote chains...] -- 2:38:47       Average standard deviation of split frequencies: 0.050335       660000 -- (-7578.579) [...7 remote chains...] -- 2:38:33      670000 -- (-7569.114) [...7 remote chains...] -- 2:38:19      680000 -- (-7580.593) [...7 remote chains...] -- 2:38:49      690000 -- (-7574.399) [...7 remote chains...] -- 2:38:35      700000 -- (-7579.053) [...7 remote chains...] -- 2:38:21       Average standard deviation of split frequencies: 0.057049       710000 -- (-7567.022) [...7 remote chains...] -- 2:38:49      720000 -- (-7563.037) [...7 remote chains...] -- 2:38:36      730000 -- (-7582.156) [...7 remote chains...] -- 2:38:22      740000 -- (-7560.152) [...7 remote chains...] -- 2:38:49      750000 -- (-7582.617) [...7 remote chains...] -- 2:38:36       Average standard deviation of split frequencies: 0.052247       760000 -- (-7592.694) [...7 remote chains...] -- 2:38:23      770000 -- (-7590.652) [...7 remote chains...] -- 2:38:48      780000 -- (-7581.337) [...7 remote chains...] -- 2:38:35      790000 -- (-7604.129) [...7 remote chains...] -- 2:38:22      800000 -- (-7607.724) [...7 remote chains...] -- 2:38:46       Average standard deviation of split frequencies: 0.049266       810000 -- (-7605.244) [...7 remote chains...] -- 2:38:33      820000 -- (-7575.247) [...7 remote chains...] -- 2:38:21      830000 -- (-7564.862) [...7 remote chains...] -- 2:38:44      840000 -- (-7582.251) [...7 remote chains...] -- 2:38:31      850000 -- (-7571.620) [...7 remote chains...] -- 2:38:19       Average standard deviation of split frequencies: 0.047156       860000 -- (-7580.603) [...7 remote chains...] -- 2:38:41      870000 -- (-7582.425) [...7 remote chains...] -- 2:38:29      880000 -- (-7591.131) [...7 remote chains...] -- 2:38:17      890000 -- (-7550.413) [...7 remote chains...] -- 2:38:05      900000 -- (-7576.498) [...7 remote chains...] -- 2:38:26       Average standard deviation of split frequencies: 0.045579       910000 -- (-7582.474) [...7 remote chains...] -- 2:38:14      920000 -- (-7574.759) [...7 remote chains...] -- 2:38:02      930000 -- (-7576.239) [...7 remote chains...] -- 2:38:22      940000 -- (-7581.626) [...7 remote chains...] -- 2:38:10      950000 -- (-7596.457) [...7 remote chains...] -- 2:38:30       Average standard deviation of split frequencies: 0.044335       960000 -- (-7578.943) [...7 remote chains...] -- 2:38:18      970000 -- (-7584.878) [...7 remote chains...] -- 2:38:07      980000 -- (-7583.436) [...7 remote chains...] -- 2:38:25      990000 -- (-7592.570) [...7 remote chains...] -- 2:38:14      1000000 -- (-7562.030) [...7 remote chains...] -- 2:38:03       Average standard deviation of split frequencies: 0.041119       1010000 -- (-7563.868) [...7 remote chains...] -- 2:38:20      1020000 -- (-7582.245) [...7 remote chains...] -- 2:38:09      1030000 -- (-7562.930) [...7 remote chains...] -- 2:38:26      1040000 -- (-7566.675) [...7 remote chains...] -- 2:38:15      1050000 -- (-7559.496) [...7 remote chains...] -- 2:38:04       Average standard deviation of split frequencies: 0.042122       1060000 -- (-7550.455) [...7 remote chains...] -- 2:38:21      1070000 -- (-7568.619) [...7 remote chains...] -- 2:38:10      1080000 -- (-7566.558) [...7 remote chains...] -- 2:37:59      1090000 -- (-7561.393) [...7 remote chains...] -- 2:38:15      1100000 -- (-7571.505) [...7 remote chains...] -- 2:38:04       Average standard deviation of split frequencies: 0.041743       1110000 -- (-7554.399) [...7 remote chains...] -- 2:38:19      1120000 -- (-7573.188) [...7 remote chains...] -- 2:38:09      1130000 -- (-7555.915) [...7 remote chains...] -- 2:37:58      1140000 -- (-7551.925) [...7 remote chains...] -- 2:37:48      1150000 -- (-7561.623) [...7 remote chains...] -- 2:38:02       Average standard deviation of split frequencies: 0.042252       1160000 -- (-7551.239) [...7 remote chains...] -- 2:37:52      1170000 -- (-7548.218) [...7 remote chains...] -- 2:38:06      1180000 -- (-7547.653) [...7 remote chains...] -- 2:37:56      1190000 -- (-7541.484) [...7 remote chains...] -- 2:37:46      1200000 -- (-7538.787) [...7 remote chains...] -- 2:38:00       Average standard deviation of split frequencies: 0.045521       1210000 -- (-7566.012) [...7 remote chains...] -- 2:37:49      1220000 -- (-7562.969) [...7 remote chains...] -- 2:37:39      1230000 -- (-7560.687) [...7 remote chains...] -- 2:37:53      1240000 -- (-7575.102) [...7 remote chains...] -- 2:37:42      1250000 -- (-7547.908) [...7 remote chains...] -- 2:37:33       Average standard deviation of split frequencies: 0.047345       1260000 -- (-7557.794) [...7 remote chains...] -- 2:37:45      1270000 -- (-7573.964) [...7 remote chains...] -- 2:37:36      1280000 -- (-7574.831) [...7 remote chains...] -- 2:37:26      1290000 -- (-7572.541) [...7 remote chains...] -- 2:37:38      1300000 -- (-7555.025) [...7 remote chains...] -- 2:37:28       Average standard deviation of split frequencies: 0.046262       1310000 -- (-7572.650) [...7 remote chains...] -- 2:37:19      1320000 -- (-7572.830) [...7 remote chains...] -- 2:37:31      1330000 -- (-7564.850) [...7 remote chains...] -- 2:37:21      1340000 -- (-7557.892) [...7 remote chains...] -- 2:37:12      1350000 -- (-7556.880) [...7 remote chains...] -- 2:37:23       Average standard deviation of split frequencies: 0.046442       1360000 -- (-7572.781) [...7 remote chains...] -- 2:37:14      1370000 -- (-7568.766) [...7 remote chains...] -- 2:37:04      1380000 -- (-7563.307) [...7 remote chains...] -- 2:37:16      1390000 -- (-7571.524) [...7 remote chains...] -- 2:37:06      1400000 -- (-7557.011) [...7 remote chains...] -- 2:36:57       Average standard deviation of split frequencies: 0.045606       1410000 -- (-7563.401) [...7 remote chains...] -- 2:37:08      1420000 -- (-7567.074) [...7 remote chains...] -- 2:36:59      1430000 -- (-7562.783) [...7 remote chains...] -- 2:36:50      1440000 -- (-7554.405) [...7 remote chains...] -- 2:37:00      1450000 -- (-7573.029) [...7 remote chains...] -- 2:36:51       Average standard deviation of split frequencies: 0.043031       1460000 -- (-7554.707) [...7 remote chains...] -- 2:36:42      1470000 -- (-7557.136) [...7 remote chains...] -- 2:36:52      1480000 -- (-7550.079) [...7 remote chains...] -- 2:36:43      1490000 -- (-7559.995) [...7 remote chains...] -- 2:36:54      1500000 -- (-7552.105) [...7 remote chains...] -- 2:36:45       Average standard deviation of split frequencies: 0.042390       1510000 -- (-7553.168) [...7 remote chains...] -- 2:36:36      1520000 -- (-7543.878) [...7 remote chains...] -- 2:36:45      1530000 -- (-7554.568) [...7 remote chains...] -- 2:36:36      1540000 -- (-7565.418) [...7 remote chains...] -- 2:36:28      1550000 -- (-7547.690) [...7 remote chains...] -- 2:36:37       Average standard deviation of split frequencies: 0.043528       1560000 -- (-7551.515) [...7 remote chains...] -- 2:36:28      1570000 -- (-7557.953) [...7 remote chains...] -- 2:36:20      1580000 -- (-7547.256) [...7 remote chains...] -- 2:36:29      1590000 -- (-7557.536) [...7 remote chains...] -- 2:36:20      1600000 -- (-7556.387) [...7 remote chains...] -- 2:36:29       Average standard deviation of split frequencies: 0.044253       1610000 -- (-7565.788) [...7 remote chains...] -- 2:36:21      1620000 -- [-7568.105] [...7 remote chains...] -- 2:36:12      1630000 -- [-7574.746] [...7 remote chains...] -- 2:36:21      1640000 -- [-7556.080] [...7 remote chains...] -- 2:36:12      1650000 -- [-7556.440] [...7 remote chains...] -- 2:36:21       Average standard deviation of split frequencies: 0.041273       1660000 -- [-7542.513] [...7 remote chains...] -- 2:36:12      1670000 -- [-7532.419] [...7 remote chains...] -- 2:36:04      1680000 -- [-7530.538] [...7 remote chains...] -- 2:36:12      1690000 -- [-7535.140] [...7 remote chains...] -- 2:36:04      1700000 -- [-7534.762] [...7 remote chains...] -- 2:35:55       Average standard deviation of split frequencies: 0.039856       1710000 -- [-7536.735] [...7 remote chains...] -- 2:36:03      1720000 -- [-7547.936] [...7 remote chains...] -- 2:35:55      1730000 -- [-7547.334] [...7 remote chains...] -- 2:35:47      1740000 -- [-7539.824] [...7 remote chains...] -- 2:35:55      1750000 -- [-7544.736] [...7 remote chains...] -- 2:35:46       Average standard deviation of split frequencies: 0.038067       1760000 -- [-7563.117] [...7 remote chains...] -- 2:35:38      1770000 -- [-7559.593] [...7 remote chains...] -- 2:35:46      1780000 -- [-7565.638] [...7 remote chains...] -- 2:35:37      1790000 -- [-7540.777] [...7 remote chains...] -- 2:35:29      1800000 -- [-7525.381] [...7 remote chains...] -- 2:35:37       Average standard deviation of split frequencies: 0.036001       1810000 -- [-7531.984] [...7 remote chains...] -- 2:35:29      1820000 -- [-7533.707] [...7 remote chains...] -- 2:35:36      1830000 -- [-7558.964] [...7 remote chains...] -- 2:35:28      1840000 -- [-7555.926] [...7 remote chains...] -- 2:35:20      1850000 -- (-7561.948) [...7 remote chains...] -- 2:35:27       Average standard deviation of split frequencies: 0.035423       1860000 -- [-7558.599] [...7 remote chains...] -- 2:35:19      1870000 -- [-7554.381] [...7 remote chains...] -- 2:35:26      1880000 -- [-7549.629] [...7 remote chains...] -- 2:35:18      1890000 -- (-7562.410) [...7 remote chains...] -- 2:35:10      1900000 -- (-7554.749) [...7 remote chains...] -- 2:35:17       Average standard deviation of split frequencies: 0.034733       1910000 -- [-7544.658] [...7 remote chains...] -- 2:35:09      1920000 -- [-7558.665] [...7 remote chains...] -- 2:35:16      1930000 -- (-7559.734) [...7 remote chains...] -- 2:35:08      1940000 -- [-7548.256] [...7 remote chains...] -- 2:35:00      1950000 -- [-7563.504] [...7 remote chains...] -- 2:35:06       Average standard deviation of split frequencies: 0.034084       1960000 -- [-7558.030] [...7 remote chains...] -- 2:34:58      1970000 -- [-7551.493] [...7 remote chains...] -- 2:35:05      1980000 -- [-7557.939] [...7 remote chains...] -- 2:34:57      1990000 -- [-7548.784] [...7 remote chains...] -- 2:34:49      2000000 -- [-7547.008] [...7 remote chains...] -- 2:34:56       Average standard deviation of split frequencies: 0.033038       2010000 -- [-7547.306] [...7 remote chains...] -- 2:34:48      2020000 -- [-7544.312] [...7 remote chains...] -- 2:34:54      2030000 -- [-7563.926] [...7 remote chains...] -- 2:34:46      2040000 -- (-7552.097) [...7 remote chains...] -- 2:34:38      2050000 -- (-7572.443) [...7 remote chains...] -- 2:34:44       Average standard deviation of split frequencies: 0.033041       2060000 -- (-7565.395) [...7 remote chains...] -- 2:34:37      2070000 -- (-7548.708) [...7 remote chains...] -- 2:34:43      2080000 -- (-7545.115) [...7 remote chains...] -- 2:34:35      2090000 -- (-7550.820) [...7 remote chains...] -- 2:34:27      2100000 -- (-7546.042) [...7 remote chains...] -- 2:34:33       Average standard deviation of split frequencies: 0.032949       2110000 -- [-7525.854] [...7 remote chains...] -- 2:34:25      2120000 -- (-7533.677) [...7 remote chains...] -- 2:34:18      2130000 -- (-7538.103) [...7 remote chains...] -- 2:34:23      2140000 -- [-7545.663] [...7 remote chains...] -- 2:34:16      2150000 -- [-7536.615] [...7 remote chains...] -- 2:34:08       Average standard deviation of split frequencies: 0.032926       2160000 -- [-7553.809] [...7 remote chains...] -- 2:34:14      2170000 -- (-7543.950) [...7 remote chains...] -- 2:34:06      2180000 -- (-7539.335) [...7 remote chains...] -- 2:33:59      2190000 -- (-7547.437) [...7 remote chains...] -- 2:34:04      2200000 -- (-7555.867) [...7 remote chains...] -- 2:33:57       Average standard deviation of split frequencies: 0.031574       2210000 -- (-7571.059) [...7 remote chains...] -- 2:34:02      2220000 -- (-7563.956) [...7 remote chains...] -- 2:33:54      2230000 -- (-7554.664) [...7 remote chains...] -- 2:33:47      2240000 -- (-7568.218) [...7 remote chains...] -- 2:33:52      2250000 -- (-7557.061) [...7 remote chains...] -- 2:33:45       Average standard deviation of split frequencies: 0.031108       2260000 -- (-7550.270) [...7 remote chains...] -- 2:33:50      2270000 -- (-7574.325) [...7 remote chains...] -- 2:33:42      2280000 -- (-7580.109) [...7 remote chains...] -- 2:33:35      2290000 -- (-7575.593) [...7 remote chains...] -- 2:33:40      2300000 -- (-7565.735) [...7 remote chains...] -- 2:33:33       Average standard deviation of split frequencies: 0.030979       2310000 -- (-7568.458) [...7 remote chains...] -- 2:33:26      2320000 -- (-7558.862) [...7 remote chains...] -- 2:33:30      2330000 -- (-7544.483) [...7 remote chains...] -- 2:33:23      2340000 -- (-7571.305) [...7 remote chains...] -- 2:33:16      2350000 -- (-7561.924) [...7 remote chains...] -- 2:33:20       Average standard deviation of split frequencies: 0.030315       2360000 -- (-7535.277) [...7 remote chains...] -- 2:33:13      2370000 -- (-7548.666) [...7 remote chains...] -- 2:33:18      2380000 -- (-7547.910) [...7 remote chains...] -- 2:33:11      2390000 -- (-7544.869) [...7 remote chains...] -- 2:33:04      2400000 -- (-7561.254) [...7 remote chains...] -- 2:33:08       Average standard deviation of split frequencies: 0.029816       2410000 -- (-7569.945) [...7 remote chains...] -- 2:33:01      2420000 -- [-7579.117] [...7 remote chains...] -- 2:32:54      2430000 -- (-7585.377) [...7 remote chains...] -- 2:32:58      2440000 -- (-7545.705) [...7 remote chains...] -- 2:32:51      2450000 -- (-7544.206) [...7 remote chains...] -- 2:32:55       Average standard deviation of split frequencies: 0.029121       2460000 -- [-7553.826] [...7 remote chains...] -- 2:32:48      2470000 -- [-7578.071] [...7 remote chains...] -- 2:32:41      2480000 -- [-7568.740] [...7 remote chains...] -- 2:32:45      2490000 -- [-7559.011] [...7 remote chains...] -- 2:32:38      2500000 -- [-7549.510] [...7 remote chains...] -- 2:32:32       Average standard deviation of split frequencies: 0.029295       2510000 -- [-7555.109] [...7 remote chains...] -- 2:32:36      2520000 -- [-7550.392] [...7 remote chains...] -- 2:32:29      2530000 -- (-7560.086) [...7 remote chains...] -- 2:32:22      2540000 -- (-7568.713) [...7 remote chains...] -- 2:32:26      2550000 -- (-7574.823) [...7 remote chains...] -- 2:32:19       Average standard deviation of split frequencies: 0.028894       2560000 -- (-7539.075) [...7 remote chains...] -- 2:32:12      2570000 -- (-7566.874) [...7 remote chains...] -- 2:32:16      2580000 -- (-7551.237) [...7 remote chains...] -- 2:32:09      2590000 -- (-7543.080) [...7 remote chains...] -- 2:32:02      2600000 -- (-7545.359) [...7 remote chains...] -- 2:32:06       Average standard deviation of split frequencies: 0.028787       2610000 -- (-7549.251) [...7 remote chains...] -- 2:31:59      2620000 -- (-7562.044) [...7 remote chains...] -- 2:32:03      2630000 -- [-7551.199] [...7 remote chains...] -- 2:31:56      2640000 -- (-7555.124) [...7 remote chains...] -- 2:31:49      2650000 -- (-7557.479) [...7 remote chains...] -- 2:31:53       Average standard deviation of split frequencies: 0.028063       2660000 -- (-7569.883) [...7 remote chains...] -- 2:31:46      2670000 -- (-7551.091) [...7 remote chains...] -- 2:31:39      2680000 -- (-7549.673) [...7 remote chains...] -- 2:31:43      2690000 -- (-7559.107) [...7 remote chains...] -- 2:31:36      2700000 -- (-7567.755) [...7 remote chains...] -- 2:31:40       Average standard deviation of split frequencies: 0.027472       2710000 -- [-7543.765] [...7 remote chains...] -- 2:31:33      2720000 -- (-7562.112) [...7 remote chains...] -- 2:31:26      2730000 -- (-7564.874) [...7 remote chains...] -- 2:31:30      2740000 -- [-7548.663] [...7 remote chains...] -- 2:31:23      2750000 -- [-7552.148] [...7 remote chains...] -- 2:31:16       Average standard deviation of split frequencies: 0.027485       2760000 -- [-7550.159] [...7 remote chains...] -- 2:31:20      2770000 -- [-7547.746] [...7 remote chains...] -- 2:31:13      2780000 -- [-7551.130] [...7 remote chains...] -- 2:31:16      2790000 -- [-7546.681] [...7 remote chains...] -- 2:31:10      2800000 -- [-7562.168] [...7 remote chains...] -- 2:31:03       Average standard deviation of split frequencies: 0.026862       2810000 -- [-7565.593] [...7 remote chains...] -- 2:31:06      2820000 -- (-7566.331) [...7 remote chains...] -- 2:31:00      2830000 -- [-7561.743] [...7 remote chains...] -- 2:30:53      2840000 -- [-7559.303] [...7 remote chains...] -- 2:30:56      2850000 -- (-7552.746) [...7 remote chains...] -- 2:30:50       Average standard deviation of split frequencies: 0.026784       2860000 -- (-7560.630) [...7 remote chains...] -- 2:30:43      2870000 -- (-7546.211) [...7 remote chains...] -- 2:30:46      2880000 -- [-7554.395] [...7 remote chains...] -- 2:30:40      2890000 -- [-7539.304] [...7 remote chains...] -- 2:30:42      2900000 -- [-7552.259] [...7 remote chains...] -- 2:30:36       Average standard deviation of split frequencies: 0.026411       2910000 -- [-7556.433] [...7 remote chains...] -- 2:30:30      2920000 -- [-7542.471] [...7 remote chains...] -- 2:30:32      2930000 -- (-7544.514) [...7 remote chains...] -- 2:30:26      2940000 -- (-7545.230) [...7 remote chains...] -- 2:30:20      2950000 -- [-7547.651] [...7 remote chains...] -- 2:30:22       Average standard deviation of split frequencies: 0.026063       2960000 -- (-7555.797) [...7 remote chains...] -- 2:30:16      2970000 -- [-7559.950] [...7 remote chains...] -- 2:30:10      2980000 -- (-7532.313) [...7 remote chains...] -- 2:30:12      2990000 -- [-7541.109] [...7 remote chains...] -- 2:30:06      3000000 -- [-7549.364] [...7 remote chains...] -- 2:30:00       Average standard deviation of split frequencies: 0.027280       3010000 -- (-7577.032) [...7 remote chains...] -- 2:30:02      3020000 -- (-7554.154) [...7 remote chains...] -- 2:29:56      3030000 -- [-7557.111] [...7 remote chains...] -- 2:29:50      3040000 -- [-7529.339] [...7 remote chains...] -- 2:29:52      3050000 -- [-7536.176] [...7 remote chains...] -- 2:29:46       Average standard deviation of split frequencies: 0.027908       3060000 -- (-7547.756) [...7 remote chains...] -- 2:29:48      3070000 -- (-7550.101) [...7 remote chains...] -- 2:29:42      3080000 -- (-7563.290) [...7 remote chains...] -- 2:29:36      3090000 -- (-7548.594) [...7 remote chains...] -- 2:29:38      3100000 -- [-7538.326] [...7 remote chains...] -- 2:29:32       Average standard deviation of split frequencies: 0.028157       3110000 -- (-7561.588) [...7 remote chains...] -- 2:29:26      3120000 -- [-7546.642] [...7 remote chains...] -- 2:29:28      3130000 -- [-7554.568] [...7 remote chains...] -- 2:29:22      3140000 -- (-7550.702) [...7 remote chains...] -- 2:29:16      3150000 -- [-7536.829] [...7 remote chains...] -- 2:29:18       Average standard deviation of split frequencies: 0.027252       3160000 -- [-7550.144] [...7 remote chains...] -- 2:29:12      3170000 -- [-7528.103] [...7 remote chains...] -- 2:29:06      3180000 -- [-7540.788] [...7 remote chains...] -- 2:29:08      3190000 -- (-7543.491) [...7 remote chains...] -- 2:29:02      3200000 -- (-7539.942) [...7 remote chains...] -- 2:28:56       Average standard deviation of split frequencies: 0.026548       3210000 -- (-7567.970) [...7 remote chains...] -- 2:28:58      3220000 -- [-7563.330] [...7 remote chains...] -- 2:28:52      3230000 -- (-7564.365) [...7 remote chains...] -- 2:28:54      3240000 -- (-7568.868) [...7 remote chains...] -- 2:28:48      3250000 -- [-7559.851] [...7 remote chains...] -- 2:28:42       Average standard deviation of split frequencies: 0.026024       3260000 -- (-7578.629) [...7 remote chains...] -- 2:28:44      3270000 -- [-7548.123] [...7 remote chains...] -- 2:28:38      3280000 -- [-7553.697] [...7 remote chains...] -- 2:28:32      3290000 -- [-7552.420] [...7 remote chains...] -- 2:28:34      3300000 -- (-7560.175) [...7 remote chains...] -- 2:28:28       Average standard deviation of split frequencies: 0.026424       3310000 -- (-7539.763) [...7 remote chains...] -- 2:28:22      3320000 -- (-7577.633) [...7 remote chains...] -- 2:28:24      3330000 -- [-7562.306] [...7 remote chains...] -- 2:28:18      3340000 -- (-7570.832) [...7 remote chains...] -- 2:28:11      3350000 -- (-7555.987) [...7 remote chains...] -- 2:28:13       Average standard deviation of split frequencies: 0.025845       3360000 -- [-7534.440] [...7 remote chains...] -- 2:28:07      3370000 -- (-7560.486) [...7 remote chains...] -- 2:28:01      3380000 -- (-7574.124) [...7 remote chains...] -- 2:28:03      3390000 -- (-7555.313) [...7 remote chains...] -- 2:27:57      3400000 -- (-7556.017) [...7 remote chains...] -- 2:27:59       Average standard deviation of split frequencies: 0.025153       3410000 -- (-7544.154) [...7 remote chains...] -- 2:27:53      3420000 -- (-7600.548) [...7 remote chains...] -- 2:27:47      3430000 -- (-7573.417) [...7 remote chains...] -- 2:27:49      3440000 -- (-7557.521) [...7 remote chains...] -- 2:27:43      3450000 -- (-7587.381) [...7 remote chains...] -- 2:27:37       Average standard deviation of split frequencies: 0.025716       3460000 -- (-7548.818) [...7 remote chains...] -- 2:27:39      3470000 -- (-7557.085) [...7 remote chains...] -- 2:27:33      3480000 -- (-7565.816) [...7 remote chains...] -- 2:27:35      3490000 -- (-7566.434) [...7 remote chains...] -- 2:27:29      3500000 -- (-7589.597) [...7 remote chains...] -- 2:27:23       Average standard deviation of split frequencies: 0.025980       3510000 -- (-7560.905) [...7 remote chains...] -- 2:27:25      3520000 -- (-7568.096) [...7 remote chains...] -- 2:27:19      3530000 -- (-7551.130) [...7 remote chains...] -- 2:27:13      3540000 -- (-7560.991) [...7 remote chains...] -- 2:27:14      3550000 -- (-7564.651) [...7 remote chains...] -- 2:27:09       Average standard deviation of split frequencies: 0.026330       3560000 -- (-7592.014) [...7 remote chains...] -- 2:27:03      3570000 -- (-7578.993) [...7 remote chains...] -- 2:27:04      3580000 -- (-7573.780) [...7 remote chains...] -- 2:26:58      3590000 -- (-7582.191) [...7 remote chains...] -- 2:26:53      3600000 -- (-7549.915) [...7 remote chains...] -- 2:26:54       Average standard deviation of split frequencies: 0.025588       3610000 -- (-7569.161) [...7 remote chains...] -- 2:26:48      3620000 -- (-7573.297) [...7 remote chains...] -- 2:26:43      3630000 -- (-7553.800) [...7 remote chains...] -- 2:26:44      3640000 -- (-7557.120) [...7 remote chains...] -- 2:26:38      3650000 -- (-7562.895) [...7 remote chains...] -- 2:26:32       Average standard deviation of split frequencies: 0.025268       3660000 -- (-7575.298) [...7 remote chains...] -- 2:26:34      3670000 -- (-7568.729) [...7 remote chains...] -- 2:26:28      3680000 -- (-7550.892) [...7 remote chains...] -- 2:26:22      3690000 -- (-7550.717) [...7 remote chains...] -- 2:26:24      3700000 -- (-7556.981) [...7 remote chains...] -- 2:26:18       Average standard deviation of split frequencies: 0.025332       3710000 -- (-7571.791) [...7 remote chains...] -- 2:26:12      3720000 -- (-7557.874) [...7 remote chains...] -- 2:26:14      3730000 -- (-7570.984) [...7 remote chains...] -- 2:26:08      3740000 -- (-7544.772) [...7 remote chains...] -- 2:26:02      3750000 -- (-7545.003) [...7 remote chains...] -- 2:26:04       Average standard deviation of split frequencies: 0.025706       3760000 -- (-7558.839) [...7 remote chains...] -- 2:25:58      3770000 -- (-7565.712) [...7 remote chains...] -- 2:25:52      3780000 -- (-7564.503) [...7 remote chains...] -- 2:25:53      3790000 -- (-7540.398) [...7 remote chains...] -- 2:25:48      3800000 -- (-7561.562) [...7 remote chains...] -- 2:25:49       Average standard deviation of split frequencies: 0.025309       3810000 -- (-7545.584) [...7 remote chains...] -- 2:25:43      3820000 -- (-7564.691) [...7 remote chains...] -- 2:25:38      3830000 -- (-7578.229) [...7 remote chains...] -- 2:25:39      3840000 -- (-7559.592) [...7 remote chains...] -- 2:25:33      3850000 -- (-7555.629) [...7 remote chains...] -- 2:25:27       Average standard deviation of split frequencies: 0.025382       3860000 -- (-7567.899) [...7 remote chains...] -- 2:25:29      3870000 -- (-7557.137) [...7 remote chains...] -- 2:25:23      3880000 -- [-7557.155] [...7 remote chains...] -- 2:25:17      3890000 -- [-7552.033] [...7 remote chains...] -- 2:25:18      3900000 -- [-7546.012] [...7 remote chains...] -- 2:25:13       Average standard deviation of split frequencies: 0.025652       3910000 -- [-7538.567] [...7 remote chains...] -- 2:25:14      3920000 -- [-7550.706] [...7 remote chains...] -- 2:25:08      3930000 -- [-7532.532] [...7 remote chains...] -- 2:25:03      3940000 -- (-7526.444) [...7 remote chains...] -- 2:25:04      3950000 -- [-7558.226] [...7 remote chains...] -- 2:24:58       Average standard deviation of split frequencies: 0.024421       3960000 -- [-7546.016] [...7 remote chains...] -- 2:24:53      3970000 -- [-7545.947] [...7 remote chains...] -- 2:24:54      3980000 -- (-7551.004) [...7 remote chains...] -- 2:24:48      3990000 -- (-7551.583) [...7 remote chains...] -- 2:24:43      4000000 -- (-7560.516) [...7 remote chains...] -- 2:24:44       Average standard deviation of split frequencies: 0.025068       4010000 -- (-7574.998) [...7 remote chains...] -- 2:24:38      4020000 -- (-7558.151) [...7 remote chains...] -- 2:24:39      4030000 -- (-7546.169) [...7 remote chains...] -- 2:24:33      4040000 -- (-7557.504) [...7 remote chains...] -- 2:24:34      4050000 -- (-7571.795) [...7 remote chains...] -- 2:24:29       Average standard deviation of split frequencies: 0.024362       4060000 -- (-7572.743) [...7 remote chains...] -- 2:24:23      4070000 -- (-7571.093) [...7 remote chains...] -- 2:24:24      4080000 -- (-7580.508) [...7 remote chains...] -- 2:24:19      4090000 -- (-7568.936) [...7 remote chains...] -- 2:24:13      4100000 -- (-7574.502) [...7 remote chains...] -- 2:24:14       Average standard deviation of split frequencies: 0.024084       4110000 -- [-7566.880] [...7 remote chains...] -- 2:24:08      4120000 -- [-7565.837] [...7 remote chains...] -- 2:24:09      4130000 -- (-7566.811) [...7 remote chains...] -- 2:24:04      4140000 -- [-7552.835] [...7 remote chains...] -- 2:23:58      4150000 -- [-7561.870] [...7 remote chains...] -- 2:23:59       Average standard deviation of split frequencies: 0.024254       4160000 -- [-7547.730] [...7 remote chains...] -- 2:23:54      4170000 -- [-7557.990] [...7 remote chains...] -- 2:23:54      4180000 -- (-7561.057) [...7 remote chains...] -- 2:23:49      4190000 -- [-7570.342] [...7 remote chains...] -- 2:23:43      4200000 -- (-7559.095) [...7 remote chains...] -- 2:23:44       Average standard deviation of split frequencies: 0.023505       4210000 -- (-7580.855) [...7 remote chains...] -- 2:23:39      4220000 -- (-7576.232) [...7 remote chains...] -- 2:23:33      4230000 -- (-7588.969) [...7 remote chains...] -- 2:23:34      4240000 -- (-7587.998) [...7 remote chains...] -- 2:23:28      4250000 -- (-7581.636) [...7 remote chains...] -- 2:23:23       Average standard deviation of split frequencies: 0.023231       4260000 -- (-7551.444) [...7 remote chains...] -- 2:23:24      4270000 -- (-7561.707) [...7 remote chains...] -- 2:23:18      4280000 -- (-7553.345) [...7 remote chains...] -- 2:23:13      4290000 -- (-7558.109) [...7 remote chains...] -- 2:23:13      4300000 -- (-7558.425) [...7 remote chains...] -- 2:23:08       Average standard deviation of split frequencies: 0.023478       4310000 -- (-7575.767) [...7 remote chains...] -- 2:23:09      4320000 -- (-7570.853) [...7 remote chains...] -- 2:23:03      4330000 -- (-7556.409) [...7 remote chains...] -- 2:22:58      4340000 -- (-7565.061) [...7 remote chains...] -- 2:22:58      4350000 -- (-7570.530) [...7 remote chains...] -- 2:22:53       Average standard deviation of split frequencies: 0.023207       4360000 -- (-7570.662) [...7 remote chains...] -- 2:22:48      4370000 -- (-7565.304) [...7 remote chains...] -- 2:22:42      4380000 -- (-7558.417) [...7 remote chains...] -- 2:22:43      4390000 -- (-7550.513) [...7 remote chains...] -- 2:22:38      4400000 -- (-7552.640) [...7 remote chains...] -- 2:22:32       Average standard deviation of split frequencies: 0.022766       4410000 -- (-7556.901) [...7 remote chains...] -- 2:22:33      4420000 -- (-7554.249) [...7 remote chains...] -- 2:22:27      4430000 -- (-7564.778) [...7 remote chains...] -- 2:22:22      4440000 -- (-7553.584) [...7 remote chains...] -- 2:22:23      4450000 -- (-7563.038) [...7 remote chains...] -- 2:22:17       Average standard deviation of split frequencies: 0.022260       4460000 -- (-7551.755) [...7 remote chains...] -- 2:22:18      4470000 -- (-7574.113) [...7 remote chains...] -- 2:22:12      4480000 -- (-7557.036) [...7 remote chains...] -- 2:22:07      4490000 -- (-7554.222) [...7 remote chains...] -- 2:22:07      4500000 -- (-7577.103) [...7 remote chains...] -- 2:22:02       Average standard deviation of split frequencies: 0.022284       4510000 -- (-7562.931) [...7 remote chains...] -- 2:21:57      4520000 -- (-7572.453) [...7 remote chains...] -- 2:21:57      4530000 -- (-7581.904) [...7 remote chains...] -- 2:21:52      4540000 -- (-7565.276) [...7 remote chains...] -- 2:21:47      4550000 -- (-7560.756) [...7 remote chains...] -- 2:21:47       Average standard deviation of split frequencies: 0.022521       4560000 -- (-7575.499) [...7 remote chains...] -- 2:21:42      4570000 -- (-7565.778) [...7 remote chains...] -- 2:21:42      4580000 -- (-7563.306) [...7 remote chains...] -- 2:21:37      4590000 -- (-7547.663) [...7 remote chains...] -- 2:21:32      4600000 -- (-7550.277) [...7 remote chains...] -- 2:21:32       Average standard deviation of split frequencies: 0.022255       4610000 -- (-7544.924) [...7 remote chains...] -- 2:21:27      4620000 -- [-7559.038] [...7 remote chains...] -- 2:21:21      4630000 -- (-7555.679) [...7 remote chains...] -- 2:21:22      4640000 -- (-7569.236) [...7 remote chains...] -- 2:21:17      4650000 -- (-7575.443) [...7 remote chains...] -- 2:21:11       Average standard deviation of split frequencies: 0.021952       4660000 -- (-7546.465) [...7 remote chains...] -- 2:21:06      4670000 -- [-7549.419] [...7 remote chains...] -- 2:21:06      4680000 -- (-7580.132) [...7 remote chains...] -- 2:21:01      4690000 -- (-7576.484) [...7 remote chains...] -- 2:20:56      4700000 -- (-7586.208) [...7 remote chains...] -- 2:20:56       Average standard deviation of split frequencies: 0.021587       4710000 -- (-7557.386) [...7 remote chains...] -- 2:20:51      4720000 -- (-7559.039) [...7 remote chains...] -- 2:20:46      4730000 -- (-7563.816) [...7 remote chains...] -- 2:20:46      4740000 -- (-7558.514) [...7 remote chains...] -- 2:20:41      4750000 -- (-7557.430) [...7 remote chains...] -- 2:20:36       Average standard deviation of split frequencies: 0.021435       4760000 -- (-7557.014) [...7 remote chains...] -- 2:20:36      4770000 -- (-7563.346) [...7 remote chains...] -- 2:20:31      4780000 -- (-7570.259) [...7 remote chains...] -- 2:20:26      4790000 -- (-7562.973) [...7 remote chains...] -- 2:20:26      4800000 -- (-7565.456) [...7 remote chains...] -- 2:20:21       Average standard deviation of split frequencies: 0.020741       4810000 -- (-7581.166) [...7 remote chains...] -- 2:20:15      4820000 -- (-7556.720) [...7 remote chains...] -- 2:20:15      4830000 -- (-7551.999) [...7 remote chains...] -- 2:20:10      4840000 -- (-7551.122) [...7 remote chains...] -- 2:20:05      4850000 -- (-7550.351) [...7 remote chains...] -- 2:20:05       Average standard deviation of split frequencies: 0.019836       4860000 -- (-7553.863) [...7 remote chains...] -- 2:20:00      4870000 -- (-7577.857) [...7 remote chains...] -- 2:19:55      4880000 -- (-7570.153) [...7 remote chains...] -- 2:19:55      4890000 -- (-7569.192) [...7 remote chains...] -- 2:19:50      4900000 -- (-7563.005) [...7 remote chains...] -- 2:19:45       Average standard deviation of split frequencies: 0.019828       4910000 -- (-7580.794) [...7 remote chains...] -- 2:19:45      4920000 -- (-7558.728) [...7 remote chains...] -- 2:19:40      4930000 -- (-7557.260) [...7 remote chains...] -- 2:19:35      4940000 -- (-7567.722) [...7 remote chains...] -- 2:19:35      4950000 -- (-7574.580) [...7 remote chains...] -- 2:19:30       Average standard deviation of split frequencies: 0.020256       4960000 -- (-7554.274) [...7 remote chains...] -- 2:19:30      4970000 -- (-7569.584) [...7 remote chains...] -- 2:19:25      4980000 -- (-7556.845) [...7 remote chains...] -- 2:19:20      4990000 -- (-7553.596) [...7 remote chains...] -- 2:19:20      5000000 -- (-7559.507) [...7 remote chains...] -- 2:19:15       Average standard deviation of split frequencies: 0.019815       5010000 -- (-7575.167) [...7 remote chains...] -- 2:19:09      5020000 -- (-7569.729) [...7 remote chains...] -- 2:19:09      5030000 -- (-7562.475) [...7 remote chains...] -- 2:19:04      5040000 -- (-7555.545) [...7 remote chains...] -- 2:18:59      5050000 -- (-7555.525) [...7 remote chains...] -- 2:18:59       Average standard deviation of split frequencies: 0.019417       5060000 -- (-7570.884) [...7 remote chains...] -- 2:18:54      5070000 -- (-7550.104) [...7 remote chains...] -- 2:18:49      5080000 -- (-7550.615) [...7 remote chains...] -- 2:18:44      5090000 -- (-7545.439) [...7 remote chains...] -- 2:18:44      5100000 -- (-7549.087) [...7 remote chains...] -- 2:18:39       Average standard deviation of split frequencies: 0.019111       5110000 -- (-7549.880) [...7 remote chains...] -- 2:18:34      5120000 -- (-7551.255) [...7 remote chains...] -- 2:18:34      5130000 -- (-7544.394) [...7 remote chains...] -- 2:18:29      5140000 -- (-7559.750) [...7 remote chains...] -- 2:18:24      5150000 -- (-7565.785) [...7 remote chains...] -- 2:18:24       Average standard deviation of split frequencies: 0.018857       5160000 -- (-7568.478) [...7 remote chains...] -- 2:18:19      5170000 -- (-7555.106) [...7 remote chains...] -- 2:18:14      5180000 -- (-7584.300) [...7 remote chains...] -- 2:18:14      5190000 -- (-7584.309) [...7 remote chains...] -- 2:18:09      5200000 -- (-7558.054) [...7 remote chains...] -- 2:18:04       Average standard deviation of split frequencies: 0.018871       5210000 -- (-7565.890) [...7 remote chains...] -- 2:18:03      5220000 -- (-7580.857) [...7 remote chains...] -- 2:17:58      5230000 -- (-7570.940) [...7 remote chains...] -- 2:17:54      5240000 -- (-7578.689) [...7 remote chains...] -- 2:17:53      5250000 -- (-7558.364) [...7 remote chains...] -- 2:17:48       Average standard deviation of split frequencies: 0.018901       5260000 -- (-7579.664) [...7 remote chains...] -- 2:17:48      5270000 -- (-7577.111) [...7 remote chains...] -- 2:17:43      5280000 -- (-7575.645) [...7 remote chains...] -- 2:17:38      5290000 -- (-7571.383) [...7 remote chains...] -- 2:17:38      5300000 -- (-7547.335) [...7 remote chains...] -- 2:17:33       Average standard deviation of split frequencies: 0.018910       5310000 -- (-7562.581) [...7 remote chains...] -- 2:17:28      5320000 -- (-7539.492) [...7 remote chains...] -- 2:17:28      5330000 -- (-7565.057) [...7 remote chains...] -- 2:17:23      5340000 -- (-7582.058) [...7 remote chains...] -- 2:17:18      5350000 -- (-7574.540) [...7 remote chains...] -- 2:17:18       Average standard deviation of split frequencies: 0.018764       5360000 -- (-7552.515) [...7 remote chains...] -- 2:17:13      5370000 -- (-7572.477) [...7 remote chains...] -- 2:17:12      5380000 -- [-7566.886] [...7 remote chains...] -- 2:17:08      5390000 -- [-7549.885] [...7 remote chains...] -- 2:17:03      5400000 -- [-7544.398] [...7 remote chains...] -- 2:16:58       Average standard deviation of split frequencies: 0.018525       5410000 -- [-7559.760] [...7 remote chains...] -- 2:16:57      5420000 -- (-7554.443) [...7 remote chains...] -- 2:16:52      5430000 -- [-7548.535] [...7 remote chains...] -- 2:16:52      5440000 -- [-7541.543] [...7 remote chains...] -- 2:16:47      5450000 -- [-7547.491] [...7 remote chains...] -- 2:16:42       Average standard deviation of split frequencies: 0.019111       5460000 -- (-7554.801) [...7 remote chains...] -- 2:16:42      5470000 -- (-7564.099) [...7 remote chains...] -- 2:16:37      5480000 -- (-7558.994) [...7 remote chains...] -- 2:16:32      5490000 -- (-7559.076) [...7 remote chains...] -- 2:16:32      5500000 -- (-7540.733) [...7 remote chains...] -- 2:16:27       Average standard deviation of split frequencies: 0.018966       5510000 -- (-7574.377) [...7 remote chains...] -- 2:16:22      5520000 -- (-7551.313) [...7 remote chains...] -- 2:16:22      5530000 -- (-7544.647) [...7 remote chains...] -- 2:16:17      5540000 -- (-7548.533) [...7 remote chains...] -- 2:16:12      5550000 -- (-7574.206) [...7 remote chains...] -- 2:16:12       Average standard deviation of split frequencies: 0.018883       5560000 -- (-7552.299) [...7 remote chains...] -- 2:16:07      5570000 -- (-7568.937) [...7 remote chains...] -- 2:16:06      5580000 -- (-7559.479) [...7 remote chains...] -- 2:16:01      5590000 -- (-7584.150) [...7 remote chains...] -- 2:15:57      5600000 -- (-7576.127) [...7 remote chains...] -- 2:15:56       Average standard deviation of split frequencies: 0.018651       5610000 -- (-7560.746) [...7 remote chains...] -- 2:15:51      5620000 -- (-7558.372) [...7 remote chains...] -- 2:15:46      5630000 -- (-7543.617) [...7 remote chains...] -- 2:15:46      5640000 -- (-7553.620) [...7 remote chains...] -- 2:15:41      5650000 -- (-7546.967) [...7 remote chains...] -- 2:15:36       Average standard deviation of split frequencies: 0.017926       5660000 -- (-7549.769) [...7 remote chains...] -- 2:15:36      5670000 -- (-7568.555) [...7 remote chains...] -- 2:15:31      5680000 -- (-7556.260) [...7 remote chains...] -- 2:15:26      5690000 -- (-7560.303) [...7 remote chains...] -- 2:15:26      5700000 -- (-7564.330) [...7 remote chains...] -- 2:15:21       Average standard deviation of split frequencies: 0.017834       5710000 -- (-7551.343) [...7 remote chains...] -- 2:15:16      5720000 -- (-7560.395) [...7 remote chains...] -- 2:15:15      5730000 -- (-7553.896) [...7 remote chains...] -- 2:15:11      5740000 -- (-7563.465) [...7 remote chains...] -- 2:15:10      5750000 -- (-7601.750) [...7 remote chains...] -- 2:15:05       Average standard deviation of split frequencies: 0.017666       5760000 -- (-7585.994) [...7 remote chains...] -- 2:15:01      5770000 -- (-7591.144) [...7 remote chains...] -- 2:15:00      5780000 -- (-7566.160) [...7 remote chains...] -- 2:14:55      5790000 -- (-7569.962) [...7 remote chains...] -- 2:14:55      5800000 -- (-7545.762) [...7 remote chains...] -- 2:14:50       Average standard deviation of split frequencies: 0.017931       5810000 -- (-7564.788) [...7 remote chains...] -- 2:14:45      5820000 -- (-7556.225) [...7 remote chains...] -- 2:14:44      5830000 -- (-7579.585) [...7 remote chains...] -- 2:14:40      5840000 -- (-7558.926) [...7 remote chains...] -- 2:14:35      5850000 -- (-7558.992) [...7 remote chains...] -- 2:14:34       Average standard deviation of split frequencies: 0.017701       5860000 -- (-7568.104) [...7 remote chains...] -- 2:14:30      5870000 -- (-7566.174) [...7 remote chains...] -- 2:14:25      5880000 -- (-7534.987) [...7 remote chains...] -- 2:14:24      5890000 -- (-7561.343) [...7 remote chains...] -- 2:14:19      5900000 -- (-7563.668) [...7 remote chains...] -- 2:14:15       Average standard deviation of split frequencies: 0.017811       5910000 -- (-7579.943) [...7 remote chains...] -- 2:14:14      5920000 -- (-7566.192) [...7 remote chains...] -- 2:14:09      5930000 -- (-7566.303) [...7 remote chains...] -- 2:14:04      5940000 -- (-7556.982) [...7 remote chains...] -- 2:14:04      5950000 -- (-7571.469) [...7 remote chains...] -- 2:13:59       Average standard deviation of split frequencies: 0.017927       5960000 -- (-7543.784) [...7 remote chains...] -- 2:13:54      5970000 -- (-7543.153) [...7 remote chains...] -- 2:13:50      5980000 -- (-7570.906) [...7 remote chains...] -- 2:13:49      5990000 -- (-7553.624) [...7 remote chains...] -- 2:13:44      6000000 -- (-7581.085) [...7 remote chains...] -- 2:13:39       Average standard deviation of split frequencies: 0.017958       6010000 -- (-7569.225) [...7 remote chains...] -- 2:13:39      6020000 -- (-7532.924) [...7 remote chains...] -- 2:13:34      6030000 -- (-7538.722) [...7 remote chains...] -- 2:13:29      6040000 -- (-7549.391) [...7 remote chains...] -- 2:13:29      6050000 -- (-7549.040) [...7 remote chains...] -- 2:13:24       Average standard deviation of split frequencies: 0.017747       6060000 -- (-7555.737) [...7 remote chains...] -- 2:13:19      6070000 -- (-7549.244) [...7 remote chains...] -- 2:13:19      6080000 -- (-7538.320) [...7 remote chains...] -- 2:13:14      6090000 -- (-7586.306) [...7 remote chains...] -- 2:13:09      6100000 -- (-7593.681) [...7 remote chains...] -- 2:13:08       Average standard deviation of split frequencies: 0.017011       6110000 -- (-7582.211) [...7 remote chains...] -- 2:13:04      6120000 -- (-7570.190) [...7 remote chains...] -- 2:12:59      6130000 -- (-7544.269) [...7 remote chains...] -- 2:12:58      6140000 -- (-7562.182) [...7 remote chains...] -- 2:12:54      6150000 -- (-7555.666) [...7 remote chains...] -- 2:12:49       Average standard deviation of split frequencies: 0.016677       6160000 -- (-7557.274) [...7 remote chains...] -- 2:12:48      6170000 -- (-7568.967) [...7 remote chains...] -- 2:12:43      6180000 -- (-7566.269) [...7 remote chains...] -- 2:12:39      6190000 -- (-7564.626) [...7 remote chains...] -- 2:12:38      6200000 -- (-7562.661) [...7 remote chains...] -- 2:12:33       Average standard deviation of split frequencies: 0.017088       6210000 -- [-7582.731] [...7 remote chains...] -- 2:12:29      6220000 -- [-7552.300] [...7 remote chains...] -- 2:12:28      6230000 -- (-7571.495) [...7 remote chains...] -- 2:12:23      6240000 -- [-7548.372] [...7 remote chains...] -- 2:12:19      6250000 -- (-7556.808) [...7 remote chains...] -- 2:12:18       Average standard deviation of split frequencies: 0.016966       6260000 -- (-7560.573) [...7 remote chains...] -- 2:12:13      6270000 -- (-7566.216) [...7 remote chains...] -- 2:12:08      6280000 -- [-7546.707] [...7 remote chains...] -- 2:12:08      6290000 -- [-7559.411] [...7 remote chains...] -- 2:12:03      6300000 -- [-7555.794] [...7 remote chains...] -- 2:11:58       Average standard deviation of split frequencies: 0.016965       6310000 -- (-7576.869) [...7 remote chains...] -- 2:11:57      6320000 -- [-7547.904] [...7 remote chains...] -- 2:11:53      6330000 -- [-7556.719] [...7 remote chains...] -- 2:11:52      6340000 -- (-7548.357) [...7 remote chains...] -- 2:11:47      6350000 -- (-7567.224) [...7 remote chains...] -- 2:11:43       Average standard deviation of split frequencies: 0.016970       6360000 -- [-7564.972] [...7 remote chains...] -- 2:11:38      6370000 -- [-7553.293] [...7 remote chains...] -- 2:11:37      6380000 -- [-7549.436] [...7 remote chains...] -- 2:11:33      6390000 -- [-7549.240] [...7 remote chains...] -- 2:11:32      6400000 -- [-7544.183] [...7 remote chains...] -- 2:11:27       Average standard deviation of split frequencies: 0.016996       6410000 -- [-7551.115] [...7 remote chains...] -- 2:11:22      6420000 -- [-7558.688] [...7 remote chains...] -- 2:11:22      6430000 -- (-7547.213) [...7 remote chains...] -- 2:11:17      6440000 -- (-7572.895) [...7 remote chains...] -- 2:11:12      6450000 -- [-7565.805] [...7 remote chains...] -- 2:11:11       Average standard deviation of split frequencies: 0.016950       6460000 -- [-7569.687] [...7 remote chains...] -- 2:11:07      6470000 -- [-7568.303] [...7 remote chains...] -- 2:11:02      6480000 -- (-7563.069) [...7 remote chains...] -- 2:11:01      6490000 -- (-7557.218) [...7 remote chains...] -- 2:10:57      6500000 -- (-7563.662) [...7 remote chains...] -- 2:10:52       Average standard deviation of split frequencies: 0.016691       6510000 -- (-7562.576) [...7 remote chains...] -- 2:10:51      6520000 -- (-7579.396) [...7 remote chains...] -- 2:10:47      6530000 -- (-7554.642) [...7 remote chains...] -- 2:10:42      6540000 -- (-7584.811) [...7 remote chains...] -- 2:10:41      6550000 -- (-7547.895) [...7 remote chains...] -- 2:10:36       Average standard deviation of split frequencies: 0.016538       6560000 -- (-7552.871) [...7 remote chains...] -- 2:10:32      6570000 -- (-7582.385) [...7 remote chains...] -- 2:10:31      6580000 -- (-7569.726) [...7 remote chains...] -- 2:10:26      6590000 -- (-7552.552) [...7 remote chains...] -- 2:10:25      6600000 -- (-7547.307) [...7 remote chains...] -- 2:10:21       Average standard deviation of split frequencies: 0.016344       6610000 -- (-7560.197) [...7 remote chains...] -- 2:10:16      6620000 -- (-7556.648) [...7 remote chains...] -- 2:10:12      6630000 -- (-7535.333) [...7 remote chains...] -- 2:10:11      6640000 -- (-7556.104) [...7 remote chains...] -- 2:10:06      6650000 -- (-7563.521) [...7 remote chains...] -- 2:10:02       Average standard deviation of split frequencies: 0.016442       6660000 -- (-7557.233) [...7 remote chains...] -- 2:10:01      6670000 -- (-7565.006) [...7 remote chains...] -- 2:09:56      6680000 -- (-7585.239) [...7 remote chains...] -- 2:09:51      6690000 -- (-7553.879) [...7 remote chains...] -- 2:09:50      6700000 -- (-7560.397) [...7 remote chains...] -- 2:09:46       Average standard deviation of split frequencies: 0.016174       6710000 -- (-7560.669) [...7 remote chains...] -- 2:09:41      6720000 -- (-7556.759) [...7 remote chains...] -- 2:09:40      6730000 -- (-7572.849) [...7 remote chains...] -- 2:09:36      6740000 -- (-7564.134) [...7 remote chains...] -- 2:09:31      6750000 -- (-7571.295) [...7 remote chains...] -- 2:09:30       Average standard deviation of split frequencies: 0.015906       6760000 -- (-7557.856) [...7 remote chains...] -- 2:09:26      6770000 -- (-7557.352) [...7 remote chains...] -- 2:09:21      6780000 -- (-7559.968) [...7 remote chains...] -- 2:09:20      6790000 -- (-7594.187) [...7 remote chains...] -- 2:09:16      6800000 -- (-7570.544) [...7 remote chains...] -- 2:09:11       Average standard deviation of split frequencies: 0.015805       6810000 -- (-7583.550) [...7 remote chains...] -- 2:09:10      6820000 -- (-7593.349) [...7 remote chains...] -- 2:09:05      6830000 -- (-7571.280) [...7 remote chains...] -- 2:09:04      6840000 -- (-7583.439) [...7 remote chains...] -- 2:09:00      6850000 -- (-7545.662) [...7 remote chains...] -- 2:08:55       Average standard deviation of split frequencies: 0.015827       6860000 -- (-7536.832) [...7 remote chains...] -- 2:08:51      6870000 -- (-7547.470) [...7 remote chains...] -- 2:08:50      6880000 -- (-7571.592) [...7 remote chains...] -- 2:08:45      6890000 -- (-7565.488) [...7 remote chains...] -- 2:08:41      6900000 -- (-7557.693) [...7 remote chains...] -- 2:08:40       Average standard deviation of split frequencies: 0.015933       6910000 -- (-7558.159) [...7 remote chains...] -- 2:08:35      6920000 -- (-7585.054) [...7 remote chains...] -- 2:08:34      6930000 -- (-7555.009) [...7 remote chains...] -- 2:08:29      6940000 -- (-7560.451) [...7 remote chains...] -- 2:08:25      6950000 -- (-7578.655) [...7 remote chains...] -- 2:08:24       Average standard deviation of split frequencies: 0.016301       6960000 -- (-7562.145) [...7 remote chains...] -- 2:08:19      6970000 -- (-7552.145) [...7 remote chains...] -- 2:08:15      6980000 -- [-7561.568] [...7 remote chains...] -- 2:08:14      6990000 -- (-7559.965) [...7 remote chains...] -- 2:08:09      7000000 -- (-7576.550) [...7 remote chains...] -- 2:08:08       Average standard deviation of split frequencies: 0.016714       7010000 -- (-7557.567) [...7 remote chains...] -- 2:08:04      7020000 -- (-7558.975) [...7 remote chains...] -- 2:07:59      7030000 -- [-7566.055] [...7 remote chains...] -- 2:07:58      7040000 -- (-7565.192) [...7 remote chains...] -- 2:07:53      7050000 -- (-7561.724) [...7 remote chains...] -- 2:07:52       Average standard deviation of split frequencies: 0.017122       7060000 -- (-7556.734) [...7 remote chains...] -- 2:07:48      7070000 -- (-7545.929) [...7 remote chains...] -- 2:07:43      7080000 -- (-7544.664) [...7 remote chains...] -- 2:07:42      7090000 -- (-7550.981) [...7 remote chains...] -- 2:07:38      7100000 -- (-7556.890) [...7 remote chains...] -- 2:07:33       Average standard deviation of split frequencies: 0.017842       7110000 -- (-7579.609) [...7 remote chains...] -- 2:07:32      7120000 -- (-7565.843) [...7 remote chains...] -- 2:07:28      7130000 -- (-7548.644) [...7 remote chains...] -- 2:07:26      7140000 -- (-7567.610) [...7 remote chains...] -- 2:07:22      7150000 -- (-7589.953) [...7 remote chains...] -- 2:07:17       Average standard deviation of split frequencies: 0.018373       7160000 -- (-7571.287) [...7 remote chains...] -- 2:07:13      7170000 -- (-7564.393) [...7 remote chains...] -- 2:07:12      7180000 -- (-7565.352) [...7 remote chains...] -- 2:07:07      7190000 -- (-7548.713) [...7 remote chains...] -- 2:07:06      7200000 -- (-7549.103) [...7 remote chains...] -- 2:07:02       Average standard deviation of split frequencies: 0.018478       7210000 -- (-7553.294) [...7 remote chains...] -- 2:06:57      7220000 -- (-7540.813) [...7 remote chains...] -- 2:06:56      7230000 -- (-7551.809) [...7 remote chains...] -- 2:06:52      7240000 -- (-7544.320) [...7 remote chains...] -- 2:06:47      7250000 -- (-7570.311) [...7 remote chains...] -- 2:06:46       Average standard deviation of split frequencies: 0.018461       7260000 -- (-7585.420) [...7 remote chains...] -- 2:06:41      7270000 -- (-7585.975) [...7 remote chains...] -- 2:06:37      7280000 -- (-7545.382) [...7 remote chains...] -- 2:06:36      7290000 -- (-7557.291) [...7 remote chains...] -- 2:06:31      7300000 -- (-7554.799) [...7 remote chains...] -- 2:06:27       Average standard deviation of split frequencies: 0.018270       7310000 -- (-7565.531) [...7 remote chains...] -- 2:06:26      7320000 -- (-7562.487) [...7 remote chains...] -- 2:06:21      7330000 -- (-7553.390) [...7 remote chains...] -- 2:06:17      7340000 -- (-7573.618) [...7 remote chains...] -- 2:06:15      7350000 -- (-7561.550) [...7 remote chains...] -- 2:06:11       Average standard deviation of split frequencies: 0.018377       7360000 -- (-7559.686) [...7 remote chains...] -- 2:06:10      7370000 -- (-7559.582) [...7 remote chains...] -- 2:06:05      7380000 -- (-7565.629) [...7 remote chains...] -- 2:06:04      7390000 -- (-7566.526) [...7 remote chains...] -- 2:06:00      7400000 -- (-7566.243) [...7 remote chains...] -- 2:05:55       Average standard deviation of split frequencies: 0.018310       7410000 -- (-7556.313) [...7 remote chains...] -- 2:05:54      7420000 -- (-7559.754) [...7 remote chains...] -- 2:05:49      7430000 -- (-7572.875) [...7 remote chains...] -- 2:05:45      7440000 -- (-7573.844) [...7 remote chains...] -- 2:05:44      7450000 -- (-7558.860) [...7 remote chains...] -- 2:05:39       Average standard deviation of split frequencies: 0.018017       7460000 -- [-7558.567] [...7 remote chains...] -- 2:05:38      7470000 -- (-7558.302) [...7 remote chains...] -- 2:05:34      7480000 -- (-7568.318) [...7 remote chains...] -- 2:05:29      7490000 -- (-7583.414) [...7 remote chains...] -- 2:05:25      7500000 -- (-7561.633) [...7 remote chains...] -- 2:05:24       Average standard deviation of split frequencies: 0.017967       7510000 -- (-7555.074) [...7 remote chains...] -- 2:05:19      7520000 -- (-7559.925) [...7 remote chains...] -- 2:05:15      7530000 -- (-7572.050) [...7 remote chains...] -- 2:05:13      7540000 -- (-7542.356) [...7 remote chains...] -- 2:05:09      7550000 -- (-7544.097) [...7 remote chains...] -- 2:05:05       Average standard deviation of split frequencies: 0.017724       7560000 -- (-7568.633) [...7 remote chains...] -- 2:05:03      7570000 -- (-7579.881) [...7 remote chains...] -- 2:04:59      7580000 -- (-7564.243) [...7 remote chains...] -- 2:04:55      7590000 -- (-7556.677) [...7 remote chains...] -- 2:04:53      7600000 -- (-7549.576) [...7 remote chains...] -- 2:04:49       Average standard deviation of split frequencies: 0.017686       7610000 -- (-7566.881) [...7 remote chains...] -- 2:04:44      7620000 -- (-7569.208) [...7 remote chains...] -- 2:04:40      7630000 -- (-7567.128) [...7 remote chains...] -- 2:04:39      7640000 -- (-7563.980) [...7 remote chains...] -- 2:04:34      7650000 -- (-7562.628) [...7 remote chains...] -- 2:04:30       Average standard deviation of split frequencies: 0.017922       7660000 -- (-7560.083) [...7 remote chains...] -- 2:04:29      7670000 -- (-7571.641) [...7 remote chains...] -- 2:04:24      7680000 -- (-7549.090) [...7 remote chains...] -- 2:04:20      7690000 -- (-7567.203) [...7 remote chains...] -- 2:04:18      7700000 -- (-7536.881) [...7 remote chains...] -- 2:04:14       Average standard deviation of split frequencies: 0.018018       7710000 -- (-7574.820) [...7 remote chains...] -- 2:04:10      7720000 -- (-7556.890) [...7 remote chains...] -- 2:04:08      7730000 -- (-7576.478) [...7 remote chains...] -- 2:04:04      7740000 -- (-7563.278) [...7 remote chains...] -- 2:04:00      7750000 -- (-7567.872) [...7 remote chains...] -- 2:03:58       Average standard deviation of split frequencies: 0.018282       7760000 -- (-7545.233) [...7 remote chains...] -- 2:03:54      7770000 -- (-7554.254) [...7 remote chains...] -- 2:03:50      7780000 -- (-7553.561) [...7 remote chains...] -- 2:03:48      7790000 -- (-7570.806) [...7 remote chains...] -- 2:03:44      7800000 -- (-7561.995) [...7 remote chains...] -- 2:03:39       Average standard deviation of split frequencies: 0.017899       7810000 -- (-7547.001) [...7 remote chains...] -- 2:03:38      7820000 -- (-7577.868) [...7 remote chains...] -- 2:03:34      7830000 -- (-7547.237) [...7 remote chains...] -- 2:03:29      7840000 -- (-7558.500) [...7 remote chains...] -- 2:03:28      7850000 -- (-7567.584) [...7 remote chains...] -- 2:03:24       Average standard deviation of split frequencies: 0.018148       7860000 -- (-7558.248) [...7 remote chains...] -- 2:03:19      7870000 -- (-7560.140) [...7 remote chains...] -- 2:03:18      7880000 -- (-7581.339) [...7 remote chains...] -- 2:03:13      7890000 -- (-7576.271) [...7 remote chains...] -- 2:03:09      7900000 -- (-7580.174) [...7 remote chains...] -- 2:03:08       Average standard deviation of split frequencies: 0.018113       7910000 -- (-7594.981) [...7 remote chains...] -- 2:03:03      7920000 -- (-7574.993) [...7 remote chains...] -- 2:03:02      7930000 -- (-7582.358) [...7 remote chains...] -- 2:02:58      7940000 -- (-7580.819) [...7 remote chains...] -- 2:02:53      7950000 -- (-7550.346) [...7 remote chains...] -- 2:02:49       Average standard deviation of split frequencies: 0.017845       7960000 -- (-7572.627) [...7 remote chains...] -- 2:02:47      7970000 -- (-7568.628) [...7 remote chains...] -- 2:02:43      7980000 -- (-7578.734) [...7 remote chains...] -- 2:02:39      7990000 -- [-7562.893] [...7 remote chains...] -- 2:02:37      8000000 -- (-7569.016) [...7 remote chains...] -- 2:02:33       Average standard deviation of split frequencies: 0.017770       8010000 -- [-7559.146] [...7 remote chains...] -- 2:02:29      8020000 -- (-7562.218) [...7 remote chains...] -- 2:02:27      8030000 -- [-7560.284] [...7 remote chains...] -- 2:02:23      8040000 -- (-7555.918) [...7 remote chains...] -- 2:02:19      8050000 -- (-7557.876) [...7 remote chains...] -- 2:02:17       Average standard deviation of split frequencies: 0.017951       8060000 -- (-7577.792) [...7 remote chains...] -- 2:02:13      8070000 -- (-7565.222) [...7 remote chains...] -- 2:02:09      8080000 -- (-7564.290) [...7 remote chains...] -- 2:02:07      8090000 -- (-7563.294) [...7 remote chains...] -- 2:02:03      8100000 -- (-7580.280) [...7 remote chains...] -- 2:01:58       Average standard deviation of split frequencies: 0.017480       8110000 -- (-7574.347) [...7 remote chains...] -- 2:01:54      8120000 -- (-7560.100) [...7 remote chains...] -- 2:01:53      8130000 -- (-7559.991) [...7 remote chains...] -- 2:01:48      8140000 -- (-7572.196) [...7 remote chains...] -- 2:01:44      8150000 -- (-7551.428) [...7 remote chains...] -- 2:01:42       Average standard deviation of split frequencies: 0.017123       8160000 -- (-7555.601) [...7 remote chains...] -- 2:01:38      8170000 -- (-7537.274) [...7 remote chains...] -- 2:01:34      8180000 -- (-7573.967) [...7 remote chains...] -- 2:01:32      8190000 -- (-7582.681) [...7 remote chains...] -- 2:01:28      8200000 -- (-7559.967) [...7 remote chains...] -- 2:01:24       Average standard deviation of split frequencies: 0.017199       8210000 -- (-7544.128) [...7 remote chains...] -- 2:01:20      8220000 -- (-7562.422) [...7 remote chains...] -- 2:01:18      8230000 -- (-7550.288) [...7 remote chains...] -- 2:01:14      8240000 -- (-7562.749) [...7 remote chains...] -- 2:01:10      8250000 -- (-7549.020) [...7 remote chains...] -- 2:01:08       Average standard deviation of split frequencies: 0.017409       8260000 -- (-7569.437) [...7 remote chains...] -- 2:01:04      8270000 -- (-7570.088) [...7 remote chains...] -- 2:00:59      8280000 -- (-7574.064) [...7 remote chains...] -- 2:00:58      8290000 -- (-7558.844) [...7 remote chains...] -- 2:00:54      8300000 -- (-7571.293) [...7 remote chains...] -- 2:00:49       Average standard deviation of split frequencies: 0.017402       8310000 -- (-7563.422) [...7 remote chains...] -- 2:00:45      8320000 -- (-7573.689) [...7 remote chains...] -- 2:00:44      8330000 -- (-7563.178) [...7 remote chains...] -- 2:00:39      8340000 -- (-7559.681) [...7 remote chains...] -- 2:00:35      8350000 -- (-7565.009) [...7 remote chains...] -- 2:00:33       Average standard deviation of split frequencies: 0.017297       8360000 -- (-7547.505) [...7 remote chains...] -- 2:00:29      8370000 -- (-7552.499) [...7 remote chains...] -- 2:00:28      8380000 -- (-7552.839) [...7 remote chains...] -- 2:00:23      8390000 -- (-7548.762) [...7 remote chains...] -- 2:00:19      8400000 -- (-7562.113) [...7 remote chains...] -- 2:00:17       Average standard deviation of split frequencies: 0.017436       8410000 -- (-7556.493) [...7 remote chains...] -- 2:00:13      8420000 -- (-7569.055) [...7 remote chains...] -- 2:00:09      8430000 -- (-7548.153) [...7 remote chains...] -- 2:00:05      8440000 -- (-7549.983) [...7 remote chains...] -- 2:00:03      8450000 -- (-7546.931) [...7 remote chains...] -- 1:59:59       Average standard deviation of split frequencies: 0.017649       8460000 -- (-7542.507) [...7 remote chains...] -- 1:59:55      8470000 -- (-7560.625) [...7 remote chains...] -- 1:59:53      8480000 -- (-7558.804) [...7 remote chains...] -- 1:59:49      8490000 -- (-7569.972) [...7 remote chains...] -- 1:59:45      8500000 -- (-7560.777) [...7 remote chains...] -- 1:59:43       Average standard deviation of split frequencies: 0.017465       8510000 -- (-7561.235) [...7 remote chains...] -- 1:59:39      8520000 -- (-7582.127) [...7 remote chains...] -- 1:59:35      8530000 -- (-7579.150) [...7 remote chains...] -- 1:59:33      8540000 -- (-7574.131) [...7 remote chains...] -- 1:59:29      8550000 -- (-7563.922) [...7 remote chains...] -- 1:59:25       Average standard deviation of split frequencies: 0.017293       8560000 -- (-7548.759) [...7 remote chains...] -- 1:59:23      8570000 -- (-7548.733) [...7 remote chains...] -- 1:59:19      8580000 -- (-7574.132) [...7 remote chains...] -- 1:59:14      8590000 -- (-7564.500) [...7 remote chains...] -- 1:59:13      8600000 -- [-7556.378] [...7 remote chains...] -- 1:59:09       Average standard deviation of split frequencies: 0.017232       8610000 -- [-7559.325] [...7 remote chains...] -- 1:59:04      8620000 -- (-7552.547) [...7 remote chains...] -- 1:59:03      8630000 -- [-7546.671] [...7 remote chains...] -- 1:58:59      8640000 -- [-7552.484] [...7 remote chains...] -- 1:58:54      8650000 -- [-7564.256] [...7 remote chains...] -- 1:58:50       Average standard deviation of split frequencies: 0.017024       8660000 -- [-7547.464] [...7 remote chains...] -- 1:58:48      8670000 -- [-7548.204] [...7 remote chains...] -- 1:58:44      8680000 -- [-7547.144] [...7 remote chains...] -- 1:58:40      8690000 -- [-7551.091] [...7 remote chains...] -- 1:58:38      8700000 -- (-7550.335) [...7 remote chains...] -- 1:58:34       Average standard deviation of split frequencies: 0.017035       8710000 -- (-7529.369) [...7 remote chains...] -- 1:58:30      8720000 -- (-7544.850) [...7 remote chains...] -- 1:58:28      8730000 -- (-7547.342) [...7 remote chains...] -- 1:58:24      8740000 -- (-7550.775) [...7 remote chains...] -- 1:58:20      8750000 -- [-7548.359] [...7 remote chains...] -- 1:58:18       Average standard deviation of split frequencies: 0.016705       8760000 -- (-7564.101) [...7 remote chains...] -- 1:58:14      8770000 -- [-7557.111] [...7 remote chains...] -- 1:58:10      8780000 -- [-7551.422] [...7 remote chains...] -- 1:58:06      8790000 -- (-7562.957) [...7 remote chains...] -- 1:58:04      8800000 -- [-7564.241] [...7 remote chains...] -- 1:58:00       Average standard deviation of split frequencies: 0.016625       8810000 -- [-7550.807] [...7 remote chains...] -- 1:57:56      8820000 -- [-7552.818] [...7 remote chains...] -- 1:57:54      8830000 -- (-7587.944) [...7 remote chains...] -- 1:57:50      8840000 -- (-7563.430) [...7 remote chains...] -- 1:57:48      8850000 -- (-7565.150) [...7 remote chains...] -- 1:57:44       Average standard deviation of split frequencies: 0.016439       8860000 -- (-7539.910) [...7 remote chains...] -- 1:57:40      8870000 -- (-7574.248) [...7 remote chains...] -- 1:57:38      8880000 -- (-7563.987) [...7 remote chains...] -- 1:57:34      8890000 -- [-7554.232] [...7 remote chains...] -- 1:57:30      8900000 -- (-7567.828) [...7 remote chains...] -- 1:57:25       Average standard deviation of split frequencies: 0.016370       8910000 -- (-7566.733) [...7 remote chains...] -- 1:57:24      8920000 -- (-7563.217) [...7 remote chains...] -- 1:57:20      8930000 -- (-7560.437) [...7 remote chains...] -- 1:57:15      8940000 -- (-7551.193) [...7 remote chains...] -- 1:57:14      8950000 -- (-7548.337) [...7 remote chains...] -- 1:57:09       Average standard deviation of split frequencies: 0.015965       8960000 -- (-7561.214) [...7 remote chains...] -- 1:57:05      8970000 -- (-7545.378) [...7 remote chains...] -- 1:57:04      8980000 -- (-7554.353) [...7 remote chains...] -- 1:56:59      8990000 -- (-7566.830) [...7 remote chains...] -- 1:56:55      9000000 -- (-7559.171) [...7 remote chains...] -- 1:56:54       Average standard deviation of split frequencies: 0.015638       9010000 -- (-7565.048) [...7 remote chains...] -- 1:56:49      9020000 -- (-7576.634) [...7 remote chains...] -- 1:56:45      9030000 -- (-7573.043) [...7 remote chains...] -- 1:56:43      9040000 -- (-7585.403) [...7 remote chains...] -- 1:56:39      9050000 -- (-7587.054) [...7 remote chains...] -- 1:56:35       Average standard deviation of split frequencies: 0.015687       9060000 -- (-7574.933) [...7 remote chains...] -- 1:56:33      9070000 -- (-7562.080) [...7 remote chains...] -- 1:56:29      9080000 -- (-7559.212) [...7 remote chains...] -- 1:56:25      9090000 -- (-7580.302) [...7 remote chains...] -- 1:56:23      9100000 -- (-7553.876) [...7 remote chains...] -- 1:56:19       Average standard deviation of split frequencies: 0.015509       9110000 -- (-7554.636) [...7 remote chains...] -- 1:56:15      9120000 -- (-7585.145) [...7 remote chains...] -- 1:56:13      9130000 -- (-7561.814) [...7 remote chains...] -- 1:56:09      9140000 -- [-7545.415] [...7 remote chains...] -- 1:56:05      9150000 -- [-7559.905] [...7 remote chains...] -- 1:56:03       Average standard deviation of split frequencies: 0.015256       9160000 -- [-7566.853] [...7 remote chains...] -- 1:55:59      9170000 -- (-7547.204) [...7 remote chains...] -- 1:55:55      9180000 -- (-7565.773) [...7 remote chains...] -- 1:55:53      9190000 -- (-7555.283) [...7 remote chains...] -- 1:55:49      9200000 -- (-7546.974) [...7 remote chains...] -- 1:55:45       Average standard deviation of split frequencies: 0.015340       9210000 -- (-7581.456) [...7 remote chains...] -- 1:55:43      9220000 -- (-7560.311) [...7 remote chains...] -- 1:55:39      9230000 -- (-7578.493) [...7 remote chains...] -- 1:55:37      9240000 -- (-7574.806) [...7 remote chains...] -- 1:55:33      9250000 -- (-7567.574) [...7 remote chains...] -- 1:55:29       Average standard deviation of split frequencies: 0.015608       9260000 -- (-7583.183) [...7 remote chains...] -- 1:55:25      9270000 -- (-7579.863) [...7 remote chains...] -- 1:55:23      9280000 -- (-7572.987) [...7 remote chains...] -- 1:55:19      9290000 -- (-7562.603) [...7 remote chains...] -- 1:55:15      9300000 -- (-7559.404) [...7 remote chains...] -- 1:55:13       Average standard deviation of split frequencies: 0.015421       9310000 -- (-7554.388) [...7 remote chains...] -- 1:55:09      9320000 -- (-7576.761) [...7 remote chains...] -- 1:55:05      9330000 -- (-7585.404) [...7 remote chains...] -- 1:55:03      9340000 -- (-7566.682) [...7 remote chains...] -- 1:54:59      9350000 -- (-7561.855) [...7 remote chains...] -- 1:54:55       Average standard deviation of split frequencies: 0.015283       9360000 -- (-7554.781) [...7 remote chains...] -- 1:54:51      9370000 -- (-7565.770) [...7 remote chains...] -- 1:54:49      9380000 -- (-7557.733) [...7 remote chains...] -- 1:54:45      9390000 -- (-7561.332) [...7 remote chains...] -- 1:54:40      9400000 -- (-7569.722) [...7 remote chains...] -- 1:54:39       Average standard deviation of split frequencies: 0.015001       9410000 -- (-7554.351) [...7 remote chains...] -- 1:54:35      9420000 -- (-7575.163) [...7 remote chains...] -- 1:54:30      9430000 -- (-7548.115) [...7 remote chains...] -- 1:54:29      9440000 -- (-7565.692) [...7 remote chains...] -- 1:54:24      9450000 -- (-7584.282) [...7 remote chains...] -- 1:54:20       Average standard deviation of split frequencies: 0.014976       9460000 -- (-7559.009) [...7 remote chains...] -- 1:54:18      9470000 -- (-7572.055) [...7 remote chains...] -- 1:54:14      9480000 -- (-7581.981) [...7 remote chains...] -- 1:54:10      9490000 -- (-7558.681) [...7 remote chains...] -- 1:54:06      9500000 -- (-7549.662) [...7 remote chains...] -- 1:54:04       Average standard deviation of split frequencies: 0.015026       9510000 -- (-7570.496) [...7 remote chains...] -- 1:54:00      9520000 -- (-7555.272) [...7 remote chains...] -- 1:53:56      9530000 -- (-7553.282) [...7 remote chains...] -- 1:53:54      9540000 -- (-7564.147) [...7 remote chains...] -- 1:53:50      9550000 -- (-7583.444) [...7 remote chains...] -- 1:53:46       Average standard deviation of split frequencies: 0.014938       9560000 -- (-7577.786) [...7 remote chains...] -- 1:53:44      9570000 -- (-7573.982) [...7 remote chains...] -- 1:53:40      9580000 -- (-7560.847) [...7 remote chains...] -- 1:53:36      9590000 -- (-7580.194) [...7 remote chains...] -- 1:53:32      9600000 -- (-7554.512) [...7 remote chains...] -- 1:53:30       Average standard deviation of split frequencies: 0.014824       9610000 -- (-7551.623) [...7 remote chains...] -- 1:53:26      9620000 -- (-7555.603) [...7 remote chains...] -- 1:53:22      9630000 -- (-7550.291) [...7 remote chains...] -- 1:53:20      9640000 -- (-7558.454) [...7 remote chains...] -- 1:53:16      9650000 -- [-7552.973] [...7 remote chains...] -- 1:53:12       Average standard deviation of split frequencies: 0.014815       9660000 -- (-7571.481) [...7 remote chains...] -- 1:53:10      9670000 -- (-7553.871) [...7 remote chains...] -- 1:53:06      9680000 -- (-7556.854) [...7 remote chains...] -- 1:53:02      9690000 -- (-7555.872) [...7 remote chains...] -- 1:52:58      9700000 -- (-7551.786) [...7 remote chains...] -- 1:52:56       Average standard deviation of split frequencies: 0.015091       9710000 -- (-7557.991) [...7 remote chains...] -- 1:52:52      9720000 -- (-7558.180) [...7 remote chains...] -- 1:52:48      9730000 -- (-7562.131) [...7 remote chains...] -- 1:52:44      9740000 -- (-7576.841) [...7 remote chains...] -- 1:52:42      9750000 -- (-7576.332) [...7 remote chains...] -- 1:52:38       Average standard deviation of split frequencies: 0.015195       9760000 -- (-7570.063) [...7 remote chains...] -- 1:52:34      9770000 -- (-7534.007) [...7 remote chains...] -- 1:52:32      9780000 -- (-7563.775) [...7 remote chains...] -- 1:52:28      9790000 -- (-7597.318) [...7 remote chains...] -- 1:52:24      9800000 -- (-7553.875) [...7 remote chains...] -- 1:52:22       Average standard deviation of split frequencies: 0.015095       9810000 -- (-7588.740) [...7 remote chains...] -- 1:52:18      9820000 -- (-7569.518) [...7 remote chains...] -- 1:52:14      9830000 -- (-7556.032) [...7 remote chains...] -- 1:52:12      9840000 -- (-7551.730) [...7 remote chains...] -- 1:52:08      9850000 -- (-7571.699) [...7 remote chains...] -- 1:52:04       Average standard deviation of split frequencies: 0.014877       9860000 -- (-7572.016) [...7 remote chains...] -- 1:52:02      9870000 -- (-7568.484) [...7 remote chains...] -- 1:51:58      9880000 -- (-7553.062) [...7 remote chains...] -- 1:51:54      9890000 -- (-7559.323) [...7 remote chains...] -- 1:51:50      9900000 -- (-7571.238) [...7 remote chains...] -- 1:51:48       Average standard deviation of split frequencies: 0.014659       9910000 -- (-7561.725) [...7 remote chains...] -- 1:51:44      9920000 -- (-7558.995) [...7 remote chains...] -- 1:51:40      9930000 -- (-7560.794) [...7 remote chains...] -- 1:51:38      9940000 -- (-7554.776) [...7 remote chains...] -- 1:51:34      9950000 -- (-7571.485) [...7 remote chains...] -- 1:51:30       Average standard deviation of split frequencies: 0.014754       9960000 -- (-7537.419) [...7 remote chains...] -- 1:51:28      9970000 -- (-7563.247) [...7 remote chains...] -- 1:51:24      9980000 -- (-7551.633) [...7 remote chains...] -- 1:51:20      9990000 -- (-7551.340) [...7 remote chains...] -- 1:51:16      10000000 -- (-7546.487) [...7 remote chains...] -- 1:51:14       Average standard deviation of split frequencies: 0.014802       10010000 -- (-7564.758) [...7 remote chains...] -- 1:51:09      10020000 -- (-7568.298) [...7 remote chains...] -- 1:51:05      10030000 -- (-7582.037) [...7 remote chains...] -- 1:51:03      10040000 -- (-7592.377) [...7 remote chains...] -- 1:50:59      10050000 -- (-7571.076) [...7 remote chains...] -- 1:50:55       Average standard deviation of split frequencies: 0.014644       10060000 -- (-7548.006) [...7 remote chains...] -- 1:50:53      10070000 -- (-7545.798) [...7 remote chains...] -- 1:50:49      10080000 -- (-7558.604) [...7 remote chains...] -- 1:50:45      10090000 -- (-7558.805) [...7 remote chains...] -- 1:50:41      10100000 -- (-7551.691) [...7 remote chains...] -- 1:50:39       Average standard deviation of split frequencies: 0.014807       10110000 -- (-7564.972) [...7 remote chains...] -- 1:50:35      10120000 -- (-7569.440) [...7 remote chains...] -- 1:50:31      10130000 -- (-7594.451) [...7 remote chains...] -- 1:50:29      10140000 -- (-7563.758) [...7 remote chains...] -- 1:50:25      10150000 -- (-7557.834) [...7 remote chains...] -- 1:50:21       Average standard deviation of split frequencies: 0.014814       10160000 -- (-7568.282) [...7 remote chains...] -- 1:50:17      10170000 -- (-7555.963) [...7 remote chains...] -- 1:50:15      10180000 -- (-7548.314) [...7 remote chains...] -- 1:50:11      10190000 -- (-7555.270) [...7 remote chains...] -- 1:50:07      10200000 -- (-7534.446) [...7 remote chains...] -- 1:50:05       Average standard deviation of split frequencies: 0.014758       10210000 -- (-7558.041) [...7 remote chains...] -- 1:50:01      10220000 -- (-7575.055) [...7 remote chains...] -- 1:49:57      10230000 -- (-7558.999) [...7 remote chains...] -- 1:49:53      10240000 -- (-7568.544) [...7 remote chains...] -- 1:49:51      10250000 -- (-7566.252) [...7 remote chains...] -- 1:49:47       Average standard deviation of split frequencies: 0.014708       10260000 -- (-7559.618) [...7 remote chains...] -- 1:49:43      10270000 -- (-7571.648) [...7 remote chains...] -- 1:49:41      10280000 -- (-7576.862) [...7 remote chains...] -- 1:49:37      10290000 -- (-7553.115) [...7 remote chains...] -- 1:49:33      10300000 -- (-7553.310) [...7 remote chains...] -- 1:49:29       Average standard deviation of split frequencies: 0.014447       10310000 -- (-7571.226) [...7 remote chains...] -- 1:49:27      10320000 -- (-7549.337) [...7 remote chains...] -- 1:49:23      10330000 -- (-7576.799) [...7 remote chains...] -- 1:49:19      10340000 -- (-7604.443) [...7 remote chains...] -- 1:49:17      10350000 -- (-7572.442) [...7 remote chains...] -- 1:49:13       Average standard deviation of split frequencies: 0.014337       10360000 -- (-7573.480) [...7 remote chains...] -- 1:49:09      10370000 -- (-7572.200) [...7 remote chains...] -- 1:49:07      10380000 -- (-7584.458) [...7 remote chains...] -- 1:49:03      10390000 -- (-7553.926) [...7 remote chains...] -- 1:48:59      10400000 -- (-7572.495) [...7 remote chains...] -- 1:48:55       Average standard deviation of split frequencies: 0.014360       10410000 -- (-7598.619) [...7 remote chains...] -- 1:48:53      10420000 -- (-7569.770) [...7 remote chains...] -- 1:48:49      10430000 -- (-7543.916) [...7 remote chains...] -- 1:48:45      10440000 -- (-7554.678) [...7 remote chains...] -- 1:48:43      10450000 -- (-7569.563) [...7 remote chains...] -- 1:48:39       Average standard deviation of split frequencies: 0.014167       10460000 -- (-7575.877) [...7 remote chains...] -- 1:48:35      10470000 -- [-7559.308] [...7 remote chains...] -- 1:48:31      10480000 -- (-7590.251) [...7 remote chains...] -- 1:48:29      10490000 -- [-7562.301] [...7 remote chains...] -- 1:48:25      10500000 -- [-7544.436] [...7 remote chains...] -- 1:48:21       Average standard deviation of split frequencies: 0.014041       10510000 -- [-7555.845] [...7 remote chains...] -- 1:48:19      10520000 -- [-7538.739] [...7 remote chains...] -- 1:48:15      10530000 -- [-7558.715] [...7 remote chains...] -- 1:48:11      10540000 -- [-7534.545] [...7 remote chains...] -- 1:48:07      10550000 -- [-7560.504] [...7 remote chains...] -- 1:48:05       Average standard deviation of split frequencies: 0.013827       10560000 -- (-7542.699) [...7 remote chains...] -- 1:48:01      10570000 -- [-7551.788] [...7 remote chains...] -- 1:47:57      10580000 -- [-7532.701] [...7 remote chains...] -- 1:47:55      10590000 -- [-7561.348] [...7 remote chains...] -- 1:47:51      10600000 -- [-7548.111] [...7 remote chains...] -- 1:47:47       Average standard deviation of split frequencies: 0.013507       10610000 -- [-7544.383] [...7 remote chains...] -- 1:47:45      10620000 -- [-7562.350] [...7 remote chains...] -- 1:47:41      10630000 -- [-7547.201] [...7 remote chains...] -- 1:47:37      10640000 -- [-7542.336] [...7 remote chains...] -- 1:47:35      10650000 -- [-7533.093] [...7 remote chains...] -- 1:47:31       Average standard deviation of split frequencies: 0.013549       10660000 -- [-7555.879] [...7 remote chains...] -- 1:47:27      10670000 -- [-7545.038] [...7 remote chains...] -- 1:47:23      10680000 -- (-7569.521) [...7 remote chains...] -- 1:47:21      10690000 -- [-7552.694] [...7 remote chains...] -- 1:47:17      10700000 -- [-7553.539] [...7 remote chains...] -- 1:47:13       Average standard deviation of split frequencies: 0.013374       10710000 -- (-7558.833) [...7 remote chains...] -- 1:47:10      10720000 -- [-7541.697] [...7 remote chains...] -- 1:47:07      10730000 -- [-7545.097] [...7 remote chains...] -- 1:47:03      10740000 -- [-7548.684] [...7 remote chains...] -- 1:47:00      10750000 -- [-7539.667] [...7 remote chains...] -- 1:46:56       Average standard deviation of split frequencies: 0.013557       10760000 -- [-7560.978] [...7 remote chains...] -- 1:46:53      10770000 -- [-7555.542] [...7 remote chains...] -- 1:46:50      10780000 -- [-7561.522] [...7 remote chains...] -- 1:46:46      10790000 -- [-7558.683] [...7 remote chains...] -- 1:46:43      10800000 -- [-7544.222] [...7 remote chains...] -- 1:46:40       Average standard deviation of split frequencies: 0.013470       10810000 -- (-7557.203) [...7 remote chains...] -- 1:46:36      10820000 -- (-7565.556) [...7 remote chains...] -- 1:46:32      10830000 -- (-7547.688) [...7 remote chains...] -- 1:46:30      10840000 -- [-7572.762] [...7 remote chains...] -- 1:46:26      10850000 -- [-7568.893] [...7 remote chains...] -- 1:46:22       Average standard deviation of split frequencies: 0.013403       10860000 -- [-7571.278] [...7 remote chains...] -- 1:46:20      10870000 -- [-7555.376] [...7 remote chains...] -- 1:46:16      10880000 -- (-7563.826) [...7 remote chains...] -- 1:46:12      10890000 -- [-7560.379] [...7 remote chains...] -- 1:46:10      10900000 -- [-7557.175] [...7 remote chains...] -- 1:46:06       Average standard deviation of split frequencies: 0.013506       10910000 -- [-7547.349] [...7 remote chains...] -- 1:46:02      10920000 -- [-7551.696] [...7 remote chains...] -- 1:45:58      10930000 -- (-7557.642) [...7 remote chains...] -- 1:45:56      10940000 -- [-7552.696] [...7 remote chains...] -- 1:45:52      10950000 -- [-7559.556] [...7 remote chains...] -- 1:45:48       Average standard deviation of split frequencies: 0.013592       10960000 -- [-7559.669] [...7 remote chains...] -- 1:45:46      10970000 -- (-7550.259) [...7 remote chains...] -- 1:45:42      10980000 -- [-7550.650] [...7 remote chains...] -- 1:45:38      10990000 -- [-7563.602] [...7 remote chains...] -- 1:45:36      11000000 -- [-7554.621] [...7 remote chains...] -- 1:45:32       Average standard deviation of split frequencies: 0.013576       11010000 -- [-7567.440] [...7 remote chains...] -- 1:45:28      11020000 -- [-7566.268] [...7 remote chains...] -- 1:45:26      11030000 -- [-7561.541] [...7 remote chains...] -- 1:45:22      11040000 -- [-7553.687] [...7 remote chains...] -- 1:45:18      11050000 -- [-7551.013] [...7 remote chains...] -- 1:45:14       Average standard deviation of split frequencies: 0.013476       11060000 -- [-7557.793] [...7 remote chains...] -- 1:45:12      11070000 -- [-7557.708] [...7 remote chains...] -- 1:45:08      11080000 -- [-7554.081] [...7 remote chains...] -- 1:45:04      11090000 -- [-7544.224] [...7 remote chains...] -- 1:45:02      11100000 -- [-7547.740] [...7 remote chains...] -- 1:44:58       Average standard deviation of split frequencies: 0.013358       11110000 -- (-7556.162) [...7 remote chains...] -- 1:44:54      11120000 -- (-7548.472) [...7 remote chains...] -- 1:44:52      11130000 -- (-7566.350) [...7 remote chains...] -- 1:44:48      11140000 -- (-7554.160) [...7 remote chains...] -- 1:44:44      11150000 -- (-7539.052) [...7 remote chains...] -- 1:44:40       Average standard deviation of split frequencies: 0.013159       11160000 -- (-7564.859) [...7 remote chains...] -- 1:44:38      11170000 -- (-7566.127) [...7 remote chains...] -- 1:44:34      11180000 -- (-7558.773) [...7 remote chains...] -- 1:44:30      11190000 -- (-7548.620) [...7 remote chains...] -- 1:44:26      11200000 -- (-7569.904) [...7 remote chains...] -- 1:44:24       Average standard deviation of split frequencies: 0.013111       11210000 -- (-7562.247) [...7 remote chains...] -- 1:44:20      11220000 -- (-7587.502) [...7 remote chains...] -- 1:44:16      11230000 -- (-7569.309) [...7 remote chains...] -- 1:44:12      11240000 -- (-7566.350) [...7 remote chains...] -- 1:44:10      11250000 -- (-7589.402) [...7 remote chains...] -- 1:44:06       Average standard deviation of split frequencies: 0.012724       11260000 -- (-7582.645) [...7 remote chains...] -- 1:44:02      11270000 -- (-7557.235) [...7 remote chains...] -- 1:44:00      11280000 -- (-7568.749) [...7 remote chains...] -- 1:43:56      11290000 -- (-7576.125) [...7 remote chains...] -- 1:43:52      11300000 -- (-7573.661) [...7 remote chains...] -- 1:43:48       Average standard deviation of split frequencies: 0.012775       11310000 -- (-7562.057) [...7 remote chains...] -- 1:43:46      11320000 -- (-7556.617) [...7 remote chains...] -- 1:43:42      11330000 -- (-7567.692) [...7 remote chains...] -- 1:43:38      11340000 -- (-7587.040) [...7 remote chains...] -- 1:43:36      11350000 -- (-7596.704) [...7 remote chains...] -- 1:43:32       Average standard deviation of split frequencies: 0.012685       11360000 -- (-7577.360) [...7 remote chains...] -- 1:43:28      11370000 -- (-7563.281) [...7 remote chains...] -- 1:43:25      11380000 -- (-7580.280) [...7 remote chains...] -- 1:43:22      11390000 -- (-7555.848) [...7 remote chains...] -- 1:43:18      11400000 -- (-7571.608) [...7 remote chains...] -- 1:43:15       Average standard deviation of split frequencies: 0.012416       11410000 -- (-7574.049) [...7 remote chains...] -- 1:43:12      11420000 -- (-7553.464) [...7 remote chains...] -- 1:43:08      11430000 -- (-7589.581) [...7 remote chains...] -- 1:43:05      11440000 -- (-7581.601) [...7 remote chains...] -- 1:43:01      11450000 -- (-7574.327) [...7 remote chains...] -- 1:42:59       Average standard deviation of split frequencies: 0.012249       11460000 -- (-7561.200) [...7 remote chains...] -- 1:42:55      11470000 -- (-7579.785) [...7 remote chains...] -- 1:42:51      11480000 -- (-7559.490) [...7 remote chains...] -- 1:42:49      11490000 -- (-7542.331) [...7 remote chains...] -- 1:42:45      11500000 -- (-7557.341) [...7 remote chains...] -- 1:42:41       Average standard deviation of split frequencies: 0.012234       11510000 -- (-7557.489) [...7 remote chains...] -- 1:42:37      11520000 -- (-7557.481) [...7 remote chains...] -- 1:42:35      11530000 -- (-7549.225) [...7 remote chains...] -- 1:42:31      11540000 -- (-7555.523) [...7 remote chains...] -- 1:42:27      11550000 -- (-7569.191) [...7 remote chains...] -- 1:42:25       Average standard deviation of split frequencies: 0.012145       11560000 -- (-7575.350) [...7 remote chains...] -- 1:42:21      11570000 -- [-7568.468] [...7 remote chains...] -- 1:42:17      11580000 -- [-7543.444] [...7 remote chains...] -- 1:42:15      11590000 -- (-7551.521) [...7 remote chains...] -- 1:42:11      11600000 -- [-7586.517] [...7 remote chains...] -- 1:42:07       Average standard deviation of split frequencies: 0.012109       11610000 -- [-7559.377] [...7 remote chains...] -- 1:42:03      11620000 -- [-7553.930] [...7 remote chains...] -- 1:42:01      11630000 -- (-7539.060) [...7 remote chains...] -- 1:41:57      11640000 -- (-7555.046) [...7 remote chains...] -- 1:41:53      11650000 -- (-7544.921) [...7 remote chains...] -- 1:41:49       Average standard deviation of split frequencies: 0.011976       11660000 -- [-7557.440] [...7 remote chains...] -- 1:41:47      11670000 -- [-7548.381] [...7 remote chains...] -- 1:41:43      11680000 -- (-7567.711) [...7 remote chains...] -- 1:41:39      11690000 -- [-7562.837] [...7 remote chains...] -- 1:41:37      11700000 -- [-7555.982] [...7 remote chains...] -- 1:41:33       Average standard deviation of split frequencies: 0.011868       11710000 -- [-7562.762] [...7 remote chains...] -- 1:41:29      11720000 -- (-7562.185) [...7 remote chains...] -- 1:41:27      11730000 -- [-7567.135] [...7 remote chains...] -- 1:41:23      11740000 -- [-7541.752] [...7 remote chains...] -- 1:41:19      11750000 -- [-7550.773] [...7 remote chains...] -- 1:41:17       Average standard deviation of split frequencies: 0.011668       11760000 -- [-7541.251] [...7 remote chains...] -- 1:41:13      11770000 -- (-7548.010) [...7 remote chains...] -- 1:41:09      11780000 -- [-7551.316] [...7 remote chains...] -- 1:41:06      11790000 -- [-7552.639] [...7 remote chains...] -- 1:41:03      11800000 -- [-7537.910] [...7 remote chains...] -- 1:40:59       Average standard deviation of split frequencies: 0.011477       11810000 -- [-7547.595] [...7 remote chains...] -- 1:40:56      11820000 -- [-7547.939] [...7 remote chains...] -- 1:40:52      11830000 -- (-7550.960) [...7 remote chains...] -- 1:40:50      11840000 -- [-7556.675] [...7 remote chains...] -- 1:40:46      11850000 -- (-7567.154) [...7 remote chains...] -- 1:40:42       Average standard deviation of split frequencies: 0.011401       11860000 -- (-7552.505) [...7 remote chains...] -- 1:40:40      11870000 -- (-7559.721) [...7 remote chains...] -- 1:40:36      11880000 -- (-7559.409) [...7 remote chains...] -- 1:40:32      11890000 -- (-7564.324) [...7 remote chains...] -- 1:40:28      11900000 -- (-7589.145) [...7 remote chains...] -- 1:40:26       Average standard deviation of split frequencies: 0.011191       11910000 -- (-7567.987) [...7 remote chains...] -- 1:40:22      11920000 -- (-7547.958) [...7 remote chains...] -- 1:40:18      11930000 -- (-7553.233) [...7 remote chains...] -- 1:40:16      11940000 -- (-7553.494) [...7 remote chains...] -- 1:40:12      11950000 -- (-7564.996) [...7 remote chains...] -- 1:40:08       Average standard deviation of split frequencies: 0.011161       11960000 -- (-7556.847) [...7 remote chains...] -- 1:40:04      11970000 -- (-7583.374) [...7 remote chains...] -- 1:40:02      11980000 -- [-7550.071] [...7 remote chains...] -- 1:39:58      11990000 -- [-7555.216] [...7 remote chains...] -- 1:39:54      12000000 -- [-7552.358] [...7 remote chains...] -- 1:39:51       Average standard deviation of split frequencies: 0.011011       12010000 -- [-7541.748] [...7 remote chains...] -- 1:39:48      12020000 -- [-7558.453] [...7 remote chains...] -- 1:39:44      12030000 -- [-7543.385] [...7 remote chains...] -- 1:39:41      12040000 -- [-7551.126] [...7 remote chains...] -- 1:39:38      12050000 -- (-7548.101) [...7 remote chains...] -- 1:39:34       Average standard deviation of split frequencies: 0.010987       12060000 -- (-7570.341) [...7 remote chains...] -- 1:39:31      12070000 -- (-7562.937) [...7 remote chains...] -- 1:39:28      12080000 -- [-7560.568] [...7 remote chains...] -- 1:39:24      12090000 -- [-7574.529] [...7 remote chains...] -- 1:39:21      12100000 -- [-7551.339] [...7 remote chains...] -- 1:39:17       Average standard deviation of split frequencies: 0.010800       12110000 -- (-7564.712) [...7 remote chains...] -- 1:39:14      12120000 -- (-7560.137) [...7 remote chains...] -- 1:39:11      12130000 -- [-7556.880] [...7 remote chains...] -- 1:39:07      12140000 -- (-7572.504) [...7 remote chains...] -- 1:39:04      12150000 -- (-7561.061) [...7 remote chains...] -- 1:39:01       Average standard deviation of split frequencies: 0.010669       12160000 -- (-7560.771) [...7 remote chains...] -- 1:38:57      12170000 -- (-7555.880) [...7 remote chains...] -- 1:38:53      12180000 -- (-7571.823) [...7 remote chains...] -- 1:38:51      12190000 -- (-7573.234) [...7 remote chains...] -- 1:38:47      12200000 -- (-7561.117) [...7 remote chains...] -- 1:38:43       Average standard deviation of split frequencies: 0.010719       12210000 -- (-7546.347) [...7 remote chains...] -- 1:38:39      12220000 -- (-7567.006) [...7 remote chains...] -- 1:38:37      12230000 -- (-7556.787) [...7 remote chains...] -- 1:38:33      12240000 -- [-7579.652] [...7 remote chains...] -- 1:38:29      12250000 -- (-7535.961) [...7 remote chains...] -- 1:38:26       Average standard deviation of split frequencies: 0.010643       12260000 -- (-7561.707) [...7 remote chains...] -- 1:38:22      12270000 -- (-7573.652) [...7 remote chains...] -- 1:38:19      12280000 -- (-7561.875) [...7 remote chains...] -- 1:38:16      12290000 -- (-7554.896) [...7 remote chains...] -- 1:38:12      12300000 -- (-7547.605) [...7 remote chains...] -- 1:38:09       Average standard deviation of split frequencies: 0.010418       12310000 -- [-7546.531] [...7 remote chains...] -- 1:38:06      12320000 -- [-7534.794] [...7 remote chains...] -- 1:38:02      12330000 -- (-7545.621) [...7 remote chains...] -- 1:37:58      12340000 -- [-7536.929] [...7 remote chains...] -- 1:37:56      12350000 -- (-7564.664) [...7 remote chains...] -- 1:37:52       Average standard deviation of split frequencies: 0.010229       12360000 -- (-7594.861) [...7 remote chains...] -- 1:37:48      12370000 -- (-7581.003) [...7 remote chains...] -- 1:37:46      12380000 -- (-7551.155) [...7 remote chains...] -- 1:37:42      12390000 -- (-7554.376) [...7 remote chains...] -- 1:37:38      12400000 -- (-7549.265) [...7 remote chains...] -- 1:37:34       Average standard deviation of split frequencies: 0.010133       12410000 -- (-7562.630) [...7 remote chains...] -- 1:37:32      12420000 -- (-7558.996) [...7 remote chains...] -- 1:37:28      12430000 -- (-7549.466) [...7 remote chains...] -- 1:37:24      12440000 -- (-7569.440) [...7 remote chains...] -- 1:37:22      12450000 -- (-7564.664) [...7 remote chains...] -- 1:37:18       Average standard deviation of split frequencies: 0.010232       12460000 -- (-7551.937) [...7 remote chains...] -- 1:37:14      12470000 -- (-7557.413) [...7 remote chains...] -- 1:37:11      12480000 -- (-7567.473) [...7 remote chains...] -- 1:37:08      12490000 -- (-7570.009) [...7 remote chains...] -- 1:37:04      12500000 -- (-7567.664) [...7 remote chains...] -- 1:37:01       Average standard deviation of split frequencies: 0.010442       12510000 -- (-7572.065) [...7 remote chains...] -- 1:36:58      12520000 -- (-7573.135) [...7 remote chains...] -- 1:36:55      12530000 -- (-7583.150) [...7 remote chains...] -- 1:36:51      12540000 -- (-7547.443) [...7 remote chains...] -- 1:36:47      12550000 -- (-7552.429) [...7 remote chains...] -- 1:36:45       Average standard deviation of split frequencies: 0.010512       12560000 -- (-7548.556) [...7 remote chains...] -- 1:36:41      12570000 -- (-7551.335) [...7 remote chains...] -- 1:36:37      12580000 -- (-7577.044) [...7 remote chains...] -- 1:36:33      12590000 -- (-7568.657) [...7 remote chains...] -- 1:36:31      12600000 -- (-7561.982) [...7 remote chains...] -- 1:36:27       Average standard deviation of split frequencies: 0.010463       12610000 -- (-7555.867) [...7 remote chains...] -- 1:36:23      12620000 -- (-7561.019) [...7 remote chains...] -- 1:36:21      12630000 -- (-7549.112) [...7 remote chains...] -- 1:36:17      12640000 -- (-7558.875) [...7 remote chains...] -- 1:36:13      12650000 -- (-7559.469) [...7 remote chains...] -- 1:36:10       Average standard deviation of split frequencies: 0.010411       12660000 -- (-7563.484) [...7 remote chains...] -- 1:36:07      12670000 -- (-7554.745) [...7 remote chains...] -- 1:36:03      12680000 -- (-7555.372) [...7 remote chains...] -- 1:36:00      12690000 -- (-7543.806) [...7 remote chains...] -- 1:35:56      12700000 -- [-7543.205] [...7 remote chains...] -- 1:35:53       Average standard deviation of split frequencies: 0.010323       12710000 -- [-7546.176] [...7 remote chains...] -- 1:35:50      12720000 -- (-7534.497) [...7 remote chains...] -- 1:35:46      12730000 -- (-7542.170) [...7 remote chains...] -- 1:35:42      12740000 -- (-7557.383) [...7 remote chains...] -- 1:35:40      12750000 -- (-7556.785) [...7 remote chains...] -- 1:35:36       Average standard deviation of split frequencies: 0.010232       12760000 -- (-7548.645) [...7 remote chains...] -- 1:35:32      12770000 -- [-7565.758] [...7 remote chains...] -- 1:35:28      12780000 -- [-7553.375] [...7 remote chains...] -- 1:35:26      12790000 -- (-7545.985) [...7 remote chains...] -- 1:35:22      12800000 -- (-7561.441) [...7 remote chains...] -- 1:35:19       Average standard deviation of split frequencies: 0.010156       12810000 -- (-7542.217) [...7 remote chains...] -- 1:35:15      12820000 -- (-7549.704) [...7 remote chains...] -- 1:35:12      12830000 -- (-7568.477) [...7 remote chains...] -- 1:35:09      12840000 -- (-7544.555) [...7 remote chains...] -- 1:35:05      12850000 -- (-7570.147) [...7 remote chains...] -- 1:35:01       Average standard deviation of split frequencies: 0.010002       12860000 -- (-7568.417) [...7 remote chains...] -- 1:34:59      12870000 -- (-7568.927) [...7 remote chains...] -- 1:34:55      12880000 -- (-7564.715) [...7 remote chains...] -- 1:34:51      12890000 -- [-7540.831] [...7 remote chains...] -- 1:34:47      12900000 -- (-7535.970) [...7 remote chains...] -- 1:34:45       Average standard deviation of split frequencies: 0.009998       12910000 -- [-7543.654] [...7 remote chains...] -- 1:34:41      12920000 -- [-7542.263] [...7 remote chains...] -- 1:34:37      12930000 -- [-7548.236] [...7 remote chains...] -- 1:34:35      12940000 -- [-7539.617] [...7 remote chains...] -- 1:34:31      12950000 -- [-7537.572] [...7 remote chains...] -- 1:34:27       Average standard deviation of split frequencies: 0.010076       12960000 -- [-7537.342] [...7 remote chains...] -- 1:34:25      12970000 -- (-7549.619) [...7 remote chains...] -- 1:34:21      12980000 -- [-7546.034] [...7 remote chains...] -- 1:34:18      12990000 -- [-7551.153] [...7 remote chains...] -- 1:34:14      13000000 -- [-7539.266] [...7 remote chains...] -- 1:34:11       Average standard deviation of split frequencies: 0.010125       13010000 -- [-7552.777] [...7 remote chains...] -- 1:34:08      13020000 -- [-7553.466] [...7 remote chains...] -- 1:34:04      13030000 -- [-7546.589] [...7 remote chains...] -- 1:34:00      13040000 -- [-7552.376] [...7 remote chains...] -- 1:33:56      13050000 -- [-7545.903] [...7 remote chains...] -- 1:33:54       Average standard deviation of split frequencies: 0.010067       13060000 -- [-7577.999] [...7 remote chains...] -- 1:33:50      13070000 -- [-7564.786] [...7 remote chains...] -- 1:33:46      13080000 -- [-7529.293] [...7 remote chains...] -- 1:33:44      13090000 -- [-7551.947] [...7 remote chains...] -- 1:33:40      13100000 -- [-7568.946] [...7 remote chains...] -- 1:33:36       Average standard deviation of split frequencies: 0.010011       13110000 -- [-7574.911] [...7 remote chains...] -- 1:33:34      13120000 -- [-7555.856] [...7 remote chains...] -- 1:33:30      13130000 -- [-7551.494] [...7 remote chains...] -- 1:33:27      13140000 -- [-7543.640] [...7 remote chains...] -- 1:33:23      13150000 -- [-7560.985] [...7 remote chains...] -- 1:33:20       Average standard deviation of split frequencies: 0.009859       13160000 -- [-7571.876] [...7 remote chains...] -- 1:33:17      13170000 -- [-7571.255] [...7 remote chains...] -- 1:33:13      13180000 -- [-7550.634] [...7 remote chains...] -- 1:33:09      13190000 -- [-7547.034] [...7 remote chains...] -- 1:33:07      13200000 -- [-7564.333] [...7 remote chains...] -- 1:33:03       Average standard deviation of split frequencies: 0.009859       13210000 -- [-7547.296] [...7 remote chains...] -- 1:32:59      13220000 -- [-7551.786] [...7 remote chains...] -- 1:32:55      13230000 -- [-7552.188] [...7 remote chains...] -- 1:32:53      13240000 -- [-7567.618] [...7 remote chains...] -- 1:32:49      13250000 -- [-7555.930] [...7 remote chains...] -- 1:32:46       Average standard deviation of split frequencies: 0.009752       13260000 -- (-7551.690) [...7 remote chains...] -- 1:32:42      13270000 -- (-7554.512) [...7 remote chains...] -- 1:32:39      13280000 -- (-7567.506) [...7 remote chains...] -- 1:32:36      13290000 -- (-7551.353) [...7 remote chains...] -- 1:32:32      13300000 -- [-7561.611] [...7 remote chains...] -- 1:32:29       Average standard deviation of split frequencies: 0.009762       13310000 -- (-7567.980) [...7 remote chains...] -- 1:32:26      13320000 -- (-7561.266) [...7 remote chains...] -- 1:32:22      13330000 -- [-7563.927] [...7 remote chains...] -- 1:32:18      13340000 -- (-7569.349) [...7 remote chains...] -- 1:32:16      13350000 -- (-7542.892) [...7 remote chains...] -- 1:32:12       Average standard deviation of split frequencies: 0.009831       13360000 -- [-7551.399] [...7 remote chains...] -- 1:32:08      13370000 -- [-7562.777] [...7 remote chains...] -- 1:32:05      13380000 -- (-7558.322) [...7 remote chains...] -- 1:32:02      13390000 -- (-7566.770) [...7 remote chains...] -- 1:31:58      13400000 -- [-7539.859] [...7 remote chains...] -- 1:31:55       Average standard deviation of split frequencies: 0.010103       13410000 -- (-7558.943) [...7 remote chains...] -- 1:31:51      13420000 -- (-7567.201) [...7 remote chains...] -- 1:31:48      13430000 -- [-7555.821] [...7 remote chains...] -- 1:31:45      13440000 -- (-7544.116) [...7 remote chains...] -- 1:31:41      13450000 -- (-7540.643) [...7 remote chains...] -- 1:31:37       Average standard deviation of split frequencies: 0.010056       13460000 -- (-7534.604) [...7 remote chains...] -- 1:31:35      13470000 -- (-7546.898) [...7 remote chains...] -- 1:31:31      13480000 -- (-7558.485) [...7 remote chains...] -- 1:31:27      13490000 -- (-7548.014) [...7 remote chains...] -- 1:31:25      13500000 -- (-7556.185) [...7 remote chains...] -- 1:31:21       Average standard deviation of split frequencies: 0.010109       13510000 -- (-7559.049) [...7 remote chains...] -- 1:31:17      13520000 -- (-7560.799) [...7 remote chains...] -- 1:31:14      13530000 -- (-7561.825) [...7 remote chains...] -- 1:31:11      13540000 -- (-7552.172) [...7 remote chains...] -- 1:31:08      13550000 -- (-7563.613) [...7 remote chains...] -- 1:31:04       Average standard deviation of split frequencies: 0.010147       13560000 -- (-7558.622) [...7 remote chains...] -- 1:31:00      13570000 -- (-7535.172) [...7 remote chains...] -- 1:30:58      13580000 -- (-7561.004) [...7 remote chains...] -- 1:30:54      13590000 -- (-7562.579) [...7 remote chains...] -- 1:30:50      13600000 -- (-7568.551) [...7 remote chains...] -- 1:30:46       Average standard deviation of split frequencies: 0.010116       13610000 -- (-7568.043) [...7 remote chains...] -- 1:30:44      13620000 -- (-7554.223) [...7 remote chains...] -- 1:30:40      13630000 -- (-7556.031) [...7 remote chains...] -- 1:30:37      13640000 -- (-7549.234) [...7 remote chains...] -- 1:30:33      13650000 -- (-7560.705) [...7 remote chains...] -- 1:30:30       Average standard deviation of split frequencies: 0.010051       13660000 -- (-7544.487) [...7 remote chains...] -- 1:30:27      13670000 -- (-7566.244) [...7 remote chains...] -- 1:30:23      13680000 -- (-7562.961) [...7 remote chains...] -- 1:30:19      13690000 -- (-7571.031) [...7 remote chains...] -- 1:30:17      13700000 -- (-7572.483) [...7 remote chains...] -- 1:30:13       Average standard deviation of split frequencies: 0.010169       13710000 -- (-7560.969) [...7 remote chains...] -- 1:30:09      13720000 -- (-7569.173) [...7 remote chains...] -- 1:30:06      13730000 -- (-7554.407) [...7 remote chains...] -- 1:30:02      13740000 -- (-7558.390) [...7 remote chains...] -- 1:29:59      13750000 -- (-7549.613) [...7 remote chains...] -- 1:29:56       Average standard deviation of split frequencies: 0.010264       13760000 -- (-7557.353) [...7 remote chains...] -- 1:29:52      13770000 -- (-7561.278) [...7 remote chains...] -- 1:29:49      13780000 -- (-7561.447) [...7 remote chains...] -- 1:29:46      13790000 -- (-7561.739) [...7 remote chains...] -- 1:29:42      13800000 -- (-7567.176) [...7 remote chains...] -- 1:29:40       Average standard deviation of split frequencies: 0.010034       13810000 -- (-7551.848) [...7 remote chains...] -- 1:29:36      13820000 -- (-7574.456) [...7 remote chains...] -- 1:29:32      13830000 -- (-7558.061) [...7 remote chains...] -- 1:29:28      13840000 -- (-7557.893) [...7 remote chains...] -- 1:29:26      13850000 -- (-7576.915) [...7 remote chains...] -- 1:29:22       Average standard deviation of split frequencies: 0.009902       13860000 -- (-7567.623) [...7 remote chains...] -- 1:29:19      13870000 -- (-7541.889) [...7 remote chains...] -- 1:29:15      13880000 -- (-7542.744) [...7 remote chains...] -- 1:29:12      13890000 -- (-7566.969) [...7 remote chains...] -- 1:29:09      13900000 -- (-7560.621) [...7 remote chains...] -- 1:29:05       Average standard deviation of split frequencies: 0.009720       13910000 -- (-7559.344) [...7 remote chains...] -- 1:29:01      13920000 -- (-7560.296) [...7 remote chains...] -- 1:28:59      13930000 -- (-7544.971) [...7 remote chains...] -- 1:28:55      13940000 -- (-7555.014) [...7 remote chains...] -- 1:28:51      13950000 -- (-7570.317) [...7 remote chains...] -- 1:28:48       Average standard deviation of split frequencies: 0.009564       13960000 -- (-7563.750) [...7 remote chains...] -- 1:28:45      13970000 -- (-7554.682) [...7 remote chains...] -- 1:28:41      13980000 -- (-7559.876) [...7 remote chains...] -- 1:28:38      13990000 -- (-7561.247) [...7 remote chains...] -- 1:28:34      14000000 -- (-7558.216) [...7 remote chains...] -- 1:28:32       Average standard deviation of split frequencies: 0.009516       14010000 -- (-7571.674) [...7 remote chains...] -- 1:28:28      14020000 -- (-7576.978) [...7 remote chains...] -- 1:28:24      14030000 -- (-7558.936) [...7 remote chains...] -- 1:28:20      14040000 -- (-7561.318) [...7 remote chains...] -- 1:28:17      14050000 -- (-7559.691) [...7 remote chains...] -- 1:28:14       Average standard deviation of split frequencies: 0.009829       14060000 -- (-7552.587) [...7 remote chains...] -- 1:28:11      14070000 -- (-7570.135) [...7 remote chains...] -- 1:28:07      14080000 -- (-7557.326) [...7 remote chains...] -- 1:28:04      14090000 -- (-7565.609) [...7 remote chains...] -- 1:28:01      14100000 -- (-7585.199) [...7 remote chains...] -- 1:27:57       Average standard deviation of split frequencies: 0.009791       14110000 -- (-7545.032) [...7 remote chains...] -- 1:27:53      14120000 -- (-7544.097) [...7 remote chains...] -- 1:27:51      14130000 -- (-7554.545) [...7 remote chains...] -- 1:27:47      14140000 -- (-7569.077) [...7 remote chains...] -- 1:27:43      14150000 -- (-7583.521) [...7 remote chains...] -- 1:27:40       Average standard deviation of split frequencies: 0.009705       14160000 -- (-7599.918) [...7 remote chains...] -- 1:27:37      14170000 -- (-7561.181) [...7 remote chains...] -- 1:27:33      14180000 -- (-7570.935) [...7 remote chains...] -- 1:27:30      14190000 -- (-7550.674) [...7 remote chains...] -- 1:27:27      14200000 -- (-7563.408) [...7 remote chains...] -- 1:27:24       Average standard deviation of split frequencies: 0.009782       14210000 -- (-7566.586) [...7 remote chains...] -- 1:27:20      14220000 -- (-7553.379) [...7 remote chains...] -- 1:27:16      14230000 -- (-7560.989) [...7 remote chains...] -- 1:27:14      14240000 -- (-7550.121) [...7 remote chains...] -- 1:27:10      14250000 -- (-7577.033) [...7 remote chains...] -- 1:27:06       Average standard deviation of split frequencies: 0.009872       14260000 -- (-7569.369) [...7 remote chains...] -- 1:27:03      14270000 -- (-7562.804) [...7 remote chains...] -- 1:27:00      14280000 -- (-7541.977) [...7 remote chains...] -- 1:26:56      14290000 -- (-7556.897) [...7 remote chains...] -- 1:26:53      14300000 -- (-7541.499) [...7 remote chains...] -- 1:26:50       Average standard deviation of split frequencies: 0.009803       14310000 -- (-7564.550) [...7 remote chains...] -- 1:26:46      14320000 -- (-7593.959) [...7 remote chains...] -- 1:26:43      14330000 -- (-7572.485) [...7 remote chains...] -- 1:26:40      14340000 -- (-7553.983) [...7 remote chains...] -- 1:26:37      14350000 -- (-7555.204) [...7 remote chains...] -- 1:26:33       Average standard deviation of split frequencies: 0.009732       14360000 -- (-7574.826) [...7 remote chains...] -- 1:26:29      14370000 -- (-7563.212) [...7 remote chains...] -- 1:26:27      14380000 -- (-7582.310) [...7 remote chains...] -- 1:26:23      14390000 -- (-7572.016) [...7 remote chains...] -- 1:26:19      14400000 -- (-7561.447) [...7 remote chains...] -- 1:26:16       Average standard deviation of split frequencies: 0.009761       14410000 -- (-7581.552) [...7 remote chains...] -- 1:26:13      14420000 -- (-7583.505) [...7 remote chains...] -- 1:26:09      14430000 -- (-7564.182) [...7 remote chains...] -- 1:26:06      14440000 -- (-7581.812) [...7 remote chains...] -- 1:26:02      14450000 -- (-7569.691) [...7 remote chains...] -- 1:26:00       Average standard deviation of split frequencies: 0.009845       14460000 -- (-7568.743) [...7 remote chains...] -- 1:25:56      14470000 -- (-7545.214) [...7 remote chains...] -- 1:25:52      14480000 -- (-7559.627) [...7 remote chains...] -- 1:25:50      14490000 -- (-7566.734) [...7 remote chains...] -- 1:25:46      14500000 -- (-7563.248) [...7 remote chains...] -- 1:25:42       Average standard deviation of split frequencies: 0.009834       14510000 -- (-7571.176) [...7 remote chains...] -- 1:25:39      14520000 -- (-7561.683) [...7 remote chains...] -- 1:25:36      14530000 -- (-7554.813) [...7 remote chains...] -- 1:25:32      14540000 -- (-7564.970) [...7 remote chains...] -- 1:25:29      14550000 -- (-7548.658) [...7 remote chains...] -- 1:25:25       Average standard deviation of split frequencies: 0.009881       14560000 -- (-7551.360) [...7 remote chains...] -- 1:25:22      14570000 -- (-7552.893) [...7 remote chains...] -- 1:25:19      14580000 -- (-7549.919) [...7 remote chains...] -- 1:25:15      14590000 -- (-7548.019) [...7 remote chains...] -- 1:25:12      14600000 -- (-7569.818) [...7 remote chains...] -- 1:25:09       Average standard deviation of split frequencies: 0.009833       14610000 -- (-7542.800) [...7 remote chains...] -- 1:25:05      14620000 -- (-7556.366) [...7 remote chains...] -- 1:25:02      14630000 -- (-7570.843) [...7 remote chains...] -- 1:24:58      14640000 -- (-7564.393) [...7 remote chains...] -- 1:24:55      14650000 -- (-7547.058) [...7 remote chains...] -- 1:24:52       Average standard deviation of split frequencies: 0.009923       14660000 -- (-7566.284) [...7 remote chains...] -- 1:24:48      14670000 -- (-7557.639) [...7 remote chains...] -- 1:24:44      14680000 -- (-7570.417) [...7 remote chains...] -- 1:24:41      14690000 -- (-7571.583) [...7 remote chains...] -- 1:24:38      14700000 -- (-7558.897) [...7 remote chains...] -- 1:24:35       Average standard deviation of split frequencies: 0.010086       14710000 -- (-7576.988) [...7 remote chains...] -- 1:24:31      14720000 -- (-7565.048) [...7 remote chains...] -- 1:24:27      14730000 -- (-7556.772) [...7 remote chains...] -- 1:24:25      14740000 -- (-7548.716) [...7 remote chains...] -- 1:24:21      14750000 -- (-7578.866) [...7 remote chains...] -- 1:24:17       Average standard deviation of split frequencies: 0.010201       14760000 -- (-7548.276) [...7 remote chains...] -- 1:24:15      14770000 -- (-7574.808) [...7 remote chains...] -- 1:24:11      14780000 -- (-7542.921) [...7 remote chains...] -- 1:24:07      14790000 -- (-7548.719) [...7 remote chains...] -- 1:24:04      14800000 -- (-7552.903) [...7 remote chains...] -- 1:24:00       Average standard deviation of split frequencies: 0.010203       14810000 -- (-7547.668) [...7 remote chains...] -- 1:23:58      14820000 -- (-7554.094) [...7 remote chains...] -- 1:23:54      14830000 -- (-7549.851) [...7 remote chains...] -- 1:23:50      14840000 -- (-7547.507) [...7 remote chains...] -- 1:23:47      14850000 -- (-7548.351) [...7 remote chains...] -- 1:23:44       Average standard deviation of split frequencies: 0.009995       14860000 -- (-7542.158) [...7 remote chains...] -- 1:23:40      14870000 -- (-7550.039) [...7 remote chains...] -- 1:23:37      14880000 -- (-7562.745) [...7 remote chains...] -- 1:23:34      14890000 -- (-7545.863) [...7 remote chains...] -- 1:23:30      14900000 -- (-7561.670) [...7 remote chains...] -- 1:23:27       Average standard deviation of split frequencies: 0.010071       14910000 -- (-7575.841) [...7 remote chains...] -- 1:23:23      14920000 -- (-7568.677) [...7 remote chains...] -- 1:23:21      14930000 -- (-7562.772) [...7 remote chains...] -- 1:23:17      14940000 -- (-7569.377) [...7 remote chains...] -- 1:23:13      14950000 -- (-7561.808) [...7 remote chains...] -- 1:23:11       Average standard deviation of split frequencies: 0.010163       14960000 -- (-7558.897) [...7 remote chains...] -- 1:23:07      14970000 -- (-7535.309) [...7 remote chains...] -- 1:23:03      14980000 -- (-7568.255) [...7 remote chains...] -- 1:23:00      14990000 -- (-7580.227) [...7 remote chains...] -- 1:22:56      15000000 -- (-7565.633) [...7 remote chains...] -- 1:22:54       Average standard deviation of split frequencies: 0.010290       15010000 -- (-7553.996) [...7 remote chains...] -- 1:22:50      15020000 -- (-7554.822) [...7 remote chains...] -- 1:22:46      15030000 -- (-7558.740) [...7 remote chains...] -- 1:22:43      15040000 -- (-7545.869) [...7 remote chains...] -- 1:22:40      15050000 -- (-7539.841) [...7 remote chains...] -- 1:22:36       Average standard deviation of split frequencies: 0.010234       15060000 -- (-7565.421) [...7 remote chains...] -- 1:22:33      15070000 -- (-7560.322) [...7 remote chains...] -- 1:22:29      15080000 -- (-7562.492) [...7 remote chains...] -- 1:22:26      15090000 -- (-7559.167) [...7 remote chains...] -- 1:22:23      15100000 -- (-7574.424) [...7 remote chains...] -- 1:22:19       Average standard deviation of split frequencies: 0.010406       15110000 -- (-7566.213) [...7 remote chains...] -- 1:22:16      15120000 -- (-7558.637) [...7 remote chains...] -- 1:22:13      15130000 -- (-7562.516) [...7 remote chains...] -- 1:22:09      15140000 -- (-7590.656) [...7 remote chains...] -- 1:22:06      15150000 -- (-7562.805) [...7 remote chains...] -- 1:22:02       Average standard deviation of split frequencies: 0.010020       15160000 -- (-7560.699) [...7 remote chains...] -- 1:21:58      15170000 -- (-7568.580) [...7 remote chains...] -- 1:21:56      15180000 -- (-7566.703) [...7 remote chains...] -- 1:21:52      15190000 -- (-7567.142) [...7 remote chains...] -- 1:21:49      15200000 -- (-7562.150) [...7 remote chains...] -- 1:21:45       Average standard deviation of split frequencies: 0.009779       15210000 -- (-7560.591) [...7 remote chains...] -- 1:21:42      15220000 -- (-7551.059) [...7 remote chains...] -- 1:21:39      15230000 -- (-7570.577) [...7 remote chains...] -- 1:21:35      15240000 -- (-7541.688) [...7 remote chains...] -- 1:21:31      15250000 -- [-7538.470] [...7 remote chains...] -- 1:21:29       Average standard deviation of split frequencies: 0.009606       15260000 -- [-7518.922] [...7 remote chains...] -- 1:21:25      15270000 -- [-7539.518] [...7 remote chains...] -- 1:21:22      15280000 -- [-7548.037] [...7 remote chains...] -- 1:21:19      15290000 -- [-7546.057] [...7 remote chains...] -- 1:21:15      15300000 -- [-7536.118] [...7 remote chains...] -- 1:21:12       Average standard deviation of split frequencies: 0.009455       15310000 -- [-7543.711] [...7 remote chains...] -- 1:21:08      15320000 -- (-7561.557) [...7 remote chains...] -- 1:21:05      15330000 -- (-7549.634) [...7 remote chains...] -- 1:21:02      15340000 -- (-7577.138) [...7 remote chains...] -- 1:20:58      15350000 -- (-7548.770) [...7 remote chains...] -- 1:20:55       Average standard deviation of split frequencies: 0.009425       15360000 -- (-7537.576) [...7 remote chains...] -- 1:20:52      15370000 -- (-7555.461) [...7 remote chains...] -- 1:20:48      15380000 -- [-7556.639] [...7 remote chains...] -- 1:20:45      15390000 -- (-7554.142) [...7 remote chains...] -- 1:20:41      15400000 -- (-7547.897) [...7 remote chains...] -- 1:20:38       Average standard deviation of split frequencies: 0.009404       15410000 -- (-7544.446) [...7 remote chains...] -- 1:20:35      15420000 -- [-7530.838] [...7 remote chains...] -- 1:20:31      15430000 -- [-7550.641] [...7 remote chains...] -- 1:20:28      15440000 -- [-7528.060] [...7 remote chains...] -- 1:20:25      15450000 -- [-7540.818] [...7 remote chains...] -- 1:20:21       Average standard deviation of split frequencies: 0.009234       15460000 -- [-7545.998] [...7 remote chains...] -- 1:20:18      15470000 -- (-7557.529) [...7 remote chains...] -- 1:20:15      15480000 -- [-7552.070] [...7 remote chains...] -- 1:20:11      15490000 -- [-7554.577] [...7 remote chains...] -- 1:20:08      15500000 -- [-7538.316] [...7 remote chains...] -- 1:20:04       Average standard deviation of split frequencies: 0.009145       15510000 -- [-7550.250] [...7 remote chains...] -- 1:20:01      15520000 -- [-7553.318] [...7 remote chains...] -- 1:19:58      15530000 -- [-7555.335] [...7 remote chains...] -- 1:19:54      15540000 -- [-7553.841] [...7 remote chains...] -- 1:19:51      15550000 -- [-7539.089] [...7 remote chains...] -- 1:19:48       Average standard deviation of split frequencies: 0.009359       15560000 -- [-7542.995] [...7 remote chains...] -- 1:19:44      15570000 -- [-7536.677] [...7 remote chains...] -- 1:19:41      15580000 -- [-7562.916] [...7 remote chains...] -- 1:19:37      15590000 -- (-7550.225) [...7 remote chains...] -- 1:19:34      15600000 -- [-7553.339] [...7 remote chains...] -- 1:19:31       Average standard deviation of split frequencies: 0.009828       15610000 -- [-7547.840] [...7 remote chains...] -- 1:19:27      15620000 -- [-7549.224] [...7 remote chains...] -- 1:19:24      15630000 -- (-7558.029) [...7 remote chains...] -- 1:19:21      15640000 -- [-7562.911] [...7 remote chains...] -- 1:19:17      15650000 -- (-7578.275) [...7 remote chains...] -- 1:19:14       Average standard deviation of split frequencies: 0.010170       15660000 -- [-7559.255] [...7 remote chains...] -- 1:19:10      15670000 -- [-7550.008] [...7 remote chains...] -- 1:19:08      15680000 -- (-7556.742) [...7 remote chains...] -- 1:19:04      15690000 -- (-7546.288) [...7 remote chains...] -- 1:19:00      15700000 -- (-7555.205) [...7 remote chains...] -- 1:18:57       Average standard deviation of split frequencies: 0.010236       15710000 -- (-7543.542) [...7 remote chains...] -- 1:18:53      15720000 -- (-7553.095) [...7 remote chains...] -- 1:18:50      15730000 -- (-7555.937) [...7 remote chains...] -- 1:18:47      15740000 -- (-7594.069) [...7 remote chains...] -- 1:18:43      15750000 -- (-7579.010) [...7 remote chains...] -- 1:18:40       Average standard deviation of split frequencies: 0.010232       15760000 -- (-7573.871) [...7 remote chains...] -- 1:18:37      15770000 -- [-7549.239] [...7 remote chains...] -- 1:18:33      15780000 -- [-7563.139] [...7 remote chains...] -- 1:18:30      15790000 -- [-7572.993] [...7 remote chains...] -- 1:18:26      15800000 -- [-7557.435] [...7 remote chains...] -- 1:18:23       Average standard deviation of split frequencies: 0.010069       15810000 -- [-7553.057] [...7 remote chains...] -- 1:18:20      15820000 -- [-7556.902] [...7 remote chains...] -- 1:18:16      15830000 -- [-7560.263] [...7 remote chains...] -- 1:18:13      15840000 -- (-7568.680) [...7 remote chains...] -- 1:18:09      15850000 -- [-7555.313] [...7 remote chains...] -- 1:18:06       Average standard deviation of split frequencies: 0.010042       15860000 -- [-7562.081] [...7 remote chains...] -- 1:18:03      15870000 -- (-7554.828) [...7 remote chains...] -- 1:17:59      15880000 -- [-7542.662] [...7 remote chains...] -- 1:17:56      15890000 -- [-7557.641] [...7 remote chains...] -- 1:17:53      15900000 -- [-7557.326] [...7 remote chains...] -- 1:17:49       Average standard deviation of split frequencies: 0.009958       15910000 -- [-7548.742] [...7 remote chains...] -- 1:17:46      15920000 -- (-7563.916) [...7 remote chains...] -- 1:17:42      15930000 -- (-7569.114) [...7 remote chains...] -- 1:17:39      15940000 -- (-7551.282) [...7 remote chains...] -- 1:17:36      15950000 -- (-7556.413) [...7 remote chains...] -- 1:17:32       Average standard deviation of split frequencies: 0.009982       15960000 -- (-7556.563) [...7 remote chains...] -- 1:17:29      15970000 -- (-7549.312) [...7 remote chains...] -- 1:17:26      15980000 -- [-7547.122] [...7 remote chains...] -- 1:17:22      15990000 -- [-7572.692] [...7 remote chains...] -- 1:17:19      16000000 -- [-7543.501] [...7 remote chains...] -- 1:17:16       Average standard deviation of split frequencies: 0.009989       16010000 -- [-7571.544] [...7 remote chains...] -- 1:17:13      16020000 -- [-7557.611] [...7 remote chains...] -- 1:17:09      16030000 -- (-7546.494) [...7 remote chains...] -- 1:17:05      16040000 -- [-7560.714] [...7 remote chains...] -- 1:17:03      16050000 -- [-7579.185] [...7 remote chains...] -- 1:16:59       Average standard deviation of split frequencies: 0.009978       16060000 -- [-7552.544] [...7 remote chains...] -- 1:16:55      16070000 -- [-7553.108] [...7 remote chains...] -- 1:16:52      16080000 -- (-7565.289) [...7 remote chains...] -- 1:16:48      16090000 -- [-7539.371] [...7 remote chains...] -- 1:16:46      16100000 -- [-7551.859] [...7 remote chains...] -- 1:16:42       Average standard deviation of split frequencies: 0.009747       16110000 -- [-7555.101] [...7 remote chains...] -- 1:16:38      16120000 -- [-7569.518] [...7 remote chains...] -- 1:16:35      16130000 -- [-7540.820] [...7 remote chains...] -- 1:16:32      16140000 -- [-7560.595] [...7 remote chains...] -- 1:16:29      16150000 -- (-7558.655) [...7 remote chains...] -- 1:16:25       Average standard deviation of split frequencies: 0.009667       16160000 -- [-7575.054] [...7 remote chains...] -- 1:16:21      16170000 -- [-7552.005] [...7 remote chains...] -- 1:16:18      16180000 -- [-7541.215] [...7 remote chains...] -- 1:16:15      16190000 -- (-7548.034) [...7 remote chains...] -- 1:16:12      16200000 -- [-7575.941] [...7 remote chains...] -- 1:16:08       Average standard deviation of split frequencies: 0.009682       16210000 -- [-7567.090] [...7 remote chains...] -- 1:16:04      16220000 -- [-7550.269] [...7 remote chains...] -- 1:16:02      16230000 -- [-7533.351] [...7 remote chains...] -- 1:15:58      16240000 -- [-7563.322] [...7 remote chains...] -- 1:15:55      16250000 -- [-7538.232] [...7 remote chains...] -- 1:15:51       Average standard deviation of split frequencies: 0.009476       16260000 -- [-7549.289] [...7 remote chains...] -- 1:15:48      16270000 -- [-7570.040] [...7 remote chains...] -- 1:15:45      16280000 -- (-7588.005) [...7 remote chains...] -- 1:15:41      16290000 -- (-7578.748) [...7 remote chains...] -- 1:15:38      16300000 -- (-7564.075) [...7 remote chains...] -- 1:15:34       Average standard deviation of split frequencies: 0.009412       16310000 -- (-7581.570) [...7 remote chains...] -- 1:15:31      16320000 -- (-7573.429) [...7 remote chains...] -- 1:15:28      16330000 -- (-7560.336) [...7 remote chains...] -- 1:15:24      16340000 -- (-7580.098) [...7 remote chains...] -- 1:15:21      16350000 -- (-7556.576) [...7 remote chains...] -- 1:15:18       Average standard deviation of split frequencies: 0.009462       16360000 -- (-7562.503) [...7 remote chains...] -- 1:15:14      16370000 -- (-7567.644) [...7 remote chains...] -- 1:15:11      16380000 -- (-7569.613) [...7 remote chains...] -- 1:15:07      16390000 -- (-7553.132) [...7 remote chains...] -- 1:15:04      16400000 -- (-7561.439) [...7 remote chains...] -- 1:15:01       Average standard deviation of split frequencies: 0.009496       16410000 -- (-7565.977) [...7 remote chains...] -- 1:14:57      16420000 -- (-7573.368) [...7 remote chains...] -- 1:14:54      16430000 -- (-7565.275) [...7 remote chains...] -- 1:14:51      16440000 -- (-7559.703) [...7 remote chains...] -- 1:14:47      16450000 -- (-7562.394) [...7 remote chains...] -- 1:14:44       Average standard deviation of split frequencies: 0.009709       16460000 -- (-7563.210) [...7 remote chains...] -- 1:14:40      16470000 -- (-7574.910) [...7 remote chains...] -- 1:14:37      16480000 -- (-7546.066) [...7 remote chains...] -- 1:14:34      16490000 -- (-7573.422) [...7 remote chains...] -- 1:14:30      16500000 -- (-7575.377) [...7 remote chains...] -- 1:14:27       Average standard deviation of split frequencies: 0.009862       16510000 -- (-7570.313) [...7 remote chains...] -- 1:14:24      16520000 -- (-7562.837) [...7 remote chains...] -- 1:14:20      16530000 -- (-7549.602) [...7 remote chains...] -- 1:14:17      16540000 -- (-7549.537) [...7 remote chains...] -- 1:14:14      16550000 -- (-7549.938) [...7 remote chains...] -- 1:14:11       Average standard deviation of split frequencies: 0.009880       16560000 -- (-7550.909) [...7 remote chains...] -- 1:14:07      16570000 -- [-7539.654] [...7 remote chains...] -- 1:14:03      16580000 -- (-7542.705) [...7 remote chains...] -- 1:14:01      16590000 -- (-7548.312) [...7 remote chains...] -- 1:13:57      16600000 -- [-7549.788] [...7 remote chains...] -- 1:13:54       Average standard deviation of split frequencies: 0.010001       16610000 -- [-7546.812] [...7 remote chains...] -- 1:13:50      16620000 -- [-7548.154] [...7 remote chains...] -- 1:13:47      16630000 -- [-7563.008] [...7 remote chains...] -- 1:13:44      16640000 -- [-7577.237] [...7 remote chains...] -- 1:13:40      16650000 -- [-7542.214] [...7 remote chains...] -- 1:13:37       Average standard deviation of split frequencies: 0.009892       16660000 -- [-7553.156] [...7 remote chains...] -- 1:13:34      16670000 -- [-7537.776] [...7 remote chains...] -- 1:13:30      16680000 -- [-7550.735] [...7 remote chains...] -- 1:13:27      16690000 -- [-7560.558] [...7 remote chains...] -- 1:13:23      16700000 -- [-7554.566] [...7 remote chains...] -- 1:13:20       Average standard deviation of split frequencies: 0.009851       16710000 -- [-7547.124] [...7 remote chains...] -- 1:13:17      16720000 -- [-7567.839] [...7 remote chains...] -- 1:13:13      16730000 -- [-7561.541] [...7 remote chains...] -- 1:13:11      16740000 -- [-7548.122] [...7 remote chains...] -- 1:13:07      16750000 -- [-7548.577] [...7 remote chains...] -- 1:13:03       Average standard deviation of split frequencies: 0.010109       16760000 -- [-7553.856] [...7 remote chains...] -- 1:13:00      16770000 -- [-7550.076] [...7 remote chains...] -- 1:12:56      16780000 -- [-7548.812] [...7 remote chains...] -- 1:12:54      16790000 -- [-7537.904] [...7 remote chains...] -- 1:12:50      16800000 -- (-7554.457) [...7 remote chains...] -- 1:12:47       Average standard deviation of split frequencies: 0.010085       16810000 -- (-7549.881) [...7 remote chains...] -- 1:12:43      16820000 -- (-7557.050) [...7 remote chains...] -- 1:12:40      16830000 -- (-7550.005) [...7 remote chains...] -- 1:12:37      16840000 -- (-7560.685) [...7 remote chains...] -- 1:12:33      16850000 -- (-7559.670) [...7 remote chains...] -- 1:12:30       Average standard deviation of split frequencies: 0.009946       16860000 -- (-7576.175) [...7 remote chains...] -- 1:12:27      16870000 -- (-7562.072) [...7 remote chains...] -- 1:12:23      16880000 -- (-7541.723) [...7 remote chains...] -- 1:12:20      16890000 -- (-7577.878) [...7 remote chains...] -- 1:12:16      16900000 -- (-7558.458) [...7 remote chains...] -- 1:12:13       Average standard deviation of split frequencies: 0.009680       16910000 -- (-7569.494) [...7 remote chains...] -- 1:12:10      16920000 -- (-7568.834) [...7 remote chains...] -- 1:12:06      16930000 -- (-7565.554) [...7 remote chains...] -- 1:12:03      16940000 -- (-7563.633) [...7 remote chains...] -- 1:12:00      16950000 -- (-7541.467) [...7 remote chains...] -- 1:11:56       Average standard deviation of split frequencies: 0.009539       16960000 -- (-7557.255) [...7 remote chains...] -- 1:11:53      16970000 -- (-7567.085) [...7 remote chains...] -- 1:11:49      16980000 -- (-7561.840) [...7 remote chains...] -- 1:11:47      16990000 -- (-7557.922) [...7 remote chains...] -- 1:11:43      17000000 -- (-7563.044) [...7 remote chains...] -- 1:11:39       Average standard deviation of split frequencies: 0.009493       17010000 -- [-7555.162] [...7 remote chains...] -- 1:11:36      17020000 -- [-7535.653] [...7 remote chains...] -- 1:11:33      17030000 -- [-7536.929] [...7 remote chains...] -- 1:11:30      17040000 -- [-7534.056] [...7 remote chains...] -- 1:11:26      17050000 -- [-7551.677] [...7 remote chains...] -- 1:11:22       Average standard deviation of split frequencies: 0.009557       17060000 -- [-7540.526] [...7 remote chains...] -- 1:11:20      17070000 -- [-7535.465] [...7 remote chains...] -- 1:11:16      17080000 -- (-7555.164) [...7 remote chains...] -- 1:11:13      17090000 -- (-7569.983) [...7 remote chains...] -- 1:11:09      17100000 -- (-7556.610) [...7 remote chains...] -- 1:11:06       Average standard deviation of split frequencies: 0.009549       17110000 -- (-7550.046) [...7 remote chains...] -- 1:11:03      17120000 -- (-7558.306) [...7 remote chains...] -- 1:10:59      17130000 -- [-7554.142] [...7 remote chains...] -- 1:10:56      17140000 -- [-7551.989] [...7 remote chains...] -- 1:10:53      17150000 -- (-7545.944) [...7 remote chains...] -- 1:10:49       Average standard deviation of split frequencies: 0.009668       17160000 -- (-7547.448) [...7 remote chains...] -- 1:10:46      17170000 -- [-7538.940] [...7 remote chains...] -- 1:10:42      17180000 -- [-7562.196] [...7 remote chains...] -- 1:10:40      17190000 -- [-7541.643] [...7 remote chains...] -- 1:10:36      17200000 -- [-7534.505] [...7 remote chains...] -- 1:10:32       Average standard deviation of split frequencies: 0.009707       17210000 -- (-7557.687) [...7 remote chains...] -- 1:10:30      17220000 -- (-7543.350) [...7 remote chains...] -- 1:10:26      17230000 -- (-7546.274) [...7 remote chains...] -- 1:10:23      17240000 -- (-7575.256) [...7 remote chains...] -- 1:10:19      17250000 -- (-7577.920) [...7 remote chains...] -- 1:10:16       Average standard deviation of split frequencies: 0.009750       17260000 -- (-7558.941) [...7 remote chains...] -- 1:10:13      17270000 -- (-7567.881) [...7 remote chains...] -- 1:10:09      17280000 -- (-7568.948) [...7 remote chains...] -- 1:10:06      17290000 -- (-7553.562) [...7 remote chains...] -- 1:10:03      17300000 -- (-7547.884) [...7 remote chains...] -- 1:09:59       Average standard deviation of split frequencies: 0.009935       17310000 -- (-7565.532) [...7 remote chains...] -- 1:09:56      17320000 -- (-7561.925) [...7 remote chains...] -- 1:09:52      17330000 -- (-7549.232) [...7 remote chains...] -- 1:09:49      17340000 -- (-7531.420) [...7 remote chains...] -- 1:09:46      17350000 -- (-7538.411) [...7 remote chains...] -- 1:09:42       Average standard deviation of split frequencies: 0.009951       17360000 -- (-7555.709) [...7 remote chains...] -- 1:09:40      17370000 -- (-7559.870) [...7 remote chains...] -- 1:09:36      17380000 -- (-7538.548) [...7 remote chains...] -- 1:09:33      17390000 -- (-7555.521) [...7 remote chains...] -- 1:09:29      17400000 -- (-7573.609) [...7 remote chains...] -- 1:09:26       Average standard deviation of split frequencies: 0.009869       17410000 -- (-7556.330) [...7 remote chains...] -- 1:09:23      17420000 -- (-7556.556) [...7 remote chains...] -- 1:09:19      17430000 -- [-7542.606] [...7 remote chains...] -- 1:09:16      17440000 -- [-7540.600] [...7 remote chains...] -- 1:09:13      17450000 -- [-7551.578] [...7 remote chains...] -- 1:09:09       Average standard deviation of split frequencies: 0.009769       17460000 -- [-7551.756] [...7 remote chains...] -- 1:09:06      17470000 -- [-7556.605] [...7 remote chains...] -- 1:09:02      17480000 -- (-7559.369) [...7 remote chains...] -- 1:08:59      17490000 -- (-7546.883) [...7 remote chains...] -- 1:08:56      17500000 -- (-7575.112) [...7 remote chains...] -- 1:08:52       Average standard deviation of split frequencies: 0.009758       17510000 -- (-7561.563) [...7 remote chains...] -- 1:08:49      17520000 -- [-7574.220] [...7 remote chains...] -- 1:08:46      17530000 -- [-7556.001] [...7 remote chains...] -- 1:08:42      17540000 -- [-7532.242] [...7 remote chains...] -- 1:08:39      17550000 -- [-7552.296] [...7 remote chains...] -- 1:08:35       Average standard deviation of split frequencies: 0.009575       17560000 -- (-7571.069) [...7 remote chains...] -- 1:08:33      17570000 -- (-7557.145) [...7 remote chains...] -- 1:08:29      17580000 -- (-7556.408) [...7 remote chains...] -- 1:08:26      17590000 -- (-7554.929) [...7 remote chains...] -- 1:08:22      17600000 -- (-7552.816) [...7 remote chains...] -- 1:08:19       Average standard deviation of split frequencies: 0.009604       17610000 -- (-7545.917) [...7 remote chains...] -- 1:08:16      17620000 -- (-7558.458) [...7 remote chains...] -- 1:08:12      17630000 -- (-7578.175) [...7 remote chains...] -- 1:08:09      17640000 -- (-7580.808) [...7 remote chains...] -- 1:08:06      17650000 -- (-7541.405) [...7 remote chains...] -- 1:08:02       Average standard deviation of split frequencies: 0.009578       17660000 -- (-7559.157) [...7 remote chains...] -- 1:07:59      17670000 -- (-7544.148) [...7 remote chains...] -- 1:07:56      17680000 -- (-7549.853) [...7 remote chains...] -- 1:07:52      17690000 -- (-7558.394) [...7 remote chains...] -- 1:07:49      17700000 -- (-7550.753) [...7 remote chains...] -- 1:07:45       Average standard deviation of split frequencies: 0.009751       17710000 -- (-7550.191) [...7 remote chains...] -- 1:07:43      17720000 -- (-7534.543) [...7 remote chains...] -- 1:07:39      17730000 -- (-7552.756) [...7 remote chains...] -- 1:07:36      17740000 -- (-7540.845) [...7 remote chains...] -- 1:07:33      17750000 -- (-7568.650) [...7 remote chains...] -- 1:07:29       Average standard deviation of split frequencies: 0.009806       17760000 -- (-7564.166) [...7 remote chains...] -- 1:07:26      17770000 -- (-7572.360) [...7 remote chains...] -- 1:07:22      17780000 -- (-7552.951) [...7 remote chains...] -- 1:07:19      17790000 -- (-7567.503) [...7 remote chains...] -- 1:07:16      17800000 -- (-7572.831) [...7 remote chains...] -- 1:07:12       Average standard deviation of split frequencies: 0.009719       17810000 -- (-7569.319) [...7 remote chains...] -- 1:07:10      17820000 -- (-7548.887) [...7 remote chains...] -- 1:07:06      17830000 -- (-7552.727) [...7 remote chains...] -- 1:07:02      17840000 -- (-7565.860) [...7 remote chains...] -- 1:06:59      17850000 -- (-7542.686) [...7 remote chains...] -- 1:06:56       Average standard deviation of split frequencies: 0.009659       17860000 -- (-7537.996) [...7 remote chains...] -- 1:06:53      17870000 -- (-7540.272) [...7 remote chains...] -- 1:06:49      17880000 -- (-7540.600) [...7 remote chains...] -- 1:06:46      17890000 -- (-7551.853) [...7 remote chains...] -- 1:06:43      17900000 -- (-7551.578) [...7 remote chains...] -- 1:06:39       Average standard deviation of split frequencies: 0.009598       17910000 -- (-7549.980) [...7 remote chains...] -- 1:06:36      17920000 -- (-7552.388) [...7 remote chains...] -- 1:06:32      17930000 -- (-7555.086) [...7 remote chains...] -- 1:06:29      17940000 -- (-7578.880) [...7 remote chains...] -- 1:06:26      17950000 -- (-7570.906) [...7 remote chains...] -- 1:06:22       Average standard deviation of split frequencies: 0.009571       17960000 -- (-7564.990) [...7 remote chains...] -- 1:06:19      17970000 -- (-7579.099) [...7 remote chains...] -- 1:06:16      17980000 -- (-7552.860) [...7 remote chains...] -- 1:06:13      17990000 -- (-7585.459) [...7 remote chains...] -- 1:06:09      18000000 -- (-7564.367) [...7 remote chains...] -- 1:06:06       Average standard deviation of split frequencies: 0.009540       18010000 -- [-7552.596] [...7 remote chains...] -- 1:06:03      18020000 -- [-7567.517] [...7 remote chains...] -- 1:05:59      18030000 -- (-7573.115) [...7 remote chains...] -- 1:05:56      18040000 -- (-7563.754) [...7 remote chains...] -- 1:05:53      18050000 -- (-7547.017) [...7 remote chains...] -- 1:05:49       Average standard deviation of split frequencies: 0.009542       18060000 -- [-7567.253] [...7 remote chains...] -- 1:05:46      18070000 -- (-7556.092) [...7 remote chains...] -- 1:05:43      18080000 -- (-7572.086) [...7 remote chains...] -- 1:05:39      18090000 -- [-7564.542] [...7 remote chains...] -- 1:05:36      18100000 -- (-7573.100) [...7 remote chains...] -- 1:05:32       Average standard deviation of split frequencies: 0.009761       18110000 -- (-7547.433) [...7 remote chains...] -- 1:05:30      18120000 -- [-7543.715] [...7 remote chains...] -- 1:05:26      18130000 -- [-7551.703] [...7 remote chains...] -- 1:05:23      18140000 -- [-7553.426] [...7 remote chains...] -- 1:05:20      18150000 -- (-7548.212) [...7 remote chains...] -- 1:05:16       Average standard deviation of split frequencies: 0.009925       18160000 -- (-7558.580) [...7 remote chains...] -- 1:05:13      18170000 -- (-7561.072) [...7 remote chains...] -- 1:05:09      18180000 -- (-7553.393) [...7 remote chains...] -- 1:05:06      18190000 -- (-7562.739) [...7 remote chains...] -- 1:05:03      18200000 -- (-7545.158) [...7 remote chains...] -- 1:04:59       Average standard deviation of split frequencies: 0.010036       18210000 -- (-7567.870) [...7 remote chains...] -- 1:04:56      18220000 -- (-7571.128) [...7 remote chains...] -- 1:04:53      18230000 -- (-7585.324) [...7 remote chains...] -- 1:04:49      18240000 -- (-7585.298) [...7 remote chains...] -- 1:04:46      18250000 -- (-7566.363) [...7 remote chains...] -- 1:04:42       Average standard deviation of split frequencies: 0.009921       18260000 -- (-7577.123) [...7 remote chains...] -- 1:04:40      18270000 -- (-7552.678) [...7 remote chains...] -- 1:04:36      18280000 -- (-7550.842) [...7 remote chains...] -- 1:04:33      18290000 -- (-7557.749) [...7 remote chains...] -- 1:04:30      18300000 -- (-7568.357) [...7 remote chains...] -- 1:04:26       Average standard deviation of split frequencies: 0.009844       18310000 -- (-7539.793) [...7 remote chains...] -- 1:04:23      18320000 -- (-7569.319) [...7 remote chains...] -- 1:04:19      18330000 -- (-7571.067) [...7 remote chains...] -- 1:04:16      18340000 -- (-7577.826) [...7 remote chains...] -- 1:04:13      18350000 -- [-7555.244] [...7 remote chains...] -- 1:04:09       Average standard deviation of split frequencies: 0.009902       18360000 -- [-7554.194] [...7 remote chains...] -- 1:04:07      18370000 -- [-7560.314] [...7 remote chains...] -- 1:04:03      18380000 -- [-7544.354] [...7 remote chains...] -- 1:04:00      18390000 -- [-7548.860] [...7 remote chains...] -- 1:03:56      18400000 -- [-7565.492] [...7 remote chains...] -- 1:03:53       Average standard deviation of split frequencies: 0.009829       18410000 -- [-7540.061] [...7 remote chains...] -- 1:03:50      18420000 -- [-7548.687] [...7 remote chains...] -- 1:03:46      18430000 -- (-7539.800) [...7 remote chains...] -- 1:03:43      18440000 -- [-7567.238] [...7 remote chains...] -- 1:03:40      18450000 -- (-7546.464) [...7 remote chains...] -- 1:03:36       Average standard deviation of split frequencies: 0.009805       18460000 -- [-7549.839] [...7 remote chains...] -- 1:03:33      18470000 -- [-7558.175] [...7 remote chains...] -- 1:03:29      18480000 -- [-7541.597] [...7 remote chains...] -- 1:03:26      18490000 -- [-7548.902] [...7 remote chains...] -- 1:03:23      18500000 -- [-7541.472] [...7 remote chains...] -- 1:03:19       Average standard deviation of split frequencies: 0.009741       18510000 -- [-7561.977] [...7 remote chains...] -- 1:03:16      18520000 -- [-7564.847] [...7 remote chains...] -- 1:03:13      18530000 -- [-7547.881] [...7 remote chains...] -- 1:03:10      18540000 -- [-7547.755] [...7 remote chains...] -- 1:03:06      18550000 -- [-7566.306] [...7 remote chains...] -- 1:03:03       Average standard deviation of split frequencies: 0.009826       18560000 -- (-7556.341) [...7 remote chains...] -- 1:03:00      18570000 -- (-7550.406) [...7 remote chains...] -- 1:02:56      18580000 -- (-7556.441) [...7 remote chains...] -- 1:02:53      18590000 -- (-7552.861) [...7 remote chains...] -- 1:02:49      18600000 -- [-7566.409] [...7 remote chains...] -- 1:02:46       Average standard deviation of split frequencies: 0.009742       18610000 -- [-7564.533] [...7 remote chains...] -- 1:02:43      18620000 -- [-7556.679] [...7 remote chains...] -- 1:02:39      18630000 -- (-7578.905) [...7 remote chains...] -- 1:02:36      18640000 -- (-7584.702) [...7 remote chains...] -- 1:02:33      18650000 -- (-7558.273) [...7 remote chains...] -- 1:02:30       Average standard deviation of split frequencies: 0.009598       18660000 -- (-7558.934) [...7 remote chains...] -- 1:02:26      18670000 -- (-7562.738) [...7 remote chains...] -- 1:02:23      18680000 -- (-7585.954) [...7 remote chains...] -- 1:02:20      18690000 -- (-7569.450) [...7 remote chains...] -- 1:02:16      18700000 -- (-7539.568) [...7 remote chains...] -- 1:02:13       Average standard deviation of split frequencies: 0.009703       18710000 -- (-7589.730) [...7 remote chains...] -- 1:02:09      18720000 -- (-7583.250) [...7 remote chains...] -- 1:02:06      18730000 -- (-7557.768) [...7 remote chains...] -- 1:02:03      18740000 -- (-7554.695) [...7 remote chains...] -- 1:01:59      18750000 -- (-7532.862) [...7 remote chains...] -- 1:01:56       Average standard deviation of split frequencies: 0.009772       18760000 -- (-7545.634) [...7 remote chains...] -- 1:01:53      18770000 -- (-7579.185) [...7 remote chains...] -- 1:01:50      18780000 -- (-7553.292) [...7 remote chains...] -- 1:01:46      18790000 -- (-7559.746) [...7 remote chains...] -- 1:01:43      18800000 -- (-7557.986) [...7 remote chains...] -- 1:01:40       Average standard deviation of split frequencies: 0.009576       18810000 -- (-7560.632) [...7 remote chains...] -- 1:01:36      18820000 -- (-7544.252) [...7 remote chains...] -- 1:01:33      18830000 -- (-7551.825) [...7 remote chains...] -- 1:01:30      18840000 -- [-7539.058] [...7 remote chains...] -- 1:01:26      18850000 -- (-7555.937) [...7 remote chains...] -- 1:01:23       Average standard deviation of split frequencies: 0.009438       18860000 -- (-7553.556) [...7 remote chains...] -- 1:01:19      18870000 -- (-7552.730) [...7 remote chains...] -- 1:01:16      18880000 -- [-7565.098] [...7 remote chains...] -- 1:01:13      18890000 -- (-7560.500) [...7 remote chains...] -- 1:01:10      18900000 -- (-7566.227) [...7 remote chains...] -- 1:01:06       Average standard deviation of split frequencies: 0.009538       18910000 -- (-7569.573) [...7 remote chains...] -- 1:01:03      18920000 -- (-7556.936) [...7 remote chains...] -- 1:01:00      18930000 -- (-7572.178) [...7 remote chains...] -- 1:00:56      18940000 -- (-7555.927) [...7 remote chains...] -- 1:00:53      18950000 -- (-7556.235) [...7 remote chains...] -- 1:00:50       Average standard deviation of split frequencies: 0.009396       18960000 -- (-7582.396) [...7 remote chains...] -- 1:00:46      18970000 -- (-7575.746) [...7 remote chains...] -- 1:00:43      18980000 -- (-7569.635) [...7 remote chains...] -- 1:00:39      18990000 -- (-7551.444) [...7 remote chains...] -- 1:00:36      19000000 -- (-7562.507) [...7 remote chains...] -- 1:00:33       Average standard deviation of split frequencies: 0.009307       19010000 -- (-7563.074) [...7 remote chains...] -- 1:00:29      19020000 -- [-7558.638] [...7 remote chains...] -- 1:00:26      19030000 -- (-7551.041) [...7 remote chains...] -- 1:00:23      19040000 -- (-7570.611) [...7 remote chains...] -- 1:00:20      19050000 -- (-7556.278) [...7 remote chains...] -- 1:00:16       Average standard deviation of split frequencies: 0.009458       19060000 -- (-7564.842) [...7 remote chains...] -- 1:00:13      19070000 -- (-7574.077) [...7 remote chains...] -- 1:00:10      19080000 -- (-7559.442) [...7 remote chains...] -- 1:00:06      19090000 -- (-7553.847) [...7 remote chains...] -- 1:00:03      19100000 -- (-7555.243) [...7 remote chains...] -- 0:59:59       Average standard deviation of split frequencies: 0.009409       19110000 -- (-7541.927) [...7 remote chains...] -- 0:59:56      19120000 -- (-7558.932) [...7 remote chains...] -- 0:59:53      19130000 -- (-7563.851) [...7 remote chains...] -- 0:59:49      19140000 -- (-7565.468) [...7 remote chains...] -- 0:59:46      19150000 -- (-7547.685) [...7 remote chains...] -- 0:59:43       Average standard deviation of split frequencies: 0.009379       19160000 -- (-7565.491) [...7 remote chains...] -- 0:59:40      19170000 -- (-7560.658) [...7 remote chains...] -- 0:59:36      19180000 -- (-7546.491) [...7 remote chains...] -- 0:59:33      19190000 -- (-7561.707) [...7 remote chains...] -- 0:59:30      19200000 -- (-7545.655) [...7 remote chains...] -- 0:59:26       Average standard deviation of split frequencies: 0.009378       19210000 -- (-7558.110) [...7 remote chains...] -- 0:59:23      19220000 -- (-7559.052) [...7 remote chains...] -- 0:59:19      19230000 -- (-7553.347) [...7 remote chains...] -- 0:59:16      19240000 -- (-7565.369) [...7 remote chains...] -- 0:59:13      19250000 -- (-7574.134) [...7 remote chains...] -- 0:59:10       Average standard deviation of split frequencies: 0.009562       19260000 -- [-7558.725] [...7 remote chains...] -- 0:59:06      19270000 -- (-7563.283) [...7 remote chains...] -- 0:59:03      19280000 -- (-7577.444) [...7 remote chains...] -- 0:59:00      19290000 -- (-7561.211) [...7 remote chains...] -- 0:58:56      19300000 -- (-7567.890) [...7 remote chains...] -- 0:58:53       Average standard deviation of split frequencies: 0.009570       19310000 -- [-7558.014] [...7 remote chains...] -- 0:58:49      19320000 -- [-7544.522] [...7 remote chains...] -- 0:58:46      19330000 -- [-7547.494] [...7 remote chains...] -- 0:58:43      19340000 -- [-7545.881] [...7 remote chains...] -- 0:58:39      19350000 -- [-7564.818] [...7 remote chains...] -- 0:58:36       Average standard deviation of split frequencies: 0.009556       19360000 -- [-7564.213] [...7 remote chains...] -- 0:58:33      19370000 -- (-7553.592) [...7 remote chains...] -- 0:58:30      19380000 -- (-7563.237) [...7 remote chains...] -- 0:58:26      19390000 -- (-7556.070) [...7 remote chains...] -- 0:58:23      19400000 -- (-7562.843) [...7 remote chains...] -- 0:58:20       Average standard deviation of split frequencies: 0.009418       19410000 -- (-7552.542) [...7 remote chains...] -- 0:58:16      19420000 -- (-7564.808) [...7 remote chains...] -- 0:58:13      19430000 -- (-7575.425) [...7 remote chains...] -- 0:58:09      19440000 -- (-7558.959) [...7 remote chains...] -- 0:58:06      19450000 -- (-7548.489) [...7 remote chains...] -- 0:58:03       Average standard deviation of split frequencies: 0.009465       19460000 -- (-7573.355) [...7 remote chains...] -- 0:57:59      19470000 -- (-7547.914) [...7 remote chains...] -- 0:57:56      19480000 -- (-7569.853) [...7 remote chains...] -- 0:57:53      19490000 -- (-7573.078) [...7 remote chains...] -- 0:57:50      19500000 -- (-7556.267) [...7 remote chains...] -- 0:57:46       Average standard deviation of split frequencies: 0.009690       19510000 -- (-7584.295) [...7 remote chains...] -- 0:57:43      19520000 -- (-7545.138) [...7 remote chains...] -- 0:57:40      19530000 -- (-7550.311) [...7 remote chains...] -- 0:57:36      19540000 -- (-7572.670) [...7 remote chains...] -- 0:57:33      19550000 -- (-7558.375) [...7 remote chains...] -- 0:57:29       Average standard deviation of split frequencies: 0.009581       19560000 -- (-7560.776) [...7 remote chains...] -- 0:57:26      19570000 -- [-7550.066] [...7 remote chains...] -- 0:57:23      19580000 -- (-7570.225) [...7 remote chains...] -- 0:57:19      19590000 -- (-7598.349) [...7 remote chains...] -- 0:57:16      19600000 -- (-7567.473) [...7 remote chains...] -- 0:57:13       Average standard deviation of split frequencies: 0.009459       19610000 -- (-7563.507) [...7 remote chains...] -- 0:57:10      19620000 -- (-7580.503) [...7 remote chains...] -- 0:57:06      19630000 -- (-7556.678) [...7 remote chains...] -- 0:57:03      19640000 -- (-7577.434) [...7 remote chains...] -- 0:56:59      19650000 -- (-7571.747) [...7 remote chains...] -- 0:56:56       Average standard deviation of split frequencies: 0.009314       19660000 -- (-7565.141) [...7 remote chains...] -- 0:56:53      19670000 -- (-7575.442) [...7 remote chains...] -- 0:56:49      19680000 -- (-7542.745) [...7 remote chains...] -- 0:56:46      19690000 -- (-7551.173) [...7 remote chains...] -- 0:56:43      19700000 -- (-7554.085) [...7 remote chains...] -- 0:56:40       Average standard deviation of split frequencies: 0.009371       19710000 -- [-7571.209] [...7 remote chains...] -- 0:56:36      19720000 -- (-7558.039) [...7 remote chains...] -- 0:56:33      19730000 -- (-7558.324) [...7 remote chains...] -- 0:56:30      19740000 -- [-7566.809] [...7 remote chains...] -- 0:56:26      19750000 -- [-7544.647] [...7 remote chains...] -- 0:56:23       Average standard deviation of split frequencies: 0.009395       19760000 -- [-7539.189] [...7 remote chains...] -- 0:56:19      19770000 -- (-7554.631) [...7 remote chains...] -- 0:56:16      19780000 -- (-7560.915) [...7 remote chains...] -- 0:56:13      19790000 -- (-7559.972) [...7 remote chains...] -- 0:56:09      19800000 -- (-7562.457) [...7 remote chains...] -- 0:56:06       Average standard deviation of split frequencies: 0.009358       19810000 -- (-7556.522) [...7 remote chains...] -- 0:56:03      19820000 -- (-7562.137) [...7 remote chains...] -- 0:56:00      19830000 -- (-7571.611) [...7 remote chains...] -- 0:55:56      19840000 -- (-7582.384) [...7 remote chains...] -- 0:55:53      19850000 -- [-7551.970] [...7 remote chains...] -- 0:55:49       Average standard deviation of split frequencies: 0.009340       19860000 -- (-7565.250) [...7 remote chains...] -- 0:55:46      19870000 -- (-7591.810) [...7 remote chains...] -- 0:55:43      19880000 -- (-7561.172) [...7 remote chains...] -- 0:55:39      19890000 -- [-7546.103] [...7 remote chains...] -- 0:55:36      19900000 -- (-7559.858) [...7 remote chains...] -- 0:55:33       Average standard deviation of split frequencies: 0.009358       19910000 -- [-7545.009] [...7 remote chains...] -- 0:55:30      19920000 -- (-7546.968) [...7 remote chains...] -- 0:55:26      19930000 -- (-7559.247) [...7 remote chains...] -- 0:55:23      19940000 -- (-7567.825) [...7 remote chains...] -- 0:55:20      19950000 -- [-7559.097] [...7 remote chains...] -- 0:55:16       Average standard deviation of split frequencies: 0.009369       19960000 -- [-7546.508] [...7 remote chains...] -- 0:55:13      19970000 -- [-7555.265] [...7 remote chains...] -- 0:55:09      19980000 -- [-7560.779] [...7 remote chains...] -- 0:55:06      19990000 -- [-7556.385] [...7 remote chains...] -- 0:55:03      20000000 -- [-7562.290] [...7 remote chains...] -- 0:55:00       Average standard deviation of split frequencies: 0.009327       20010000 -- [-7561.540] [...7 remote chains...] -- 0:54:57      20020000 -- [-7548.341] [...7 remote chains...] -- 0:54:53      20030000 -- (-7558.442) [...7 remote chains...] -- 0:54:50      20040000 -- [-7541.569] [...7 remote chains...] -- 0:54:46      20050000 -- [-7544.965] [...7 remote chains...] -- 0:54:43       Average standard deviation of split frequencies: 0.009368       20060000 -- [-7537.281] [...7 remote chains...] -- 0:54:40      20070000 -- [-7549.777] [...7 remote chains...] -- 0:54:36      20080000 -- (-7557.204) [...7 remote chains...] -- 0:54:33      20090000 -- [-7558.096] [...7 remote chains...] -- 0:54:30      20100000 -- [-7559.354] [...7 remote chains...] -- 0:54:27       Average standard deviation of split frequencies: 0.009295       20110000 -- [-7546.151] [...7 remote chains...] -- 0:54:23      20120000 -- [-7563.586] [...7 remote chains...] -- 0:54:20      20130000 -- [-7557.037] [...7 remote chains...] -- 0:54:17      20140000 -- [-7559.825] [...7 remote chains...] -- 0:54:13      20150000 -- [-7545.566] [...7 remote chains...] -- 0:54:10       Average standard deviation of split frequencies: 0.009131       20160000 -- [-7563.975] [...7 remote chains...] -- 0:54:06      20170000 -- [-7544.996] [...7 remote chains...] -- 0:54:03      20180000 -- (-7550.276) [...7 remote chains...] -- 0:54:00      20190000 -- [-7566.546] [...7 remote chains...] -- 0:53:56      20200000 -- (-7558.267) [...7 remote chains...] -- 0:53:53       Average standard deviation of split frequencies: 0.009088       20210000 -- (-7547.001) [...7 remote chains...] -- 0:53:50      20220000 -- (-7557.746) [...7 remote chains...] -- 0:53:47      20230000 -- (-7558.595) [...7 remote chains...] -- 0:53:43      20240000 -- (-7576.400) [...7 remote chains...] -- 0:53:40      20250000 -- (-7557.588) [...7 remote chains...] -- 0:53:37       Average standard deviation of split frequencies: 0.009176       20260000 -- (-7560.163) [...7 remote chains...] -- 0:53:33      20270000 -- (-7592.289) [...7 remote chains...] -- 0:53:30      20280000 -- (-7577.033) [...7 remote chains...] -- 0:53:27      20290000 -- (-7579.322) [...7 remote chains...] -- 0:53:23      20300000 -- (-7588.032) [...7 remote chains...] -- 0:53:20       Average standard deviation of split frequencies: 0.009245       20310000 -- (-7577.749) [...7 remote chains...] -- 0:53:17      20320000 -- (-7549.737) [...7 remote chains...] -- 0:53:14      20330000 -- (-7547.696) [...7 remote chains...] -- 0:53:10      20340000 -- (-7567.787) [...7 remote chains...] -- 0:53:07      20350000 -- (-7554.206) [...7 remote chains...] -- 0:53:03       Average standard deviation of split frequencies: 0.009218       20360000 -- (-7572.240) [...7 remote chains...] -- 0:53:00      20370000 -- (-7579.271) [...7 remote chains...] -- 0:52:57      20380000 -- (-7538.877) [...7 remote chains...] -- 0:52:53      20390000 -- (-7561.683) [...7 remote chains...] -- 0:52:50      20400000 -- (-7568.018) [...7 remote chains...] -- 0:52:47       Average standard deviation of split frequencies: 0.009235       20410000 -- (-7539.026) [...7 remote chains...] -- 0:52:44      20420000 -- (-7574.469) [...7 remote chains...] -- 0:52:40      20430000 -- (-7538.393) [...7 remote chains...] -- 0:52:37      20440000 -- (-7544.602) [...7 remote chains...] -- 0:52:33      20450000 -- (-7555.116) [...7 remote chains...] -- 0:52:30       Average standard deviation of split frequencies: 0.009280       20460000 -- (-7539.580) [...7 remote chains...] -- 0:52:27      20470000 -- (-7557.202) [...7 remote chains...] -- 0:52:23      20480000 -- (-7550.615) [...7 remote chains...] -- 0:52:20      20490000 -- (-7568.990) [...7 remote chains...] -- 0:52:17      20500000 -- (-7553.938) [...7 remote chains...] -- 0:52:14       Average standard deviation of split frequencies: 0.009357       20510000 -- (-7552.949) [...7 remote chains...] -- 0:52:10      20520000 -- (-7555.634) [...7 remote chains...] -- 0:52:07      20530000 -- (-7549.013) [...7 remote chains...] -- 0:52:04      20540000 -- [-7565.615] [...7 remote chains...] -- 0:52:00      20550000 -- (-7577.174) [...7 remote chains...] -- 0:51:57       Average standard deviation of split frequencies: 0.009311       20560000 -- (-7561.138) [...7 remote chains...] -- 0:51:53      20570000 -- (-7563.640) [...7 remote chains...] -- 0:51:50      20580000 -- [-7555.729] [...7 remote chains...] -- 0:51:47      20590000 -- (-7553.979) [...7 remote chains...] -- 0:51:44      20600000 -- (-7551.884) [...7 remote chains...] -- 0:51:40       Average standard deviation of split frequencies: 0.009207       20610000 -- (-7545.903) [...7 remote chains...] -- 0:51:37      20620000 -- [-7547.098] [...7 remote chains...] -- 0:51:34      20630000 -- (-7548.579) [...7 remote chains...] -- 0:51:30      20640000 -- [-7553.351] [...7 remote chains...] -- 0:51:27      20650000 -- [-7556.369] [...7 remote chains...] -- 0:51:24       Average standard deviation of split frequencies: 0.009228       20660000 -- (-7570.373) [...7 remote chains...] -- 0:51:20      20670000 -- [-7561.887] [...7 remote chains...] -- 0:51:17      20680000 -- (-7552.108) [...7 remote chains...] -- 0:51:14      20690000 -- (-7568.085) [...7 remote chains...] -- 0:51:11      20700000 -- [-7558.551] [...7 remote chains...] -- 0:51:07       Average standard deviation of split frequencies: 0.009328       20710000 -- [-7539.967] [...7 remote chains...] -- 0:51:04      20720000 -- [-7546.571] [...7 remote chains...] -- 0:51:01      20730000 -- [-7558.306] [...7 remote chains...] -- 0:50:57      20740000 -- [-7549.879] [...7 remote chains...] -- 0:50:54      20750000 -- [-7567.593] [...7 remote chains...] -- 0:50:50       Average standard deviation of split frequencies: 0.009498       20760000 -- [-7556.437] [...7 remote chains...] -- 0:50:47      20770000 -- [-7554.918] [...7 remote chains...] -- 0:50:44      20780000 -- [-7563.931] [...7 remote chains...] -- 0:50:41      20790000 -- (-7585.351) [...7 remote chains...] -- 0:50:37      20800000 -- [-7564.400] [...7 remote chains...] -- 0:50:34       Average standard deviation of split frequencies: 0.009620       20810000 -- [-7546.098] [...7 remote chains...] -- 0:50:31      20820000 -- [-7549.847] [...7 remote chains...] -- 0:50:27      20830000 -- (-7557.586) [...7 remote chains...] -- 0:50:24      20840000 -- [-7559.423] [...7 remote chains...] -- 0:50:21      20850000 -- (-7567.518) [...7 remote chains...] -- 0:50:17       Average standard deviation of split frequencies: 0.009591       20860000 -- (-7540.291) [...7 remote chains...] -- 0:50:14      20870000 -- (-7559.708) [...7 remote chains...] -- 0:50:11      20880000 -- (-7551.078) [...7 remote chains...] -- 0:50:08      20890000 -- (-7547.393) [...7 remote chains...] -- 0:50:04      20900000 -- (-7551.334) [...7 remote chains...] -- 0:50:01       Average standard deviation of split frequencies: 0.009477       20910000 -- (-7555.836) [...7 remote chains...] -- 0:49:58      20920000 -- (-7556.688) [...7 remote chains...] -- 0:49:54      20930000 -- (-7554.062) [...7 remote chains...] -- 0:49:51      20940000 -- (-7545.419) [...7 remote chains...] -- 0:49:47      20950000 -- (-7533.703) [...7 remote chains...] -- 0:49:44       Average standard deviation of split frequencies: 0.009488       20960000 -- (-7537.688) [...7 remote chains...] -- 0:49:41      20970000 -- (-7565.481) [...7 remote chains...] -- 0:49:38      20980000 -- (-7567.166) [...7 remote chains...] -- 0:49:34      20990000 -- (-7555.758) [...7 remote chains...] -- 0:49:31      21000000 -- (-7560.253) [...7 remote chains...] -- 0:49:28       Average standard deviation of split frequencies: 0.009538       21010000 -- (-7554.991) [...7 remote chains...] -- 0:49:24      21020000 -- (-7564.451) [...7 remote chains...] -- 0:49:21      21030000 -- (-7556.145) [...7 remote chains...] -- 0:49:18      21040000 -- (-7567.819) [...7 remote chains...] -- 0:49:15      21050000 -- (-7586.697) [...7 remote chains...] -- 0:49:11       Average standard deviation of split frequencies: 0.009726       21060000 -- (-7592.417) [...7 remote chains...] -- 0:49:08      21070000 -- [-7589.446] [...7 remote chains...] -- 0:49:05      21080000 -- (-7548.461) [...7 remote chains...] -- 0:49:01      21090000 -- [-7543.293] [...7 remote chains...] -- 0:48:58      21100000 -- [-7545.138] [...7 remote chains...] -- 0:48:55       Average standard deviation of split frequencies: 0.009839       21110000 -- (-7537.742) [...7 remote chains...] -- 0:48:51      21120000 -- [-7541.781] [...7 remote chains...] -- 0:48:48      21130000 -- [-7568.733] [...7 remote chains...] -- 0:48:45      21140000 -- (-7568.054) [...7 remote chains...] -- 0:48:42      21150000 -- [-7557.084] [...7 remote chains...] -- 0:48:38       Average standard deviation of split frequencies: 0.009820       21160000 -- [-7554.527] [...7 remote chains...] -- 0:48:35      21170000 -- (-7558.156) [...7 remote chains...] -- 0:48:31      21180000 -- (-7565.091) [...7 remote chains...] -- 0:48:28      21190000 -- (-7570.233) [...7 remote chains...] -- 0:48:25      21200000 -- (-7556.603) [...7 remote chains...] -- 0:48:21       Average standard deviation of split frequencies: 0.009707       21210000 -- (-7543.564) [...7 remote chains...] -- 0:48:18      21220000 -- (-7561.843) [...7 remote chains...] -- 0:48:15      21230000 -- [-7558.797] [...7 remote chains...] -- 0:48:12      21240000 -- (-7561.253) [...7 remote chains...] -- 0:48:08      21250000 -- (-7551.011) [...7 remote chains...] -- 0:48:05       Average standard deviation of split frequencies: 0.009675       21260000 -- (-7571.822) [...7 remote chains...] -- 0:48:01      21270000 -- (-7566.310) [...7 remote chains...] -- 0:47:58      21280000 -- (-7541.269) [...7 remote chains...] -- 0:47:55      21290000 -- (-7561.454) [...7 remote chains...] -- 0:47:51      21300000 -- (-7561.687) [...7 remote chains...] -- 0:47:48       Average standard deviation of split frequencies: 0.009598       21310000 -- (-7567.229) [...7 remote chains...] -- 0:47:45      21320000 -- [-7548.395] [...7 remote chains...] -- 0:47:42      21330000 -- [-7539.200] [...7 remote chains...] -- 0:47:39      21340000 -- [-7548.677] [...7 remote chains...] -- 0:47:35      21350000 -- [-7533.055] [...7 remote chains...] -- 0:47:32       Average standard deviation of split frequencies: 0.009582       21360000 -- [-7545.835] [...7 remote chains...] -- 0:47:28      21370000 -- [-7536.368] [...7 remote chains...] -- 0:47:25      21380000 -- (-7556.529) [...7 remote chains...] -- 0:47:22      21390000 -- (-7538.980) [...7 remote chains...] -- 0:47:19      21400000 -- (-7549.933) [...7 remote chains...] -- 0:47:15       Average standard deviation of split frequencies: 0.009471       21410000 -- (-7587.101) [...7 remote chains...] -- 0:47:12      21420000 -- (-7560.875) [...7 remote chains...] -- 0:47:09      21430000 -- (-7574.561) [...7 remote chains...] -- 0:47:05      21440000 -- (-7565.875) [...7 remote chains...] -- 0:47:02      21450000 -- (-7536.074) [...7 remote chains...] -- 0:46:59       Average standard deviation of split frequencies: 0.009241       21460000 -- (-7551.825) [...7 remote chains...] -- 0:46:55      21470000 -- (-7555.573) [...7 remote chains...] -- 0:46:52      21480000 -- (-7551.394) [...7 remote chains...] -- 0:46:49      21490000 -- (-7554.289) [...7 remote chains...] -- 0:46:46      21500000 -- (-7565.970) [...7 remote chains...] -- 0:46:42       Average standard deviation of split frequencies: 0.009096       21510000 -- (-7551.209) [...7 remote chains...] -- 0:46:39      21520000 -- (-7569.345) [...7 remote chains...] -- 0:46:35      21530000 -- (-7542.670) [...7 remote chains...] -- 0:46:32      21540000 -- [-7561.165] [...7 remote chains...] -- 0:46:29      21550000 -- (-7555.355) [...7 remote chains...] -- 0:46:25       Average standard deviation of split frequencies: 0.009021       21560000 -- (-7546.354) [...7 remote chains...] -- 0:46:22      21570000 -- (-7534.082) [...7 remote chains...] -- 0:46:19      21580000 -- [-7551.161] [...7 remote chains...] -- 0:46:16      21590000 -- [-7542.345] [...7 remote chains...] -- 0:46:12      21600000 -- [-7549.727] [...7 remote chains...] -- 0:46:09       Average standard deviation of split frequencies: 0.009025       21610000 -- [-7553.032] [...7 remote chains...] -- 0:46:05      21620000 -- (-7565.119) [...7 remote chains...] -- 0:46:02      21630000 -- (-7554.743) [...7 remote chains...] -- 0:45:59      21640000 -- (-7570.288) [...7 remote chains...] -- 0:45:56      21650000 -- (-7570.949) [...7 remote chains...] -- 0:45:52       Average standard deviation of split frequencies: 0.009063       21660000 -- [-7546.512] [...7 remote chains...] -- 0:45:49      21670000 -- (-7554.440) [...7 remote chains...] -- 0:45:46      21680000 -- (-7533.407) [...7 remote chains...] -- 0:45:42      21690000 -- (-7550.912) [...7 remote chains...] -- 0:45:39      21700000 -- (-7572.633) [...7 remote chains...] -- 0:45:36       Average standard deviation of split frequencies: 0.009003       21710000 -- (-7547.340) [...7 remote chains...] -- 0:45:32      21720000 -- (-7549.314) [...7 remote chains...] -- 0:45:29      21730000 -- (-7556.924) [...7 remote chains...] -- 0:45:26      21740000 -- (-7569.814) [...7 remote chains...] -- 0:45:23      21750000 -- (-7554.650) [...7 remote chains...] -- 0:45:19       Average standard deviation of split frequencies: 0.008861       21760000 -- [-7518.533] [...7 remote chains...] -- 0:45:16      21770000 -- [-7524.528] [...7 remote chains...] -- 0:45:13      21780000 -- (-7545.324) [...7 remote chains...] -- 0:45:09      21790000 -- [-7535.455] [...7 remote chains...] -- 0:45:06      21800000 -- [-7540.367] [...7 remote chains...] -- 0:45:03       Average standard deviation of split frequencies: 0.008833       21810000 -- [-7531.801] [...7 remote chains...] -- 0:44:59      21820000 -- [-7535.753] [...7 remote chains...] -- 0:44:56      21830000 -- [-7556.445] [...7 remote chains...] -- 0:44:53      21840000 -- [-7554.268] [...7 remote chains...] -- 0:44:50      21850000 -- [-7539.314] [...7 remote chains...] -- 0:44:46       Average standard deviation of split frequencies: 0.008760       21860000 -- (-7556.301) [...7 remote chains...] -- 0:44:43      21870000 -- (-7539.446) [...7 remote chains...] -- 0:44:39      21880000 -- (-7550.848) [...7 remote chains...] -- 0:44:36      21890000 -- (-7546.827) [...7 remote chains...] -- 0:44:33      21900000 -- [-7549.521] [...7 remote chains...] -- 0:44:30       Average standard deviation of split frequencies: 0.008691       21910000 -- (-7573.631) [...7 remote chains...] -- 0:44:26      21920000 -- (-7565.982) [...7 remote chains...] -- 0:44:23      21930000 -- (-7557.396) [...7 remote chains...] -- 0:44:20      21940000 -- (-7578.338) [...7 remote chains...] -- 0:44:16      21950000 -- (-7565.193) [...7 remote chains...] -- 0:44:13       Average standard deviation of split frequencies: 0.008620       21960000 -- [-7553.595] [...7 remote chains...] -- 0:44:10      21970000 -- (-7568.868) [...7 remote chains...] -- 0:44:06      21980000 -- [-7550.896] [...7 remote chains...] -- 0:44:03      21990000 -- [-7559.442] [...7 remote chains...] -- 0:44:00      22000000 -- [-7554.038] [...7 remote chains...] -- 0:43:57       Average standard deviation of split frequencies: 0.008673       22010000 -- [-7564.862] [...7 remote chains...] -- 0:43:53      22020000 -- [-7550.615] [...7 remote chains...] -- 0:43:50      22030000 -- [-7554.007] [...7 remote chains...] -- 0:43:47      22040000 -- [-7542.980] [...7 remote chains...] -- 0:43:43      22050000 -- [-7545.761] [...7 remote chains...] -- 0:43:40       Average standard deviation of split frequencies: 0.008767       22060000 -- (-7554.369) [...7 remote chains...] -- 0:43:37      22070000 -- (-7562.178) [...7 remote chains...] -- 0:43:33      22080000 -- [-7559.685] [...7 remote chains...] -- 0:43:30      22090000 -- (-7539.219) [...7 remote chains...] -- 0:43:27      22100000 -- (-7556.545) [...7 remote chains...] -- 0:43:23       Average standard deviation of split frequencies: 0.008770       22110000 -- [-7532.349] [...7 remote chains...] -- 0:43:20      22120000 -- [-7529.243] [...7 remote chains...] -- 0:43:17      22130000 -- (-7560.534) [...7 remote chains...] -- 0:43:13      22140000 -- (-7578.221) [...7 remote chains...] -- 0:43:10      22150000 -- (-7566.904) [...7 remote chains...] -- 0:43:07       Average standard deviation of split frequencies: 0.008887       22160000 -- (-7553.722) [...7 remote chains...] -- 0:43:04      22170000 -- (-7574.894) [...7 remote chains...] -- 0:43:00      22180000 -- [-7559.841] [...7 remote chains...] -- 0:42:57      22190000 -- (-7568.431) [...7 remote chains...] -- 0:42:54      22200000 -- (-7569.261) [...7 remote chains...] -- 0:42:50       Average standard deviation of split frequencies: 0.008928       22210000 -- (-7556.780) [...7 remote chains...] -- 0:42:47      22220000 -- (-7556.126) [...7 remote chains...] -- 0:42:44      22230000 -- (-7561.744) [...7 remote chains...] -- 0:42:40      22240000 -- (-7545.944) [...7 remote chains...] -- 0:42:37      22250000 -- (-7544.933) [...7 remote chains...] -- 0:42:34       Average standard deviation of split frequencies: 0.008907       22260000 -- [-7546.730] [...7 remote chains...] -- 0:42:30      22270000 -- (-7569.197) [...7 remote chains...] -- 0:42:27      22280000 -- [-7562.425] [...7 remote chains...] -- 0:42:24      22290000 -- [-7547.237] [...7 remote chains...] -- 0:42:20      22300000 -- [-7564.806] [...7 remote chains...] -- 0:42:17       Average standard deviation of split frequencies: 0.008745       22310000 -- [-7548.555] [...7 remote chains...] -- 0:42:14      22320000 -- [-7547.091] [...7 remote chains...] -- 0:42:11      22330000 -- [-7557.297] [...7 remote chains...] -- 0:42:07      22340000 -- [-7546.722] [...7 remote chains...] -- 0:42:04      22350000 -- [-7558.744] [...7 remote chains...] -- 0:42:01       Average standard deviation of split frequencies: 0.008636       22360000 -- [-7556.939] [...7 remote chains...] -- 0:41:57      22370000 -- [-7556.386] [...7 remote chains...] -- 0:41:54      22380000 -- [-7530.985] [...7 remote chains...] -- 0:41:51      22390000 -- [-7531.851] [...7 remote chains...] -- 0:41:48      22400000 -- [-7552.411] [...7 remote chains...] -- 0:41:44       Average standard deviation of split frequencies: 0.008614       22410000 -- (-7569.099) [...7 remote chains...] -- 0:41:41      22420000 -- [-7550.695] [...7 remote chains...] -- 0:41:37      22430000 -- [-7545.056] [...7 remote chains...] -- 0:41:34      22440000 -- [-7548.029] [...7 remote chains...] -- 0:41:31      22450000 -- [-7558.803] [...7 remote chains...] -- 0:41:27       Average standard deviation of split frequencies: 0.008707       22460000 -- [-7549.685] [...7 remote chains...] -- 0:41:24      22470000 -- [-7559.347] [...7 remote chains...] -- 0:41:21      22480000 -- [-7554.921] [...7 remote chains...] -- 0:41:18      22490000 -- [-7563.923] [...7 remote chains...] -- 0:41:14      22500000 -- [-7563.233] [...7 remote chains...] -- 0:41:11       Average standard deviation of split frequencies: 0.008645       22510000 -- [-7556.381] [...7 remote chains...] -- 0:41:08      22520000 -- [-7559.170] [...7 remote chains...] -- 0:41:04      22530000 -- [-7570.539] [...7 remote chains...] -- 0:41:01      22540000 -- [-7557.699] [...7 remote chains...] -- 0:40:58      22550000 -- [-7546.181] [...7 remote chains...] -- 0:40:55       Average standard deviation of split frequencies: 0.008686       22560000 -- [-7545.347] [...7 remote chains...] -- 0:40:51      22570000 -- [-7541.770] [...7 remote chains...] -- 0:40:48      22580000 -- [-7539.680] [...7 remote chains...] -- 0:40:45      22590000 -- [-7555.685] [...7 remote chains...] -- 0:40:41      22600000 -- [-7541.979] [...7 remote chains...] -- 0:40:38       Average standard deviation of split frequencies: 0.008638       22610000 -- [-7570.931] [...7 remote chains...] -- 0:40:35      22620000 -- [-7545.697] [...7 remote chains...] -- 0:40:31      22630000 -- [-7544.228] [...7 remote chains...] -- 0:40:28      22640000 -- [-7553.341] [...7 remote chains...] -- 0:40:25      22650000 -- [-7545.193] [...7 remote chains...] -- 0:40:21       Average standard deviation of split frequencies: 0.008528       22660000 -- [-7555.698] [...7 remote chains...] -- 0:40:18      22670000 -- [-7550.196] [...7 remote chains...] -- 0:40:15      22680000 -- [-7556.003] [...7 remote chains...] -- 0:40:11      22690000 -- [-7577.957] [...7 remote chains...] -- 0:40:08      22700000 -- (-7552.901) [...7 remote chains...] -- 0:40:05       Average standard deviation of split frequencies: 0.008467       22710000 -- [-7557.897] [...7 remote chains...] -- 0:40:02      22720000 -- (-7553.577) [...7 remote chains...] -- 0:39:58      22730000 -- (-7559.268) [...7 remote chains...] -- 0:39:55      22740000 -- (-7564.585) [...7 remote chains...] -- 0:39:51      22750000 -- (-7557.728) [...7 remote chains...] -- 0:39:48       Average standard deviation of split frequencies: 0.008440       22760000 -- (-7588.144) [...7 remote chains...] -- 0:39:45      22770000 -- (-7559.399) [...7 remote chains...] -- 0:39:42      22780000 -- (-7551.637) [...7 remote chains...] -- 0:39:38      22790000 -- (-7553.369) [...7 remote chains...] -- 0:39:35      22800000 -- (-7569.284) [...7 remote chains...] -- 0:39:32       Average standard deviation of split frequencies: 0.008501       22810000 -- (-7570.953) [...7 remote chains...] -- 0:39:28      22820000 -- (-7580.781) [...7 remote chains...] -- 0:39:25      22830000 -- (-7580.240) [...7 remote chains...] -- 0:39:22      22840000 -- (-7578.305) [...7 remote chains...] -- 0:39:18      22850000 -- (-7579.544) [...7 remote chains...] -- 0:39:15       Average standard deviation of split frequencies: 0.008472       22860000 -- (-7571.336) [...7 remote chains...] -- 0:39:12      22870000 -- (-7548.639) [...7 remote chains...] -- 0:39:09      22880000 -- (-7553.574) [...7 remote chains...] -- 0:39:05      22890000 -- (-7553.368) [...7 remote chains...] -- 0:39:02      22900000 -- (-7556.026) [...7 remote chains...] -- 0:38:58       Average standard deviation of split frequencies: 0.008472       22910000 -- (-7568.308) [...7 remote chains...] -- 0:38:55      22920000 -- (-7559.401) [...7 remote chains...] -- 0:38:52      22930000 -- (-7567.046) [...7 remote chains...] -- 0:38:49      22940000 -- (-7580.833) [...7 remote chains...] -- 0:38:45      22950000 -- (-7574.036) [...7 remote chains...] -- 0:38:42       Average standard deviation of split frequencies: 0.008451       22960000 -- (-7570.520) [...7 remote chains...] -- 0:38:39      22970000 -- (-7561.662) [...7 remote chains...] -- 0:38:35      22980000 -- (-7580.782) [...7 remote chains...] -- 0:38:32      22990000 -- (-7578.579) [...7 remote chains...] -- 0:38:29      23000000 -- (-7551.564) [...7 remote chains...] -- 0:38:26       Average standard deviation of split frequencies: 0.008480       23010000 -- (-7565.753) [...7 remote chains...] -- 0:38:22      23020000 -- (-7564.193) [...7 remote chains...] -- 0:38:19      23030000 -- (-7576.275) [...7 remote chains...] -- 0:38:15      23040000 -- (-7560.195) [...7 remote chains...] -- 0:38:12      23050000 -- (-7575.425) [...7 remote chains...] -- 0:38:09       Average standard deviation of split frequencies: 0.008393       23060000 -- (-7560.986) [...7 remote chains...] -- 0:38:06      23070000 -- (-7576.439) [...7 remote chains...] -- 0:38:02      23080000 -- (-7557.505) [...7 remote chains...] -- 0:37:59      23090000 -- (-7569.808) [...7 remote chains...] -- 0:37:56      23100000 -- (-7574.027) [...7 remote chains...] -- 0:37:52       Average standard deviation of split frequencies: 0.008376       23110000 -- (-7596.497) [...7 remote chains...] -- 0:37:49      23120000 -- (-7559.905) [...7 remote chains...] -- 0:37:46      23130000 -- (-7572.052) [...7 remote chains...] -- 0:37:42      23140000 -- (-7547.411) [...7 remote chains...] -- 0:37:39      23150000 -- (-7568.771) [...7 remote chains...] -- 0:37:36       Average standard deviation of split frequencies: 0.008426       23160000 -- (-7549.708) [...7 remote chains...] -- 0:37:33      23170000 -- (-7553.467) [...7 remote chains...] -- 0:37:29      23180000 -- (-7552.778) [...7 remote chains...] -- 0:37:26      23190000 -- (-7565.824) [...7 remote chains...] -- 0:37:22      23200000 -- (-7559.304) [...7 remote chains...] -- 0:37:19       Average standard deviation of split frequencies: 0.008429       23210000 -- (-7573.703) [...7 remote chains...] -- 0:37:16      23220000 -- (-7563.539) [...7 remote chains...] -- 0:37:13      23230000 -- (-7550.201) [...7 remote chains...] -- 0:37:09      23240000 -- (-7557.724) [...7 remote chains...] -- 0:37:06      23250000 -- (-7539.743) [...7 remote chains...] -- 0:37:03       Average standard deviation of split frequencies: 0.008508       23260000 -- (-7546.622) [...7 remote chains...] -- 0:36:59      23270000 -- (-7548.898) [...7 remote chains...] -- 0:36:56      23280000 -- (-7557.637) [...7 remote chains...] -- 0:36:53      23290000 -- (-7553.766) [...7 remote chains...] -- 0:36:50      23300000 -- (-7561.505) [...7 remote chains...] -- 0:36:46       Average standard deviation of split frequencies: 0.008573       23310000 -- (-7572.097) [...7 remote chains...] -- 0:36:43      23320000 -- (-7571.035) [...7 remote chains...] -- 0:36:39      23330000 -- (-7571.520) [...7 remote chains...] -- 0:36:36      23340000 -- (-7554.241) [...7 remote chains...] -- 0:36:33      23350000 -- (-7549.641) [...7 remote chains...] -- 0:36:30       Average standard deviation of split frequencies: 0.008464       23360000 -- (-7536.320) [...7 remote chains...] -- 0:36:26      23370000 -- (-7549.968) [...7 remote chains...] -- 0:36:23      23380000 -- (-7565.575) [...7 remote chains...] -- 0:36:20      23390000 -- (-7576.819) [...7 remote chains...] -- 0:36:16      23400000 -- (-7566.758) [...7 remote chains...] -- 0:36:13       Average standard deviation of split frequencies: 0.008334       23410000 -- (-7561.985) [...7 remote chains...] -- 0:36:10      23420000 -- (-7560.966) [...7 remote chains...] -- 0:36:07      23430000 -- (-7559.031) [...7 remote chains...] -- 0:36:03      23440000 -- (-7537.436) [...7 remote chains...] -- 0:36:00      23450000 -- (-7538.330) [...7 remote chains...] -- 0:35:57       Average standard deviation of split frequencies: 0.008246       23460000 -- (-7545.341) [...7 remote chains...] -- 0:35:53      23470000 -- (-7580.618) [...7 remote chains...] -- 0:35:50      23480000 -- (-7574.836) [...7 remote chains...] -- 0:35:47      23490000 -- (-7593.439) [...7 remote chains...] -- 0:35:43      23500000 -- [-7545.260] [...7 remote chains...] -- 0:35:40       Average standard deviation of split frequencies: 0.008214       23510000 -- (-7558.409) [...7 remote chains...] -- 0:35:37      23520000 -- [-7548.511] [...7 remote chains...] -- 0:35:34      23530000 -- (-7568.140) [...7 remote chains...] -- 0:35:30      23540000 -- (-7547.060) [...7 remote chains...] -- 0:35:27      23550000 -- (-7553.399) [...7 remote chains...] -- 0:35:23       Average standard deviation of split frequencies: 0.008151       23560000 -- (-7573.301) [...7 remote chains...] -- 0:35:20      23570000 -- (-7549.009) [...7 remote chains...] -- 0:35:17      23580000 -- (-7549.393) [...7 remote chains...] -- 0:35:14      23590000 -- [-7557.932] [...7 remote chains...] -- 0:35:10      23600000 -- [-7540.135] [...7 remote chains...] -- 0:35:07       Average standard deviation of split frequencies: 0.007983       23610000 -- (-7562.039) [...7 remote chains...] -- 0:35:04      23620000 -- (-7549.883) [...7 remote chains...] -- 0:35:00      23630000 -- (-7568.603) [...7 remote chains...] -- 0:34:57      23640000 -- (-7564.624) [...7 remote chains...] -- 0:34:54      23650000 -- (-7555.768) [...7 remote chains...] -- 0:34:51       Average standard deviation of split frequencies: 0.007936       23660000 -- (-7570.169) [...7 remote chains...] -- 0:34:47      23670000 -- (-7573.270) [...7 remote chains...] -- 0:34:44      23680000 -- (-7553.756) [...7 remote chains...] -- 0:34:41      23690000 -- (-7560.366) [...7 remote chains...] -- 0:34:37      23700000 -- (-7547.714) [...7 remote chains...] -- 0:34:34       Average standard deviation of split frequencies: 0.007858       23710000 -- (-7563.125) [...7 remote chains...] -- 0:34:31      23720000 -- (-7562.139) [...7 remote chains...] -- 0:34:27      23730000 -- (-7545.036) [...7 remote chains...] -- 0:34:24      23740000 -- (-7545.395) [...7 remote chains...] -- 0:34:21      23750000 -- (-7556.552) [...7 remote chains...] -- 0:34:17       Average standard deviation of split frequencies: 0.007812       23760000 -- (-7564.883) [...7 remote chains...] -- 0:34:14      23770000 -- (-7547.596) [...7 remote chains...] -- 0:34:11      23780000 -- (-7557.709) [...7 remote chains...] -- 0:34:08      23790000 -- (-7540.343) [...7 remote chains...] -- 0:34:04      23800000 -- (-7545.538) [...7 remote chains...] -- 0:34:01       Average standard deviation of split frequencies: 0.007790       23810000 -- (-7567.241) [...7 remote chains...] -- 0:33:58      23820000 -- (-7563.950) [...7 remote chains...] -- 0:33:54      23830000 -- (-7554.137) [...7 remote chains...] -- 0:33:51      23840000 -- (-7543.663) [...7 remote chains...] -- 0:33:48      23850000 -- (-7550.575) [...7 remote chains...] -- 0:33:44       Average standard deviation of split frequencies: 0.007647       23860000 -- (-7564.811) [...7 remote chains...] -- 0:33:41      23870000 -- (-7554.674) [...7 remote chains...] -- 0:33:38      23880000 -- (-7557.867) [...7 remote chains...] -- 0:33:34      23890000 -- (-7568.297) [...7 remote chains...] -- 0:33:31      23900000 -- (-7548.664) [...7 remote chains...] -- 0:33:28       Average standard deviation of split frequencies: 0.007594       23910000 -- (-7548.921) [...7 remote chains...] -- 0:33:25      23920000 -- (-7567.254) [...7 remote chains...] -- 0:33:21      23930000 -- (-7562.002) [...7 remote chains...] -- 0:33:18      23940000 -- (-7559.737) [...7 remote chains...] -- 0:33:15      23950000 -- (-7552.326) [...7 remote chains...] -- 0:33:11       Average standard deviation of split frequencies: 0.007516       23960000 -- (-7531.229) [...7 remote chains...] -- 0:33:08      23970000 -- (-7570.792) [...7 remote chains...] -- 0:33:05      23980000 -- (-7569.893) [...7 remote chains...] -- 0:33:01      23990000 -- (-7560.197) [...7 remote chains...] -- 0:32:58      24000000 -- (-7547.455) [...7 remote chains...] -- 0:32:55       Average standard deviation of split frequencies: 0.007555       24010000 -- (-7551.322) [...7 remote chains...] -- 0:32:52      24020000 -- (-7546.665) [...7 remote chains...] -- 0:32:48      24030000 -- (-7577.758) [...7 remote chains...] -- 0:32:45      24040000 -- (-7574.346) [...7 remote chains...] -- 0:32:42      24050000 -- (-7562.251) [...7 remote chains...] -- 0:32:38       Average standard deviation of split frequencies: 0.007682       24060000 -- (-7567.701) [...7 remote chains...] -- 0:32:35      24070000 -- (-7540.240) [...7 remote chains...] -- 0:32:32      24080000 -- (-7586.467) [...7 remote chains...] -- 0:32:28      24090000 -- (-7574.684) [...7 remote chains...] -- 0:32:25      24100000 -- (-7574.709) [...7 remote chains...] -- 0:32:22       Average standard deviation of split frequencies: 0.007721       24110000 -- [-7541.390] [...7 remote chains...] -- 0:32:18      24120000 -- (-7566.766) [...7 remote chains...] -- 0:32:15      24130000 -- (-7565.098) [...7 remote chains...] -- 0:32:12      24140000 -- (-7548.406) [...7 remote chains...] -- 0:32:09      24150000 -- (-7568.254) [...7 remote chains...] -- 0:32:05       Average standard deviation of split frequencies: 0.007691       24160000 -- (-7548.231) [...7 remote chains...] -- 0:32:02      24170000 -- (-7561.021) [...7 remote chains...] -- 0:31:59      24180000 -- (-7559.477) [...7 remote chains...] -- 0:31:55      24190000 -- [-7545.842] [...7 remote chains...] -- 0:31:52      24200000 -- (-7550.920) [...7 remote chains...] -- 0:31:49       Average standard deviation of split frequencies: 0.007695       24210000 -- (-7584.071) [...7 remote chains...] -- 0:31:46      24220000 -- (-7568.304) [...7 remote chains...] -- 0:31:42      24230000 -- (-7545.269) [...7 remote chains...] -- 0:31:39      24240000 -- (-7554.674) [...7 remote chains...] -- 0:31:36      24250000 -- (-7562.304) [...7 remote chains...] -- 0:31:32       Average standard deviation of split frequencies: 0.007742       24260000 -- (-7553.156) [...7 remote chains...] -- 0:31:29      24270000 -- (-7566.287) [...7 remote chains...] -- 0:31:26      24280000 -- (-7549.250) [...7 remote chains...] -- 0:31:22      24290000 -- (-7551.864) [...7 remote chains...] -- 0:31:19      24300000 -- (-7546.983) [...7 remote chains...] -- 0:31:16       Average standard deviation of split frequencies: 0.007694       24310000 -- (-7563.650) [...7 remote chains...] -- 0:31:12      24320000 -- (-7577.416) [...7 remote chains...] -- 0:31:09      24330000 -- (-7568.632) [...7 remote chains...] -- 0:31:06      24340000 -- (-7552.521) [...7 remote chains...] -- 0:31:03      24350000 -- (-7566.444) [...7 remote chains...] -- 0:30:59       Average standard deviation of split frequencies: 0.007758       24360000 -- (-7570.341) [...7 remote chains...] -- 0:30:56      24370000 -- (-7546.915) [...7 remote chains...] -- 0:30:53      24380000 -- (-7548.854) [...7 remote chains...] -- 0:30:49      24390000 -- (-7556.899) [...7 remote chains...] -- 0:30:46      24400000 -- [-7536.717] [...7 remote chains...] -- 0:30:43       Average standard deviation of split frequencies: 0.007858       24410000 -- (-7557.377) [...7 remote chains...] -- 0:30:39      24420000 -- (-7557.447) [...7 remote chains...] -- 0:30:36      24430000 -- (-7553.218) [...7 remote chains...] -- 0:30:33      24440000 -- (-7554.787) [...7 remote chains...] -- 0:30:29      24450000 -- (-7556.963) [...7 remote chains...] -- 0:30:26       Average standard deviation of split frequencies: 0.007821       24460000 -- (-7556.079) [...7 remote chains...] -- 0:30:23      24470000 -- (-7565.125) [...7 remote chains...] -- 0:30:20      24480000 -- (-7569.780) [...7 remote chains...] -- 0:30:16      24490000 -- (-7572.981) [...7 remote chains...] -- 0:30:13      24500000 -- (-7541.692) [...7 remote chains...] -- 0:30:10       Average standard deviation of split frequencies: 0.007873       24510000 -- [-7541.579] [...7 remote chains...] -- 0:30:06      24520000 -- (-7528.905) [...7 remote chains...] -- 0:30:03      24530000 -- (-7558.339) [...7 remote chains...] -- 0:30:00      24540000 -- (-7576.052) [...7 remote chains...] -- 0:29:57      24550000 -- [-7541.542] [...7 remote chains...] -- 0:29:53       Average standard deviation of split frequencies: 0.007861       24560000 -- (-7567.111) [...7 remote chains...] -- 0:29:50      24570000 -- (-7553.487) [...7 remote chains...] -- 0:29:47      24580000 -- (-7566.435) [...7 remote chains...] -- 0:29:43      24590000 -- (-7566.542) [...7 remote chains...] -- 0:29:40      24600000 -- (-7557.097) [...7 remote chains...] -- 0:29:37       Average standard deviation of split frequencies: 0.007858       24610000 -- (-7554.439) [...7 remote chains...] -- 0:29:34      24620000 -- (-7551.285) [...7 remote chains...] -- 0:29:30      24630000 -- (-7548.395) [...7 remote chains...] -- 0:29:27      24640000 -- (-7559.430) [...7 remote chains...] -- 0:29:23      24650000 -- (-7552.731) [...7 remote chains...] -- 0:29:20       Average standard deviation of split frequencies: 0.007679       24660000 -- [-7544.867] [...7 remote chains...] -- 0:29:17      24670000 -- (-7558.230) [...7 remote chains...] -- 0:29:14      24680000 -- (-7570.488) [...7 remote chains...] -- 0:29:10      24690000 -- (-7572.265) [...7 remote chains...] -- 0:29:07      24700000 -- (-7553.653) [...7 remote chains...] -- 0:29:04       Average standard deviation of split frequencies: 0.007661       24710000 -- (-7566.714) [...7 remote chains...] -- 0:29:00      24720000 -- (-7567.644) [...7 remote chains...] -- 0:28:57      24730000 -- (-7567.659) [...7 remote chains...] -- 0:28:54      24740000 -- (-7575.671) [...7 remote chains...] -- 0:28:51      24750000 -- (-7574.625) [...7 remote chains...] -- 0:28:47       Average standard deviation of split frequencies: 0.007568       24760000 -- (-7559.887) [...7 remote chains...] -- 0:28:44      24770000 -- (-7563.281) [...7 remote chains...] -- 0:28:41      24780000 -- (-7567.029) [...7 remote chains...] -- 0:28:37      24790000 -- (-7564.063) [...7 remote chains...] -- 0:28:34      24800000 -- (-7569.514) [...7 remote chains...] -- 0:28:31       Average standard deviation of split frequencies: 0.007594       24810000 -- (-7555.978) [...7 remote chains...] -- 0:28:28      24820000 -- (-7557.724) [...7 remote chains...] -- 0:28:24      24830000 -- (-7572.708) [...7 remote chains...] -- 0:28:21      24840000 -- (-7573.622) [...7 remote chains...] -- 0:28:17      24850000 -- (-7586.710) [...7 remote chains...] -- 0:28:14       Average standard deviation of split frequencies: 0.007642       24860000 -- (-7582.089) [...7 remote chains...] -- 0:28:11      24870000 -- (-7592.706) [...7 remote chains...] -- 0:28:08      24880000 -- (-7562.638) [...7 remote chains...] -- 0:28:04      24890000 -- (-7563.141) [...7 remote chains...] -- 0:28:01      24900000 -- (-7557.004) [...7 remote chains...] -- 0:27:58       Average standard deviation of split frequencies: 0.007684       24910000 -- (-7559.597) [...7 remote chains...] -- 0:27:54      24920000 -- (-7566.282) [...7 remote chains...] -- 0:27:51      24930000 -- (-7549.420) [...7 remote chains...] -- 0:27:48      24940000 -- (-7567.451) [...7 remote chains...] -- 0:27:45      24950000 -- (-7561.170) [...7 remote chains...] -- 0:27:41       Average standard deviation of split frequencies: 0.007789       24960000 -- (-7557.669) [...7 remote chains...] -- 0:27:38      24970000 -- (-7555.636) [...7 remote chains...] -- 0:27:35      24980000 -- (-7561.933) [...7 remote chains...] -- 0:27:31      24990000 -- (-7558.473) [...7 remote chains...] -- 0:27:28      25000000 -- (-7557.018) [...7 remote chains...] -- 0:27:25       Average standard deviation of split frequencies: 0.007762       25010000 -- (-7563.298) [...7 remote chains...] -- 0:27:21      25020000 -- (-7555.032) [...7 remote chains...] -- 0:27:18      25030000 -- (-7576.513) [...7 remote chains...] -- 0:27:15      25040000 -- (-7570.010) [...7 remote chains...] -- 0:27:12      25050000 -- (-7556.024) [...7 remote chains...] -- 0:27:08       Average standard deviation of split frequencies: 0.007764       25060000 -- (-7568.985) [...7 remote chains...] -- 0:27:05      25070000 -- (-7564.002) [...7 remote chains...] -- 0:27:02      25080000 -- (-7546.615) [...7 remote chains...] -- 0:26:58      25090000 -- (-7550.131) [...7 remote chains...] -- 0:26:55      25100000 -- (-7568.144) [...7 remote chains...] -- 0:26:52       Average standard deviation of split frequencies: 0.007823       25110000 -- (-7579.595) [...7 remote chains...] -- 0:26:48      25120000 -- (-7565.595) [...7 remote chains...] -- 0:26:45      25130000 -- (-7548.280) [...7 remote chains...] -- 0:26:42      25140000 -- (-7565.576) [...7 remote chains...] -- 0:26:39      25150000 -- (-7543.145) [...7 remote chains...] -- 0:26:35       Average standard deviation of split frequencies: 0.007870       25160000 -- (-7572.651) [...7 remote chains...] -- 0:26:32      25170000 -- (-7561.089) [...7 remote chains...] -- 0:26:29      25180000 -- (-7574.426) [...7 remote chains...] -- 0:26:25      25190000 -- (-7583.722) [...7 remote chains...] -- 0:26:22      25200000 -- (-7567.840) [...7 remote chains...] -- 0:26:19       Average standard deviation of split frequencies: 0.007974       25210000 -- (-7560.191) [...7 remote chains...] -- 0:26:15      25220000 -- (-7580.433) [...7 remote chains...] -- 0:26:12      25230000 -- (-7575.797) [...7 remote chains...] -- 0:26:09      25240000 -- (-7571.711) [...7 remote chains...] -- 0:26:06      25250000 -- (-7578.612) [...7 remote chains...] -- 0:26:02       Average standard deviation of split frequencies: 0.007881       25260000 -- (-7571.820) [...7 remote chains...] -- 0:25:59      25270000 -- (-7549.380) [...7 remote chains...] -- 0:25:56      25280000 -- (-7581.994) [...7 remote chains...] -- 0:25:52      25290000 -- (-7555.504) [...7 remote chains...] -- 0:25:49      25300000 -- (-7551.870) [...7 remote chains...] -- 0:25:46       Average standard deviation of split frequencies: 0.007933       25310000 -- (-7551.347) [...7 remote chains...] -- 0:25:43      25320000 -- (-7568.770) [...7 remote chains...] -- 0:25:39      25330000 -- (-7548.168) [...7 remote chains...] -- 0:25:36      25340000 -- (-7551.675) [...7 remote chains...] -- 0:25:32      25350000 -- (-7545.858) [...7 remote chains...] -- 0:25:29       Average standard deviation of split frequencies: 0.007962       25360000 -- (-7556.161) [...7 remote chains...] -- 0:25:26      25370000 -- (-7551.624) [...7 remote chains...] -- 0:25:23      25380000 -- (-7583.010) [...7 remote chains...] -- 0:25:19      25390000 -- (-7561.456) [...7 remote chains...] -- 0:25:16      25400000 -- (-7599.618) [...7 remote chains...] -- 0:25:13       Average standard deviation of split frequencies: 0.007849       25410000 -- (-7591.112) [...7 remote chains...] -- 0:25:09      25420000 -- (-7566.324) [...7 remote chains...] -- 0:25:06      25430000 -- (-7569.319) [...7 remote chains...] -- 0:25:03      25440000 -- (-7578.540) [...7 remote chains...] -- 0:25:00      25450000 -- (-7592.273) [...7 remote chains...] -- 0:24:56       Average standard deviation of split frequencies: 0.007821       25460000 -- (-7604.027) [...7 remote chains...] -- 0:24:53      25470000 -- (-7567.001) [...7 remote chains...] -- 0:24:50      25480000 -- [-7561.578] [...7 remote chains...] -- 0:24:46      25490000 -- [-7551.931] [...7 remote chains...] -- 0:24:43      25500000 -- [-7557.878] [...7 remote chains...] -- 0:24:40       Average standard deviation of split frequencies: 0.007924       25510000 -- [-7556.689] [...7 remote chains...] -- 0:24:37      25520000 -- [-7560.632] [...7 remote chains...] -- 0:24:33      25530000 -- [-7558.680] [...7 remote chains...] -- 0:24:30      25540000 -- (-7545.521) [...7 remote chains...] -- 0:24:27      25550000 -- [-7547.376] [...7 remote chains...] -- 0:24:23       Average standard deviation of split frequencies: 0.008010       25560000 -- [-7550.584] [...7 remote chains...] -- 0:24:20      25570000 -- [-7561.396] [...7 remote chains...] -- 0:24:17      25580000 -- [-7559.280] [...7 remote chains...] -- 0:24:14      25590000 -- [-7543.508] [...7 remote chains...] -- 0:24:10      25600000 -- [-7527.495] [...7 remote chains...] -- 0:24:07       Average standard deviation of split frequencies: 0.008060       25610000 -- [-7545.951] [...7 remote chains...] -- 0:24:04      25620000 -- [-7550.660] [...7 remote chains...] -- 0:24:00      25630000 -- (-7547.072) [...7 remote chains...] -- 0:23:57      25640000 -- [-7552.064] [...7 remote chains...] -- 0:23:54      25650000 -- [-7559.274] [...7 remote chains...] -- 0:23:51       Average standard deviation of split frequencies: 0.008091       25660000 -- [-7546.216] [...7 remote chains...] -- 0:23:47      25670000 -- [-7543.902] [...7 remote chains...] -- 0:23:44      25680000 -- [-7537.084] [...7 remote chains...] -- 0:23:40      25690000 -- [-7529.581] [...7 remote chains...] -- 0:23:37      25700000 -- [-7538.015] [...7 remote chains...] -- 0:23:34       Average standard deviation of split frequencies: 0.008067       25710000 -- (-7545.747) [...7 remote chains...] -- 0:23:31      25720000 -- [-7539.138] [...7 remote chains...] -- 0:23:27      25730000 -- (-7573.221) [...7 remote chains...] -- 0:23:24      25740000 -- [-7551.429] [...7 remote chains...] -- 0:23:21      25750000 -- (-7548.426) [...7 remote chains...] -- 0:23:17       Average standard deviation of split frequencies: 0.008053       25760000 -- (-7549.536) [...7 remote chains...] -- 0:23:14      25770000 -- (-7560.199) [...7 remote chains...] -- 0:23:11      25780000 -- (-7565.731) [...7 remote chains...] -- 0:23:08      25790000 -- (-7558.557) [...7 remote chains...] -- 0:23:04      25800000 -- (-7573.183) [...7 remote chains...] -- 0:23:01       Average standard deviation of split frequencies: 0.008151       25810000 -- (-7570.888) [...7 remote chains...] -- 0:22:58      25820000 -- (-7563.923) [...7 remote chains...] -- 0:22:54      25830000 -- (-7560.575) [...7 remote chains...] -- 0:22:51      25840000 -- (-7563.476) [...7 remote chains...] -- 0:22:48      25850000 -- (-7584.499) [...7 remote chains...] -- 0:22:45       Average standard deviation of split frequencies: 0.008101       25860000 -- (-7576.085) [...7 remote chains...] -- 0:22:41      25870000 -- (-7549.258) [...7 remote chains...] -- 0:22:38      25880000 -- (-7560.261) [...7 remote chains...] -- 0:22:35      25890000 -- (-7554.098) [...7 remote chains...] -- 0:22:31      25900000 -- (-7553.306) [...7 remote chains...] -- 0:22:28       Average standard deviation of split frequencies: 0.008016       25910000 -- (-7559.873) [...7 remote chains...] -- 0:22:25      25920000 -- (-7546.288) [...7 remote chains...] -- 0:22:22      25930000 -- (-7555.630) [...7 remote chains...] -- 0:22:18      25940000 -- (-7553.316) [...7 remote chains...] -- 0:22:15      25950000 -- (-7547.982) [...7 remote chains...] -- 0:22:12       Average standard deviation of split frequencies: 0.007941       25960000 -- (-7561.790) [...7 remote chains...] -- 0:22:08      25970000 -- (-7581.837) [...7 remote chains...] -- 0:22:05      25980000 -- [-7580.481] [...7 remote chains...] -- 0:22:02      25990000 -- [-7566.091] [...7 remote chains...] -- 0:21:58      26000000 -- (-7569.213) [...7 remote chains...] -- 0:21:55       Average standard deviation of split frequencies: 0.008097       26010000 -- [-7553.587] [...7 remote chains...] -- 0:21:52      26020000 -- (-7559.537) [...7 remote chains...] -- 0:21:49      26030000 -- [-7560.232] [...7 remote chains...] -- 0:21:45      26040000 -- [-7561.465] [...7 remote chains...] -- 0:21:42      26050000 -- [-7558.152] [...7 remote chains...] -- 0:21:39       Average standard deviation of split frequencies: 0.008215       26060000 -- (-7556.740) [...7 remote chains...] -- 0:21:35      26070000 -- (-7553.469) [...7 remote chains...] -- 0:21:32      26080000 -- (-7551.688) [...7 remote chains...] -- 0:21:29      26090000 -- (-7556.562) [...7 remote chains...] -- 0:21:25      26100000 -- (-7574.806) [...7 remote chains...] -- 0:21:22       Average standard deviation of split frequencies: 0.008143       26110000 -- (-7558.299) [...7 remote chains...] -- 0:21:19      26120000 -- [-7553.337] [...7 remote chains...] -- 0:21:16      26130000 -- (-7562.942) [...7 remote chains...] -- 0:21:12      26140000 -- (-7561.267) [...7 remote chains...] -- 0:21:09      26150000 -- (-7557.941) [...7 remote chains...] -- 0:21:06       Average standard deviation of split frequencies: 0.008105       26160000 -- (-7552.937) [...7 remote chains...] -- 0:21:02      26170000 -- (-7568.062) [...7 remote chains...] -- 0:20:59      26180000 -- (-7533.569) [...7 remote chains...] -- 0:20:56      26190000 -- (-7566.741) [...7 remote chains...] -- 0:20:52      26200000 -- (-7555.960) [...7 remote chains...] -- 0:20:49       Average standard deviation of split frequencies: 0.008094       26210000 -- (-7551.164) [...7 remote chains...] -- 0:20:46      26220000 -- [-7550.894] [...7 remote chains...] -- 0:20:43      26230000 -- [-7553.305] [...7 remote chains...] -- 0:20:39      26240000 -- (-7534.961) [...7 remote chains...] -- 0:20:36      26250000 -- [-7547.507] [...7 remote chains...] -- 0:20:33       Average standard deviation of split frequencies: 0.008061       26260000 -- (-7558.753) [...7 remote chains...] -- 0:20:29      26270000 -- (-7571.846) [...7 remote chains...] -- 0:20:26      26280000 -- (-7561.491) [...7 remote chains...] -- 0:20:23      26290000 -- (-7566.058) [...7 remote chains...] -- 0:20:20      26300000 -- (-7554.207) [...7 remote chains...] -- 0:20:16       Average standard deviation of split frequencies: 0.008082       26310000 -- (-7575.849) [...7 remote chains...] -- 0:20:13      26320000 -- (-7577.010) [...7 remote chains...] -- 0:20:10      26330000 -- (-7577.320) [...7 remote chains...] -- 0:20:06      26340000 -- (-7560.164) [...7 remote chains...] -- 0:20:03      26350000 -- (-7563.719) [...7 remote chains...] -- 0:20:00       Average standard deviation of split frequencies: 0.008125       26360000 -- (-7551.688) [...7 remote chains...] -- 0:19:57      26370000 -- [-7549.116] [...7 remote chains...] -- 0:19:53      26380000 -- (-7563.181) [...7 remote chains...] -- 0:19:50      26390000 -- (-7551.009) [...7 remote chains...] -- 0:19:47      26400000 -- (-7563.575) [...7 remote chains...] -- 0:19:43       Average standard deviation of split frequencies: 0.008055       26410000 -- (-7576.886) [...7 remote chains...] -- 0:19:40      26420000 -- (-7555.674) [...7 remote chains...] -- 0:19:37      26430000 -- (-7574.864) [...7 remote chains...] -- 0:19:34      26440000 -- (-7573.703) [...7 remote chains...] -- 0:19:30      26450000 -- (-7536.156) [...7 remote chains...] -- 0:19:27       Average standard deviation of split frequencies: 0.007887       26460000 -- (-7584.927) [...7 remote chains...] -- 0:19:24      26470000 -- (-7590.806) [...7 remote chains...] -- 0:19:20      26480000 -- (-7587.553) [...7 remote chains...] -- 0:19:17      26490000 -- [-7563.430] [...7 remote chains...] -- 0:19:14      26500000 -- [-7560.769] [...7 remote chains...] -- 0:19:11       Average standard deviation of split frequencies: 0.007764       26510000 -- [-7557.103] [...7 remote chains...] -- 0:19:07      26520000 -- [-7546.163] [...7 remote chains...] -- 0:19:04      26530000 -- [-7544.804] [...7 remote chains...] -- 0:19:01      26540000 -- [-7556.720] [...7 remote chains...] -- 0:18:57      26550000 -- [-7551.139] [...7 remote chains...] -- 0:18:54       Average standard deviation of split frequencies: 0.007737       26560000 -- [-7564.912] [...7 remote chains...] -- 0:18:51      26570000 -- (-7556.012) [...7 remote chains...] -- 0:18:47      26580000 -- [-7563.583] [...7 remote chains...] -- 0:18:44      26590000 -- [-7559.844] [...7 remote chains...] -- 0:18:41      26600000 -- [-7547.632] [...7 remote chains...] -- 0:18:38       Average standard deviation of split frequencies: 0.007770       26610000 -- (-7584.033) [...7 remote chains...] -- 0:18:34      26620000 -- [-7547.103] [...7 remote chains...] -- 0:18:31      26630000 -- (-7562.365) [...7 remote chains...] -- 0:18:28      26640000 -- (-7567.463) [...7 remote chains...] -- 0:18:24      26650000 -- (-7586.278) [...7 remote chains...] -- 0:18:21       Average standard deviation of split frequencies: 0.007685       26660000 -- (-7590.358) [...7 remote chains...] -- 0:18:18      26670000 -- (-7582.669) [...7 remote chains...] -- 0:18:15      26680000 -- (-7558.533) [...7 remote chains...] -- 0:18:11      26690000 -- (-7557.749) [...7 remote chains...] -- 0:18:08      26700000 -- (-7556.662) [...7 remote chains...] -- 0:18:05       Average standard deviation of split frequencies: 0.007655       26710000 -- (-7544.246) [...7 remote chains...] -- 0:18:01      26720000 -- (-7549.001) [...7 remote chains...] -- 0:17:58      26730000 -- (-7561.516) [...7 remote chains...] -- 0:17:55      26740000 -- (-7575.102) [...7 remote chains...] -- 0:17:51      26750000 -- (-7541.037) [...7 remote chains...] -- 0:17:48       Average standard deviation of split frequencies: 0.007669       26760000 -- (-7554.132) [...7 remote chains...] -- 0:17:45      26770000 -- (-7563.808) [...7 remote chains...] -- 0:17:42      26780000 -- (-7560.176) [...7 remote chains...] -- 0:17:38      26790000 -- (-7567.323) [...7 remote chains...] -- 0:17:35      26800000 -- (-7557.603) [...7 remote chains...] -- 0:17:32       Average standard deviation of split frequencies: 0.007694       26810000 -- (-7575.154) [...7 remote chains...] -- 0:17:28      26820000 -- (-7574.875) [...7 remote chains...] -- 0:17:25      26830000 -- (-7579.769) [...7 remote chains...] -- 0:17:22      26840000 -- (-7568.207) [...7 remote chains...] -- 0:17:18      26850000 -- (-7582.100) [...7 remote chains...] -- 0:17:15       Average standard deviation of split frequencies: 0.007670       26860000 -- (-7572.437) [...7 remote chains...] -- 0:17:12      26870000 -- (-7574.028) [...7 remote chains...] -- 0:17:09      26880000 -- (-7564.403) [...7 remote chains...] -- 0:17:05      26890000 -- (-7565.843) [...7 remote chains...] -- 0:17:02      26900000 -- (-7579.386) [...7 remote chains...] -- 0:16:59       Average standard deviation of split frequencies: 0.007615       26910000 -- (-7583.978) [...7 remote chains...] -- 0:16:55      26920000 -- (-7558.714) [...7 remote chains...] -- 0:16:52      26930000 -- (-7583.036) [...7 remote chains...] -- 0:16:49      26940000 -- (-7549.269) [...7 remote chains...] -- 0:16:46      26950000 -- (-7564.788) [...7 remote chains...] -- 0:16:42       Average standard deviation of split frequencies: 0.007714       26960000 -- (-7565.880) [...7 remote chains...] -- 0:16:39      26970000 -- [-7554.248] [...7 remote chains...] -- 0:16:36      26980000 -- [-7550.899] [...7 remote chains...] -- 0:16:32      26990000 -- [-7553.233] [...7 remote chains...] -- 0:16:29      27000000 -- [-7555.252] [...7 remote chains...] -- 0:16:26       Average standard deviation of split frequencies: 0.007659       27010000 -- [-7555.963] [...7 remote chains...] -- 0:16:23      27020000 -- [-7564.141] [...7 remote chains...] -- 0:16:19      27030000 -- [-7548.454] [...7 remote chains...] -- 0:16:16      27040000 -- [-7553.666] [...7 remote chains...] -- 0:16:13      27050000 -- [-7540.642] [...7 remote chains...] -- 0:16:09       Average standard deviation of split frequencies: 0.007624       27060000 -- (-7567.759) [...7 remote chains...] -- 0:16:06      27070000 -- (-7564.402) [...7 remote chains...] -- 0:16:03      27080000 -- (-7559.786) [...7 remote chains...] -- 0:15:59      27090000 -- (-7572.854) [...7 remote chains...] -- 0:15:56      27100000 -- (-7559.269) [...7 remote chains...] -- 0:15:53       Average standard deviation of split frequencies: 0.007858       27110000 -- (-7560.743) [...7 remote chains...] -- 0:15:50      27120000 -- [-7546.378] [...7 remote chains...] -- 0:15:46      27130000 -- (-7560.834) [...7 remote chains...] -- 0:15:43      27140000 -- [-7538.142] [...7 remote chains...] -- 0:15:40      27150000 -- (-7539.054) [...7 remote chains...] -- 0:15:36       Average standard deviation of split frequencies: 0.007867       27160000 -- [-7548.738] [...7 remote chains...] -- 0:15:33      27170000 -- (-7565.759) [...7 remote chains...] -- 0:15:30      27180000 -- (-7571.282) [...7 remote chains...] -- 0:15:27      27190000 -- (-7551.666) [...7 remote chains...] -- 0:15:23      27200000 -- (-7559.438) [...7 remote chains...] -- 0:15:20       Average standard deviation of split frequencies: 0.007862       27210000 -- (-7582.793) [...7 remote chains...] -- 0:15:17      27220000 -- (-7563.058) [...7 remote chains...] -- 0:15:13      27230000 -- (-7583.931) [...7 remote chains...] -- 0:15:10      27240000 -- (-7540.040) [...7 remote chains...] -- 0:15:07      27250000 -- (-7558.777) [...7 remote chains...] -- 0:15:04       Average standard deviation of split frequencies: 0.007935       27260000 -- (-7558.679) [...7 remote chains...] -- 0:15:00      27270000 -- (-7538.132) [...7 remote chains...] -- 0:14:57      27280000 -- (-7544.599) [...7 remote chains...] -- 0:14:54      27290000 -- (-7531.286) [...7 remote chains...] -- 0:14:50      27300000 -- (-7582.229) [...7 remote chains...] -- 0:14:47       Average standard deviation of split frequencies: 0.007866       27310000 -- (-7581.814) [...7 remote chains...] -- 0:14:44      27320000 -- (-7592.291) [...7 remote chains...] -- 0:14:41      27330000 -- (-7559.550) [...7 remote chains...] -- 0:14:37      27340000 -- (-7570.929) [...7 remote chains...] -- 0:14:34      27350000 -- (-7565.886) [...7 remote chains...] -- 0:14:31       Average standard deviation of split frequencies: 0.007841       27360000 -- (-7561.400) [...7 remote chains...] -- 0:14:27      27370000 -- [-7552.558] [...7 remote chains...] -- 0:14:24      27380000 -- (-7560.988) [...7 remote chains...] -- 0:14:21      27390000 -- (-7545.310) [...7 remote chains...] -- 0:14:17      27400000 -- (-7560.915) [...7 remote chains...] -- 0:14:14       Average standard deviation of split frequencies: 0.007847       27410000 -- (-7558.953) [...7 remote chains...] -- 0:14:11      27420000 -- (-7558.768) [...7 remote chains...] -- 0:14:08      27430000 -- (-7552.545) [...7 remote chains...] -- 0:14:04      27440000 -- [-7543.473] [...7 remote chains...] -- 0:14:01      27450000 -- [-7554.905] [...7 remote chains...] -- 0:13:58       Average standard deviation of split frequencies: 0.007768       27460000 -- (-7559.826) [...7 remote chains...] -- 0:13:54      27470000 -- (-7561.205) [...7 remote chains...] -- 0:13:51      27480000 -- (-7557.279) [...7 remote chains...] -- 0:13:48      27490000 -- (-7599.662) [...7 remote chains...] -- 0:13:45      27500000 -- (-7553.688) [...7 remote chains...] -- 0:13:41       Average standard deviation of split frequencies: 0.007716       27510000 -- (-7566.188) [...7 remote chains...] -- 0:13:38      27520000 -- (-7561.461) [...7 remote chains...] -- 0:13:35      27530000 -- (-7562.745) [...7 remote chains...] -- 0:13:31      27540000 -- (-7545.073) [...7 remote chains...] -- 0:13:28      27550000 -- (-7547.757) [...7 remote chains...] -- 0:13:25       Average standard deviation of split frequencies: 0.007651       27560000 -- [-7543.749] [...7 remote chains...] -- 0:13:22      27570000 -- [-7543.449] [...7 remote chains...] -- 0:13:18      27580000 -- [-7542.840] [...7 remote chains...] -- 0:13:15      27590000 -- (-7545.345) [...7 remote chains...] -- 0:13:12      27600000 -- (-7541.249) [...7 remote chains...] -- 0:13:08       Average standard deviation of split frequencies: 0.007689       27610000 -- [-7530.910] [...7 remote chains...] -- 0:13:05      27620000 -- [-7545.842] [...7 remote chains...] -- 0:13:02      27630000 -- [-7562.266] [...7 remote chains...] -- 0:12:58      27640000 -- [-7549.742] [...7 remote chains...] -- 0:12:55      27650000 -- (-7559.622) [...7 remote chains...] -- 0:12:52       Average standard deviation of split frequencies: 0.007635       27660000 -- (-7533.567) [...7 remote chains...] -- 0:12:49      27670000 -- (-7568.848) [...7 remote chains...] -- 0:12:45      27680000 -- (-7547.382) [...7 remote chains...] -- 0:12:42      27690000 -- (-7553.418) [...7 remote chains...] -- 0:12:39      27700000 -- (-7575.208) [...7 remote chains...] -- 0:12:35       Average standard deviation of split frequencies: 0.007554       27710000 -- (-7571.717) [...7 remote chains...] -- 0:12:32      27720000 -- (-7585.939) [...7 remote chains...] -- 0:12:29      27730000 -- (-7579.429) [...7 remote chains...] -- 0:12:26      27740000 -- (-7583.269) [...7 remote chains...] -- 0:12:22      27750000 -- (-7563.886) [...7 remote chains...] -- 0:12:19       Average standard deviation of split frequencies: 0.007490       27760000 -- (-7582.051) [...7 remote chains...] -- 0:12:16      27770000 -- (-7572.335) [...7 remote chains...] -- 0:12:12      27780000 -- (-7543.087) [...7 remote chains...] -- 0:12:09      27790000 -- (-7590.074) [...7 remote chains...] -- 0:12:06      27800000 -- (-7570.563) [...7 remote chains...] -- 0:12:03       Average standard deviation of split frequencies: 0.007491       27810000 -- (-7561.104) [...7 remote chains...] -- 0:11:59      27820000 -- (-7567.063) [...7 remote chains...] -- 0:11:56      27830000 -- (-7540.916) [...7 remote chains...] -- 0:11:53      27840000 -- (-7575.949) [...7 remote chains...] -- 0:11:49      27850000 -- (-7571.126) [...7 remote chains...] -- 0:11:46       Average standard deviation of split frequencies: 0.007470       27860000 -- (-7569.637) [...7 remote chains...] -- 0:11:43      27870000 -- [-7567.656] [...7 remote chains...] -- 0:11:40      27880000 -- (-7584.933) [...7 remote chains...] -- 0:11:36      27890000 -- (-7555.814) [...7 remote chains...] -- 0:11:33      27900000 -- (-7561.001) [...7 remote chains...] -- 0:11:30       Average standard deviation of split frequencies: 0.007502       27910000 -- (-7566.006) [...7 remote chains...] -- 0:11:26      27920000 -- (-7567.807) [...7 remote chains...] -- 0:11:23      27930000 -- (-7574.223) [...7 remote chains...] -- 0:11:20      27940000 -- (-7577.651) [...7 remote chains...] -- 0:11:17      27950000 -- (-7549.218) [...7 remote chains...] -- 0:11:13       Average standard deviation of split frequencies: 0.007477       27960000 -- (-7562.600) [...7 remote chains...] -- 0:11:10      27970000 -- (-7552.885) [...7 remote chains...] -- 0:11:07      27980000 -- (-7556.889) [...7 remote chains...] -- 0:11:03      27990000 -- (-7553.857) [...7 remote chains...] -- 0:11:00      28000000 -- (-7564.408) [...7 remote chains...] -- 0:10:57       Average standard deviation of split frequencies: 0.007441       28010000 -- (-7551.038) [...7 remote chains...] -- 0:10:54      28020000 -- (-7545.368) [...7 remote chains...] -- 0:10:50      28030000 -- (-7565.048) [...7 remote chains...] -- 0:10:47      28040000 -- (-7570.361) [...7 remote chains...] -- 0:10:44      28050000 -- (-7575.940) [...7 remote chains...] -- 0:10:40       Average standard deviation of split frequencies: 0.007545       28060000 -- (-7568.793) [...7 remote chains...] -- 0:10:37      28070000 -- (-7555.558) [...7 remote chains...] -- 0:10:34      28080000 -- (-7601.182) [...7 remote chains...] -- 0:10:30      28090000 -- [-7562.052] [...7 remote chains...] -- 0:10:27      28100000 -- (-7559.464) [...7 remote chains...] -- 0:10:24       Average standard deviation of split frequencies: 0.007644       28110000 -- (-7541.403) [...7 remote chains...] -- 0:10:21      28120000 -- (-7545.206) [...7 remote chains...] -- 0:10:17      28130000 -- (-7544.945) [...7 remote chains...] -- 0:10:14      28140000 -- (-7552.922) [...7 remote chains...] -- 0:10:11      28150000 -- [-7567.179] [...7 remote chains...] -- 0:10:07       Average standard deviation of split frequencies: 0.007735       28160000 -- [-7550.760] [...7 remote chains...] -- 0:10:04      28170000 -- [-7546.675] [...7 remote chains...] -- 0:10:01      28180000 -- [-7542.352] [...7 remote chains...] -- 0:09:58      28190000 -- [-7535.680] [...7 remote chains...] -- 0:09:54      28200000 -- (-7575.237) [...7 remote chains...] -- 0:09:51       Average standard deviation of split frequencies: 0.007740       28210000 -- (-7534.189) [...7 remote chains...] -- 0:09:48      28220000 -- (-7545.505) [...7 remote chains...] -- 0:09:44      28230000 -- [-7554.020] [...7 remote chains...] -- 0:09:41      28240000 -- (-7553.332) [...7 remote chains...] -- 0:09:38      28250000 -- (-7562.041) [...7 remote chains...] -- 0:09:35       Average standard deviation of split frequencies: 0.007798       28260000 -- [-7561.594] [...7 remote chains...] -- 0:09:31      28270000 -- [-7539.919] [...7 remote chains...] -- 0:09:28      28280000 -- (-7560.998) [...7 remote chains...] -- 0:09:25      28290000 -- [-7545.501] [...7 remote chains...] -- 0:09:21      28300000 -- [-7541.974] [...7 remote chains...] -- 0:09:18       Average standard deviation of split frequencies: 0.007796       28310000 -- [-7549.616] [...7 remote chains...] -- 0:09:15      28320000 -- [-7560.802] [...7 remote chains...] -- 0:09:12      28330000 -- [-7551.030] [...7 remote chains...] -- 0:09:08      28340000 -- [-7545.328] [...7 remote chains...] -- 0:09:05      28350000 -- [-7550.843] [...7 remote chains...] -- 0:09:02       Average standard deviation of split frequencies: 0.007908       28360000 -- [-7558.467] [...7 remote chains...] -- 0:08:58      28370000 -- [-7538.647] [...7 remote chains...] -- 0:08:55      28380000 -- [-7543.490] [...7 remote chains...] -- 0:08:52      28390000 -- [-7542.529] [...7 remote chains...] -- 0:08:49      28400000 -- [-7548.156] [...7 remote chains...] -- 0:08:45       Average standard deviation of split frequencies: 0.007926       28410000 -- (-7566.633) [...7 remote chains...] -- 0:08:42      28420000 -- (-7570.299) [...7 remote chains...] -- 0:08:39      28430000 -- (-7555.262) [...7 remote chains...] -- 0:08:35      28440000 -- [-7563.034] [...7 remote chains...] -- 0:08:32      28450000 -- (-7568.122) [...7 remote chains...] -- 0:08:29       Average standard deviation of split frequencies: 0.007888       28460000 -- (-7552.558) [...7 remote chains...] -- 0:08:26      28470000 -- (-7567.543) [...7 remote chains...] -- 0:08:22      28480000 -- (-7560.006) [...7 remote chains...] -- 0:08:19      28490000 -- [-7566.256] [...7 remote chains...] -- 0:08:16      28500000 -- (-7560.141) [...7 remote chains...] -- 0:08:12       Average standard deviation of split frequencies: 0.007915       28510000 -- (-7584.691) [...7 remote chains...] -- 0:08:09      28520000 -- (-7557.945) [...7 remote chains...] -- 0:08:06      28530000 -- (-7579.526) [...7 remote chains...] -- 0:08:03      28540000 -- (-7588.336) [...7 remote chains...] -- 0:07:59      28550000 -- (-7566.908) [...7 remote chains...] -- 0:07:56       Average standard deviation of split frequencies: 0.007852       28560000 -- (-7567.227) [...7 remote chains...] -- 0:07:53      28570000 -- (-7590.323) [...7 remote chains...] -- 0:07:49      28580000 -- (-7559.847) [...7 remote chains...] -- 0:07:46      28590000 -- (-7571.116) [...7 remote chains...] -- 0:07:43      28600000 -- (-7552.820) [...7 remote chains...] -- 0:07:40       Average standard deviation of split frequencies: 0.007824       28610000 -- [-7536.833] [...7 remote chains...] -- 0:07:36      28620000 -- (-7547.833) [...7 remote chains...] -- 0:07:33      28630000 -- (-7549.970) [...7 remote chains...] -- 0:07:30      28640000 -- (-7573.960) [...7 remote chains...] -- 0:07:26      28650000 -- (-7560.266) [...7 remote chains...] -- 0:07:23       Average standard deviation of split frequencies: 0.007831       28660000 -- (-7572.567) [...7 remote chains...] -- 0:07:20      28670000 -- (-7575.436) [...7 remote chains...] -- 0:07:16      28680000 -- (-7575.490) [...7 remote chains...] -- 0:07:13      28690000 -- (-7566.628) [...7 remote chains...] -- 0:07:10      28700000 -- (-7560.048) [...7 remote chains...] -- 0:07:07       Average standard deviation of split frequencies: 0.007963       28710000 -- (-7572.819) [...7 remote chains...] -- 0:07:03      28720000 -- (-7554.921) [...7 remote chains...] -- 0:07:00      28730000 -- (-7550.955) [...7 remote chains...] -- 0:06:57      28740000 -- (-7562.358) [...7 remote chains...] -- 0:06:53      28750000 -- (-7574.529) [...7 remote chains...] -- 0:06:50       Average standard deviation of split frequencies: 0.008077       28760000 -- (-7558.578) [...7 remote chains...] -- 0:06:47      28770000 -- (-7554.092) [...7 remote chains...] -- 0:06:44      28780000 -- (-7577.586) [...7 remote chains...] -- 0:06:40      28790000 -- (-7559.528) [...7 remote chains...] -- 0:06:37      28800000 -- (-7590.627) [...7 remote chains...] -- 0:06:34       Average standard deviation of split frequencies: 0.008161       28810000 -- (-7582.234) [...7 remote chains...] -- 0:06:30      28820000 -- (-7593.266) [...7 remote chains...] -- 0:06:27      28830000 -- (-7579.386) [...7 remote chains...] -- 0:06:24      28840000 -- (-7571.112) [...7 remote chains...] -- 0:06:21      28850000 -- (-7574.020) [...7 remote chains...] -- 0:06:17       Average standard deviation of split frequencies: 0.008089       28860000 -- (-7574.543) [...7 remote chains...] -- 0:06:14      28870000 -- (-7586.876) [...7 remote chains...] -- 0:06:11      28880000 -- (-7564.302) [...7 remote chains...] -- 0:06:07      28890000 -- (-7565.175) [...7 remote chains...] -- 0:06:04      28900000 -- (-7580.071) [...7 remote chains...] -- 0:06:01       Average standard deviation of split frequencies: 0.008069       28910000 -- (-7550.478) [...7 remote chains...] -- 0:05:58      28920000 -- (-7567.803) [...7 remote chains...] -- 0:05:54      28930000 -- (-7552.863) [...7 remote chains...] -- 0:05:51      28940000 -- (-7536.442) [...7 remote chains...] -- 0:05:48      28950000 -- (-7564.608) [...7 remote chains...] -- 0:05:44       Average standard deviation of split frequencies: 0.008120       28960000 -- (-7579.459) [...7 remote chains...] -- 0:05:41      28970000 -- (-7571.413) [...7 remote chains...] -- 0:05:38      28980000 -- (-7593.437) [...7 remote chains...] -- 0:05:35      28990000 -- (-7561.151) [...7 remote chains...] -- 0:05:31      29000000 -- (-7557.696) [...7 remote chains...] -- 0:05:28       Average standard deviation of split frequencies: 0.008144       29010000 -- (-7546.000) [...7 remote chains...] -- 0:05:25      29020000 -- (-7557.067) [...7 remote chains...] -- 0:05:21      29030000 -- (-7570.178) [...7 remote chains...] -- 0:05:18      29040000 -- (-7569.775) [...7 remote chains...] -- 0:05:15      29050000 -- (-7577.876) [...7 remote chains...] -- 0:05:12       Average standard deviation of split frequencies: 0.008143       29060000 -- (-7567.605) [...7 remote chains...] -- 0:05:08      29070000 -- (-7580.156) [...7 remote chains...] -- 0:05:05      29080000 -- (-7555.155) [...7 remote chains...] -- 0:05:02      29090000 -- (-7553.108) [...7 remote chains...] -- 0:04:58      29100000 -- (-7568.712) [...7 remote chains...] -- 0:04:55       Average standard deviation of split frequencies: 0.008152       29110000 -- (-7549.798) [...7 remote chains...] -- 0:04:52      29120000 -- (-7556.671) [...7 remote chains...] -- 0:04:49      29130000 -- (-7565.158) [...7 remote chains...] -- 0:04:45      29140000 -- (-7555.560) [...7 remote chains...] -- 0:04:42      29150000 -- (-7566.658) [...7 remote chains...] -- 0:04:39       Average standard deviation of split frequencies: 0.008141       29160000 -- (-7570.659) [...7 remote chains...] -- 0:04:35      29170000 -- (-7562.020) [...7 remote chains...] -- 0:04:32      29180000 -- (-7552.889) [...7 remote chains...] -- 0:04:29      29190000 -- (-7562.357) [...7 remote chains...] -- 0:04:26      29200000 -- (-7551.624) [...7 remote chains...] -- 0:04:22       Average standard deviation of split frequencies: 0.008123       29210000 -- (-7552.513) [...7 remote chains...] -- 0:04:19      29220000 -- (-7565.316) [...7 remote chains...] -- 0:04:16      29230000 -- (-7554.899) [...7 remote chains...] -- 0:04:12      29240000 -- (-7548.554) [...7 remote chains...] -- 0:04:09      29250000 -- (-7560.468) [...7 remote chains...] -- 0:04:06       Average standard deviation of split frequencies: 0.008094       29260000 -- (-7539.723) [...7 remote chains...] -- 0:04:03      29270000 -- (-7554.876) [...7 remote chains...] -- 0:03:59      29280000 -- (-7573.729) [...7 remote chains...] -- 0:03:56      29290000 -- [-7559.239] [...7 remote chains...] -- 0:03:53      29300000 -- (-7553.338) [...7 remote chains...] -- 0:03:49       Average standard deviation of split frequencies: 0.008112       29310000 -- (-7569.875) [...7 remote chains...] -- 0:03:46      29320000 -- (-7545.405) [...7 remote chains...] -- 0:03:43      29330000 -- (-7563.383) [...7 remote chains...] -- 0:03:40      29340000 -- (-7546.736) [...7 remote chains...] -- 0:03:36      29350000 -- (-7550.119) [...7 remote chains...] -- 0:03:33       Average standard deviation of split frequencies: 0.008133       29360000 -- (-7558.040) [...7 remote chains...] -- 0:03:30      29370000 -- (-7566.660) [...7 remote chains...] -- 0:03:26      29380000 -- (-7555.788) [...7 remote chains...] -- 0:03:23      29390000 -- (-7538.656) [...7 remote chains...] -- 0:03:20      29400000 -- (-7549.492) [...7 remote chains...] -- 0:03:17       Average standard deviation of split frequencies: 0.008154       29410000 -- (-7541.529) [...7 remote chains...] -- 0:03:13      29420000 -- (-7544.001) [...7 remote chains...] -- 0:03:10      29430000 -- (-7544.900) [...7 remote chains...] -- 0:03:07      29440000 -- (-7552.557) [...7 remote chains...] -- 0:03:03      29450000 -- (-7555.838) [...7 remote chains...] -- 0:03:00       Average standard deviation of split frequencies: 0.008143       29460000 -- (-7554.760) [...7 remote chains...] -- 0:02:57      29470000 -- (-7553.834) [...7 remote chains...] -- 0:02:54      29480000 -- (-7562.474) [...7 remote chains...] -- 0:02:50      29490000 -- (-7547.011) [...7 remote chains...] -- 0:02:47      29500000 -- (-7564.201) [...7 remote chains...] -- 0:02:44       Average standard deviation of split frequencies: 0.008282       29510000 -- (-7550.126) [...7 remote chains...] -- 0:02:40      29520000 -- (-7529.525) [...7 remote chains...] -- 0:02:37      29530000 -- (-7536.344) [...7 remote chains...] -- 0:02:34      29540000 -- (-7583.031) [...7 remote chains...] -- 0:02:31      29550000 -- (-7547.769) [...7 remote chains...] -- 0:02:27       Average standard deviation of split frequencies: 0.008405       29560000 -- (-7570.046) [...7 remote chains...] -- 0:02:24      29570000 -- (-7561.200) [...7 remote chains...] -- 0:02:21      29580000 -- (-7542.977) [...7 remote chains...] -- 0:02:17      29590000 -- (-7543.221) [...7 remote chains...] -- 0:02:14      29600000 -- (-7551.457) [...7 remote chains...] -- 0:02:11       Average standard deviation of split frequencies: 0.008507       29610000 -- (-7536.815) [...7 remote chains...] -- 0:02:08      29620000 -- (-7577.078) [...7 remote chains...] -- 0:02:04      29630000 -- (-7596.503) [...7 remote chains...] -- 0:02:01      29640000 -- (-7563.753) [...7 remote chains...] -- 0:01:58      29650000 -- [-7571.030] [...7 remote chains...] -- 0:01:54       Average standard deviation of split frequencies: 0.008542       29660000 -- [-7556.405] [...7 remote chains...] -- 0:01:51      29670000 -- [-7547.404] [...7 remote chains...] -- 0:01:48      29680000 -- (-7535.776) [...7 remote chains...] -- 0:01:45      29690000 -- [-7557.767] [...7 remote chains...] -- 0:01:41      29700000 -- [-7554.569] [...7 remote chains...] -- 0:01:38       Average standard deviation of split frequencies: 0.008479       29710000 -- [-7545.544] [...7 remote chains...] -- 0:01:35      29720000 -- [-7560.831] [...7 remote chains...] -- 0:01:31      29730000 -- [-7562.448] [...7 remote chains...] -- 0:01:28      29740000 -- [-7559.711] [...7 remote chains...] -- 0:01:25      29750000 -- (-7573.264) [...7 remote chains...] -- 0:01:22       Average standard deviation of split frequencies: 0.008352       29760000 -- (-7562.805) [...7 remote chains...] -- 0:01:18      29770000 -- [-7545.449] [...7 remote chains...] -- 0:01:15      29780000 -- [-7545.558] [...7 remote chains...] -- 0:01:12      29790000 -- [-7550.521] [...7 remote chains...] -- 0:01:08      29800000 -- [-7556.060] [...7 remote chains...] -- 0:01:05       Average standard deviation of split frequencies: 0.008409       29810000 -- [-7558.073] [...7 remote chains...] -- 0:01:02      29820000 -- [-7552.146] [...7 remote chains...] -- 0:00:59      29830000 -- [-7549.003] [...7 remote chains...] -- 0:00:55      29840000 -- [-7545.592] [...7 remote chains...] -- 0:00:52      29850000 -- [-7548.123] [...7 remote chains...] -- 0:00:49       Average standard deviation of split frequencies: 0.008385       29860000 -- [-7560.849] [...7 remote chains...] -- 0:00:45      29870000 -- [-7548.047] [...7 remote chains...] -- 0:00:42      29880000 -- [-7550.947] [...7 remote chains...] -- 0:00:39      29890000 -- [-7564.817] [...7 remote chains...] -- 0:00:36      29900000 -- [-7559.222] [...7 remote chains...] -- 0:00:32       Average standard deviation of split frequencies: 0.008245       29910000 -- [-7569.225] [...7 remote chains...] -- 0:00:29      29920000 -- [-7556.703] [...7 remote chains...] -- 0:00:26      29930000 -- [-7539.136] [...7 remote chains...] -- 0:00:22      29940000 -- [-7581.739] [...7 remote chains...] -- 0:00:19      29950000 -- [-7548.014] [...7 remote chains...] -- 0:00:16       Average standard deviation of split frequencies: 0.008193       29960000 -- [-7542.172] [...7 remote chains...] -- 0:00:13      29970000 -- [-7546.267] [...7 remote chains...] -- 0:00:09      29980000 -- [-7546.662] [...7 remote chains...] -- 0:00:06      29990000 -- [-7558.857] [...7 remote chains...] -- 0:00:03      30000000 -- [-7540.551] [...7 remote chains...] -- 0:00:00       Average standard deviation of split frequencies: 0.008259       Analysis completed in 2 hours 44 mins 14 seconds      Analysis used 9852.34 seconds of CPU time on processor 0      Likelihood of best state for "cold" chain of run 1 was -7508.71      Likelihood of best state for "cold" chain of run 2 was -7511.71       Acceptance rates for the moves in the "cold" chain of run 1:         With prob.   (last 1000)   chain accepted proposals by move            31.3 %     ( 27 %)     Dirichlet(Tratio{4})            24.7 %     ( 24 %)     Dirichlet(Tratio{5})            24.8 %     ( 26 %)     Dirichlet(Pi{4})            26.3 %     ( 26 %)     Slider(Pi{4})            21.4 %     ( 27 %)     Dirichlet(Pi{5})            25.1 %     ( 25 %)     Slider(Pi{5})            31.4 %     ( 25 %)     Multiplier(Alpha{1,2,3})            29.6 %     ( 24 %)     Multiplier(Alpha{4})            25.3 %     ( 23 %)     Multiplier(Alpha{5})            25.0 %     ( 26 %)     Dirichlet(Ratemultiplier{all})            70.4 %     ( 64 %)     Slider(Ratemultiplier{all})             8.9 %     ( 10 %)     ExtSPRClock(Tau{all},V{all})            25.9 %     ( 29 %)     NNIClock(Tau{all},V{all})             4.2 %     (  5 %)     ParsSPRClock(Tau{all},V{all})             7.1 %     (  5 %)     AddBranch(V{all})             7.1 %     (  5 %)     DelBranch(V{all})            46.8 %     ( 56 %)     NodesliderClock(V{all})            35.5 %     ( 22 %)     TreeStretch(V{all})            43.7 %     ( 24 %)     Multiplier(Net_speciation{all})            24.4 %     ( 34 %)     Slider(Relative_extinction{all})            23.4 %     ( 31 %)     Slider(Relative_fossilization{all})            61.2 %     ( 26 %)     Multiplier(ILNvar{1})            59.5 %     ( 25 %)     Multiplier(ILNvar{2})            61.8 %     ( 26 %)     Multiplier(ILNvar{3})            59.1 %     ( 25 %)     Multiplier(ILNvar{4,5})            30.4 %     ( 16 %)     Multiplier(IlnBrlens{1})            29.8 %     ( 37 %)     Multiplier(IlnBrlens{2})            30.1 %     ( 27 %)     Multiplier(IlnBrlens{3})            29.9 %     ( 16 %)     Multiplier(IlnBrlens{4,5})            25.0 %     ( 22 %)     Multiplier(Clockrate{all})       Acceptance rates for the moves in the "cold" chain of run 2:         With prob.   (last 1000)   chain accepted proposals by move            31.3 %     ( 26 %)     Dirichlet(Tratio{4})            24.7 %     ( 25 %)     Dirichlet(Tratio{5})            24.8 %     ( 24 %)     Dirichlet(Pi{4})            26.4 %     ( 24 %)     Slider(Pi{4})            21.7 %     ( 23 %)     Dirichlet(Pi{5})            25.0 %     ( 26 %)     Slider(Pi{5})            31.0 %     ( 26 %)     Multiplier(Alpha{1,2,3})            29.7 %     ( 28 %)     Multiplier(Alpha{4})            25.2 %     ( 25 %)     Multiplier(Alpha{5})            24.7 %     ( 22 %)     Dirichlet(Ratemultiplier{all})            69.8 %     ( 55 %)     Slider(Ratemultiplier{all})             8.9 %     ( 10 %)     ExtSPRClock(Tau{all},V{all})            26.0 %     ( 29 %)     NNIClock(Tau{all},V{all})             4.2 %     (  5 %)     ParsSPRClock(Tau{all},V{all})             7.3 %     (  7 %)     AddBranch(V{all})             7.2 %     (  7 %)     DelBranch(V{all})            46.8 %     ( 49 %)     NodesliderClock(V{all})            35.3 %     ( 23 %)     TreeStretch(V{all})            43.7 %     ( 24 %)     Multiplier(Net_speciation{all})            24.6 %     ( 24 %)     Slider(Relative_extinction{all})            23.7 %     ( 26 %)     Slider(Relative_fossilization{all})            61.2 %     ( 24 %)     Multiplier(ILNvar{1})            58.8 %     ( 27 %)     Multiplier(ILNvar{2})            61.8 %     ( 28 %)     Multiplier(ILNvar{3})            59.1 %     ( 25 %)     Multiplier(ILNvar{4,5})            30.7 %     ( 22 %)     Multiplier(IlnBrlens{1})            30.5 %     ( 31 %)     Multiplier(IlnBrlens{2})            30.7 %     ( 21 %)     Multiplier(IlnBrlens{3})            30.0 %     ( 23 %)     Multiplier(IlnBrlens{4,5})            25.0 %     ( 26 %)     Multiplier(Clockrate{all})       Chain swap information for run 1:                     1        2        3        4            --------------------------------------         1 |              0.18     0.01     0.00          2 |  4998643              0.24     0.02          3 |  5003106  4998547              0.27          4 |  5001015  5000414  4998275                 Chain swap information for run 2:                     1        2        3        4            --------------------------------------         1 |              0.16     0.01     0.00          2 |  5000048              0.24     0.02          3 |  4996171  4998987              0.27          4 |  5000596  5004165  5000033                 Upper diagonal: Proportion of successful state exchanges between chains      Lower diagonal: Number of attempted state exchanges between chains       Chain information:         ID -- Heat        -----------         1 -- 1.00  (cold chain)         2 -- 0.93          3 -- 0.88          4 -- 0.83        Heat = 1 / (1 + T * (ID - 1))         (where T = 0.07 is the temperature and ID is the chain number)       Setting sumt output file name to "run.te.maj"      Summarizing trees in files "run.te.run1.t" and "run.te.run2.t"      Using relative burnin ('relburnin=yes'), discarding the first 25 % of sampled trees      Writing statistics to files run.te.maj.<parts|tstat|vstat|trprobs|con>      Examining first file ...      Found one tree block in file "run.te.run1.t" with 75001 trees in last block      Expecting the same number of trees in the last tree block of all files       Tree reading status:       0      10      20      30      40      50      60      70      80      90     100      v-------v-------v-------v-------v-------v-------v-------v-------v-------v-------v      *********************************************************************************       Read a total of 150002 trees in 2 files (sampling 112502 of them)         (Each file contained 75001 trees of which 56251 were sampled)                                                                                         General explanation:                                                                                                                                                   In an unrooted tree, a taxon bipartition (split) is specified by removing a         branch, thereby dividing the species into those to the left and those to the        right of the branch. Here, taxa to one side of the removed branch are denoted       '.' and those to the other side are denoted '*'. Specifically, the '.' symbol       is used for the taxa on the same side as the outgroup.                                                                                                                 In a rooted or clock tree, the tree is rooted using the model and not by            reference to an outgroup. Each bipartition therefore corresponds to a clade,        that is, a group that includes all the descendants of a particular branch in        the tree.  Taxa that are included in each clade are denoted using '*', and          taxa that are not included are denoted using the '.' symbol.                                                                                                           The output first includes a key to all the bipartitions with frequency larger       or equal to (Minpartfreq) in at least one run. Minpartfreq is a parameter to        sumt command and currently it is set to 0.10.  This is followed by a table        with statistics for the informative bipartitions (those including at least          two taxa), sorted from highest to lowest probability. For each bipartition,         the table gives the number of times the partition or split was observed in all      runs (#obs) and the posterior probability of the bipartition (Probab.), which       is the same as the split frequency. If several runs are summarized, this is         followed by the minimum split frequency (Min(s)), the maximum frequency             (Max(s)), and the standard deviation of frequencies (Stddev(s)) across runs.        The latter value should approach 0 for all bipartitions as MCMC runs converge.                                                                                         This is followed by a table summarizing branch lengths, node heights (if a          clock model was used) and relaxed clock parameters (if a relaxed clock model        was used). The mean, variance, and 95 % credible interval are given for each       of these parameters. If several runs are summarized, the potential scale            reduction factor (PSRF) is also given; it should approach 1 as runs converge.       Node heights will take calibration points into account, if such points were         used in the analysis.                                                                                                                                                   Note that Stddev may be unreliable if the partition is not present in all           runs (the last column indicates the number of runs that sampled the partition       if more than one run is summarized). The PSRF is not calculated at all if           the partition is not present in all runs.The PSRF is also sensitive to small        sample sizes and it should only be considered a rough guide to convergence          since some of the assumptions allowing one to interpret it as a true potential      scale reduction factor are violated in MrBayes.                                                                                                                         List of taxa in bipartitions:                                                                                                                                             1 -- Euconodonta         2 -- Jamoytius         3 -- Euphanerops         4 -- Achanarella         5 -- Ciderius         6 -- Cornovichthys         7 -- Lasanius         8 -- Birkenia         9 -- Rhyncholepis        10 -- Myxinikela        11 -- Tethymyxine        12 -- Paramyxine_fernholmi        13 -- Eptatretus_burgeri        14 -- Eptatretus_stoutii        15 -- Myxine_glutinosa        16 -- Rubicundus_eos        17 -- Rubicundus_lopheliae        18 -- Neomyxine_biniplicata        19 -- Myxineidus        20 -- Gilpichthys        21 -- Lethenteron_camtschaticum        22 -- Petromyzon_marinus        23 -- Lampetra_fluviatilis        24 -- Geotria_australis        25 -- Ichthyomyzon_bdellium        26 -- Ichthyomyzon_castaneus        27 -- Ichthyomyzon_unicuspis        28 -- Mordacia_mordax        29 -- Mordacia_lapicida        30 -- Caspiomyzon_wagneri        31 -- Tetrapleurodon_spadiceus        32 -- Entosphenus_macrostomus        33 -- Entosphenus_minimus        34 -- Entosphenus_similis        35 -- Entosphenus_tridentatus        36 -- Eudontomyzon_danfordi        37 -- Eudontomyzon_morii        38 -- Lampetra_ayresii        39 -- Mesomyzon        40 -- Yanliaomyzon_ingensdentes        41 -- Yanliaomyzon_occisor        42 -- Priscomyzon        43 -- Mayomyzon        44 -- Hardistiella        45 -- Pipiscius       Key to taxon bipartitions (saved to file "run.te.maj.parts"):        ID -- Partition      ----------------------------------------------------        0 -- *********************************************        1 -- *............................................        2 -- .*...........................................        3 -- ..*..........................................        4 -- ...*.........................................        5 -- ....*........................................        6 -- .....*.......................................        7 -- ......*......................................        8 -- .......*.....................................        9 -- ........*....................................       10 -- .........*...................................       11 -- ..........*..................................       12 -- ...........*.................................       13 -- ............*................................       14 -- .............*...............................       15 -- ..............*..............................       16 -- ...............*.............................       17 -- ................*............................       18 -- .................*...........................       19 -- ..................*..........................       20 -- ...................*.........................       21 -- ....................*........................       22 -- .....................*.......................       23 -- ......................*......................       24 -- .......................*.....................       25 -- ........................*....................       26 -- .........................*...................       27 -- ..........................*..................       28 -- ...........................*.................       29 -- ............................*................       30 -- .............................*...............       31 -- ..............................*..............       32 -- ...............................*.............       33 -- ................................*............       34 -- .................................*...........       35 -- ..................................*..........       36 -- ...................................*.........       37 -- ....................................*........       38 -- .....................................*.......       39 -- ......................................*......       40 -- .......................................*.....       41 -- ........................................*....       42 -- .........................................*...       43 -- ..........................................*..       44 -- ...........................................*.       45 -- ............................................*       46 -- .********************************************       47 -- ..........********...........................       48 -- ...........***...............................       49 -- ....................***.***..*********.......       50 -- ....................******************.......       51 -- ...........................**................       52 -- ....................*********************....       53 -- ...............**............................       54 -- ..............*..*...........................       55 -- ......................*..............*.......       56 -- .......**....................................       57 -- ...................................**........       58 -- .........*********...........................       59 -- ........................***..................       60 -- .**..........................................       61 -- ...............................*..*..........       62 -- ....................*************************       63 -- ...........*******...........................       64 -- ...........****..*...........................       65 -- ....................*.*............***.......       66 -- .....................*..***..*...............       67 -- .....................*..***..................       68 -- ....................*.*.......********.......       69 -- ......***....................................       70 -- .**...***....................................       71 -- ....................***.**************.......       72 -- ...***...************************************       73 -- ...............................****..........       74 -- ...........**................................       75 -- ..............................*****..........       76 -- ....................******************.**....       77 -- ....................*********************..*.       78 -- ...**........................................       79 -- ...............................*.**..........       80 -- ....................*********************.***       81 -- ..................**.........................       82 -- ....................*..............**........       83 -- ....................******************..*....       84 -- ........................*.*..................       85 -- ........................**...................       86 -- .........************************************       87 -- ....................*********************.**.       88 -- .........***********.........................       89 -- .......................................**....       90 -- ..........................................*.*       91 -- ...........*.*...............................       92 -- ...***.......................................       93 -- ....................*.*..............*.......       94 -- ...*.*.......................................       95 -- ....................*******************......       96 -- .........*********.*.........................       97 -- ......................*............***.......       98 -- ................................**...........       99 -- .........................**..................      100 -- ....................************************.      101 -- ....................*******************.*....      102 -- ....................*.*.......*....***.......      103 -- ..........................................**.      104 -- ....................**********************...      105 -- .**...*......................................      106 -- .........*********.**************************      107 -- ..............................**..*..........      108 -- .....*...************************************      109 -- .......................*...**................      110 -- .........**********..........................      111 -- ......................................***....      112 -- ....................*********************.*..      113 -- ...............................**.*..........      114 -- .....*............*..........................      115 -- ....................*******..*********.......      116 -- ....................*.*.......*****..*.......      117 -- ....................*.*......*********.......      118 -- ..................***************************      119 -- .....................*.......*...............      120 -- ..........................................***      121 -- ...***............*..........................      122 -- ...................**************************      ----------------------------------------------------       Summary statistics for informative taxon bipartitions (clades)         (saved to file "run.te.maj.tstat"):        ID   #obs      Probab.     Sd(s)+      Min(s)      Max(s)   Nruns       -------------------------------------------------------------------       46  112502    1.000000    0.000000    1.000000    1.000000    2       47  112502    1.000000    0.000000    1.000000    1.000000    2       48  112502    1.000000    0.000000    1.000000    1.000000    2       49  112502    1.000000    0.000000    1.000000    1.000000    2       50  112502    1.000000    0.000000    1.000000    1.000000    2       51  112500    0.999982    0.000025    0.999964    1.000000    2       52  112491    0.999902    0.000038    0.999876    0.999929    2       53  112482    0.999822    0.000176    0.999698    0.999947    2       54  112468    0.999698    0.000226    0.999538    0.999858    2       55  112215    0.997449    0.000189    0.997316    0.997582    2       56  111277    0.989111    0.001622    0.987965    0.990258    2       57  110959    0.986285    0.004412    0.983165    0.989405    2       58  106872    0.949956    0.002791    0.947983    0.951930    2       59  105376    0.936659    0.002137    0.935148    0.938170    2       60  102522    0.911290    0.004576    0.908055    0.914526    2       61   99000    0.879984    0.000101    0.879913    0.880055    2       62   98763    0.877878    0.009038    0.871487    0.884269    2       63   98467    0.875247    0.013413    0.865762    0.884731    2       64   98332    0.874047    0.022376    0.858225    0.889869    2       65   94004    0.835576    0.049880    0.800306    0.870847    2       66   91306    0.811594    0.017750    0.799044    0.824145    2       67   90991    0.808795    0.008586    0.802724    0.814866    2       68   89108    0.792057    0.005154    0.788413    0.795701    2       69   84122    0.747738    0.003620    0.745178    0.750298    2       70   82474    0.733089    0.001760    0.731845    0.734334    2       71   81931    0.728263    0.022061    0.712663    0.743862    2       72   79547    0.707072    0.005267    0.703347    0.710796    2       73   78779    0.700245    0.000088    0.700183    0.700308    2       74   77926    0.692663    0.017222    0.680486    0.704841    2       75   68044    0.604825    0.030521    0.583243    0.626407    2       76   61760    0.548968    0.020716    0.534319    0.563617    2       77   60954    0.541804    0.010861    0.534124    0.549484    2       78   54218    0.481929    0.016669    0.470143    0.493716    2       79   51827    0.460676    0.004236    0.457681    0.463672    2       80   50580    0.449592    0.012897    0.440472    0.458712    2       81   47515    0.422348    0.000314    0.422126    0.422570    2       82   45872    0.407744    0.015135    0.397042    0.418446    2       83   45294    0.402606    0.023331    0.386109    0.419104    2       84   43843    0.389709    0.005971    0.385486    0.393931    2       85   42050    0.373771    0.002690    0.371869    0.375673    2       86   38384    0.341185    0.012646    0.332243    0.350127    2       87   37636    0.334536    0.008372    0.328616    0.340456    2       88   37618    0.334376    0.006084    0.330074    0.338678    2       89   33984    0.302075    0.010358    0.294750    0.309399    2       90   33969    0.301941    0.002250    0.300350    0.303532    2       91   33640    0.299017    0.016065    0.287657    0.310377    2       92   33301    0.296004    0.000163    0.295888    0.296119    2       93   31695    0.281728    0.007555    0.276386    0.287070    2       94   30790    0.273684    0.011087    0.265844    0.281524    2       95   29204    0.259586    0.020565    0.245045    0.274128    2       96   28048    0.249311    0.001483    0.248262    0.250360    2       97   28015    0.249018    0.015474    0.238076    0.259960    2       98   26066    0.231694    0.003671    0.229098    0.234289    2       99   24818    0.220601    0.001508    0.219534    0.221667    2      100   24200    0.215107    0.014054    0.205170    0.225045    2      101   22951    0.204005    0.019271    0.190379    0.217632    2      102   20921    0.185961    0.012508    0.177117    0.194805    2      103   18440    0.163908    0.003796    0.161224    0.166593    2      104   18185    0.161642    0.001647    0.160477    0.162806    2      105   17465    0.155242    0.004890    0.151784    0.158699    2      106   17455    0.155153    0.002778    0.153188    0.157117    2      107   17188    0.152780    0.001383    0.151802    0.153757    2      108   17086    0.151873    0.005682    0.147855    0.155891    2      109   16468    0.146380    0.013476    0.136851    0.155908    2      110   16451    0.146229    0.006474    0.141651    0.150806    2      111   16346    0.145295    0.001081    0.144531    0.146060    2      112   15720    0.139731    0.004324    0.136673    0.142789    2      113   15699    0.139544    0.002678    0.137651    0.141437    2      114   14788    0.131447    0.002665    0.129562    0.133331    2      115   14103    0.125358    0.008586    0.119287    0.131429    2      116   14035    0.124753    0.045996    0.092229    0.157277    2      117   13828    0.122913    0.012143    0.114327    0.131500    2      118   13614    0.121011    0.004953    0.117509    0.124513    2      119   13084    0.116300    0.005707    0.112265    0.120336    2      120   12794    0.113722    0.002715    0.111802    0.115642    2      121   11428    0.101580    0.000503    0.101225    0.101936    2      122   11311    0.100540    0.005493    0.096656    0.104425    2      -------------------------------------------------------------------      + Convergence diagnostic (standard deviation of split frequencies)        should approach 0.0 as runs converge.        Summary statistics for branch and node parameters         (saved to file "run.te.maj.vstat"):                                                               95% HPD Interval                                                            --------------------      Parameter                      Mean       Variance     Lower       Upper       Median     PSRF+  Nruns      ------------------------------------------------------------------------------------------------------      length{all}[1]                0.230486    0.018863    0.000053    0.494806    0.204519    1.001    2      length{all}[2]                0.021639    0.003064    0.000000    0.137200    0.000000    1.001    2      length{all}[3]                0.235312    0.006366    0.100831    0.411349    0.222566    1.000    2      length{all}[4]                0.141601    0.006660    0.000000    0.278699    0.137914    1.000    2      length{all}[5]                0.028165    0.004258    0.000000    0.167562    0.000000    1.001    2      length{all}[6]                0.153468    0.018030    0.000000    0.408986    0.125681    1.000    2      length{all}[7]                0.141924    0.005534    0.034793    0.304946    0.126915    1.001    2      length{all}[8]                0.001681    0.000058    0.000000    0.011928    0.000000    1.000    2      length{all}[9]                0.013404    0.000068    0.005767    0.027352    0.011741    1.000    2      length{all}[10]               0.024711    0.008808    0.000000    0.181001    0.000000    1.000    2      length{all}[11]               0.004187    0.000336    0.000000    0.029163    0.000000    1.000    2      length{all}[12]               0.037259    0.000200    0.013538    0.064844    0.035105    1.001    2      length{all}[13]               0.043191    0.000331    0.014417    0.079039    0.040061    1.000    2      length{all}[14]               0.049889    0.000386    0.016923    0.088731    0.047013    1.001    2      length{all}[15]               0.121058    0.002006    0.040360    0.207352    0.115047    1.006    2      length{all}[16]               0.021595    0.000493    0.000090    0.063238    0.014957    1.000    2      length{all}[17]               0.021594    0.000492    0.000090    0.063238    0.014957    1.000    2      length{all}[18]               0.121054    0.002005    0.040339    0.207326    0.115046    1.006    2      length{all}[19]               0.317576    0.037511    0.000000    0.681514    0.286313    1.000    2      length{all}[20]               0.301295    0.035719    0.000000    0.655154    0.265304    1.000    2      length{all}[21]               0.024824    0.000102    0.006660    0.044510    0.023606    1.000    2      length{all}[22]               0.029024    0.000294    0.003532    0.062361    0.025306    1.000    2      length{all}[23]               0.008792    0.000038    0.000500    0.020873    0.007380    1.000    2      length{all}[24]               0.257343    0.006015    0.118471    0.414142    0.247664    1.000    2      length{all}[25]               0.005769    0.000030    0.000049    0.016015    0.004220    1.000    2      length{all}[26]               0.007086    0.000042    0.000041    0.019510    0.005232    1.000    2      length{all}[27]               0.006979    0.000042    0.000051    0.019062    0.005155    1.000    2      length{all}[28]               0.019668    0.000256    0.000997    0.050804    0.015261    1.000    2      length{all}[29]               0.019668    0.000256    0.000997    0.050804    0.015261    1.000    2      length{all}[30]               0.051090    0.000690    0.009665    0.101761    0.046518    1.001    2      length{all}[31]               0.033700    0.000431    0.001841    0.072140    0.030554    1.001    2      length{all}[32]               0.004169    0.000027    0.000000    0.014007    0.002436    1.000    2      length{all}[33]               0.020980    0.000243    0.001438    0.051711    0.016784    1.000    2      length{all}[34]               0.015656    0.000153    0.000342    0.040249    0.012241    1.000    2      length{all}[35]               0.004389    0.000031    0.000000    0.014994    0.002507    1.000    2      length{all}[36]               0.009862    0.000090    0.000226    0.026673    0.007255    1.001    2      length{all}[37]               0.009906    0.000098    0.000247    0.026653    0.007251    1.001    2      length{all}[38]               0.008789    0.000037    0.000499    0.020863    0.007380    1.000    2      length{all}[39]               0.183696    0.011002    0.018662    0.388187    0.165254    1.001    2      length{all}[40]               0.000970    0.000023    0.000000    0.005597    0.000000    1.000    2      length{all}[41]               0.007669    0.000104    0.000000    0.024654    0.000000    1.000    2      length{all}[42]               0.060822    0.004823    0.000000    0.194534    0.044708    1.000    2      length{all}[43]               0.217969    0.012691    0.000000    0.421236    0.204980    1.000    2      length{all}[44]               0.060573    0.007865    0.000000    0.242202    0.000000    1.000    2      length{all}[45]               0.280772    0.019114    0.052135    0.549814    0.260572    1.000    2      length{all}[46]               0.146909    0.013414    0.000001    0.371546    0.121743    1.000    2      length{all}[47]               0.640641    0.031958    0.328426    0.999417    0.615416    1.001    2      length{all}[48]               0.142155    0.003126    0.043284    0.252753    0.135313    1.002    2      length{all}[49]               0.126608    0.003141    0.032235    0.236989    0.117899    1.001    2      length{all}[50]               0.287467    0.017735    0.050839    0.549342    0.269129    1.001    2      length{all}[51]               0.195727    0.004340    0.082948    0.329709    0.185710    1.000    2      length{all}[52]               0.504233    0.029803    0.187313    0.851047    0.483302    1.000    2      length{all}[53]               0.238390    0.004793    0.108113    0.376034    0.232180    1.000    2      length{all}[54]               0.078382    0.001693    0.015128    0.159337    0.069888    1.000    2      length{all}[55]               0.018625    0.000091    0.002618    0.037376    0.017194    1.000    2      length{all}[56]               0.131857    0.007432    0.013724    0.301356    0.111299    1.000    2      length{all}[57]               0.019114    0.000234    0.000708    0.048572    0.015163    1.003    2      length{all}[58]               0.421750    0.051988    0.049256    0.857707    0.389137    1.000    2      length{all}[59]               0.021568    0.000263    0.000001    0.052176    0.017678    1.000    2      length{all}[60]               0.194859    0.011133    0.020634    0.403654    0.177152    1.000    2      length{all}[61]               0.011845    0.000117    0.000001    0.032995    0.008721    1.000    2      length{all}[62]               0.212760    0.015334    0.022094    0.458252    0.189503    1.001    2      length{all}[63]               0.076985    0.003919    0.000003    0.198101    0.062515    1.000    2      length{all}[64]               0.075084    0.002550    0.000098    0.171582    0.064709    1.002    2      length{all}[65]               0.022634    0.000287    0.000534    0.056809    0.018134    1.000    2      length{all}[66]               0.036586    0.000467    0.000001    0.076707    0.033162    1.000    2      length{all}[67]               0.029877    0.000483    0.000007    0.071974    0.024787    1.001    2      length{all}[68]               0.033791    0.000418    0.000001    0.071835    0.030465    1.000    2      length{all}[69]               0.137969    0.009750    0.000035    0.325476    0.117416    1.000    2      length{all}[70]               0.192573    0.012863    0.000271    0.402830    0.174001    1.000    2      length{all}[71]               0.067104    0.002148    0.000020    0.155187    0.056760    1.000    2      length{all}[72]               0.211807    0.016921    0.000188    0.452692    0.190598    1.000    2      length{all}[73]               0.020146    0.000276    0.000004    0.052353    0.015807    1.001    2      length{all}[74]               0.018325    0.000170    0.000706    0.043602    0.015262    1.000    2      length{all}[75]               0.020334    0.000273    0.000001    0.052688    0.016218    1.000    2      length{all}[76]               0.104537    0.006939    0.000003    0.265140    0.084325    1.001    2      length{all}[77]               0.117813    0.006566    0.000007    0.273165    0.098964    1.000    2      length{all}[78]               0.146212    0.011093    0.000017    0.348065    0.123393    1.000    2      length{all}[79]               0.012211    0.000142    0.000000    0.035526    0.008684    1.001    2      length{all}[80]               0.076787    0.003064    0.000012    0.181160    0.066260    1.001    2      length{all}[81]               0.271408    0.032145    0.000014    0.598319    0.244466    1.000    2      length{all}[82]               0.009959    0.000062    0.000000    0.025230    0.008278    1.001    2      length{all}[83]               0.015499    0.000047    0.000003    0.025463    0.015435    1.000    2      length{all}[84]               0.005234    0.000033    0.000000    0.016384    0.003413    1.000    2      length{all}[85]               0.005209    0.000034    0.000000    0.016363    0.003364    1.000    2      length{all}[86]               0.191698    0.021849    0.000005    0.479554    0.156417    1.001    2      length{all}[87]               0.091050    0.004645    0.000003    0.217989    0.078958    1.000    2      length{all}[88]               0.216126    0.026068    0.000007    0.522047    0.184718    1.001    2      length{all}[89]               0.042272    0.001646    0.000001    0.118999    0.030718    1.000    2      length{all}[90]               0.095122    0.006474    0.000000    0.251541    0.075288    1.000    2      length{all}[91]               0.020050    0.000190    0.000492    0.046172    0.017030    1.000    2      length{all}[92]               0.146489    0.012445    0.000013    0.363341    0.122625    1.000    2      length{all}[93]               0.010502    0.000121    0.000001    0.032364    0.007019    1.000    2      length{all}[94]               0.108805    0.007256    0.000003    0.275254    0.088359    1.000    2      length{all}[95]               0.051758    0.001881    0.000002    0.128099    0.042571    1.001    2      length{all}[96]               0.188247    0.021672    0.000006    0.467383    0.154878    1.000    2      length{all}[97]               0.006665    0.000035    0.000000    0.018056    0.005112    1.001    2      length{all}[98]               0.008312    0.000081    0.000000    0.026239    0.005463    1.000    2      length{all}[99]               0.003894    0.000025    0.000000    0.012923    0.002278    1.000    2      length{all}[100]              0.138048    0.017542    0.000003    0.401283    0.096263    1.000    2      length{all}[101]              0.015280    0.000062    0.000042    0.025854    0.015479    1.000    2      length{all}[102]              0.013785    0.000170    0.000002    0.039790    0.010087    1.000    2      length{all}[103]              0.095933    0.006528    0.000004    0.252672    0.074512    1.000    2      length{all}[104]              0.099921    0.004686    0.000024    0.229096    0.086476    1.001    2      length{all}[105]              0.087046    0.005777    0.000008    0.235510    0.066878    1.000    2      length{all}[106]              0.183434    0.020264    0.000021    0.455227    0.150460    1.000    2      length{all}[107]              0.011710    0.000141    0.000002    0.035504    0.007918    1.000    2      length{all}[108]              0.121806    0.011822    0.000002    0.335149    0.092451    1.000    2      length{all}[109]              0.054691    0.002570    0.000021    0.155075    0.041022    1.003    2      length{all}[110]              0.201279    0.027340    0.000001    0.528046    0.162277    1.001    2      length{all}[111]              0.065315    0.003740    0.000004    0.188266    0.048225    1.000    2      length{all}[112]              0.110300    0.007551    0.000001    0.281515    0.094260    1.000    2      length{all}[113]              0.007452    0.000065    0.000000    0.023178    0.004936    1.003    2      length{all}[114]              0.172952    0.015617    0.000063    0.414799    0.144331    1.000    2      length{all}[115]              0.055180    0.002278    0.000022    0.146542    0.043063    1.000    2      length{all}[116]              0.019202    0.000224    0.000004    0.048160    0.015336    1.002    2      length{all}[117]              0.020094    0.000273    0.000000    0.050996    0.016308    1.003    2      length{all}[118]              0.119765    0.009336    0.000019    0.302303    0.096470    1.001    2      length{all}[119]              0.013151    0.000148    0.000001    0.037234    0.009710    1.000    2      length{all}[120]              0.081532    0.004962    0.000003    0.220322    0.063278    1.000    2      length{all}[121]              0.158974    0.013863    0.000020    0.385110    0.135209    1.001    2      length{all}[122]              0.122603    0.010322    0.000005    0.319794    0.098436    1.002    2      height{all}[0]                2.067647    0.249103    1.211980    3.060242    1.993437    1.001    2      height{all}[1]                1.837161    0.203031    1.069096    2.744634    1.772565    1.001    2      height{all}[2]                1.493744    0.134221    0.869252    2.231585    1.441222    1.001    2      height{all}[3]                1.269374    0.096927    0.738684    1.896386    1.224741    1.001    2      height{all}[4]                1.320835    0.104946    0.768631    1.973266    1.274393    1.001    2      height{all}[5]                1.482080    0.132133    0.862464    2.214159    1.429968    1.001    2      height{all}[6]                1.320835    0.104946    0.768631    1.973266    1.274393    1.001    2      height{all}[7]                1.469043    0.129818    0.854877    2.194682    1.417390    1.001    2      height{all}[8]                1.493744    0.134221    0.869252    2.231585    1.441223    1.001    2      height{all}[9]                1.481908    0.132102    0.862364    2.213902    1.429803    1.001    2      height{all}[10]               0.960607    0.055508    0.559004    1.435103    0.926831    1.001    2      height{all}[11]               0.325920    0.006390    0.189662    0.486910    0.314461    1.001    2      height{all}[12]               0.000000    0.000000    0.000000    0.000000    0.000000    1.000    2      height{all}[13]               0.000000    0.000000    0.000000    0.000000    0.000000    1.000    2      height{all}[14]               0.000000    0.000000    0.000000    0.000000    0.000000    1.000    2      height{all}[15]               0.000000    0.000000    0.000000    0.000000    0.000000    1.000    2      height{all}[16]               0.000000    0.000000    0.000000    0.000000    0.000000    1.000    2      height{all}[17]               0.000000    0.000000    0.000000    0.000000    0.000000    1.000    2      height{all}[18]               0.000000    0.000000    0.000000    0.000000    0.000000    1.000    2      height{all}[19]               0.960607    0.055508    0.559004    1.435103    0.926831    1.001    2      height{all}[20]               0.960607    0.055508    0.559004    1.435103    0.926831    1.001    2      height{all}[21]               0.000000    0.000000    0.000000    0.000000    0.000000    1.000    2      height{all}[22]               0.000000    0.000000    0.000000    0.000000    0.000000    1.000    2      height{all}[23]               0.000000    0.000000    0.000000    0.000000    0.000000    1.000    2      height{all}[24]               0.000000    0.000000    0.000000    0.000000    0.000000    1.000    2      height{all}[25]               0.000000    0.000000    0.000000    0.000000    0.000000    1.000    2      height{all}[26]               0.000000    0.000000    0.000000    0.000000    0.000000    1.000    2      height{all}[27]               0.000000    0.000000    0.000000    0.000000    0.000000    1.000    2      height{all}[28]               0.000000    0.000000    0.000000    0.000000    0.000000    1.000    2      height{all}[29]               0.000000    0.000000    0.000000    0.000000    0.000000    1.000    2      height{all}[30]               0.000000    0.000000    0.000000    0.000000    0.000000    1.000    2      height{all}[31]               0.000000    0.000000    0.000000    0.000000    0.000000    1.000    2      height{all}[32]               0.000000    0.000000    0.000000    0.000000    0.000000    1.000    2      height{all}[33]               0.000000    0.000000    0.000000    0.000000    0.000000    1.000    2      height{all}[34]               0.000000    0.000000    0.000000    0.000000    0.000000    1.000    2      height{all}[35]               0.000000    0.000000    0.000000    0.000000    0.000000    1.000    2      height{all}[36]               0.000000    0.000000    0.000000    0.000000    0.000000    1.000    2      height{all}[37]               0.000000    0.000000    0.000000    0.000000    0.000000    1.000    2      height{all}[38]               0.000000    0.000000    0.000000    0.000000    0.000000    1.000    2      height{all}[39]               0.428843    0.011063    0.249556    0.640671    0.413764    1.001    2      height{all}[40]               0.559211    0.018811    0.325420    0.835435    0.539548    1.001    2      height{all}[41]               0.542057    0.017675    0.315438    0.809808    0.522998    1.001    2      height{all}[42]               1.235066    0.091759    0.718720    1.845132    1.191640    1.001    2      height{all}[43]               0.960607    0.055508    0.559004    1.435103    0.926831    1.001    2      height{all}[44]               1.097837    0.072501    0.638862    1.640117    1.059236    1.001    2      height{all}[45]               0.960607    0.055508    0.559004    1.435103    0.926831    1.001    2      height{all}[46]               1.920738    0.209061    1.140637    2.837493    1.852664    1.001    2      height{all}[47]               0.334992    0.006539    0.196325    0.496163    0.322477    1.000    2      height{all}[48]               0.055947    0.000365    0.024350    0.093151    0.052659    1.000    2      height{all}[49]               0.089872    0.000605    0.048180    0.138762    0.086145    1.000    2      height{all}[50]               0.272266    0.005601    0.142820    0.420922    0.261035    1.000    2      height{all}[51]               0.019665    0.000256    0.000993    0.050795    0.015261    1.000    2      height{all}[52]               0.640152    0.028388    0.351028    0.974219    0.614521    1.001    2      height{all}[53]               0.021551    0.000481    0.000090    0.063147    0.014954    1.000    2      height{all}[54]               0.121034    0.002004    0.040457    0.207368    0.115026    1.006    2      height{all}[55]               0.008748    0.000037    0.000336    0.020541    0.007364    1.000    2      height{all}[56]               1.495604    0.134411    0.868703    2.231509    1.443163    1.001    2      height{all}[57]               0.009307    0.000064    0.000326    0.024194    0.007161    1.001    2      height{all}[58]               0.967045    0.058134    0.555236    1.450385    0.931630    1.001    2      height{all}[59]               0.009337    0.000045    0.000708    0.022336    0.007694    1.000    2      height{all}[60]               1.504250    0.136927    0.871995    2.245303    1.450864    1.000    2      height{all}[61]               0.003410    0.000016    0.000000    0.011211    0.002084    1.000    2      height{all}[62]               1.333945    0.100569    0.783646    1.971782    1.289476    1.001    2      height{all}[63]               0.259724    0.004094    0.146085    0.387200    0.252314    1.000    2      height{all}[64]               0.193514    0.002796    0.099341    0.297257    0.187183    1.002    2      height{all}[65]               0.030680    0.000112    0.013333    0.051873    0.028830    1.000    2      height{all}[66]               0.047716    0.000396    0.013860    0.086282    0.044517    1.000    2      height{all}[67]               0.027663    0.000227    0.005008    0.057057    0.024484    1.000    2      height{all}[68]               0.052729    0.000304    0.023209    0.086676    0.050076    1.000    2      height{all}[69]               1.606436    0.152891    0.925195    2.379345    1.550473    1.000    2      height{all}[70]               1.711434    0.168929    1.007679    2.532307    1.654697    1.000    2      height{all}[71]               0.207386    0.003313    0.108239    0.321128    0.199409    1.000    2      height{all}[72]               1.709715    0.161706    1.021285    2.523892    1.652349    1.000    2      height{all}[73]               0.021225    0.000142    0.003453    0.044600    0.018581    1.001    2      height{all}[74]               0.037341    0.000198    0.013760    0.064852    0.035245    1.001    2      height{all}[75]               0.030749    0.000227    0.007497    0.060749    0.027948    1.001    2      height{all}[76]               0.564510    0.019578    0.326439    0.844504    0.544621    1.001    2      height{all}[77]               1.109695    0.073325    0.640670    1.649326    1.069772    1.001    2      height{all}[78]               1.472890    0.129853    0.859251    2.206461    1.423369    1.001    2      height{all}[79]               0.011733    0.000070    0.000832    0.028202    0.009582    1.000    2      height{all}[80]               1.249281    0.087921    0.725604    1.835406    1.209952    1.002    2      height{all}[81]               1.185817    0.094047    0.671138    1.802848    1.138931    1.001    2      height{all}[82]               0.019984    0.000074    0.005626    0.037325    0.018731    1.000    2      height{all}[83]               0.540127    0.017211    0.310304    0.795376    0.521927    1.000    2      height{all}[84]               0.004614    0.000017    0.000049    0.012609    0.003427    1.000    2      height{all}[85]               0.004590    0.000017    0.000041    0.012488    0.003440    1.000    2      height{all}[86]               1.577074    0.141083    0.918887    2.326455    1.517784    1.001    2      height{all}[87]               1.183113    0.080605    0.690903    1.737426    1.148352    1.001    2      height{all}[88]               1.347093    0.113407    0.752726    2.016233    1.300401    1.000    2      height{all}[89]               0.553874    0.019548    0.316608    0.838999    0.531865    1.003    2      height{all}[90]               1.169022    0.081036    0.680883    1.743652    1.129860    1.001    2      height{all}[91]               0.036725    0.000198    0.013541    0.064491    0.034508    1.001    2      height{all}[92]               1.535234    0.144365    0.899130    2.320638    1.478029    1.000    2      height{all}[93]               0.026404    0.000084    0.010956    0.044551    0.025031    1.001    2      height{all}[94]               1.446968    0.129845    0.830606    2.152933    1.388987    1.000    2      height{all}[95]               0.529205    0.017412    0.308802    0.794904    0.509658    1.000    2      height{all}[96]               1.244602    0.094775    0.719053    1.872767    1.203116    1.002    2      height{all}[97]               0.022747    0.000073    0.007947    0.039444    0.021445    1.000    2      height{all}[98]               0.014226    0.000093    0.001298    0.033119    0.011863    1.000    2      height{all}[99]               0.005466    0.000021    0.000129    0.013888    0.004288    1.000    2      height{all}[100]              1.293207    0.097594    0.770118    1.921097    1.248308    1.001    2      height{all}[101]              0.564897    0.017799    0.341148    0.831237    0.547581    1.000    2      height{all}[102]              0.043388    0.000240    0.018765    0.074859    0.040588    1.000    2      height{all}[103]              1.170966    0.085897    0.664963    1.765895    1.129973    1.000    2      height{all}[104]              1.226332    0.094392    0.707767    1.847856    1.179862    1.000    2      height{all}[105]              1.612536    0.152468    0.952070    2.412199    1.554730    1.002    2      height{all}[106]              1.507535    0.115328    0.949531    2.206175    1.458036    1.001    2      height{all}[107]              0.014285    0.000097    0.001523    0.034065    0.011813    1.000    2      height{all}[108]              1.634475    0.145169    0.959017    2.384571    1.583374    1.001    2      height{all}[109]              0.198615    0.004363    0.087476    0.326033    0.189825    1.000    2      height{all}[110]              1.225049    0.096005    0.686313    1.842752    1.182189    1.000    2      height{all}[111]              0.571302    0.021348    0.326195    0.875531    0.549382    1.001    2      height{all}[112]              1.083119    0.075254    0.610159    1.614037    1.048520    1.002    2      height{all}[113]              0.014012    0.000087    0.001762    0.032562    0.011656    1.001    2      height{all}[114]              1.331125    0.106651    0.764886    1.963170    1.286005    1.001    2      height{all}[115]              0.226307    0.003487    0.126854    0.342821    0.218411    1.000    2      height{all}[116]              0.039706    0.000197    0.016765    0.068063    0.037210    1.001    2      height{all}[117]              0.069314    0.000426    0.035036    0.111559    0.066472    1.000    2      height{all}[118]              1.477764    0.133686    0.856488    2.208800    1.418396    1.000    2      height{all}[119]              0.029454    0.000226    0.006592    0.059267    0.026524    1.001    2      height{all}[120]              1.249767    0.094426    0.724987    1.862445    1.205443    1.000    2      height{all}[121]              1.498692    0.121085    0.894958    2.194312    1.454902    1.000    2      height{all}[122]              1.430528    0.117742    0.827328    2.113040    1.389040    1.001    2      age{all}[0]                 604.554734  1574.010251  535.518584  679.295816  598.151577    1.000    2      age{all}[1]                 535.500019    0.000000  535.499920  535.500121  535.500019    1.000    2      age{all}[2]                 435.400019    0.000000  435.399932  435.400106  435.400018    1.000    2      age{all}[3]                 370.000019    0.000000  369.999941  370.000101  370.000018    1.000    2      age{all}[4]                 385.000019    0.000000  384.999941  385.000098  385.000018    1.000    2      age{all}[5]                 432.000019    0.000000  431.999938  432.000104  432.000018    1.000    2      age{all}[6]                 385.000019    0.000000  384.999943  385.000099  385.000018    1.000    2      age{all}[7]                 428.200019    0.000000  428.199933  428.200106  428.200018    1.000    2      age{all}[8]                 435.400019    0.000000  435.399933  435.400107  435.400019    1.000    2      age{all}[9]                 431.950019    0.000000  431.949933  431.950106  431.950019    1.000    2      age{all}[10]                280.000019    0.000000  279.999960  280.000078  280.000018    1.000    2      age{all}[11]                 95.000019    0.000000   94.999985   95.000059   95.000016    1.000    2      age{all}[12]                  0.000019    0.000000    0.000000    0.000056    0.000014    1.000    2      age{all}[13]                  0.000019    0.000000    0.000000    0.000056    0.000014    1.000    2      age{all}[14]                  0.000019    0.000000    0.000000    0.000056    0.000014    1.000    2      age{all}[15]                  0.000019    0.000000    0.000000    0.000056    0.000014    1.000    2      age{all}[16]                  0.000019    0.000000    0.000000    0.000056    0.000014    1.000    2      age{all}[17]                  0.000019    0.000000    0.000000    0.000056    0.000014    1.000    2      age{all}[18]                  0.000019    0.000000    0.000000    0.000056    0.000014    1.000    2      age{all}[19]                280.000019    0.000000  279.999956  280.000086  280.000018    1.000    2      age{all}[20]                280.000019    0.000000  279.999957  280.000083  280.000018    1.000    2      age{all}[21]                  0.000019    0.000000    0.000000    0.000055    0.000014    1.000    2      age{all}[22]                  0.000019    0.000000    0.000000    0.000055    0.000014    1.000    2      age{all}[23]                  0.000019    0.000000    0.000000    0.000055    0.000014    1.000    2      age{all}[24]                  0.000019    0.000000    0.000000    0.000056    0.000015    1.000    2      age{all}[25]                  0.000019    0.000000    0.000000    0.000055    0.000014    1.000    2      age{all}[26]                  0.000019    0.000000    0.000000    0.000055    0.000014    1.000    2      age{all}[27]                  0.000019    0.000000    0.000000    0.000055    0.000014    1.000    2      age{all}[28]                  0.000019    0.000000    0.000000    0.000056    0.000014    1.000    2      age{all}[29]                  0.000019    0.000000    0.000000    0.000056    0.000014    1.000    2      age{all}[30]                  0.000019    0.000000    0.000000    0.000055    0.000014    1.000    2      age{all}[31]                  0.000019    0.000000    0.000000    0.000055    0.000014    1.000    2      age{all}[32]                  0.000019    0.000000    0.000000    0.000055    0.000014    1.000    2      age{all}[33]                  0.000019    0.000000    0.000000    0.000055    0.000014    1.000    2      age{all}[34]                  0.000019    0.000000    0.000000    0.000055    0.000014    1.000    2      age{all}[35]                  0.000019    0.000000    0.000000    0.000055    0.000014    1.000    2      age{all}[36]                  0.000019    0.000000    0.000000    0.000055    0.000014    1.000    2      age{all}[37]                  0.000019    0.000000    0.000000    0.000055    0.000014    1.000    2      age{all}[38]                  0.000019    0.000000    0.000000    0.000055    0.000014    1.000    2      age{all}[39]                125.000019    0.000000  124.999978  125.000064  125.000017    1.000    2      age{all}[40]                163.000019    0.000000  162.999977  163.000064  163.000017    1.000    2      age{all}[41]                158.000019    0.000000  157.999978  158.000064  158.000017    1.000    2      age{all}[42]                360.000019    0.000000  359.999949  360.000090  360.000018    1.000    2      age{all}[43]                280.000019    0.000000  279.999957  280.000082  280.000018    1.000    2      age{all}[44]                320.000019    0.000000  319.999954  320.000085  320.000018    1.000    2      age{all}[45]                280.000019    0.000000  279.999957  280.000082  280.000018    1.000    2      age{all}[46]                562.129546  1497.309170  492.206846  640.579377  558.739487    1.000    2      age{all}[47]                 98.099826   79.776720   94.999968  117.028703   95.000021    1.000    2      age{all}[48]                 16.870624   34.422170    6.261741   28.364374   15.981050    1.000    2      age{all}[49]                 27.093616   59.566340   13.082519   42.170121   26.195560    1.001    2      age{all}[50]                 81.784576  480.383218   40.399691  125.000154   79.797791    1.001    2      age{all}[51]                  5.891142   22.605126    0.293238   15.087555    4.594848    1.000    2      age{all}[52]                187.339603  601.144474  162.999951  235.875318  181.570818    1.000    2      age{all}[53]                  6.475560   43.200072    0.027378   19.077766    4.475624    1.000    2      age{all}[54]                 36.737361  217.081566   10.988779   64.886043   34.787472    1.008    2      age{all}[55]                  2.639908    3.426662    0.137613    6.294682    2.194240    1.000    2      age{all}[56]                435.915016    5.336126  435.399842  439.113384  435.400025    1.000    2      age{all}[57]                  2.801477    5.777826    0.081125    7.349390    2.156220    1.000    2      age{all}[58]                282.509538  128.845904  279.999899  296.645186  280.000022    1.000    2      age{all}[59]                  2.814091    4.098084    0.193237    6.708102    2.319123    1.000    2      age{all}[60]                439.669676  136.552972  435.399843  464.305699  435.400037    1.000    2      age{all}[61]                  1.024821    1.473342    0.000013    3.360505    0.624808    1.000    2      age{all}[62]                391.885067  674.844569  359.999899  438.699557  389.735421    1.000    2      age{all}[63]                 75.362903  196.232657   47.629831   94.998715   77.007334    1.000    2      age{all}[64]                 58.299965  255.721750   28.257368   87.798191   57.089966    1.005    2      age{all}[65]                  9.274016   11.476617    3.498308   16.001459    8.770730    1.001    2      age{all}[66]                 14.413466   37.949481    4.268712   26.752965   13.414509    1.001    2      age{all}[67]                  8.338085   21.113772    1.577856   17.570094    7.355174    1.001    2      age{all}[68]                 15.946729   29.598129    6.474026   26.874948   15.194363    1.000    2      age{all}[69]                468.051129  328.066191  439.867286  503.470242  464.531386    1.000    2      age{all}[70]                502.796193  710.096090  458.391876  556.668985  498.867945    1.000    2      age{all}[71]                 61.958634  324.928510   29.758899   97.894830   59.955198    1.001    2      age{all}[72]                496.946456  1265.415603  431.999912  561.015123  493.313944    1.000    2      age{all}[73]                  6.388012   12.822867    1.131791   13.468729    5.600384    1.002    2      age{all}[74]                 11.209423   19.199475    3.621648   19.718835   10.597777    1.001    2      age{all}[75]                  9.309948   20.862851    2.114745   18.389836    8.437868    1.004    2      age{all}[76]                166.407971   57.607815  162.999942  181.953401  163.000034    1.000    2      age{all}[77]                325.525955  173.082335  319.999899  355.347604  320.000034    1.000    2      age{all}[78]                435.561987  102.098042  431.999836  455.398571  432.000037    1.000    2      age{all}[79]                  3.531302    6.296345    0.267151    8.495203    2.901995    1.000    2      age{all}[80]                358.169466  519.181411  319.999939  400.356533  353.062008    1.000    2      age{all}[81]                343.330746  1307.860901  287.866467  414.082742  336.215152    1.000    2      age{all}[82]                  6.056234    7.386644    1.389161   11.275712    5.662378    1.001    2      age{all}[83]                158.549834    1.904109  157.999949  161.902893  158.000025    1.001    2      age{all}[84]                  1.391910    1.569330    0.022635    3.853313    1.035559    1.000    2      age{all}[85]                  1.387053    1.524392    0.010366    3.810573    1.027179    1.000    2      age{all}[86]                450.603866  1450.234020  384.384686  527.053711  445.757407    1.001    2      age{all}[87]                347.591152  453.023800  319.999924  386.928880  343.916680    1.000    2      age{all}[88]                388.528839  1662.936898  315.122162  467.965323  384.966440    1.000    2      age{all}[89]                163.378239    2.273445  162.999943  165.603570  163.000022    1.000    2      age{all}[90]                337.058185  636.223564  294.285021  387.210898  333.103956    1.001    2      age{all}[91]                 11.298358   19.903088    3.781328   20.345395   10.619397    1.000    2      age{all}[92]                449.987060  576.259019  431.999876  499.663568  439.486499    1.000    2      age{all}[93]                  7.923463    8.709261    2.740384   13.713238    7.507329    1.000    2      age{all}[94]                415.548449  284.724463  389.377943  448.486893  412.907449    1.000    2      age{all}[95]                150.468276  149.881983  128.406130  176.330490  149.537819    1.000    2      age{all}[96]                366.850069  1730.920433  298.911060  449.051258  361.009486    1.000    2      age{all}[97]                  6.926258    7.503181    2.308021   12.307272    6.560631    1.000    2      age{all}[98]                  4.271132    8.324667    0.370076    9.959156    3.569857    1.000    2      age{all}[99]                  1.645173    1.868878    0.038319    4.242647    1.282244    1.000    2      age{all}[100]               382.693060  447.338027  359.999902  421.316886  380.520419    1.000    2      age{all}[101]               158.979455    3.050646  157.999953  162.555543  158.000033    1.000    2      age{all}[102]                12.984508   23.806585    4.673624   22.485926   12.206369    1.000    2      age{all}[103]               344.021236  519.129546  319.999886  386.163443  340.736492    1.000    2      age{all}[104]               365.473904  140.997193  359.999909  392.384246  360.000040    1.000    2      age{all}[105]               474.933526  376.709315  442.984555  512.485291  471.516336    1.000    2      age{all}[106]               445.819802  1368.193259  381.821753  519.828866  441.271341    1.000    2      age{all}[107]                 4.285229    8.869101    0.388405   10.317888    3.535742    1.000    2      age{all}[108]               462.397292  1230.403469  401.329790  537.276208  458.272457    1.001    2      age{all}[109]                61.572351  440.491070   23.823075  102.148423   59.860511    1.001    2      age{all}[110]               357.200673  1480.344968  294.003713  432.884444  350.836931    1.001    2      age{all}[111]               169.529187  107.605439  162.999941  190.674749  163.825662    1.001    2      age{all}[112]               310.986193  697.312765  279.999929  361.145141  308.628233    1.000    2      age{all}[113]                 4.215095    7.942479    0.537076    9.701616    3.525199    1.002    2      age{all}[114]               400.705668  716.589695  384.999892  458.288847  385.000062    1.000    2      age{all}[115]                70.052551  332.776446   35.880576  106.516051   68.401588    1.007    2      age{all}[116]                11.967403   18.226098    4.980394   20.709852   11.155249    1.000    2      age{all}[117]                20.855627   40.129303    9.869689   33.741087   20.113851    1.002    2      age{all}[118]               441.151307  1155.843837  380.075766  506.187435  437.113845    1.000    2      age{all}[119]                 8.871039   20.824720    1.942701   18.044664    7.942157    1.000    2      age{all}[120]               365.487789  598.669000  319.999910  407.496982  364.929559    1.000    2      age{all}[121]               459.409756  817.178756  431.999883  514.743750  452.202704    1.000    2      age{all}[122]               434.948938  1224.908809  359.999922  497.375239  431.931747    1.000    2      IlnBrlens{1}_length[1]        0.510649    0.340648    0.000001    1.533807    0.344374    1.000    2      IlnBrlens{1}_length[2]        0.010501    0.001214    0.000000    0.062500    0.000000    1.000    2      IlnBrlens{1}_length[3]        0.151378    0.015436    0.007516    0.386985    0.118838    1.000    2      IlnBrlens{1}_length[4]        0.126149    0.017121    0.000000    0.372082    0.089120    1.000    2      IlnBrlens{1}_length[5]        0.014028    0.001698    0.000000    0.081123    0.000000    1.001    2      IlnBrlens{1}_length[6]        0.124343    0.027725    0.000000    0.435452    0.069164    1.000    2      IlnBrlens{1}_length[7]        0.060876    0.003749    0.000000    0.175741    0.042742    1.000    2      IlnBrlens{1}_length[8]        0.001021    0.000025    0.000000    0.006469    0.000000    1.000    2      IlnBrlens{1}_length[9]        0.010577    0.000091    0.000000    0.028029    0.007999    1.000    2      IlnBrlens{1}_length[10]       0.038616    0.046317    0.000000    0.196749    0.000000    1.000    2      IlnBrlens{1}_length[11]       0.003373    0.000332    0.000000    0.016873    0.000000    1.000    2      IlnBrlens{1}_length[12]       0.023081    0.000426    0.000459    0.061026    0.017380    1.000    2      IlnBrlens{1}_length[13]       0.018422    0.000322    0.000283    0.052620    0.013095    1.000    2      IlnBrlens{1}_length[14]       0.025468    0.000518    0.000435    0.068510    0.019209    1.000    2      IlnBrlens{1}_length[15]       0.049502    0.001651    0.001832    0.127895    0.038660    1.001    2      IlnBrlens{1}_length[16]       0.013230    0.000276    0.000003    0.043768    0.007827    1.000    2      IlnBrlens{1}_length[17]       0.014702    0.000315    0.000001    0.048065    0.008993    1.000    2      IlnBrlens{1}_length[18]       0.045183    0.001481    0.000648    0.119398    0.034557    1.001    2      IlnBrlens{1}_length[19]       0.428674    0.220172    0.000000    1.230583    0.296018    1.000    2      IlnBrlens{1}_length[20]       0.240126    0.073442    0.000000    0.732527    0.159342    1.000    2      IlnBrlens{1}_length[21]       0.015089    0.000176    0.000148    0.040588    0.011433    1.000    2      IlnBrlens{1}_length[22]       0.025362    0.000418    0.000564    0.064749    0.020008    1.000    2      IlnBrlens{1}_length[23]       0.007937    0.000065    0.000042    0.022961    0.005502    1.000    2      IlnBrlens{1}_length[24]       0.129802    0.009358    0.002616    0.316110    0.107087    1.000    2      IlnBrlens{1}_length[25]       0.003415    0.000020    0.000003    0.011648    0.001921    1.000    2      IlnBrlens{1}_length[26]       0.004060    0.000029    0.000002    0.013743    0.002283    1.000    2      IlnBrlens{1}_length[27]       0.003915    0.000027    0.000001    0.013206    0.002244    1.000    2      IlnBrlens{1}_length[28]       0.021054    0.000502    0.000033    0.063710    0.014237    1.000    2      IlnBrlens{1}_length[29]       0.015804    0.000325    0.000013    0.048880    0.010254    1.000    2      IlnBrlens{1}_length[30]       0.019020    0.000320    0.000189    0.053571    0.013796    1.000    2      IlnBrlens{1}_length[31]       0.013253    0.000188    0.000112    0.038387    0.009149    1.000    2      IlnBrlens{1}_length[32]       0.002847    0.000024    0.000000    0.011072    0.001197    1.000    2      IlnBrlens{1}_length[33]       0.010897    0.000128    0.000062    0.032330    0.007390    1.000    2      IlnBrlens{1}_length[34]       0.014326    0.000242    0.000017    0.043822    0.009412    1.000    2      IlnBrlens{1}_length[35]       0.002785    0.000021    0.000000    0.010701    0.001213    1.000    2      IlnBrlens{1}_length[36]       0.006101    0.000058    0.000015    0.019992    0.003632    1.000    2      IlnBrlens{1}_length[37]       0.010115    0.000152    0.000023    0.032692    0.006202    1.001    2      IlnBrlens{1}_length[38]       0.005304    0.000039    0.000016    0.017142    0.003261    1.000    2      IlnBrlens{1}_length[39]       0.193159    0.027752    0.000000    0.501658    0.149050    1.000    2      IlnBrlens{1}_length[40]       0.000476    0.000008    0.000000    0.002050    0.000000    1.000    2      IlnBrlens{1}_length[41]       0.004096    0.000067    0.000000    0.018937    0.000000    1.000    2      IlnBrlens{1}_length[42]       0.097074    0.022015    0.000000    0.372678    0.044612    1.000    2      IlnBrlens{1}_length[43]       0.188840    0.031789    0.000000    0.519633    0.140001    1.000    2      IlnBrlens{1}_length[44]       0.093402    0.029952    0.000000    0.425396    0.000000    1.000    2      IlnBrlens{1}_length[45]       0.144147    0.022421    0.000000    0.413490    0.099918    1.000    2      IlnBrlens{1}_length[46]       0.289175    0.192455    0.000001    1.095797    0.122751    1.000    2      IlnBrlens{1}_length[47]       3.796292   25.793971    0.119532   11.701510    2.330797    1.000    2      IlnBrlens{1}_length[48]       0.052048    0.002042    0.000639    0.141184    0.039424    1.000    2      IlnBrlens{1}_length[49]       0.082514    0.004555    0.001059    0.211466    0.064971    1.000    2      IlnBrlens{1}_length[50]       0.383327    0.092785    0.009767    0.943383    0.309774    1.000    2      IlnBrlens{1}_length[51]       0.121654    0.007421    0.004931    0.288042    0.102010    1.000    2      IlnBrlens{1}_length[52]       0.641594    0.274428    0.030998    1.621466    0.500223    1.000    2      IlnBrlens{1}_length[53]       0.082865    0.004368    0.001621    0.210833    0.065067    1.000    2      IlnBrlens{1}_length[54]       0.067417    0.003493    0.000964    0.181505    0.050652    1.000    2      IlnBrlens{1}_length[55]       0.008132    0.000069    0.000049    0.023301    0.005689    1.000    2      IlnBrlens{1}_length[56]       0.184070    0.026941    0.000886    0.487515    0.138589    1.000    2      IlnBrlens{1}_length[57]       0.008428    0.000090    0.000023    0.025776    0.005528    1.001    2      IlnBrlens{1}_length[58]       3.474262   36.305742    0.000051   12.269140    1.654862    1.000    2      IlnBrlens{1}_length[59]       0.022565    0.000460    0.000000    0.063579    0.016540    1.000    2      IlnBrlens{1}_length[60]       0.122548    0.016939    0.000011    0.361255    0.083640    1.000    2      IlnBrlens{1}_length[61]       0.007337    0.000077    0.000001    0.024050    0.004434    1.000    2      IlnBrlens{1}_length[62]       0.247901    0.122252    0.000006    0.882977    0.128165    1.000    2      IlnBrlens{1}_length[63]       0.044113    0.003175    0.000000    0.149453    0.024970    1.000    2      IlnBrlens{1}_length[64]       0.056773    0.002941    0.000001    0.158711    0.041717    1.000    2      IlnBrlens{1}_length[65]       0.012294    0.000161    0.000001    0.036552    0.008445    1.000    2      IlnBrlens{1}_length[66]       0.013963    0.000209    0.000000    0.041296    0.009554    1.000    2      IlnBrlens{1}_length[67]       0.020143    0.000405    0.000000    0.058450    0.014302    1.000    2      IlnBrlens{1}_length[68]       0.013940    0.000213    0.000000    0.041618    0.009453    1.000    2      IlnBrlens{1}_length[69]       0.093053    0.013064    0.000005    0.303542    0.056286    1.000    2      IlnBrlens{1}_length[70]       0.196363    0.056975    0.000002    0.632666    0.119056    1.000    2      IlnBrlens{1}_length[71]       0.041614    0.002187    0.000005    0.129742    0.026815    1.000    2      IlnBrlens{1}_length[72]       0.236652    0.071758    0.000004    0.696759    0.160358    1.000    2      IlnBrlens{1}_length[73]       0.009325    0.000109    0.000000    0.028987    0.006005    1.000    2      IlnBrlens{1}_length[74]       0.011617    0.000189    0.000001    0.037332    0.007231    1.000    2      IlnBrlens{1}_length[75]       0.008736    0.000098    0.000000    0.027625    0.005506    1.000    2      IlnBrlens{1}_length[76]       0.072940    0.009958    0.000001    0.247920    0.040819    1.000    2      IlnBrlens{1}_length[77]       0.304059    0.107148    0.000001    0.894780    0.210445    1.000    2      IlnBrlens{1}_length[78]       0.121354    0.022489    0.000009    0.390916    0.073889    1.000    2      IlnBrlens{1}_length[79]       0.008891    0.000098    0.000000    0.027579    0.005813    1.000    2      IlnBrlens{1}_length[80]       0.112571    0.035243    0.000002    0.401138    0.057206    1.000    2      IlnBrlens{1}_length[81]       0.225322    0.093578    0.000002    0.743553    0.131914    1.000    2      IlnBrlens{1}_length[82]       0.007085    0.000085    0.000000    0.024292    0.003970    1.000    2      IlnBrlens{1}_length[83]       0.010798    0.000169    0.000000    0.034422    0.006797    1.000    2      IlnBrlens{1}_length[84]       0.003125    0.000022    0.000000    0.011407    0.001515    1.000    2      IlnBrlens{1}_length[85]       0.003201    0.000024    0.000000    0.011837    0.001521    1.000    2      IlnBrlens{1}_length[86]       0.225882    0.076458    0.000001    0.735839    0.137798    1.000    2      IlnBrlens{1}_length[87]       0.177278    0.083056    0.000001    0.696502    0.072009    1.000    2      IlnBrlens{1}_length[88]       0.369193    0.938419    0.000002    1.379717    0.122916    1.001    2      IlnBrlens{1}_length[89]       0.034062    0.003153    0.000000    0.125273    0.016053    1.000    2      IlnBrlens{1}_length[90]       0.064570    0.007714    0.000000    0.221225    0.035415    1.000    2      IlnBrlens{1}_length[91]       0.011181    0.000176    0.000001    0.035570    0.006868    1.000    2      IlnBrlens{1}_length[92]       0.131076    0.029926    0.000007    0.447697    0.072772    1.000    2      IlnBrlens{1}_length[93]       0.005291    0.000052    0.000000    0.018757    0.002825    1.000    2      IlnBrlens{1}_length[94]       0.080704    0.011981    0.000001    0.270597    0.045923    1.000    2      IlnBrlens{1}_length[95]       0.054325    0.006103    0.000000    0.196859    0.027295    1.000    2      IlnBrlens{1}_length[96]       0.240240    0.376903    0.000004    0.868324    0.086752    1.000    2      IlnBrlens{1}_length[97]       0.003753    0.000025    0.000000    0.013085    0.002060    1.000    2      IlnBrlens{1}_length[98]       0.004673    0.000044    0.000000    0.016511    0.002432    1.000    2      IlnBrlens{1}_length[99]       0.002411    0.000015    0.000000    0.009139    0.001081    1.000    2      IlnBrlens{1}_length[100]      0.233512    0.169896    0.000001    0.981733    0.075413    1.001    2      IlnBrlens{1}_length[101]      0.010433    0.000174    0.000001    0.033202    0.006408    1.000    2      IlnBrlens{1}_length[102]      0.008741    0.000116    0.000000    0.029068    0.005069    1.000    2      IlnBrlens{1}_length[103]      0.127870    0.041921    0.000002    0.496667    0.055533    1.001    2      IlnBrlens{1}_length[104]      0.248209    0.093605    0.000011    0.773327    0.159694    1.000    2      IlnBrlens{1}_length[105]      0.074846    0.011023    0.000003    0.263879    0.039545    1.000    2      IlnBrlens{1}_length[106]      0.215043    0.068739    0.000003    0.699771    0.133150    1.000    2      IlnBrlens{1}_length[107]      0.005760    0.000053    0.000001    0.019712    0.003262    1.000    2      IlnBrlens{1}_length[108]      0.116860    0.030043    0.000001    0.437646    0.057608    1.001    2      IlnBrlens{1}_length[109]      0.048053    0.003828    0.000002    0.157796    0.027197    1.002    2      IlnBrlens{1}_length[110]      1.045594   10.786167    0.000000    4.288569    0.234602    1.000    2      IlnBrlens{1}_length[111]      0.099619    0.020030    0.000001    0.372926    0.050184    1.000    2      IlnBrlens{1}_length[112]      0.140505    0.049167    0.000002    0.542131    0.061582    1.000    2      IlnBrlens{1}_length[113]      0.005652    0.000056    0.000000    0.019992    0.003022    1.001    2      IlnBrlens{1}_length[114]      0.161863    0.044838    0.000011    0.548168    0.091385    1.000    2      IlnBrlens{1}_length[115]      0.031987    0.001735    0.000003    0.110609    0.018132    1.000    2      IlnBrlens{1}_length[116]      0.008007    0.000074    0.000004    0.024040    0.005385    1.000    2      IlnBrlens{1}_length[117]      0.013397    0.000249    0.000000    0.042666    0.008399    1.000    2      IlnBrlens{1}_length[118]      0.157745    0.053147    0.000004    0.545148    0.085312    1.000    2      IlnBrlens{1}_length[119]      0.006793    0.000075    0.000000    0.023227    0.003868    1.001    2      IlnBrlens{1}_length[120]      0.062604    0.008656    0.000001    0.225332    0.030918    1.000    2      IlnBrlens{1}_length[121]      0.131863    0.031034    0.000005    0.462584    0.071590    1.000    2      IlnBrlens{1}_length[122]      0.160236    0.060336    0.000004    0.570803    0.082003    1.000    2      IlnBrlens{1}_rate[1]          2.494541    8.622971    0.019988    7.581260    1.634132    1.000    2      IlnBrlens{1}_rate[2]          0.876044    0.110308    0.095322    1.000000    1.000000    1.001    2      IlnBrlens{1}_rate[3]          0.682836    0.384066    0.023633    1.703305    0.532926    1.000    2      IlnBrlens{1}_rate[4]          1.019737    1.397499    0.005673    2.885771    0.757757    1.000    2      IlnBrlens{1}_rate[5]          0.859962    0.125975    0.056919    1.000000    1.000000    1.000    2      IlnBrlens{1}_rate[6]          0.943698    1.003690    0.007459    2.575282    0.775362    1.000    2      IlnBrlens{1}_rate[7]          0.470861    0.195660    0.006475    1.271416    0.345040    1.000    2      IlnBrlens{1}_rate[8]          0.976268    0.045028    0.427190    1.001246    1.000000    1.000    2      IlnBrlens{1}_rate[9]          0.848968    0.503979    0.021281    2.204200    0.667379    1.001    2      IlnBrlens{1}_rate[10]         1.067702    0.393196    0.081775    1.445968    1.000000    1.000    2      IlnBrlens{1}_rate[11]         0.993209    0.077833    0.203518    1.003378    1.000000    1.000    2      IlnBrlens{1}_rate[12]         0.656764    0.338117    0.013499    1.737583    0.497164    1.000    2      IlnBrlens{1}_rate[13]         0.452631    0.180509    0.008673    1.253033    0.326128    1.000    2      IlnBrlens{1}_rate[14]         0.548203    0.240969    0.013557    1.448943    0.415304    1.000    2      IlnBrlens{1}_rate[15]         0.441055    0.138337    0.009943    1.148004    0.340602    1.000    2      IlnBrlens{1}_rate[16]         0.823722    1.337601    0.006112    2.490883    0.524636    1.000    2      IlnBrlens{1}_rate[17]         0.911015    1.181108    0.011590    2.690330    0.595662    1.000    2      IlnBrlens{1}_rate[18]         0.402811    0.122357    0.009525    1.089762    0.300554    1.000    2      IlnBrlens{1}_rate[19]         1.514483    2.475102    0.032946    4.238809    1.071682    1.000    2      IlnBrlens{1}_rate[20]         0.917022    1.136032    0.009731    2.651363    0.627667    1.000    2      IlnBrlens{1}_rate[21]         0.640263    0.297984    0.017811    1.643652    0.496998    1.000    2      IlnBrlens{1}_rate[22]         1.066653    1.051474    0.022797    2.885560    0.781478    1.000    2      IlnBrlens{1}_rate[23]         1.083917    1.224358    0.016754    3.020309    0.772037    1.000    2      IlnBrlens{1}_rate[24]         0.531028    0.162546    0.015528    1.294964    0.435851    1.000    2      IlnBrlens{1}_rate[25]         0.695966    0.566614    0.009579    2.037692    0.470060    1.000    2      IlnBrlens{1}_rate[26]         0.673815    0.567963    0.008126    1.968758    0.463420    1.000    2      IlnBrlens{1}_rate[27]         0.662785    0.518486    0.003828    1.954469    0.450479    1.000    2      IlnBrlens{1}_rate[28]         1.345814    2.313037    0.008710    3.952301    0.926263    1.000    2      IlnBrlens{1}_rate[29]         1.010475    1.452433    0.011395    3.119904    0.656109    1.000    2      IlnBrlens{1}_rate[30]         0.429259    0.189544    0.007203    1.233239    0.303188    1.000    2      IlnBrlens{1}_rate[31]         0.481832    0.294593    0.004925    1.383062    0.325009    1.000    2      IlnBrlens{1}_rate[32]         0.811504    1.022082    0.007739    2.439688    0.521470    1.000    2      IlnBrlens{1}_rate[33]         0.655591    0.524957    0.008135    1.928339    0.441641    1.000    2      IlnBrlens{1}_rate[34]         1.103690    1.407505    0.007374    3.147001    0.766409    1.000    2      IlnBrlens{1}_rate[35]         0.787074    0.931898    0.005020    2.338848    0.514745    1.000    2      IlnBrlens{1}_rate[36]         0.775777    0.905137    0.011462    2.371586    0.499458    1.000    2      IlnBrlens{1}_rate[37]         1.243962    2.153809    0.014525    3.667586    0.841229    1.000    2      IlnBrlens{1}_rate[38]         0.688522    0.644853    0.006271    2.008378    0.466587    1.000    2      IlnBrlens{1}_rate[39]         1.216975    1.128594    0.040353    3.134315    0.928604    1.001    2      IlnBrlens{1}_rate[40]         0.958003    0.042454    0.343265    1.000000    1.000000    1.000    2      IlnBrlens{1}_rate[41]         0.783924    0.192347    0.013000    1.100765    1.000000    1.000    2      IlnBrlens{1}_rate[42]         1.474223    1.997299    0.053460    3.909568    1.000000    1.000    2      IlnBrlens{1}_rate[43]         0.985309    0.989581    0.013573    2.744175    0.719987    1.000    2      IlnBrlens{1}_rate[44]         1.337550    1.495537    0.071377    3.363990    1.000000    1.000    2      IlnBrlens{1}_rate[45]         0.573162    0.344539    0.004410    1.623731    0.397376    1.000    2      IlnBrlens{1}_rate[46]         1.997100    9.339036    0.005610    6.761399    1.039252    1.000    2      IlnBrlens{1}_rate[47]         5.865474   54.387253    0.265931   17.248506    3.762471    1.000    2      IlnBrlens{1}_rate[48]         0.390286    0.111008    0.010352    1.040499    0.297417    1.000    2      IlnBrlens{1}_rate[49]         0.723583    0.396847    0.004147    1.892343    0.553213    1.000    2      IlnBrlens{1}_rate[50]         1.523912    1.775135    0.039790    3.824643    1.165771    1.001    2      IlnBrlens{1}_rate[51]         0.661650    0.231325    0.033500    1.579509    0.544224    1.001    2      IlnBrlens{1}_rate[52]         1.334450    1.157730    0.082312    3.347784    1.046441    1.000    2      IlnBrlens{1}_rate[53]         0.367230    0.091882    0.010280    0.948776    0.283761    1.001    2      IlnBrlens{1}_rate[54]         0.955675    0.695807    0.019663    2.534661    0.732480    1.000    2      IlnBrlens{1}_rate[55]         0.481413    0.215168    0.007029    1.321286    0.345775    1.000    2      IlnBrlens{1}_rate[56]         1.692617    2.585413    0.034025    4.523203    1.245436    1.000    2      IlnBrlens{1}_rate[57]         0.518906    0.273910    0.004951    1.490440    0.366637    1.000    2      IlnBrlens{1}_rate[58]         7.891983  144.898118    0.016351   26.959921    4.253351    1.000    2      IlnBrlens{1}_rate[59]         1.293572    1.932896    0.016967    3.588488    0.919181    1.000    2      IlnBrlens{1}_rate[60]         0.684977    0.479326    0.009774    1.899360    0.494969    1.000    2      IlnBrlens{1}_rate[61]         0.765631    0.755883    0.003344    2.235979    0.519056    1.000    2      IlnBrlens{1}_rate[62]         1.111788    1.727642    0.009768    3.391960    0.711489    1.000    2      IlnBrlens{1}_rate[63]         0.666581    0.537009    0.007439    1.935524    0.450863    1.001    2      IlnBrlens{1}_rate[64]         0.887855    0.786662    0.010161    2.476207    0.652347    1.000    2      IlnBrlens{1}_rate[65]         0.660030    0.424926    0.011459    1.866840    0.470898    1.000    2      IlnBrlens{1}_rate[66]         0.443828    0.200758    0.007678    1.293724    0.309599    1.000    2      IlnBrlens{1}_rate[67]         0.833786    0.702787    0.008389    2.350415    0.596988    1.000    2      IlnBrlens{1}_rate[68]         0.475300    0.242450    0.007203    1.316144    0.340264    1.000    2      IlnBrlens{1}_rate[69]         0.753332    0.666176    0.006176    2.228543    0.515937    1.000    2      IlnBrlens{1}_rate[70]         1.096019    1.595461    0.002996    3.308704    0.717540    1.000    2      IlnBrlens{1}_rate[71]         0.701716    0.527092    0.003501    2.042149    0.494529    1.000    2      IlnBrlens{1}_rate[72]         1.262400    1.857083    0.021281    3.672535    0.877035    1.000    2      IlnBrlens{1}_rate[73]         0.590669    0.414035    0.003007    1.732223    0.401030    1.001    2      IlnBrlens{1}_rate[74]         0.713598    0.664960    0.005366    2.027885    0.490342    1.000    2      IlnBrlens{1}_rate[75]         0.546972    0.381986    0.006670    1.607248    0.370606    1.001    2      IlnBrlens{1}_rate[76]         0.797153    0.893768    0.005261    2.382672    0.517157    1.000    2      IlnBrlens{1}_rate[77]         2.996987   10.122291    0.037170    9.045023    2.057215    1.000    2      IlnBrlens{1}_rate[78]         0.908670    0.979231    0.008670    2.531299    0.638131    1.000    2      IlnBrlens{1}_rate[79]         0.972734    1.208706    0.015038    2.762400    0.662197    1.000    2      IlnBrlens{1}_rate[80]         1.572213    4.597540    0.008553    5.279921    0.892522    1.000    2      IlnBrlens{1}_rate[81]         0.883509    1.049482    0.011938    2.608977    0.597734    1.000    2      IlnBrlens{1}_rate[82]         0.813449    0.945213    0.007625    2.401913    0.534294    1.000    2      IlnBrlens{1}_rate[83]         0.688618    0.528576    0.009156    2.046694    0.468002    1.000    2      IlnBrlens{1}_rate[84]         0.724761    0.692425    0.004231    2.157638    0.489832    1.000    2      IlnBrlens{1}_rate[85]         0.739267    0.749973    0.007807    2.210093    0.495272    1.000    2      IlnBrlens{1}_rate[86]         1.308930    2.069067    0.011978    3.803509    0.885346    1.000    2      IlnBrlens{1}_rate[87]         1.842749    6.444904    0.009392    6.145760    1.019718    1.000    2      IlnBrlens{1}_rate[88]         1.478513    9.217132    0.007157    4.702485    0.765257    1.001    2      IlnBrlens{1}_rate[89]         0.880082    1.104008    0.003699    2.701941    0.558297    1.000    2      IlnBrlens{1}_rate[90]         0.809183    1.336904    0.008633    2.378406    0.517998    1.000    2      IlnBrlens{1}_rate[91]         0.630961    0.455727    0.001592    1.806392    0.429569    1.000    2      IlnBrlens{1}_rate[92]         0.978607    1.357121    0.012617    2.823841    0.647024    1.000    2      IlnBrlens{1}_rate[93]         0.650995    0.667275    0.008874    1.880722    0.428755    1.000    2      IlnBrlens{1}_rate[94]         0.818704    1.025648    0.003183    2.332533    0.543191    1.000    2      IlnBrlens{1}_rate[95]         1.074847    1.717686    0.012643    3.264593    0.708118    1.000    2      IlnBrlens{1}_rate[96]         1.158782    3.491922    0.005958    3.843399    0.640538    1.000    2      IlnBrlens{1}_rate[97]         0.692309    0.715458    0.005025    2.050607    0.463035    1.000    2      IlnBrlens{1}_rate[98]         0.706615    0.705311    0.011679    2.088950    0.472718    1.000    2      IlnBrlens{1}_rate[99]         0.761996    0.767150    0.005312    2.258182    0.503412    1.000    2      IlnBrlens{1}_rate[100]        1.479323    3.402275    0.006349    4.726101    0.911977    1.001    2      IlnBrlens{1}_rate[101]        0.675488    0.555494    0.014803    1.913624    0.461371    1.000    2      IlnBrlens{1}_rate[102]        0.788128    0.775857    0.005591    2.294205    0.541097    1.000    2      IlnBrlens{1}_rate[103]        1.369236    3.783649    0.007044    4.377260    0.798903    1.000    2      IlnBrlens{1}_rate[104]        2.643132    8.434022    0.042515    7.878578    1.787661    1.001    2      IlnBrlens{1}_rate[105]        0.963006    1.334291    0.004894    2.957214    0.621713    1.000    2      IlnBrlens{1}_rate[106]        1.290323    2.176845    0.013764    3.742431    0.895032    1.000    2      IlnBrlens{1}_rate[107]        0.633395    0.475563    0.010304    1.788357    0.423372    1.000    2      IlnBrlens{1}_rate[108]        1.026054    1.526284    0.001215    3.024034    0.655890    1.000    2      IlnBrlens{1}_rate[109]        1.000733    1.233317    0.003119    2.910598    0.710793    1.000    2      IlnBrlens{1}_rate[110]        3.946823   91.489869    0.015968   14.838359    1.531774    1.000    2      IlnBrlens{1}_rate[111]        1.648096    4.457287    0.015479    4.910720    1.019416    1.000    2      IlnBrlens{1}_rate[112]        1.273537    2.509387    0.007091    4.198248    0.746629    1.000    2      IlnBrlens{1}_rate[113]        0.956735    1.372846    0.008904    2.853301    0.636039    1.001    2      IlnBrlens{1}_rate[114]        1.019915    1.284785    0.011249    3.063591    0.676909    1.000    2      IlnBrlens{1}_rate[115]        0.695454    0.603490    0.008387    2.012826    0.479846    1.000    2      IlnBrlens{1}_rate[116]        0.520063    0.277054    0.013918    1.499542    0.357367    1.001    2      IlnBrlens{1}_rate[117]        0.809229    0.926536    0.014531    2.384644    0.539967    1.000    2      IlnBrlens{1}_rate[118]        1.382997    2.812185    0.014601    4.146762    0.906395    1.000    2      IlnBrlens{1}_rate[119]        0.653338    0.547324    0.004161    1.905874    0.450706    1.000    2      IlnBrlens{1}_rate[120]        0.870189    1.178352    0.013671    2.640839    0.553090    1.000    2      IlnBrlens{1}_rate[121]        0.906099    1.130983    0.005734    2.676376    0.591967    1.000    2      IlnBrlens{1}_rate[122]        1.346631    3.018598    0.019824    4.062952    0.866598    1.000    2      IlnBrlens{2}_length[1]        0.221982    0.068902    0.000004    0.616914    0.157328    1.000    2      IlnBrlens{2}_length[2]        0.020728    0.004564    0.000000    0.126267    0.000000    1.001    2      IlnBrlens{2}_length[3]        0.359440    0.132643    0.013837    0.957217    0.265117    1.000    2      IlnBrlens{2}_length[4]        0.152908    0.032394    0.000000    0.427697    0.112182    1.000    2      IlnBrlens{2}_length[5]        0.026912    0.005954    0.000000    0.153768    0.000000    1.000    2      IlnBrlens{2}_length[6]        0.169535    0.053045    0.000000    0.536274    0.107868    1.000    2      IlnBrlens{2}_length[7]        0.124498    0.013934    0.000000    0.325458    0.094697    1.001    2      IlnBrlens{2}_length[8]        0.001368    0.000047    0.000000    0.008284    0.000000    1.000    2      IlnBrlens{2}_length[9]        0.016874    0.000258    0.000000    0.044854    0.012500    1.000    2      IlnBrlens{2}_length[10]       0.024548    0.013730    0.000000    0.148703    0.000000    1.000    2      IlnBrlens{2}_length[11]       0.003475    0.000315    0.000000    0.020235    0.000000    1.000    2      IlnBrlens{2}_length[12]       0.048981    0.002042    0.000974    0.125178    0.037472    1.000    2      IlnBrlens{2}_length[13]       0.043654    0.001520    0.000640    0.110516    0.034219    1.000    2      IlnBrlens{2}_length[14]       0.063611    0.003545    0.001519    0.165199    0.048101    1.000    2      IlnBrlens{2}_length[15]       0.110225    0.009438    0.001687    0.275553    0.087865    1.000    2      IlnBrlens{2}_length[16]       0.017015    0.000468    0.000012    0.055994    0.010087    1.000    2      IlnBrlens{2}_length[17]       0.017163    0.000511    0.000014    0.056208    0.010112    1.000    2      IlnBrlens{2}_length[18]       0.097649    0.007215    0.001558    0.236695    0.078918    1.001    2      IlnBrlens{2}_length[19]       0.355197    0.195543    0.000000    1.020324    0.240074    1.000    2      IlnBrlens{2}_length[20]       0.266555    0.080461    0.000000    0.760017    0.186692    1.000    2      IlnBrlens{2}_length[21]       0.020641    0.000316    0.000308    0.051844    0.016382    1.001    2      IlnBrlens{2}_length[22]       0.023752    0.000504    0.000183    0.065327    0.017659    1.000    2      IlnBrlens{2}_length[23]       0.008106    0.000092    0.000023    0.024148    0.005333    1.000    2      IlnBrlens{2}_length[24]       0.146077    0.011860    0.003881    0.342799    0.122959    1.004    2      IlnBrlens{2}_length[25]       0.005354    0.000059    0.000003    0.017704    0.003064    1.000    2      IlnBrlens{2}_length[26]       0.006422    0.000074    0.000002    0.020953    0.003791    1.000    2      IlnBrlens{2}_length[27]       0.006276    0.000071    0.000003    0.020128    0.003748    1.000    2      IlnBrlens{2}_length[28]       0.016584    0.000375    0.000036    0.050077    0.010774    1.000    2      IlnBrlens{2}_length[29]       0.016977    0.000395    0.000023    0.052252    0.010811    1.000    2      IlnBrlens{2}_length[30]       0.038719    0.001163    0.000290    0.100177    0.029842    1.000    2      IlnBrlens{2}_length[31]       0.027293    0.000698    0.000052    0.075628    0.020070    1.000    2      IlnBrlens{2}_length[32]       0.003877    0.000042    0.000000    0.014632    0.001759    1.000    2      IlnBrlens{2}_length[33]       0.018003    0.000403    0.000037    0.053104    0.012056    1.000    2      IlnBrlens{2}_length[34]       0.013651    0.000262    0.000027    0.041760    0.008808    1.000    2      IlnBrlens{2}_length[35]       0.004032    0.000047    0.000000    0.015136    0.001783    1.000    2      IlnBrlens{2}_length[36]       0.008771    0.000128    0.000027    0.028018    0.005357    1.002    2      IlnBrlens{2}_length[37]       0.008955    0.000147    0.000033    0.028415    0.005449    1.001    2      IlnBrlens{2}_length[38]       0.007923    0.000085    0.000022    0.023679    0.005253    1.000    2      IlnBrlens{2}_length[39]       0.127005    0.012252    0.000000    0.331157    0.098971    1.002    2      IlnBrlens{2}_length[40]       0.000747    0.000017    0.000000    0.003789    0.000000    1.000    2      IlnBrlens{2}_length[41]       0.006400    0.000132    0.000000    0.026441    0.000000    1.000    2      IlnBrlens{2}_length[42]       0.053261    0.006099    0.000000    0.195676    0.025463    1.000    2      IlnBrlens{2}_length[43]       0.142019    0.016653    0.000000    0.374377    0.109621    1.001    2      IlnBrlens{2}_length[44]       0.042553    0.005908    0.000000    0.194034    0.000000    1.001    2      IlnBrlens{2}_length[45]       0.367041    0.138227    0.000000    0.944663    0.275250    1.000    2      IlnBrlens{2}_length[46]       0.143978    0.034667    0.000001    0.455157    0.089844    1.000    2      IlnBrlens{2}_length[47]       0.581428    0.192081    0.033748    1.352085    0.486099    1.000    2      IlnBrlens{2}_length[48]       0.144156    0.021245    0.001377    0.380500    0.108494    1.000    2      IlnBrlens{2}_length[49]       0.082477    0.004691    0.001089    0.203070    0.066590    1.001    2      IlnBrlens{2}_length[50]       0.189783    0.025311    0.002001    0.473096    0.152217    1.002    2      IlnBrlens{2}_length[51]       0.116618    0.007941    0.002086    0.279734    0.096424    1.002    2      IlnBrlens{2}_length[52]       0.434404    0.121941    0.015972    1.024067    0.358015    1.000    2      IlnBrlens{2}_length[53]       0.253695    0.049979    0.008907    0.608633    0.207909    1.000    2      IlnBrlens{2}_length[54]       0.104167    0.013118    0.000987    0.288226    0.073342    1.000    2      IlnBrlens{2}_length[55]       0.015965    0.000227    0.000080    0.042908    0.012057    1.000    2      IlnBrlens{2}_length[56]       0.130226    0.018363    0.001194    0.368251    0.092010    1.000    2      IlnBrlens{2}_length[57]       0.016417    0.000375    0.000063    0.049939    0.010845    1.001    2      IlnBrlens{2}_length[58]       0.529858    0.306662    0.000989    1.419539    0.390890    1.000    2      IlnBrlens{2}_length[59]       0.017906    0.000378    0.000000    0.053754    0.012090    1.000    2      IlnBrlens{2}_length[60]       0.312636    0.139722    0.000024    0.875518    0.220039    1.000    2      IlnBrlens{2}_length[61]       0.010430    0.000186    0.000000    0.033670    0.006225    1.000    2      IlnBrlens{2}_length[62]       0.190773    0.039244    0.001052    0.536615    0.135298    1.000    2      IlnBrlens{2}_length[63]       0.080891    0.011594    0.000000    0.256875    0.049898    1.000    2      IlnBrlens{2}_length[64]       0.098314    0.015938    0.000001    0.299079    0.062620    1.000    2      IlnBrlens{2}_length[65]       0.018697    0.000419    0.000002    0.055569    0.012556    1.000    2      IlnBrlens{2}_length[66]       0.028625    0.000728    0.000000    0.076470    0.021557    1.001    2      IlnBrlens{2}_length[67]       0.023972    0.000656    0.000001    0.070767    0.016330    1.000    2      IlnBrlens{2}_length[68]       0.027253    0.000714    0.000001    0.074187    0.020311    1.000    2      IlnBrlens{2}_length[69]       0.214260    0.081434    0.000008    0.662946    0.136744    1.001    2      IlnBrlens{2}_length[70]       0.209466    0.069555    0.000013    0.608019    0.141152    1.000    2      IlnBrlens{2}_length[71]       0.049785    0.002390    0.000003    0.140464    0.036142    1.000    2      IlnBrlens{2}_length[72]       0.240017    0.100670    0.000010    0.705275    0.159024    1.000    2      IlnBrlens{2}_length[73]       0.016803    0.000369    0.000001    0.051876    0.010840    1.000    2      IlnBrlens{2}_length[74]       0.017754    0.000464    0.000037    0.052678    0.011577    1.000    2      IlnBrlens{2}_length[75]       0.017071    0.000397    0.000001    0.052527    0.011012    1.000    2      IlnBrlens{2}_length[76]       0.086330    0.009486    0.000003    0.258606    0.057605    1.001    2      IlnBrlens{2}_length[77]       0.102681    0.013239    0.000005    0.303103    0.069415    1.000    2      IlnBrlens{2}_length[78]       0.207635    0.077346    0.000006    0.642354    0.130552    1.000    2      IlnBrlens{2}_length[79]       0.010577    0.000201    0.000000    0.034643    0.006136    1.000    2      IlnBrlens{2}_length[80]       0.070860    0.005762    0.000004    0.205981    0.049836    1.001    2      IlnBrlens{2}_length[81]       0.313148    0.136764    0.000005    0.938357    0.210122    1.000    2      IlnBrlens{2}_length[82]       0.008870    0.000126    0.000000    0.027254    0.005602    1.001    2      IlnBrlens{2}_length[83]       0.014292    0.000201    0.000000    0.038048    0.011035    1.000    2      IlnBrlens{2}_length[84]       0.004913    0.000058    0.000000    0.017664    0.002469    1.000    2      IlnBrlens{2}_length[85]       0.004787    0.000056    0.000000    0.017127    0.002368    1.000    2      IlnBrlens{2}_length[86]       0.177312    0.037900    0.000002    0.524884    0.120100    1.001    2      IlnBrlens{2}_length[87]       0.078459    0.007779    0.000005    0.235878    0.052862    1.000    2      IlnBrlens{2}_length[88]       0.228662    0.076227    0.000004    0.693875    0.151323    1.000    2      IlnBrlens{2}_length[89]       0.046364    0.003507    0.000001    0.151389    0.028040    1.000    2      IlnBrlens{2}_length[90]       0.075178    0.008756    0.000000    0.237539    0.046197    1.000    2      IlnBrlens{2}_length[91]       0.022258    0.000731    0.000006    0.066637    0.014790    1.000    2      IlnBrlens{2}_length[92]       0.203095    0.098069    0.000014    0.647855    0.119424    1.000    2      IlnBrlens{2}_length[93]       0.009324    0.000213    0.000000    0.032352    0.004756    1.000    2      IlnBrlens{2}_length[94]       0.133909    0.030174    0.000003    0.391414    0.087749    1.000    2      IlnBrlens{2}_length[95]       0.049701    0.003060    0.000002    0.146928    0.033947    1.000    2      IlnBrlens{2}_length[96]       0.187993    0.066086    0.000002    0.581417    0.115025    1.000    2      IlnBrlens{2}_length[97]       0.006094    0.000066    0.000000    0.019999    0.003536    1.000    2      IlnBrlens{2}_length[98]       0.007342    0.000109    0.000000    0.025612    0.003878    1.000    2      IlnBrlens{2}_length[99]       0.003678    0.000041    0.000000    0.013905    0.001647    1.000    2      IlnBrlens{2}_length[100]      0.129945    0.035807    0.000002    0.447655    0.069421    1.000    2      IlnBrlens{2}_length[101]      0.013933    0.000214    0.000001    0.040114    0.010145    1.000    2      IlnBrlens{2}_length[102]      0.011750    0.000226    0.000000    0.039238    0.006800    1.000    2      IlnBrlens{2}_length[103]      0.080749    0.009723    0.000002    0.254793    0.050333    1.000    2      IlnBrlens{2}_length[104]      0.112006    0.016634    0.000009    0.333606    0.074103    1.000    2      IlnBrlens{2}_length[105]      0.078829    0.013247    0.000004    0.259496    0.045377    1.000    2      IlnBrlens{2}_length[106]      0.181503    0.054123    0.000041    0.556074    0.117266    1.000    2      IlnBrlens{2}_length[107]      0.010587    0.000209    0.000001    0.037356    0.005683    1.000    2      IlnBrlens{2}_length[108]      0.116322    0.028802    0.000003    0.376844    0.067097    1.000    2      IlnBrlens{2}_length[109]      0.042308    0.002652    0.000003    0.134006    0.026244    1.002    2      IlnBrlens{2}_length[110]      0.193576    0.059393    0.000000    0.624099    0.118142    1.001    2      IlnBrlens{2}_length[111]      0.056825    0.006641    0.000002    0.187074    0.032495    1.000    2      IlnBrlens{2}_length[112]      0.081360    0.009173    0.000001    0.246013    0.053194    1.000    2      IlnBrlens{2}_length[113]      0.006813    0.000113    0.000000    0.024244    0.003461    1.001    2      IlnBrlens{2}_length[114]      0.250567    0.144346    0.000038    0.828004    0.141541    1.000    2      IlnBrlens{2}_length[115]      0.041850    0.002340    0.000003    0.133061    0.026265    1.001    2      IlnBrlens{2}_length[116]      0.016256    0.000352    0.000003    0.050096    0.010638    1.000    2      IlnBrlens{2}_length[117]      0.016627    0.000360    0.000000    0.051365    0.011079    1.001    2      IlnBrlens{2}_length[118]      0.116213    0.022032    0.000009    0.362589    0.071891    1.000    2      IlnBrlens{2}_length[119]      0.011142    0.000197    0.000000    0.036841    0.006511    1.000    2      IlnBrlens{2}_length[120]      0.071375    0.007591    0.000001    0.230464    0.043782    1.000    2      IlnBrlens{2}_length[121]      0.243211    0.160233    0.000010    0.811376    0.134001    1.000    2      IlnBrlens{2}_length[122]      0.123880    0.029040    0.000001    0.394395    0.073107    1.002    2      IlnBrlens{2}_rate[1]          0.978896    0.784685    0.020672    2.360455    0.830716    1.000    2      IlnBrlens{2}_rate[2]          0.991334    0.123262    0.250126    1.342761    1.000000    1.000    2      IlnBrlens{2}_rate[3]          1.538341    2.176129    0.095636    3.831521    1.149135    1.000    2      IlnBrlens{2}_rate[4]          1.054046    0.734409    0.021574    2.458787    0.917976    1.000    2      IlnBrlens{2}_rate[5]          0.981325    0.120298    0.167809    1.370260    1.000000    1.000    2      IlnBrlens{2}_rate[6]          1.105457    0.757813    0.075584    2.469460    1.000000    1.000    2      IlnBrlens{2}_rate[7]          0.902635    0.492175    0.024867    2.049844    0.793344    1.000    2      IlnBrlens{2}_rate[8]          0.984732    0.021770    0.684353    1.080168    1.000000    1.000    2      IlnBrlens{2}_rate[9]          1.304961    1.225648    0.054353    3.226524    1.022162    1.000    2      IlnBrlens{2}_rate[10]         0.997014    0.071556    0.395078    1.136134    1.000000    1.000    2      IlnBrlens{2}_rate[11]         0.982840    0.028770    0.588002    1.102484    1.000000    1.000    2      IlnBrlens{2}_rate[12]         1.347424    1.323768    0.082178    3.396886    1.046323    1.001    2      IlnBrlens{2}_rate[13]         1.050473    0.797415    0.027214    2.548523    0.881453    1.000    2      IlnBrlens{2}_rate[14]         1.300228    1.227917    0.056568    3.206780    1.025195    1.001    2      IlnBrlens{2}_rate[15]         0.925869    0.520949    0.015050    2.086305    0.810834    1.000    2      IlnBrlens{2}_rate[16]         0.853974    0.435071    0.023606    1.962745    0.756601    1.000    2      IlnBrlens{2}_rate[17]         0.860919    0.432054    0.011084    1.960012    0.753383    1.000    2      IlnBrlens{2}_rate[18]         0.828306    0.455995    0.021605    1.836379    0.723391    1.000    2      IlnBrlens{2}_rate[19]         1.117803    1.167228    0.020271    2.719018    0.904922    1.000    2      IlnBrlens{2}_rate[20]         0.907931    0.531012    0.019562    2.065940    0.796962    1.000    2      IlnBrlens{2}_rate[21]         0.847010    0.391348    0.029037    1.921797    0.749548    1.000    2      IlnBrlens{2}_rate[22]         0.849804    0.421541    0.020971    1.936863    0.738433    1.001    2      IlnBrlens{2}_rate[23]         0.942220    0.621704    0.028428    2.253896    0.800391    1.000    2      IlnBrlens{2}_rate[24]         0.582972    0.161070    0.014955    1.258970    0.505985    1.003    2      IlnBrlens{2}_rate[25]         0.955792    0.733976    0.024583    2.307618    0.805402    1.000    2      IlnBrlens{2}_rate[26]         0.941147    0.666068    0.018097    2.251640    0.797311    1.000    2      IlnBrlens{2}_rate[27]         0.943532    0.681097    0.023157    2.225884    0.801617    1.000    2      IlnBrlens{2}_rate[28]         0.886045    0.531254    0.021094    2.011011    0.768475    1.000    2      IlnBrlens{2}_rate[29]         0.903761    0.558002    0.016267    2.149375    0.764169    1.001    2      IlnBrlens{2}_rate[30]         0.787727    0.314601    0.022213    1.762676    0.703248    1.001    2      IlnBrlens{2}_rate[31]         0.849383    0.426360    0.028135    1.933266    0.750335    1.000    2      IlnBrlens{2}_rate[32]         0.970216    0.804750    0.015279    2.288964    0.810960    1.000    2      IlnBrlens{2}_rate[33]         0.894755    0.542228    0.015132    2.061818    0.774197    1.000    2      IlnBrlens{2}_rate[34]         0.903660    0.536595    0.016613    2.079970    0.779338    1.001    2      IlnBrlens{2}_rate[35]         0.964269    0.820781    0.023112    2.311168    0.808356    1.001    2      IlnBrlens{2}_rate[36]         0.937527    0.679784    0.025783    2.194565    0.797795    1.000    2      IlnBrlens{2}_rate[37]         0.949723    0.639953    0.028354    2.259481    0.810190    1.000    2      IlnBrlens{2}_rate[38]         0.922175    0.569272    0.026542    2.176217    0.796923    1.000    2      IlnBrlens{2}_rate[39]         0.731194    0.248581    0.010815    1.590308    0.652876    1.001    2      IlnBrlens{2}_rate[40]         0.980973    0.021232    0.645321    1.087760    1.000000    1.000    2      IlnBrlens{2}_rate[41]         0.918895    0.189234    0.043925    1.374410    1.000000    1.000    2      IlnBrlens{2}_rate[42]         0.940116    0.224591    0.056495    1.652870    1.000000    1.000    2      IlnBrlens{2}_rate[43]         0.674369    0.209289    0.015921    1.409648    0.612551    1.001    2      IlnBrlens{2}_rate[44]         0.864687    0.128325    0.060518    1.227875    1.000000    1.001    2      IlnBrlens{2}_rate[45]         1.369560    1.741184    0.118465    3.348062    1.072159    1.000    2      IlnBrlens{2}_rate[46]         0.998546    0.846503    0.015642    2.406485    0.838489    1.000    2      IlnBrlens{2}_rate[47]         0.911403    0.387571    0.054965    1.969379    0.810115    1.000    2      IlnBrlens{2}_rate[48]         1.012381    0.758526    0.016957    2.411961    0.856186    1.000    2      IlnBrlens{2}_rate[49]         0.678925    0.216921    0.012574    1.465412    0.608105    1.002    2      IlnBrlens{2}_rate[50]         0.682564    0.230404    0.025723    1.471437    0.603476    1.001    2      IlnBrlens{2}_rate[51]         0.613010    0.174667    0.011599    1.333698    0.532831    1.004    2      IlnBrlens{2}_rate[52]         0.879200    0.412791    0.038356    1.962558    0.773337    1.000    2      IlnBrlens{2}_rate[53]         1.087515    0.845356    0.040736    2.509480    0.910750    1.000    2      IlnBrlens{2}_rate[54]         1.365494    1.689333    0.035417    3.527089    1.032623    1.000    2      IlnBrlens{2}_rate[55]         0.875538    0.438832    0.032056    2.011226    0.764348    1.000    2      IlnBrlens{2}_rate[56]         1.028325    0.668574    0.030185    2.378361    0.884456    1.000    2      IlnBrlens{2}_rate[57]         0.898568    0.520826    0.030813    2.078614    0.780682    1.000    2      IlnBrlens{2}_rate[58]         1.351464    1.619619    0.026052    3.420566    1.040612    1.000    2      IlnBrlens{2}_rate[59]         0.871819    0.487092    0.023735    2.030456    0.766117    1.000    2      IlnBrlens{2}_rate[60]         1.758474    4.297522    0.041536    4.827784    1.176944    1.000    2      IlnBrlens{2}_rate[61]         0.922913    0.620268    0.028142    2.127512    0.784088    1.000    2      IlnBrlens{2}_rate[62]         0.911417    0.556014    0.028917    2.115965    0.788683    1.000    2      IlnBrlens{2}_rate[63]         1.072425    1.036229    0.019695    2.575437    0.886547    1.000    2      IlnBrlens{2}_rate[64]         1.301275    1.785524    0.009896    3.372036    0.986085    1.000    2      IlnBrlens{2}_rate[65]         0.868809    0.461076    0.017774    2.009984    0.755565    1.000    2      IlnBrlens{2}_rate[66]         0.823188    0.381264    0.029153    1.881355    0.726276    1.001    2      IlnBrlens{2}_rate[67]         0.847521    0.422447    0.008689    1.938748    0.748570    1.000    2      IlnBrlens{2}_rate[68]         0.843409    0.391007    0.022514    1.909621    0.741458    1.000    2      IlnBrlens{2}_rate[69]         1.615424    3.560353    0.024067    4.603277    1.092891    1.001    2      IlnBrlens{2}_rate[70]         1.095093    1.149178    0.029207    2.764160    0.880091    1.000    2      IlnBrlens{2}_rate[71]         0.793508    0.361320    0.023200    1.759562    0.701729    1.000    2      IlnBrlens{2}_rate[72]         1.126252    1.280930    0.023435    2.809249    0.892104    1.000    2      IlnBrlens{2}_rate[73]         0.893729    0.494064    0.018293    2.082266    0.778962    1.000    2      IlnBrlens{2}_rate[74]         0.981906    0.718514    0.010859    2.384064    0.832781    1.000    2      IlnBrlens{2}_rate[75]         0.883329    0.500309    0.020711    2.065333    0.769933    1.000    2      IlnBrlens{2}_rate[76]         0.882366    0.513096    0.034726    2.008135    0.783418    1.000    2      IlnBrlens{2}_rate[77]         0.889580    0.501863    0.017101    2.074075    0.773971    1.000    2      IlnBrlens{2}_rate[78]         1.453655    2.282243    0.034421    3.832999    1.082521    1.000    2      IlnBrlens{2}_rate[79]         0.914013    0.577782    0.026152    2.146280    0.794080    1.000    2      IlnBrlens{2}_rate[80]         0.958144    0.587548    0.009691    2.177967    0.830191    1.000    2      IlnBrlens{2}_rate[81]         1.174124    1.280969    0.025544    2.988739    0.925342    1.000    2      IlnBrlens{2}_rate[82]         0.919928    0.562198    0.010569    2.124950    0.796617    1.000    2      IlnBrlens{2}_rate[83]         0.910037    0.547607    0.032680    2.154245    0.779490    1.000    2      IlnBrlens{2}_rate[84]         0.966263    0.713049    0.017044    2.317971    0.823494    1.000    2      IlnBrlens{2}_rate[85]         0.955763    0.708779    0.012510    2.286235    0.811760    1.000    2      IlnBrlens{2}_rate[86]         0.965346    0.623351    0.030941    2.157310    0.851473    1.000    2      IlnBrlens{2}_rate[87]         0.894680    0.507774    0.032510    2.027093    0.783681    1.000    2      IlnBrlens{2}_rate[88]         1.091021    1.088659    0.039579    2.652914    0.893553    1.000    2      IlnBrlens{2}_rate[89]         1.188049    1.258879    0.021366    2.983895    0.942367    1.000    2      IlnBrlens{2}_rate[90]         0.836982    0.451970    0.017370    1.914288    0.733982    1.000    2      IlnBrlens{2}_rate[91]         1.132182    1.216316    0.031020    2.728257    0.906918    1.000    2      IlnBrlens{2}_rate[92]         1.364563    3.891836    0.031182    3.455905    0.995388    1.000    2      IlnBrlens{2}_rate[93]         0.918326    0.581841    0.011692    2.154314    0.783778    1.000    2      IlnBrlens{2}_rate[94]         1.269616    1.436290    0.048116    3.044809    0.995811    1.000    2      IlnBrlens{2}_rate[95]         1.036860    0.790755    0.034220    2.489411    0.871183    1.000    2      IlnBrlens{2}_rate[96]         1.020188    1.080727    0.031421    2.542773    0.835011    1.000    2      IlnBrlens{2}_rate[97]         0.937140    0.610282    0.021769    2.202304    0.804427    1.000    2      IlnBrlens{2}_rate[98]         0.957088    0.794771    0.028830    2.323390    0.801443    1.001    2      IlnBrlens{2}_rate[99]         0.974260    0.795973    0.025587    2.352672    0.819218    1.000    2      IlnBrlens{2}_rate[100]        0.969439    0.892023    0.026508    2.305746    0.817876    1.000    2      IlnBrlens{2}_rate[101]        0.904505    0.618518    0.025491    2.201407    0.755807    1.001    2      IlnBrlens{2}_rate[102]        0.900535    0.529836    0.014131    2.047528    0.775474    1.000    2      IlnBrlens{2}_rate[103]        0.893056    0.561808    0.012676    2.112113    0.758703    1.000    2      IlnBrlens{2}_rate[104]        1.145006    1.059022    0.032219    2.843528    0.912408    1.001    2      IlnBrlens{2}_rate[105]        0.948660    0.850056    0.032018    2.439704    0.755198    1.000    2      IlnBrlens{2}_rate[106]        1.031387    0.911847    0.028235    2.495366    0.863130    1.000    2      IlnBrlens{2}_rate[107]        0.931017    0.557417    0.036211    2.268996    0.795890    1.001    2      IlnBrlens{2}_rate[108]        0.993363    0.980329    0.016519    2.331670    0.818356    1.000    2      IlnBrlens{2}_rate[109]        0.838519    0.478663    0.039292    1.895943    0.745126    1.000    2      IlnBrlens{2}_rate[110]        0.987642    0.710556    0.016096    2.437049    0.832058    1.000    2      IlnBrlens{2}_rate[111]        0.917089    0.647120    0.014640    2.133309    0.785968    1.001    2      IlnBrlens{2}_rate[112]        0.780697    0.389765    0.030842    1.707904    0.700407    1.000    2      IlnBrlens{2}_rate[113]        0.944324    0.681402    0.031794    2.264928    0.799666    1.000    2      IlnBrlens{2}_rate[114]        1.439386    2.940794    0.045404    3.911004    0.998553    1.001    2      IlnBrlens{2}_rate[115]        0.827543    0.427836    0.026508    1.884106    0.723140    1.001    2      IlnBrlens{2}_rate[116]        0.882952    0.479271    0.010130    2.080031    0.744047    1.000    2      IlnBrlens{2}_rate[117]        0.878998    0.421003    0.021661    2.046068    0.774253    1.000    2      IlnBrlens{2}_rate[118]        0.992794    0.822564    0.025761    2.297874    0.836921    1.001    2      IlnBrlens{2}_rate[119]        0.891411    0.477900    0.007017    2.039138    0.795916    1.001    2      IlnBrlens{2}_rate[120]        0.930868    0.621240    0.019323    2.239570    0.781811    1.001    2      IlnBrlens{2}_rate[121]        1.475374    6.646889    0.028198    4.013220    1.024995    1.000    2      IlnBrlens{2}_rate[122]        1.031474    1.074859    0.030109    2.499833    0.847752    1.000    2      IlnBrlens{3}_length[1]        0.177232    0.091552    0.000003    0.662223    0.080337    1.000    2      IlnBrlens{3}_length[2]        0.010652    0.002201    0.000000    0.057399    0.000000    1.001    2      IlnBrlens{3}_length[3]        0.106500    0.025197    0.000000    0.334431    0.063240    1.000    2      IlnBrlens{3}_length[4]        0.095259    0.028582    0.000000    0.346315    0.044783    1.000    2      IlnBrlens{3}_length[5]        0.012715    0.002783    0.000000    0.064880    0.000000    1.000    2      IlnBrlens{3}_length[6]        0.089869    0.031854    0.000000    0.348863    0.034870    1.000    2      IlnBrlens{3}_length[7]        0.092443    0.022560    0.000000    0.330775    0.046249    1.000    2      IlnBrlens{3}_length[8]        0.001008    0.000050    0.000000    0.004109    0.000000    1.000    2      IlnBrlens{3}_length[9]        0.011262    0.000415    0.000000    0.040999    0.005212    1.000    2      IlnBrlens{3}_length[10]       0.010353    0.005385    0.000000    0.047910    0.000000    1.000    2      IlnBrlens{3}_length[11]       0.001426    0.000083    0.000000    0.006161    0.000000    1.000    2      IlnBrlens{3}_length[12]       0.022126    0.001070    0.000047    0.075172    0.011935    1.000    2      IlnBrlens{3}_length[13]       0.024220    0.001272    0.000066    0.083359    0.013219    1.000    2      IlnBrlens{3}_length[14]       0.027431    0.001635    0.000096    0.092074    0.014770    1.000    2      IlnBrlens{3}_length[15]       0.053747    0.004635    0.000229    0.174726    0.031631    1.000    2      IlnBrlens{3}_length[16]       0.013408    0.000688    0.000001    0.051517    0.005483    1.000    2      IlnBrlens{3}_length[17]       0.013554    0.000679    0.000002    0.051540    0.005682    1.000    2      IlnBrlens{3}_length[18]       0.054632    0.004724    0.000185    0.180272    0.032637    1.000    2      IlnBrlens{3}_length[19]       0.263336    0.136052    0.000000    0.859789    0.153154    1.000    2      IlnBrlens{3}_length[20]       0.165301    0.084276    0.000000    0.572248    0.083702    1.000    2      IlnBrlens{3}_length[21]       0.005990    0.000038    0.000040    0.017952    0.004091    1.000    2      IlnBrlens{3}_length[22]       0.014111    0.000139    0.000108    0.036399    0.010998    1.000    2      IlnBrlens{3}_length[23]       0.002851    0.000011    0.000002    0.009139    0.001752    1.000    2      IlnBrlens{3}_length[24]       0.022193    0.000507    0.000561    0.058321    0.016688    1.000    2      IlnBrlens{3}_length[25]       0.002033    0.000007    0.000001    0.007069    0.001104    1.000    2      IlnBrlens{3}_length[26]       0.004449    0.000022    0.000018    0.013287    0.003017    1.000    2      IlnBrlens{3}_length[27]       0.004584    0.000023    0.000008    0.013754    0.003102    1.000    2      IlnBrlens{3}_length[28]       0.005470    0.000033    0.000017    0.016244    0.003696    1.000    2      IlnBrlens{3}_length[29]       0.005605    0.000033    0.000019    0.016598    0.003878    1.000    2      IlnBrlens{3}_length[30]       0.056755    0.002152    0.000141    0.140768    0.046768    1.000    2      IlnBrlens{3}_length[31]       0.014385    0.000148    0.000130    0.037329    0.011269    1.000    2      IlnBrlens{3}_length[32]       0.001540    0.000006    0.000000    0.005757    0.000711    1.000    2      IlnBrlens{3}_length[33]       0.016713    0.000149    0.000847    0.040131    0.013653    1.000    2      IlnBrlens{3}_length[34]       0.003967    0.000020    0.000005    0.012357    0.002559    1.000    2      IlnBrlens{3}_length[35]       0.001592    0.000006    0.000000    0.005951    0.000731    1.000    2      IlnBrlens{3}_length[36]       0.005296    0.000028    0.000024    0.015493    0.003696    1.000    2      IlnBrlens{3}_length[37]       0.003072    0.000015    0.000004    0.010094    0.001805    1.000    2      IlnBrlens{3}_length[38]       0.005185    0.000027    0.000024    0.015020    0.003634    1.000    2      IlnBrlens{3}_length[39]       0.155874    0.023401    0.000000    0.423223    0.116044    1.000    2      IlnBrlens{3}_length[40]       0.000199    0.000001    0.000000    0.000924    0.000000    1.000    2      IlnBrlens{3}_length[41]       0.001834    0.000013    0.000000    0.008837    0.000000    1.000    2      IlnBrlens{3}_length[42]       0.043873    0.010114    0.000000    0.198337    0.008710    1.000    2      IlnBrlens{3}_length[43]       0.151297    0.044829    0.000000    0.502586    0.087568    1.000    2      IlnBrlens{3}_length[44]       0.030764    0.007137    0.000000    0.148276    0.000000    1.000    2      IlnBrlens{3}_length[45]       0.655358    0.953634    0.000000    2.099577    0.377349    1.000    2      IlnBrlens{3}_length[46]       0.131545    0.082784    0.000001    0.520670    0.046882    1.000    2      IlnBrlens{3}_length[47]       0.379173    0.307474    0.002121    1.199891    0.226326    1.000    2      IlnBrlens{3}_length[48]       0.065689    0.006347    0.000297    0.210583    0.040159    1.000    2      IlnBrlens{3}_length[49]       0.195658    0.057393    0.000084    0.655064    0.102982    1.001    2      IlnBrlens{3}_length[50]       0.022789    0.000543    0.000281    0.058885    0.016940    1.001    2      IlnBrlens{3}_length[51]       0.418836    0.156631    0.003406    1.149365    0.305086    1.001    2      IlnBrlens{3}_length[52]       0.256624    0.084264    0.001373    0.745774    0.175010    1.000    2      IlnBrlens{3}_length[53]       0.085707    0.010477    0.000723    0.264458    0.053663    1.000    2      IlnBrlens{3}_length[54]       0.063310    0.006882    0.000087    0.208180    0.036535    1.000    2      IlnBrlens{3}_length[55]       0.025168    0.000294    0.000971    0.058986    0.021170    1.000    2      IlnBrlens{3}_length[56]       0.219411    0.129898    0.000072    0.728285    0.119035    1.000    2      IlnBrlens{3}_length[57]       0.026732    0.000386    0.000002    0.063204    0.022246    1.000    2      IlnBrlens{3}_length[58]       0.356910    0.385093    0.000013    1.253033    0.171984    1.000    2      IlnBrlens{3}_length[59]       0.007872    0.000068    0.000000    0.024027    0.005341    1.000    2      IlnBrlens{3}_length[60]       0.248607    0.159790    0.000007    0.852887    0.129455    1.000    2      IlnBrlens{3}_length[61]       0.004755    0.000026    0.000000    0.014652    0.003140    1.000    2      IlnBrlens{3}_length[62]       2.715573   10.993704    0.000003    7.538469    1.901501    1.000    2      IlnBrlens{3}_length[63]       0.038609    0.003627    0.000001    0.143067    0.018342    1.000    2      IlnBrlens{3}_length[64]       0.039511    0.003745    0.000003    0.140119    0.020445    1.000    2      IlnBrlens{3}_length[65]       0.025283    0.000474    0.000004    0.066235    0.019916    1.000    2      IlnBrlens{3}_length[66]       0.127190    0.014143    0.000001    0.344072    0.102703    1.000    2      IlnBrlens{3}_length[67]       0.033202    0.001572    0.000000    0.103376    0.021424    1.000    2      IlnBrlens{3}_length[68]       0.108428    0.012935    0.000000    0.319466    0.080975    1.000    2      IlnBrlens{3}_length[69]       0.165772    0.134089    0.000001    0.647562    0.059777    1.000    2      IlnBrlens{3}_length[70]       0.716681    0.850526    0.000003    2.251985    0.450182    1.000    2      IlnBrlens{3}_length[71]       0.401932    0.118609    0.000002    1.011872    0.324213    1.001    2      IlnBrlens{3}_length[72]       0.177163    0.109537    0.000006    0.661690    0.075930    1.000    2      IlnBrlens{3}_length[73]       0.013878    0.000165    0.000001    0.038166    0.010417    1.000    2      IlnBrlens{3}_length[74]       0.012500    0.000443    0.000000    0.046593    0.005598    1.000    2      IlnBrlens{3}_length[75]       0.017495    0.000379    0.000000    0.054288    0.011510    1.003    2      IlnBrlens{3}_length[76]       0.131183    0.029218    0.000000    0.449360    0.075706    1.001    2      IlnBrlens{3}_length[77]       0.095143    0.023017    0.000001    0.352064    0.044568    1.000    2      IlnBrlens{3}_length[78]       0.110506    0.040303    0.000002    0.415258    0.048407    1.000    2      IlnBrlens{3}_length[79]       0.003560    0.000021    0.000000    0.012120    0.002024    1.000    2      IlnBrlens{3}_length[80]       0.065590    0.014729    0.000001    0.254043    0.027140    1.000    2      IlnBrlens{3}_length[81]       0.172127    0.121556    0.000000    0.605450    0.081516    1.000    2      IlnBrlens{3}_length[82]       0.004623    0.000042    0.000000    0.016324    0.002441    1.000    2      IlnBrlens{3}_length[83]       0.004930    0.000034    0.000000    0.015797    0.003081    1.001    2      IlnBrlens{3}_length[84]       0.002171    0.000010    0.000000    0.008033    0.001061    1.000    2      IlnBrlens{3}_length[85]       0.002040    0.000009    0.000000    0.007515    0.000996    1.000    2      IlnBrlens{3}_length[86]       0.219301    0.292131    0.000010    0.899314    0.066868    1.001    2      IlnBrlens{3}_length[87]       0.070598    0.014544    0.000001    0.273400    0.029857    1.000    2      IlnBrlens{3}_length[88]       0.190963    0.202316    0.000000    0.770370    0.070917    1.001    2      IlnBrlens{3}_length[89]       0.010299    0.000200    0.000000    0.033715    0.006018    1.001    2      IlnBrlens{3}_length[90]       0.074931    0.019072    0.000000    0.290024    0.030517    1.000    2      IlnBrlens{3}_length[91]       0.013255    0.000560    0.000000    0.048654    0.006018    1.000    2      IlnBrlens{3}_length[92]       0.105835    0.036845    0.000004    0.405267    0.044505    1.000    2      IlnBrlens{3}_length[93]       0.005412    0.000077    0.000000    0.020284    0.002453    1.000    2      IlnBrlens{3}_length[94]       0.071293    0.016983    0.000001    0.270199    0.030921    1.000    2      IlnBrlens{3}_length[95]       0.011257    0.000300    0.000000    0.034390    0.006706    1.001    2      IlnBrlens{3}_length[96]       0.214933    0.209929    0.000001    0.837638    0.074829    1.001    2      IlnBrlens{3}_length[97]       0.004286    0.000032    0.000000    0.015235    0.002295    1.000    2      IlnBrlens{3}_length[98]       0.003918    0.000024    0.000000    0.013268    0.002245    1.000    2      IlnBrlens{3}_length[99]       0.001510    0.000006    0.000000    0.005716    0.000680    1.000    2      IlnBrlens{3}_length[100]      1.083706    3.809543    0.000001    4.640211    0.155884    1.001    2      IlnBrlens{3}_length[101]      0.004668    0.000035    0.000000    0.016079    0.002648    1.001    2      IlnBrlens{3}_length[102]      0.007499    0.000099    0.000000    0.026221    0.004055    1.001    2      IlnBrlens{3}_length[103]      0.077658    0.026177    0.000002    0.293520    0.029245    1.002    2      IlnBrlens{3}_length[104]      0.120839    0.064983    0.000004    0.444142    0.050284    1.001    2      IlnBrlens{3}_length[105]      0.112075    0.077399    0.000004    0.469228    0.031280    1.000    2      IlnBrlens{3}_length[106]      0.269345    0.572751    0.000001    1.107470    0.068812    1.002    2      IlnBrlens{3}_length[107]      0.005369    0.000032    0.000000    0.015887    0.003637    1.000    2      IlnBrlens{3}_length[108]      0.113924    0.084602    0.000000    0.442938    0.036726    1.000    2      IlnBrlens{3}_length[109]      0.010195    0.000158    0.000001    0.032735    0.006401    1.000    2      IlnBrlens{3}_length[110]      0.149643    0.147542    0.000001    0.539659    0.055989    1.002    2      IlnBrlens{3}_length[111]      0.012236    0.000239    0.000001    0.039030    0.007598    1.000    2      IlnBrlens{3}_length[112]      0.094987    0.033393    0.000000    0.402468    0.033788    1.001    2      IlnBrlens{3}_length[113]      0.002474    0.000012    0.000000    0.008717    0.001300    1.000    2      IlnBrlens{3}_length[114]      0.159508    0.087243    0.000011    0.632755    0.065701    1.000    2      IlnBrlens{3}_length[115]      0.010754    0.000211    0.000002    0.033997    0.006662    1.002    2      IlnBrlens{3}_length[116]      0.026941    0.000515    0.000001    0.068470    0.021777    1.000    2      IlnBrlens{3}_length[117]      0.023188    0.002230    0.000000    0.092617    0.007992    1.003    2      IlnBrlens{3}_length[118]      0.141929    0.179809    0.000001    0.537429    0.038985    1.002    2      IlnBrlens{3}_length[119]      0.006601    0.000064    0.000000    0.022079    0.003904    1.000    2      IlnBrlens{3}_length[120]      0.063614    0.022390    0.000001    0.244470    0.023540    1.001    2      IlnBrlens{3}_length[121]      0.118489    0.038598    0.000003    0.455742    0.052264    1.001    2      IlnBrlens{3}_length[122]      0.202911    0.424638    0.000005    0.997260    0.038431    1.002    2      IlnBrlens{3}_rate[1]          0.829097    1.903405    0.001498    2.898127    0.413094    1.000    2      IlnBrlens{3}_rate[2]          0.876297    0.155248    0.058050    1.000000    1.000000    1.000    2      IlnBrlens{3}_rate[3]          0.459768    0.375045    0.002613    1.426859    0.285120    1.000    2      IlnBrlens{3}_rate[4]          0.715365    1.195028    0.002166    2.301887    0.404060    1.000    2      IlnBrlens{3}_rate[5]          0.831827    0.236031    0.046254    1.000000    1.000000    1.000    2      IlnBrlens{3}_rate[6]          0.689845    0.914967    0.000717    1.922369    0.455472    1.000    2      IlnBrlens{3}_rate[7]          0.682406    0.969321    0.002904    2.297777    0.378233    1.000    2      IlnBrlens{3}_rate[8]          0.965247    0.056537    0.320953    1.000000    1.000000    1.000    2      IlnBrlens{3}_rate[9]          0.863194    1.831788    0.003512    3.091765    0.434778    1.000    2      IlnBrlens{3}_rate[10]         0.922513    0.093974    0.136317    1.000000    1.000000    1.000    2      IlnBrlens{3}_rate[11]         0.937187    0.059736    0.203887    1.000000    1.000000    1.000    2      IlnBrlens{3}_rate[12]         0.613075    0.782005    0.002496    2.041627    0.343020    1.000    2      IlnBrlens{3}_rate[13]         0.584751    0.646971    0.001965    1.961494    0.332572    1.000    2      IlnBrlens{3}_rate[14]         0.568567    0.648677    0.002031    1.861680    0.321075    1.000    2      IlnBrlens{3}_rate[15]         0.462138    0.316195    0.002529    1.489731    0.279776    1.000    2      IlnBrlens{3}_rate[16]         0.715854    1.199844    0.001944    2.454304    0.374221    1.000    2      IlnBrlens{3}_rate[17]         0.735609    1.335321    0.004090    2.555787    0.391142    1.000    2      IlnBrlens{3}_rate[18]         0.470319    0.326787    0.001912    1.508229    0.287867    1.000    2      IlnBrlens{3}_rate[19]         0.991396    2.081656    0.004153    3.228002    0.580392    1.000    2      IlnBrlens{3}_rate[20]         0.655103    1.446572    0.002194    2.136223    0.339510    1.000    2      IlnBrlens{3}_rate[21]         0.263980    0.083176    0.000599    0.772375    0.177856    1.000    2      IlnBrlens{3}_rate[22]         0.629632    0.460000    0.007078    1.834303    0.432380    1.000    2      IlnBrlens{3}_rate[23]         0.402759    0.256955    0.003378    1.279966    0.247589    1.000    2      IlnBrlens{3}_rate[24]         0.089947    0.009098    0.001717    0.230102    0.067002    1.000    2      IlnBrlens{3}_rate[25]         0.459415    0.373038    0.000934    1.545749    0.265301    1.000    2      IlnBrlens{3}_rate[26]         0.952199    1.522249    0.003122    2.955245    0.577613    1.000    2      IlnBrlens{3}_rate[27]         0.991619    1.699125    0.004356    3.068766    0.615547    1.000    2      IlnBrlens{3}_rate[28]         0.385897    0.234611    0.001328    1.200711    0.241330    1.000    2      IlnBrlens{3}_rate[29]         0.394834    0.231105    0.004060    1.216506    0.250170    1.000    2      IlnBrlens{3}_rate[30]         1.500445    3.136183    0.003281    4.424186    1.015421    1.000    2      IlnBrlens{3}_rate[31]         0.689637    0.944850    0.004831    2.414794    0.385523    1.000    2      IlnBrlens{3}_rate[32]         0.563226    0.783913    0.002863    1.884088    0.304042    1.000    2      IlnBrlens{3}_rate[33]         1.180792    1.500883    0.016557    3.470068    0.815830    1.000    2      IlnBrlens{3}_rate[34]         0.371418    0.359278    0.001228    1.214099    0.207190    1.000    2      IlnBrlens{3}_rate[35]         0.550753    0.686464    0.002480    1.869832    0.302070    1.000    2      IlnBrlens{3}_rate[36]         0.767831    0.765071    0.003625    2.291673    0.502662    1.000    2      IlnBrlens{3}_rate[37]         0.415155    0.300802    0.001558    1.356614    0.245124    1.000    2      IlnBrlens{3}_rate[38]         0.770132    0.845388    0.006482    2.285867    0.509204    1.000    2      IlnBrlens{3}_rate[39]         1.191630    2.110175    0.004237    3.571399    0.783351    1.000    2      IlnBrlens{3}_rate[40]         0.932733    0.055790    0.179335    1.000000    1.000000    1.000    2      IlnBrlens{3}_rate[41]         0.647753    0.175134    0.039319    1.000021    1.000000    1.000    2      IlnBrlens{3}_rate[42]         0.860829    0.855113    0.002726    1.970050    1.000000    1.000    2      IlnBrlens{3}_rate[43]         0.744604    0.894953    0.005907    2.304141    0.475742    1.000    2      IlnBrlens{3}_rate[44]         0.763629    0.303483    0.004371    1.148858    1.000000    1.000    2      IlnBrlens{3}_rate[45]         2.489988   10.818069    0.005277    7.754065    1.518496    1.000    2      IlnBrlens{3}_rate[46]         0.906766    2.465755    0.002936    3.269683    0.447342    1.000    2      IlnBrlens{3}_rate[47]         0.600205    0.687347    0.001816    1.865908    0.365413    1.000    2      IlnBrlens{3}_rate[48]         0.495326    0.385635    0.001866    1.561511    0.306578    1.000    2      IlnBrlens{3}_rate[49]         1.545530    3.457271    0.002433    5.017228    0.902427    1.001    2      IlnBrlens{3}_rate[50]         0.093149    0.011896    0.001451    0.254975    0.065246    1.000    2      IlnBrlens{3}_rate[51]         2.233015    4.422571    0.029954    6.109813    1.647554    1.001    2      IlnBrlens{3}_rate[52]         0.525489    0.337238    0.004584    1.526528    0.365518    1.000    2      IlnBrlens{3}_rate[53]         0.369837    0.186247    0.003550    1.133533    0.234552    1.000    2      IlnBrlens{3}_rate[54]         0.877015    1.344268    0.004197    2.844843    0.512472    1.000    2      IlnBrlens{3}_rate[55]         1.683604    2.385590    0.009193    4.294138    1.277338    1.000    2      IlnBrlens{3}_rate[56]         1.822989    6.500191    0.004422    5.748369    1.113586    1.000    2      IlnBrlens{3}_rate[57]         2.229436    7.039370    0.005187    6.401896    1.553856    1.000    2      IlnBrlens{3}_rate[58]         0.886697    2.012819    0.002375    3.023406    0.479795    1.000    2      IlnBrlens{3}_rate[59]         0.489596    0.396073    0.003690    1.529436    0.301698    1.000    2      IlnBrlens{3}_rate[60]         1.309793    3.439503    0.002680    4.203216    0.768064    1.000    2      IlnBrlens{3}_rate[61]         0.612540    0.675096    0.002381    2.017293    0.361834    1.000    2      IlnBrlens{3}_rate[62]        15.508018  346.427420    0.007953   45.810516   10.282379    1.000    2      IlnBrlens{3}_rate[63]         0.621080    1.261531    0.001497    2.083812    0.331547    1.000    2      IlnBrlens{3}_rate[64]         0.602452    0.859919    0.001821    1.982887    0.333851    1.000    2      IlnBrlens{3}_rate[65]         1.690437    4.633418    0.005340    5.039445    1.080137    1.000    2      IlnBrlens{3}_rate[66]         4.380930   27.934000    0.001705   13.479978    2.868229    1.000    2      IlnBrlens{3}_rate[67]         1.430209    4.352899    0.001930    4.722063    0.818436    1.000    2      IlnBrlens{3}_rate[68]         3.876598   23.295566    0.000978   12.677502    2.412596    1.000    2      IlnBrlens{3}_rate[69]         1.181138    4.425301    0.002144    4.217946    0.556335    1.000    2      IlnBrlens{3}_rate[70]         4.092985   26.345716    0.002387   13.038908    2.576358    1.000    2      IlnBrlens{3}_rate[71]         7.700030   72.411167    0.010817   22.864222    5.353156    1.000    2      IlnBrlens{3}_rate[72]         0.901362    2.540578    0.001408    3.258716    0.421851    1.000    2      IlnBrlens{3}_rate[73]         1.067440    1.863554    0.002144    3.370494    0.657104    1.001    2      IlnBrlens{3}_rate[74]         0.732073    1.239674    0.003688    2.486627    0.381219    1.000    2      IlnBrlens{3}_rate[75]         1.283109    3.955023    0.001271    4.366663    0.668220    1.003    2      IlnBrlens{3}_rate[76]         1.506222    4.452468    0.007403    4.995606    0.852001    1.000    2      IlnBrlens{3}_rate[77]         0.859118    1.454453    0.002722    2.934702    0.480546    1.001    2      IlnBrlens{3}_rate[78]         0.820520    2.205876    0.001442    2.810552    0.424289    1.000    2      IlnBrlens{3}_rate[79]         0.417077    0.345225    0.001351    1.339050    0.239872    1.000    2      IlnBrlens{3}_rate[80]         0.905140    2.142028    0.002943    3.169452    0.450433    1.000    2      IlnBrlens{3}_rate[81]         0.722345    3.209418    0.001505    2.358931    0.366033    1.000    2      IlnBrlens{3}_rate[82]         0.597977    0.829783    0.001319    1.996824    0.326745    1.000    2      IlnBrlens{3}_rate[83]         0.333854    0.156038    0.002323    1.044929    0.213444    1.000    2      IlnBrlens{3}_rate[84]         0.592366    0.836399    0.003929    1.949595    0.330443    1.000    2      IlnBrlens{3}_rate[85]         0.572903    0.883265    0.001352    1.853270    0.310800    1.000    2      IlnBrlens{3}_rate[86]         1.166246    6.362318    0.003251    4.435584    0.465057    1.001    2      IlnBrlens{3}_rate[87]         0.829589    1.496826    0.002038    2.931389    0.449505    1.000    2      IlnBrlens{3}_rate[88]         1.015596    6.568149    0.001729    3.598543    0.426958    1.001    2      IlnBrlens{3}_rate[89]         0.354137    0.251958    0.001764    1.142295    0.210268    1.000    2      IlnBrlens{3}_rate[90]         0.872981    1.822284    0.002943    3.059204    0.457007    1.000    2      IlnBrlens{3}_rate[91]         0.720605    1.324152    0.004261    2.470545    0.371269    1.000    2      IlnBrlens{3}_rate[92]         0.779164    1.877396    0.003350    2.743877    0.405586    1.000    2      IlnBrlens{3}_rate[93]         0.698483    1.175657    0.001441    2.402900    0.358571    1.000    2      IlnBrlens{3}_rate[94]         0.711651    1.296947    0.003799    2.497696    0.366995    1.000    2      IlnBrlens{3}_rate[95]         0.295485    0.163931    0.002629    0.957656    0.175923    1.000    2      IlnBrlens{3}_rate[96]         1.332140   10.620255    0.001100    4.483401    0.534178    1.002    2      IlnBrlens{3}_rate[97]         0.824127    1.199515    0.002088    2.737038    0.477624    1.000    2      IlnBrlens{3}_rate[98]         0.738161    1.089477    0.002712    2.524732    0.413066    1.000    2      IlnBrlens{3}_rate[99]         0.578032    0.804326    0.001680    1.916637    0.316413    1.000    2      IlnBrlens{3}_rate[100]        5.865314  106.056911    0.005185   22.205072    2.045390    1.001    2      IlnBrlens{3}_rate[101]        0.335957    0.206606    0.002544    1.060189    0.201613    1.000    2      IlnBrlens{3}_rate[102]        0.774602    1.377139    0.001229    2.534577    0.414692    1.000    2      IlnBrlens{3}_rate[103]        0.870259    2.098842    0.003452    3.098420    0.443014    1.000    2      IlnBrlens{3}_rate[104]        1.201554    3.834082    0.005090    4.181816    0.606286    1.001    2      IlnBrlens{3}_rate[105]        1.217531    5.368922    0.002706    4.395395    0.520367    1.000    2      IlnBrlens{3}_rate[106]        1.336049    8.818156    0.000601    5.024853    0.511495    1.004    2      IlnBrlens{3}_rate[107]        0.773723    1.149113    0.007442    2.582591    0.467820    1.000    2      IlnBrlens{3}_rate[108]        0.992377    4.785010    0.003092    3.495147    0.435810    1.001    2      IlnBrlens{3}_rate[109]        0.293807    0.217299    0.002245    0.925491    0.165024    1.000    2      IlnBrlens{3}_rate[110]        0.803700    2.643814    0.001698    2.593193    0.391931    1.002    2      IlnBrlens{3}_rate[111]        0.287246    0.190993    0.001192    0.898411    0.164723    1.000    2      IlnBrlens{3}_rate[112]        0.844151    1.523357    0.002762    3.075618    0.445971    1.000    2      IlnBrlens{3}_rate[113]        0.483976    0.492905    0.002389    1.608612    0.277814    1.000    2      IlnBrlens{3}_rate[114]        0.951506    2.751566    0.001773    3.255101    0.504575    1.001    2      IlnBrlens{3}_rate[115]        0.306083    0.248094    0.002937    0.967426    0.169863    1.000    2      IlnBrlens{3}_rate[116]        2.051446    6.421943    0.005791    6.094972    1.340686    1.000    2      IlnBrlens{3}_rate[117]        1.192918    4.526187    0.009165    4.260550    0.541099    1.000    2      IlnBrlens{3}_rate[118]        1.099435    6.455188    0.003902    4.152402    0.436023    1.002    2      IlnBrlens{3}_rate[119]        0.704767    1.115408    0.001334    2.343488    0.403900    1.000    2      IlnBrlens{3}_rate[120]        0.823526    1.765799    0.002979    2.966142    0.413688    1.000    2      IlnBrlens{3}_rate[121]        0.811190    1.562072    0.003410    2.839541    0.436979    1.001    2      IlnBrlens{3}_rate[122]        1.278663    8.265914    0.004819    5.347388    0.460958    1.002    2      IlnBrlens{4,5}_length[1]      0.229569    0.070576    0.000001    0.666377    0.156413    1.000    2      IlnBrlens{4,5}_length[2]      0.017758    0.003381    0.000000    0.105774    0.000000    1.000    2      IlnBrlens{4,5}_length[3]      0.233407    0.047830    0.008582    0.603051    0.176251    1.000    2      IlnBrlens{4,5}_length[4]      0.137996    0.023902    0.000000    0.388879    0.099849    1.000    2      IlnBrlens{4,5}_length[5]      0.023531    0.004780    0.000000    0.134824    0.000000    1.001    2      IlnBrlens{4,5}_length[6]      0.144344    0.043684    0.000000    0.473930    0.088297    1.000    2      IlnBrlens{4,5}_length[7]      0.144030    0.026287    0.000000    0.400455    0.100456    1.000    2      IlnBrlens{4,5}_length[8]      0.001368    0.000054    0.000000    0.007703    0.000000    1.000    2      IlnBrlens{4,5}_length[9]      0.013444    0.000230    0.000000    0.035771    0.009662    1.000    2      IlnBrlens{4,5}_length[10]     0.020659    0.011533    0.000000    0.116637    0.000000    1.000    2      IlnBrlens{4,5}_length[11]     0.002955    0.000286    0.000000    0.016780    0.000000    1.000    2      IlnBrlens{4,5}_length[12]     0.024603    0.000045    0.012013    0.037555    0.024023    1.000    2      IlnBrlens{4,5}_length[13]     0.046919    0.000119    0.025300    0.068293    0.046303    1.001    2      IlnBrlens{4,5}_length[14]     0.032577    0.000088    0.014705    0.051141    0.031924    1.000    2      IlnBrlens{4,5}_length[15]     0.306417    0.003671    0.197851    0.426786    0.299123    1.000    2      IlnBrlens{4,5}_length[16]     0.021845    0.001212    0.000017    0.073503    0.011625    1.000    2      IlnBrlens{4,5}_length[17]     0.019787    0.000575    0.000018    0.067460    0.011522    1.000    2      IlnBrlens{4,5}_length[18]     0.279082    0.003131    0.177621    0.388542    0.273042    1.000    2      IlnBrlens{4,5}_length[19]     0.317170    0.138668    0.000000    0.916320    0.214412    1.000    2      IlnBrlens{4,5}_length[20]     0.294691    0.110603    0.000000    0.858127    0.201328    1.000    2      IlnBrlens{4,5}_length[21]     0.024522    0.000091    0.004017    0.041520    0.024683    1.000    2      IlnBrlens{4,5}_length[22]     0.026876    0.000387    0.001052    0.067165    0.021753    1.000    2      IlnBrlens{4,5}_length[23]     0.008192    0.000046    0.000095    0.022001    0.006274    1.000    2      IlnBrlens{4,5}_length[24]     0.139214    0.001712    0.061840    0.220880    0.135883    1.000    2      IlnBrlens{4,5}_length[25]     0.005701    0.000067    0.000004    0.018810    0.003235    1.000    2      IlnBrlens{4,5}_length[26]     0.007075    0.000099    0.000016    0.022946    0.004080    1.000    2      IlnBrlens{4,5}_length[27]     0.006838    0.000105    0.000003    0.022276    0.003931    1.000    2      IlnBrlens{4,5}_length[28]     0.023414    0.001148    0.000115    0.079556    0.012773    1.000    2      IlnBrlens{4,5}_length[29]     0.019709    0.000766    0.000087    0.062292    0.011908    1.000    2      IlnBrlens{4,5}_length[30]     0.050591    0.002761    0.001265    0.137898    0.036529    1.000    2      IlnBrlens{4,5}_length[31]     0.033965    0.001585    0.000313    0.099256    0.022861    1.001    2      IlnBrlens{4,5}_length[32]     0.004085    0.000060    0.000000    0.014823    0.001821    1.000    2      IlnBrlens{4,5}_length[33]     0.021054    0.000716    0.000233    0.063991    0.013308    1.000    2      IlnBrlens{4,5}_length[34]     0.015575    0.000405    0.000035    0.048908    0.009580    1.000    2      IlnBrlens{4,5}_length[35]     0.004376    0.000064    0.000000    0.016553    0.001889    1.000    2      IlnBrlens{4,5}_length[36]     0.009869    0.000212    0.000027    0.032206    0.005723    1.000    2      IlnBrlens{4,5}_length[37]     0.009801    0.000203    0.000040    0.031188    0.005693    1.001    2      IlnBrlens{4,5}_length[38]     0.008855    0.000116    0.000043    0.026464    0.005709    1.000    2      IlnBrlens{4,5}_length[39]     0.184034    0.045924    0.000000    0.520664    0.125550    1.000    2      IlnBrlens{4,5}_length[40]     0.000720    0.000019    0.000000    0.003467    0.000000    1.000    2      IlnBrlens{4,5}_length[41]     0.007074    0.000176    0.000000    0.029301    0.000000    1.000    2      IlnBrlens{4,5}_length[42]     0.055035    0.008026    0.000000    0.208918    0.025216    1.000    2      IlnBrlens{4,5}_length[43]     0.211262    0.052860    0.000000    0.585593    0.152311    1.000    2      IlnBrlens{4,5}_length[44]     0.052457    0.010539    0.000000    0.231865    0.000000    1.000    2
[truncated: 299,499 more chars]
