# Supplementary material for: The rise of predation in Jurassic lampreys
Source: Nat Commun. 2023 Oct 31;14:6652. doi: 10.1038/s41467-023-42251-0 (PMC10618186; doi:10.1038/s41467-023-42251-0)
Supplement: Supplementary file 4 — Supplementary Code 1-8 [file 41467_2023_42251_MOESM4_ESM.zip › Supplementary Codes 1-8/Supplementary Code 5.rtf]

Supplementary Code 5: Reconstruction of ancestral states of feeding mechanism (apparatus) 1 #NEXUS Begin MrBayes;       execute ../data/morph_molec.nex;               [partitions]       charset MVothers = 1-44 73-134 147-153 156-166 184-192 194 195;       charset BranchialApparatus = 45-63 135 146 193;       charset FeedingMechanism = 64-72 136-145 154 155 167-183 196-208;       charset 16S = 209-980;       charset CO1 = 981-1701;       partition five = 5: MVothers, BranchialApparatus, FeedingMechanism, 16S, CO1;       set partition = five;        [substitution model]       exclude 1 2 4-8 13-16 19 20 26 29 30 31 33 34 36 37 38 40 42 46 48-52 56 61 62 65 67 72 74 77 78 79 80 87 88 93 96 98                100 101 103 105 106 107 109 113-119 122 124 128 129 130 131 133 134 135 142 147 151 153 159 163 196;       lset applyto = (1,2,3) coding = variable rates = gamma; [Mkv+G]       lset applyto = (4,5) nst = 2 rates = gamma; [HKY+G]        unlink statefreq = (all) tratio = (all) shape = (4,5);       prset applyto = (all) ratepr = variable;        [constraints/outgroup]       constraint ingroup = 2-.;       constraint crown_lamprey = Lethenteron_camtschaticum Eudontomyzon_danfordi Eudontomyzon_morii Lampetra_fluviatilis Lampetra_ayresii                    Tetrapleurodon_spadiceus Entosphenus_macrostomus Entosphenus_minimus Entosphenus_similis Entosphenus_tridentatus                    Petromyzon_marinus Ichthyomyzon_bdellium Ichthyomyzon_castaneus Ichthyomyzon_unicuspis Caspiomyzon_wagneri                    Mordacia_mordax Mordacia_lapicida Geotria_australis;       constraint north_lamprey =  Lethenteron_camtschaticum Eudontomyzon_danfordi Eudontomyzon_morii Lampetra_fluviatilis Lampetra_ayresii                    Tetrapleurodon_spadiceus Entosphenus_macrostomus Entosphenus_minimus Entosphenus_similis Entosphenus_tridentatus                    Petromyzon_marinus Ichthyomyzon_bdellium Ichthyomyzon_castaneus Ichthyomyzon_unicuspis Caspiomyzon_wagneri;        [relaxed clock model]       prset clockratepr = lognorm(-6, 1.0);       prset clockvarpr = iln; [independent lognormal]       unlink ilnvar = (1,2,3);        [tip dates]       calibrate       Euconodonta = fixed(535.5)       Jamoytius = fixed(435.4)       Euphanerops = fixed(370)       Achanarella = fixed(385)       Ciderius = fixed(432)       Cornovichthys = fixed(385)       Lasanius = fixed(428.2)       Birkenia = fixed(435.4)       Rhyncholepis = fixed(431.95)       Myxinikela = fixed(280)       Tethymyxine = fixed(95)       Myxineidus = fixed(280)       Gilpichthys = fixed(280)             Mesomyzon = fixed(125)       Yanliaomyzon_occisor = fixed(158)       Yanliaomyzon_ingensdentes = fixed(163)       Priscomyzon = fixed(360)       Mayomyzon = fixed(280)       Hardistiella = fixed(320)       Pipiscius = fixed(280)       ;       prset nodeagepr = calibrated;        [fossilized birth-death model]       prset brlenspr = clock:fossilization;       prset samplestrat = random;       prset sampleprob = 0.3; [extant sampling prob]       prset speciationpr = exp(100);       prset extinctionpr = beta(2,1);       prset fossilizationpr = beta(1,9);       prset treeagepr = offsetexp(500, 600);       prset topologypr = constraint(ingroup, crown_lamprey, north_lamprey);     report applyto=(1,2,3) ancstates=yes;        [mcmc settings]       mcmcp ngen = 30000000 samplefr = 400 printfr = 10000 diagnfr = 50000;       mcmcp filename = run.te temp = 0.07;        mcmc;       sumt output = run.te.maj;       sumt output = run.te.all contype=allcompat;       sump;End; 
