# Supplementary material for: The rise of predation in Jurassic lampreys
Source: Nat Commun. 2023 Oct 31;14:6652. doi: 10.1038/s41467-023-42251-0 (PMC10618186; doi:10.1038/s41467-023-42251-0)
Supplement: Supplementary file 4 — Supplementary Code 1-8 [file 41467_2023_42251_MOESM4_ESM.zip › Supplementary Codes 1-8/Supplementary Code 7.rtf]

Supplementary Code 7: Reconstruction of ancestral areas of some key nodes (distribution partitions)  #NEXUS Begin data;       Dimensions ntax = 25 nchar = 1;       Format datatype = standard gap = - missing = ?;        Matrix       Geotria_australis          {17}  [Australia (southern) and Tasmania and South America]       Mordacia_lapicida          7     [South America]       Mordacia_mordax            1     [Australia (southern) and Tasmania]       Lethenteron_camtschaticum  {24}  [Asia and Western North America (Pacific)]       Eudontomyzon_morii         2     [Asia (North, NE)]       Eudontomyzon_danfordi      3     [Europe]       Lampetra_fluviatilis       3     [Europe]       Lampetra_ayresii           4     [Western North America (Pacific)]       Tetrapleurodon_spadiceus   4     [Western North America (Pacific)]       Entosphenus_tridentatus    {24}  [Asia (North, NE) and Western North America (Pacific)]       Entosphenus_macrostomus    4     [Western North America (Pacific)]       Entosphenus_minimus        4     [Western North America (Pacific)]       Entosphenus_similis        4     [Western North America (Pacific)]       Ichthyomyzon_bdellium      6     [Eastern North America (Atlantic)]       Ichthyomyzon_castaneus     6     [Eastern North America (Atlantic)]       Ichthyomyzon_unicuspis     6     [Eastern North America (Atlantic)]       Petromyzon_marinus         {36}  [Europe and  Eastern North America (Atlantic)]       Caspiomyzon_wagneri        3     [Europe ]       Mesomyzon_mengae           5     [East Laurasia (North China Craton)]       Yanliaomyzon_occior        5     [East Laurasia (North China Craton)]       Yanliaomyzon_ingensdentes  5     [East Laurasia (North China Craton)]       Hardistiella               8     [West Laurasia]       Mayomyzon                  8     [West Laurasia]       Pipiscius                  8     [West Laurasia]       Priscomyzon                0     [Africa (Southern Gondwana)]       ;End; Begin trees;   tree con_all_compat = (((((((((((((Lethenteron_camtschaticum:5.554189,(Eudontomyzon_danfordi:2.148957,Eudontomyzon_morii:2.148957):3.405232):3.032519,(Lampetra_fluviatilis:2.195836,Lampetra_ayresii:2.195836):6.390872):6.323132,(Tetrapleurodon_spadiceus:8.256678,(((Entosphenus_macrostomus:0.6169668,Entosphenus_tridentatus:0.6169668):2.193060,Entosphenus_similis:2.810027):2.699906,Entosphenus_minimus:5.509933):2.746745):6.653162):10.78734,((Petromyzon_marinus:7.322286,((Ichthyomyzon_bdellium:1.031436,Ichthyomyzon_unicuspis:1.031436):1.281146,Ichthyomyzon_castaneus:2.312583):5.009704):5.835086,Caspiomyzon_wagneri:13.15737):12.53981):32.90971,(Mordacia_mordax:4.648191,Mordacia_lapicida:4.648191):53.95870):19.36247,Geotria_australis:77.96936):80.03065,Yanliaomyzon_occior:8.147523e-06):5.000011,Yanliaomyzon_ingensdentes:1.934078e-05) :18.80828,Mesomyzon_mengae:56.80830):138.1917,Hardistiella:1.621613e-05):24.84012,Mayomyzon:34.84014):8.710507,Pipiscius:43.55065):36.56725,Priscomyzon:30.11790);End; Begin MrBayes;       [substitution model]       lset coding = variable;        [clock model]       prset clockratepr = gamma(2, 200);        [tip dates]       calibrate                   Mesomyzon_mengae = fixed(125)              Yanliaomyzon_occior = fixed(158)              Yanliaomyzon_ingensdentes = fixed(163)              Mayomyzon = fixed(310)              Pipiscius = fixed(310)              Hardistiella = fixed(320)              Priscomyzon = fixed(360)       ;       prset nodeagepr = calibrated;       prset brlenspr = clock:uniform;       prset treeagepr = offsetexp(300, 390);        [constraints]       constraint A = 1-21;       constraint B = 1-18 20 21;       constraint C = 1-18; [crown]       constraint D = 2-18;       constraint E = 4-18; [north]       constraint F = 4-13;       constraint G = 14-18;       prset topologypr = constraint(A, B, C, D, E, F, G);     [infer ancestral states]    report ancstates=yes;        [mcmc settings]       mcmcp nchain = 1 ngen = 1000000 samplefr = 100 printfr = 10000 diagnfr = 50000;        [disable tree moves]       propset ExtSPRClock(Tau,V)$prob=0;       propset NNIClock(Tau,V)$prob=0;       propset ParsSPRClock(Tau,V)$prob=0;       propset NodesliderClock(V)$prob=0;       propset TreeStretch(V)$prob=0;        startvals Tau = con_all_compat V = con_all_compat;        mcmc;       [sumt;]       sump;     End; 
