# Supplementary material for: The rise of predation in Jurassic lampreys
Source: Nat Commun. 2023 Oct 31;14:6652. doi: 10.1038/s41467-023-42251-0 (PMC10618186; doi:10.1038/s41467-023-42251-0)
Supplement: Supplementary file 4 — Supplementary Code 1-8 [file 41467_2023_42251_MOESM4_ESM.zip › Supplementary Codes 1-8/Supplementary Code 8.rtf]

Supplementary Code 8: Reconstruction of ancestral areas of some key nodes (run)                               MrBayes 3.2.7b x86_64                       (Bayesian Analysis of Phylogeny)               Distributed under the GNU General Public License                 Type "help" or "help <command>" for information                     on the commands that are available.                    Type "about" for authorship and general                       information about the program.      Executing file "location.nex"   UNIX line termination   Longest line length = 1006   Parsing file   Expecting NEXUS formatted file   Reading data block      Allocated taxon set      Allocated matrix      Defining new matrix with 25 taxa and 1 characters      Data is Standard      Gaps coded as -      Missing data coded as ?      Taxon  1 -> Geotria_australis      Taxon  2 -> Mordacia_lapicida      Taxon  3 -> Mordacia_mordax      Taxon  4 -> Lethenteron_camtschaticum      Taxon  5 -> Eudontomyzon_morii      Taxon  6 -> Eudontomyzon_danfordi      Taxon  7 -> Lampetra_fluviatilis      Taxon  8 -> Lampetra_ayresii      Taxon  9 -> Tetrapleurodon_spadiceus      Taxon 10 -> Entosphenus_tridentatus      Taxon 11 -> Entosphenus_macrostomus      Taxon 12 -> Entosphenus_minimus      Taxon 13 -> Entosphenus_similis      Taxon 14 -> Ichthyomyzon_bdellium      Taxon 15 -> Ichthyomyzon_castaneus      Taxon 16 -> Ichthyomyzon_unicuspis      Taxon 17 -> Petromyzon_marinus      Taxon 18 -> Caspiomyzon_wagneri      Taxon 19 -> Mesomyzon_mengae      Taxon 20 -> Yanliaomyzon_occior      Taxon 21 -> Yanliaomyzon_ingensdentes      Taxon 22 -> Hardistiella      Taxon 23 -> Mayomyzon      Taxon 24 -> Pipiscius      Taxon 25 -> Priscomyzon      Successfully read matrix      Matrix contains polymorphisms, interpreted as ambiguity      Setting default partition (does not divide up characters)      Setting model defaults      Seed (for generating default start values) = 1679040803      Setting output file names to "location.nex.run<i>.<p|t>"   Exiting data block   Reading trees block      Successfully read tree 'con_all_compat'   Exiting trees block   Reading mrbayes block      Enabling Coding Variable      Successfully set likelihood model parameters      Setting Clockratepr to Gamma(2.00,200.00)      Successfully set prior model parameters      Setting age of taxon 'Mesomyzon_mengae' to Fixed(125.00)      Setting age of taxon 'Yanliaomyzon_occior' to Fixed(158.00)      Setting age of taxon 'Yanliaomyzon_ingensdentes' to Fixed(163.00)      Setting age of taxon 'Mayomyzon' to Fixed(310.00)      Setting age of taxon 'Pipiscius' to Fixed(310.00)      Setting age of taxon 'Hardistiella' to Fixed(320.00)      Setting age of taxon 'Priscomyzon' to Fixed(360.00)      Setting Nodeagepr to Calibrated      Successfully set prior model parameters      Setting Brlenspr to Clock:Uniform      Successfully set prior model parameters      Setting Treeagepr to Offsetexponential(300.00,390.00)      Successfully set prior model parameters      Defining constraint called 'A'      Defining constraint called 'B'      Defining constraint called 'C'      Defining constraint called 'D'      Defining constraint called 'E'      Defining constraint called 'F'      Defining constraint called 'G'      Setting Topologypr to Constraints      Successfully set prior model parameters      Reporting ancestral states (if applicable)      Setting number of chains to 1      Setting number of generations to 1000000      Setting sample frequency to 100      Setting print frequency to 10000      Setting diagnosing frequency to 50000      Successfully set chain parameters      Successfully set proposal parameters      Successfully set proposal parameters      Successfully set proposal parameters      Successfully set proposal parameters      Successfully set proposal parameters      Successfully set starting values      Running Markov chain      MCMC stamp = 7498101375      Seed = 798677753      Swapseed = 1679040803      Model settings:          Data not partitioned --            Datatype  = Standard            Coding    = Variable            # States  = Variable, up to 24                        State frequencies are fixed to be equal            Rates     = Equal       Active parameters:           Parameters         ---------------------         Statefreq           1         Ratemultiplier      2         Topology            3         Brlens              4         Clockrate           5         ---------------------          1 --  Parameter  = Alpha_symdir               Type       = Symmetric diricihlet/beta distribution alpha_i parameter               Prior      = Symmetric dirichlet with all parameters fixed to infinity          2 --  Parameter  = Ratemultiplier               Type       = Partition-specific rate multiplier               Prior      = Fixed(1.0)          3 --  Parameter  = Tau               Type       = Topology               Prior      = Prior on topology obeys the following constraints:                            -- Hard constraint "A"                            -- Hard constraint "B"                            -- Hard constraint "C"                            -- Hard constraint "D"                            -- Hard constraint "E"                            -- Hard constraint "F"                            -- Hard constraint "G"               Subparam.  = V          4 --  Parameter  = V               Type       = Branch lengths               Prior      = Clock:Uniform                            Node depths are constrained by the following age constraints:                            -- The age of terminal "Mesomyzon_mengae" is Fixed(125.00)                            -- The age of terminal "Yanliaomyzon_occior" is Fixed(158.00)                            -- The age of terminal "Yanliaomyzon_ingensdentes" is Fixed(163.00)                            -- The age of terminal "Hardistiella" is Fixed(320.00)                            -- The age of terminal "Mayomyzon" is Fixed(310.00)                            -- The age of terminal "Pipiscius" is Fixed(310.00)                            -- The age of terminal "Priscomyzon" is Fixed(360.00)                            -- Tree age has a Offsetexponential(300.00,390.00) distribution          5 --  Parameter  = Clockrate               Type       = Base rate of clock               Prior      = Gamma(2.00,200.00)                            The clock rate is constant (strict clock)         The MCMC sampler will use the following moves:         With prob.  Chain will use move          100.00 %   Multiplier(Clockrate)       Division 1 has 3 unique site patterns      Initializing conditional likelihoods      Using standard non-SSE likelihood calculator for division 1 (single-precision)       Initial log likelihoods and log prior probs for run 1:         Chain 1 -- -50.816354 -- -290.793831       Initial log likelihoods and log prior probs for run 2:         Chain 1 -- -50.816354 -- -290.793831       Overwriting file "location.nex.mcmc"      Overwriting file "location.nex.run1.p"      Overwriting file "location.nex.run1.t"      Overwriting file "location.nex.run2.p"      Overwriting file "location.nex.run2.t"       Using a relative burnin of 25.0 % for diagnostics       Chain results (1000000 generations requested):           0 -- -50.816 * -50.816       10000 -- -41.901 * -41.609 -- 0:01:39      20000 -- -42.341 * -41.774 -- 0:00:49      30000 -- -41.421 * -41.893 -- 0:00:32      40000 -- -41.285 * -41.695 -- 0:00:24      50000 -- -41.397 * -41.479 -- 0:00:19       Average standard deviation of split frequencies: 0.000000       60000 -- -42.459 * -42.195 -- 0:00:31      70000 -- -42.754 * -42.131 -- 0:00:26      80000 -- -41.592 * -42.197 -- 0:00:23      90000 -- -46.080 * -41.930 -- 0:00:20      100000 -- -42.893 * -43.403 -- 0:00:18       Average standard deviation of split frequencies: 0.000000       110000 -- -41.143 * -41.180 -- 0:00:24      120000 -- -41.227 * -42.765 -- 0:00:22      130000 -- -41.431 * -41.237 -- 0:00:20      140000 -- -44.283 * -41.157 -- 0:00:18      150000 -- -44.239 * -42.213 -- 0:00:17       Average standard deviation of split frequencies: 0.000000       160000 -- -42.013 * -41.727 -- 0:00:15      170000 -- -41.329 * -41.538 -- 0:00:19      180000 -- -41.623 * -41.766 -- 0:00:18      190000 -- -41.165 * -41.217 -- 0:00:17      200000 -- -41.495 * -41.642 -- 0:00:16       Average standard deviation of split frequencies: 0.000000       210000 -- -41.185 * -41.560 -- 0:00:15      220000 -- -41.469 * -41.352 -- 0:00:17      230000 -- -41.156 * -44.774 -- 0:00:16      240000 -- -41.143 * -42.275 -- 0:00:15      250000 -- -41.156 * -43.653 -- 0:00:15       Average standard deviation of split frequencies: 0.000000       260000 -- -41.339 * -45.046 -- 0:00:14      270000 -- -42.145 * -42.947 -- 0:00:16      280000 -- -43.131 * -42.747 -- 0:00:15      290000 -- -41.419 * -42.475 -- 0:00:14      300000 -- -41.785 * -41.385 -- 0:00:14       Average standard deviation of split frequencies: 0.000000       310000 -- -41.363 * -41.202 -- 0:00:13      320000 -- -41.161 * -41.179 -- 0:00:14      330000 -- -44.422 * -41.519 -- 0:00:14      340000 -- -41.577 * -41.657 -- 0:00:13      350000 -- -41.170 * -41.160 -- 0:00:13       Average standard deviation of split frequencies: 0.000000       360000 -- -41.150 * -41.195 -- 0:00:12      370000 -- -41.904 * -41.152 -- 0:00:13      380000 -- -43.375 * -41.622 -- 0:00:13      390000 -- -41.859 * -41.292 -- 0:00:12      400000 -- -43.300 * -42.609 -- 0:00:12       Average standard deviation of split frequencies: 0.000000       410000 -- -41.399 * -42.522 -- 0:00:11      420000 -- -41.159 * -42.778 -- 0:00:11      430000 -- -42.516 * -41.807 -- 0:00:11      440000 -- -42.499 * -41.149 -- 0:00:11      450000 -- -41.424 * -42.993 -- 0:00:11       Average standard deviation of split frequencies: 0.000000       460000 -- -42.344 * -43.782 -- 0:00:10      470000 -- -41.468 * -41.876 -- 0:00:10      480000 -- -41.211 * -41.166 -- 0:00:10      490000 -- -41.966 * -41.910 -- 0:00:10      500000 -- -41.456 * -41.149 -- 0:00:10       Average standard deviation of split frequencies: 0.000000       510000 -- -41.617 * -42.019 -- 0:00:09      520000 -- -41.147 * -42.942 -- 0:00:09      530000 -- -41.581 * -41.146 -- 0:00:08      540000 -- -41.274 * -41.310 -- 0:00:09      550000 -- -41.171 * -41.185 -- 0:00:09       Average standard deviation of split frequencies: 0.000000       560000 -- -41.623 * -41.359 -- 0:00:08      570000 -- -42.245 * -41.294 -- 0:00:08      580000 -- -41.176 * -41.887 -- 0:00:07      590000 -- -43.146 * -41.192 -- 0:00:08      600000 -- -41.205 * -41.238 -- 0:00:08       Average standard deviation of split frequencies: 0.000000       610000 -- -41.191 * -41.154 -- 0:00:07      620000 -- -41.638 * -41.149 -- 0:00:07      630000 -- -41.313 * -41.837 -- 0:00:07      640000 -- -41.161 * -42.597 -- 0:00:07      650000 -- -41.258 * -41.889 -- 0:00:07       Average standard deviation of split frequencies: 0.000000       660000 -- -42.042 * -41.848 -- 0:00:06      670000 -- -41.250 * -41.736 -- 0:00:06      680000 -- -41.522 * -41.522 -- 0:00:06      690000 -- -41.498 * -41.165 -- 0:00:06      700000 -- -41.330 * -42.145 -- 0:00:06       Average standard deviation of split frequencies: 0.000000       710000 -- -43.354 * -41.151 -- 0:00:05      720000 -- -41.868 * -41.565 -- 0:00:05      730000 -- -42.034 * -41.335 -- 0:00:05      740000 -- -41.945 * -42.449 -- 0:00:04      750000 -- -41.536 * -41.568 -- 0:00:05       Average standard deviation of split frequencies: 0.000000       760000 -- -41.146 * -42.401 -- 0:00:04      770000 -- -41.174 * -41.173 -- 0:00:04      780000 -- -41.446 * -41.686 -- 0:00:04      790000 -- -41.144 * -41.328 -- 0:00:03      800000 -- -42.149 * -44.396 -- 0:00:04       Average standard deviation of split frequencies: 0.000000       810000 -- -42.035 * -42.216 -- 0:00:03      820000 -- -41.844 * -41.579 -- 0:00:03      830000 -- -42.355 * -42.178 -- 0:00:03      840000 -- -41.155 * -41.840 -- 0:00:03      850000 -- -42.038 * -41.456 -- 0:00:03       Average standard deviation of split frequencies: 0.000000       860000 -- -41.408 * -41.543 -- 0:00:02      870000 -- -42.726 * -41.402 -- 0:00:02      880000 -- -41.595 * -44.851 -- 0:00:02      890000 -- -41.526 * -41.639 -- 0:00:02      900000 -- -41.335 * -42.881 -- 0:00:02       Average standard deviation of split frequencies: 0.000000       910000 -- -41.566 * -43.659 -- 0:00:01      920000 -- -41.329 * -41.278 -- 0:00:01      930000 -- -41.290 * -42.063 -- 0:00:01      940000 -- -41.551 * -42.012 -- 0:00:01      950000 -- -41.907 * -41.764 -- 0:00:01       Average standard deviation of split frequencies: 0.000000       960000 -- -41.334 * -41.873 -- 0:00:00      970000 -- -43.627 * -41.155 -- 0:00:00      980000 -- -42.319 * -42.311 -- 0:00:00      990000 -- -42.165 * -41.151 -- 0:00:00      1000000 -- -41.444 * -41.148 -- 0:00:00       Average standard deviation of split frequencies: 0.000000       Analysis completed in 20 seconds      Analysis used 19.09 seconds of CPU time      Likelihood of best state for run 1 was -41.14      Likelihood of best state for run 2 was -41.14      Acceptance rates for the moves in run 1:         With prob.   (last 1000)   chain accepted proposals by move            29.5 %     ( 23 %)     Multiplier(Clockrate)      Acceptance rates for the moves in run 2:         With prob.   (last 1000)   chain accepted proposals by move            29.5 %     ( 27 %)     Multiplier(Clockrate)      Summarizing parameters in files location.nex.run1.p and location.nex.run2.p      Writing summary statistics to file location.nex.pstat      Using relative burnin ('relburnin=yes'), discarding the first 25 % of samples       Below are rough plots of the generation (x-axis) versus the log         probability of observing the data (y-axis). You can use these           graphs to determine what the burn in for your analysis should be.       When the log probability starts to plateau you may be at station-       arity. Sample trees and parameters after the log probability            plateaus. Of course, this is not a guarantee that you are at sta-       tionarity. Also examine the convergence diagnostics provided by         the 'sump' and 'sumt' commands for all the parameters in your           model. Remember that the burn in is the number of samples to dis-       card. There are a total of ngen / samplefreq samples taken during       a MCMC analysis.                                                         Overlay plot for both runs:      (1 = Run number 1; 2 = Run number 2; * = Both runs)       +------------------------------------------------------------+ -41.72      |                                    1                       |      |                                               2            |      |  2  1           1   2      1                               |      |         1  1     2       2    2 1       2   2   2      1  1|      |   1 2                         1    2                      2|      |           1  1   1   2    1    2 2    1        2 1   1     |      | 1 2  2 2    12 1   1   1 1  *     2   2   12      * 2      |      |  1    *  2 22 2   221        1         2                2* |      |22  2                 12             2    1  1*1     121    |      |        1 12                     2   1* 1 2 1   1           |      |1        2     1        2  2    1  1     1       1  *       |      |                 2       2  2                           21  |      |      1         2             2            2                |      |    1              1   1                                    |      |                         1        1               2    2    |      +------+-----+-----+-----+-----+-----+-----+-----+-----+-----+ -42.07      ^                                                            ^      250000                                                       1000000       Overwriting file "location.nex.lstat"       Estimated marginal likelihoods for runs sampled in files         "location.nex.run1.p" and "location.nex.run2.p":         (Use the harmonic mean for Bayes factor comparisons of models)          (Values are saved to the file location.nex.lstat)       Run   Arithmetic mean   Harmonic mean      --------------------------------------        1        -41.66           -42.73        2        -41.66           -42.77      --------------------------------------      TOTAL      -41.66           -42.75      --------------------------------------        Model parameter summaries over the runs sampled in files         "location.nex.run1.p" and "location.nex.run2.p":         Summaries are based on a total of 15002 samples from 2 runs.         Each run produced 10001 samples of which 7501 samples were included.         Parameter summaries saved to file "location.nex.pstat".      Overwriting file "location.nex.pstat"                                             95% HPD Interval                                          --------------------      Parameter      Mean      Variance     Lower       Upper       Median    min ESS*  avg ESS    PSRF+       --------------------------------------------------------------------------------------------------      TH          8.115959    7.859666    3.337114   13.718340    7.719811   7492.74   7496.87    1.000      TL         16.575880   32.785182    6.815659   28.018080   15.766790   7492.74   7496.87    1.000      clockrate   0.020804    0.000052    0.008554    0.035165    0.019788   7492.74   7496.87    1.000      p(0){1@A}   0.013825    0.000110    0.000763    0.035085    0.010921   7460.53   7480.77    1.000      p(1){1@A}   0.013825    0.000110    0.000763    0.035085    0.010921   7460.53   7480.77    1.000      p(2){1@A}   0.013825    0.000110    0.000763    0.035085    0.010921   7460.53   7480.77    1.000      p(3){1@A}   0.013825    0.000110    0.000763    0.035085    0.010921   7460.53   7480.77    1.000      p(4){1@A}   0.013825    0.000110    0.000763    0.035085    0.010921   7460.53   7480.77    1.000      p(5){1@A}   0.885078    0.006799    0.716086    0.987830    0.907888   7461.33   7481.16    1.000      p(6){1@A}   0.013825    0.000110    0.000763    0.035085    0.010921   7460.53   7480.77    1.000      p(7){1@A}   0.013825    0.000110    0.000763    0.035085    0.010921   7460.53   7480.77    1.000      p(8){1@A}   0.018145    0.000085    0.006011    0.037227    0.015674   7467.98   7484.49    1.000      p(0){1@B}   0.000000    0.000000    0.000000    0.000000    0.000000   7501.00   7501.00    1.000      p(1){1@B}   0.000000    0.000000    0.000000    0.000000    0.000000   7501.00   7501.00    1.000      p(2){1@B}   0.000000    0.000000    0.000000    0.000000    0.000000   7501.00   7501.00    1.000      p(3){1@B}   0.000000    0.000000    0.000000    0.000000    0.000000   7501.00   7501.00    1.000      p(4){1@B}   0.000000    0.000000    0.000000    0.000000    0.000000   7501.00   7501.00    1.000      p(5){1@B}   1.000000    0.000000    1.000000    1.000000    1.000000   7501.00   7501.00    1.000      p(6){1@B}   0.000000    0.000000    0.000000    0.000000    0.000000   7501.00   7501.00    1.000      p(7){1@B}   0.000000    0.000000    0.000000    0.000000    0.000000   7501.00   7501.00    1.000      p(8){1@B}   0.000000    0.000000    0.000000    0.000000    0.000000   7501.00   7501.00    1.000      p(0){1@C}   0.053408    0.000383    0.017118    0.090734    0.052506   7479.98   7490.49    1.000      p(1){1@C}   0.195382    0.001971    0.120323    0.281824    0.189667   7501.00   7501.00    1.000      p(2){1@C}   0.054977    0.000390    0.017919    0.092240    0.054228   7482.47   7491.73    1.000      p(3){1@C}   0.091352    0.000161    0.067236    0.111307    0.093444   7501.00   7501.00    1.000      p(4){1@C}   0.129948    0.000090    0.109743    0.137177    0.133600   7501.00   7501.00    1.000      p(5){1@C}   0.143065    0.000074    0.124969    0.151015    0.146200   7468.66   7484.83    1.000      p(6){1@C}   0.083080    0.000224    0.054703    0.109175    0.084595   7501.00   7501.00    1.000      p(7){1@C}   0.195382    0.001971    0.120323    0.281824    0.189667   7501.00   7501.00    1.000      p(8){1@C}   0.053408    0.000383    0.017118    0.090734    0.052506   7479.98   7490.49    1.000      p(0){1@D}   0.044685    0.000305    0.013006    0.078551    0.043307   7472.36   7486.68    1.000      p(1){1@D}   0.193025    0.001841    0.120747    0.277065    0.187228   7501.00   7501.00    1.000      p(2){1@D}   0.047552    0.000325    0.014202    0.082045    0.046342   7474.83   7487.92    1.000      p(3){1@D}   0.111966    0.000119    0.089538    0.122379    0.115481   7501.00   7501.00    1.000      p(4){1@D}   0.180677    0.000147    0.155408    0.190005    0.185426   6517.39   7009.20    1.000      p(5){1@D}   0.086934    0.000016    0.077393    0.093578    0.088057   7501.00   7501.00    1.000      p(6){1@D}   0.097449    0.000182    0.071560    0.116188    0.099888   7501.00   7501.00    1.000      p(7){1@D}   0.193025    0.001841    0.120747    0.277065    0.187228   7501.00   7501.00    1.000      p(8){1@D}   0.044685    0.000305    0.012994    0.078546    0.043307   7472.36   7486.68    1.000      p(0){1@E}   0.015316    0.000035    0.005393    0.026897    0.014389   7488.97   7494.99    1.000      p(1){1@E}   0.032208    0.000013    0.029101    0.039871    0.030862   6724.43   7112.72    1.000      p(2){1@E}   0.024647    0.000074    0.009745    0.041767    0.023503   7481.45   7491.22    1.000      p(3){1@E}   0.229007    0.000073    0.212203    0.240117    0.231128   7475.84   7488.42    1.000      p(4){1@E}   0.447926    0.000172    0.421897    0.458901    0.452348   7501.00   7501.00    1.000      p(5){1@E}   0.020099    0.000016    0.013591    0.028520    0.019341   7501.00   7501.00    1.000      p(6){1@E}   0.183273    0.000003    0.179857    0.184679    0.183877   6543.30   7022.15    1.000      p(7){1@E}   0.032208    0.000013    0.029101    0.039871    0.030862   6724.43   7112.71    1.000      p(8){1@E}   0.015316    0.000035    0.005393    0.026897    0.014389   7488.97   7494.99    1.000      p(0){1@F}   0.001278    0.000001    0.000096    0.003159    0.001009   7501.00   7501.00    1.000      p(1){1@F}   0.001808    0.000001    0.000742    0.003496    0.001574   7501.00   7501.00    1.000      p(2){1@F}   0.020096    0.000051    0.008090    0.034447    0.019044   7488.17   7494.58    1.000      p(3){1@F}   0.099444    0.000004    0.095408    0.101948    0.099928   7431.06   7466.03    1.000      p(4){1@F}   0.865495    0.000151    0.841439    0.885359    0.867649   7501.00   7501.00    1.000      p(5){1@F}   0.001431    0.000001    0.000221    0.003262    0.001185   7501.00   7501.00    1.000      p(6){1@F}   0.007363    0.000006    0.002970    0.012373    0.007030   7493.76   7497.38    1.000      p(7){1@F}   0.001808    0.000001    0.000742    0.003496    0.001574   7501.00   7501.00    1.000      p(8){1@F}   0.001278    0.000001    0.000096    0.003159    0.001009   7501.00   7501.00    1.000      p(0){1@G}   0.007505    0.000009    0.002669    0.013392    0.006993   7500.20   7500.60    1.000      p(1){1@G}   0.010753    0.000003    0.009690    0.013946    0.010119   7501.00   7501.00    1.000      p(2){1@G}   0.009265    0.000012    0.003432    0.015993    0.008732   7494.57   7497.79    1.000      p(3){1@G}   0.416611    0.000215    0.387481    0.440530    0.419101   7486.76   7493.88    1.000      p(4){1@G}   0.089816    0.000007    0.084612    0.092470    0.090577   7469.18   7485.09    1.000      p(5){1@G}   0.008423    0.000007    0.004197    0.013610    0.007938   7501.00   7501.00    1.000      p(6){1@G}   0.439368    0.000007    0.434634    0.443395    0.439591   7428.74   7464.87    1.000      p(7){1@G}   0.010753    0.000003    0.009690    0.013946    0.010119   7501.00   7501.00    1.000      p(8){1@G}   0.007505    0.000009    0.002669    0.013392    0.006993   7500.20   7500.60    1.000      --------------------------------------------------------------------------------------------------      * Convergence diagnostic (ESS = Estimated Sample Size); min and avg values        correspond to minimal and average ESS among runs.         ESS value below 100 may indicate that the parameter is undersampled.       + Convergence diagnostic (PSRF = Potential Scale Reduction Factor; Gelman        and Rubin, 1992) should approach 1.0 as runs converge.     Exiting mrbayes block   Reached end of file    Tasks completed, exiting program because mode is noninteractive   To return control to the command line after completion of file processing,    set mode to interactive with 'mb -i <filename>' (i is for interactive)   or use 'set mode=interactive'    
